# Supplementary material for: Isolation, identification, and whole-genome sequencing of high-yield protease bacteria from Daqu of ZhangGong Laojiu
Source: PLoS One. 2022 Apr 26;17(4):e0264677. doi: 10.1371/journal.pone.0264677 (PMC9041807; doi:10.1371/journal.pone.0264677)
Supplement: S1 Raw data — (ZIP) [file pone.0264677.s002.zip › Raw data/GDD20120317-1_Bacillus_velezensis_Genome_result/4_Basic_Annot/KEGG/Bac.htm]

Bac


Bac Pathway Enrichment

| # | Pathway | Count (1209) | Pathway ID |
| 1 | Metabolic pathways | 667 | ko01100 |
| 2 | Biosynthesis of secondary metabolites | 316 | ko01110 |
| 3 | Microbial metabolism in diverse environments | 168 | ko01120 |
| 4 | Two-component system | 124 | ko02020 |
| 5 | Biosynthesis of amino acids | 117 | ko01230 |
| 6 | ABC transporters | 110 | ko02010 |
| 7 | Carbon metabolism | 91 | ko01200 |
| 8 | Quorum sensing | 66 | ko02024 |
| 9 | Ribosome | 59 | ko03010 |
| 10 | Purine metabolism | 50 | ko00230 |
| 11 | Pyruvate metabolism | 44 | ko00620 |
| 12 | Cysteine and methionine metabolism | 42 | ko00270 |
| 13 | Amino sugar and nucleotide sugar metabolism | 40 | ko00520 |
| 14 | Glycolysis / Gluconeogenesis | 38 | ko00010 |
| 15 | Starch and sucrose metabolism | 36 | ko00500 |
| 16 | Alanine, aspartate and glutamate metabolism | 35 | ko00250 |
| 17 | Propanoate metabolism | 35 | ko00640 |
| 18 | Oxidative phosphorylation | 34 | ko00190 |
| 19 | Pyrimidine metabolism | 32 | ko00240 |
| 20 | Glycine, serine and threonine metabolism | 32 | ko00260 |
| 21 | Glyoxylate and dicarboxylate metabolism | 32 | ko00630 |
| 22 | Fatty acid metabolism | 32 | ko01212 |
| 23 | Flagellar assembly | 32 | ko02040 |
| 24 | Peptidoglycan biosynthesis | 29 | ko00550 |
| 25 | Fatty acid biosynthesis | 28 | ko00061 |
| 26 | 2-Oxocarboxylic acid metabolism | 27 | ko01210 |
| 27 | Aminoacyl-tRNA biosynthesis | 26 | ko00970 |
| 28 | Butanoate metabolism | 25 | ko00650 |
| 29 | Pentose phosphate pathway | 24 | ko00030 |
| 30 | Valine, leucine and isoleucine degradation | 24 | ko00280 |
| 31 | Carbon fixation pathways in prokaryotes | 24 | ko00720 |
| 32 | Arginine and proline metabolism | 23 | ko00330 |
| 33 | Folate biosynthesis | 23 | ko00790 |
| 34 | Phosphotransferase system (PTS) | 23 | ko02060 |
| 35 | Citrate cycle (TCA cycle) | 22 | ko00020 |
| 36 | Arginine biosynthesis | 21 | ko00220 |
| 37 | Phenylalanine, tyrosine and tryptophan biosynthesis | 21 | ko00400 |
| 38 | Methane metabolism | 21 | ko00680 |
| 39 | Porphyrin and chlorophyll metabolism | 21 | ko00860 |
| 40 | Bacterial chemotaxis | 21 | ko02030 |
| 41 | Galactose metabolism | 20 | ko00052 |
| 42 | Glycerophospholipid metabolism | 20 | ko00564 |
| 43 | Pantothenate and CoA biosynthesis | 20 | ko00770 |
| 44 | Fructose and mannose metabolism | 19 | ko00051 |
| 45 | Thiamine metabolism | 19 | ko00730 |
| 46 | Biotin metabolism | 19 | ko00780 |
| 47 | Protein export | 19 | ko03060 |
| 48 | Homologous recombination | 19 | ko03440 |
| 49 | Nitrogen metabolism | 18 | ko00910 |
| 50 | Mismatch repair | 18 | ko03430 |
| 51 | Pentose and glucuronate interconversions | 17 | ko00040 |
| 52 | Fatty acid degradation | 17 | ko00071 |
| 53 | Lysine biosynthesis | 17 | ko00300 |
| 54 | Histidine metabolism | 17 | ko00340 |
| 55 | Glycerolipid metabolism | 17 | ko00561 |
| 56 | Lysine degradation | 16 | ko00310 |
| 57 | Sulfur metabolism | 16 | ko00920 |
| 58 | DNA replication | 16 | ko03030 |
| 59 | Nicotinate and nicotinamide metabolism | 15 | ko00760 |
| 60 | Terpenoid backbone biosynthesis | 15 | ko00900 |
| 61 | RNA degradation | 15 | ko03018 |
| 62 | Tryptophan metabolism | 14 | ko00380 |
| 63 | Inositol phosphate metabolism | 14 | ko00562 |
| 64 | Bacterial secretion system | 14 | ko03070 |
| 65 | Valine, leucine and isoleucine biosynthesis | 13 | ko00290 |
| 66 | Cell cycle - Caulobacter | 13 | ko04112 |
| 67 | Sulfur relay system | 13 | ko04122 |
| 68 | Ubiquinone and other terpenoid-quinone biosynthesis | 12 | ko00130 |
| 69 | One carbon pool by folate | 12 | ko00670 |
| 70 | Selenocompound metabolism | 11 | ko00450 |
| 71 | Carbon fixation in photosynthetic organisms | 11 | ko00710 |
| 72 | Base excision repair | 11 | ko03410 |
| 73 | Prodigiosin biosyntheses | 10 | ko00333 |
| 74 | Riboflavin metabolism | 10 | ko00740 |
| 75 | Nonribosomal peptide structures | 10 | ko01054 |
| 76 | Degradation of aromatic compounds | 10 | ko01220 |
| 77 | Monobactam biosynthesis | 9 | ko00261 |
| 78 | Benzoate degradation | 9 | ko00362 |
| 79 | Glutathione metabolism | 9 | ko00480 |
| 80 | Streptomycin biosynthesis | 9 | ko00521 |
| 81 | Aminobenzoate degradation | 9 | ko00627 |
| 82 | C5-Branched dibasic acid metabolism | 9 | ko00660 |
| 83 | Nucleotide excision repair | 9 | ko03420 |
| 84 | Biofilm formation - Vibrio cholerae | 9 | ko05111 |
| 85 | Synthesis and degradation of ketone bodies | 8 | ko00072 |
| 86 | Photosynthesis | 8 | ko00195 |
| 87 | Tyrosine metabolism | 8 | ko00350 |
| 88 | Drug metabolism - other enzymes | 8 | ko00983 |
| 89 | Biosynthesis of various secondary metabolites - part 2 | 8 | ko00998 |
| 90 | Phenylalanine metabolism | 7 | ko00360 |
| 91 | beta-Alanine metabolism | 7 | ko00410 |
| 92 | Taurine and hypotaurine metabolism | 7 | ko00430 |
| 93 | Chloroalkane and chloroalkene degradation | 7 | ko00625 |
| 94 | Biofilm formation - Escherichia coli | 7 | ko02026 |
| 95 | Ascorbate and aldarate metabolism | 6 | ko00053 |
| 96 | D-Glutamine and D-glutamate metabolism | 6 | ko00471 |
| 97 | D-Alanine metabolism | 6 | ko00473 |
| 98 | Vitamin B6 metabolism | 5 | ko00750 |
| 99 | Biosynthesis of siderophore group nonribosomal peptides | 5 | ko01053 |
| 100 | RNA polymerase | 5 | ko03020 |
| 101 | Novobiocin biosynthesis | 4 | ko00401 |
| 102 | Cyanoamino acid metabolism | 4 | ko00460 |
| 103 | Polyketide sugar unit biosynthesis | 4 | ko00523 |
| 104 | Lipoic acid metabolism | 4 | ko00785 |
| 105 | Biofilm formation - Pseudomonas aeruginosa | 4 | ko02025 |
| 106 | Plant-pathogen interaction | 4 | ko04626 |
| 107 | Penicillin and cephalosporin biosynthesis | 3 | ko00311 |
| 108 | Carbapenem biosynthesis | 3 | ko00332 |
| 109 | Naphthalene degradation | 3 | ko00626 |
| 110 | Atrazine degradation | 3 | ko00791 |
| 111 | Limonene and pinene degradation | 3 | ko00903 |
| 112 | NOD-like receptor signaling pathway | 3 | ko04621 |
| 113 | Geraniol degradation | 2 | ko00281 |
| 114 | Chlorocyclohexane and chlorobenzene degradation | 2 | ko00361 |
| 115 | Other glycan degradation | 2 | ko00511 |
| 116 | Acarbose and validamycin biosynthesis | 2 | ko00525 |
| 117 | Sphingolipid metabolism | 2 | ko00600 |
| 118 | Xylene degradation | 2 | ko00622 |
| 119 | Non-homologous end-joining | 2 | ko03450 |
| 120 | Phenazine biosynthesis | 1 | ko00405 |
| 121 | Phosphonate and phosphinate metabolism | 1 | ko00440 |
| 122 | D-Arginine and D-ornithine metabolism | 1 | ko00472 |
| 123 | Neomycin, kanamycin and gentamicin biosynthesis | 1 | ko00524 |
| 124 | Arabinogalactan biosynthesis - Mycobacterium | 1 | ko00572 |
| 125 | Arachidonic acid metabolism | 1 | ko00590 |
| 126 | alpha-Linolenic acid metabolism | 1 | ko00592 |
| 127 | Dioxin degradation | 1 | ko00621 |
| 128 | Ethylbenzene degradation | 1 | ko00642 |
| 129 | Styrene degradation | 1 | ko00643 |
| 130 | Carotenoid biosynthesis | 1 | ko00906 |
| 131 | Biosynthesis of ansamycins | 1 | ko01051 |
| 132 | Biosynthesis of vancomycin group antibiotics | 1 | ko01055 |

Pathway Detail

| # | Pathway | Pathway ID | K\_id | genes | desc |
| 1 | Metabolic pathways | ko01100 || K00005 | KSG66\_02915 | gldA; glycerol dehydrogenase [EC:1.1.1.6] |
| K00008 | KSG66\_03200 | SORD, gutB; L-iditol 2-dehydrogenase [EC:1.1.1.14] |
| K00009 | KSG66\_02115 | mtlD; mannitol-1-phosphate 5-dehydrogenase [EC:1.1.1.17] |
| K00010 | KSG66\_18720 | iolG; myo-inositol 2-dehydrogenase / D-chiro-inositol 1-dehydrogenase [EC:1.1.1.18 1.1.1.369] |
| K00012 | KSG66\_16760 | UGDH, ugd; UDPglucose 6-dehydrogenase [EC:1.1.1.22] |
| K00012 | KSG66\_17100 | UGDH, ugd; UDPglucose 6-dehydrogenase [EC:1.1.1.22] |
| K00013 | KSG66\_16445 | hisD; histidinol dehydrogenase [EC:1.1.1.23] |
| K00014 | KSG66\_12170 | aroE; shikimate dehydrogenase [EC:1.1.1.25] |
| K00016 | KSG66\_01650 | LDH, ldh; L-lactate dehydrogenase [EC:1.1.1.27] |
| K00019 | KSG66\_09180 | E1.1.1.30, bdh; 3-hydroxybutyrate dehydrogenase [EC:1.1.1.30] |
| K00019 | KSG66\_19245 | E1.1.1.30, bdh; 3-hydroxybutyrate dehydrogenase [EC:1.1.1.30] |
| K00020 | KSG66\_06960 | mmsB, HIBADH; 3-hydroxyisobutyrate dehydrogenase [EC:1.1.1.31] |
| K00024 | KSG66\_13385 | mdh; malate dehydrogenase [EC:1.1.1.37] |
| K00031 | KSG66\_13390 | IDH1, IDH2, icd; isocitrate dehydrogenase [EC:1.1.1.42] |
| K00033 | KSG66\_11260 | PGD, gnd, gntZ; 6-phosphogluconate dehydrogenase [EC:1.1.1.44 1.1.1.343] |
| K00034 | KSG66\_01550 | gdh; glucose 1-dehydrogenase [EC:1.1.1.47] |
| K00034 | KSG66\_02080 | gdh; glucose 1-dehydrogenase [EC:1.1.1.47] |
| K00036 | KSG66\_11255 | G6PD, zwf; glucose-6-phosphate 1-dehydrogenase [EC:1.1.1.49 1.1.1.363] |
| K00041 | KSG66\_06135 | uxaB; tagaturonate reductase [EC:1.1.1.58] |
| K00052 | KSG66\_12970 | leuB, IMDH; 3-isopropylmalate dehydrogenase [EC:1.1.1.85] |
| K00053 | KSG66\_12980 | ilvC; ketol-acid reductoisomerase [EC:1.1.1.86] |
| K00058 | KSG66\_09435 | serA, PHGDH; D-3-phosphoglycerate dehydrogenase / 2-oxoglutarate reductase [EC:1.1.1.95 1.1.1.399] |
| K00058 | KSG66\_10775 | serA, PHGDH; D-3-phosphoglycerate dehydrogenase / 2-oxoglutarate reductase [EC:1.1.1.95 1.1.1.399] |
| K00059 | KSG66\_07950 | fabG; 3-oxoacyl-[acyl-carrier protein] reductase [EC:1.1.1.100] |
| K00059 | KSG66\_08440 | fabG; 3-oxoacyl-[acyl-carrier protein] reductase [EC:1.1.1.100] |
| K00059 | KSG66\_09410 | fabG; 3-oxoacyl-[acyl-carrier protein] reductase [EC:1.1.1.100] |
| K00059 | KSG66\_11195 | fabG; 3-oxoacyl-[acyl-carrier protein] reductase [EC:1.1.1.100] |
| K00059 | KSG66\_13500 | fabG; 3-oxoacyl-[acyl-carrier protein] reductase [EC:1.1.1.100] |
| K00059 | KSG66\_15330 | fabG; 3-oxoacyl-[acyl-carrier protein] reductase [EC:1.1.1.100] |
| K00059 | KSG66\_15750 | fabG; 3-oxoacyl-[acyl-carrier protein] reductase [EC:1.1.1.100] |
| K00060 | KSG66\_08495 | tdh; threonine 3-dehydrogenase [EC:1.1.1.103] |
| K00067 | KSG66\_17890 | rfbD, rmlD; dTDP-4-dehydrorhamnose reductase [EC:1.1.1.133] |
| K00074 | KSG66\_11400 | paaH, hbd, fadB, mmgB; 3-hydroxybutyryl-CoA dehydrogenase [EC:1.1.1.157] |
| K00075 | KSG66\_07625 | murB; UDP-N-acetylmuramate dehydrogenase [EC:1.3.1.98] |
| K00077 | KSG66\_07185 | panE, apbA; 2-dehydropantoate 2-reductase [EC:1.1.1.169] |
| K00077 | KSG66\_07565 | panE, apbA; 2-dehydropantoate 2-reductase [EC:1.1.1.169] |
| K00077 | KSG66\_15355 | panE, apbA; 2-dehydropantoate 2-reductase [EC:1.1.1.169] |
| K00088 | KSG66\_00070 | IMPDH, guaB; IMP dehydrogenase [EC:1.1.1.205] |
| K00090 | KSG66\_16350 | ghrB; glyoxylate/hydroxypyruvate/2-ketogluconate reductase [EC:1.1.1.79 1.1.1.81 1.1.1.215] |
| K00099 | KSG66\_08275 | dxr; 1-deoxy-D-xylulose-5-phosphate reductoisomerase [EC:1.1.1.267] |
| K00104 | KSG66\_13180 | glcD; glycolate oxidase [EC:1.1.3.15] |
| K00121 | KSG66\_01785 | frmA, ADH5, adhC; S-(hydroxymethyl)glutathione dehydrogenase / alcohol dehydrogenase [EC:1.1.1.284 1.1.1.1] |
| K00121 | KSG66\_02865 | frmA, ADH5, adhC; S-(hydroxymethyl)glutathione dehydrogenase / alcohol dehydrogenase [EC:1.1.1.284 1.1.1.1] |
| K00123 | KSG66\_06060 | fdoG, fdhF, fdwA; formate dehydrogenase major subunit [EC:1.17.1.9] |
| K00123 | KSG66\_12455 | fdoG, fdhF, fdwA; formate dehydrogenase major subunit [EC:1.17.1.9] |
| K00128 | KSG66\_09600 | ALDH; aldehyde dehydrogenase (NAD+) [EC:1.2.1.3] |
| K00128 | KSG66\_13620 | ALDH; aldehyde dehydrogenase (NAD+) [EC:1.2.1.3] |
| K00128 | KSG66\_18800 | ALDH; aldehyde dehydrogenase (NAD+) [EC:1.2.1.3] |
| K00130 | KSG66\_14370 | betB, gbsA; betaine-aldehyde dehydrogenase [EC:1.2.1.8] |
| K00133 | KSG66\_08375 | asd; aspartate-semialdehyde dehydrogenase [EC:1.2.1.11] |
| K00134 | KSG66\_13335 | GAPDH, gapA; glyceraldehyde 3-phosphate dehydrogenase [EC:1.2.1.12] |
| K00134 | KSG66\_15990 | GAPDH, gapA; glyceraldehyde 3-phosphate dehydrogenase [EC:1.2.1.12] |
| K00135 | KSG66\_02070 | gabD; succinate-semialdehyde dehydrogenase / glutarate-semialdehyde dehydrogenase [EC:1.2.1.16 1.2.1.79 1.2.1.20] |
| K00140 | KSG66\_18750 | mmsA, iolA, ALDH6A1; malonate-semialdehyde dehydrogenase (acetylating) / methylmalonate-semialdehyde dehydrogenase [EC:1.2.1.18 1.2.1.27] |
| K00145 | KSG66\_05535 | argC; N-acetyl-gamma-glutamyl-phosphate reductase [EC:1.2.1.38] |
| K00147 | KSG66\_06570 | proA; glutamate-5-semialdehyde dehydrogenase [EC:1.2.1.41] |
| K00158 | KSG66\_02275 | E1.2.3.3, poxL; pyruvate oxidase [EC:1.2.3.3] |
| K00161 | KSG66\_07305 | PDHA, pdhA; pyruvate dehydrogenase E1 component alpha subunit [EC:1.2.4.1] |
| K00162 | KSG66\_07310 | PDHB, pdhB; pyruvate dehydrogenase E1 component beta subunit [EC:1.2.4.1] |
| K00164 | KSG66\_09635 | OGDH, sucA; 2-oxoglutarate dehydrogenase E1 component [EC:1.2.4.2] |
| K00166 | KSG66\_11345 | BCKDHA, bkdA1; 2-oxoisovalerate dehydrogenase E1 component alpha subunit [EC:1.2.4.4] |
| K00167 | KSG66\_11340 | BCKDHB, bkdA2; 2-oxoisovalerate dehydrogenase E1 component beta subunit [EC:1.2.4.4] |
| K00208 | KSG66\_05810 | fabI; enoyl-[acyl-carrier protein] reductase I [EC:1.3.1.9 1.3.1.10] |
| K00215 | KSG66\_10485 | dapB; 4-hydroxy-tetrahydrodipicolinate reductase [EC:1.17.1.8] |
| K00231 | KSG66\_05110 | PPOX, hemY; protoporphyrinogen/coproporphyrinogen III oxidase [EC:1.3.3.4 1.3.3.15] |
| K00239 | KSG66\_13060 | sdhA, frdA; succinate dehydrogenase / fumarate reductase, flavoprotein subunit [EC:1.3.5.1 1.3.5.4] |
| K00240 | KSG66\_13055 | sdhB, frdB; succinate dehydrogenase / fumarate reductase, iron-sulfur subunit [EC:1.3.5.1 1.3.5.4] |
| K00241 | KSG66\_13065 | sdhC, frdC; succinate dehydrogenase / fumarate reductase, cytochrome b subunit |
| K00259 | KSG66\_14805 | ald; alanine dehydrogenase [EC:1.4.1.1] |
| K00260 | KSG66\_10725 | gudB, rocG; glutamate dehydrogenase [EC:1.4.1.2] |
| K00260 | KSG66\_17855 | gudB, rocG; glutamate dehydrogenase [EC:1.4.1.2] |
| K00263 | KSG66\_11360 | E1.4.1.9; leucine dehydrogenase [EC:1.4.1.9] |
| K00265 | KSG66\_09385 | gltB; glutamate synthase (NADPH) large chain [EC:1.4.1.13] |
| K00266 | KSG66\_09380 | gltD; glutamate synthase (NADPH) small chain [EC:1.4.1.13] |
| K00278 | KSG66\_12765 | nadB; L-aspartate oxidase [EC:1.4.3.16] |
| K00282 | KSG66\_11625 | gcvPA; glycine dehydrogenase subunit 1 [EC:1.4.4.2] |
| K00283 | KSG66\_11620 | gcvPB; glycine dehydrogenase subunit 2 [EC:1.4.4.2] |
| K00285 | KSG66\_15175 | dadA; D-amino-acid dehydrogenase [EC:1.4.5.1] |
| K00286 | KSG66\_09400 | proC; pyrroline-5-carboxylate reductase [EC:1.5.1.2] |
| K00286 | KSG66\_11140 | proC; pyrroline-5-carboxylate reductase [EC:1.5.1.2] |
| K00287 | KSG66\_10125 | DHFR, folA; dihydrofolate reductase [EC:1.5.1.3] |
| K00294 | KSG66\_01730 | E1.2.1.88; 1-pyrroline-5-carboxylate dehydrogenase [EC:1.2.1.88] |
| K00294 | KSG66\_17850 | E1.2.1.88; 1-pyrroline-5-carboxylate dehydrogenase [EC:1.2.1.88] |
| K00318 | KSG66\_01725 | PRODH; proline dehydrogenase [EC:1.5.-.-] |
| K00318 | KSG66\_15300 | PRODH; proline dehydrogenase [EC:1.5.-.-] |
| K00355 | KSG66\_04130 | NQO1; NAD(P)H dehydrogenase (quinone) [EC:1.6.5.2] |
| K00362 | KSG66\_01760 | nirB; nitrite reductase (NADH) large subunit [EC:1.7.1.15] |
| K00362 | KSG66\_01770 | nirB; nitrite reductase (NADH) large subunit [EC:1.7.1.15] |
| K00363 | KSG66\_01755 | nirD; nitrite reductase (NADH) small subunit [EC:1.7.1.15] |
| K00364 | KSG66\_14905 | E1.7.1.7, guaC; GMP reductase [EC:1.7.1.7] |
| K00370 | KSG66\_17605 | narG, narZ, nxrA; nitrate reductase / nitrite oxidoreductase, alpha subunit [EC:1.7.5.1 1.7.99.-] |
| K00371 | KSG66\_17600 | narH, narY, nxrB; nitrate reductase / nitrite oxidoreductase, beta subunit [EC:1.7.5.1 1.7.99.-] |
| K00372 | KSG66\_01765 | nasA; assimilatory nitrate reductase catalytic subunit [EC:1.7.99.-] |
| K00374 | KSG66\_17590 | narI, narV; nitrate reductase gamma subunit [EC:1.7.5.1 1.7.99.-] |
| K00380 | KSG66\_15640 | cysJ; sulfite reductase (NADPH) flavoprotein alpha-component [EC:1.8.1.2] |
| K00381 | KSG66\_15635 | cysI; sulfite reductase (NADPH) hemoprotein beta-component [EC:1.8.1.2] |
| K00382 | KSG66\_04065 | DLD, lpd, pdhD; dihydrolipoamide dehydrogenase [EC:1.8.1.4] |
| K00382 | KSG66\_07320 | DLD, lpd, pdhD; dihydrolipoamide dehydrogenase [EC:1.8.1.4] |
| K00382 | KSG66\_11350 | DLD, lpd, pdhD; dihydrolipoamide dehydrogenase [EC:1.8.1.4] |
| K00390 | KSG66\_07780 | cysH; phosphoadenosine phosphosulfate reductase [EC:1.8.4.8 1.8.4.10] |
| K00432 | KSG66\_10165 | gpx; glutathione peroxidase [EC:1.11.1.9] |
| K00435 | KSG66\_17790 | hemQ; Fe-coproporphyrin III decarboxylase [EC:1.11.1.-] |
| K00459 | KSG66\_14465 | ncd2, npd; nitronate monooxygenase [EC:1.13.12.16] |
| K00483 | KSG66\_01300 | hpaB; 4-hydroxyphenylacetate 3-monooxygenase [EC:1.14.14.9] |
| K00491 | KSG66\_03800 | nos; nitric-oxide synthase, bacterial [EC:1.14.14.47] |
| K00525 | KSG66\_08685 | E1.17.4.1A, nrdA, nrdE; ribonucleoside-diphosphate reductase alpha chain [EC:1.17.4.1] |
| K00526 | KSG66\_08690 | E1.17.4.1B, nrdB, nrdF; ribonucleoside-diphosphate reductase beta chain [EC:1.17.4.1] |
| K00547 | KSG66\_01390 | mmuM, BHMT2; homocysteine S-methyltransferase [EC:2.1.1.10] |
| K00549 | KSG66\_06615 | metE; 5-methyltetrahydropteroyltriglutamate--homocysteine methyltransferase [EC:2.1.1.14] |
| K00549 | KSG66\_18440 | metE; 5-methyltetrahydropteroyltriglutamate--homocysteine methyltransferase [EC:2.1.1.14] |
| K00558 | KSG66\_18550 | DNMT1, dcm; DNA (cytosine-5)-methyltransferase 1 [EC:2.1.1.37] |
| K00560 | KSG66\_10130 | thyA, TYMS; thymidylate synthase [EC:2.1.1.45] |
| K00600 | KSG66\_17420 | glyA, SHMT; glycine hydroxymethyltransferase [EC:2.1.2.1] |
| K00602 | KSG66\_03395 | purH; phosphoribosylaminoimidazolecarboxamide formyltransferase / IMP cyclohydrolase [EC:2.1.2.3 3.5.4.10] |
| K00605 | KSG66\_11630 | gcvT, AMT; aminomethyltransferase [EC:2.1.2.10] |
| K00606 | KSG66\_10455 | panB; 3-methyl-2-oxobutanoate hydroxymethyltransferase [EC:2.1.2.11] |
| K00609 | KSG66\_07740 | pyrB, PYR2; aspartate carbamoyltransferase catalytic subunit [EC:2.1.3.2] |
| K00611 | KSG66\_05565 | OTC, argF, argI; ornithine carbamoyltransferase [EC:2.1.3.3] |
| K00615 | KSG66\_08935 | E2.2.1.1, tktA, tktB; transketolase [EC:2.2.1.1] |
| K00616 | KSG66\_17525 | E2.2.1.2, talA, talB; transaldolase [EC:2.2.1.2] |
| K00620 | KSG66\_05540 | argJ; glutamate N-acetyltransferase / amino-acid N-acetyltransferase [EC:2.3.1.35 2.3.1.1] |
| K00625 | KSG66\_17785 | E2.3.1.8, pta; phosphate acetyltransferase [EC:2.3.1.8] |
| K00626 | KSG66\_05195 | E2.3.1.9, atoB; acetyl-CoA C-acetyltransferase [EC:2.3.1.9] |
| K00626 | KSG66\_11405 | E2.3.1.9, atoB; acetyl-CoA C-acetyltransferase [EC:2.3.1.9] |
| K00627 | KSG66\_04060 | DLAT, aceF, pdhC; pyruvate dehydrogenase E2 component (dihydrolipoamide acetyltransferase) [EC:2.3.1.12] |
| K00627 | KSG66\_07315 | DLAT, aceF, pdhC; pyruvate dehydrogenase E2 component (dihydrolipoamide acetyltransferase) [EC:2.3.1.12] |
| K00632 | KSG66\_15285 | fadA, fadI; acetyl-CoA acyltransferase [EC:2.3.1.16] |
| K00634 | KSG66\_11365 | ptb; phosphate butyryltransferase [EC:2.3.1.19] |
| K00639 | KSG66\_08500 | kbl, GCAT; glycine C-acetyltransferase [EC:2.3.1.29] |
| K00640 | KSG66\_00615 | cysE; serine O-acetyltransferase [EC:2.3.1.30] |
| K00645 | KSG66\_07945 | fabD; [acyl-carrier-protein] S-malonyltransferase [EC:2.3.1.39] |
| K00645 | KSG66\_09175 | fabD; [acyl-carrier-protein] S-malonyltransferase [EC:2.3.1.39] |
| K00648 | KSG66\_05125 | fabH; 3-oxoacyl-[acyl-carrier-protein] synthase III [EC:2.3.1.180] |
| K00648 | KSG66\_05605 | fabH; 3-oxoacyl-[acyl-carrier-protein] synthase III [EC:2.3.1.180] |
| K00651 | KSG66\_10170 | metA; homoserine O-succinyltransferase/O-acetyltransferase [EC:2.3.1.46 2.3.1.31] |
| K00652 | KSG66\_09215 | bioF; 8-amino-7-oxononanoate synthase [EC:2.3.1.47] |
| K00655 | KSG66\_04800 | plsC; 1-acyl-sn-glycerol-3-phosphate acyltransferase [EC:2.3.1.51] |
| K00655 | KSG66\_10120 | plsC; 1-acyl-sn-glycerol-3-phosphate acyltransferase [EC:2.3.1.51] |
| K00657 | KSG66\_02940 | speG, SAT; diamine N-acetyltransferase [EC:2.3.1.57] |
| K00658 | KSG66\_09630 | DLST, sucB; 2-oxoglutarate dehydrogenase E2 component (dihydrolipoamide succinyltransferase) [EC:2.3.1.61] |
| K00681 | KSG66\_09350 | ggt; gamma-glutamyltranspeptidase / glutathione hydrolase [EC:2.3.2.2 3.4.19.13] |
| K00692 | KSG66\_19175 | sacB; levansucrase [EC:2.4.1.10] |
| K00756 | KSG66\_18600 | pdp; pyrimidine-nucleoside phosphorylase [EC:2.4.2.2] |
| K00759 | KSG66\_12665 | APRT, apt; adenine phosphoribosyltransferase [EC:2.4.2.7] |
| K00760 | KSG66\_00415 | hprT, hpt, HPRT1; hypoxanthine phosphoribosyltransferase [EC:2.4.2.8] |
| K00761 | KSG66\_17415 | upp, UPRT; uracil phosphoribosyltransferase [EC:2.4.2.9] |
| K00762 | KSG66\_07775 | pyrE; orotate phosphoribosyltransferase [EC:2.4.2.10] |
| K00763 | KSG66\_14725 | pncB, NAPRT1; nicotinate phosphoribosyltransferase [EC:6.3.4.21] |
| K00764 | KSG66\_03380 | purF, PPAT; amidophosphoribosyltransferase [EC:2.4.2.14] |
| K00765 | KSG66\_16450 | hisG; ATP phosphoribosyltransferase [EC:2.4.2.17] |
| K00766 | KSG66\_10580 | trpD; anthranilate phosphoribosyltransferase [EC:2.4.2.18] |
| K00767 | KSG66\_12760 | nadC, QPRT; nicotinate-nucleotide pyrophosphorylase (carboxylating) [EC:2.4.2.19] |
| K00788 | KSG66\_18120 | thiE; thiamine-phosphate pyrophosphorylase [EC:2.5.1.3] |
| K00789 | KSG66\_14080 | metK; S-adenosylmethionine synthetase [EC:2.5.1.6] |
| K00790 | KSG66\_17350 | murA; UDP-N-acetylglucosamine 1-carboxyvinyltransferase [EC:2.5.1.7] |
| K00790 | KSG66\_17520 | murA; UDP-N-acetylglucosamine 1-carboxyvinyltransferase [EC:2.5.1.7] |
| K00791 | KSG66\_08660 | miaA, TRIT1; tRNA dimethylallyltransferase [EC:2.5.1.75] |
| K00793 | KSG66\_10875 | ribE, RIB5; riboflavin synthase [EC:2.5.1.9] |
| K00794 | KSG66\_10865 | ribH, RIB4; 6,7-dimethyl-8-ribityllumazine synthase [EC:2.5.1.78] |
| K00796 | KSG66\_00460 | folP; dihydropteroate synthase [EC:2.5.1.15] |
| K00797 | KSG66\_17670 | speE, SRM; spermidine synthase [EC:2.5.1.16] |
| K00798 | KSG66\_15475 | MMAB, pduO; cob(I)alamin adenosyltransferase [EC:2.5.1.17] |
| K00800 | KSG66\_10545 | aroA; 3-phosphoshikimate 1-carboxyvinyltransferase [EC:2.5.1.19] |
| K00812 | KSG66\_10425 | aspB; aspartate aminotransferase [EC:2.6.1.1] |
| K00817 | KSG66\_10555 | hisC; histidinol-phosphate aminotransferase [EC:2.6.1.9] |
| K00819 | KSG66\_18965 | rocD, OAT; ornithine--oxo-acid transaminase [EC:2.6.1.13] |
| K00820 | KSG66\_01140 | glmS, GFPT; glucosamine---fructose-6-phosphate aminotransferase (isomerizing) [EC:2.6.1.16] |
| K00821 | KSG66\_05550 | argD; acetylornithine/N-succinyldiaminopimelate aminotransferase [EC:2.6.1.11 2.6.1.17] |
| K00824 | KSG66\_04865 | dat; D-alanine transaminase [EC:2.6.1.21] |
| K00826 | KSG66\_01380 | E2.6.1.42, ilvE; branched-chain amino acid aminotransferase [EC:2.6.1.42] |
| K00826 | KSG66\_18220 | E2.6.1.42, ilvE; branched-chain amino acid aminotransferase [EC:2.6.1.42] |
| K00831 | KSG66\_05055 | serC, PSAT1; phosphoserine aminotransferase [EC:2.6.1.52] |
| K00839 | KSG66\_15095 | pucG; (S)-ureidoglycine---glyoxylate transaminase [EC:2.6.1.112] |
| K00841 | KSG66\_06980 | patA; aminotransferase [EC:2.6.1.-] |
| K00845 | KSG66\_11770 | glk; glucokinase [EC:2.7.1.2] |
| K00847 | KSG66\_03210 | E2.7.1.4, scrK; fructokinase [EC:2.7.1.4] |
| K00849 | KSG66\_05995 | galK; galactokinase [EC:2.7.1.6] |
| K00849 | KSG66\_18075 | galK; galactokinase [EC:2.7.1.6] |
| K00850 | KSG66\_13440 | pfkA, PFK; 6-phosphofructokinase 1 [EC:2.7.1.11] |
| K00851 | KSG66\_16055 | E2.7.1.12, gntK, idnK; gluconokinase [EC:2.7.1.12] |
| K00852 | KSG66\_16940 | rbsK, RBKS; ribokinase [EC:2.7.1.15] |
| K00853 | KSG66\_13230 | araB; L-ribulokinase [EC:2.7.1.16] |
| K00854 | KSG66\_08785 | xylB, XYLB; xylulokinase [EC:2.7.1.17] |
| K00857 | KSG66\_17500 | tdk, TK; thymidine kinase [EC:2.7.1.21] |
| K00858 | KSG66\_05755 | ppnK, NADK; NAD+ kinase [EC:2.7.1.23] |
| K00858 | KSG66\_13555 | ppnK, NADK; NAD+ kinase [EC:2.7.1.23] |
| K00859 | KSG66\_13355 | coaE; dephospho-CoA kinase [EC:2.7.1.24] |
| K00860 | KSG66\_07795 | cysC; adenylylsulfate kinase [EC:2.7.1.25] |
| K00864 | KSG66\_04690 | glpK, GK; glycerol kinase [EC:2.7.1.30] |
| K00865 | KSG66\_00095 | glxK, garK; glycerate 2-kinase [EC:2.7.1.165] |
| K00867 | KSG66\_11120 | coaA; type I pantothenate kinase [EC:2.7.1.33] |
| K00868 | KSG66\_17985 | pdxK, pdxY; pyridoxine kinase [EC:2.7.1.35] |
| K00872 | KSG66\_14990 | thrB1; homoserine kinase [EC:2.7.1.39] |
| K00873 | KSG66\_13435 | PK, pyk; pyruvate kinase [EC:2.7.1.40] |
| K00874 | KSG66\_09060 | kdgK; 2-dehydro-3-deoxygluconokinase [EC:2.7.1.45] |
| K00876 | KSG66\_12510 | udk, UCK; uridine kinase [EC:2.7.1.48] |
| K00878 | KSG66\_18125 | thiM; hydroxyethylthiazole kinase [EC:2.7.1.50] |
| K00882 | KSG66\_07160 | fruK; 1-phosphofructokinase [EC:2.7.1.56] |
| K00891 | KSG66\_01710 | E2.7.1.71, aroK, aroL; shikimate kinase [EC:2.7.1.71] |
| K00899 | KSG66\_06765 | mtnK; 5-methylthioribose kinase [EC:2.7.1.100] |
| K00919 | KSG66\_00295 | ispE; 4-diphosphocytidyl-2-C-methyl-D-erythritol kinase [EC:2.7.1.148] |
| K00925 | KSG66\_13520 | ackA; acetate kinase [EC:2.7.2.1] |
| K00927 | KSG66\_15985 | PGK, pgk; phosphoglycerate kinase [EC:2.7.2.3] |
| K00928 | KSG66\_02010 | lysC; aspartate kinase [EC:2.7.2.4] |
| K00928 | KSG66\_08380 | lysC; aspartate kinase [EC:2.7.2.4] |
| K00928 | KSG66\_13075 | lysC; aspartate kinase [EC:2.7.2.4] |
| K00929 | KSG66\_11355 | buk; butyrate kinase [EC:2.7.2.7] |
| K00930 | KSG66\_05545 | argB; acetylglutamate kinase [EC:2.7.2.8] |
| K00931 | KSG66\_06565 | proB; glutamate 5-kinase [EC:2.7.2.11] |
| K00931 | KSG66\_09395 | proB; glutamate 5-kinase [EC:2.7.2.11] |
| K00939 | KSG66\_00835 | adk, AK; adenylate kinase [EC:2.7.4.3] |
| K00940 | KSG66\_10610 | ndk, NME; nucleoside-diphosphate kinase [EC:2.7.4.6] |
| K00941 | KSG66\_05805 | thiD; hydroxymethylpyrimidine/phosphomethylpyrimidine kinase [EC:2.7.1.49 2.7.4.7] |
| K00942 | KSG66\_07835 | E2.7.4.8, gmk; guanylate kinase [EC:2.7.4.8] |
| K00943 | KSG66\_00205 | tmk, DTYMK; dTMP kinase [EC:2.7.4.9] |
| K00945 | KSG66\_10690 | cmk; CMP/dCMP kinase [EC:2.7.4.25] |
| K00946 | KSG66\_03100 | thiL; thiamine-monophosphate kinase [EC:2.7.4.16] |
| K00948 | KSG66\_00320 | PRPS, prsA; ribose-phosphate pyrophosphokinase [EC:2.7.6.1] |
| K00949 | KSG66\_07895 | thiN, TPK1, THI80; thiamine pyrophosphokinase [EC:2.7.6.2] |
| K00950 | KSG66\_00470 | folK; 2-amino-4-hydroxy-6-hydroxymethyldihydropteridine diphosphokinase [EC:2.7.6.3] |
| K00954 | KSG66\_07520 | E2.7.7.3A, coaD, kdtB; pantetheine-phosphate adenylyltransferase [EC:2.7.7.3] |
| K00958 | KSG66\_07790 | sat, met3; sulfate adenylyltransferase [EC:2.7.7.4] |
| K00963 | KSG66\_16805 | UGP2, galU, galF; UTP--glucose-1-phosphate uridylyltransferase [EC:2.7.7.9] |
| K00965 | KSG66\_05985 | galT, GALT; UDPglucose--hexose-1-phosphate uridylyltransferase [EC:2.7.7.12] |
| K00965 | KSG66\_18070 | galT, GALT; UDPglucose--hexose-1-phosphate uridylyltransferase [EC:2.7.7.12] |
| K00969 | KSG66\_12160 | nadD; nicotinate-nucleotide adenylyltransferase [EC:2.7.7.18] |
| K00973 | KSG66\_17900 | E2.7.7.24, rfbA, rffH; glucose-1-phosphate thymidylyltransferase [EC:2.7.7.24] |
| K00978 | KSG66\_03650 | rfbF; glucose-1-phosphate cytidylyltransferase [EC:2.7.7.33] |
| K00981 | KSG66\_08270 | E2.7.7.41, CDS1, CDS2, cdsA; phosphatidate cytidylyltransferase [EC:2.7.7.41] |
| K00991 | KSG66\_00600 | ispD; 2-C-methyl-D-erythritol 4-phosphate cytidylyltransferase [EC:2.7.7.60] |
| K00995 | KSG66\_08460 | pgsA, PGS1; CDP-diacylglycerol---glycerol-3-phosphate 3-phosphatidyltransferase [EC:2.7.8.5] |
| K01000 | KSG66\_07605 | mraY; phospho-N-acetylmuramoyl-pentapeptide-transferase [EC:2.7.8.13] |
| K01012 | KSG66\_09205 | bioB; biotin synthase [EC:2.8.1.6] |
| K01028 | KSG66\_09190 | E2.8.3.5A, scoA; 3-oxoacid CoA-transferase subunit A [EC:2.8.3.5] |
| K01029 | KSG66\_09185 | E2.8.3.5B, scoB; 3-oxoacid CoA-transferase subunit B [EC:2.8.3.5] |
| K01034 | KSG66\_09850 | atoD; acetate CoA/acetoacetate CoA-transferase alpha subunit [EC:2.8.3.8 2.8.3.9] |
| K01035 | KSG66\_09845 | atoA; acetate CoA/acetoacetate CoA-transferase beta subunit [EC:2.8.3.8 2.8.3.9] |
| K01046 | KSG66\_01510 | lip, TGL2; triacylglycerol lipase [EC:3.1.1.3] |
| K01069 | KSG66\_08535 | gloB, gloC, HAGH; hydroxyacylglutathione hydrolase [EC:3.1.2.6] |
| K01069 | KSG66\_11745 | gloB, gloC, HAGH; hydroxyacylglutathione hydrolase [EC:3.1.2.6] |
| K01071 | KSG66\_16565 | MCH; medium-chain acyl-[acyl-carrier-protein] hydrolase [EC:3.1.2.21] |
| K01077 | KSG66\_04750 | E3.1.3.1, phoA, phoB; alkaline phosphatase [EC:3.1.3.1] |
| K01081 | KSG66\_04640 | E3.1.3.5; 5'-nucleotidase [EC:3.1.3.5] |
| K01081 | KSG66\_15045 | E3.1.3.5; 5'-nucleotidase [EC:3.1.3.5] |
| K01083 | KSG66\_09895 | E3.1.3.8; 3-phytase [EC:3.1.3.8] |
| K01092 | KSG66\_01010 | E3.1.3.25, IMPA, suhB; myo-inositol-1(or 4)-monophosphatase [EC:3.1.3.25] |
| K01092 | KSG66\_07360 | E3.1.3.25, IMPA, suhB; myo-inositol-1(or 4)-monophosphatase [EC:3.1.3.25] |
| K01113 | KSG66\_01470 | phoD; alkaline phosphatase D [EC:3.1.3.1] |
| K01139 | KSG66\_12660 | spoT; GTP diphosphokinase / guanosine-3',5'-bis(diphosphate) 3'-diphosphatase [EC:2.7.6.5 3.1.7.2] |
| K01179 | KSG66\_09130 | E3.2.1.4; endoglucanase [EC:3.2.1.4] |
| K01182 | KSG66\_01555 | IMA, malL; oligo-1,6-glucosidase [EC:3.2.1.10] |
| K01182 | KSG66\_16290 | IMA, malL; oligo-1,6-glucosidase [EC:3.2.1.10] |
| K01187 | KSG66\_14475 | malZ; alpha-glucosidase [EC:3.2.1.20] |
| K01193 | KSG66\_16265 | INV, sacA; beta-fructofuranosidase [EC:3.2.1.26] |
| K01193 | KSG66\_17995 | INV, sacA; beta-fructofuranosidase [EC:3.2.1.26] |
| K01198 | KSG66\_08770 | xynB; xylan 1,4-beta-xylosidase [EC:3.2.1.37] |
| K01201 | KSG66\_09750 | GBA, srfJ; glucosylceramidase [EC:3.2.1.45] |
| K01207 | KSG66\_01060 | nagZ; beta-N-acetylhexosaminidase [EC:3.2.1.52] |
| K01208 | KSG66\_16320 | cd, ma, nplT; cyclomaltodextrinase / maltogenic alpha-amylase / neopullulanase [EC:3.2.1.54 3.2.1.133 3.2.1.135] |
| K01209 | KSG66\_13090 | abfA; alpha-N-arabinofuranosidase [EC:3.2.1.55] |
| K01209 | KSG66\_13195 | abfA; alpha-N-arabinofuranosidase [EC:3.2.1.55] |
| K01218 | KSG66\_18310 | gmuG; mannan endo-1,4-beta-mannosidase [EC:3.2.1.78] |
| K01220 | KSG66\_06010 | E3.2.1.85, lacG; 6-phospho-beta-galactosidase [EC:3.2.1.85] |
| K01226 | KSG66\_03875 | treC; trehalose-6-phosphate hydrolase [EC:3.2.1.93] |
| K01232 | KSG66\_04080 | glvA; maltose-6'-phosphate glucosidase [EC:3.2.1.122] |
| K01233 | KSG66\_15195 | csn; chitosanase [EC:3.2.1.132] |
| K01243 | KSG66\_12480 | mtnN, mtn, pfs; adenosylhomocysteine nucleosidase [EC:3.2.2.9] |
| K01255 | KSG66\_14865 | CARP, pepA; leucyl aminopeptidase [EC:3.4.11.1] |
| K01424 | KSG66\_01505 | E3.5.1.1, ansA, ansB; L-asparaginase [EC:3.5.1.1] |
| K01424 | KSG66\_11025 | E3.5.1.1, ansA, ansB; L-asparaginase [EC:3.5.1.1] |
| K01425 | KSG66\_01400 | glsA, GLS; glutaminase [EC:3.5.1.2] |
| K01425 | KSG66\_07425 | glsA, GLS; glutaminase [EC:3.5.1.2] |
| K01428 | KSG66\_17290 | ureC; urease subunit alpha [EC:3.5.1.5] |
| K01429 | KSG66\_17295 | ureB; urease subunit beta [EC:3.5.1.5] |
| K01430 | KSG66\_17300 | ureA; urease subunit gamma [EC:3.5.1.5] |
| K01438 | KSG66\_09840 | argE; acetylornithine deacetylase [EC:3.5.1.16] |
| K01439 | KSG66\_13865 | dapE; succinyl-diaminopimelate desuccinylase [EC:3.5.1.18] |
| K01443 | KSG66\_16485 | nagA, AMDHD2; N-acetylglucosamine-6-phosphate deacetylase [EC:3.5.1.25] |
| K01465 | KSG66\_07745 | URA4, pyrC; dihydroorotase [EC:3.5.2.3] |
| K01466 | KSG66\_15065 | allB; allantoinase [EC:3.5.2.5] |
| K01468 | KSG66\_18585 | hutI, AMDHD1; imidazolonepropionase [EC:3.5.2.7] |
| K01476 | KSG66\_18955 | E3.5.3.1, rocF, arg; arginase [EC:3.5.3.1] |
| K01479 | KSG66\_18590 | hutG; formiminoglutamase [EC:3.5.3.8] |
| K01480 | KSG66\_17665 | speB; agmatinase [EC:3.5.3.11] |
| K01486 | KSG66\_03410 | ade; adenine deaminase [EC:3.5.4.2] |
| K01486 | KSG66\_07225 | ade; adenine deaminase [EC:3.5.4.2] |
| K01489 | KSG66\_11980 | cdd, CDA; cytidine deaminase [EC:3.5.4.5] |
| K01491 | KSG66\_11505 | folD; methylenetetrahydrofolate dehydrogenase (NADP+) / methenyltetrahydrofolate cyclohydrolase [EC:1.5.1.5 3.5.4.9] |
| K01493 | KSG66\_12130 | comEB; dCMP deaminase [EC:3.5.4.12] |
| K01495 | KSG66\_10635 | GCH1, folE; GTP cyclohydrolase IA [EC:3.5.4.16] |
| K01512 | KSG66\_03805 | acyP; acylphosphatase [EC:3.6.1.7] |
| K01515 | KSG66\_11040 | nudF; ADP-ribose pyrophosphatase [EC:3.6.1.13] |
| K01520 | KSG66\_08885 | dut, DUT; dUTP pyrophosphatase [EC:3.6.1.23] |
| K01560 | KSG66\_01210 | E3.8.1.2; 2-haloacid dehalogenase [EC:3.8.1.2] |
| K01569 | KSG66\_15530 | oxdD; oxalate decarboxylase [EC:4.1.1.2] |
| K01574 | KSG66\_15350 | adc; acetoacetate decarboxylase [EC:4.1.1.4] |
| K01579 | KSG66\_10445 | panD; aspartate 1-decarboxylase [EC:4.1.1.11] |
| K01585 | KSG66\_07340 | speA; arginine decarboxylase [EC:4.1.1.19] |
| K01586 | KSG66\_10910 | lysA; diaminopimelate decarboxylase [EC:4.1.1.20] |
| K01588 | KSG66\_03345 | purE; 5-(carboxyamino)imidazole ribonucleotide mutase [EC:5.4.99.18] |
| K01589 | KSG66\_03350 | purK; 5-(carboxyamino)imidazole ribonucleotide synthase [EC:6.3.4.18] |
| K01591 | KSG66\_07770 | pyrF; orotidine-5'-phosphate decarboxylase [EC:4.1.1.23] |
| K01599 | KSG66\_05100 | hemE, UROD; uroporphyrinogen decarboxylase [EC:4.1.1.37] |
| K01607 | KSG66\_19235 | pcaC; 4-carboxymuconolactone decarboxylase [EC:4.1.1.44] |
| K01609 | KSG66\_10575 | trpC; indole-3-glycerol phosphate synthase [EC:4.1.1.48] |
| K01610 | KSG66\_14085 | E4.1.1.49, pckA; phosphoenolpyruvate carboxykinase (ATP) [EC:4.1.1.49] |
| K01611 | KSG66\_13330 | speD, AMD1; S-adenosylmethionine decarboxylase [EC:4.1.1.50] |
| K01613 | KSG66\_01360 | psd, PISD; phosphatidylserine decarboxylase [EC:4.1.1.65] |
| K01619 | KSG66\_18610 | deoC, DERA; deoxyribose-phosphate aldolase [EC:4.1.2.4] |
| K01624 | KSG66\_17530 | FBA, fbaA; fructose-bisphosphate aldolase, class II [EC:4.1.2.13] |
| K01625 | KSG66\_09070 | eda; 2-dehydro-3-deoxyphosphogluconate aldolase / (4S)-4-hydroxy-2-oxoglutarate aldolase [EC:4.1.2.14 4.1.3.42] |
| K01633 | KSG66\_00465 | folB; 7,8-dihydroneopterin aldolase/epimerase/oxygenase [EC:4.1.2.25 5.1.99.8 1.13.11.81] |
| K01640 | KSG66\_09255 | E4.1.3.4, HMGCL, hmgL; hydroxymethylglutaryl-CoA lyase [EC:4.1.3.4] |
| K01647 | KSG66\_04765 | CS, gltA; citrate synthase [EC:2.3.3.1] |
| K01647 | KSG66\_11390 | CS, gltA; citrate synthase [EC:2.3.3.1] |
| K01649 | KSG66\_12975 | leuA, IMS; 2-isopropylmalate synthase [EC:2.3.3.13] |
| K01652 | KSG66\_12990 | E2.2.1.6L, ilvB, ilvG, ilvI; acetolactate synthase I/II/III large subunit [EC:2.2.1.6] |
| K01652 | KSG66\_16980 | E2.2.1.6L, ilvB, ilvG, ilvI; acetolactate synthase I/II/III large subunit [EC:2.2.1.6] |
| K01653 | KSG66\_12985 | E2.2.1.6S, ilvH, ilvN; acetolactate synthase I/III small subunit [EC:2.2.1.6] |
| K01657 | KSG66\_10585 | trpE; anthranilate synthase component I [EC:4.1.3.27] |
| K01661 | KSG66\_14190 | menB; naphthoate synthase [EC:4.1.3.36] |
| K01662 | KSG66\_11485 | dxs; 1-deoxy-D-xylulose-5-phosphate synthase [EC:2.2.1.7] |
| K01673 | KSG66\_14150 | cynT, can; carbonic anhydrase [EC:4.2.1.1] |
| K01673 | KSG66\_16345 | cynT, can; carbonic anhydrase [EC:4.2.1.1] |
| K01679 | KSG66\_15415 | E4.2.1.2B, fumC, FH; fumarate hydratase, class II [EC:4.2.1.2] |
| K01681 | KSG66\_08990 | ACO, acnA; aconitate hydratase [EC:4.2.1.3] |
| K01685 | KSG66\_06140 | uxaA; altronate hydrolase [EC:4.2.1.7] |
| K01686 | KSG66\_09075 | uxuA; mannonate dehydratase [EC:4.2.1.8] |
| K01687 | KSG66\_10150 | ilvD; dihydroxy-acid dehydratase [EC:4.2.1.9] |
| K01689 | KSG66\_15970 | ENO, eno; enolase [EC:4.2.1.11] |
| K01693 | KSG66\_16440 | hisB; imidazoleglycerol-phosphate dehydratase [EC:4.2.1.19] |
| K01695 | KSG66\_10560 | trpA; tryptophan synthase alpha chain [EC:4.2.1.20] |
| K01696 | KSG66\_10565 | trpB; tryptophan synthase beta chain [EC:4.2.1.20] |
| K01698 | KSG66\_12900 | hemB, ALAD; porphobilinogen synthase [EC:4.2.1.24] |
| K01703 | KSG66\_12965 | leuC, IPMI-L; 3-isopropylmalate/(R)-2-methylmalate dehydratase large subunit [EC:4.2.1.33 4.2.1.35] |
| K01704 | KSG66\_12960 | leuD, IPMI-S; 3-isopropylmalate/(R)-2-methylmalate dehydratase small subunit [EC:4.2.1.33 4.2.1.35] |
| K01709 | KSG66\_03655 | rfbG; CDP-glucose 4,6-dehydratase [EC:4.2.1.45] |
| K01710 | KSG66\_17895 | E4.2.1.46, rfbB, rffG; dTDP-glucose 4,6-dehydratase [EC:4.2.1.46] |
| K01712 | KSG66\_18580 | hutU, UROC1; urocanate hydratase [EC:4.2.1.49] |
| K01714 | KSG66\_02785 | dapA; 4-hydroxy-tetrahydrodipicolinate synthase [EC:4.3.3.7] |
| K01714 | KSG66\_08385 | dapA; 4-hydroxy-tetrahydrodipicolinate synthase [EC:4.3.3.7] |
| K01719 | KSG66\_06085 | hemD, UROS; uroporphyrinogen-III synthase [EC:4.2.1.75] |
| K01719 | KSG66\_12905 | hemD, UROS; uroporphyrinogen-III synthase [EC:4.2.1.75] |
| K01720 | KSG66\_11385 | prpD; 2-methylcitrate dehydratase [EC:4.2.1.79] |
| K01728 | KSG66\_03750 | pel; pectate lyase [EC:4.2.2.2] |
| K01733 | KSG66\_14995 | thrC; threonine synthase [EC:4.2.3.1] |
| K01734 | KSG66\_10480 | mgsA; methylglyoxal synthase [EC:4.2.3.3] |
| K01735 | KSG66\_10595 | aroB; 3-dehydroquinate synthase [EC:4.2.3.4] |
| K01736 | KSG66\_10600 | aroC; chorismate synthase [EC:4.2.3.5] |
| K01737 | KSG66\_06845 | queD, ptpS, PTS; 6-pyruvoyltetrahydropterin/6-carboxytetrahydropterin synthase [EC:4.2.3.12 4.1.2.50] |
| K01738 | KSG66\_00440 | cysK; cysteine synthase [EC:2.5.1.47] |
| K01738 | KSG66\_13860 | cysK; cysteine synthase [EC:2.5.1.47] |
| K01739 | KSG66\_05910 | metB; cystathionine gamma-synthase [EC:2.5.1.48] |
| K01743 | KSG66\_08820 | E4.2.1.1; carbonic anhydrase [EC:4.2.1.1] |
| K01744 | KSG66\_11020 | aspA; aspartate ammonia-lyase [EC:4.3.1.1] |
| K01745 | KSG66\_18575 | hutH, HAL; histidine ammonia-lyase [EC:4.3.1.3] |
| K01749 | KSG66\_12910 | hemC, HMBS; hydroxymethylbilane synthase [EC:2.5.1.61] |
| K01752 | KSG66\_07920 | E4.3.1.17, sdaA, sdaB, tdcG; L-serine dehydratase [EC:4.3.1.17] |
| K01752 | KSG66\_07925 | E4.3.1.17, sdaA, sdaB, tdcG; L-serine dehydratase [EC:4.3.1.17] |
| K01753 | KSG66\_11125 | dsdA; D-serine dehydratase [EC:4.3.1.18] |
| K01754 | KSG66\_10105 | E4.3.1.19, ilvA, tdcB; threonine dehydratase [EC:4.3.1.19] |
| K01755 | KSG66\_13505 | argH, ASL; argininosuccinate lyase [EC:4.3.2.1] |
| K01756 | KSG66\_03355 | purB, ADSL; adenylosuccinate lyase [EC:4.3.2.2] |
| K01759 | KSG66\_03600 | GLO1, gloA; lactoylglutathione lyase [EC:4.4.1.5] |
| K01759 | KSG66\_05955 | GLO1, gloA; lactoylglutathione lyase [EC:4.4.1.5] |
| K01759 | KSG66\_18165 | GLO1, gloA; lactoylglutathione lyase [EC:4.4.1.5] |
| K01759 | KSG66\_19300 | GLO1, gloA; lactoylglutathione lyase [EC:4.4.1.5] |
| K01760 | KSG66\_05915 | metC; cysteine-S-conjugate beta-lyase [EC:4.4.1.13] |
| K01770 | KSG66\_00605 | ispF; 2-C-methyl-D-erythritol 2,4-cyclodiphosphate synthase [EC:4.6.1.12] |
| K01772 | KSG66\_05105 | hemH, FECH; protoporphyrin/coproporphyrin ferrochelatase [EC:4.99.1.1 4.99.1.9] |
| K01775 | KSG66\_02475 | alr; alanine racemase [EC:5.1.1.1] |
| K01775 | KSG66\_11455 | alr; alanine racemase [EC:5.1.1.1] |
| K01776 | KSG66\_13035 | murI; glutamate racemase [EC:5.1.1.3] |
| K01778 | KSG66\_14955 | dapF; diaminopimelate epimerase [EC:5.1.1.7] |
| K01779 | KSG66\_02710 | racD; aspartate racemase [EC:5.1.1.13] |
| K01779 | KSG66\_12225 | racD; aspartate racemase [EC:5.1.1.13] |
| K01783 | KSG66\_07890 | rpe, RPE; ribulose-phosphate 3-epimerase [EC:5.1.3.1] |
| K01784 | KSG66\_05990 | galE, GALE; UDP-glucose 4-epimerase [EC:5.1.3.2] |
| K01784 | KSG66\_18420 | galE, GALE; UDP-glucose 4-epimerase [EC:5.1.3.2] |
| K01785 | KSG66\_09320 | galM, GALM; aldose 1-epimerase [EC:5.1.3.3] |
| K01790 | KSG66\_17885 | rfbC, rmlC; dTDP-4-dehydrorhamnose 3,5-epimerase [EC:5.1.3.13] |
| K01791 | KSG66\_16800 | wecB; UDP-N-acetylglucosamine 2-epimerase (non-hydrolysing) [EC:5.1.3.14] |
| K01803 | KSG66\_15980 | TPI, tpiA; triosephosphate isomerase (TIM) [EC:5.3.1.1] |
| K01804 | KSG66\_13235 | araA; L-arabinose isomerase [EC:5.3.1.4] |
| K01805 | KSG66\_08780 | xylA; xylose isomerase [EC:5.3.1.5] |
| K01808 | KSG66\_17430 | rpiB; ribose 5-phosphate isomerase B [EC:5.3.1.6] |
| K01809 | KSG66\_12385 | manA, MPI; mannose-6-phosphate isomerase [EC:5.3.1.8] |
| K01809 | KSG66\_16850 | manA, MPI; mannose-6-phosphate isomerase [EC:5.3.1.8] |
| K01809 | KSG66\_18305 | manA, MPI; mannose-6-phosphate isomerase [EC:5.3.1.8] |
| K01810 | KSG66\_14515 | GPI, pgi; glucose-6-phosphate isomerase [EC:5.3.1.9] |
| K01812 | KSG66\_06120 | uxaC; glucuronate isomerase [EC:5.3.1.12] |
| K01814 | KSG66\_16430 | hisA; phosphoribosylformimino-5-aminoimidazole carboxamide ribotide isomerase [EC:5.3.1.16] |
| K01817 | KSG66\_10570 | trpF; phosphoribosylanthranilate isomerase [EC:5.3.1.24] |
| K01821 | KSG66\_17695 | praC, xylH; 4-oxalocrotonate tautomerase [EC:5.3.2.6] |
| K01823 | KSG66\_10680 | idi, IDI; isopentenyl-diphosphate Delta-isomerase [EC:5.3.3.2] |
| K01835 | KSG66\_04700 | pgm; phosphoglucomutase [EC:5.4.2.2] |
| K01839 | KSG66\_10975 | deoB; phosphopentomutase [EC:5.4.2.7] |
| K01843 | KSG66\_09830 | kamA; lysine 2,3-aminomutase [EC:5.4.3.2] |
| K01845 | KSG66\_04300 | hemL; glutamate-1-semialdehyde 2,1-aminomutase [EC:5.4.3.8] |
| K01845 | KSG66\_12895 | hemL; glutamate-1-semialdehyde 2,1-aminomutase [EC:5.4.3.8] |
| K01874 | KSG66\_00255 | MARS, metG; methionyl-tRNA synthetase [EC:6.1.1.10] |
| K01895 | KSG66\_13565 | ACSS, acs; acetyl-CoA synthetase [EC:6.2.1.1] |
| K01895 | KSG66\_13725 | ACSS, acs; acetyl-CoA synthetase [EC:6.2.1.1] |
| K01897 | KSG66\_02195 | ACSL, fadD; long-chain acyl-CoA synthetase [EC:6.2.1.3] |
| K01897 | KSG66\_05165 | ACSL, fadD; long-chain acyl-CoA synthetase [EC:6.2.1.3] |
| K01897 | KSG66\_05200 | ACSL, fadD; long-chain acyl-CoA synthetase [EC:6.2.1.3] |
| K01897 | KSG66\_13115 | ACSL, fadD; long-chain acyl-CoA synthetase [EC:6.2.1.3] |
| K01902 | KSG66\_08045 | sucD; succinyl-CoA synthetase alpha subunit [EC:6.2.1.5] |
| K01903 | KSG66\_08040 | sucC; succinyl-CoA synthetase beta subunit [EC:6.2.1.5] |
| K01906 | KSG66\_09225 | bioW; 6-carboxyhexanoate--CoA ligase [EC:6.2.1.14] |
| K01911 | KSG66\_14185 | menE; O-succinylbenzoic acid---CoA ligase [EC:6.2.1.26] |
| K01915 | KSG66\_08725 | glnA, GLUL; glutamine synthetase [EC:6.3.1.2] |
| K01916 | KSG66\_01685 | nadE; NAD+ synthase [EC:6.3.1.5] |
| K01918 | KSG66\_10450 | panC; pantoate--beta-alanine ligase [EC:6.3.2.1] |
| K01921 | KSG66\_02435 | ddl; D-alanine-D-alanine ligase [EC:6.3.2.4] |
| K01923 | KSG66\_03360 | purC; phosphoribosylaminoimidazole-succinocarboxamide synthase [EC:6.3.2.6] |
| K01924 | KSG66\_13780 | murC; UDP-N-acetylmuramate--alanine ligase [EC:6.3.2.8] |
| K01925 | KSG66\_07610 | murD; UDP-N-acetylmuramoylalanine--D-glutamate ligase [EC:6.3.2.9] |
| K01928 | KSG66\_07600 | murE; UDP-N-acetylmuramoyl-L-alanyl-D-glutamate--2,6-diaminopimelate ligase [EC:6.3.2.13] |
| K01929 | KSG66\_02440 | murF; UDP-N-acetylmuramoyl-tripeptide--D-alanyl-D-alanine ligase [EC:6.3.2.10] |
| K01933 | KSG66\_03385 | purM; phosphoribosylformylglycinamidine cyclo-ligase [EC:6.3.3.1] |
| K01934 | KSG66\_11790 | MTHFS; 5-formyltetrahydrofolate cyclo-ligase [EC:6.3.3.2] |
| K01935 | KSG66\_09210 | bioD; dethiobiotin synthetase [EC:6.3.3.3] |
| K01937 | KSG66\_17545 | pyrG, CTPS; CTP synthase [EC:6.3.4.2] |
| K01939 | KSG66\_19070 | purA, ADSS; adenylosuccinate synthase [EC:6.3.4.4] |
| K01940 | KSG66\_13510 | argG, ASS1; argininosuccinate synthase [EC:6.3.4.5] |
| K01945 | KSG66\_03400 | purD; phosphoribosylamine---glycine ligase [EC:6.3.4.13] |
| K01951 | KSG66\_03300 | guaA, GMPS; GMP synthase (glutamine-hydrolysing) [EC:6.3.5.2] |
| K01953 | KSG66\_05395 | asnB, ASNS; asparagine synthase (glutamine-hydrolysing) [EC:6.3.5.4] |
| K01953 | KSG66\_14075 | asnB, ASNS; asparagine synthase (glutamine-hydrolysing) [EC:6.3.5.4] |
| K01955 | KSG66\_05560 | carB, CPA2; carbamoyl-phosphate synthase large subunit [EC:6.3.5.5] |
| K01955 | KSG66\_07755 | carB, CPA2; carbamoyl-phosphate synthase large subunit [EC:6.3.5.5] |
| K01956 | KSG66\_05555 | carA, CPA1; carbamoyl-phosphate synthase small subunit [EC:6.3.5.5] |
| K01956 | KSG66\_07750 | carA, CPA1; carbamoyl-phosphate synthase small subunit [EC:6.3.5.5] |
| K01958 | KSG66\_07440 | PC, pyc; pyruvate carboxylase [EC:6.4.1.1] |
| K01961 | KSG66\_09265 | accC; acetyl-CoA carboxylase, biotin carboxylase subunit [EC:6.4.1.2 6.3.4.14] |
| K01961 | KSG66\_11520 | accC; acetyl-CoA carboxylase, biotin carboxylase subunit [EC:6.4.1.2 6.3.4.14] |
| K01962 | KSG66\_13445 | accA; acetyl-CoA carboxylase carboxyl transferase subunit alpha [EC:6.4.1.2 2.1.3.15] |
| K01963 | KSG66\_13450 | accD; acetyl-CoA carboxylase carboxyl transferase subunit beta [EC:6.4.1.2 2.1.3.15] |
| K01966 | KSG66\_11285 | PCCB, pccB; propionyl-CoA carboxylase beta chain [EC:6.4.1.3 2.1.3.15] |
| K02078 | KSG66\_07955 | acpP; acyl carrier protein |
| K02078 | KSG66\_11205 | acpP; acyl carrier protein |
| K02083 | KSG66\_15100 | allC; allantoate deiminase [EC:3.5.3.9] |
| K02108 | KSG66\_17405 | ATPF0A, atpB; F-type H+-transporting ATPase subunit a |
| K02109 | KSG66\_17395 | ATPF0B, atpF; F-type H+-transporting ATPase subunit b |
| K02110 | KSG66\_17400 | ATPF0C, atpE; F-type H+-transporting ATPase subunit c |
| K02111 | KSG66\_17385 | ATPF1A, atpA; F-type H+/Na+-transporting ATPase subunit alpha [EC:7.1.2.2 7.2.2.1] |
| K02112 | KSG66\_17375 | ATPF1B, atpD; F-type H+/Na+-transporting ATPase subunit beta [EC:7.1.2.2 7.2.2.1] |
| K02113 | KSG66\_17390 | ATPF1D, atpH; F-type H+-transporting ATPase subunit delta |
| K02114 | KSG66\_17370 | ATPF1E, atpC; F-type H+-transporting ATPase subunit epsilon |
| K02115 | KSG66\_17380 | ATPF1G, atpG; F-type H+-transporting ATPase subunit gamma |
| K02160 | KSG66\_09260 | accB, bccP; acetyl-CoA carboxylase biotin carboxyl carrier protein |
| K02160 | KSG66\_11525 | accB, bccP; acetyl-CoA carboxylase biotin carboxyl carrier protein |
| K02257 | KSG66\_04120 | COX10, ctaB, cyoE; heme o synthase [EC:2.5.1.141] |
| K02257 | KSG66\_07450 | COX10, ctaB, cyoE; heme o synthase [EC:2.5.1.141] |
| K02259 | KSG66\_07445 | COX15, ctaA; cytochrome c oxidase assembly protein subunit 15 |
| K02274 | KSG66\_07460 | coxA, ctaD; cytochrome c oxidase subunit I [EC:1.9.3.1] |
| K02275 | KSG66\_07455 | coxB, ctaC; cytochrome c oxidase subunit II [EC:1.9.3.1] |
| K02276 | KSG66\_07465 | coxC, ctaE; cytochrome c oxidase subunit III [EC:1.9.3.1] |
| K02277 | KSG66\_07470 | coxD, ctaF; cytochrome c oxidase subunit IV [EC:1.9.3.1] |
| K02291 | KSG66\_05400 | crtB; 15-cis-phytoene synthase [EC:2.5.1.32] |
| K02304 | KSG66\_07810 | MET8; precorrin-2 dehydrogenase / sirohydrochlorin ferrochelatase [EC:1.3.1.76 4.99.1.4] |
| K02361 | KSG66\_14835 | entC; isochorismate synthase [EC:5.4.4.2] |
| K02372 | KSG66\_02120 | fabZ; 3-hydroxyacyl-[acyl-carrier-protein] dehydratase [EC:4.2.1.59] |
| K02372 | KSG66\_17165 | fabZ; 3-hydroxyacyl-[acyl-carrier-protein] dehydratase [EC:4.2.1.59] |
| K02428 | KSG66\_13020 | rdgB; XTP/dITP diphosphohydrolase [EC:3.6.1.66] |
| K02433 | KSG66\_03470 | gatA, QRSL1; aspartyl-tRNA(Asn)/glutamyl-tRNA(Gln) amidotransferase subunit A [EC:6.3.5.6 6.3.5.7] |
| K02434 | KSG66\_03475 | gatB, PET112; aspartyl-tRNA(Asn)/glutamyl-tRNA(Gln) amidotransferase subunit B [EC:6.3.5.6 6.3.5.7] |
| K02435 | KSG66\_03465 | gatC, GATC; aspartyl-tRNA(Asn)/glutamyl-tRNA(Gln) amidotransferase subunit C [EC:6.3.5.6 6.3.5.7] |
| K02437 | KSG66\_15270 | gcvH, GCSH; glycine cleavage system H protein |
| K02446 | KSG66\_17515 | glpX; fructose-1,6-bisphosphatase II [EC:3.1.3.11] |
| K02492 | KSG66\_12920 | hemA; glutamyl-tRNA reductase [EC:1.2.1.70] |
| K02495 | KSG66\_04940 | hemN, hemZ; oxygen-independent coproporphyrinogen III oxidase [EC:1.3.98.3] |
| K02495 | KSG66\_12080 | hemN, hemZ; oxygen-independent coproporphyrinogen III oxidase [EC:1.3.98.3] |
| K02500 | KSG66\_16425 | hisF; imidazole glycerol-phosphate synthase subunit HisF [EC:4.3.2.10] |
| K02501 | KSG66\_16435 | hisH; imidazole glycerol-phosphate synthase subunit HisH [EC:4.3.2.10] |
| K02502 | KSG66\_16455 | hisZ; ATP phosphoribosyltransferase regulatory subunit |
| K02548 | KSG66\_18190 | menA; 1,4-dihydroxy-2-naphthoate octaprenyltransferase [EC:2.5.1.74 2.5.1.-] |
| K02549 | KSG66\_14180 | menC; O-succinylbenzoate synthase [EC:4.2.1.113] |
| K02551 | KSG66\_14200 | menD; 2-succinyl-5-enolpyruvyl-6-hydroxy-3-cyclohexene-1-carboxylate synthase [EC:2.2.1.9] |
| K02563 | KSG66\_07620 | murG; UDP-N-acetylglucosamine--N-acetylmuramyl-(pentapeptide) pyrophosphoryl-undecaprenol N-acetylglucosamine transferase [EC:2.4.1.227] |
| K02564 | KSG66\_16490 | nagB, GNPDA; glucosamine-6-phosphate deaminase [EC:3.5.99.6] |
| K02770 | KSG66\_07165 | PTS-Fru-EIIC, fruA; PTS system, fructose-specific IIC component |
| K02770 | KSG66\_12390 | PTS-Fru-EIIC, fruA; PTS system, fructose-specific IIC component |
| K02777 | KSG66\_10355 | PTS-Glc-EIIA, crr; PTS system, sugar-specific IIA component [EC:2.7.1.-] |
| K02786 | KSG66\_06005 | PTS-Lac-EIIA, lacF; PTS system, lactose-specific IIA component [EC:2.7.1.207] |
| K02788 | KSG66\_06000 | PTS-Lac-EIIC, lacE; PTS system, lactose-specific IIC component |
| K02798 | KSG66\_02110 | PTS-Mtl-EIIA, mtlA, cmtB; PTS system, mannitol-specific IIA component [EC:2.7.1.197] |
| K02800 | KSG66\_02105 | PTS-Mtl-EIIC, mtlA, cmtA; PTS system, mannitol-specific IIC component |
| K02823 | KSG66\_07760 | pyrDII; dihydroorotate dehydrogenase electron transfer subunit |
| K02825 | KSG66\_07730 | pyrR; pyrimidine operon attenuation protein / uracil phosphoribosyltransferase [EC:2.4.2.9] |
| K02826 | KSG66\_18060 | qoxA; cytochrome aa3-600 menaquinol oxidase subunit II [EC:7.1.1.5] |
| K02827 | KSG66\_18055 | qoxB; cytochrome aa3-600 menaquinol oxidase subunit I [EC:7.1.1.5] |
| K02828 | KSG66\_18050 | qoxC; cytochrome aa3-600 menaquinol oxidase subunit III [EC:7.1.1.5] |
| K02829 | KSG66\_18045 | qoxD; cytochrome aa3-600 menaquinol oxidase subunit IV [EC:7.1.1.5] |
| K02851 | KSG66\_16735 | wecA, tagO, rfe; UDP-GlcNAc:undecaprenyl-phosphate/decaprenyl-phosphate GlcNAc-1-phosphate transferase [EC:2.7.8.33 2.7.8.35] |
| K03077 | KSG66\_13225 | araD, ulaF, sgaE, sgbE; L-ribulose-5-phosphate 4-epimerase [EC:5.1.3.4] |
| K03147 | KSG66\_04430 | thiC; phosphomethylpyrimidine synthase [EC:4.1.99.17] |
| K03148 | KSG66\_05800 | thiF; sulfur carrier protein ThiS adenylyltransferase [EC:2.7.7.73] |
| K03149 | KSG66\_05795 | thiG; thiazole synthase [EC:2.8.1.10] |
| K03151 | KSG66\_13575 | thiI; tRNA uracil 4-sulfurtransferase [EC:2.8.1.4] |
| K03153 | KSG66\_05785 | thiO; glycine oxidase [EC:1.4.3.19] |
| K03183 | KSG66\_10620 | ubiE; demethylmenaquinone methyltransferase / 2-methoxy-6-polyprenyl-1,4-benzoquinol methylase [EC:2.1.1.163 2.1.1.201] |
| K03186 | KSG66\_01890 | ubiX, bsdB, PAD1; flavin prenyltransferase [EC:2.5.1.129] |
| K03335 | KSG66\_18730 | iolE; inosose dehydratase [EC:4.2.1.44] |
| K03336 | KSG66\_18735 | iolD; 3D-(3,5/4)-trihydroxycyclohexane-1,2-dione acylhydrolase (decyclizing) [EC:3.7.1.22] |
| K03337 | KSG66\_18745 | iolB; 5-deoxy-glucuronate isomerase [EC:5.3.1.30] |
| K03338 | KSG66\_18740 | iolC; 5-dehydro-2-deoxygluconokinase [EC:2.7.1.92] |
| K03339 | KSG66\_18705 | iolJ; 6-phospho-5-dehydro-2-deoxy-D-gluconate aldolase [EC:4.1.2.29] |
| K03367 | KSG66\_18200 | dltA; D-alanine--poly(phosphoribitol) ligase subunit 1 [EC:6.1.1.13] |
| K03417 | KSG66\_11380 | prpB; methylisocitrate lyase [EC:4.1.3.30] |
| K03429 | KSG66\_10175 | ugtP; processive 1,2-diacylglycerol beta-glucosyltransferase [EC:2.4.1.315] |
| K03431 | KSG66\_01135 | glmM; phosphoglucosamine mutase [EC:5.4.2.10] |
| K03517 | KSG66\_12755 | nadA; quinolinate synthase [EC:2.5.1.72] |
| K03524 | KSG66\_10460 | birA; BirA family transcriptional regulator, biotin operon repressor / biotin---[acetyl-CoA-carboxylase] ligase [EC:6.3.4.15] |
| K03525 | KSG66\_00425 | coaX; type III pantothenate kinase [EC:2.7.1.33] |
| K03526 | KSG66\_11865 | gcpE, ispG; (E)-4-hydroxy-3-methylbut-2-enyl-diphosphate synthase [EC:1.17.7.1 1.17.7.3] |
| K03527 | KSG66\_11910 | ispH, lytB; 4-hydroxy-3-methylbut-2-en-1-yl diphosphate reductase [EC:1.17.7.4] |
| K03621 | KSG66\_07940 | plsX; phosphate acyltransferase [EC:2.3.1.274] |
| K03635 | KSG66\_07125 | MOCS2B, moaE; molybdopterin synthase catalytic subunit [EC:2.8.1.12] |
| K03637 | KSG66\_03130 | moaC, CNX3; cyclic pyranopterin monophosphate synthase [EC:4.6.1.17] |
| K03638 | KSG66\_13515 | moaB; molybdopterin adenylyltransferase [EC:2.7.7.75] |
| K03639 | KSG66\_17320 | moaA, CNX2; GTP 3',8-cyclase [EC:4.1.99.22] |
| K03644 | KSG66\_15030 | lipA; lipoyl synthase [EC:2.8.1.8] |
| K03707 | KSG66\_05775 | tenA; thiaminase (transcriptional activator TenA) [EC:3.5.99.2] |
| K03750 | KSG66\_07115 | moeA; molybdopterin molybdotransferase [EC:2.10.1.1] |
| K03752 | KSG66\_07105 | mobA; molybdenum cofactor guanylyltransferase [EC:2.7.7.77] |
| K03781 | KSG66\_04470 | katE, CAT, catB, srpA; catalase [EC:1.11.1.6] |
| K03781 | KSG66\_18315 | katE, CAT, catB, srpA; catalase [EC:1.11.1.6] |
| K03781 | KSG66\_18465 | katE, CAT, catB, srpA; catalase [EC:1.11.1.6] |
| K03783 | KSG66\_10970 | punA, PNP; purine-nucleoside phosphorylase [EC:2.4.2.1] |
| K03784 | KSG66\_09785 | deoD; purine-nucleoside phosphorylase [EC:2.4.2.1] |
| K03785 | KSG66\_03920 | aroD; 3-dehydroquinate dehydratase I [EC:4.2.1.10] |
| K03794 | KSG66\_07805 | sirB; sirohydrochlorin ferrochelatase [EC:4.99.1.4] |
| K03800 | KSG66\_05155 | lplA, lplJ; lipoate---protein ligase [EC:6.3.1.20] |
| K03809 | KSG66\_04580 | wrbA; NAD(P)H dehydrogenase (quinone) [EC:1.6.5.2] |
| K03816 | KSG66\_10290 | xpt; xanthine phosphoribosyltransferase [EC:2.4.2.22] |
| K03851 | KSG66\_04675 | tpa; taurine-pyruvate aminotransferase [EC:2.6.1.77] |
| K03886 | KSG66\_10525 | MQCRA, qcrA, bfcA, petC; menaquinol-cytochrome c reductase iron-sulfur subunit [EC:1.10.2.-] |
| K03887 | KSG66\_10520 | MQCRB, qcrB, bfcB, petB; menaquinol-cytochrome c reductase cytochrome b subunit |
| K03888 | KSG66\_10515 | MQCRC, qcrC, bfcC, petD; menaquinol-cytochrome c reductase cytochrome b/c subunit |
| K03897 | KSG66\_04945 | iucD; lysine N6-hydroxylase [EC:1.14.13.59] |
| K04041 | KSG66\_18855 | fbp3; fructose-1,6-bisphosphatase III [EC:3.1.3.11] |
| K04042 | KSG66\_00315 | glmU; bifunctional UDP-N-acetylglucosamine pyrophosphorylase / Glucosamine-1-phosphate N-acetyltransferase [EC:2.7.7.23 2.3.1.157] |
| K04091 | KSG66\_04495 | ssuD; alkanesulfonate monooxygenase [EC:1.14.14.5] |
| K04486 | KSG66\_13595 | E3.1.3.15B; histidinol-phosphatase (PHP family) [EC:3.1.3.15] |
| K04487 | KSG66\_12610 | iscS, NFS1; cysteine desulfurase [EC:2.8.1.7] |
| K04487 | KSG66\_12770 | iscS, NFS1; cysteine desulfurase [EC:2.8.1.7] |
| K04517 | KSG66\_10550 | tyrA2; prephenate dehydrogenase [EC:1.3.1.12] |
| K04518 | KSG66\_12780 | pheA2; prephenate dehydratase [EC:4.2.1.51] |
| K05366 | KSG66\_10400 | mrcA; penicillin-binding protein 1A [EC:2.4.1.129 3.4.16.4] |
| K05375 | KSG66\_14815 | mbtH, nocI; MbtH protein |
| K05606 | KSG66\_11290 | MCEE, epi; methylmalonyl-CoA/ethylmalonyl-CoA epimerase [EC:5.1.99.1] |
| K05607 | KSG66\_09250 | AUH; methylglutaconyl-CoA hydratase [EC:4.2.1.18] |
| K05822 | KSG66\_07070 | dapH, dapD; tetrahydrodipicolinate N-acetyltransferase [EC:2.3.1.89] |
| K05823 | KSG66\_07075 | dapL; N-acetyldiaminopimelate deacetylase [EC:3.5.1.47] |
| K05825 | KSG66\_16095 | LYSN; 2-aminoadipate transaminase [EC:2.6.1.-] |
| K05887 | KSG66\_03915 | ydiB; quinate/shikimate dehydrogenase [EC:1.1.1.282] |
| K06131 | KSG66\_17265 | clsA\_B; cardiolipin synthase A/B [EC:2.7.8.-] |
| K06131 | KSG66\_17560 | clsA\_B; cardiolipin synthase A/B [EC:2.7.8.-] |
| K06131 | KSG66\_17580 | clsA\_B; cardiolipin synthase A/B [EC:2.7.8.-] |
| K06208 | KSG66\_10590 | aroH; chorismate mutase [EC:5.4.99.5] |
| K06209 | KSG66\_12785 | pheB; chorismate mutase [EC:5.4.99.5] |
| K06606 | KSG66\_18710 | iolI; 2-keto-myo-inositol isomerase [EC:5.3.99.11] |
| K06881 | KSG66\_13480 | nrnA; bifunctional oligoribonuclease and PAP phosphatase NrnA [EC:3.1.3.7 3.1.13.3] |
| K06920 | KSG66\_06840 | queC; 7-cyano-7-deazaguanine synthase [EC:6.3.4.20] |
| K06949 | KSG66\_07885 | rsgA, engC; ribosome biogenesis GTPase / thiamine phosphate phosphatase [EC:3.6.1.- 3.1.3.100] |
| K07029 | KSG66\_03490 | dagK; diacylglycerol kinase (ATP) [EC:2.7.1.107] |
| K07104 | KSG66\_04115 | catE; catechol 2,3-dioxygenase [EC:1.13.11.2] |
| K07106 | KSG66\_01075 | murQ; N-acetylmuramic acid 6-phosphate etherase [EC:4.2.1.126] |
| K07160 | KSG66\_02135 | K07160; UPF0271 protein |
| K07173 | KSG66\_14140 | luxS; S-ribosylhomocysteine lyase [EC:4.4.1.21] |
| K07232 | KSG66\_07215 | CHAC, chaC; glutathione-specific gamma-glutamylcyclotransferase [EC:4.3.2.7] |
| K07246 | KSG66\_02965 | ttuC, dmlA; tartrate dehydrogenase/decarboxylase / D-malate dehydrogenase [EC:1.1.1.93 4.1.1.73 1.1.1.83] |
| K07250 | KSG66\_02065 | gabT; 4-aminobutyrate aminotransferase / (S)-3-amino-2-methylpropionate transaminase / 5-aminovalerate transaminase [EC:2.6.1.19 2.6.1.22 2.6.1.48] |
| K07258 | KSG66\_00075 | dacC, dacA, dacD; serine-type D-Ala-D-Ala carboxypeptidase (penicillin-binding protein 5/6) [EC:3.4.16.4] |
| K07258 | KSG66\_10830 | dacC, dacA, dacD; serine-type D-Ala-D-Ala carboxypeptidase (penicillin-binding protein 5/6) [EC:3.4.16.4] |
| K07258 | KSG66\_10965 | dacC, dacA, dacD; serine-type D-Ala-D-Ala carboxypeptidase (penicillin-binding protein 5/6) [EC:3.4.16.4] |
| K07260 | KSG66\_09780 | vanY; zinc D-Ala-D-Ala carboxypeptidase [EC:3.4.17.14] |
| K07406 | KSG66\_13935 | melA; alpha-galactosidase [EC:3.2.1.22] |
| K07516 | KSG66\_15290 | fadN; 3-hydroxyacyl-CoA dehydrogenase [EC:1.1.1.35] |
| K08093 | KSG66\_01825 | hxlA; 3-hexulose-6-phosphate synthase [EC:4.1.2.43] |
| K08094 | KSG66\_01820 | hxlB; 6-phospho-3-hexuloisomerase [EC:5.3.1.27] |
| K08289 | KSG66\_01345 | purT; phosphoribosylglycinamide formyltransferase 2 [EC:2.1.2.2] |
| K08591 | KSG66\_09030 | plsY; acyl phosphate:glycerol-3-phosphate acyltransferase [EC:2.3.1.275] |
| K08680 | KSG66\_14195 | menH; 2-succinyl-6-hydroxy-2,4-cyclohexadiene-1-carboxylate synthase [EC:4.2.99.20] |
| K08693 | KSG66\_03900 | yfkN; 2',3'-cyclic-nucleotide 2'-phosphodiesterase / 3'-nucleotidase / 5'-nucleotidase [EC:3.1.4.16 3.1.3.6 3.1.3.5] |
| K08963 | KSG66\_06760 | mtnA; methylthioribose-1-phosphate isomerase [EC:5.3.1.23] |
| K08964 | KSG66\_06790 | mtnB; methylthioribulose-1-phosphate dehydratase [EC:4.2.1.109] |
| K08965 | KSG66\_06780 | mtnW; 2,3-diketo-5-methylthiopentyl-1-phosphate enolase [EC:5.3.2.5] |
| K08966 | KSG66\_06785 | mtnX; 2-hydroxy-3-keto-5-methylthiopentenyl-1-phosphate phosphatase [EC:3.1.3.87] |
| K08967 | KSG66\_06795 | mtnD, mtnZ, ADI1; 1,2-dihydroxy-3-keto-5-methylthiopentene dioxygenase [EC:1.13.11.53 1.13.11.54] |
| K08969 | KSG66\_06775 | mtnE, mtnV; aminotransferase [EC:2.6.1.-] |
| K09457 | KSG66\_06855 | queF; 7-cyano-7-deazaguanine reductase [EC:1.7.1.13] |
| K09458 | KSG66\_02925 | fabF; 3-oxoacyl-[acyl-carrier-protein] synthase II [EC:2.3.1.179] |
| K09458 | KSG66\_05610 | fabF; 3-oxoacyl-[acyl-carrier-protein] synthase II [EC:2.3.1.179] |
| K09698 | KSG66\_00610 | gltX; nondiscriminating glutamyl-tRNA synthetase [EC:6.1.1.24] |
| K09699 | KSG66\_11335 | DBT, bkdB; 2-oxoisovalerate dehydrogenase E2 component (dihydrolipoyl transacylase) [EC:2.3.1.168] |
| K09903 | KSG66\_08255 | pyrH; uridylate kinase [EC:2.7.4.22] |
| K10026 | KSG66\_06850 | queE; 7-carboxy-7-deazaguanine synthase [EC:4.3.99.3] |
| K10780 | KSG66\_04270 | fabL; enoyl-[acyl-carrier protein] reductase III [EC:1.3.1.104] |
| K10810 | KSG66\_05780 | tenI; thiazole tautomerase (transcriptional regulator TenI) [EC:5.3.99.10] |
| K11175 | KSG66\_03390 | purN; phosphoribosylglycinamide formyltransferase 1 [EC:2.1.2.2] |
| K11358 | KSG66\_04815 | yhdR; aspartate aminotransferase [EC:2.6.1.1] |
| K11440 | KSG66\_14365 | gbsB; choline dehydrogenase [EC:1.1.1.1] |
| K11473 | KSG66\_13185 | glcF; glycolate oxidase iron-sulfur subunit |
| K11717 | KSG66\_15210 | sufS; cysteine desulfurase / selenocysteine lyase [EC:2.8.1.7 4.4.1.16] |
| K11752 | KSG66\_10880 | ribD; diaminohydroxyphosphoribosylaminopyrimidine deaminase / 5-amino-6-(5-phosphoribosylamino)uracil reductase [EC:3.5.4.26 1.1.1.193] |
| K11753 | KSG66\_08335 | ribF; riboflavin kinase / FMN adenylyltransferase [EC:2.7.1.26 2.7.7.2] |
| K11754 | KSG66\_12870 | folC; dihydrofolate synthase / folylpolyglutamate synthase [EC:6.3.2.12 6.3.2.17] |
| K11755 | KSG66\_16420 | hisIE; phosphoribosyl-ATP pyrophosphohydrolase / phosphoribosyl-AMP cyclohydrolase [EC:3.6.1.31 3.5.4.19] |
| K12555 | KSG66\_05095 | pbp2A; penicillin-binding protein 2A [EC:2.4.1.129 3.4.16.4] |
| K13010 | KSG66\_15945 | per, rfbE; perosamine synthetase [EC:2.6.1.102] |
| K13015 | KSG66\_15960 | wbpA; UDP-N-acetyl-D-glucosamine dehydrogenase [EC:1.1.1.136] |
| K13037 | KSG66\_17810 | bacD; L-alanine-L-anticapsin ligase [EC:6.3.2.49] |
| K13038 | KSG66\_07845 | coaBC, dfp; phosphopantothenoylcysteine decarboxylase / phosphopantothenate---cysteine ligase [EC:4.1.1.36 6.3.2.5] |
| K13542 | KSG66\_01750 | cobA-hemD; uroporphyrinogen III methyltransferase / synthase [EC:2.1.1.107 4.2.1.75] |
| K13566 | KSG66\_06770 | NIT2, yafV; omega-amidase [EC:3.5.1.3] |
| K13767 | KSG66\_13105 | fadB; enoyl-CoA hydratase [EC:4.2.1.17] |
| K13789 | KSG66\_11490 | GGPS; geranylgeranyl diphosphate synthase, type II [EC:2.5.1.1 2.5.1.10 2.5.1.29] |
| K13853 | KSG66\_13760 | aroG, aroA; 3-deoxy-7-phosphoheptulonate synthase / chorismate mutase [EC:2.5.1.54 5.4.99.5] |
| K13953 | KSG66\_08815 | adhP; alcohol dehydrogenase, propanol-preferring [EC:1.1.1.1] |
| K14155 | KSG66\_14570 | patB, malY; cysteine-S-conjugate beta-lyase [EC:4.4.1.13] |
| K14188 | KSG66\_18210 | dltC; D-alanine--poly(phosphoribitol) ligase subunit 2 [EC:6.1.1.13] |
| K14274 | KSG66\_15500 | xylC; xylonolactonase [EC:3.1.1.-] |
| K14652 | KSG66\_10870 | ribBA; 3,4-dihydroxy 2-butanone 4-phosphate synthase / GTP cyclohydrolase II [EC:4.1.99.12 3.5.4.25] |
| K15519 | KSG66\_00105 | dck; deoxyadenosine/deoxycytidine kinase [EC:2.7.1.76 2.7.1.74] |
| K15633 | KSG66\_15975 | gpmI; 2,3-bisphosphoglycerate-independent phosphoglycerate mutase [EC:5.4.2.12] |
| K15652 | KSG66\_01700 | asbF; 3-dehydroshikimate dehydratase [EC:4.2.1.118] |
| K15866 | KSG66\_04965 | paaG; 2-(1,2-epoxy-1,2-dihydrophenyl)acetyl-CoA isomerase [EC:5.3.3.18] |
| K15894 | KSG66\_10065 | pseB; UDP-N-acetylglucosamine 4,6-dehydratase [EC:4.2.1.115] |
| K15921 | KSG66\_09155 | xynD; arabinoxylan arabinofuranohydrolase [EC:3.2.1.55] |
| K16044 | KSG66\_15690 | iolW; scyllo-inositol 2-dehydrogenase (NADP+) [EC:1.1.1.371] |
| K16150 | KSG66\_19105 | K16150; glycogen synthase [EC:2.4.1.11] |
| K16593 | KSG66\_09200 | bioI, CYP107H; pimeloyl-[acyl-carrier protein] synthase [EC:1.14.14.46] |
| K16869 | KSG66\_17770 | lipL; octanoyl-[GcvH]:protein N-octanoyltransferase [EC:2.3.1.204] |
| K17103 | KSG66\_01350 | CHO1, pssA; CDP-diacylglycerol---serine O-phosphatidyltransferase [EC:2.7.8.8] |
| K17216 | KSG66\_12475 | mccA; cystathionine beta-synthase (O-acetyl-L-serine) [EC:2.5.1.134] |
| K17217 | KSG66\_12470 | mccB; cystathionine gamma-lyase / homocysteine desulfhydrase [EC:4.4.1.1 4.4.1.2] |
| K17828 | KSG66\_07765 | pyrDI; dihydroorotate dehydrogenase (NAD+) catalytic subunit [EC:1.3.1.14] |
| K19005 | KSG66\_03645 | ltaS; lipoteichoic acid synthase [EC:2.7.8.20] |
| K19005 | KSG66\_03845 | ltaS; lipoteichoic acid synthase [EC:2.7.8.20] |
| K19005 | KSG66\_15575 | ltaS; lipoteichoic acid synthase [EC:2.7.8.20] |
| K19222 | KSG66\_14680 | menI, DHNAT; 1,4-dihydroxy-2-naphthoyl-CoA hydrolase [EC:3.1.2.28] |
| K19285 | KSG66\_18025 | nfrA1; FMN reductase (NADPH) [EC:1.5.1.38] |
| K19286 | KSG66\_02045 | nfrA2; FMN reductase [NAD(P)H] [EC:1.5.1.39] |
| K19546 | KSG66\_17825 | bacA; prephenate decarboxylase [EC:4.1.1.100] |
| K19547 | KSG66\_17820 | bacB; 3-[(4R)-4-hydroxycyclohexa-1,5-dien-1-yl]-2-oxopropanoate isomerase [EC:5.3.3.19] |
| K19548 | KSG66\_17815 | bacC; dihydroanticapsin dehydrogenase [EC:1.1.1.385] |
| K19549 | KSG66\_17800 | bacF; bacilysin biosynthesis transaminase BacF [EC:2.6.1.-] |
| K19550 | KSG66\_17795 | bacG; bacilysin biosynthesis oxidoreductase BacG [EC:1.3.1.-] |
| K19563 | KSG66\_09220 | bioA, bioK; lysine---8-amino-7-oxononanoate aminotransferase [EC:2.6.1.105] |
| K19745 | KSG66\_03055 | acuI; acrylyl-CoA reductase (NADPH) [EC:1.3.1.-] |
| K20118 | KSG66\_06930 | PTS-Glc1-EIIC, ptsG, glcA, glcB; PTS system, glucose-specific IIC component |
| K20895 | KSG66\_07670 | ylmB; formylaminopyrimidine deformylase [EC:3.5.1.-] |
| K21064 | KSG66\_02130 | ycsE, yitU, ywtE; 5-amino-6-(5-phospho-D-ribitylamino)uracil phosphatase [EC:3.1.3.104] |
| K21064 | KSG66\_05505 | ycsE, yitU, ywtE; 5-amino-6-(5-phospho-D-ribitylamino)uracil phosphatase [EC:3.1.3.104] |
| K21064 | KSG66\_16885 | ycsE, yitU, ywtE; 5-amino-6-(5-phospho-D-ribitylamino)uracil phosphatase [EC:3.1.3.104] |
| K22230 | KSG66\_14425 | iolU; scyllo-inositol 2-dehydrogenase (NADP+) [EC:1.1.1.-] |
| K22602 | KSG66\_17025 | hpxW; oxamate amidohydrolase [EC:3.5.1.126] |
| K23257 | KSG66\_13350 |  |
| K23257 | KSG66\_15620 |  |
| K23264 | KSG66\_03365 |  |
| K23265 | KSG66\_03370 |  |
| K23269 | KSG66\_03375 |  |
| K23734 | KSG66\_11610 |  |
| K24042 | KSG66\_05445 |  |

| 2 | Biosynthesis of secondary metabolites | ko01110 || K00010 | KSG66\_18720 | iolG; myo-inositol 2-dehydrogenase / D-chiro-inositol 1-dehydrogenase [EC:1.1.1.18 1.1.1.369] |
| K00013 | KSG66\_16445 | hisD; histidinol dehydrogenase [EC:1.1.1.23] |
| K00014 | KSG66\_12170 | aroE; shikimate dehydrogenase [EC:1.1.1.25] |
| K00016 | KSG66\_01650 | LDH, ldh; L-lactate dehydrogenase [EC:1.1.1.27] |
| K00024 | KSG66\_13385 | mdh; malate dehydrogenase [EC:1.1.1.37] |
| K00031 | KSG66\_13390 | IDH1, IDH2, icd; isocitrate dehydrogenase [EC:1.1.1.42] |
| K00033 | KSG66\_11260 | PGD, gnd, gntZ; 6-phosphogluconate dehydrogenase [EC:1.1.1.44 1.1.1.343] |
| K00036 | KSG66\_11255 | G6PD, zwf; glucose-6-phosphate 1-dehydrogenase [EC:1.1.1.49 1.1.1.363] |
| K00052 | KSG66\_12970 | leuB, IMDH; 3-isopropylmalate dehydrogenase [EC:1.1.1.85] |
| K00053 | KSG66\_12980 | ilvC; ketol-acid reductoisomerase [EC:1.1.1.86] |
| K00057 | KSG66\_10660 | gpsA; glycerol-3-phosphate dehydrogenase (NAD(P)+) [EC:1.1.1.94] |
| K00058 | KSG66\_09435 | serA, PHGDH; D-3-phosphoglycerate dehydrogenase / 2-oxoglutarate reductase [EC:1.1.1.95 1.1.1.399] |
| K00058 | KSG66\_10775 | serA, PHGDH; D-3-phosphoglycerate dehydrogenase / 2-oxoglutarate reductase [EC:1.1.1.95 1.1.1.399] |
| K00059 | KSG66\_07950 | fabG; 3-oxoacyl-[acyl-carrier protein] reductase [EC:1.1.1.100] |
| K00059 | KSG66\_08440 | fabG; 3-oxoacyl-[acyl-carrier protein] reductase [EC:1.1.1.100] |
| K00059 | KSG66\_09410 | fabG; 3-oxoacyl-[acyl-carrier protein] reductase [EC:1.1.1.100] |
| K00059 | KSG66\_11195 | fabG; 3-oxoacyl-[acyl-carrier protein] reductase [EC:1.1.1.100] |
| K00059 | KSG66\_13500 | fabG; 3-oxoacyl-[acyl-carrier protein] reductase [EC:1.1.1.100] |
| K00059 | KSG66\_15330 | fabG; 3-oxoacyl-[acyl-carrier protein] reductase [EC:1.1.1.100] |
| K00059 | KSG66\_15750 | fabG; 3-oxoacyl-[acyl-carrier protein] reductase [EC:1.1.1.100] |
| K00067 | KSG66\_17890 | rfbD, rmlD; dTDP-4-dehydrorhamnose reductase [EC:1.1.1.133] |
| K00077 | KSG66\_07185 | panE, apbA; 2-dehydropantoate 2-reductase [EC:1.1.1.169] |
| K00077 | KSG66\_07565 | panE, apbA; 2-dehydropantoate 2-reductase [EC:1.1.1.169] |
| K00077 | KSG66\_15355 | panE, apbA; 2-dehydropantoate 2-reductase [EC:1.1.1.169] |
| K00088 | KSG66\_00070 | IMPDH, guaB; IMP dehydrogenase [EC:1.1.1.205] |
| K00090 | KSG66\_16350 | ghrB; glyoxylate/hydroxypyruvate/2-ketogluconate reductase [EC:1.1.1.79 1.1.1.81 1.1.1.215] |
| K00099 | KSG66\_08275 | dxr; 1-deoxy-D-xylulose-5-phosphate reductoisomerase [EC:1.1.1.267] |
| K00104 | KSG66\_13180 | glcD; glycolate oxidase [EC:1.1.3.15] |
| K00111 | KSG66\_04695 | glpA, glpD; glycerol-3-phosphate dehydrogenase [EC:1.1.5.3] |
| K00121 | KSG66\_01785 | frmA, ADH5, adhC; S-(hydroxymethyl)glutathione dehydrogenase / alcohol dehydrogenase [EC:1.1.1.284 1.1.1.1] |
| K00121 | KSG66\_02865 | frmA, ADH5, adhC; S-(hydroxymethyl)glutathione dehydrogenase / alcohol dehydrogenase [EC:1.1.1.284 1.1.1.1] |
| K00128 | KSG66\_09600 | ALDH; aldehyde dehydrogenase (NAD+) [EC:1.2.1.3] |
| K00128 | KSG66\_13620 | ALDH; aldehyde dehydrogenase (NAD+) [EC:1.2.1.3] |
| K00128 | KSG66\_18800 | ALDH; aldehyde dehydrogenase (NAD+) [EC:1.2.1.3] |
| K00133 | KSG66\_08375 | asd; aspartate-semialdehyde dehydrogenase [EC:1.2.1.11] |
| K00134 | KSG66\_13335 | GAPDH, gapA; glyceraldehyde 3-phosphate dehydrogenase [EC:1.2.1.12] |
| K00134 | KSG66\_15990 | GAPDH, gapA; glyceraldehyde 3-phosphate dehydrogenase [EC:1.2.1.12] |
| K00145 | KSG66\_05535 | argC; N-acetyl-gamma-glutamyl-phosphate reductase [EC:1.2.1.38] |
| K00147 | KSG66\_06570 | proA; glutamate-5-semialdehyde dehydrogenase [EC:1.2.1.41] |
| K00161 | KSG66\_07305 | PDHA, pdhA; pyruvate dehydrogenase E1 component alpha subunit [EC:1.2.4.1] |
| K00162 | KSG66\_07310 | PDHB, pdhB; pyruvate dehydrogenase E1 component beta subunit [EC:1.2.4.1] |
| K00164 | KSG66\_09635 | OGDH, sucA; 2-oxoglutarate dehydrogenase E1 component [EC:1.2.4.2] |
| K00166 | KSG66\_11345 | BCKDHA, bkdA1; 2-oxoisovalerate dehydrogenase E1 component alpha subunit [EC:1.2.4.4] |
| K00167 | KSG66\_11340 | BCKDHB, bkdA2; 2-oxoisovalerate dehydrogenase E1 component beta subunit [EC:1.2.4.4] |
| K00208 | KSG66\_05810 | fabI; enoyl-[acyl-carrier protein] reductase I [EC:1.3.1.9 1.3.1.10] |
| K00215 | KSG66\_10485 | dapB; 4-hydroxy-tetrahydrodipicolinate reductase [EC:1.17.1.8] |
| K00216 | KSG66\_14840 | entA; 2,3-dihydro-2,3-dihydroxybenzoate dehydrogenase [EC:1.3.1.28] |
| K00231 | KSG66\_05110 | PPOX, hemY; protoporphyrinogen/coproporphyrinogen III oxidase [EC:1.3.3.4 1.3.3.15] |
| K00239 | KSG66\_13060 | sdhA, frdA; succinate dehydrogenase / fumarate reductase, flavoprotein subunit [EC:1.3.5.1 1.3.5.4] |
| K00240 | KSG66\_13055 | sdhB, frdB; succinate dehydrogenase / fumarate reductase, iron-sulfur subunit [EC:1.3.5.1 1.3.5.4] |
| K00241 | KSG66\_13065 | sdhC, frdC; succinate dehydrogenase / fumarate reductase, cytochrome b subunit |
| K00263 | KSG66\_11360 | E1.4.1.9; leucine dehydrogenase [EC:1.4.1.9] |
| K00265 | KSG66\_09385 | gltB; glutamate synthase (NADPH) large chain [EC:1.4.1.13] |
| K00266 | KSG66\_09380 | gltD; glutamate synthase (NADPH) small chain [EC:1.4.1.13] |
| K00282 | KSG66\_11625 | gcvPA; glycine dehydrogenase subunit 1 [EC:1.4.4.2] |
| K00283 | KSG66\_11620 | gcvPB; glycine dehydrogenase subunit 2 [EC:1.4.4.2] |
| K00286 | KSG66\_09400 | proC; pyrroline-5-carboxylate reductase [EC:1.5.1.2] |
| K00286 | KSG66\_11140 | proC; pyrroline-5-carboxylate reductase [EC:1.5.1.2] |
| K00318 | KSG66\_01725 | PRODH; proline dehydrogenase [EC:1.5.-.-] |
| K00318 | KSG66\_15300 | PRODH; proline dehydrogenase [EC:1.5.-.-] |
| K00355 | KSG66\_04130 | NQO1; NAD(P)H dehydrogenase (quinone) [EC:1.6.5.2] |
| K00382 | KSG66\_04065 | DLD, lpd, pdhD; dihydrolipoamide dehydrogenase [EC:1.8.1.4] |
| K00382 | KSG66\_07320 | DLD, lpd, pdhD; dihydrolipoamide dehydrogenase [EC:1.8.1.4] |
| K00382 | KSG66\_11350 | DLD, lpd, pdhD; dihydrolipoamide dehydrogenase [EC:1.8.1.4] |
| K00435 | KSG66\_17790 | hemQ; Fe-coproporphyrin III decarboxylase [EC:1.11.1.-] |
| K00491 | KSG66\_03800 | nos; nitric-oxide synthase, bacterial [EC:1.14.14.47] |
| K00547 | KSG66\_01390 | mmuM, BHMT2; homocysteine S-methyltransferase [EC:2.1.1.10] |
| K00549 | KSG66\_06615 | metE; 5-methyltetrahydropteroyltriglutamate--homocysteine methyltransferase [EC:2.1.1.14] |
| K00549 | KSG66\_18440 | metE; 5-methyltetrahydropteroyltriglutamate--homocysteine methyltransferase [EC:2.1.1.14] |
| K00600 | KSG66\_17420 | glyA, SHMT; glycine hydroxymethyltransferase [EC:2.1.2.1] |
| K00602 | KSG66\_03395 | purH; phosphoribosylaminoimidazolecarboxamide formyltransferase / IMP cyclohydrolase [EC:2.1.2.3 3.5.4.10] |
| K00605 | KSG66\_11630 | gcvT, AMT; aminomethyltransferase [EC:2.1.2.10] |
| K00606 | KSG66\_10455 | panB; 3-methyl-2-oxobutanoate hydroxymethyltransferase [EC:2.1.2.11] |
| K00611 | KSG66\_05565 | OTC, argF, argI; ornithine carbamoyltransferase [EC:2.1.3.3] |
| K00615 | KSG66\_08935 | E2.2.1.1, tktA, tktB; transketolase [EC:2.2.1.1] |
| K00616 | KSG66\_17525 | E2.2.1.2, talA, talB; transaldolase [EC:2.2.1.2] |
| K00620 | KSG66\_05540 | argJ; glutamate N-acetyltransferase / amino-acid N-acetyltransferase [EC:2.3.1.35 2.3.1.1] |
| K00626 | KSG66\_05195 | E2.3.1.9, atoB; acetyl-CoA C-acetyltransferase [EC:2.3.1.9] |
| K00626 | KSG66\_11405 | E2.3.1.9, atoB; acetyl-CoA C-acetyltransferase [EC:2.3.1.9] |
| K00627 | KSG66\_04060 | DLAT, aceF, pdhC; pyruvate dehydrogenase E2 component (dihydrolipoamide acetyltransferase) [EC:2.3.1.12] |
| K00627 | KSG66\_07315 | DLAT, aceF, pdhC; pyruvate dehydrogenase E2 component (dihydrolipoamide acetyltransferase) [EC:2.3.1.12] |
| K00632 | KSG66\_15285 | fadA, fadI; acetyl-CoA acyltransferase [EC:2.3.1.16] |
| K00640 | KSG66\_00615 | cysE; serine O-acetyltransferase [EC:2.3.1.30] |
| K00645 | KSG66\_07945 | fabD; [acyl-carrier-protein] S-malonyltransferase [EC:2.3.1.39] |
| K00645 | KSG66\_09175 | fabD; [acyl-carrier-protein] S-malonyltransferase [EC:2.3.1.39] |
| K00651 | KSG66\_10170 | metA; homoserine O-succinyltransferase/O-acetyltransferase [EC:2.3.1.46 2.3.1.31] |
| K00655 | KSG66\_04800 | plsC; 1-acyl-sn-glycerol-3-phosphate acyltransferase [EC:2.3.1.51] |
| K00655 | KSG66\_10120 | plsC; 1-acyl-sn-glycerol-3-phosphate acyltransferase [EC:2.3.1.51] |
| K00658 | KSG66\_09630 | DLST, sucB; 2-oxoglutarate dehydrogenase E2 component (dihydrolipoamide succinyltransferase) [EC:2.3.1.61] |
| K00760 | KSG66\_00415 | hprT, hpt, HPRT1; hypoxanthine phosphoribosyltransferase [EC:2.4.2.8] |
| K00764 | KSG66\_03380 | purF, PPAT; amidophosphoribosyltransferase [EC:2.4.2.14] |
| K00765 | KSG66\_16450 | hisG; ATP phosphoribosyltransferase [EC:2.4.2.17] |
| K00766 | KSG66\_10580 | trpD; anthranilate phosphoribosyltransferase [EC:2.4.2.18] |
| K00789 | KSG66\_14080 | metK; S-adenosylmethionine synthetase [EC:2.5.1.6] |
| K00791 | KSG66\_08660 | miaA, TRIT1; tRNA dimethylallyltransferase [EC:2.5.1.75] |
| K00793 | KSG66\_10875 | ribE, RIB5; riboflavin synthase [EC:2.5.1.9] |
| K00794 | KSG66\_10865 | ribH, RIB4; 6,7-dimethyl-8-ribityllumazine synthase [EC:2.5.1.78] |
| K00800 | KSG66\_10545 | aroA; 3-phosphoshikimate 1-carboxyvinyltransferase [EC:2.5.1.19] |
| K00805 | KSG66\_10615 | hepST; heptaprenyl diphosphate synthase [EC:2.5.1.30] |
| K00805 | KSG66\_10625 | hepST; heptaprenyl diphosphate synthase [EC:2.5.1.30] |
| K00806 | KSG66\_08265 | uppS; undecaprenyl diphosphate synthase [EC:2.5.1.31] |
| K00812 | KSG66\_10425 | aspB; aspartate aminotransferase [EC:2.6.1.1] |
| K00817 | KSG66\_10555 | hisC; histidinol-phosphate aminotransferase [EC:2.6.1.9] |
| K00819 | KSG66\_18965 | rocD, OAT; ornithine--oxo-acid transaminase [EC:2.6.1.13] |
| K00821 | KSG66\_05550 | argD; acetylornithine/N-succinyldiaminopimelate aminotransferase [EC:2.6.1.11 2.6.1.17] |
| K00826 | KSG66\_01380 | E2.6.1.42, ilvE; branched-chain amino acid aminotransferase [EC:2.6.1.42] |
| K00826 | KSG66\_18220 | E2.6.1.42, ilvE; branched-chain amino acid aminotransferase [EC:2.6.1.42] |
| K00831 | KSG66\_05055 | serC, PSAT1; phosphoserine aminotransferase [EC:2.6.1.52] |
| K00841 | KSG66\_06980 | patA; aminotransferase [EC:2.6.1.-] |
| K00845 | KSG66\_11770 | glk; glucokinase [EC:2.7.1.2] |
| K00847 | KSG66\_03210 | E2.7.1.4, scrK; fructokinase [EC:2.7.1.4] |
| K00850 | KSG66\_13440 | pfkA, PFK; 6-phosphofructokinase 1 [EC:2.7.1.11] |
| K00851 | KSG66\_16055 | E2.7.1.12, gntK, idnK; gluconokinase [EC:2.7.1.12] |
| K00865 | KSG66\_00095 | glxK, garK; glycerate 2-kinase [EC:2.7.1.165] |
| K00872 | KSG66\_14990 | thrB1; homoserine kinase [EC:2.7.1.39] |
| K00873 | KSG66\_13435 | PK, pyk; pyruvate kinase [EC:2.7.1.40] |
| K00891 | KSG66\_01710 | E2.7.1.71, aroK, aroL; shikimate kinase [EC:2.7.1.71] |
| K00919 | KSG66\_00295 | ispE; 4-diphosphocytidyl-2-C-methyl-D-erythritol kinase [EC:2.7.1.148] |
| K00927 | KSG66\_15985 | PGK, pgk; phosphoglycerate kinase [EC:2.7.2.3] |
| K00928 | KSG66\_02010 | lysC; aspartate kinase [EC:2.7.2.4] |
| K00928 | KSG66\_08380 | lysC; aspartate kinase [EC:2.7.2.4] |
| K00928 | KSG66\_13075 | lysC; aspartate kinase [EC:2.7.2.4] |
| K00930 | KSG66\_05545 | argB; acetylglutamate kinase [EC:2.7.2.8] |
| K00931 | KSG66\_06565 | proB; glutamate 5-kinase [EC:2.7.2.11] |
| K00931 | KSG66\_09395 | proB; glutamate 5-kinase [EC:2.7.2.11] |
| K00939 | KSG66\_00835 | adk, AK; adenylate kinase [EC:2.7.4.3] |
| K00940 | KSG66\_10610 | ndk, NME; nucleoside-diphosphate kinase [EC:2.7.4.6] |
| K00948 | KSG66\_00320 | PRPS, prsA; ribose-phosphate pyrophosphokinase [EC:2.7.6.1] |
| K00958 | KSG66\_07790 | sat, met3; sulfate adenylyltransferase [EC:2.7.7.4] |
| K00963 | KSG66\_16805 | UGP2, galU, galF; UTP--glucose-1-phosphate uridylyltransferase [EC:2.7.7.9] |
| K00973 | KSG66\_17900 | E2.7.7.24, rfbA, rffH; glucose-1-phosphate thymidylyltransferase [EC:2.7.7.24] |
| K00981 | KSG66\_08270 | E2.7.7.41, CDS1, CDS2, cdsA; phosphatidate cytidylyltransferase [EC:2.7.7.41] |
| K00991 | KSG66\_00600 | ispD; 2-C-methyl-D-erythritol 4-phosphate cytidylyltransferase [EC:2.7.7.60] |
| K01060 | KSG66\_01715 | cah; cephalosporin-C deacetylase [EC:3.1.1.41] |
| K01081 | KSG66\_04640 | E3.1.3.5; 5'-nucleotidase [EC:3.1.3.5] |
| K01081 | KSG66\_15045 | E3.1.3.5; 5'-nucleotidase [EC:3.1.3.5] |
| K01092 | KSG66\_01010 | E3.1.3.25, IMPA, suhB; myo-inositol-1(or 4)-monophosphatase [EC:3.1.3.25] |
| K01092 | KSG66\_07360 | E3.1.3.25, IMPA, suhB; myo-inositol-1(or 4)-monophosphatase [EC:3.1.3.25] |
| K01187 | KSG66\_14475 | malZ; alpha-glucosidase [EC:3.2.1.20] |
| K01193 | KSG66\_16265 | INV, sacA; beta-fructofuranosidase [EC:3.2.1.26] |
| K01193 | KSG66\_17995 | INV, sacA; beta-fructofuranosidase [EC:3.2.1.26] |
| K01208 | KSG66\_16320 | cd, ma, nplT; cyclomaltodextrinase / maltogenic alpha-amylase / neopullulanase [EC:3.2.1.54 3.2.1.133 3.2.1.135] |
| K01424 | KSG66\_01505 | E3.5.1.1, ansA, ansB; L-asparaginase [EC:3.5.1.1] |
| K01424 | KSG66\_11025 | E3.5.1.1, ansA, ansB; L-asparaginase [EC:3.5.1.1] |
| K01438 | KSG66\_09840 | argE; acetylornithine deacetylase [EC:3.5.1.16] |
| K01476 | KSG66\_18955 | E3.5.3.1, rocF, arg; arginase [EC:3.5.3.1] |
| K01579 | KSG66\_10445 | panD; aspartate 1-decarboxylase [EC:4.1.1.11] |
| K01586 | KSG66\_10910 | lysA; diaminopimelate decarboxylase [EC:4.1.1.20] |
| K01588 | KSG66\_03345 | purE; 5-(carboxyamino)imidazole ribonucleotide mutase [EC:5.4.99.18] |
| K01589 | KSG66\_03350 | purK; 5-(carboxyamino)imidazole ribonucleotide synthase [EC:6.3.4.18] |
| K01599 | KSG66\_05100 | hemE, UROD; uroporphyrinogen decarboxylase [EC:4.1.1.37] |
| K01609 | KSG66\_10575 | trpC; indole-3-glycerol phosphate synthase [EC:4.1.1.48] |
| K01610 | KSG66\_14085 | E4.1.1.49, pckA; phosphoenolpyruvate carboxykinase (ATP) [EC:4.1.1.49] |
| K01613 | KSG66\_01360 | psd, PISD; phosphatidylserine decarboxylase [EC:4.1.1.65] |
| K01624 | KSG66\_17530 | FBA, fbaA; fructose-bisphosphate aldolase, class II [EC:4.1.2.13] |
| K01640 | KSG66\_09255 | E4.1.3.4, HMGCL, hmgL; hydroxymethylglutaryl-CoA lyase [EC:4.1.3.4] |
| K01647 | KSG66\_04765 | CS, gltA; citrate synthase [EC:2.3.3.1] |
| K01647 | KSG66\_11390 | CS, gltA; citrate synthase [EC:2.3.3.1] |
| K01649 | KSG66\_12975 | leuA, IMS; 2-isopropylmalate synthase [EC:2.3.3.13] |
| K01652 | KSG66\_12990 | E2.2.1.6L, ilvB, ilvG, ilvI; acetolactate synthase I/II/III large subunit [EC:2.2.1.6] |
| K01652 | KSG66\_16980 | E2.2.1.6L, ilvB, ilvG, ilvI; acetolactate synthase I/II/III large subunit [EC:2.2.1.6] |
| K01653 | KSG66\_12985 | E2.2.1.6S, ilvH, ilvN; acetolactate synthase I/III small subunit [EC:2.2.1.6] |
| K01657 | KSG66\_10585 | trpE; anthranilate synthase component I [EC:4.1.3.27] |
| K01661 | KSG66\_14190 | menB; naphthoate synthase [EC:4.1.3.36] |
| K01662 | KSG66\_11485 | dxs; 1-deoxy-D-xylulose-5-phosphate synthase [EC:2.2.1.7] |
| K01679 | KSG66\_15415 | E4.2.1.2B, fumC, FH; fumarate hydratase, class II [EC:4.2.1.2] |
| K01681 | KSG66\_08990 | ACO, acnA; aconitate hydratase [EC:4.2.1.3] |
| K01687 | KSG66\_10150 | ilvD; dihydroxy-acid dehydratase [EC:4.2.1.9] |
| K01689 | KSG66\_15970 | ENO, eno; enolase [EC:4.2.1.11] |
| K01693 | KSG66\_16440 | hisB; imidazoleglycerol-phosphate dehydratase [EC:4.2.1.19] |
| K01695 | KSG66\_10560 | trpA; tryptophan synthase alpha chain [EC:4.2.1.20] |
| K01696 | KSG66\_10565 | trpB; tryptophan synthase beta chain [EC:4.2.1.20] |
| K01698 | KSG66\_12900 | hemB, ALAD; porphobilinogen synthase [EC:4.2.1.24] |
| K01703 | KSG66\_12965 | leuC, IPMI-L; 3-isopropylmalate/(R)-2-methylmalate dehydratase large subunit [EC:4.2.1.33 4.2.1.35] |
| K01704 | KSG66\_12960 | leuD, IPMI-S; 3-isopropylmalate/(R)-2-methylmalate dehydratase small subunit [EC:4.2.1.33 4.2.1.35] |
| K01710 | KSG66\_17895 | E4.2.1.46, rfbB, rffG; dTDP-glucose 4,6-dehydratase [EC:4.2.1.46] |
| K01714 | KSG66\_02785 | dapA; 4-hydroxy-tetrahydrodipicolinate synthase [EC:4.3.3.7] |
| K01714 | KSG66\_08385 | dapA; 4-hydroxy-tetrahydrodipicolinate synthase [EC:4.3.3.7] |
| K01719 | KSG66\_06085 | hemD, UROS; uroporphyrinogen-III synthase [EC:4.2.1.75] |
| K01719 | KSG66\_12905 | hemD, UROS; uroporphyrinogen-III synthase [EC:4.2.1.75] |
| K01733 | KSG66\_14995 | thrC; threonine synthase [EC:4.2.3.1] |
| K01735 | KSG66\_10595 | aroB; 3-dehydroquinate synthase [EC:4.2.3.4] |
| K01736 | KSG66\_10600 | aroC; chorismate synthase [EC:4.2.3.5] |
| K01738 | KSG66\_00440 | cysK; cysteine synthase [EC:2.5.1.47] |
| K01738 | KSG66\_13860 | cysK; cysteine synthase [EC:2.5.1.47] |
| K01739 | KSG66\_05910 | metB; cystathionine gamma-synthase [EC:2.5.1.48] |
| K01749 | KSG66\_12910 | hemC, HMBS; hydroxymethylbilane synthase [EC:2.5.1.61] |
| K01752 | KSG66\_07920 | E4.3.1.17, sdaA, sdaB, tdcG; L-serine dehydratase [EC:4.3.1.17] |
| K01752 | KSG66\_07925 | E4.3.1.17, sdaA, sdaB, tdcG; L-serine dehydratase [EC:4.3.1.17] |
| K01754 | KSG66\_10105 | E4.3.1.19, ilvA, tdcB; threonine dehydratase [EC:4.3.1.19] |
| K01755 | KSG66\_13505 | argH, ASL; argininosuccinate lyase [EC:4.3.2.1] |
| K01756 | KSG66\_03355 | purB, ADSL; adenylosuccinate lyase [EC:4.3.2.2] |
| K01760 | KSG66\_05915 | metC; cysteine-S-conjugate beta-lyase [EC:4.4.1.13] |
| K01770 | KSG66\_00605 | ispF; 2-C-methyl-D-erythritol 2,4-cyclodiphosphate synthase [EC:4.6.1.12] |
| K01772 | KSG66\_05105 | hemH, FECH; protoporphyrin/coproporphyrin ferrochelatase [EC:4.99.1.1 4.99.1.9] |
| K01778 | KSG66\_14955 | dapF; diaminopimelate epimerase [EC:5.1.1.7] |
| K01783 | KSG66\_07890 | rpe, RPE; ribulose-phosphate 3-epimerase [EC:5.1.3.1] |
| K01785 | KSG66\_09320 | galM, GALM; aldose 1-epimerase [EC:5.1.3.3] |
| K01790 | KSG66\_17885 | rfbC, rmlC; dTDP-4-dehydrorhamnose 3,5-epimerase [EC:5.1.3.13] |
| K01803 | KSG66\_15980 | TPI, tpiA; triosephosphate isomerase (TIM) [EC:5.3.1.1] |
| K01808 | KSG66\_17430 | rpiB; ribose 5-phosphate isomerase B [EC:5.3.1.6] |
| K01809 | KSG66\_12385 | manA, MPI; mannose-6-phosphate isomerase [EC:5.3.1.8] |
| K01809 | KSG66\_16850 | manA, MPI; mannose-6-phosphate isomerase [EC:5.3.1.8] |
| K01809 | KSG66\_18305 | manA, MPI; mannose-6-phosphate isomerase [EC:5.3.1.8] |
| K01810 | KSG66\_14515 | GPI, pgi; glucose-6-phosphate isomerase [EC:5.3.1.9] |
| K01814 | KSG66\_16430 | hisA; phosphoribosylformimino-5-aminoimidazole carboxamide ribotide isomerase [EC:5.3.1.16] |
| K01817 | KSG66\_10570 | trpF; phosphoribosylanthranilate isomerase [EC:5.3.1.24] |
| K01823 | KSG66\_10680 | idi, IDI; isopentenyl-diphosphate Delta-isomerase [EC:5.3.3.2] |
| K01835 | KSG66\_04700 | pgm; phosphoglucomutase [EC:5.4.2.2] |
| K01845 | KSG66\_04300 | hemL; glutamate-1-semialdehyde 2,1-aminomutase [EC:5.4.3.8] |
| K01845 | KSG66\_12895 | hemL; glutamate-1-semialdehyde 2,1-aminomutase [EC:5.4.3.8] |
| K01895 | KSG66\_13565 | ACSS, acs; acetyl-CoA synthetase [EC:6.2.1.1] |
| K01895 | KSG66\_13725 | ACSS, acs; acetyl-CoA synthetase [EC:6.2.1.1] |
| K01902 | KSG66\_08045 | sucD; succinyl-CoA synthetase alpha subunit [EC:6.2.1.5] |
| K01903 | KSG66\_08040 | sucC; succinyl-CoA synthetase beta subunit [EC:6.2.1.5] |
| K01911 | KSG66\_14185 | menE; O-succinylbenzoic acid---CoA ligase [EC:6.2.1.26] |
| K01918 | KSG66\_10450 | panC; pantoate--beta-alanine ligase [EC:6.3.2.1] |
| K01923 | KSG66\_03360 | purC; phosphoribosylaminoimidazole-succinocarboxamide synthase [EC:6.3.2.6] |
| K01933 | KSG66\_03385 | purM; phosphoribosylformylglycinamidine cyclo-ligase [EC:6.3.3.1] |
| K01940 | KSG66\_13510 | argG, ASS1; argininosuccinate synthase [EC:6.3.4.5] |
| K01945 | KSG66\_03400 | purD; phosphoribosylamine---glycine ligase [EC:6.3.4.13] |
| K01953 | KSG66\_05395 | asnB, ASNS; asparagine synthase (glutamine-hydrolysing) [EC:6.3.5.4] |
| K01953 | KSG66\_14075 | asnB, ASNS; asparagine synthase (glutamine-hydrolysing) [EC:6.3.5.4] |
| K01961 | KSG66\_09265 | accC; acetyl-CoA carboxylase, biotin carboxylase subunit [EC:6.4.1.2 6.3.4.14] |
| K01961 | KSG66\_11520 | accC; acetyl-CoA carboxylase, biotin carboxylase subunit [EC:6.4.1.2 6.3.4.14] |
| K01962 | KSG66\_13445 | accA; acetyl-CoA carboxylase carboxyl transferase subunit alpha [EC:6.4.1.2 2.1.3.15] |
| K01963 | KSG66\_13450 | accD; acetyl-CoA carboxylase carboxyl transferase subunit beta [EC:6.4.1.2 2.1.3.15] |
| K01966 | KSG66\_11285 | PCCB, pccB; propionyl-CoA carboxylase beta chain [EC:6.4.1.3 2.1.3.15] |
| K02078 | KSG66\_07955 | acpP; acyl carrier protein |
| K02078 | KSG66\_11205 | acpP; acyl carrier protein |
| K02160 | KSG66\_09260 | accB, bccP; acetyl-CoA carboxylase biotin carboxyl carrier protein |
| K02160 | KSG66\_11525 | accB, bccP; acetyl-CoA carboxylase biotin carboxyl carrier protein |
| K02257 | KSG66\_04120 | COX10, ctaB, cyoE; heme o synthase [EC:2.5.1.141] |
| K02257 | KSG66\_07450 | COX10, ctaB, cyoE; heme o synthase [EC:2.5.1.141] |
| K02259 | KSG66\_07445 | COX15, ctaA; cytochrome c oxidase assembly protein subunit 15 |
| K02291 | KSG66\_05400 | crtB; 15-cis-phytoene synthase [EC:2.5.1.32] |
| K02304 | KSG66\_07810 | MET8; precorrin-2 dehydrogenase / sirohydrochlorin ferrochelatase [EC:1.3.1.76 4.99.1.4] |
| K02361 | KSG66\_14835 | entC; isochorismate synthase [EC:5.4.4.2] |
| K02363 | KSG66\_14830 | entE, dhbE, vibE, mxcE; 2,3-dihydroxybenzoate-AMP ligase [EC:6.3.2.14 2.7.7.58] |
| K02437 | KSG66\_15270 | gcvH, GCSH; glycine cleavage system H protein |
| K02446 | KSG66\_17515 | glpX; fructose-1,6-bisphosphatase II [EC:3.1.3.11] |
| K02492 | KSG66\_12920 | hemA; glutamyl-tRNA reductase [EC:1.2.1.70] |
| K02495 | KSG66\_04940 | hemN, hemZ; oxygen-independent coproporphyrinogen III oxidase [EC:1.3.98.3] |
| K02495 | KSG66\_12080 | hemN, hemZ; oxygen-independent coproporphyrinogen III oxidase [EC:1.3.98.3] |
| K02500 | KSG66\_16425 | hisF; imidazole glycerol-phosphate synthase subunit HisF [EC:4.3.2.10] |
| K02501 | KSG66\_16435 | hisH; imidazole glycerol-phosphate synthase subunit HisH [EC:4.3.2.10] |
| K02502 | KSG66\_16455 | hisZ; ATP phosphoribosyltransferase regulatory subunit |
| K02548 | KSG66\_18190 | menA; 1,4-dihydroxy-2-naphthoate octaprenyltransferase [EC:2.5.1.74 2.5.1.-] |
| K02549 | KSG66\_14180 | menC; O-succinylbenzoate synthase [EC:4.2.1.113] |
| K02551 | KSG66\_14200 | menD; 2-succinyl-5-enolpyruvyl-6-hydroxy-3-cyclohexene-1-carboxylate synthase [EC:2.2.1.9] |
| K03183 | KSG66\_10620 | ubiE; demethylmenaquinone methyltransferase / 2-methoxy-6-polyprenyl-1,4-benzoquinol methylase [EC:2.1.1.163 2.1.1.201] |
| K03186 | KSG66\_01890 | ubiX, bsdB, PAD1; flavin prenyltransferase [EC:2.5.1.129] |
| K03526 | KSG66\_11865 | gcpE, ispG; (E)-4-hydroxy-3-methylbut-2-enyl-diphosphate synthase [EC:1.17.7.1 1.17.7.3] |
| K03527 | KSG66\_11910 | ispH, lytB; 4-hydroxy-3-methylbut-2-en-1-yl diphosphate reductase [EC:1.17.7.4] |
| K03621 | KSG66\_07940 | plsX; phosphate acyltransferase [EC:2.3.1.274] |
| K03781 | KSG66\_04470 | katE, CAT, catB, srpA; catalase [EC:1.11.1.6] |
| K03781 | KSG66\_18315 | katE, CAT, catB, srpA; catalase [EC:1.11.1.6] |
| K03781 | KSG66\_18465 | katE, CAT, catB, srpA; catalase [EC:1.11.1.6] |
| K03783 | KSG66\_10970 | punA, PNP; purine-nucleoside phosphorylase [EC:2.4.2.1] |
| K03784 | KSG66\_09785 | deoD; purine-nucleoside phosphorylase [EC:2.4.2.1] |
| K03785 | KSG66\_03920 | aroD; 3-dehydroquinate dehydratase I [EC:4.2.1.10] |
| K03794 | KSG66\_07805 | sirB; sirohydrochlorin ferrochelatase [EC:4.99.1.4] |
| K03809 | KSG66\_04580 | wrbA; NAD(P)H dehydrogenase (quinone) [EC:1.6.5.2] |
| K03816 | KSG66\_10290 | xpt; xanthine phosphoribosyltransferase [EC:2.4.2.22] |
| K03823 | KSG66\_17250 | pat; phosphinothricin acetyltransferase [EC:2.3.1.183] |
| K04041 | KSG66\_18855 | fbp3; fructose-1,6-bisphosphatase III [EC:3.1.3.11] |
| K04486 | KSG66\_13595 | E3.1.3.15B; histidinol-phosphatase (PHP family) [EC:3.1.3.15] |
| K04517 | KSG66\_10550 | tyrA2; prephenate dehydrogenase [EC:1.3.1.12] |
| K04518 | KSG66\_12780 | pheA2; prephenate dehydratase [EC:4.2.1.51] |
| K05375 | KSG66\_14815 | mbtH, nocI; MbtH protein |
| K05822 | KSG66\_07070 | dapH, dapD; tetrahydrodipicolinate N-acetyltransferase [EC:2.3.1.89] |
| K05823 | KSG66\_07075 | dapL; N-acetyldiaminopimelate deacetylase [EC:3.5.1.47] |
| K05825 | KSG66\_16095 | LYSN; 2-aminoadipate transaminase [EC:2.6.1.-] |
| K05887 | KSG66\_03915 | ydiB; quinate/shikimate dehydrogenase [EC:1.1.1.282] |
| K06013 | KSG66\_05175 | STE24; STE24 endopeptidase [EC:3.4.24.84] |
| K06208 | KSG66\_10590 | aroH; chorismate mutase [EC:5.4.99.5] |
| K06209 | KSG66\_12785 | pheB; chorismate mutase [EC:5.4.99.5] |
| K07029 | KSG66\_03490 | dagK; diacylglycerol kinase (ATP) [EC:2.7.1.107] |
| K07145 | KSG66\_03595 | isdG, isdI; heme oxygenase (staphylobilin-producing) [EC:1.14.99.48] |
| K08289 | KSG66\_01345 | purT; phosphoribosylglycinamide formyltransferase 2 [EC:2.1.2.2] |
| K08591 | KSG66\_09030 | plsY; acyl phosphate:glycerol-3-phosphate acyltransferase [EC:2.3.1.275] |
| K08680 | KSG66\_14195 | menH; 2-succinyl-6-hydroxy-2,4-cyclohexadiene-1-carboxylate synthase [EC:4.2.99.20] |
| K08693 | KSG66\_03900 | yfkN; 2',3'-cyclic-nucleotide 2'-phosphodiesterase / 3'-nucleotidase / 5'-nucleotidase [EC:3.1.4.16 3.1.3.6 3.1.3.5] |
| K09699 | KSG66\_11335 | DBT, bkdB; 2-oxoisovalerate dehydrogenase E2 component (dihydrolipoyl transacylase) [EC:2.3.1.168] |
| K11175 | KSG66\_03390 | purN; phosphoribosylglycinamide formyltransferase 1 [EC:2.1.2.2] |
| K11358 | KSG66\_04815 | yhdR; aspartate aminotransferase [EC:2.6.1.1] |
| K11473 | KSG66\_13185 | glcF; glycolate oxidase iron-sulfur subunit |
| K11752 | KSG66\_10880 | ribD; diaminohydroxyphosphoribosylaminopyrimidine deaminase / 5-amino-6-(5-phosphoribosylamino)uracil reductase [EC:3.5.4.26 1.1.1.193] |
| K11753 | KSG66\_08335 | ribF; riboflavin kinase / FMN adenylyltransferase [EC:2.7.1.26 2.7.7.2] |
| K11755 | KSG66\_16420 | hisIE; phosphoribosyl-ATP pyrophosphohydrolase / phosphoribosyl-AMP cyclohydrolase [EC:3.6.1.31 3.5.4.19] |
| K13037 | KSG66\_17810 | bacD; L-alanine-L-anticapsin ligase [EC:6.3.2.49] |
| K13542 | KSG66\_01750 | cobA-hemD; uroporphyrinogen III methyltransferase / synthase [EC:2.1.1.107 4.2.1.75] |
| K13789 | KSG66\_11490 | GGPS; geranylgeranyl diphosphate synthase, type II [EC:2.5.1.1 2.5.1.10 2.5.1.29] |
| K13853 | KSG66\_13760 | aroG, aroA; 3-deoxy-7-phosphoheptulonate synthase / chorismate mutase [EC:2.5.1.54 5.4.99.5] |
| K13953 | KSG66\_08815 | adhP; alcohol dehydrogenase, propanol-preferring [EC:1.1.1.1] |
| K14155 | KSG66\_14570 | patB, malY; cysteine-S-conjugate beta-lyase [EC:4.4.1.13] |
| K14652 | KSG66\_10870 | ribBA; 3,4-dihydroxy 2-butanone 4-phosphate synthase / GTP cyclohydrolase II [EC:4.1.99.12 3.5.4.25] |
| K15633 | KSG66\_15975 | gpmI; 2,3-bisphosphoglycerate-independent phosphoglycerate mutase [EC:5.4.2.12] |
| K15652 | KSG66\_01700 | asbF; 3-dehydroshikimate dehydratase [EC:4.2.1.118] |
| K16150 | KSG66\_19105 | K16150; glycogen synthase [EC:2.4.1.11] |
| K17103 | KSG66\_01350 | CHO1, pssA; CDP-diacylglycerol---serine O-phosphatidyltransferase [EC:2.7.8.8] |
| K17217 | KSG66\_12470 | mccB; cystathionine gamma-lyase / homocysteine desulfhydrase [EC:4.4.1.1 4.4.1.2] |
| K19222 | KSG66\_14680 | menI, DHNAT; 1,4-dihydroxy-2-naphthoyl-CoA hydrolase [EC:3.1.2.28] |
| K19546 | KSG66\_17825 | bacA; prephenate decarboxylase [EC:4.1.1.100] |
| K19547 | KSG66\_17820 | bacB; 3-[(4R)-4-hydroxycyclohexa-1,5-dien-1-yl]-2-oxopropanoate isomerase [EC:5.3.3.19] |
| K19548 | KSG66\_17815 | bacC; dihydroanticapsin dehydrogenase [EC:1.1.1.385] |
| K19549 | KSG66\_17800 | bacF; bacilysin biosynthesis transaminase BacF [EC:2.6.1.-] |
| K19550 | KSG66\_17795 | bacG; bacilysin biosynthesis oxidoreductase BacG [EC:1.3.1.-] |
| K21064 | KSG66\_02130 | ycsE, yitU, ywtE; 5-amino-6-(5-phospho-D-ribitylamino)uracil phosphatase [EC:3.1.3.104] |
| K21064 | KSG66\_05505 | ycsE, yitU, ywtE; 5-amino-6-(5-phospho-D-ribitylamino)uracil phosphatase [EC:3.1.3.104] |
| K21064 | KSG66\_16885 | ycsE, yitU, ywtE; 5-amino-6-(5-phospho-D-ribitylamino)uracil phosphatase [EC:3.1.3.104] |
| K23264 | KSG66\_03365 |  |
| K23265 | KSG66\_03370 |  |
| K23269 | KSG66\_03375 |  |
| K23989 | KSG66\_16845 |  |
| K24042 | KSG66\_05445 |  |

| 3 | Microbial metabolism in diverse environments | ko01120 || K00010 | KSG66\_18720 | iolG; myo-inositol 2-dehydrogenase / D-chiro-inositol 1-dehydrogenase [EC:1.1.1.18 1.1.1.369] |
| K00016 | KSG66\_01650 | LDH, ldh; L-lactate dehydrogenase [EC:1.1.1.27] |
| K00024 | KSG66\_13385 | mdh; malate dehydrogenase [EC:1.1.1.37] |
| K00031 | KSG66\_13390 | IDH1, IDH2, icd; isocitrate dehydrogenase [EC:1.1.1.42] |
| K00033 | KSG66\_11260 | PGD, gnd, gntZ; 6-phosphogluconate dehydrogenase [EC:1.1.1.44 1.1.1.343] |
| K00034 | KSG66\_01550 | gdh; glucose 1-dehydrogenase [EC:1.1.1.47] |
| K00034 | KSG66\_02080 | gdh; glucose 1-dehydrogenase [EC:1.1.1.47] |
| K00036 | KSG66\_11255 | G6PD, zwf; glucose-6-phosphate 1-dehydrogenase [EC:1.1.1.49 1.1.1.363] |
| K00058 | KSG66\_09435 | serA, PHGDH; D-3-phosphoglycerate dehydrogenase / 2-oxoglutarate reductase [EC:1.1.1.95 1.1.1.399] |
| K00058 | KSG66\_10775 | serA, PHGDH; D-3-phosphoglycerate dehydrogenase / 2-oxoglutarate reductase [EC:1.1.1.95 1.1.1.399] |
| K00074 | KSG66\_11400 | paaH, hbd, fadB, mmgB; 3-hydroxybutyryl-CoA dehydrogenase [EC:1.1.1.157] |
| K00090 | KSG66\_16350 | ghrB; glyoxylate/hydroxypyruvate/2-ketogluconate reductase [EC:1.1.1.79 1.1.1.81 1.1.1.215] |
| K00104 | KSG66\_13180 | glcD; glycolate oxidase [EC:1.1.3.15] |
| K00121 | KSG66\_01785 | frmA, ADH5, adhC; S-(hydroxymethyl)glutathione dehydrogenase / alcohol dehydrogenase [EC:1.1.1.284 1.1.1.1] |
| K00121 | KSG66\_02865 | frmA, ADH5, adhC; S-(hydroxymethyl)glutathione dehydrogenase / alcohol dehydrogenase [EC:1.1.1.284 1.1.1.1] |
| K00123 | KSG66\_06060 | fdoG, fdhF, fdwA; formate dehydrogenase major subunit [EC:1.17.1.9] |
| K00123 | KSG66\_12455 | fdoG, fdhF, fdwA; formate dehydrogenase major subunit [EC:1.17.1.9] |
| K00128 | KSG66\_09600 | ALDH; aldehyde dehydrogenase (NAD+) [EC:1.2.1.3] |
| K00128 | KSG66\_13620 | ALDH; aldehyde dehydrogenase (NAD+) [EC:1.2.1.3] |
| K00128 | KSG66\_18800 | ALDH; aldehyde dehydrogenase (NAD+) [EC:1.2.1.3] |
| K00133 | KSG66\_08375 | asd; aspartate-semialdehyde dehydrogenase [EC:1.2.1.11] |
| K00134 | KSG66\_13335 | GAPDH, gapA; glyceraldehyde 3-phosphate dehydrogenase [EC:1.2.1.12] |
| K00134 | KSG66\_15990 | GAPDH, gapA; glyceraldehyde 3-phosphate dehydrogenase [EC:1.2.1.12] |
| K00135 | KSG66\_02070 | gabD; succinate-semialdehyde dehydrogenase / glutarate-semialdehyde dehydrogenase [EC:1.2.1.16 1.2.1.79 1.2.1.20] |
| K00161 | KSG66\_07305 | PDHA, pdhA; pyruvate dehydrogenase E1 component alpha subunit [EC:1.2.4.1] |
| K00162 | KSG66\_07310 | PDHB, pdhB; pyruvate dehydrogenase E1 component beta subunit [EC:1.2.4.1] |
| K00164 | KSG66\_09635 | OGDH, sucA; 2-oxoglutarate dehydrogenase E1 component [EC:1.2.4.2] |
| K00215 | KSG66\_10485 | dapB; 4-hydroxy-tetrahydrodipicolinate reductase [EC:1.17.1.8] |
| K00239 | KSG66\_13060 | sdhA, frdA; succinate dehydrogenase / fumarate reductase, flavoprotein subunit [EC:1.3.5.1 1.3.5.4] |
| K00240 | KSG66\_13055 | sdhB, frdB; succinate dehydrogenase / fumarate reductase, iron-sulfur subunit [EC:1.3.5.1 1.3.5.4] |
| K00241 | KSG66\_13065 | sdhC, frdC; succinate dehydrogenase / fumarate reductase, cytochrome b subunit |
| K00260 | KSG66\_10725 | gudB, rocG; glutamate dehydrogenase [EC:1.4.1.2] |
| K00260 | KSG66\_17855 | gudB, rocG; glutamate dehydrogenase [EC:1.4.1.2] |
| K00265 | KSG66\_09385 | gltB; glutamate synthase (NADPH) large chain [EC:1.4.1.13] |
| K00266 | KSG66\_09380 | gltD; glutamate synthase (NADPH) small chain [EC:1.4.1.13] |
| K00362 | KSG66\_01760 | nirB; nitrite reductase (NADH) large subunit [EC:1.7.1.15] |
| K00362 | KSG66\_01770 | nirB; nitrite reductase (NADH) large subunit [EC:1.7.1.15] |
| K00363 | KSG66\_01755 | nirD; nitrite reductase (NADH) small subunit [EC:1.7.1.15] |
| K00370 | KSG66\_17605 | narG, narZ, nxrA; nitrate reductase / nitrite oxidoreductase, alpha subunit [EC:1.7.5.1 1.7.99.-] |
| K00371 | KSG66\_17600 | narH, narY, nxrB; nitrate reductase / nitrite oxidoreductase, beta subunit [EC:1.7.5.1 1.7.99.-] |
| K00372 | KSG66\_01765 | nasA; assimilatory nitrate reductase catalytic subunit [EC:1.7.99.-] |
| K00374 | KSG66\_17590 | narI, narV; nitrate reductase gamma subunit [EC:1.7.5.1 1.7.99.-] |
| K00380 | KSG66\_15640 | cysJ; sulfite reductase (NADPH) flavoprotein alpha-component [EC:1.8.1.2] |
| K00381 | KSG66\_15635 | cysI; sulfite reductase (NADPH) hemoprotein beta-component [EC:1.8.1.2] |
| K00382 | KSG66\_04065 | DLD, lpd, pdhD; dihydrolipoamide dehydrogenase [EC:1.8.1.4] |
| K00382 | KSG66\_07320 | DLD, lpd, pdhD; dihydrolipoamide dehydrogenase [EC:1.8.1.4] |
| K00382 | KSG66\_11350 | DLD, lpd, pdhD; dihydrolipoamide dehydrogenase [EC:1.8.1.4] |
| K00390 | KSG66\_07780 | cysH; phosphoadenosine phosphosulfate reductase [EC:1.8.4.8 1.8.4.10] |
| K00483 | KSG66\_01300 | hpaB; 4-hydroxyphenylacetate 3-monooxygenase [EC:1.14.14.9] |
| K00600 | KSG66\_17420 | glyA, SHMT; glycine hydroxymethyltransferase [EC:2.1.2.1] |
| K00615 | KSG66\_08935 | E2.2.1.1, tktA, tktB; transketolase [EC:2.2.1.1] |
| K00616 | KSG66\_17525 | E2.2.1.2, talA, talB; transaldolase [EC:2.2.1.2] |
| K00625 | KSG66\_17785 | E2.3.1.8, pta; phosphate acetyltransferase [EC:2.3.1.8] |
| K00626 | KSG66\_05195 | E2.3.1.9, atoB; acetyl-CoA C-acetyltransferase [EC:2.3.1.9] |
| K00626 | KSG66\_11405 | E2.3.1.9, atoB; acetyl-CoA C-acetyltransferase [EC:2.3.1.9] |
| K00627 | KSG66\_04060 | DLAT, aceF, pdhC; pyruvate dehydrogenase E2 component (dihydrolipoamide acetyltransferase) [EC:2.3.1.12] |
| K00627 | KSG66\_07315 | DLAT, aceF, pdhC; pyruvate dehydrogenase E2 component (dihydrolipoamide acetyltransferase) [EC:2.3.1.12] |
| K00632 | KSG66\_15285 | fadA, fadI; acetyl-CoA acyltransferase [EC:2.3.1.16] |
| K00640 | KSG66\_00615 | cysE; serine O-acetyltransferase [EC:2.3.1.30] |
| K00658 | KSG66\_09630 | DLST, sucB; 2-oxoglutarate dehydrogenase E2 component (dihydrolipoamide succinyltransferase) [EC:2.3.1.61] |
| K00821 | KSG66\_05550 | argD; acetylornithine/N-succinyldiaminopimelate aminotransferase [EC:2.6.1.11 2.6.1.17] |
| K00831 | KSG66\_05055 | serC, PSAT1; phosphoserine aminotransferase [EC:2.6.1.52] |
| K00845 | KSG66\_11770 | glk; glucokinase [EC:2.7.1.2] |
| K00850 | KSG66\_13440 | pfkA, PFK; 6-phosphofructokinase 1 [EC:2.7.1.11] |
| K00851 | KSG66\_16055 | E2.7.1.12, gntK, idnK; gluconokinase [EC:2.7.1.12] |
| K00860 | KSG66\_07795 | cysC; adenylylsulfate kinase [EC:2.7.1.25] |
| K00865 | KSG66\_00095 | glxK, garK; glycerate 2-kinase [EC:2.7.1.165] |
| K00872 | KSG66\_14990 | thrB1; homoserine kinase [EC:2.7.1.39] |
| K00873 | KSG66\_13435 | PK, pyk; pyruvate kinase [EC:2.7.1.40] |
| K00874 | KSG66\_09060 | kdgK; 2-dehydro-3-deoxygluconokinase [EC:2.7.1.45] |
| K00925 | KSG66\_13520 | ackA; acetate kinase [EC:2.7.2.1] |
| K00927 | KSG66\_15985 | PGK, pgk; phosphoglycerate kinase [EC:2.7.2.3] |
| K00928 | KSG66\_02010 | lysC; aspartate kinase [EC:2.7.2.4] |
| K00928 | KSG66\_08380 | lysC; aspartate kinase [EC:2.7.2.4] |
| K00928 | KSG66\_13075 | lysC; aspartate kinase [EC:2.7.2.4] |
| K00948 | KSG66\_00320 | PRPS, prsA; ribose-phosphate pyrophosphokinase [EC:2.7.6.1] |
| K00958 | KSG66\_07790 | sat, met3; sulfate adenylyltransferase [EC:2.7.7.4] |
| K01034 | KSG66\_09850 | atoD; acetate CoA/acetoacetate CoA-transferase alpha subunit [EC:2.8.3.8 2.8.3.9] |
| K01035 | KSG66\_09845 | atoA; acetate CoA/acetoacetate CoA-transferase beta subunit [EC:2.8.3.8 2.8.3.9] |
| K01101 | KSG66\_15015 | E3.1.3.41; 4-nitrophenyl phosphatase [EC:3.1.3.41] |
| K01428 | KSG66\_17290 | ureC; urease subunit alpha [EC:3.5.1.5] |
| K01429 | KSG66\_17295 | ureB; urease subunit beta [EC:3.5.1.5] |
| K01430 | KSG66\_17300 | ureA; urease subunit gamma [EC:3.5.1.5] |
| K01439 | KSG66\_13865 | dapE; succinyl-diaminopimelate desuccinylase [EC:3.5.1.18] |
| K01466 | KSG66\_15065 | allB; allantoinase [EC:3.5.2.5] |
| K01491 | KSG66\_11505 | folD; methylenetetrahydrofolate dehydrogenase (NADP+) / methenyltetrahydrofolate cyclohydrolase [EC:1.5.1.5 3.5.4.9] |
| K01512 | KSG66\_03805 | acyP; acylphosphatase [EC:3.6.1.7] |
| K01560 | KSG66\_01210 | E3.8.1.2; 2-haloacid dehalogenase [EC:3.8.1.2] |
| K01586 | KSG66\_10910 | lysA; diaminopimelate decarboxylase [EC:4.1.1.20] |
| K01607 | KSG66\_19235 | pcaC; 4-carboxymuconolactone decarboxylase [EC:4.1.1.44] |
| K01610 | KSG66\_14085 | E4.1.1.49, pckA; phosphoenolpyruvate carboxykinase (ATP) [EC:4.1.1.49] |
| K01612 | KSG66\_01895 | bsdC; vanillate/4-hydroxybenzoate decarboxylase subunit C [EC:4.1.1.- 4.1.1.61] |
| K01624 | KSG66\_17530 | FBA, fbaA; fructose-bisphosphate aldolase, class II [EC:4.1.2.13] |
| K01625 | KSG66\_09070 | eda; 2-dehydro-3-deoxyphosphogluconate aldolase / (4S)-4-hydroxy-2-oxoglutarate aldolase [EC:4.1.2.14 4.1.3.42] |
| K01647 | KSG66\_04765 | CS, gltA; citrate synthase [EC:2.3.3.1] |
| K01647 | KSG66\_11390 | CS, gltA; citrate synthase [EC:2.3.3.1] |
| K01679 | KSG66\_15415 | E4.2.1.2B, fumC, FH; fumarate hydratase, class II [EC:4.2.1.2] |
| K01681 | KSG66\_08990 | ACO, acnA; aconitate hydratase [EC:4.2.1.3] |
| K01689 | KSG66\_15970 | ENO, eno; enolase [EC:4.2.1.11] |
| K01698 | KSG66\_12900 | hemB, ALAD; porphobilinogen synthase [EC:4.2.1.24] |
| K01714 | KSG66\_02785 | dapA; 4-hydroxy-tetrahydrodipicolinate synthase [EC:4.3.3.7] |
| K01714 | KSG66\_08385 | dapA; 4-hydroxy-tetrahydrodipicolinate synthase [EC:4.3.3.7] |
| K01719 | KSG66\_06085 | hemD, UROS; uroporphyrinogen-III synthase [EC:4.2.1.75] |
| K01719 | KSG66\_12905 | hemD, UROS; uroporphyrinogen-III synthase [EC:4.2.1.75] |
| K01733 | KSG66\_14995 | thrC; threonine synthase [EC:4.2.3.1] |
| K01734 | KSG66\_10480 | mgsA; methylglyoxal synthase [EC:4.2.3.3] |
| K01738 | KSG66\_00440 | cysK; cysteine synthase [EC:2.5.1.47] |
| K01738 | KSG66\_13860 | cysK; cysteine synthase [EC:2.5.1.47] |
| K01749 | KSG66\_12910 | hemC, HMBS; hydroxymethylbilane synthase [EC:2.5.1.61] |
| K01778 | KSG66\_14955 | dapF; diaminopimelate epimerase [EC:5.1.1.7] |
| K01783 | KSG66\_07890 | rpe, RPE; ribulose-phosphate 3-epimerase [EC:5.1.3.1] |
| K01785 | KSG66\_09320 | galM, GALM; aldose 1-epimerase [EC:5.1.3.3] |
| K01803 | KSG66\_15980 | TPI, tpiA; triosephosphate isomerase (TIM) [EC:5.3.1.1] |
| K01808 | KSG66\_17430 | rpiB; ribose 5-phosphate isomerase B [EC:5.3.1.6] |
| K01810 | KSG66\_14515 | GPI, pgi; glucose-6-phosphate isomerase [EC:5.3.1.9] |
| K01821 | KSG66\_17695 | praC, xylH; 4-oxalocrotonate tautomerase [EC:5.3.2.6] |
| K01835 | KSG66\_04700 | pgm; phosphoglucomutase [EC:5.4.2.2] |
| K01845 | KSG66\_04300 | hemL; glutamate-1-semialdehyde 2,1-aminomutase [EC:5.4.3.8] |
| K01845 | KSG66\_12895 | hemL; glutamate-1-semialdehyde 2,1-aminomutase [EC:5.4.3.8] |
| K01895 | KSG66\_13565 | ACSS, acs; acetyl-CoA synthetase [EC:6.2.1.1] |
| K01895 | KSG66\_13725 | ACSS, acs; acetyl-CoA synthetase [EC:6.2.1.1] |
| K01902 | KSG66\_08045 | sucD; succinyl-CoA synthetase alpha subunit [EC:6.2.1.5] |
| K01903 | KSG66\_08040 | sucC; succinyl-CoA synthetase beta subunit [EC:6.2.1.5] |
| K01915 | KSG66\_08725 | glnA, GLUL; glutamine synthetase [EC:6.3.1.2] |
| K01958 | KSG66\_07440 | PC, pyc; pyruvate carboxylase [EC:6.4.1.1] |
| K01961 | KSG66\_09265 | accC; acetyl-CoA carboxylase, biotin carboxylase subunit [EC:6.4.1.2 6.3.4.14] |
| K01961 | KSG66\_11520 | accC; acetyl-CoA carboxylase, biotin carboxylase subunit [EC:6.4.1.2 6.3.4.14] |
| K01962 | KSG66\_13445 | accA; acetyl-CoA carboxylase carboxyl transferase subunit alpha [EC:6.4.1.2 2.1.3.15] |
| K01963 | KSG66\_13450 | accD; acetyl-CoA carboxylase carboxyl transferase subunit beta [EC:6.4.1.2 2.1.3.15] |
| K01966 | KSG66\_11285 | PCCB, pccB; propionyl-CoA carboxylase beta chain [EC:6.4.1.3 2.1.3.15] |
| K02083 | KSG66\_15100 | allC; allantoate deiminase [EC:3.5.3.9] |
| K02160 | KSG66\_09260 | accB, bccP; acetyl-CoA carboxylase biotin carboxyl carrier protein |
| K02160 | KSG66\_11525 | accB, bccP; acetyl-CoA carboxylase biotin carboxyl carrier protein |
| K02304 | KSG66\_07810 | MET8; precorrin-2 dehydrogenase / sirohydrochlorin ferrochelatase [EC:1.3.1.76 4.99.1.4] |
| K02446 | KSG66\_17515 | glpX; fructose-1,6-bisphosphatase II [EC:3.1.3.11] |
| K02492 | KSG66\_12920 | hemA; glutamyl-tRNA reductase [EC:1.2.1.70] |
| K02770 | KSG66\_07165 | PTS-Fru-EIIC, fruA; PTS system, fructose-specific IIC component |
| K02770 | KSG66\_12390 | PTS-Fru-EIIC, fruA; PTS system, fructose-specific IIC component |
| K03077 | KSG66\_13225 | araD, ulaF, sgaE, sgbE; L-ribulose-5-phosphate 4-epimerase [EC:5.1.3.4] |
| K03186 | KSG66\_01890 | ubiX, bsdB, PAD1; flavin prenyltransferase [EC:2.5.1.129] |
| K03335 | KSG66\_18730 | iolE; inosose dehydratase [EC:4.2.1.44] |
| K03336 | KSG66\_18735 | iolD; 3D-(3,5/4)-trihydroxycyclohexane-1,2-dione acylhydrolase (decyclizing) [EC:3.7.1.22] |
| K03337 | KSG66\_18745 | iolB; 5-deoxy-glucuronate isomerase [EC:5.3.1.30] |
| K03338 | KSG66\_18740 | iolC; 5-dehydro-2-deoxygluconokinase [EC:2.7.1.92] |
| K03339 | KSG66\_18705 | iolJ; 6-phospho-5-dehydro-2-deoxy-D-gluconate aldolase [EC:4.1.2.29] |
| K03897 | KSG66\_04945 | iucD; lysine N6-hydroxylase [EC:1.14.13.59] |
| K04041 | KSG66\_18855 | fbp3; fructose-1,6-bisphosphatase III [EC:3.1.3.11] |
| K05606 | KSG66\_11290 | MCEE, epi; methylmalonyl-CoA/ethylmalonyl-CoA epimerase [EC:5.1.99.1] |
| K06606 | KSG66\_18710 | iolI; 2-keto-myo-inositol isomerase [EC:5.3.99.11] |
| K06881 | KSG66\_13480 | nrnA; bifunctional oligoribonuclease and PAP phosphatase NrnA [EC:3.1.3.7 3.1.13.3] |
| K07104 | KSG66\_04115 | catE; catechol 2,3-dioxygenase [EC:1.13.11.2] |
| K07250 | KSG66\_02065 | gabT; 4-aminobutyrate aminotransferase / (S)-3-amino-2-methylpropionate transaminase / 5-aminovalerate transaminase [EC:2.6.1.19 2.6.1.22 2.6.1.48] |
| K07516 | KSG66\_15290 | fadN; 3-hydroxyacyl-CoA dehydrogenase [EC:1.1.1.35] |
| K08093 | KSG66\_01825 | hxlA; 3-hexulose-6-phosphate synthase [EC:4.1.2.43] |
| K08094 | KSG66\_01820 | hxlB; 6-phospho-3-hexuloisomerase [EC:5.3.1.27] |
| K11473 | KSG66\_13185 | glcF; glycolate oxidase iron-sulfur subunit |
| K13542 | KSG66\_01750 | cobA-hemD; uroporphyrinogen III methyltransferase / synthase [EC:2.1.1.107 4.2.1.75] |
| K13767 | KSG66\_13105 | fadB; enoyl-CoA hydratase [EC:4.2.1.17] |
| K13953 | KSG66\_08815 | adhP; alcohol dehydrogenase, propanol-preferring [EC:1.1.1.1] |
| K14338 | KSG66\_03640 | cypD\_E, CYP102A2\_3; cytochrome P450 / NADPH-cytochrome P450 reductase [EC:1.14.14.1 1.6.2.4] |
| K14338 | KSG66\_12420 | cypD\_E, CYP102A2\_3; cytochrome P450 / NADPH-cytochrome P450 reductase [EC:1.14.14.1 1.6.2.4] |
| K15633 | KSG66\_15975 | gpmI; 2,3-bisphosphoglycerate-independent phosphoglycerate mutase [EC:5.4.2.12] |
| K15866 | KSG66\_04965 | paaG; 2-(1,2-epoxy-1,2-dihydrophenyl)acetyl-CoA isomerase [EC:5.3.3.18] |
| K16044 | KSG66\_15690 | iolW; scyllo-inositol 2-dehydrogenase (NADP+) [EC:1.1.1.371] |
| K21759 | KSG66\_01900 | bsdD; vanillate/4-hydroxybenzoate decarboxylase subunit D [EC:4.1.1.- 4.1.1.61] |
| K22230 | KSG66\_14425 | iolU; scyllo-inositol 2-dehydrogenase (NADP+) [EC:1.1.1.-] |
| K22602 | KSG66\_17025 | hpxW; oxamate amidohydrolase [EC:3.5.1.126] |
| K24042 | KSG66\_05445 |  |

| 4 | Two-component system | ko02020 || K00027 | KSG66\_11010 | ME2, sfcA, maeA; malate dehydrogenase (oxaloacetate-decarboxylating) [EC:1.1.1.38] |
| K00027 | KSG66\_13455 | ME2, sfcA, maeA; malate dehydrogenase (oxaloacetate-decarboxylating) [EC:1.1.1.38] |
| K00027 | KSG66\_13820 | ME2, sfcA, maeA; malate dehydrogenase (oxaloacetate-decarboxylating) [EC:1.1.1.38] |
| K00027 | KSG66\_17495 | ME2, sfcA, maeA; malate dehydrogenase (oxaloacetate-decarboxylating) [EC:1.1.1.38] |
| K00370 | KSG66\_17605 | narG, narZ, nxrA; nitrate reductase / nitrite oxidoreductase, alpha subunit [EC:1.7.5.1 1.7.99.-] |
| K00371 | KSG66\_17600 | narH, narY, nxrB; nitrate reductase / nitrite oxidoreductase, beta subunit [EC:1.7.5.1 1.7.99.-] |
| K00373 | KSG66\_17595 | narJ, narW; nitrate reductase molybdenum cofactor assembly chaperone NarJ/NarW |
| K00374 | KSG66\_17590 | narI, narV; nitrate reductase gamma subunit [EC:1.7.5.1 1.7.99.-] |
| K00425 | KSG66\_14160 | cydA; cytochrome bd ubiquinol oxidase subunit I [EC:7.1.1.7] |
| K00425 | KSG66\_18385 | cydA; cytochrome bd ubiquinol oxidase subunit I [EC:7.1.1.7] |
| K00426 | KSG66\_14165 | cydB; cytochrome bd ubiquinol oxidase subunit II [EC:7.1.1.7] |
| K00426 | KSG66\_18380 | cydB; cytochrome bd ubiquinol oxidase subunit II [EC:7.1.1.7] |
| K00575 | KSG66\_10605 | cheR; chemotaxis protein methyltransferase CheR [EC:2.1.1.80] |
| K00626 | KSG66\_05195 | E2.3.1.9, atoB; acetyl-CoA C-acetyltransferase [EC:2.3.1.9] |
| K00626 | KSG66\_11405 | E2.3.1.9, atoB; acetyl-CoA C-acetyltransferase [EC:2.3.1.9] |
| K00692 | KSG66\_19175 | sacB; levansucrase [EC:2.4.1.10] |
| K01034 | KSG66\_09850 | atoD; acetate CoA/acetoacetate CoA-transferase alpha subunit [EC:2.8.3.8 2.8.3.9] |
| K01035 | KSG66\_09845 | atoA; acetate CoA/acetoacetate CoA-transferase beta subunit [EC:2.8.3.8 2.8.3.9] |
| K01077 | KSG66\_04750 | E3.1.3.1, phoA, phoB; alkaline phosphatase [EC:3.1.3.1] |
| K01104 | KSG66\_03935 | E3.1.3.48; protein-tyrosine phosphatase [EC:3.1.3.48] |
| K01104 | KSG66\_17105 | E3.1.3.48; protein-tyrosine phosphatase [EC:3.1.3.48] |
| K01113 | KSG66\_01470 | phoD; alkaline phosphatase D [EC:3.1.3.1] |
| K01179 | KSG66\_09130 | E3.2.1.4; endoglucanase [EC:3.2.1.4] |
| K01425 | KSG66\_01400 | glsA, GLS; glutaminase [EC:3.5.1.2] |
| K01425 | KSG66\_07425 | glsA, GLS; glutaminase [EC:3.5.1.2] |
| K01791 | KSG66\_16800 | wecB; UDP-N-acetylglucosamine 2-epimerase (non-hydrolysing) [EC:5.1.3.14] |
| K01915 | KSG66\_08725 | glnA, GLUL; glutamine synthetase [EC:6.3.1.2] |
| K02040 | KSG66\_11830 | pstS; phosphate transport system substrate-binding protein |
| K02253 | KSG66\_14695 | comX; competence protein ComX |
| K02259 | KSG66\_07445 | COX15, ctaA; cytochrome c oxidase assembly protein subunit 15 |
| K02313 | KSG66\_00005 | dnaA; chromosomal replication initiator protein |
| K02398 | KSG66\_16685 | flgM; negative regulator of flagellin synthesis FlgM |
| K02405 | KSG66\_08235 | fliA; RNA polymerase sigma factor for flagellar operon FliA |
| K02406 | KSG66\_16650 | fliC; flagellin |
| K02490 | KSG66\_17535 | spo0F; two-component system, response regulator, stage 0 sporulation protein F |
| K02491 | KSG66\_06975 | kinA; two-component system, sporulation sensor kinase A [EC:2.7.13.3] |
| K02556 | KSG66\_06820 | motA; chemotaxis protein MotA |
| K02556 | KSG66\_13750 | motA; chemotaxis protein MotA |
| K03092 | KSG66\_16110 | rpoN; RNA polymerase sigma-54 factor |
| K03367 | KSG66\_18200 | dltA; D-alanine--poly(phosphoribitol) ligase subunit 1 [EC:6.1.1.13] |
| K03406 | KSG66\_06955 | mcp; methyl-accepting chemotaxis protein |
| K03406 | KSG66\_14435 | mcp; methyl-accepting chemotaxis protein |
| K03406 | KSG66\_14440 | mcp; methyl-accepting chemotaxis protein |
| K03406 | KSG66\_14445 | mcp; methyl-accepting chemotaxis protein |
| K03406 | KSG66\_14450 | mcp; methyl-accepting chemotaxis protein |
| K03407 | KSG66\_08215 | cheA; two-component system, chemotaxis family, sensor kinase CheA [EC:2.7.13.3] |
| K03408 | KSG66\_08220 | cheW; purine-binding chemotaxis protein CheW |
| K03412 | KSG66\_08210 | cheB; two-component system, chemotaxis family, protein-glutamate methylesterase/glutaminase [EC:3.1.1.61 3.5.1.44] |
| K03413 | KSG66\_08165 | cheY; two-component system, chemotaxis family, chemotaxis protein CheY |
| K03413 | KSG66\_08960 | cheY; two-component system, chemotaxis family, chemotaxis protein CheY |
| K03415 | KSG66\_06990 | cheV; two-component system, chemotaxis family, chemotaxis protein CheV |
| K03563 | KSG66\_16655 | csrA; carbon storage regulator |
| K03739 | KSG66\_18205 | dltB; membrane protein involved in D-alanine export |
| K03740 | KSG66\_18215 | dltD; D-alanine transfer protein |
| K04751 | KSG66\_17225 | glnB; nitrogen regulatory protein P-II 1 |
| K04771 | KSG66\_06475 | degP, htrA; serine protease Do [EC:3.4.21.107] |
| K04771 | KSG66\_15390 | degP, htrA; serine protease Do [EC:3.4.21.107] |
| K04771 | KSG66\_19020 | degP, htrA; serine protease Do [EC:3.4.21.107] |
| K05338 | KSG66\_13280 | lrgA; holin-like protein |
| K05339 | KSG66\_13275 | lrgB; holin-like protein LrgB |
| K06347 | KSG66\_14580 | kapB; kinase-associated protein B |
| K06375 | KSG66\_12795 | spo0B; stage 0 sporulation protein B (sporulation initiation phosphotransferase) [EC:2.7.-.-] |
| K07260 | KSG66\_09780 | vanY; zinc D-Ala-D-Ala carboxypeptidase [EC:3.4.17.14] |
| K07636 | KSG66\_13375 | phoR; two-component system, OmpR family, phosphate regulon sensor histidine kinase PhoR [EC:2.7.13.3] |
| K07636 | KSG66\_16405 | phoR; two-component system, OmpR family, phosphate regulon sensor histidine kinase PhoR [EC:2.7.13.3] |
| K07650 | KSG66\_15400 | cssS; two-component system, OmpR family, sensor histidine kinase CssS [EC:2.7.13.3] |
| K07651 | KSG66\_10790 | resE; two-component system, OmpR family, sensor histidine kinase ResE [EC:2.7.13.3] |
| K07652 | KSG66\_19040 | vicK; two-component system, OmpR family, sensor histidine kinase VicK [EC:2.7.13.3] |
| K07658 | KSG66\_13380 | phoB1, phoP; two-component system, OmpR family, alkaline phosphatase synthesis response regulator PhoP |
| K07668 | KSG66\_09145 | vicR; two-component system, OmpR family, response regulator VicR |
| K07668 | KSG66\_19045 | vicR; two-component system, OmpR family, response regulator VicR |
| K07680 | KSG66\_06170 | comP; two-component system, NarL family, sensor histidine kinase ComP [EC:2.7.13.3] |
| K07680 | KSG66\_14690 | comP; two-component system, NarL family, sensor histidine kinase ComP [EC:2.7.13.3] |
| K07691 | KSG66\_06175 | comA; two-component system, NarL family, competent response regulator ComA |
| K07691 | KSG66\_14685 | comA; two-component system, NarL family, competent response regulator ComA |
| K07692 | KSG66\_16715 | degU; two-component system, NarL family, response regulator DegU |
| K07693 | KSG66\_18780 | desR; two-component system, NarL family, response regulator DesR |
| K07697 | KSG66\_14575 | kinB; two-component system, sporulation sensor kinase B [EC:2.7.13.3] |
| K07698 | KSG66\_07210 | kinC; two-component system, sporulation sensor kinase C [EC:2.7.13.3] |
| K07699 | KSG66\_11460 | spo0A; two-component system, response regulator, stage 0 sporulation protein A |
| K07704 | KSG66\_13290 | lytS; two-component system, LytTR family, sensor histidine kinase LytS [EC:2.7.13.3] |
| K07705 | KSG66\_13285 | lytT, lytR; two-component system, LytTR family, response regulator LytT |
| K07714 | KSG66\_10110 | atoC; two-component system, NtrC family, response regulator AtoC |
| K07717 | KSG66\_01405 | ycbA, glnK; two-component system, sensor histidine kinase YcbA [EC:2.7.13.3] |
| K07719 | KSG66\_01410 | ycbB, glnL; two-component system, response regulator YcbB |
| K07770 | KSG66\_15395 | cssR; two-component system, OmpR family, response regulator CssR |
| K07775 | KSG66\_10795 | resD; two-component system, OmpR family, response regulator ResD |
| K07777 | KSG66\_16720 | degS; two-component system, NarL family, sensor histidine kinase DegS [EC:2.7.13.3] |
| K07778 | KSG66\_18785 | desK; two-component system, NarL family, sensor histidine kinase DesK [EC:2.7.13.3] |
| K10255 | KSG66\_02655 | FAD6, desA; acyl-lipid omega-6 desaturase (Delta-12 desaturase) [EC:1.14.19.23 1.14.19.45] |
| K10255 | KSG66\_18790 | FAD6, desA; acyl-lipid omega-6 desaturase (Delta-12 desaturase) [EC:1.14.19.23 1.14.19.45] |
| K11103 | KSG66\_02385 | dctA; aerobic C4-dicarboxylate transport protein |
| K11614 | KSG66\_14610 | yufL, malK; two-component system, CitB family, sensor histidine kinase MalK [EC:2.7.13.3] |
| K11615 | KSG66\_14615 | malR; two-component system, CitB family, response regulator MalR |
| K11616 | KSG66\_18390 | maeN; malate:Na+ symporter |
| K11617 | KSG66\_15445 | liaS; two-component system, NarL family, sensor histidine kinase LiaS [EC:2.7.13.3] |
| K11618 | KSG66\_15440 | liaR; two-component system, NarL family, response regulator LiaR |
| K11619 | KSG66\_15465 | liaI; lia operon protein LiaI |
| K11620 | KSG66\_15460 | liaH; lia operon protein LiaH |
| K11621 | KSG66\_15455 | liaG; lia operon protein LiaG |
| K11622 | KSG66\_15450 | liaF; lia operon protein LiaF |
| K11623 | KSG66\_02880 | ydfH; two-component system, NarL family, sensor histidine kinase YdfH [EC:2.7.13.3] |
| K11624 | KSG66\_02885 | ydfI; two-component system, NarL family, response regulator YdfI |
| K11625 | KSG66\_02890 | ydfJ; membrane protein YdfJ |
| K11629 | KSG66\_13985 | bceS; two-component system, OmpR family, bacitracin resistance sensor histidine kinase BceS [EC:2.7.13.3] |
| K11630 | KSG66\_13990 | bceR; two-component system, OmpR family, bacitracin resistance response regulator BceR |
| K11631 | KSG66\_13980 | bceA, vraD; bacitracin transport system ATP-binding protein |
| K11632 | KSG66\_13975 | bceB, vraE; bacitracin transport system permease protein |
| K11633 | KSG66\_18670 | yxdK; two-component system, OmpR family, sensor histidine kinase YxdK [EC:2.7.13.3] |
| K11634 | KSG66\_18675 | yxdJ; two-component system, OmpR family, response regulator YxdJ |
| K11637 | KSG66\_03775 | citS; two-component system, CitB family, sensor histidine kinase CitS [EC:2.7.13.3] |
| K11638 | KSG66\_03780 | K11638, citT; two-component system, CitB family, response regulator CitT |
| K11639 | KSG66\_03790 | citM; Mg2+/citrate complex secondary transporter |
| K11691 | KSG66\_02375 | K11691, dctS; two-component system, CitB family, sensor histidine kinase DctS [EC:2.7.13.3] |
| K11692 | KSG66\_02380 | K11692, dctR; two-component system, CitB family, response regulator DctR |
| K13532 | KSG66\_06805 | kinD; two-component system, sporulation sensor kinase D [EC:2.7.13.3] |
| K13533 | KSG66\_06750 | kinE; two-component system, sporulation sensor kinase E [EC:2.7.13.3] |
| K14188 | KSG66\_18210 | dltC; D-alanine--poly(phosphoribitol) ligase subunit 2 [EC:6.1.1.13] |
| K14205 | KSG66\_04165 | mprF, fmtC; phosphatidylglycerol lysyltransferase [EC:2.3.2.3] |
| K20487 | KSG66\_15825 | nisK, spaK; two-component system, OmpR family, lantibiotic biosynthesis sensor histidine kinase NisK/SpaK [EC:2.7.13.3] |
| K20488 | KSG66\_15830 | nisR, spaR; two-component system, OmpR family, lantibiotic biosynthesis response regulator NisR/SpaR |
| K20490 | KSG66\_15845 | nisF, spaF, cprA, epiF; lantibiotic transport system ATP-binding protein |
| K20491 | KSG66\_15840 | nisE, spaE, cprB, epiE; lantibiotic transport system permease protein |
| K20492 | KSG66\_15835 | nisG, spaG, cprC; lantibiotic transport system permease protein |

| 5 | Biosynthesis of amino acids | ko01230 || K00013 | KSG66\_16445 | hisD; histidinol dehydrogenase [EC:1.1.1.23] |
| K00014 | KSG66\_12170 | aroE; shikimate dehydrogenase [EC:1.1.1.25] |
| K00031 | KSG66\_13390 | IDH1, IDH2, icd; isocitrate dehydrogenase [EC:1.1.1.42] |
| K00052 | KSG66\_12970 | leuB, IMDH; 3-isopropylmalate dehydrogenase [EC:1.1.1.85] |
| K00053 | KSG66\_12980 | ilvC; ketol-acid reductoisomerase [EC:1.1.1.86] |
| K00058 | KSG66\_09435 | serA, PHGDH; D-3-phosphoglycerate dehydrogenase / 2-oxoglutarate reductase [EC:1.1.1.95 1.1.1.399] |
| K00058 | KSG66\_10775 | serA, PHGDH; D-3-phosphoglycerate dehydrogenase / 2-oxoglutarate reductase [EC:1.1.1.95 1.1.1.399] |
| K00133 | KSG66\_08375 | asd; aspartate-semialdehyde dehydrogenase [EC:1.2.1.11] |
| K00134 | KSG66\_13335 | GAPDH, gapA; glyceraldehyde 3-phosphate dehydrogenase [EC:1.2.1.12] |
| K00134 | KSG66\_15990 | GAPDH, gapA; glyceraldehyde 3-phosphate dehydrogenase [EC:1.2.1.12] |
| K00145 | KSG66\_05535 | argC; N-acetyl-gamma-glutamyl-phosphate reductase [EC:1.2.1.38] |
| K00147 | KSG66\_06570 | proA; glutamate-5-semialdehyde dehydrogenase [EC:1.2.1.41] |
| K00215 | KSG66\_10485 | dapB; 4-hydroxy-tetrahydrodipicolinate reductase [EC:1.17.1.8] |
| K00265 | KSG66\_09385 | gltB; glutamate synthase (NADPH) large chain [EC:1.4.1.13] |
| K00266 | KSG66\_09380 | gltD; glutamate synthase (NADPH) small chain [EC:1.4.1.13] |
| K00286 | KSG66\_09400 | proC; pyrroline-5-carboxylate reductase [EC:1.5.1.2] |
| K00286 | KSG66\_11140 | proC; pyrroline-5-carboxylate reductase [EC:1.5.1.2] |
| K00549 | KSG66\_06615 | metE; 5-methyltetrahydropteroyltriglutamate--homocysteine methyltransferase [EC:2.1.1.14] |
| K00549 | KSG66\_18440 | metE; 5-methyltetrahydropteroyltriglutamate--homocysteine methyltransferase [EC:2.1.1.14] |
| K00600 | KSG66\_17420 | glyA, SHMT; glycine hydroxymethyltransferase [EC:2.1.2.1] |
| K00611 | KSG66\_05565 | OTC, argF, argI; ornithine carbamoyltransferase [EC:2.1.3.3] |
| K00615 | KSG66\_08935 | E2.2.1.1, tktA, tktB; transketolase [EC:2.2.1.1] |
| K00616 | KSG66\_17525 | E2.2.1.2, talA, talB; transaldolase [EC:2.2.1.2] |
| K00620 | KSG66\_05540 | argJ; glutamate N-acetyltransferase / amino-acid N-acetyltransferase [EC:2.3.1.35 2.3.1.1] |
| K00640 | KSG66\_00615 | cysE; serine O-acetyltransferase [EC:2.3.1.30] |
| K00651 | KSG66\_10170 | metA; homoserine O-succinyltransferase/O-acetyltransferase [EC:2.3.1.46 2.3.1.31] |
| K00765 | KSG66\_16450 | hisG; ATP phosphoribosyltransferase [EC:2.4.2.17] |
| K00766 | KSG66\_10580 | trpD; anthranilate phosphoribosyltransferase [EC:2.4.2.18] |
| K00789 | KSG66\_14080 | metK; S-adenosylmethionine synthetase [EC:2.5.1.6] |
| K00800 | KSG66\_10545 | aroA; 3-phosphoshikimate 1-carboxyvinyltransferase [EC:2.5.1.19] |
| K00812 | KSG66\_10425 | aspB; aspartate aminotransferase [EC:2.6.1.1] |
| K00817 | KSG66\_10555 | hisC; histidinol-phosphate aminotransferase [EC:2.6.1.9] |
| K00821 | KSG66\_05550 | argD; acetylornithine/N-succinyldiaminopimelate aminotransferase [EC:2.6.1.11 2.6.1.17] |
| K00826 | KSG66\_01380 | E2.6.1.42, ilvE; branched-chain amino acid aminotransferase [EC:2.6.1.42] |
| K00826 | KSG66\_18220 | E2.6.1.42, ilvE; branched-chain amino acid aminotransferase [EC:2.6.1.42] |
| K00831 | KSG66\_05055 | serC, PSAT1; phosphoserine aminotransferase [EC:2.6.1.52] |
| K00841 | KSG66\_06980 | patA; aminotransferase [EC:2.6.1.-] |
| K00850 | KSG66\_13440 | pfkA, PFK; 6-phosphofructokinase 1 [EC:2.7.1.11] |
| K00872 | KSG66\_14990 | thrB1; homoserine kinase [EC:2.7.1.39] |
| K00873 | KSG66\_13435 | PK, pyk; pyruvate kinase [EC:2.7.1.40] |
| K00891 | KSG66\_01710 | E2.7.1.71, aroK, aroL; shikimate kinase [EC:2.7.1.71] |
| K00927 | KSG66\_15985 | PGK, pgk; phosphoglycerate kinase [EC:2.7.2.3] |
| K00928 | KSG66\_02010 | lysC; aspartate kinase [EC:2.7.2.4] |
| K00928 | KSG66\_08380 | lysC; aspartate kinase [EC:2.7.2.4] |
| K00928 | KSG66\_13075 | lysC; aspartate kinase [EC:2.7.2.4] |
| K00930 | KSG66\_05545 | argB; acetylglutamate kinase [EC:2.7.2.8] |
| K00931 | KSG66\_06565 | proB; glutamate 5-kinase [EC:2.7.2.11] |
| K00931 | KSG66\_09395 | proB; glutamate 5-kinase [EC:2.7.2.11] |
| K00948 | KSG66\_00320 | PRPS, prsA; ribose-phosphate pyrophosphokinase [EC:2.7.6.1] |
| K01243 | KSG66\_12480 | mtnN, mtn, pfs; adenosylhomocysteine nucleosidase [EC:3.2.2.9] |
| K01438 | KSG66\_09840 | argE; acetylornithine deacetylase [EC:3.5.1.16] |
| K01439 | KSG66\_13865 | dapE; succinyl-diaminopimelate desuccinylase [EC:3.5.1.18] |
| K01476 | KSG66\_18955 | E3.5.3.1, rocF, arg; arginase [EC:3.5.3.1] |
| K01586 | KSG66\_10910 | lysA; diaminopimelate decarboxylase [EC:4.1.1.20] |
| K01609 | KSG66\_10575 | trpC; indole-3-glycerol phosphate synthase [EC:4.1.1.48] |
| K01624 | KSG66\_17530 | FBA, fbaA; fructose-bisphosphate aldolase, class II [EC:4.1.2.13] |
| K01647 | KSG66\_04765 | CS, gltA; citrate synthase [EC:2.3.3.1] |
| K01647 | KSG66\_11390 | CS, gltA; citrate synthase [EC:2.3.3.1] |
| K01649 | KSG66\_12975 | leuA, IMS; 2-isopropylmalate synthase [EC:2.3.3.13] |
| K01652 | KSG66\_12990 | E2.2.1.6L, ilvB, ilvG, ilvI; acetolactate synthase I/II/III large subunit [EC:2.2.1.6] |
| K01652 | KSG66\_16980 | E2.2.1.6L, ilvB, ilvG, ilvI; acetolactate synthase I/II/III large subunit [EC:2.2.1.6] |
| K01653 | KSG66\_12985 | E2.2.1.6S, ilvH, ilvN; acetolactate synthase I/III small subunit [EC:2.2.1.6] |
| K01657 | KSG66\_10585 | trpE; anthranilate synthase component I [EC:4.1.3.27] |
| K01681 | KSG66\_08990 | ACO, acnA; aconitate hydratase [EC:4.2.1.3] |
| K01687 | KSG66\_10150 | ilvD; dihydroxy-acid dehydratase [EC:4.2.1.9] |
| K01689 | KSG66\_15970 | ENO, eno; enolase [EC:4.2.1.11] |
| K01693 | KSG66\_16440 | hisB; imidazoleglycerol-phosphate dehydratase [EC:4.2.1.19] |
| K01695 | KSG66\_10560 | trpA; tryptophan synthase alpha chain [EC:4.2.1.20] |
| K01696 | KSG66\_10565 | trpB; tryptophan synthase beta chain [EC:4.2.1.20] |
| K01703 | KSG66\_12965 | leuC, IPMI-L; 3-isopropylmalate/(R)-2-methylmalate dehydratase large subunit [EC:4.2.1.33 4.2.1.35] |
| K01704 | KSG66\_12960 | leuD, IPMI-S; 3-isopropylmalate/(R)-2-methylmalate dehydratase small subunit [EC:4.2.1.33 4.2.1.35] |
| K01714 | KSG66\_02785 | dapA; 4-hydroxy-tetrahydrodipicolinate synthase [EC:4.3.3.7] |
| K01714 | KSG66\_08385 | dapA; 4-hydroxy-tetrahydrodipicolinate synthase [EC:4.3.3.7] |
| K01733 | KSG66\_14995 | thrC; threonine synthase [EC:4.2.3.1] |
| K01735 | KSG66\_10595 | aroB; 3-dehydroquinate synthase [EC:4.2.3.4] |
| K01736 | KSG66\_10600 | aroC; chorismate synthase [EC:4.2.3.5] |
| K01738 | KSG66\_00440 | cysK; cysteine synthase [EC:2.5.1.47] |
| K01738 | KSG66\_13860 | cysK; cysteine synthase [EC:2.5.1.47] |
| K01739 | KSG66\_05910 | metB; cystathionine gamma-synthase [EC:2.5.1.48] |
| K01752 | KSG66\_07920 | E4.3.1.17, sdaA, sdaB, tdcG; L-serine dehydratase [EC:4.3.1.17] |
| K01752 | KSG66\_07925 | E4.3.1.17, sdaA, sdaB, tdcG; L-serine dehydratase [EC:4.3.1.17] |
| K01754 | KSG66\_10105 | E4.3.1.19, ilvA, tdcB; threonine dehydratase [EC:4.3.1.19] |
| K01755 | KSG66\_13505 | argH, ASL; argininosuccinate lyase [EC:4.3.2.1] |
| K01760 | KSG66\_05915 | metC; cysteine-S-conjugate beta-lyase [EC:4.4.1.13] |
| K01778 | KSG66\_14955 | dapF; diaminopimelate epimerase [EC:5.1.1.7] |
| K01783 | KSG66\_07890 | rpe, RPE; ribulose-phosphate 3-epimerase [EC:5.1.3.1] |
| K01803 | KSG66\_15980 | TPI, tpiA; triosephosphate isomerase (TIM) [EC:5.3.1.1] |
| K01808 | KSG66\_17430 | rpiB; ribose 5-phosphate isomerase B [EC:5.3.1.6] |
| K01814 | KSG66\_16430 | hisA; phosphoribosylformimino-5-aminoimidazole carboxamide ribotide isomerase [EC:5.3.1.16] |
| K01817 | KSG66\_10570 | trpF; phosphoribosylanthranilate isomerase [EC:5.3.1.24] |
| K01915 | KSG66\_08725 | glnA, GLUL; glutamine synthetase [EC:6.3.1.2] |
| K01940 | KSG66\_13510 | argG, ASS1; argininosuccinate synthase [EC:6.3.4.5] |
| K01953 | KSG66\_05395 | asnB, ASNS; asparagine synthase (glutamine-hydrolysing) [EC:6.3.5.4] |
| K01953 | KSG66\_14075 | asnB, ASNS; asparagine synthase (glutamine-hydrolysing) [EC:6.3.5.4] |
| K01958 | KSG66\_07440 | PC, pyc; pyruvate carboxylase [EC:6.4.1.1] |
| K02500 | KSG66\_16425 | hisF; imidazole glycerol-phosphate synthase subunit HisF [EC:4.3.2.10] |
| K02501 | KSG66\_16435 | hisH; imidazole glycerol-phosphate synthase subunit HisH [EC:4.3.2.10] |
| K02502 | KSG66\_16455 | hisZ; ATP phosphoribosyltransferase regulatory subunit |
| K03785 | KSG66\_03920 | aroD; 3-dehydroquinate dehydratase I [EC:4.2.1.10] |
| K04486 | KSG66\_13595 | E3.1.3.15B; histidinol-phosphatase (PHP family) [EC:3.1.3.15] |
| K04517 | KSG66\_10550 | tyrA2; prephenate dehydrogenase [EC:1.3.1.12] |
| K04518 | KSG66\_12780 | pheA2; prephenate dehydratase [EC:4.2.1.51] |
| K05822 | KSG66\_07070 | dapH, dapD; tetrahydrodipicolinate N-acetyltransferase [EC:2.3.1.89] |
| K05823 | KSG66\_07075 | dapL; N-acetyldiaminopimelate deacetylase [EC:3.5.1.47] |
| K06208 | KSG66\_10590 | aroH; chorismate mutase [EC:5.4.99.5] |
| K06209 | KSG66\_12785 | pheB; chorismate mutase [EC:5.4.99.5] |
| K07173 | KSG66\_14140 | luxS; S-ribosylhomocysteine lyase [EC:4.4.1.21] |
| K08093 | KSG66\_01825 | hxlA; 3-hexulose-6-phosphate synthase [EC:4.1.2.43] |
| K08094 | KSG66\_01820 | hxlB; 6-phospho-3-hexuloisomerase [EC:5.3.1.27] |
| K11358 | KSG66\_04815 | yhdR; aspartate aminotransferase [EC:2.6.1.1] |
| K11755 | KSG66\_16420 | hisIE; phosphoribosyl-ATP pyrophosphohydrolase / phosphoribosyl-AMP cyclohydrolase [EC:3.6.1.31 3.5.4.19] |
| K13853 | KSG66\_13760 | aroG, aroA; 3-deoxy-7-phosphoheptulonate synthase / chorismate mutase [EC:2.5.1.54 5.4.99.5] |
| K14155 | KSG66\_14570 | patB, malY; cysteine-S-conjugate beta-lyase [EC:4.4.1.13] |
| K15633 | KSG66\_15975 | gpmI; 2,3-bisphosphoglycerate-independent phosphoglycerate mutase [EC:5.4.2.12] |
| K17216 | KSG66\_12475 | mccA; cystathionine beta-synthase (O-acetyl-L-serine) [EC:2.5.1.134] |
| K17217 | KSG66\_12470 | mccB; cystathionine gamma-lyase / homocysteine desulfhydrase [EC:4.4.1.1 4.4.1.2] |
| K24042 | KSG66\_05445 |  |

| 6 | ABC transporters | ko02010 || K02000 | KSG66\_01615 | proV; glycine betaine/proline transport system ATP-binding protein [EC:3.6.3.32] |
| K02001 | KSG66\_01620 | proW; glycine betaine/proline transport system permease protein |
| K02002 | KSG66\_01625 | proX; glycine betaine/proline transport system substrate-binding protein |
| K02018 | KSG66\_15590 | modB; molybdate transport system permease protein |
| K02020 | KSG66\_15585 | modA; molybdate transport system substrate-binding protein |
| K02036 | KSG66\_11810 | pstB; phosphate transport system ATP-binding protein [EC:7.3.2.1] |
| K02036 | KSG66\_11815 | pstB; phosphate transport system ATP-binding protein [EC:7.3.2.1] |
| K02037 | KSG66\_11825 | pstC; phosphate transport system permease protein |
| K02038 | KSG66\_11820 | pstA; phosphate transport system permease protein |
| K02040 | KSG66\_11830 | pstS; phosphate transport system substrate-binding protein |
| K02071 | KSG66\_15245 | metN; D-methionine transport system ATP-binding protein |
| K02072 | KSG66\_15240 | metI; D-methionine transport system permease protein |
| K02073 | KSG66\_04605 | metQ; D-methionine transport system substrate-binding protein |
| K02073 | KSG66\_15235 | metQ; D-methionine transport system substrate-binding protein |
| K03523 | KSG66\_05205 | bioY; biotin transport system substrate-specific component |
| K03523 | KSG66\_14855 | bioY; biotin transport system substrate-specific component |
| K05845 | KSG66\_15855 | opuC; osmoprotectant transport system substrate-binding protein |
| K05845 | KSG66\_15880 | opuC; osmoprotectant transport system substrate-binding protein |
| K05846 | KSG66\_15850 | opuBD; osmoprotectant transport system permease protein |
| K05846 | KSG66\_15860 | opuBD; osmoprotectant transport system permease protein |
| K05846 | KSG66\_15875 | opuBD; osmoprotectant transport system permease protein |
| K05846 | KSG66\_15885 | opuBD; osmoprotectant transport system permease protein |
| K05847 | KSG66\_15865 | opuA; osmoprotectant transport system ATP-binding protein |
| K05847 | KSG66\_15890 | opuA; osmoprotectant transport system ATP-binding protein |
| K06726 | KSG66\_16945 | rbsD; D-ribose pyranase [EC:5.4.99.62] |
| K07335 | KSG66\_05460 | bmpA, bmpB, tmpC; basic membrane protein A and related proteins |
| K07335 | KSG66\_14620 | bmpA, bmpB, tmpC; basic membrane protein A and related proteins |
| K09692 | KSG66\_16815 | tagG; teichoic acid transport system permease protein |
| K09693 | KSG66\_16810 | tagH; teichoic acid transport system ATP-binding protein [EC:3.6.3.40] |
| K09811 | KSG66\_16595 | ftsX; cell division transport system permease protein |
| K09812 | KSG66\_16600 | ftsE; cell division transport system ATP-binding protein |
| K09815 | KSG66\_01560 | znuA; zinc transport system substrate-binding protein |
| K09816 | KSG66\_01570 | znuB; zinc transport system permease protein |
| K09817 | KSG66\_01565 | znuC; zinc transport system ATP-binding protein [EC:3.6.3.-] |
| K10009 | KSG66\_01875 | tcyB, yecS; L-cystine transport system permease protein |
| K10010 | KSG66\_01870 | tcyC, yecC; L-cystine transport system ATP-binding protein [EC:7.4.2.1] |
| K10039 | KSG66\_12565 | peb1A, glnH; aspartate/glutamate/glutamine transport system substrate-binding protein |
| K10040 | KSG66\_12570 | peb1B, glnP, glnM; aspartate/glutamate/glutamine transport system permease protein |
| K10040 | KSG66\_12575 | peb1B, glnP, glnM; aspartate/glutamate/glutamine transport system permease protein |
| K10041 | KSG66\_12560 | peb1C, glnQ; aspartate/glutamate/glutamine transport system ATP-binding protein [EC:7.4.2.1] |
| K10112 | KSG66\_15110 | msmX, msmK, malK, sugC, ggtA, msiK; multiple sugar transport system ATP-binding protein |
| K10112 | KSG66\_18400 | msmX, msmK, malK, sugC, ggtA, msiK; multiple sugar transport system ATP-binding protein |
| K10117 | KSG66\_13920 | msmE; raffinose/stachyose/melibiose transport system substrate-binding protein |
| K10117 | KSG66\_15135 | msmE; raffinose/stachyose/melibiose transport system substrate-binding protein |
| K10118 | KSG66\_13925 | msmF; raffinose/stachyose/melibiose transport system permease protein |
| K10118 | KSG66\_15130 | msmF; raffinose/stachyose/melibiose transport system permease protein |
| K10119 | KSG66\_13930 | msmG; raffinose/stachyose/melibiose transport system permease protein |
| K10119 | KSG66\_15125 | msmG; raffinose/stachyose/melibiose transport system permease protein |
| K10439 | KSG66\_16960 | rbsB; ribose transport system substrate-binding protein |
| K10440 | KSG66\_16955 | rbsC; ribose transport system permease protein |
| K10441 | KSG66\_16950 | rbsA; ribose transport system ATP-binding protein [EC:3.6.3.17] |
| K10823 | KSG66\_05675 | oppF; oligopeptide transport system ATP-binding protein |
| K11050 | KSG66\_03505 | ABC-2.CYL.A, cylA; multidrug/hemolysin transport system ATP-binding protein |
| K11051 | KSG66\_03510 | ABC-2.CYL.P, cylB; multidrug/hemolysin transport system permease protein |
| K11085 | KSG66\_04290 | msbA; ATP-binding cassette, subfamily B, bacterial MsbA [EC:3.6.3.-] |
| K11631 | KSG66\_13980 | bceA, vraD; bacitracin transport system ATP-binding protein |
| K11632 | KSG66\_13975 | bceB, vraE; bacitracin transport system permease protein |
| K15553 | KSG66\_04485 | ssuA; sulfonate transport system substrate-binding protein |
| K15554 | KSG66\_04490 | ssuC; sulfonate transport system permease protein |
| K15555 | KSG66\_04480 | ssuB; sulfonate transport system ATP-binding protein [EC:3.6.3.-] |
| K15580 | KSG66\_05655 | oppA, mppA; oligopeptide transport system substrate-binding protein |
| K15581 | KSG66\_05660 | oppB; oligopeptide transport system permease protein |
| K15582 | KSG66\_05665 | oppC; oligopeptide transport system permease protein |
| K15583 | KSG66\_05670 | oppD; oligopeptide transport system ATP-binding protein |
| K15770 | KSG66\_16315 | cycB, ganO; arabinogalactan oligomer / maltooligosaccharide transport system substrate-binding protein |
| K15771 | KSG66\_16310 | ganP; arabinogalactan oligomer / maltooligosaccharide transport system permease protein |
| K15772 | KSG66\_16305 | ganQ; arabinogalactan oligomer / maltooligosaccharide transport system permease protein |
| K16012 | KSG66\_18370 | cydC; ATP-binding cassette, subfamily C, bacterial CydC |
| K16013 | KSG66\_18375 | cydD; ATP-binding cassette, subfamily C, bacterial CydD |
| K16199 | KSG66\_06505 | dppE; dipeptide transport system substrate-binding protein |
| K16200 | KSG66\_06490 | dppB1; dipeptide transport system permease protein |
| K16201 | KSG66\_06495 | dppC; dipeptide transport system permease protein |
| K16202 | KSG66\_06500 | dppD; dipeptide transport system ATP-binding protein |
| K16785 | KSG66\_00885 | ecfT; energy-coupling factor transport system permease protein |
| K16785 | KSG66\_06630 | ecfT; energy-coupling factor transport system permease protein |
| K16786 | KSG66\_00875 | ecfA1; energy-coupling factor transport system ATP-binding protein [EC:3.6.3.-] |
| K16787 | KSG66\_00880 | ecfA2; energy-coupling factor transport system ATP-binding protein [EC:3.6.3.-] |
| K16918 | KSG66\_13995 | ytrF; acetoin utilization transport system permease protein |
| K16919 | KSG66\_14005 | ytrC\_D; acetoin utilization transport system permease protein |
| K16919 | KSG66\_14010 | ytrC\_D; acetoin utilization transport system permease protein |
| K16919 | KSG66\_14015 | ytrC\_D; acetoin utilization transport system permease protein |
| K16920 | KSG66\_14000 | ytrE; acetoin utilization transport system ATP-binding protein |
| K16921 | KSG66\_14020 | ytrB; acetoin utilization transport system ATP-binding protein |
| K16956 | KSG66\_15735 | tcyJ; L-cystine transport system substrate-binding protein |
| K16957 | KSG66\_15730 | tcyK; L-cystine transport system substrate-binding protein |
| K16958 | KSG66\_15725 | tcyL; L-cystine transport system permease protein |
| K16959 | KSG66\_15720 | tcyM; L-cystine transport system permease protein |
| K16960 | KSG66\_15715 | tcyN; L-cystine transport system ATP-binding protein [EC:7.4.2.1] |
| K17077 | KSG66\_11315 | artQ; arginine/lysine/histidine transport system permease protein |
| K17234 | KSG66\_13210 | araN; arabinosaccharide transport system substrate-binding protein |
| K17235 | KSG66\_13205 | araP; arabinosaccharide transport system permease protein |
| K17236 | KSG66\_13200 | araQ; arabinosaccharide transport system permease protein |
| K18104 | KSG66\_16415 | abcA, bmrA; ATP-binding cassette, subfamily B, bacterial AbcA/BmrA [EC:7.6.2.2] |
| K18231 | KSG66\_02900 | msrA, vmlR; macrolide transport system ATP-binding/permease protein |
| K18889 | KSG66\_04885 | mdlA, smdA; ATP-binding cassette, subfamily B, multidrug efflux pump |
| K18890 | KSG66\_04890 | mdlB, smdB; ATP-binding cassette, subfamily B, multidrug efflux pump |
| K19309 | KSG66\_03985 | bcrA; bacitracin transport system ATP-binding protein |
| K19310 | KSG66\_03990 | bcrB; bacitracin transport system permease protein |
| K20344 | KSG66\_06190 | blpA, lagD; ATP-binding cassette, subfamily C, bacteriocin exporter |
| K20459 | KSG66\_18690 | nukF, mcdF, sboF; lantibiotic transport system ATP-binding protein |
| K20460 | KSG66\_18695 | nukE, mcdE, sboE; lantibiotic transport system permease protein |
| K20461 | KSG66\_18700 | nukG, mcdG, sboG; lantibiotic transport system permease protein |
| K20490 | KSG66\_15845 | nisF, spaF, cprA, epiF; lantibiotic transport system ATP-binding protein |
| K20491 | KSG66\_15840 | nisE, spaE, cprB, epiE; lantibiotic transport system permease protein |
| K20492 | KSG66\_15835 | nisG, spaG, cprC; lantibiotic transport system permease protein |
| K23059 | KSG66\_11320 | artP, artI; arginine/lysine/histidine transporter system substrate-binding protein |
| K23060 | KSG66\_11310 | artR, artM; arginine/lysine/histidine transport system ATP-binding protein [EC:7.4.2.1] |
| K23535 | KSG66\_14630 |  |
| K23536 | KSG66\_14635 |  |
| K23537 | KSG66\_14625 |  |

| 7 | Carbon metabolism | ko01200 || K00024 | KSG66\_13385 | mdh; malate dehydrogenase [EC:1.1.1.37] |
| K00027 | KSG66\_11010 | ME2, sfcA, maeA; malate dehydrogenase (oxaloacetate-decarboxylating) [EC:1.1.1.38] |
| K00027 | KSG66\_13455 | ME2, sfcA, maeA; malate dehydrogenase (oxaloacetate-decarboxylating) [EC:1.1.1.38] |
| K00027 | KSG66\_13820 | ME2, sfcA, maeA; malate dehydrogenase (oxaloacetate-decarboxylating) [EC:1.1.1.38] |
| K00027 | KSG66\_17495 | ME2, sfcA, maeA; malate dehydrogenase (oxaloacetate-decarboxylating) [EC:1.1.1.38] |
| K00031 | KSG66\_13390 | IDH1, IDH2, icd; isocitrate dehydrogenase [EC:1.1.1.42] |
| K00033 | KSG66\_11260 | PGD, gnd, gntZ; 6-phosphogluconate dehydrogenase [EC:1.1.1.44 1.1.1.343] |
| K00034 | KSG66\_01550 | gdh; glucose 1-dehydrogenase [EC:1.1.1.47] |
| K00034 | KSG66\_02080 | gdh; glucose 1-dehydrogenase [EC:1.1.1.47] |
| K00036 | KSG66\_11255 | G6PD, zwf; glucose-6-phosphate 1-dehydrogenase [EC:1.1.1.49 1.1.1.363] |
| K00058 | KSG66\_09435 | serA, PHGDH; D-3-phosphoglycerate dehydrogenase / 2-oxoglutarate reductase [EC:1.1.1.95 1.1.1.399] |
| K00058 | KSG66\_10775 | serA, PHGDH; D-3-phosphoglycerate dehydrogenase / 2-oxoglutarate reductase [EC:1.1.1.95 1.1.1.399] |
| K00074 | KSG66\_11400 | paaH, hbd, fadB, mmgB; 3-hydroxybutyryl-CoA dehydrogenase [EC:1.1.1.157] |
| K00121 | KSG66\_01785 | frmA, ADH5, adhC; S-(hydroxymethyl)glutathione dehydrogenase / alcohol dehydrogenase [EC:1.1.1.284 1.1.1.1] |
| K00121 | KSG66\_02865 | frmA, ADH5, adhC; S-(hydroxymethyl)glutathione dehydrogenase / alcohol dehydrogenase [EC:1.1.1.284 1.1.1.1] |
| K00123 | KSG66\_06060 | fdoG, fdhF, fdwA; formate dehydrogenase major subunit [EC:1.17.1.9] |
| K00123 | KSG66\_12455 | fdoG, fdhF, fdwA; formate dehydrogenase major subunit [EC:1.17.1.9] |
| K00134 | KSG66\_13335 | GAPDH, gapA; glyceraldehyde 3-phosphate dehydrogenase [EC:1.2.1.12] |
| K00134 | KSG66\_15990 | GAPDH, gapA; glyceraldehyde 3-phosphate dehydrogenase [EC:1.2.1.12] |
| K00140 | KSG66\_18750 | mmsA, iolA, ALDH6A1; malonate-semialdehyde dehydrogenase (acetylating) / methylmalonate-semialdehyde dehydrogenase [EC:1.2.1.18 1.2.1.27] |
| K00161 | KSG66\_07305 | PDHA, pdhA; pyruvate dehydrogenase E1 component alpha subunit [EC:1.2.4.1] |
| K00162 | KSG66\_07310 | PDHB, pdhB; pyruvate dehydrogenase E1 component beta subunit [EC:1.2.4.1] |
| K00164 | KSG66\_09635 | OGDH, sucA; 2-oxoglutarate dehydrogenase E1 component [EC:1.2.4.2] |
| K00239 | KSG66\_13060 | sdhA, frdA; succinate dehydrogenase / fumarate reductase, flavoprotein subunit [EC:1.3.5.1 1.3.5.4] |
| K00240 | KSG66\_13055 | sdhB, frdB; succinate dehydrogenase / fumarate reductase, iron-sulfur subunit [EC:1.3.5.1 1.3.5.4] |
| K00241 | KSG66\_13065 | sdhC, frdC; succinate dehydrogenase / fumarate reductase, cytochrome b subunit |
| K00282 | KSG66\_11625 | gcvPA; glycine dehydrogenase subunit 1 [EC:1.4.4.2] |
| K00283 | KSG66\_11620 | gcvPB; glycine dehydrogenase subunit 2 [EC:1.4.4.2] |
| K00382 | KSG66\_04065 | DLD, lpd, pdhD; dihydrolipoamide dehydrogenase [EC:1.8.1.4] |
| K00382 | KSG66\_07320 | DLD, lpd, pdhD; dihydrolipoamide dehydrogenase [EC:1.8.1.4] |
| K00382 | KSG66\_11350 | DLD, lpd, pdhD; dihydrolipoamide dehydrogenase [EC:1.8.1.4] |
| K00600 | KSG66\_17420 | glyA, SHMT; glycine hydroxymethyltransferase [EC:2.1.2.1] |
| K00605 | KSG66\_11630 | gcvT, AMT; aminomethyltransferase [EC:2.1.2.10] |
| K00615 | KSG66\_08935 | E2.2.1.1, tktA, tktB; transketolase [EC:2.2.1.1] |
| K00616 | KSG66\_17525 | E2.2.1.2, talA, talB; transaldolase [EC:2.2.1.2] |
| K00625 | KSG66\_17785 | E2.3.1.8, pta; phosphate acetyltransferase [EC:2.3.1.8] |
| K00626 | KSG66\_05195 | E2.3.1.9, atoB; acetyl-CoA C-acetyltransferase [EC:2.3.1.9] |
| K00626 | KSG66\_11405 | E2.3.1.9, atoB; acetyl-CoA C-acetyltransferase [EC:2.3.1.9] |
| K00627 | KSG66\_04060 | DLAT, aceF, pdhC; pyruvate dehydrogenase E2 component (dihydrolipoamide acetyltransferase) [EC:2.3.1.12] |
| K00627 | KSG66\_07315 | DLAT, aceF, pdhC; pyruvate dehydrogenase E2 component (dihydrolipoamide acetyltransferase) [EC:2.3.1.12] |
| K00640 | KSG66\_00615 | cysE; serine O-acetyltransferase [EC:2.3.1.30] |
| K00658 | KSG66\_09630 | DLST, sucB; 2-oxoglutarate dehydrogenase E2 component (dihydrolipoamide succinyltransferase) [EC:2.3.1.61] |
| K00831 | KSG66\_05055 | serC, PSAT1; phosphoserine aminotransferase [EC:2.6.1.52] |
| K00845 | KSG66\_11770 | glk; glucokinase [EC:2.7.1.2] |
| K00850 | KSG66\_13440 | pfkA, PFK; 6-phosphofructokinase 1 [EC:2.7.1.11] |
| K00851 | KSG66\_16055 | E2.7.1.12, gntK, idnK; gluconokinase [EC:2.7.1.12] |
| K00873 | KSG66\_13435 | PK, pyk; pyruvate kinase [EC:2.7.1.40] |
| K00874 | KSG66\_09060 | kdgK; 2-dehydro-3-deoxygluconokinase [EC:2.7.1.45] |
| K00925 | KSG66\_13520 | ackA; acetate kinase [EC:2.7.2.1] |
| K00927 | KSG66\_15985 | PGK, pgk; phosphoglycerate kinase [EC:2.7.2.3] |
| K00948 | KSG66\_00320 | PRPS, prsA; ribose-phosphate pyrophosphokinase [EC:2.7.6.1] |
| K01491 | KSG66\_11505 | folD; methylenetetrahydrofolate dehydrogenase (NADP+) / methenyltetrahydrofolate cyclohydrolase [EC:1.5.1.5 3.5.4.9] |
| K01610 | KSG66\_14085 | E4.1.1.49, pckA; phosphoenolpyruvate carboxykinase (ATP) [EC:4.1.1.49] |
| K01624 | KSG66\_17530 | FBA, fbaA; fructose-bisphosphate aldolase, class II [EC:4.1.2.13] |
| K01625 | KSG66\_09070 | eda; 2-dehydro-3-deoxyphosphogluconate aldolase / (4S)-4-hydroxy-2-oxoglutarate aldolase [EC:4.1.2.14 4.1.3.42] |
| K01647 | KSG66\_04765 | CS, gltA; citrate synthase [EC:2.3.3.1] |
| K01647 | KSG66\_11390 | CS, gltA; citrate synthase [EC:2.3.3.1] |
| K01679 | KSG66\_15415 | E4.2.1.2B, fumC, FH; fumarate hydratase, class II [EC:4.2.1.2] |
| K01681 | KSG66\_08990 | ACO, acnA; aconitate hydratase [EC:4.2.1.3] |
| K01689 | KSG66\_15970 | ENO, eno; enolase [EC:4.2.1.11] |
| K01738 | KSG66\_00440 | cysK; cysteine synthase [EC:2.5.1.47] |
| K01738 | KSG66\_13860 | cysK; cysteine synthase [EC:2.5.1.47] |
| K01752 | KSG66\_07920 | E4.3.1.17, sdaA, sdaB, tdcG; L-serine dehydratase [EC:4.3.1.17] |
| K01752 | KSG66\_07925 | E4.3.1.17, sdaA, sdaB, tdcG; L-serine dehydratase [EC:4.3.1.17] |
| K01754 | KSG66\_10105 | E4.3.1.19, ilvA, tdcB; threonine dehydratase [EC:4.3.1.19] |
| K01783 | KSG66\_07890 | rpe, RPE; ribulose-phosphate 3-epimerase [EC:5.1.3.1] |
| K01803 | KSG66\_15980 | TPI, tpiA; triosephosphate isomerase (TIM) [EC:5.3.1.1] |
| K01808 | KSG66\_17430 | rpiB; ribose 5-phosphate isomerase B [EC:5.3.1.6] |
| K01810 | KSG66\_14515 | GPI, pgi; glucose-6-phosphate isomerase [EC:5.3.1.9] |
| K01895 | KSG66\_13565 | ACSS, acs; acetyl-CoA synthetase [EC:6.2.1.1] |
| K01895 | KSG66\_13725 | ACSS, acs; acetyl-CoA synthetase [EC:6.2.1.1] |
| K01902 | KSG66\_08045 | sucD; succinyl-CoA synthetase alpha subunit [EC:6.2.1.5] |
| K01903 | KSG66\_08040 | sucC; succinyl-CoA synthetase beta subunit [EC:6.2.1.5] |
| K01958 | KSG66\_07440 | PC, pyc; pyruvate carboxylase [EC:6.4.1.1] |
| K01961 | KSG66\_09265 | accC; acetyl-CoA carboxylase, biotin carboxylase subunit [EC:6.4.1.2 6.3.4.14] |
| K01961 | KSG66\_11520 | accC; acetyl-CoA carboxylase, biotin carboxylase subunit [EC:6.4.1.2 6.3.4.14] |
| K01962 | KSG66\_13445 | accA; acetyl-CoA carboxylase carboxyl transferase subunit alpha [EC:6.4.1.2 2.1.3.15] |
| K01963 | KSG66\_13450 | accD; acetyl-CoA carboxylase carboxyl transferase subunit beta [EC:6.4.1.2 2.1.3.15] |
| K01966 | KSG66\_11285 | PCCB, pccB; propionyl-CoA carboxylase beta chain [EC:6.4.1.3 2.1.3.15] |
| K02160 | KSG66\_09260 | accB, bccP; acetyl-CoA carboxylase biotin carboxyl carrier protein |
| K02160 | KSG66\_11525 | accB, bccP; acetyl-CoA carboxylase biotin carboxyl carrier protein |
| K02437 | KSG66\_15270 | gcvH, GCSH; glycine cleavage system H protein |
| K02446 | KSG66\_17515 | glpX; fructose-1,6-bisphosphatase II [EC:3.1.3.11] |
| K03781 | KSG66\_04470 | katE, CAT, catB, srpA; catalase [EC:1.11.1.6] |
| K03781 | KSG66\_18315 | katE, CAT, catB, srpA; catalase [EC:1.11.1.6] |
| K03781 | KSG66\_18465 | katE, CAT, catB, srpA; catalase [EC:1.11.1.6] |
| K05606 | KSG66\_11290 | MCEE, epi; methylmalonyl-CoA/ethylmalonyl-CoA epimerase [EC:5.1.99.1] |
| K07516 | KSG66\_15290 | fadN; 3-hydroxyacyl-CoA dehydrogenase [EC:1.1.1.35] |
| K08093 | KSG66\_01825 | hxlA; 3-hexulose-6-phosphate synthase [EC:4.1.2.43] |
| K08094 | KSG66\_01820 | hxlB; 6-phospho-3-hexuloisomerase [EC:5.3.1.27] |
| K15633 | KSG66\_15975 | gpmI; 2,3-bisphosphoglycerate-independent phosphoglycerate mutase [EC:5.4.2.12] |

| 8 | Quorum sensing | ko02024 || K01218 | KSG66\_18310 | gmuG; mannan endo-1,4-beta-mannosidase [EC:3.2.1.78] |
| K01318 | KSG66\_04215 | sspA; glutamyl endopeptidase [EC:3.4.21.19] |
| K01657 | KSG66\_10585 | trpE; anthranilate synthase component I [EC:4.1.3.27] |
| K01728 | KSG66\_03750 | pel; pectate lyase [EC:4.2.2.2] |
| K01897 | KSG66\_02195 | ACSL, fadD; long-chain acyl-CoA synthetase [EC:6.2.1.3] |
| K01897 | KSG66\_05165 | ACSL, fadD; long-chain acyl-CoA synthetase [EC:6.2.1.3] |
| K01897 | KSG66\_05200 | ACSL, fadD; long-chain acyl-CoA synthetase [EC:6.2.1.3] |
| K01897 | KSG66\_13115 | ACSL, fadD; long-chain acyl-CoA synthetase [EC:6.2.1.3] |
| K02031 | KSG66\_04460 | ABC.PE.A; peptide/nickel transport system ATP-binding protein |
| K02031 | KSG66\_05620 | ABC.PE.A; peptide/nickel transport system ATP-binding protein |
| K02032 | KSG66\_05625 | ABC.PE.A1; peptide/nickel transport system ATP-binding protein |
| K02032 | KSG66\_06525 | ABC.PE.A1; peptide/nickel transport system ATP-binding protein |
| K02033 | KSG66\_04450 | ABC.PE.P; peptide/nickel transport system permease protein |
| K02033 | KSG66\_05635 | ABC.PE.P; peptide/nickel transport system permease protein |
| K02034 | KSG66\_04455 | ABC.PE.P1; peptide/nickel transport system permease protein |
| K02034 | KSG66\_05640 | ABC.PE.P1; peptide/nickel transport system permease protein |
| K02035 | KSG66\_04445 | ABC.PE.S; peptide/nickel transport system substrate-binding protein |
| K02035 | KSG66\_05630 | ABC.PE.S; peptide/nickel transport system substrate-binding protein |
| K02250 | KSG66\_05230 | comK; competence protein ComK |
| K02251 | KSG66\_14700 | comQ; competence protein ComQ |
| K02253 | KSG66\_14695 | comX; competence protein ComX |
| K02490 | KSG66\_17535 | spo0F; two-component system, response regulator, stage 0 sporulation protein F |
| K03070 | KSG66\_16620 | secA; preprotein translocase subunit SecA |
| K03073 | KSG66\_00650 | secE; preprotein translocase subunit SecE |
| K03075 | KSG66\_15800 | secG; preprotein translocase subunit SecG |
| K03076 | KSG66\_00830 | secY; preprotein translocase subunit SecY |
| K03106 | KSG66\_07985 | SRP54, ffh; signal recognition particle subunit SRP54 [EC:3.6.5.4] |
| K03110 | KSG66\_07970 | ftsY; fused signal recognition particle receptor |
| K03210 | KSG66\_12710 | yajC; preprotein translocase subunit YajC |
| K03217 | KSG66\_11275 | yidC, spoIIIJ, OXA1, ccfA; YidC/Oxa1 family membrane protein insertase |
| K03217 | KSG66\_19405 | yidC, spoIIIJ, OXA1, ccfA; YidC/Oxa1 family membrane protein insertase |
| K03666 | KSG66\_08665 | hfq; host factor-I protein |
| K06352 | KSG66\_06225 | phrA; phosphatase RapA inhibitor |
| K06353 | KSG66\_02000 | phrC; phosphatase RapC regulator |
| K06359 | KSG66\_06220 | rapA, spo0L; response regulator aspartate phosphatase A (stage 0 sporulation protein L) [EC:3.1.-.-] |
| K06359 | KSG66\_09935 | rapA, spo0L; response regulator aspartate phosphatase A (stage 0 sporulation protein L) [EC:3.1.-.-] |
| K06359 | KSG66\_10015 | rapA, spo0L; response regulator aspartate phosphatase A (stage 0 sporulation protein L) [EC:3.1.-.-] |
| K06360 | KSG66\_17315 | rapB; response regulator aspartate phosphatase B [EC:3.1.-.-] |
| K06361 | KSG66\_01995 | rapC; response regulator aspartate phosphatase C [EC:3.1.-.-] |
| K06364 | KSG66\_17655 | rapF; response regulator aspartate phosphatase F [EC:3.1.-.-] |
| K06366 | KSG66\_02300 | rapH; response regulator aspartate phosphatase H [EC:3.1.-.-] |
| K06375 | KSG66\_12795 | spo0B; stage 0 sporulation protein B (sporulation initiation phosphotransferase) [EC:2.7.-.-] |
| K07173 | KSG66\_14140 | luxS; S-ribosylhomocysteine lyase [EC:4.4.1.21] |
| K07680 | KSG66\_06170 | comP; two-component system, NarL family, sensor histidine kinase ComP [EC:2.7.13.3] |
| K07680 | KSG66\_14690 | comP; two-component system, NarL family, sensor histidine kinase ComP [EC:2.7.13.3] |
| K07691 | KSG66\_06175 | comA; two-component system, NarL family, competent response regulator ComA |
| K07691 | KSG66\_14685 | comA; two-component system, NarL family, competent response regulator ComA |
| K07692 | KSG66\_16715 | degU; two-component system, NarL family, response regulator DegU |
| K07699 | KSG66\_11460 | spo0A; two-component system, response regulator, stage 0 sporulation protein A |
| K10823 | KSG66\_05675 | oppF; oligopeptide transport system ATP-binding protein |
| K11216 | KSG66\_09430 | lsrK; autoinducer-2 kinase [EC:2.7.1.189] |
| K11752 | KSG66\_10880 | ribD; diaminohydroxyphosphoribosylaminopyrimidine deaminase / 5-amino-6-(5-phosphoribosylamino)uracil reductase [EC:3.5.4.26 1.1.1.193] |
| K12257 | KSG66\_12685 | secDF; SecD/SecF fusion protein |
| K15580 | KSG66\_05655 | oppA, mppA; oligopeptide transport system substrate-binding protein |
| K15581 | KSG66\_05660 | oppB; oligopeptide transport system permease protein |
| K15582 | KSG66\_05665 | oppC; oligopeptide transport system permease protein |
| K15583 | KSG66\_05670 | oppD; oligopeptide transport system ATP-binding protein |
| K15655 | KSG66\_01840 | srfAB, lchAB; surfactin family lipopeptide synthetase B |
| K15656 | KSG66\_01845 | srfAC, lchAC; surfactin family lipopeptide synthetase C |
| K15657 | KSG66\_01850 | srfATE, srfAD, lchAD; external thioesterase TEII |
| K20344 | KSG66\_06190 | blpA, lagD; ATP-binding cassette, subfamily C, bacteriocin exporter |
| K20487 | KSG66\_15825 | nisK, spaK; two-component system, OmpR family, lantibiotic biosynthesis sensor histidine kinase NisK/SpaK [EC:2.7.13.3] |
| K20488 | KSG66\_15830 | nisR, spaR; two-component system, OmpR family, lantibiotic biosynthesis response regulator NisR/SpaR |
| K20490 | KSG66\_15845 | nisF, spaF, cprA, epiF; lantibiotic transport system ATP-binding protein |
| K20491 | KSG66\_15840 | nisE, spaE, cprB, epiE; lantibiotic transport system permease protein |
| K20492 | KSG66\_15835 | nisG, spaG, cprC; lantibiotic transport system permease protein |

| 9 | Ribosome | ko03010 || K02863 | KSG66\_00665 | RP-L1, MRPL1, rplA; large subunit ribosomal protein L1 |
| K02864 | KSG66\_00670 | RP-L10, MRPL10, rplJ; large subunit ribosomal protein L10 |
| K02867 | KSG66\_00660 | RP-L11, MRPL11, rplK; large subunit ribosomal protein L11 |
| K02871 | KSG66\_00895 | RP-L13, MRPL13, rplM; large subunit ribosomal protein L13 |
| K02874 | KSG66\_00780 | RP-L14, MRPL14, rplN; large subunit ribosomal protein L14 |
| K02876 | KSG66\_00825 | RP-L15, MRPL15, rplO; large subunit ribosomal protein L15 |
| K02878 | KSG66\_00765 | RP-L16, MRPL16, rplP; large subunit ribosomal protein L16 |
| K02879 | KSG66\_00870 | RP-L17, MRPL17, rplQ; large subunit ribosomal protein L17 |
| K02881 | KSG66\_00810 | RP-L18, MRPL18, rplR; large subunit ribosomal protein L18 |
| K02884 | KSG66\_08015 | RP-L19, MRPL19, rplS; large subunit ribosomal protein L19 |
| K02886 | KSG66\_00745 | RP-L2, MRPL2, rplB; large subunit ribosomal protein L2 |
| K02887 | KSG66\_13260 | RP-L20, MRPL20, rplT; large subunit ribosomal protein L20 |
| K02888 | KSG66\_12810 | RP-L21, MRPL21, rplU; large subunit ribosomal protein L21 |
| K02890 | KSG66\_00755 | RP-L22, MRPL22, rplV; large subunit ribosomal protein L22 |
| K02892 | KSG66\_00740 | RP-L23, MRPL23, rplW; large subunit ribosomal protein L23 |
| K02895 | KSG66\_00785 | RP-L24, MRPL24, rplX; large subunit ribosomal protein L24 |
| K02897 | KSG66\_00325 | RP-L25, rplY; large subunit ribosomal protein L25 |
| K02899 | KSG66\_12800 | RP-L27, MRPL27, rpmA; large subunit ribosomal protein L27 |
| K02902 | KSG66\_07905 | RP-L28, MRPL28, rpmB; large subunit ribosomal protein L28 |
| K02904 | KSG66\_00770 | RP-L29, rpmC; large subunit ribosomal protein L29 |
| K02906 | KSG66\_00730 | RP-L3, MRPL3, rplC; large subunit ribosomal protein L3 |
| K02907 | KSG66\_00820 | RP-L30, MRPL30, rpmD; large subunit ribosomal protein L30 |
| K02909 | KSG66\_14155 | RP-L31, rpmE; large subunit ribosomal protein L31 |
| K02909 | KSG66\_17505 | RP-L31, rpmE; large subunit ribosomal protein L31 |
| K02911 | KSG66\_07550 | RP-L32, MRPL32, rpmF; large subunit ribosomal protein L32 |
| K02913 | KSG66\_00645 | RP-L33, MRPL33, rpmG; large subunit ribosomal protein L33 |
| K02913 | KSG66\_11245 | RP-L33, MRPL33, rpmG; large subunit ribosomal protein L33 |
| K02913 | KSG66\_11795 | RP-L33, MRPL33, rpmG; large subunit ribosomal protein L33 |
| K02914 | KSG66\_19415 | RP-L34, MRPL34, rpmH; large subunit ribosomal protein L34 |
| K02916 | KSG66\_13265 | RP-L35, MRPL35, rpmI; large subunit ribosomal protein L35 |
| K02919 | KSG66\_00850 | RP-L36, MRPL36, rpmJ; large subunit ribosomal protein L36 |
| K02926 | KSG66\_00735 | RP-L4, MRPL4, rplD; large subunit ribosomal protein L4 |
| K02931 | KSG66\_00790 | RP-L5, MRPL5, rplE; large subunit ribosomal protein L5 |
| K02933 | KSG66\_00805 | RP-L6, MRPL6, rplF; large subunit ribosomal protein L6 |
| K02935 | KSG66\_00675 | RP-L7, MRPL12, rplL; large subunit ribosomal protein L7/L12 |
| K02939 | KSG66\_19135 | RP-L9, MRPL9, rplI; large subunit ribosomal protein L9 |
| K02945 | KSG66\_10685 | RP-S1, rpsA; small subunit ribosomal protein S1 |
| K02946 | KSG66\_00725 | RP-S10, MRPS10, rpsJ; small subunit ribosomal protein S10 |
| K02948 | KSG66\_00860 | RP-S11, MRPS11, rpsK; small subunit ribosomal protein S11 |
| K02950 | KSG66\_00700 | RP-S12, MRPS12, rpsL; small subunit ribosomal protein S12 |
| K02952 | KSG66\_00855 | RP-S13, rpsM; small subunit ribosomal protein S13 |
| K02954 | KSG66\_00795 | RP-S14, MRPS14, rpsN; small subunit ribosomal protein S14 |
| K02954 | KSG66\_04500 | RP-S14, MRPS14, rpsN; small subunit ribosomal protein S14 |
| K02956 | KSG66\_08340 | RP-S15, MRPS15, rpsO; small subunit ribosomal protein S15 |
| K02959 | KSG66\_07990 | RP-S16, MRPS16, rpsP; small subunit ribosomal protein S16 |
| K02961 | KSG66\_00775 | RP-S17, MRPS17, rpsQ; small subunit ribosomal protein S17 |
| K02963 | KSG66\_19325 | RP-S18, MRPS18, rpsR; small subunit ribosomal protein S18 |
| K02965 | KSG66\_00750 | RP-S19, rpsS; small subunit ribosomal protein S19 |
| K02967 | KSG66\_08245 | RP-S2, MRPS2, rpsB; small subunit ribosomal protein S2 |
| K02968 | KSG66\_12105 | RP-S20, rpsT; small subunit ribosomal protein S20 |
| K02970 | KSG66\_12035 | RP-S21, MRPS21, rpsU; small subunit ribosomal protein S21 |
| K02982 | KSG66\_00760 | RP-S3, rpsC; small subunit ribosomal protein S3 |
| K02986 | KSG66\_13615 | RP-S4, rpsD; small subunit ribosomal protein S4 |
| K02988 | KSG66\_00815 | RP-S5, MRPS5, rpsE; small subunit ribosomal protein S5 |
| K02990 | KSG66\_19335 | RP-S6, MRPS6, rpsF; small subunit ribosomal protein S6 |
| K02992 | KSG66\_00705 | RP-S7, MRPS7, rpsG; small subunit ribosomal protein S7 |
| K02994 | KSG66\_00800 | RP-S8, rpsH; small subunit ribosomal protein S8 |
| K02996 | KSG66\_00900 | RP-S9, MRPS9, rpsI; small subunit ribosomal protein S9 |
| K07590 | KSG66\_00695 | RP-L7A, rplGB; large subunit ribosomal protein L7A |

| 10 | Purine metabolism | ko00230 || K00088 | KSG66\_00070 | IMPDH, guaB; IMP dehydrogenase [EC:1.1.1.205] |
| K00364 | KSG66\_14905 | E1.7.1.7, guaC; GMP reductase [EC:1.7.1.7] |
| K00525 | KSG66\_08685 | E1.17.4.1A, nrdA, nrdE; ribonucleoside-diphosphate reductase alpha chain [EC:1.17.4.1] |
| K00526 | KSG66\_08690 | E1.17.4.1B, nrdB, nrdF; ribonucleoside-diphosphate reductase beta chain [EC:1.17.4.1] |
| K00602 | KSG66\_03395 | purH; phosphoribosylaminoimidazolecarboxamide formyltransferase / IMP cyclohydrolase [EC:2.1.2.3 3.5.4.10] |
| K00759 | KSG66\_12665 | APRT, apt; adenine phosphoribosyltransferase [EC:2.4.2.7] |
| K00760 | KSG66\_00415 | hprT, hpt, HPRT1; hypoxanthine phosphoribosyltransferase [EC:2.4.2.8] |
| K00764 | KSG66\_03380 | purF, PPAT; amidophosphoribosyltransferase [EC:2.4.2.14] |
| K00839 | KSG66\_15095 | pucG; (S)-ureidoglycine---glyoxylate transaminase [EC:2.6.1.112] |
| K00860 | KSG66\_07795 | cysC; adenylylsulfate kinase [EC:2.7.1.25] |
| K00873 | KSG66\_13435 | PK, pyk; pyruvate kinase [EC:2.7.1.40] |
| K00939 | KSG66\_00835 | adk, AK; adenylate kinase [EC:2.7.4.3] |
| K00940 | KSG66\_10610 | ndk, NME; nucleoside-diphosphate kinase [EC:2.7.4.6] |
| K00942 | KSG66\_07835 | E2.7.4.8, gmk; guanylate kinase [EC:2.7.4.8] |
| K00948 | KSG66\_00320 | PRPS, prsA; ribose-phosphate pyrophosphokinase [EC:2.7.6.1] |
| K00958 | KSG66\_07790 | sat, met3; sulfate adenylyltransferase [EC:2.7.7.4] |
| K01081 | KSG66\_04640 | E3.1.3.5; 5'-nucleotidase [EC:3.1.3.5] |
| K01081 | KSG66\_15045 | E3.1.3.5; 5'-nucleotidase [EC:3.1.3.5] |
| K01139 | KSG66\_12660 | spoT; GTP diphosphokinase / guanosine-3',5'-bis(diphosphate) 3'-diphosphatase [EC:2.7.6.5 3.1.7.2] |
| K01428 | KSG66\_17290 | ureC; urease subunit alpha [EC:3.5.1.5] |
| K01429 | KSG66\_17295 | ureB; urease subunit beta [EC:3.5.1.5] |
| K01430 | KSG66\_17300 | ureA; urease subunit gamma [EC:3.5.1.5] |
| K01466 | KSG66\_15065 | allB; allantoinase [EC:3.5.2.5] |
| K01486 | KSG66\_03410 | ade; adenine deaminase [EC:3.5.4.2] |
| K01486 | KSG66\_07225 | ade; adenine deaminase [EC:3.5.4.2] |
| K01515 | KSG66\_11040 | nudF; ADP-ribose pyrophosphatase [EC:3.6.1.13] |
| K01588 | KSG66\_03345 | purE; 5-(carboxyamino)imidazole ribonucleotide mutase [EC:5.4.99.18] |
| K01589 | KSG66\_03350 | purK; 5-(carboxyamino)imidazole ribonucleotide synthase [EC:6.3.4.18] |
| K01756 | KSG66\_03355 | purB, ADSL; adenylosuccinate lyase [EC:4.3.2.2] |
| K01835 | KSG66\_04700 | pgm; phosphoglucomutase [EC:5.4.2.2] |
| K01839 | KSG66\_10975 | deoB; phosphopentomutase [EC:5.4.2.7] |
| K01923 | KSG66\_03360 | purC; phosphoribosylaminoimidazole-succinocarboxamide synthase [EC:6.3.2.6] |
| K01933 | KSG66\_03385 | purM; phosphoribosylformylglycinamidine cyclo-ligase [EC:6.3.3.1] |
| K01939 | KSG66\_19070 | purA, ADSS; adenylosuccinate synthase [EC:6.3.4.4] |
| K01945 | KSG66\_03400 | purD; phosphoribosylamine---glycine ligase [EC:6.3.4.13] |
| K01951 | KSG66\_03300 | guaA, GMPS; GMP synthase (glutamine-hydrolysing) [EC:6.3.5.2] |
| K02083 | KSG66\_15100 | allC; allantoate deiminase [EC:3.5.3.9] |
| K02428 | KSG66\_13020 | rdgB; XTP/dITP diphosphohydrolase [EC:3.6.1.66] |
| K03783 | KSG66\_10970 | punA, PNP; purine-nucleoside phosphorylase [EC:2.4.2.1] |
| K03784 | KSG66\_09785 | deoD; purine-nucleoside phosphorylase [EC:2.4.2.1] |
| K03816 | KSG66\_10290 | xpt; xanthine phosphoribosyltransferase [EC:2.4.2.22] |
| K08289 | KSG66\_01345 | purT; phosphoribosylglycinamide formyltransferase 2 [EC:2.1.2.2] |
| K08693 | KSG66\_03900 | yfkN; 2',3'-cyclic-nucleotide 2'-phosphodiesterase / 3'-nucleotidase / 5'-nucleotidase [EC:3.1.4.16 3.1.3.6 3.1.3.5] |
| K11175 | KSG66\_03390 | purN; phosphoribosylglycinamide formyltransferase 1 [EC:2.1.2.2] |
| K15518 | KSG66\_00110 | dgk; deoxyguanosine kinase [EC:2.7.1.113] |
| K15519 | KSG66\_00105 | dck; deoxyadenosine/deoxycytidine kinase [EC:2.7.1.76 2.7.1.74] |
| K22602 | KSG66\_17025 | hpxW; oxamate amidohydrolase [EC:3.5.1.126] |
| K23264 | KSG66\_03365 |  |
| K23265 | KSG66\_03370 |  |
| K23269 | KSG66\_03375 |  |

| 11 | Pyruvate metabolism | ko00620 || K00016 | KSG66\_01650 | LDH, ldh; L-lactate dehydrogenase [EC:1.1.1.27] |
| K00024 | KSG66\_13385 | mdh; malate dehydrogenase [EC:1.1.1.37] |
| K00027 | KSG66\_11010 | ME2, sfcA, maeA; malate dehydrogenase (oxaloacetate-decarboxylating) [EC:1.1.1.38] |
| K00027 | KSG66\_13455 | ME2, sfcA, maeA; malate dehydrogenase (oxaloacetate-decarboxylating) [EC:1.1.1.38] |
| K00027 | KSG66\_13820 | ME2, sfcA, maeA; malate dehydrogenase (oxaloacetate-decarboxylating) [EC:1.1.1.38] |
| K00027 | KSG66\_17495 | ME2, sfcA, maeA; malate dehydrogenase (oxaloacetate-decarboxylating) [EC:1.1.1.38] |
| K00090 | KSG66\_16350 | ghrB; glyoxylate/hydroxypyruvate/2-ketogluconate reductase [EC:1.1.1.79 1.1.1.81 1.1.1.215] |
| K00128 | KSG66\_09600 | ALDH; aldehyde dehydrogenase (NAD+) [EC:1.2.1.3] |
| K00128 | KSG66\_13620 | ALDH; aldehyde dehydrogenase (NAD+) [EC:1.2.1.3] |
| K00128 | KSG66\_18800 | ALDH; aldehyde dehydrogenase (NAD+) [EC:1.2.1.3] |
| K00158 | KSG66\_02275 | E1.2.3.3, poxL; pyruvate oxidase [EC:1.2.3.3] |
| K00161 | KSG66\_07305 | PDHA, pdhA; pyruvate dehydrogenase E1 component alpha subunit [EC:1.2.4.1] |
| K00162 | KSG66\_07310 | PDHB, pdhB; pyruvate dehydrogenase E1 component beta subunit [EC:1.2.4.1] |
| K00382 | KSG66\_04065 | DLD, lpd, pdhD; dihydrolipoamide dehydrogenase [EC:1.8.1.4] |
| K00382 | KSG66\_07320 | DLD, lpd, pdhD; dihydrolipoamide dehydrogenase [EC:1.8.1.4] |
| K00382 | KSG66\_11350 | DLD, lpd, pdhD; dihydrolipoamide dehydrogenase [EC:1.8.1.4] |
| K00625 | KSG66\_17785 | E2.3.1.8, pta; phosphate acetyltransferase [EC:2.3.1.8] |
| K00626 | KSG66\_05195 | E2.3.1.9, atoB; acetyl-CoA C-acetyltransferase [EC:2.3.1.9] |
| K00626 | KSG66\_11405 | E2.3.1.9, atoB; acetyl-CoA C-acetyltransferase [EC:2.3.1.9] |
| K00627 | KSG66\_04060 | DLAT, aceF, pdhC; pyruvate dehydrogenase E2 component (dihydrolipoamide acetyltransferase) [EC:2.3.1.12] |
| K00627 | KSG66\_07315 | DLAT, aceF, pdhC; pyruvate dehydrogenase E2 component (dihydrolipoamide acetyltransferase) [EC:2.3.1.12] |
| K00873 | KSG66\_13435 | PK, pyk; pyruvate kinase [EC:2.7.1.40] |
| K00925 | KSG66\_13520 | ackA; acetate kinase [EC:2.7.2.1] |
| K01069 | KSG66\_08535 | gloB, gloC, HAGH; hydroxyacylglutathione hydrolase [EC:3.1.2.6] |
| K01069 | KSG66\_11745 | gloB, gloC, HAGH; hydroxyacylglutathione hydrolase [EC:3.1.2.6] |
| K01512 | KSG66\_03805 | acyP; acylphosphatase [EC:3.6.1.7] |
| K01610 | KSG66\_14085 | E4.1.1.49, pckA; phosphoenolpyruvate carboxykinase (ATP) [EC:4.1.1.49] |
| K01649 | KSG66\_12975 | leuA, IMS; 2-isopropylmalate synthase [EC:2.3.3.13] |
| K01679 | KSG66\_15415 | E4.2.1.2B, fumC, FH; fumarate hydratase, class II [EC:4.2.1.2] |
| K01759 | KSG66\_03600 | GLO1, gloA; lactoylglutathione lyase [EC:4.4.1.5] |
| K01759 | KSG66\_05955 | GLO1, gloA; lactoylglutathione lyase [EC:4.4.1.5] |
| K01759 | KSG66\_18165 | GLO1, gloA; lactoylglutathione lyase [EC:4.4.1.5] |
| K01759 | KSG66\_19300 | GLO1, gloA; lactoylglutathione lyase [EC:4.4.1.5] |
| K01895 | KSG66\_13565 | ACSS, acs; acetyl-CoA synthetase [EC:6.2.1.1] |
| K01895 | KSG66\_13725 | ACSS, acs; acetyl-CoA synthetase [EC:6.2.1.1] |
| K01958 | KSG66\_07440 | PC, pyc; pyruvate carboxylase [EC:6.4.1.1] |
| K01961 | KSG66\_09265 | accC; acetyl-CoA carboxylase, biotin carboxylase subunit [EC:6.4.1.2 6.3.4.14] |
| K01961 | KSG66\_11520 | accC; acetyl-CoA carboxylase, biotin carboxylase subunit [EC:6.4.1.2 6.3.4.14] |
| K01962 | KSG66\_13445 | accA; acetyl-CoA carboxylase carboxyl transferase subunit alpha [EC:6.4.1.2 2.1.3.15] |
| K01963 | KSG66\_13450 | accD; acetyl-CoA carboxylase carboxyl transferase subunit beta [EC:6.4.1.2 2.1.3.15] |
| K02160 | KSG66\_09260 | accB, bccP; acetyl-CoA carboxylase biotin carboxyl carrier protein |
| K02160 | KSG66\_11525 | accB, bccP; acetyl-CoA carboxylase biotin carboxyl carrier protein |
| K23257 | KSG66\_13350 |  |
| K23257 | KSG66\_15620 |  |

| 12 | Cysteine and methionine metabolism | ko00270 || K00016 | KSG66\_01650 | LDH, ldh; L-lactate dehydrogenase [EC:1.1.1.27] |
| K00024 | KSG66\_13385 | mdh; malate dehydrogenase [EC:1.1.1.37] |
| K00058 | KSG66\_09435 | serA, PHGDH; D-3-phosphoglycerate dehydrogenase / 2-oxoglutarate reductase [EC:1.1.1.95 1.1.1.399] |
| K00058 | KSG66\_10775 | serA, PHGDH; D-3-phosphoglycerate dehydrogenase / 2-oxoglutarate reductase [EC:1.1.1.95 1.1.1.399] |
| K00133 | KSG66\_08375 | asd; aspartate-semialdehyde dehydrogenase [EC:1.2.1.11] |
| K00547 | KSG66\_01390 | mmuM, BHMT2; homocysteine S-methyltransferase [EC:2.1.1.10] |
| K00549 | KSG66\_06615 | metE; 5-methyltetrahydropteroyltriglutamate--homocysteine methyltransferase [EC:2.1.1.14] |
| K00549 | KSG66\_18440 | metE; 5-methyltetrahydropteroyltriglutamate--homocysteine methyltransferase [EC:2.1.1.14] |
| K00558 | KSG66\_18550 | DNMT1, dcm; DNA (cytosine-5)-methyltransferase 1 [EC:2.1.1.37] |
| K00640 | KSG66\_00615 | cysE; serine O-acetyltransferase [EC:2.3.1.30] |
| K00651 | KSG66\_10170 | metA; homoserine O-succinyltransferase/O-acetyltransferase [EC:2.3.1.46 2.3.1.31] |
| K00789 | KSG66\_14080 | metK; S-adenosylmethionine synthetase [EC:2.5.1.6] |
| K00797 | KSG66\_17670 | speE, SRM; spermidine synthase [EC:2.5.1.16] |
| K00812 | KSG66\_10425 | aspB; aspartate aminotransferase [EC:2.6.1.1] |
| K00826 | KSG66\_01380 | E2.6.1.42, ilvE; branched-chain amino acid aminotransferase [EC:2.6.1.42] |
| K00826 | KSG66\_18220 | E2.6.1.42, ilvE; branched-chain amino acid aminotransferase [EC:2.6.1.42] |
| K00831 | KSG66\_05055 | serC, PSAT1; phosphoserine aminotransferase [EC:2.6.1.52] |
| K00899 | KSG66\_06765 | mtnK; 5-methylthioribose kinase [EC:2.7.1.100] |
| K00928 | KSG66\_02010 | lysC; aspartate kinase [EC:2.7.2.4] |
| K00928 | KSG66\_08380 | lysC; aspartate kinase [EC:2.7.2.4] |
| K00928 | KSG66\_13075 | lysC; aspartate kinase [EC:2.7.2.4] |
| K01243 | KSG66\_12480 | mtnN, mtn, pfs; adenosylhomocysteine nucleosidase [EC:3.2.2.9] |
| K01611 | KSG66\_13330 | speD, AMD1; S-adenosylmethionine decarboxylase [EC:4.1.1.50] |
| K01738 | KSG66\_00440 | cysK; cysteine synthase [EC:2.5.1.47] |
| K01738 | KSG66\_13860 | cysK; cysteine synthase [EC:2.5.1.47] |
| K01739 | KSG66\_05910 | metB; cystathionine gamma-synthase [EC:2.5.1.48] |
| K01752 | KSG66\_07920 | E4.3.1.17, sdaA, sdaB, tdcG; L-serine dehydratase [EC:4.3.1.17] |
| K01752 | KSG66\_07925 | E4.3.1.17, sdaA, sdaB, tdcG; L-serine dehydratase [EC:4.3.1.17] |
| K01760 | KSG66\_05915 | metC; cysteine-S-conjugate beta-lyase [EC:4.4.1.13] |
| K07173 | KSG66\_14140 | luxS; S-ribosylhomocysteine lyase [EC:4.4.1.21] |
| K08963 | KSG66\_06760 | mtnA; methylthioribose-1-phosphate isomerase [EC:5.3.1.23] |
| K08964 | KSG66\_06790 | mtnB; methylthioribulose-1-phosphate dehydratase [EC:4.2.1.109] |
| K08965 | KSG66\_06780 | mtnW; 2,3-diketo-5-methylthiopentyl-1-phosphate enolase [EC:5.3.2.5] |
| K08966 | KSG66\_06785 | mtnX; 2-hydroxy-3-keto-5-methylthiopentenyl-1-phosphate phosphatase [EC:3.1.3.87] |
| K08967 | KSG66\_06795 | mtnD, mtnZ, ADI1; 1,2-dihydroxy-3-keto-5-methylthiopentene dioxygenase [EC:1.13.11.53 1.13.11.54] |
| K08968 | KSG66\_13605 | msrC; L-methionine (R)-S-oxide reductase [EC:1.8.4.14] |
| K08969 | KSG66\_06775 | mtnE, mtnV; aminotransferase [EC:2.6.1.-] |
| K11358 | KSG66\_04815 | yhdR; aspartate aminotransferase [EC:2.6.1.1] |
| K14155 | KSG66\_14570 | patB, malY; cysteine-S-conjugate beta-lyase [EC:4.4.1.13] |
| K17216 | KSG66\_12475 | mccA; cystathionine beta-synthase (O-acetyl-L-serine) [EC:2.5.1.134] |
| K17217 | KSG66\_12470 | mccB; cystathionine gamma-lyase / homocysteine desulfhydrase [EC:4.4.1.1 4.4.1.2] |
| K24042 | KSG66\_05445 |  |

| 13 | Amino sugar and nucleotide sugar metabolism | ko00520 || K00012 | KSG66\_16760 | UGDH, ugd; UDPglucose 6-dehydrogenase [EC:1.1.1.22] |
| K00012 | KSG66\_17100 | UGDH, ugd; UDPglucose 6-dehydrogenase [EC:1.1.1.22] |
| K00075 | KSG66\_07625 | murB; UDP-N-acetylmuramate dehydrogenase [EC:1.3.1.98] |
| K00790 | KSG66\_17350 | murA; UDP-N-acetylglucosamine 1-carboxyvinyltransferase [EC:2.5.1.7] |
| K00790 | KSG66\_17520 | murA; UDP-N-acetylglucosamine 1-carboxyvinyltransferase [EC:2.5.1.7] |
| K00820 | KSG66\_01140 | glmS, GFPT; glucosamine---fructose-6-phosphate aminotransferase (isomerizing) [EC:2.6.1.16] |
| K00845 | KSG66\_11770 | glk; glucokinase [EC:2.7.1.2] |
| K00847 | KSG66\_03210 | E2.7.1.4, scrK; fructokinase [EC:2.7.1.4] |
| K00849 | KSG66\_05995 | galK; galactokinase [EC:2.7.1.6] |
| K00849 | KSG66\_18075 | galK; galactokinase [EC:2.7.1.6] |
| K00963 | KSG66\_16805 | UGP2, galU, galF; UTP--glucose-1-phosphate uridylyltransferase [EC:2.7.7.9] |
| K00965 | KSG66\_05985 | galT, GALT; UDPglucose--hexose-1-phosphate uridylyltransferase [EC:2.7.7.12] |
| K00965 | KSG66\_18070 | galT, GALT; UDPglucose--hexose-1-phosphate uridylyltransferase [EC:2.7.7.12] |
| K00978 | KSG66\_03650 | rfbF; glucose-1-phosphate cytidylyltransferase [EC:2.7.7.33] |
| K01198 | KSG66\_08770 | xynB; xylan 1,4-beta-xylosidase [EC:3.2.1.37] |
| K01207 | KSG66\_01060 | nagZ; beta-N-acetylhexosaminidase [EC:3.2.1.52] |
| K01209 | KSG66\_13090 | abfA; alpha-N-arabinofuranosidase [EC:3.2.1.55] |
| K01209 | KSG66\_13195 | abfA; alpha-N-arabinofuranosidase [EC:3.2.1.55] |
| K01233 | KSG66\_15195 | csn; chitosanase [EC:3.2.1.132] |
| K01443 | KSG66\_16485 | nagA, AMDHD2; N-acetylglucosamine-6-phosphate deacetylase [EC:3.5.1.25] |
| K01709 | KSG66\_03655 | rfbG; CDP-glucose 4,6-dehydratase [EC:4.2.1.45] |
| K01784 | KSG66\_05990 | galE, GALE; UDP-glucose 4-epimerase [EC:5.1.3.2] |
| K01784 | KSG66\_18420 | galE, GALE; UDP-glucose 4-epimerase [EC:5.1.3.2] |
| K01791 | KSG66\_16800 | wecB; UDP-N-acetylglucosamine 2-epimerase (non-hydrolysing) [EC:5.1.3.14] |
| K01809 | KSG66\_12385 | manA, MPI; mannose-6-phosphate isomerase [EC:5.3.1.8] |
| K01809 | KSG66\_16850 | manA, MPI; mannose-6-phosphate isomerase [EC:5.3.1.8] |
| K01809 | KSG66\_18305 | manA, MPI; mannose-6-phosphate isomerase [EC:5.3.1.8] |
| K01810 | KSG66\_14515 | GPI, pgi; glucose-6-phosphate isomerase [EC:5.3.1.9] |
| K01835 | KSG66\_04700 | pgm; phosphoglucomutase [EC:5.4.2.2] |
| K02564 | KSG66\_16490 | nagB, GNPDA; glucosamine-6-phosphate deaminase [EC:3.5.99.6] |
| K02777 | KSG66\_10355 | PTS-Glc-EIIA, crr; PTS system, sugar-specific IIA component [EC:2.7.1.-] |
| K02804 | KSG66\_03835 | PTS-Nag-EIIC, nagE; PTS system, N-acetylglucosamine-specific IIC component |
| K03431 | KSG66\_01135 | glmM; phosphoglucosamine mutase [EC:5.4.2.10] |
| K04042 | KSG66\_00315 | glmU; bifunctional UDP-N-acetylglucosamine pyrophosphorylase / Glucosamine-1-phosphate N-acetyltransferase [EC:2.7.7.23 2.3.1.157] |
| K07106 | KSG66\_01075 | murQ; N-acetylmuramic acid 6-phosphate etherase [EC:4.2.1.126] |
| K13010 | KSG66\_15945 | per, rfbE; perosamine synthetase [EC:2.6.1.102] |
| K13015 | KSG66\_15960 | wbpA; UDP-N-acetyl-D-glucosamine dehydrogenase [EC:1.1.1.136] |
| K15894 | KSG66\_10065 | pseB; UDP-N-acetylglucosamine 4,6-dehydratase [EC:4.2.1.115] |
| K15921 | KSG66\_09155 | xynD; arabinoxylan arabinofuranohydrolase [EC:3.2.1.55] |
| K20118 | KSG66\_06930 | PTS-Glc1-EIIC, ptsG, glcA, glcB; PTS system, glucose-specific IIC component |

| 14 | Glycolysis / Gluconeogenesis | ko00010 || K00016 | KSG66\_01650 | LDH, ldh; L-lactate dehydrogenase [EC:1.1.1.27] |
| K00121 | KSG66\_01785 | frmA, ADH5, adhC; S-(hydroxymethyl)glutathione dehydrogenase / alcohol dehydrogenase [EC:1.1.1.284 1.1.1.1] |
| K00121 | KSG66\_02865 | frmA, ADH5, adhC; S-(hydroxymethyl)glutathione dehydrogenase / alcohol dehydrogenase [EC:1.1.1.284 1.1.1.1] |
| K00128 | KSG66\_09600 | ALDH; aldehyde dehydrogenase (NAD+) [EC:1.2.1.3] |
| K00128 | KSG66\_13620 | ALDH; aldehyde dehydrogenase (NAD+) [EC:1.2.1.3] |
| K00128 | KSG66\_18800 | ALDH; aldehyde dehydrogenase (NAD+) [EC:1.2.1.3] |
| K00134 | KSG66\_13335 | GAPDH, gapA; glyceraldehyde 3-phosphate dehydrogenase [EC:1.2.1.12] |
| K00134 | KSG66\_15990 | GAPDH, gapA; glyceraldehyde 3-phosphate dehydrogenase [EC:1.2.1.12] |
| K00161 | KSG66\_07305 | PDHA, pdhA; pyruvate dehydrogenase E1 component alpha subunit [EC:1.2.4.1] |
| K00162 | KSG66\_07310 | PDHB, pdhB; pyruvate dehydrogenase E1 component beta subunit [EC:1.2.4.1] |
| K00382 | KSG66\_04065 | DLD, lpd, pdhD; dihydrolipoamide dehydrogenase [EC:1.8.1.4] |
| K00382 | KSG66\_07320 | DLD, lpd, pdhD; dihydrolipoamide dehydrogenase [EC:1.8.1.4] |
| K00382 | KSG66\_11350 | DLD, lpd, pdhD; dihydrolipoamide dehydrogenase [EC:1.8.1.4] |
| K00627 | KSG66\_04060 | DLAT, aceF, pdhC; pyruvate dehydrogenase E2 component (dihydrolipoamide acetyltransferase) [EC:2.3.1.12] |
| K00627 | KSG66\_07315 | DLAT, aceF, pdhC; pyruvate dehydrogenase E2 component (dihydrolipoamide acetyltransferase) [EC:2.3.1.12] |
| K00845 | KSG66\_11770 | glk; glucokinase [EC:2.7.1.2] |
| K00850 | KSG66\_13440 | pfkA, PFK; 6-phosphofructokinase 1 [EC:2.7.1.11] |
| K00873 | KSG66\_13435 | PK, pyk; pyruvate kinase [EC:2.7.1.40] |
| K00927 | KSG66\_15985 | PGK, pgk; phosphoglycerate kinase [EC:2.7.2.3] |
| K01222 | KSG66\_18225 | E3.2.1.86A, celF; 6-phospho-beta-glucosidase [EC:3.2.1.86] |
| K01223 | KSG66\_01800 | E3.2.1.86B, bglA; 6-phospho-beta-glucosidase [EC:3.2.1.86] |
| K01223 | KSG66\_09755 | E3.2.1.86B, bglA; 6-phospho-beta-glucosidase [EC:3.2.1.86] |
| K01223 | KSG66\_18295 | E3.2.1.86B, bglA; 6-phospho-beta-glucosidase [EC:3.2.1.86] |
| K01610 | KSG66\_14085 | E4.1.1.49, pckA; phosphoenolpyruvate carboxykinase (ATP) [EC:4.1.1.49] |
| K01624 | KSG66\_17530 | FBA, fbaA; fructose-bisphosphate aldolase, class II [EC:4.1.2.13] |
| K01689 | KSG66\_15970 | ENO, eno; enolase [EC:4.2.1.11] |
| K01785 | KSG66\_09320 | galM, GALM; aldose 1-epimerase [EC:5.1.3.3] |
| K01803 | KSG66\_15980 | TPI, tpiA; triosephosphate isomerase (TIM) [EC:5.3.1.1] |
| K01810 | KSG66\_14515 | GPI, pgi; glucose-6-phosphate isomerase [EC:5.3.1.9] |
| K01835 | KSG66\_04700 | pgm; phosphoglucomutase [EC:5.4.2.2] |
| K01895 | KSG66\_13565 | ACSS, acs; acetyl-CoA synthetase [EC:6.2.1.1] |
| K01895 | KSG66\_13725 | ACSS, acs; acetyl-CoA synthetase [EC:6.2.1.1] |
| K02446 | KSG66\_17515 | glpX; fructose-1,6-bisphosphatase II [EC:3.1.3.11] |
| K02777 | KSG66\_10355 | PTS-Glc-EIIA, crr; PTS system, sugar-specific IIA component [EC:2.7.1.-] |
| K04041 | KSG66\_18855 | fbp3; fructose-1,6-bisphosphatase III [EC:3.1.3.11] |
| K13953 | KSG66\_08815 | adhP; alcohol dehydrogenase, propanol-preferring [EC:1.1.1.1] |
| K15633 | KSG66\_15975 | gpmI; 2,3-bisphosphoglycerate-independent phosphoglycerate mutase [EC:5.4.2.12] |
| K20118 | KSG66\_06930 | PTS-Glc1-EIIC, ptsG, glcA, glcB; PTS system, glucose-specific IIC component |

| 15 | Starch and sucrose metabolism | ko00500 || K00692 | KSG66\_19175 | sacB; levansucrase [EC:2.4.1.10] |
| K00845 | KSG66\_11770 | glk; glucokinase [EC:2.7.1.2] |
| K00847 | KSG66\_03210 | E2.7.1.4, scrK; fructokinase [EC:2.7.1.4] |
| K00963 | KSG66\_16805 | UGP2, galU, galF; UTP--glucose-1-phosphate uridylyltransferase [EC:2.7.7.9] |
| K00978 | KSG66\_03650 | rfbF; glucose-1-phosphate cytidylyltransferase [EC:2.7.7.33] |
| K01179 | KSG66\_09130 | E3.2.1.4; endoglucanase [EC:3.2.1.4] |
| K01182 | KSG66\_01555 | IMA, malL; oligo-1,6-glucosidase [EC:3.2.1.10] |
| K01182 | KSG66\_16290 | IMA, malL; oligo-1,6-glucosidase [EC:3.2.1.10] |
| K01187 | KSG66\_14475 | malZ; alpha-glucosidase [EC:3.2.1.20] |
| K01193 | KSG66\_16265 | INV, sacA; beta-fructofuranosidase [EC:3.2.1.26] |
| K01193 | KSG66\_17995 | INV, sacA; beta-fructofuranosidase [EC:3.2.1.26] |
| K01208 | KSG66\_16320 | cd, ma, nplT; cyclomaltodextrinase / maltogenic alpha-amylase / neopullulanase [EC:3.2.1.54 3.2.1.133 3.2.1.135] |
| K01212 | KSG66\_19180 | sacC, levB; levanase [EC:3.2.1.65] |
| K01222 | KSG66\_18225 | E3.2.1.86A, celF; 6-phospho-beta-glucosidase [EC:3.2.1.86] |
| K01223 | KSG66\_01800 | E3.2.1.86B, bglA; 6-phospho-beta-glucosidase [EC:3.2.1.86] |
| K01223 | KSG66\_09755 | E3.2.1.86B, bglA; 6-phospho-beta-glucosidase [EC:3.2.1.86] |
| K01223 | KSG66\_18295 | E3.2.1.86B, bglA; 6-phospho-beta-glucosidase [EC:3.2.1.86] |
| K01226 | KSG66\_03875 | treC; trehalose-6-phosphate hydrolase [EC:3.2.1.93] |
| K01232 | KSG66\_04080 | glvA; maltose-6'-phosphate glucosidase [EC:3.2.1.122] |
| K01810 | KSG66\_14515 | GPI, pgi; glucose-6-phosphate isomerase [EC:5.3.1.9] |
| K01835 | KSG66\_04700 | pgm; phosphoglucomutase [EC:5.4.2.2] |
| K01838 | KSG66\_06020 | pgmB; beta-phosphoglucomutase [EC:5.4.2.6] |
| K01838 | KSG66\_16285 | pgmB; beta-phosphoglucomutase [EC:5.4.2.6] |
| K02750 | KSG66\_04090 | PTS-Glv-EIIC, glvC, malP, aglA; PTS system, alpha-glucoside-specific IIC component |
| K02759 | KSG66\_18230 | PTS-Cel-EIIA, celC, chbA; PTS system, cellobiose-specific IIA component [EC:2.7.1.196 2.7.1.205] |
| K02759 | KSG66\_18285 | PTS-Cel-EIIA, celC, chbA; PTS system, cellobiose-specific IIA component [EC:2.7.1.196 2.7.1.205] |
| K02760 | KSG66\_18240 | PTS-Cel-EIIB, celA, chbB; PTS system, cellobiose-specific IIB component [EC:2.7.1.196 2.7.1.205] |
| K02760 | KSG66\_18280 | PTS-Cel-EIIB, celA, chbB; PTS system, cellobiose-specific IIB component [EC:2.7.1.196 2.7.1.205] |
| K02761 | KSG66\_18170 | PTS-Cel-EIIC, celB, chbC; PTS system, cellobiose-specific IIC component |
| K02761 | KSG66\_18235 | PTS-Cel-EIIC, celB, chbC; PTS system, cellobiose-specific IIC component |
| K02761 | KSG66\_18290 | PTS-Cel-EIIC, celB, chbC; PTS system, cellobiose-specific IIC component |
| K02777 | KSG66\_10355 | PTS-Glc-EIIA, crr; PTS system, sugar-specific IIA component [EC:2.7.1.-] |
| K02810 | KSG66\_01070 | PTS-Scr-EIIC, scrA, sacP, sacX, ptsS; PTS system, sucrose-specific IIC component |
| K02810 | KSG66\_18000 | PTS-Scr-EIIC, scrA, sacP, sacX, ptsS; PTS system, sucrose-specific IIC component |
| K02819 | KSG66\_03870 | PTS-Tre-EIIC, treB; PTS system, trehalose-specific IIC component |
| K16150 | KSG66\_19105 | K16150; glycogen synthase [EC:2.4.1.11] |

| 16 | Alanine, aspartate and glutamate metabolism | ko00250 || K00135 | KSG66\_02070 | gabD; succinate-semialdehyde dehydrogenase / glutarate-semialdehyde dehydrogenase [EC:1.2.1.16 1.2.1.79 1.2.1.20] |
| K00259 | KSG66\_14805 | ald; alanine dehydrogenase [EC:1.4.1.1] |
| K00260 | KSG66\_10725 | gudB, rocG; glutamate dehydrogenase [EC:1.4.1.2] |
| K00260 | KSG66\_17855 | gudB, rocG; glutamate dehydrogenase [EC:1.4.1.2] |
| K00265 | KSG66\_09385 | gltB; glutamate synthase (NADPH) large chain [EC:1.4.1.13] |
| K00266 | KSG66\_09380 | gltD; glutamate synthase (NADPH) small chain [EC:1.4.1.13] |
| K00278 | KSG66\_12765 | nadB; L-aspartate oxidase [EC:1.4.3.16] |
| K00294 | KSG66\_01730 | E1.2.1.88; 1-pyrroline-5-carboxylate dehydrogenase [EC:1.2.1.88] |
| K00294 | KSG66\_17850 | E1.2.1.88; 1-pyrroline-5-carboxylate dehydrogenase [EC:1.2.1.88] |
| K00609 | KSG66\_07740 | pyrB, PYR2; aspartate carbamoyltransferase catalytic subunit [EC:2.1.3.2] |
| K00764 | KSG66\_03380 | purF, PPAT; amidophosphoribosyltransferase [EC:2.4.2.14] |
| K00812 | KSG66\_10425 | aspB; aspartate aminotransferase [EC:2.6.1.1] |
| K00820 | KSG66\_01140 | glmS, GFPT; glucosamine---fructose-6-phosphate aminotransferase (isomerizing) [EC:2.6.1.16] |
| K01424 | KSG66\_01505 | E3.5.1.1, ansA, ansB; L-asparaginase [EC:3.5.1.1] |
| K01424 | KSG66\_11025 | E3.5.1.1, ansA, ansB; L-asparaginase [EC:3.5.1.1] |
| K01425 | KSG66\_01400 | glsA, GLS; glutaminase [EC:3.5.1.2] |
| K01425 | KSG66\_07425 | glsA, GLS; glutaminase [EC:3.5.1.2] |
| K01744 | KSG66\_11020 | aspA; aspartate ammonia-lyase [EC:4.3.1.1] |
| K01755 | KSG66\_13505 | argH, ASL; argininosuccinate lyase [EC:4.3.2.1] |
| K01756 | KSG66\_03355 | purB, ADSL; adenylosuccinate lyase [EC:4.3.2.2] |
| K01779 | KSG66\_02710 | racD; aspartate racemase [EC:5.1.1.13] |
| K01779 | KSG66\_12225 | racD; aspartate racemase [EC:5.1.1.13] |
| K01915 | KSG66\_08725 | glnA, GLUL; glutamine synthetase [EC:6.3.1.2] |
| K01939 | KSG66\_19070 | purA, ADSS; adenylosuccinate synthase [EC:6.3.4.4] |
| K01940 | KSG66\_13510 | argG, ASS1; argininosuccinate synthase [EC:6.3.4.5] |
| K01953 | KSG66\_05395 | asnB, ASNS; asparagine synthase (glutamine-hydrolysing) [EC:6.3.5.4] |
| K01953 | KSG66\_14075 | asnB, ASNS; asparagine synthase (glutamine-hydrolysing) [EC:6.3.5.4] |
| K01955 | KSG66\_05560 | carB, CPA2; carbamoyl-phosphate synthase large subunit [EC:6.3.5.5] |
| K01955 | KSG66\_07755 | carB, CPA2; carbamoyl-phosphate synthase large subunit [EC:6.3.5.5] |
| K01956 | KSG66\_05555 | carA, CPA1; carbamoyl-phosphate synthase small subunit [EC:6.3.5.5] |
| K01956 | KSG66\_07750 | carA, CPA1; carbamoyl-phosphate synthase small subunit [EC:6.3.5.5] |
| K07250 | KSG66\_02065 | gabT; 4-aminobutyrate aminotransferase / (S)-3-amino-2-methylpropionate transaminase / 5-aminovalerate transaminase [EC:2.6.1.19 2.6.1.22 2.6.1.48] |
| K11358 | KSG66\_04815 | yhdR; aspartate aminotransferase [EC:2.6.1.1] |
| K13566 | KSG66\_06770 | NIT2, yafV; omega-amidase [EC:3.5.1.3] |
| K23265 | KSG66\_03370 |  |

| 17 | Propanoate metabolism | ko00640 || K00005 | KSG66\_02915 | gldA; glycerol dehydrogenase [EC:1.1.1.6] |
| K00016 | KSG66\_01650 | LDH, ldh; L-lactate dehydrogenase [EC:1.1.1.27] |
| K00140 | KSG66\_18750 | mmsA, iolA, ALDH6A1; malonate-semialdehyde dehydrogenase (acetylating) / methylmalonate-semialdehyde dehydrogenase [EC:1.2.1.18 1.2.1.27] |
| K00166 | KSG66\_11345 | BCKDHA, bkdA1; 2-oxoisovalerate dehydrogenase E1 component alpha subunit [EC:1.2.4.4] |
| K00167 | KSG66\_11340 | BCKDHB, bkdA2; 2-oxoisovalerate dehydrogenase E1 component beta subunit [EC:1.2.4.4] |
| K00382 | KSG66\_04065 | DLD, lpd, pdhD; dihydrolipoamide dehydrogenase [EC:1.8.1.4] |
| K00382 | KSG66\_07320 | DLD, lpd, pdhD; dihydrolipoamide dehydrogenase [EC:1.8.1.4] |
| K00382 | KSG66\_11350 | DLD, lpd, pdhD; dihydrolipoamide dehydrogenase [EC:1.8.1.4] |
| K00625 | KSG66\_17785 | E2.3.1.8, pta; phosphate acetyltransferase [EC:2.3.1.8] |
| K00626 | KSG66\_05195 | E2.3.1.9, atoB; acetyl-CoA C-acetyltransferase [EC:2.3.1.9] |
| K00626 | KSG66\_11405 | E2.3.1.9, atoB; acetyl-CoA C-acetyltransferase [EC:2.3.1.9] |
| K00925 | KSG66\_13520 | ackA; acetate kinase [EC:2.7.2.1] |
| K01034 | KSG66\_09850 | atoD; acetate CoA/acetoacetate CoA-transferase alpha subunit [EC:2.8.3.8 2.8.3.9] |
| K01035 | KSG66\_09845 | atoA; acetate CoA/acetoacetate CoA-transferase beta subunit [EC:2.8.3.8 2.8.3.9] |
| K01574 | KSG66\_15350 | adc; acetoacetate decarboxylase [EC:4.1.1.4] |
| K01720 | KSG66\_11385 | prpD; 2-methylcitrate dehydratase [EC:4.2.1.79] |
| K01734 | KSG66\_10480 | mgsA; methylglyoxal synthase [EC:4.2.3.3] |
| K01895 | KSG66\_13565 | ACSS, acs; acetyl-CoA synthetase [EC:6.2.1.1] |
| K01895 | KSG66\_13725 | ACSS, acs; acetyl-CoA synthetase [EC:6.2.1.1] |
| K01902 | KSG66\_08045 | sucD; succinyl-CoA synthetase alpha subunit [EC:6.2.1.5] |
| K01903 | KSG66\_08040 | sucC; succinyl-CoA synthetase beta subunit [EC:6.2.1.5] |
| K01961 | KSG66\_09265 | accC; acetyl-CoA carboxylase, biotin carboxylase subunit [EC:6.4.1.2 6.3.4.14] |
| K01961 | KSG66\_11520 | accC; acetyl-CoA carboxylase, biotin carboxylase subunit [EC:6.4.1.2 6.3.4.14] |
| K01962 | KSG66\_13445 | accA; acetyl-CoA carboxylase carboxyl transferase subunit alpha [EC:6.4.1.2 2.1.3.15] |
| K01963 | KSG66\_13450 | accD; acetyl-CoA carboxylase carboxyl transferase subunit beta [EC:6.4.1.2 2.1.3.15] |
| K01966 | KSG66\_11285 | PCCB, pccB; propionyl-CoA carboxylase beta chain [EC:6.4.1.3 2.1.3.15] |
| K02160 | KSG66\_09260 | accB, bccP; acetyl-CoA carboxylase biotin carboxyl carrier protein |
| K02160 | KSG66\_11525 | accB, bccP; acetyl-CoA carboxylase biotin carboxyl carrier protein |
| K03417 | KSG66\_11380 | prpB; methylisocitrate lyase [EC:4.1.3.30] |
| K05606 | KSG66\_11290 | MCEE, epi; methylmalonyl-CoA/ethylmalonyl-CoA epimerase [EC:5.1.99.1] |
| K07250 | KSG66\_02065 | gabT; 4-aminobutyrate aminotransferase / (S)-3-amino-2-methylpropionate transaminase / 5-aminovalerate transaminase [EC:2.6.1.19 2.6.1.22 2.6.1.48] |
| K09699 | KSG66\_11335 | DBT, bkdB; 2-oxoisovalerate dehydrogenase E2 component (dihydrolipoyl transacylase) [EC:2.3.1.168] |
| K19745 | KSG66\_03055 | acuI; acrylyl-CoA reductase (NADPH) [EC:1.3.1.-] |
| K23257 | KSG66\_13350 |  |
| K23257 | KSG66\_15620 |  |

| 18 | Oxidative phosphorylation | ko00190 || K00239 | KSG66\_13060 | sdhA, frdA; succinate dehydrogenase / fumarate reductase, flavoprotein subunit [EC:1.3.5.1 1.3.5.4] |
| K00240 | KSG66\_13055 | sdhB, frdB; succinate dehydrogenase / fumarate reductase, iron-sulfur subunit [EC:1.3.5.1 1.3.5.4] |
| K00241 | KSG66\_13065 | sdhC, frdC; succinate dehydrogenase / fumarate reductase, cytochrome b subunit |
| K00425 | KSG66\_14160 | cydA; cytochrome bd ubiquinol oxidase subunit I [EC:7.1.1.7] |
| K00425 | KSG66\_18385 | cydA; cytochrome bd ubiquinol oxidase subunit I [EC:7.1.1.7] |
| K00426 | KSG66\_14165 | cydB; cytochrome bd ubiquinol oxidase subunit II [EC:7.1.1.7] |
| K00426 | KSG66\_18380 | cydB; cytochrome bd ubiquinol oxidase subunit II [EC:7.1.1.7] |
| K02108 | KSG66\_17405 | ATPF0A, atpB; F-type H+-transporting ATPase subunit a |
| K02109 | KSG66\_17395 | ATPF0B, atpF; F-type H+-transporting ATPase subunit b |
| K02110 | KSG66\_17400 | ATPF0C, atpE; F-type H+-transporting ATPase subunit c |
| K02111 | KSG66\_17385 | ATPF1A, atpA; F-type H+/Na+-transporting ATPase subunit alpha [EC:7.1.2.2 7.2.2.1] |
| K02112 | KSG66\_17375 | ATPF1B, atpD; F-type H+/Na+-transporting ATPase subunit beta [EC:7.1.2.2 7.2.2.1] |
| K02113 | KSG66\_17390 | ATPF1D, atpH; F-type H+-transporting ATPase subunit delta |
| K02114 | KSG66\_17370 | ATPF1E, atpC; F-type H+-transporting ATPase subunit epsilon |
| K02115 | KSG66\_17380 | ATPF1G, atpG; F-type H+-transporting ATPase subunit gamma |
| K02257 | KSG66\_04120 | COX10, ctaB, cyoE; heme o synthase [EC:2.5.1.141] |
| K02257 | KSG66\_07450 | COX10, ctaB, cyoE; heme o synthase [EC:2.5.1.141] |
| K02259 | KSG66\_07445 | COX15, ctaA; cytochrome c oxidase assembly protein subunit 15 |
| K02274 | KSG66\_07460 | coxA, ctaD; cytochrome c oxidase subunit I [EC:1.9.3.1] |
| K02275 | KSG66\_07455 | coxB, ctaC; cytochrome c oxidase subunit II [EC:1.9.3.1] |
| K02276 | KSG66\_07465 | coxC, ctaE; cytochrome c oxidase subunit III [EC:1.9.3.1] |
| K02277 | KSG66\_07470 | coxD, ctaF; cytochrome c oxidase subunit IV [EC:1.9.3.1] |
| K02826 | KSG66\_18060 | qoxA; cytochrome aa3-600 menaquinol oxidase subunit II [EC:7.1.1.5] |
| K02827 | KSG66\_18055 | qoxB; cytochrome aa3-600 menaquinol oxidase subunit I [EC:7.1.1.5] |
| K02828 | KSG66\_18050 | qoxC; cytochrome aa3-600 menaquinol oxidase subunit III [EC:7.1.1.5] |
| K02829 | KSG66\_18045 | qoxD; cytochrome aa3-600 menaquinol oxidase subunit IV [EC:7.1.1.5] |
| K03885 | KSG66\_06115 | ndh; NADH dehydrogenase [EC:1.6.99.3] |
| K03885 | KSG66\_14890 | ndh; NADH dehydrogenase [EC:1.6.99.3] |
| K03885 | KSG66\_14970 | ndh; NADH dehydrogenase [EC:1.6.99.3] |
| K03886 | KSG66\_10525 | MQCRA, qcrA, bfcA, petC; menaquinol-cytochrome c reductase iron-sulfur subunit [EC:1.10.2.-] |
| K03887 | KSG66\_10520 | MQCRB, qcrB, bfcB, petB; menaquinol-cytochrome c reductase cytochrome b subunit |
| K03888 | KSG66\_10515 | MQCRC, qcrC, bfcC, petD; menaquinol-cytochrome c reductase cytochrome b/c subunit |
| K06019 | KSG66\_16470 | ppaX; pyrophosphatase PpaX [EC:3.6.1.1] |
| K15986 | KSG66\_19165 | ppaC; manganese-dependent inorganic pyrophosphatase [EC:3.6.1.1] |

| 19 | Pyrimidine metabolism | ko00240 || K00525 | KSG66\_08685 | E1.17.4.1A, nrdA, nrdE; ribonucleoside-diphosphate reductase alpha chain [EC:1.17.4.1] |
| K00526 | KSG66\_08690 | E1.17.4.1B, nrdB, nrdF; ribonucleoside-diphosphate reductase beta chain [EC:1.17.4.1] |
| K00560 | KSG66\_10130 | thyA, TYMS; thymidylate synthase [EC:2.1.1.45] |
| K00609 | KSG66\_07740 | pyrB, PYR2; aspartate carbamoyltransferase catalytic subunit [EC:2.1.3.2] |
| K00756 | KSG66\_18600 | pdp; pyrimidine-nucleoside phosphorylase [EC:2.4.2.2] |
| K00761 | KSG66\_17415 | upp, UPRT; uracil phosphoribosyltransferase [EC:2.4.2.9] |
| K00762 | KSG66\_07775 | pyrE; orotate phosphoribosyltransferase [EC:2.4.2.10] |
| K00857 | KSG66\_17500 | tdk, TK; thymidine kinase [EC:2.7.1.21] |
| K00876 | KSG66\_12510 | udk, UCK; uridine kinase [EC:2.7.1.48] |
| K00940 | KSG66\_10610 | ndk, NME; nucleoside-diphosphate kinase [EC:2.7.4.6] |
| K00943 | KSG66\_00205 | tmk, DTYMK; dTMP kinase [EC:2.7.4.9] |
| K00945 | KSG66\_10690 | cmk; CMP/dCMP kinase [EC:2.7.4.25] |
| K01081 | KSG66\_04640 | E3.1.3.5; 5'-nucleotidase [EC:3.1.3.5] |
| K01081 | KSG66\_15045 | E3.1.3.5; 5'-nucleotidase [EC:3.1.3.5] |
| K01465 | KSG66\_07745 | URA4, pyrC; dihydroorotase [EC:3.5.2.3] |
| K01489 | KSG66\_11980 | cdd, CDA; cytidine deaminase [EC:3.5.4.5] |
| K01493 | KSG66\_12130 | comEB; dCMP deaminase [EC:3.5.4.12] |
| K01520 | KSG66\_08885 | dut, DUT; dUTP pyrophosphatase [EC:3.6.1.23] |
| K01591 | KSG66\_07770 | pyrF; orotidine-5'-phosphate decarboxylase [EC:4.1.1.23] |
| K01937 | KSG66\_17545 | pyrG, CTPS; CTP synthase [EC:6.3.4.2] |
| K01955 | KSG66\_05560 | carB, CPA2; carbamoyl-phosphate synthase large subunit [EC:6.3.5.5] |
| K01955 | KSG66\_07755 | carB, CPA2; carbamoyl-phosphate synthase large subunit [EC:6.3.5.5] |
| K01956 | KSG66\_05555 | carA, CPA1; carbamoyl-phosphate synthase small subunit [EC:6.3.5.5] |
| K01956 | KSG66\_07750 | carA, CPA1; carbamoyl-phosphate synthase small subunit [EC:6.3.5.5] |
| K02823 | KSG66\_07760 | pyrDII; dihydroorotate dehydrogenase electron transfer subunit |
| K02825 | KSG66\_07730 | pyrR; pyrimidine operon attenuation protein / uracil phosphoribosyltransferase [EC:2.4.2.9] |
| K03783 | KSG66\_10970 | punA, PNP; purine-nucleoside phosphorylase [EC:2.4.2.1] |
| K03784 | KSG66\_09785 | deoD; purine-nucleoside phosphorylase [EC:2.4.2.1] |
| K08693 | KSG66\_03900 | yfkN; 2',3'-cyclic-nucleotide 2'-phosphodiesterase / 3'-nucleotidase / 5'-nucleotidase [EC:3.1.4.16 3.1.3.6 3.1.3.5] |
| K09903 | KSG66\_08255 | pyrH; uridylate kinase [EC:2.7.4.22] |
| K15519 | KSG66\_00105 | dck; deoxyadenosine/deoxycytidine kinase [EC:2.7.1.76 2.7.1.74] |
| K17828 | KSG66\_07765 | pyrDI; dihydroorotate dehydrogenase (NAD+) catalytic subunit [EC:1.3.1.14] |

| 20 | Glycine, serine and threonine metabolism | ko00260 || K00058 | KSG66\_09435 | serA, PHGDH; D-3-phosphoglycerate dehydrogenase / 2-oxoglutarate reductase [EC:1.1.1.95 1.1.1.399] |
| K00058 | KSG66\_10775 | serA, PHGDH; D-3-phosphoglycerate dehydrogenase / 2-oxoglutarate reductase [EC:1.1.1.95 1.1.1.399] |
| K00060 | KSG66\_08495 | tdh; threonine 3-dehydrogenase [EC:1.1.1.103] |
| K00090 | KSG66\_16350 | ghrB; glyoxylate/hydroxypyruvate/2-ketogluconate reductase [EC:1.1.1.79 1.1.1.81 1.1.1.215] |
| K00130 | KSG66\_14370 | betB, gbsA; betaine-aldehyde dehydrogenase [EC:1.2.1.8] |
| K00133 | KSG66\_08375 | asd; aspartate-semialdehyde dehydrogenase [EC:1.2.1.11] |
| K00282 | KSG66\_11625 | gcvPA; glycine dehydrogenase subunit 1 [EC:1.4.4.2] |
| K00283 | KSG66\_11620 | gcvPB; glycine dehydrogenase subunit 2 [EC:1.4.4.2] |
| K00382 | KSG66\_04065 | DLD, lpd, pdhD; dihydrolipoamide dehydrogenase [EC:1.8.1.4] |
| K00382 | KSG66\_07320 | DLD, lpd, pdhD; dihydrolipoamide dehydrogenase [EC:1.8.1.4] |
| K00382 | KSG66\_11350 | DLD, lpd, pdhD; dihydrolipoamide dehydrogenase [EC:1.8.1.4] |
| K00600 | KSG66\_17420 | glyA, SHMT; glycine hydroxymethyltransferase [EC:2.1.2.1] |
| K00605 | KSG66\_11630 | gcvT, AMT; aminomethyltransferase [EC:2.1.2.10] |
| K00639 | KSG66\_08500 | kbl, GCAT; glycine C-acetyltransferase [EC:2.3.1.29] |
| K00831 | KSG66\_05055 | serC, PSAT1; phosphoserine aminotransferase [EC:2.6.1.52] |
| K00865 | KSG66\_00095 | glxK, garK; glycerate 2-kinase [EC:2.7.1.165] |
| K00872 | KSG66\_14990 | thrB1; homoserine kinase [EC:2.7.1.39] |
| K00928 | KSG66\_02010 | lysC; aspartate kinase [EC:2.7.2.4] |
| K00928 | KSG66\_08380 | lysC; aspartate kinase [EC:2.7.2.4] |
| K00928 | KSG66\_13075 | lysC; aspartate kinase [EC:2.7.2.4] |
| K01695 | KSG66\_10560 | trpA; tryptophan synthase alpha chain [EC:4.2.1.20] |
| K01696 | KSG66\_10565 | trpB; tryptophan synthase beta chain [EC:4.2.1.20] |
| K01733 | KSG66\_14995 | thrC; threonine synthase [EC:4.2.3.1] |
| K01752 | KSG66\_07920 | E4.3.1.17, sdaA, sdaB, tdcG; L-serine dehydratase [EC:4.3.1.17] |
| K01752 | KSG66\_07925 | E4.3.1.17, sdaA, sdaB, tdcG; L-serine dehydratase [EC:4.3.1.17] |
| K01753 | KSG66\_11125 | dsdA; D-serine dehydratase [EC:4.3.1.18] |
| K01754 | KSG66\_10105 | E4.3.1.19, ilvA, tdcB; threonine dehydratase [EC:4.3.1.19] |
| K02437 | KSG66\_15270 | gcvH, GCSH; glycine cleavage system H protein |
| K11440 | KSG66\_14365 | gbsB; choline dehydrogenase [EC:1.1.1.1] |
| K15633 | KSG66\_15975 | gpmI; 2,3-bisphosphoglycerate-independent phosphoglycerate mutase [EC:5.4.2.12] |
| K17103 | KSG66\_01350 | CHO1, pssA; CDP-diacylglycerol---serine O-phosphatidyltransferase [EC:2.7.8.8] |
| K17217 | KSG66\_12470 | mccB; cystathionine gamma-lyase / homocysteine desulfhydrase [EC:4.4.1.1 4.4.1.2] |

| 21 | Glyoxylate and dicarboxylate metabolism | ko00630 || K00024 | KSG66\_13385 | mdh; malate dehydrogenase [EC:1.1.1.37] |
| K00090 | KSG66\_16350 | ghrB; glyoxylate/hydroxypyruvate/2-ketogluconate reductase [EC:1.1.1.79 1.1.1.81 1.1.1.215] |
| K00104 | KSG66\_13180 | glcD; glycolate oxidase [EC:1.1.3.15] |
| K00123 | KSG66\_06060 | fdoG, fdhF, fdwA; formate dehydrogenase major subunit [EC:1.17.1.9] |
| K00123 | KSG66\_12455 | fdoG, fdhF, fdwA; formate dehydrogenase major subunit [EC:1.17.1.9] |
| K00282 | KSG66\_11625 | gcvPA; glycine dehydrogenase subunit 1 [EC:1.4.4.2] |
| K00283 | KSG66\_11620 | gcvPB; glycine dehydrogenase subunit 2 [EC:1.4.4.2] |
| K00382 | KSG66\_04065 | DLD, lpd, pdhD; dihydrolipoamide dehydrogenase [EC:1.8.1.4] |
| K00382 | KSG66\_07320 | DLD, lpd, pdhD; dihydrolipoamide dehydrogenase [EC:1.8.1.4] |
| K00382 | KSG66\_11350 | DLD, lpd, pdhD; dihydrolipoamide dehydrogenase [EC:1.8.1.4] |
| K00600 | KSG66\_17420 | glyA, SHMT; glycine hydroxymethyltransferase [EC:2.1.2.1] |
| K00605 | KSG66\_11630 | gcvT, AMT; aminomethyltransferase [EC:2.1.2.10] |
| K00626 | KSG66\_05195 | E2.3.1.9, atoB; acetyl-CoA C-acetyltransferase [EC:2.3.1.9] |
| K00626 | KSG66\_11405 | E2.3.1.9, atoB; acetyl-CoA C-acetyltransferase [EC:2.3.1.9] |
| K00865 | KSG66\_00095 | glxK, garK; glycerate 2-kinase [EC:2.7.1.165] |
| K01433 | KSG66\_06560 | purU; formyltetrahydrofolate deformylase [EC:3.5.1.10] |
| K01569 | KSG66\_15530 | oxdD; oxalate decarboxylase [EC:4.1.1.2] |
| K01625 | KSG66\_09070 | eda; 2-dehydro-3-deoxyphosphogluconate aldolase / (4S)-4-hydroxy-2-oxoglutarate aldolase [EC:4.1.2.14 4.1.3.42] |
| K01647 | KSG66\_04765 | CS, gltA; citrate synthase [EC:2.3.3.1] |
| K01647 | KSG66\_11390 | CS, gltA; citrate synthase [EC:2.3.3.1] |
| K01681 | KSG66\_08990 | ACO, acnA; aconitate hydratase [EC:4.2.1.3] |
| K01895 | KSG66\_13565 | ACSS, acs; acetyl-CoA synthetase [EC:6.2.1.1] |
| K01895 | KSG66\_13725 | ACSS, acs; acetyl-CoA synthetase [EC:6.2.1.1] |
| K01915 | KSG66\_08725 | glnA, GLUL; glutamine synthetase [EC:6.3.1.2] |
| K01966 | KSG66\_11285 | PCCB, pccB; propionyl-CoA carboxylase beta chain [EC:6.4.1.3 2.1.3.15] |
| K02437 | KSG66\_15270 | gcvH, GCSH; glycine cleavage system H protein |
| K03781 | KSG66\_04470 | katE, CAT, catB, srpA; catalase [EC:1.11.1.6] |
| K03781 | KSG66\_18315 | katE, CAT, catB, srpA; catalase [EC:1.11.1.6] |
| K03781 | KSG66\_18465 | katE, CAT, catB, srpA; catalase [EC:1.11.1.6] |
| K05606 | KSG66\_11290 | MCEE, epi; methylmalonyl-CoA/ethylmalonyl-CoA epimerase [EC:5.1.99.1] |
| K07246 | KSG66\_02965 | ttuC, dmlA; tartrate dehydrogenase/decarboxylase / D-malate dehydrogenase [EC:1.1.1.93 4.1.1.73 1.1.1.83] |
| K11473 | KSG66\_13185 | glcF; glycolate oxidase iron-sulfur subunit |

| 22 | Fatty acid metabolism | ko01212 || K00059 | KSG66\_07950 | fabG; 3-oxoacyl-[acyl-carrier protein] reductase [EC:1.1.1.100] |
| K00059 | KSG66\_08440 | fabG; 3-oxoacyl-[acyl-carrier protein] reductase [EC:1.1.1.100] |
| K00059 | KSG66\_09410 | fabG; 3-oxoacyl-[acyl-carrier protein] reductase [EC:1.1.1.100] |
| K00059 | KSG66\_11195 | fabG; 3-oxoacyl-[acyl-carrier protein] reductase [EC:1.1.1.100] |
| K00059 | KSG66\_13500 | fabG; 3-oxoacyl-[acyl-carrier protein] reductase [EC:1.1.1.100] |
| K00059 | KSG66\_15330 | fabG; 3-oxoacyl-[acyl-carrier protein] reductase [EC:1.1.1.100] |
| K00059 | KSG66\_15750 | fabG; 3-oxoacyl-[acyl-carrier protein] reductase [EC:1.1.1.100] |
| K00208 | KSG66\_05810 | fabI; enoyl-[acyl-carrier protein] reductase I [EC:1.3.1.9 1.3.1.10] |
| K00626 | KSG66\_05195 | E2.3.1.9, atoB; acetyl-CoA C-acetyltransferase [EC:2.3.1.9] |
| K00626 | KSG66\_11405 | E2.3.1.9, atoB; acetyl-CoA C-acetyltransferase [EC:2.3.1.9] |
| K00632 | KSG66\_15285 | fadA, fadI; acetyl-CoA acyltransferase [EC:2.3.1.16] |
| K00645 | KSG66\_07945 | fabD; [acyl-carrier-protein] S-malonyltransferase [EC:2.3.1.39] |
| K00645 | KSG66\_09175 | fabD; [acyl-carrier-protein] S-malonyltransferase [EC:2.3.1.39] |
| K00648 | KSG66\_05125 | fabH; 3-oxoacyl-[acyl-carrier-protein] synthase III [EC:2.3.1.180] |
| K00648 | KSG66\_05605 | fabH; 3-oxoacyl-[acyl-carrier-protein] synthase III [EC:2.3.1.180] |
| K01897 | KSG66\_02195 | ACSL, fadD; long-chain acyl-CoA synthetase [EC:6.2.1.3] |
| K01897 | KSG66\_05165 | ACSL, fadD; long-chain acyl-CoA synthetase [EC:6.2.1.3] |
| K01897 | KSG66\_05200 | ACSL, fadD; long-chain acyl-CoA synthetase [EC:6.2.1.3] |
| K01897 | KSG66\_13115 | ACSL, fadD; long-chain acyl-CoA synthetase [EC:6.2.1.3] |
| K01961 | KSG66\_09265 | accC; acetyl-CoA carboxylase, biotin carboxylase subunit [EC:6.4.1.2 6.3.4.14] |
| K01961 | KSG66\_11520 | accC; acetyl-CoA carboxylase, biotin carboxylase subunit [EC:6.4.1.2 6.3.4.14] |
| K01962 | KSG66\_13445 | accA; acetyl-CoA carboxylase carboxyl transferase subunit alpha [EC:6.4.1.2 2.1.3.15] |
| K01963 | KSG66\_13450 | accD; acetyl-CoA carboxylase carboxyl transferase subunit beta [EC:6.4.1.2 2.1.3.15] |
| K02160 | KSG66\_09260 | accB, bccP; acetyl-CoA carboxylase biotin carboxyl carrier protein |
| K02160 | KSG66\_11525 | accB, bccP; acetyl-CoA carboxylase biotin carboxyl carrier protein |
| K02372 | KSG66\_02120 | fabZ; 3-hydroxyacyl-[acyl-carrier-protein] dehydratase [EC:4.2.1.59] |
| K02372 | KSG66\_17165 | fabZ; 3-hydroxyacyl-[acyl-carrier-protein] dehydratase [EC:4.2.1.59] |
| K07516 | KSG66\_15290 | fadN; 3-hydroxyacyl-CoA dehydrogenase [EC:1.1.1.35] |
| K09458 | KSG66\_02925 | fabF; 3-oxoacyl-[acyl-carrier-protein] synthase II [EC:2.3.1.179] |
| K09458 | KSG66\_05610 | fabF; 3-oxoacyl-[acyl-carrier-protein] synthase II [EC:2.3.1.179] |
| K10780 | KSG66\_04270 | fabL; enoyl-[acyl-carrier protein] reductase III [EC:1.3.1.104] |
| K13767 | KSG66\_13105 | fadB; enoyl-CoA hydratase [EC:4.2.1.17] |

| 23 | Flagellar assembly | ko02040 || K02387 | KSG66\_08085 | flgB; flagellar basal-body rod protein FlgB |
| K02388 | KSG66\_08090 | flgC; flagellar basal-body rod protein FlgC |
| K02389 | KSG66\_08135 | flgD; flagellar basal-body rod modification protein FlgD |
| K02390 | KSG66\_08140 | flgE; flagellar hook protein FlgE |
| K02392 | KSG66\_17175 | flgG; flagellar basal-body rod protein FlgG |
| K02392 | KSG66\_17180 | flgG; flagellar basal-body rod protein FlgG |
| K02396 | KSG66\_16675 | flgK; flagellar hook-associated protein 1 FlgK |
| K02397 | KSG66\_16670 | flgL; flagellar hook-associated protein 3 FlgL |
| K02398 | KSG66\_16685 | flgM; negative regulator of flagellin synthesis FlgM |
| K02400 | KSG66\_08195 | flhA; flagellar biosynthesis protein FlhA |
| K02401 | KSG66\_08190 | flhB; flagellar biosynthetic protein FlhB |
| K02405 | KSG66\_08235 | fliA; RNA polymerase sigma factor for flagellar operon FliA |
| K02406 | KSG66\_16650 | fliC; flagellin |
| K02407 | KSG66\_16645 | fliD; flagellar hook-associated protein 2 |
| K02408 | KSG66\_08095 | fliE; flagellar hook-basal body complex protein FliE |
| K02409 | KSG66\_08100 | fliF; flagellar M-ring protein FliF |
| K02410 | KSG66\_08105 | fliG; flagellar motor switch protein FliG |
| K02411 | KSG66\_08110 | fliH; flagellar assembly protein FliH |
| K02412 | KSG66\_08115 | fliI; flagellum-specific ATP synthase [EC:3.6.3.50] |
| K02413 | KSG66\_08120 | fliJ; flagellar FliJ protein |
| K02414 | KSG66\_08130 | fliK; flagellar hook-length control protein FliK |
| K02416 | KSG66\_08155 | fliM; flagellar motor switch protein FliM |
| K02417 | KSG66\_08160 | fliNY, fliN; flagellar motor switch protein FliN/FliY |
| K02418 | KSG66\_08170 | fliOZ, fliO; flagellar protein FliO/FliZ |
| K02419 | KSG66\_08175 | fliP; flagellar biosynthetic protein FliP |
| K02420 | KSG66\_08180 | fliQ; flagellar biosynthetic protein FliQ |
| K02421 | KSG66\_08185 | fliR; flagellar biosynthetic protein FliR |
| K02422 | KSG66\_16640 | fliS; flagellar protein FliS |
| K02423 | KSG66\_16635 | fliT; flagellar protein FliT |
| K02556 | KSG66\_06820 | motA; chemotaxis protein MotA |
| K02556 | KSG66\_13750 | motA; chemotaxis protein MotA |
| K02557 | KSG66\_06815 | motB; chemotaxis protein MotB |

| 24 | Peptidoglycan biosynthesis | ko00550 || K00075 | KSG66\_07625 | murB; UDP-N-acetylmuramate dehydrogenase [EC:1.3.1.98] |
| K00790 | KSG66\_17350 | murA; UDP-N-acetylglucosamine 1-carboxyvinyltransferase [EC:2.5.1.7] |
| K00790 | KSG66\_17520 | murA; UDP-N-acetylglucosamine 1-carboxyvinyltransferase [EC:2.5.1.7] |
| K00806 | KSG66\_08265 | uppS; undecaprenyl diphosphate synthase [EC:2.5.1.31] |
| K00887 | KSG66\_11985 | dgkA; undecaprenol kinase [EC:2.7.1.66] |
| K01000 | KSG66\_07605 | mraY; phospho-N-acetylmuramoyl-pentapeptide-transferase [EC:2.7.8.13] |
| K01921 | KSG66\_02435 | ddl; D-alanine-D-alanine ligase [EC:6.3.2.4] |
| K01924 | KSG66\_13780 | murC; UDP-N-acetylmuramate--alanine ligase [EC:6.3.2.8] |
| K01925 | KSG66\_07610 | murD; UDP-N-acetylmuramoylalanine--D-glutamate ligase [EC:6.3.2.9] |
| K01928 | KSG66\_07600 | murE; UDP-N-acetylmuramoyl-L-alanyl-D-glutamate--2,6-diaminopimelate ligase [EC:6.3.2.13] |
| K01929 | KSG66\_02440 | murF; UDP-N-acetylmuramoyl-tripeptide--D-alanyl-D-alanine ligase [EC:6.3.2.10] |
| K02563 | KSG66\_07620 | murG; UDP-N-acetylglucosamine--N-acetylmuramyl-(pentapeptide) pyrophosphoryl-undecaprenol N-acetylglucosamine transferase [EC:2.4.1.227] |
| K05366 | KSG66\_10400 | mrcA; penicillin-binding protein 1A [EC:2.4.1.129 3.4.16.4] |
| K06153 | KSG66\_14415 | bacA; undecaprenyl-diphosphatase [EC:3.6.1.27] |
| K07258 | KSG66\_00075 | dacC, dacA, dacD; serine-type D-Ala-D-Ala carboxypeptidase (penicillin-binding protein 5/6) [EC:3.4.16.4] |
| K07258 | KSG66\_10830 | dacC, dacA, dacD; serine-type D-Ala-D-Ala carboxypeptidase (penicillin-binding protein 5/6) [EC:3.4.16.4] |
| K07258 | KSG66\_10965 | dacC, dacA, dacD; serine-type D-Ala-D-Ala carboxypeptidase (penicillin-binding protein 5/6) [EC:3.4.16.4] |
| K07259 | KSG66\_09315 | dacB; serine-type D-Ala-D-Ala carboxypeptidase/endopeptidase (penicillin-binding protein 4) [EC:3.4.16.4 3.4.21.-] |
| K07260 | KSG66\_09780 | vanY; zinc D-Ala-D-Ala carboxypeptidase [EC:3.4.17.14] |
| K08384 | KSG66\_07595 | spoVD; stage V sporulation protein D (sporulation-specific penicillin-binding protein) |
| K08724 | KSG66\_07590 | pbpB; penicillin-binding protein 2B |
| K12555 | KSG66\_05095 | pbp2A; penicillin-binding protein 2A [EC:2.4.1.129 3.4.16.4] |
| K18770 | KSG66\_14595 | pbpD; penicillin-binding protein 4 [EC:2.4.1.129 3.4.16.4] |
| K19302 | KSG66\_03995 | bcrC; undecaprenyl-diphosphatase [EC:3.6.1.27] |
| K19302 | KSG66\_17235 | bcrC; undecaprenyl-diphosphatase [EC:3.6.1.27] |
| K21464 | KSG66\_17675 | pbpG; penicillin-binding protein 2D [EC:2.4.1.129 3.4.16.4] |
| K21465 | KSG66\_11835 | pbpA; penicillin-binding protein A |
| K21466 | KSG66\_06970 | pbpH; penicillin-binding protein H |
| K21469 | KSG66\_01065 | pbp4b; serine-type D-Ala-D-Ala carboxypeptidase [EC:3.4.16.4] |

| 25 | Fatty acid biosynthesis | ko00061 || K00059 | KSG66\_07950 | fabG; 3-oxoacyl-[acyl-carrier protein] reductase [EC:1.1.1.100] |
| K00059 | KSG66\_08440 | fabG; 3-oxoacyl-[acyl-carrier protein] reductase [EC:1.1.1.100] |
| K00059 | KSG66\_09410 | fabG; 3-oxoacyl-[acyl-carrier protein] reductase [EC:1.1.1.100] |
| K00059 | KSG66\_11195 | fabG; 3-oxoacyl-[acyl-carrier protein] reductase [EC:1.1.1.100] |
| K00059 | KSG66\_13500 | fabG; 3-oxoacyl-[acyl-carrier protein] reductase [EC:1.1.1.100] |
| K00059 | KSG66\_15330 | fabG; 3-oxoacyl-[acyl-carrier protein] reductase [EC:1.1.1.100] |
| K00059 | KSG66\_15750 | fabG; 3-oxoacyl-[acyl-carrier protein] reductase [EC:1.1.1.100] |
| K00208 | KSG66\_05810 | fabI; enoyl-[acyl-carrier protein] reductase I [EC:1.3.1.9 1.3.1.10] |
| K00645 | KSG66\_07945 | fabD; [acyl-carrier-protein] S-malonyltransferase [EC:2.3.1.39] |
| K00645 | KSG66\_09175 | fabD; [acyl-carrier-protein] S-malonyltransferase [EC:2.3.1.39] |
| K00648 | KSG66\_05125 | fabH; 3-oxoacyl-[acyl-carrier-protein] synthase III [EC:2.3.1.180] |
| K00648 | KSG66\_05605 | fabH; 3-oxoacyl-[acyl-carrier-protein] synthase III [EC:2.3.1.180] |
| K01071 | KSG66\_16565 | MCH; medium-chain acyl-[acyl-carrier-protein] hydrolase [EC:3.1.2.21] |
| K01897 | KSG66\_02195 | ACSL, fadD; long-chain acyl-CoA synthetase [EC:6.2.1.3] |
| K01897 | KSG66\_05165 | ACSL, fadD; long-chain acyl-CoA synthetase [EC:6.2.1.3] |
| K01897 | KSG66\_05200 | ACSL, fadD; long-chain acyl-CoA synthetase [EC:6.2.1.3] |
| K01897 | KSG66\_13115 | ACSL, fadD; long-chain acyl-CoA synthetase [EC:6.2.1.3] |
| K01961 | KSG66\_09265 | accC; acetyl-CoA carboxylase, biotin carboxylase subunit [EC:6.4.1.2 6.3.4.14] |
| K01961 | KSG66\_11520 | accC; acetyl-CoA carboxylase, biotin carboxylase subunit [EC:6.4.1.2 6.3.4.14] |
| K01962 | KSG66\_13445 | accA; acetyl-CoA carboxylase carboxyl transferase subunit alpha [EC:6.4.1.2 2.1.3.15] |
| K01963 | KSG66\_13450 | accD; acetyl-CoA carboxylase carboxyl transferase subunit beta [EC:6.4.1.2 2.1.3.15] |
| K02160 | KSG66\_09260 | accB, bccP; acetyl-CoA carboxylase biotin carboxyl carrier protein |
| K02160 | KSG66\_11525 | accB, bccP; acetyl-CoA carboxylase biotin carboxyl carrier protein |
| K02372 | KSG66\_02120 | fabZ; 3-hydroxyacyl-[acyl-carrier-protein] dehydratase [EC:4.2.1.59] |
| K02372 | KSG66\_17165 | fabZ; 3-hydroxyacyl-[acyl-carrier-protein] dehydratase [EC:4.2.1.59] |
| K09458 | KSG66\_02925 | fabF; 3-oxoacyl-[acyl-carrier-protein] synthase II [EC:2.3.1.179] |
| K09458 | KSG66\_05610 | fabF; 3-oxoacyl-[acyl-carrier-protein] synthase II [EC:2.3.1.179] |
| K10780 | KSG66\_04270 | fabL; enoyl-[acyl-carrier protein] reductase III [EC:1.3.1.104] |

| 26 | 2-Oxocarboxylic acid metabolism | ko01210 || K00031 | KSG66\_13390 | IDH1, IDH2, icd; isocitrate dehydrogenase [EC:1.1.1.42] |
| K00052 | KSG66\_12970 | leuB, IMDH; 3-isopropylmalate dehydrogenase [EC:1.1.1.85] |
| K00053 | KSG66\_12980 | ilvC; ketol-acid reductoisomerase [EC:1.1.1.86] |
| K00133 | KSG66\_08375 | asd; aspartate-semialdehyde dehydrogenase [EC:1.2.1.11] |
| K00145 | KSG66\_05535 | argC; N-acetyl-gamma-glutamyl-phosphate reductase [EC:1.2.1.38] |
| K00620 | KSG66\_05540 | argJ; glutamate N-acetyltransferase / amino-acid N-acetyltransferase [EC:2.3.1.35 2.3.1.1] |
| K00812 | KSG66\_10425 | aspB; aspartate aminotransferase [EC:2.6.1.1] |
| K00821 | KSG66\_05550 | argD; acetylornithine/N-succinyldiaminopimelate aminotransferase [EC:2.6.1.11 2.6.1.17] |
| K00826 | KSG66\_01380 | E2.6.1.42, ilvE; branched-chain amino acid aminotransferase [EC:2.6.1.42] |
| K00826 | KSG66\_18220 | E2.6.1.42, ilvE; branched-chain amino acid aminotransferase [EC:2.6.1.42] |
| K00928 | KSG66\_02010 | lysC; aspartate kinase [EC:2.7.2.4] |
| K00928 | KSG66\_08380 | lysC; aspartate kinase [EC:2.7.2.4] |
| K00928 | KSG66\_13075 | lysC; aspartate kinase [EC:2.7.2.4] |
| K00930 | KSG66\_05545 | argB; acetylglutamate kinase [EC:2.7.2.8] |
| K01438 | KSG66\_09840 | argE; acetylornithine deacetylase [EC:3.5.1.16] |
| K01647 | KSG66\_04765 | CS, gltA; citrate synthase [EC:2.3.3.1] |
| K01647 | KSG66\_11390 | CS, gltA; citrate synthase [EC:2.3.3.1] |
| K01649 | KSG66\_12975 | leuA, IMS; 2-isopropylmalate synthase [EC:2.3.3.13] |
| K01652 | KSG66\_12990 | E2.2.1.6L, ilvB, ilvG, ilvI; acetolactate synthase I/II/III large subunit [EC:2.2.1.6] |
| K01652 | KSG66\_16980 | E2.2.1.6L, ilvB, ilvG, ilvI; acetolactate synthase I/II/III large subunit [EC:2.2.1.6] |
| K01653 | KSG66\_12985 | E2.2.1.6S, ilvH, ilvN; acetolactate synthase I/III small subunit [EC:2.2.1.6] |
| K01681 | KSG66\_08990 | ACO, acnA; aconitate hydratase [EC:4.2.1.3] |
| K01687 | KSG66\_10150 | ilvD; dihydroxy-acid dehydratase [EC:4.2.1.9] |
| K01703 | KSG66\_12965 | leuC, IPMI-L; 3-isopropylmalate/(R)-2-methylmalate dehydratase large subunit [EC:4.2.1.33 4.2.1.35] |
| K01704 | KSG66\_12960 | leuD, IPMI-S; 3-isopropylmalate/(R)-2-methylmalate dehydratase small subunit [EC:4.2.1.33 4.2.1.35] |
| K05825 | KSG66\_16095 | LYSN; 2-aminoadipate transaminase [EC:2.6.1.-] |
| K11358 | KSG66\_04815 | yhdR; aspartate aminotransferase [EC:2.6.1.1] |

| 27 | Aminoacyl-tRNA biosynthesis | ko00970 || K00604 | KSG66\_07860 | MTFMT, fmt; methionyl-tRNA formyltransferase [EC:2.1.2.9] |
| K01866 | KSG66\_13720 | YARS, tyrS; tyrosyl-tRNA synthetase [EC:6.1.1.1] |
| K01867 | KSG66\_05650 | WARS, trpS; tryptophanyl-tRNA synthetase [EC:6.1.1.2] |
| K01868 | KSG66\_09365 | TARS, thrS; threonyl-tRNA synthetase [EC:6.1.1.3] |
| K01868 | KSG66\_13300 | TARS, thrS; threonyl-tRNA synthetase [EC:6.1.1.3] |
| K01869 | KSG66\_13950 | LARS, leuS; leucyl-tRNA synthetase [EC:6.1.1.4] |
| K01870 | KSG66\_07710 | IARS, ileS; isoleucyl-tRNA synthetase [EC:6.1.1.5] |
| K01872 | KSG66\_12550 | AARS, alaS; alanyl-tRNA synthetase [EC:6.1.1.7] |
| K01873 | KSG66\_12875 | VARS, valS; valyl-tRNA synthetase [EC:6.1.1.9] |
| K01874 | KSG66\_00255 | MARS, metG; methionyl-tRNA synthetase [EC:6.1.1.10] |
| K01875 | KSG66\_00090 | SARS, serS; seryl-tRNA synthetase [EC:6.1.1.11] |
| K01876 | KSG66\_12635 | aspS; aspartyl-tRNA synthetase [EC:6.1.1.12] |
| K01878 | KSG66\_11960 | glyQ; glycyl-tRNA synthetase alpha chain [EC:6.1.1.14] |
| K01879 | KSG66\_11955 | glyS; glycyl-tRNA synthetase beta chain [EC:6.1.1.14] |
| K01881 | KSG66\_08285 | PARS, proS; prolyl-tRNA synthetase [EC:6.1.1.15] |
| K01883 | KSG66\_00620 | CARS, cysS; cysteinyl-tRNA synthetase [EC:6.1.1.16] |
| K01887 | KSG66\_17630 | RARS, argS; arginyl-tRNA synthetase [EC:6.1.1.19] |
| K01889 | KSG66\_13160 | FARSA, pheS; phenylalanyl-tRNA synthetase alpha chain [EC:6.1.1.20] |
| K01890 | KSG66\_13155 | FARSB, pheT; phenylalanyl-tRNA synthetase beta chain [EC:6.1.1.20] |
| K01892 | KSG66\_12640 | HARS, hisS; histidyl-tRNA synthetase [EC:6.1.1.21] |
| K01893 | KSG66\_10420 | NARS, asnS; asparaginyl-tRNA synthetase [EC:6.1.1.22] |
| K02433 | KSG66\_03470 | gatA, QRSL1; aspartyl-tRNA(Asn)/glutamyl-tRNA(Gln) amidotransferase subunit A [EC:6.3.5.6 6.3.5.7] |
| K02434 | KSG66\_03475 | gatB, PET112; aspartyl-tRNA(Asn)/glutamyl-tRNA(Gln) amidotransferase subunit B [EC:6.3.5.6 6.3.5.7] |
| K02435 | KSG66\_03465 | gatC, GATC; aspartyl-tRNA(Asn)/glutamyl-tRNA(Gln) amidotransferase subunit C [EC:6.3.5.6 6.3.5.7] |
| K04567 | KSG66\_00485 | KARS, lysS; lysyl-tRNA synthetase, class II [EC:6.1.1.6] |
| K09698 | KSG66\_00610 | gltX; nondiscriminating glutamyl-tRNA synthetase [EC:6.1.1.24] |

| 28 | Butanoate metabolism | ko00650 || K00004 | KSG66\_01015 | BDH, butB; (R,R)-butanediol dehydrogenase / meso-butanediol dehydrogenase / diacetyl reductase [EC:1.1.1.4 1.1.1.- 1.1.1.303] |
| K00004 | KSG66\_03255 | BDH, butB; (R,R)-butanediol dehydrogenase / meso-butanediol dehydrogenase / diacetyl reductase [EC:1.1.1.4 1.1.1.- 1.1.1.303] |
| K00019 | KSG66\_09180 | E1.1.1.30, bdh; 3-hydroxybutyrate dehydrogenase [EC:1.1.1.30] |
| K00019 | KSG66\_19245 | E1.1.1.30, bdh; 3-hydroxybutyrate dehydrogenase [EC:1.1.1.30] |
| K00074 | KSG66\_11400 | paaH, hbd, fadB, mmgB; 3-hydroxybutyryl-CoA dehydrogenase [EC:1.1.1.157] |
| K00135 | KSG66\_02070 | gabD; succinate-semialdehyde dehydrogenase / glutarate-semialdehyde dehydrogenase [EC:1.2.1.16 1.2.1.79 1.2.1.20] |
| K00239 | KSG66\_13060 | sdhA, frdA; succinate dehydrogenase / fumarate reductase, flavoprotein subunit [EC:1.3.5.1 1.3.5.4] |
| K00240 | KSG66\_13055 | sdhB, frdB; succinate dehydrogenase / fumarate reductase, iron-sulfur subunit [EC:1.3.5.1 1.3.5.4] |
| K00241 | KSG66\_13065 | sdhC, frdC; succinate dehydrogenase / fumarate reductase, cytochrome b subunit |
| K00626 | KSG66\_05195 | E2.3.1.9, atoB; acetyl-CoA C-acetyltransferase [EC:2.3.1.9] |
| K00626 | KSG66\_11405 | E2.3.1.9, atoB; acetyl-CoA C-acetyltransferase [EC:2.3.1.9] |
| K00634 | KSG66\_11365 | ptb; phosphate butyryltransferase [EC:2.3.1.19] |
| K00929 | KSG66\_11355 | buk; butyrate kinase [EC:2.7.2.7] |
| K01028 | KSG66\_09190 | E2.8.3.5A, scoA; 3-oxoacid CoA-transferase subunit A [EC:2.8.3.5] |
| K01029 | KSG66\_09185 | E2.8.3.5B, scoB; 3-oxoacid CoA-transferase subunit B [EC:2.8.3.5] |
| K01034 | KSG66\_09850 | atoD; acetate CoA/acetoacetate CoA-transferase alpha subunit [EC:2.8.3.8 2.8.3.9] |
| K01035 | KSG66\_09845 | atoA; acetate CoA/acetoacetate CoA-transferase beta subunit [EC:2.8.3.8 2.8.3.9] |
| K01575 | KSG66\_16975 | alsD, budA, aldC; acetolactate decarboxylase [EC:4.1.1.5] |
| K01640 | KSG66\_09255 | E4.1.3.4, HMGCL, hmgL; hydroxymethylglutaryl-CoA lyase [EC:4.1.3.4] |
| K01652 | KSG66\_12990 | E2.2.1.6L, ilvB, ilvG, ilvI; acetolactate synthase I/II/III large subunit [EC:2.2.1.6] |
| K01652 | KSG66\_16980 | E2.2.1.6L, ilvB, ilvG, ilvI; acetolactate synthase I/II/III large subunit [EC:2.2.1.6] |
| K01653 | KSG66\_12985 | E2.2.1.6S, ilvH, ilvN; acetolactate synthase I/III small subunit [EC:2.2.1.6] |
| K07246 | KSG66\_02965 | ttuC, dmlA; tartrate dehydrogenase/decarboxylase / D-malate dehydrogenase [EC:1.1.1.93 4.1.1.73 1.1.1.83] |
| K07250 | KSG66\_02065 | gabT; 4-aminobutyrate aminotransferase / (S)-3-amino-2-methylpropionate transaminase / 5-aminovalerate transaminase [EC:2.6.1.19 2.6.1.22 2.6.1.48] |
| K07516 | KSG66\_15290 | fadN; 3-hydroxyacyl-CoA dehydrogenase [EC:1.1.1.35] |

| 29 | Pentose phosphate pathway | ko00030 || K00033 | KSG66\_11260 | PGD, gnd, gntZ; 6-phosphogluconate dehydrogenase [EC:1.1.1.44 1.1.1.343] |
| K00034 | KSG66\_01550 | gdh; glucose 1-dehydrogenase [EC:1.1.1.47] |
| K00034 | KSG66\_02080 | gdh; glucose 1-dehydrogenase [EC:1.1.1.47] |
| K00036 | KSG66\_11255 | G6PD, zwf; glucose-6-phosphate 1-dehydrogenase [EC:1.1.1.49 1.1.1.363] |
| K00090 | KSG66\_16350 | ghrB; glyoxylate/hydroxypyruvate/2-ketogluconate reductase [EC:1.1.1.79 1.1.1.81 1.1.1.215] |
| K00615 | KSG66\_08935 | E2.2.1.1, tktA, tktB; transketolase [EC:2.2.1.1] |
| K00616 | KSG66\_17525 | E2.2.1.2, talA, talB; transaldolase [EC:2.2.1.2] |
| K00850 | KSG66\_13440 | pfkA, PFK; 6-phosphofructokinase 1 [EC:2.7.1.11] |
| K00851 | KSG66\_16055 | E2.7.1.12, gntK, idnK; gluconokinase [EC:2.7.1.12] |
| K00852 | KSG66\_16940 | rbsK, RBKS; ribokinase [EC:2.7.1.15] |
| K00874 | KSG66\_09060 | kdgK; 2-dehydro-3-deoxygluconokinase [EC:2.7.1.45] |
| K00948 | KSG66\_00320 | PRPS, prsA; ribose-phosphate pyrophosphokinase [EC:2.7.6.1] |
| K01619 | KSG66\_18610 | deoC, DERA; deoxyribose-phosphate aldolase [EC:4.1.2.4] |
| K01624 | KSG66\_17530 | FBA, fbaA; fructose-bisphosphate aldolase, class II [EC:4.1.2.13] |
| K01625 | KSG66\_09070 | eda; 2-dehydro-3-deoxyphosphogluconate aldolase / (4S)-4-hydroxy-2-oxoglutarate aldolase [EC:4.1.2.14 4.1.3.42] |
| K01783 | KSG66\_07890 | rpe, RPE; ribulose-phosphate 3-epimerase [EC:5.1.3.1] |
| K01808 | KSG66\_17430 | rpiB; ribose 5-phosphate isomerase B [EC:5.3.1.6] |
| K01810 | KSG66\_14515 | GPI, pgi; glucose-6-phosphate isomerase [EC:5.3.1.9] |
| K01835 | KSG66\_04700 | pgm; phosphoglucomutase [EC:5.4.2.2] |
| K01839 | KSG66\_10975 | deoB; phosphopentomutase [EC:5.4.2.7] |
| K02446 | KSG66\_17515 | glpX; fructose-1,6-bisphosphatase II [EC:3.1.3.11] |
| K04041 | KSG66\_18855 | fbp3; fructose-1,6-bisphosphatase III [EC:3.1.3.11] |
| K08093 | KSG66\_01825 | hxlA; 3-hexulose-6-phosphate synthase [EC:4.1.2.43] |
| K08094 | KSG66\_01820 | hxlB; 6-phospho-3-hexuloisomerase [EC:5.3.1.27] |

| 30 | Valine, leucine and isoleucine degradation | ko00280 || K00020 | KSG66\_06960 | mmsB, HIBADH; 3-hydroxyisobutyrate dehydrogenase [EC:1.1.1.31] |
| K00128 | KSG66\_09600 | ALDH; aldehyde dehydrogenase (NAD+) [EC:1.2.1.3] |
| K00128 | KSG66\_13620 | ALDH; aldehyde dehydrogenase (NAD+) [EC:1.2.1.3] |
| K00128 | KSG66\_18800 | ALDH; aldehyde dehydrogenase (NAD+) [EC:1.2.1.3] |
| K00140 | KSG66\_18750 | mmsA, iolA, ALDH6A1; malonate-semialdehyde dehydrogenase (acetylating) / methylmalonate-semialdehyde dehydrogenase [EC:1.2.1.18 1.2.1.27] |
| K00166 | KSG66\_11345 | BCKDHA, bkdA1; 2-oxoisovalerate dehydrogenase E1 component alpha subunit [EC:1.2.4.4] |
| K00167 | KSG66\_11340 | BCKDHB, bkdA2; 2-oxoisovalerate dehydrogenase E1 component beta subunit [EC:1.2.4.4] |
| K00263 | KSG66\_11360 | E1.4.1.9; leucine dehydrogenase [EC:1.4.1.9] |
| K00382 | KSG66\_04065 | DLD, lpd, pdhD; dihydrolipoamide dehydrogenase [EC:1.8.1.4] |
| K00382 | KSG66\_07320 | DLD, lpd, pdhD; dihydrolipoamide dehydrogenase [EC:1.8.1.4] |
| K00382 | KSG66\_11350 | DLD, lpd, pdhD; dihydrolipoamide dehydrogenase [EC:1.8.1.4] |
| K00626 | KSG66\_05195 | E2.3.1.9, atoB; acetyl-CoA C-acetyltransferase [EC:2.3.1.9] |
| K00626 | KSG66\_11405 | E2.3.1.9, atoB; acetyl-CoA C-acetyltransferase [EC:2.3.1.9] |
| K00632 | KSG66\_15285 | fadA, fadI; acetyl-CoA acyltransferase [EC:2.3.1.16] |
| K00826 | KSG66\_01380 | E2.6.1.42, ilvE; branched-chain amino acid aminotransferase [EC:2.6.1.42] |
| K00826 | KSG66\_18220 | E2.6.1.42, ilvE; branched-chain amino acid aminotransferase [EC:2.6.1.42] |
| K01028 | KSG66\_09190 | E2.8.3.5A, scoA; 3-oxoacid CoA-transferase subunit A [EC:2.8.3.5] |
| K01029 | KSG66\_09185 | E2.8.3.5B, scoB; 3-oxoacid CoA-transferase subunit B [EC:2.8.3.5] |
| K01640 | KSG66\_09255 | E4.1.3.4, HMGCL, hmgL; hydroxymethylglutaryl-CoA lyase [EC:4.1.3.4] |
| K01966 | KSG66\_11285 | PCCB, pccB; propionyl-CoA carboxylase beta chain [EC:6.4.1.3 2.1.3.15] |
| K05606 | KSG66\_11290 | MCEE, epi; methylmalonyl-CoA/ethylmalonyl-CoA epimerase [EC:5.1.99.1] |
| K05607 | KSG66\_09250 | AUH; methylglutaconyl-CoA hydratase [EC:4.2.1.18] |
| K07250 | KSG66\_02065 | gabT; 4-aminobutyrate aminotransferase / (S)-3-amino-2-methylpropionate transaminase / 5-aminovalerate transaminase [EC:2.6.1.19 2.6.1.22 2.6.1.48] |
| K09699 | KSG66\_11335 | DBT, bkdB; 2-oxoisovalerate dehydrogenase E2 component (dihydrolipoyl transacylase) [EC:2.3.1.168] |

| 31 | Carbon fixation pathways in prokaryotes | ko00720 || K00024 | KSG66\_13385 | mdh; malate dehydrogenase [EC:1.1.1.37] |
| K00031 | KSG66\_13390 | IDH1, IDH2, icd; isocitrate dehydrogenase [EC:1.1.1.42] |
| K00239 | KSG66\_13060 | sdhA, frdA; succinate dehydrogenase / fumarate reductase, flavoprotein subunit [EC:1.3.5.1 1.3.5.4] |
| K00240 | KSG66\_13055 | sdhB, frdB; succinate dehydrogenase / fumarate reductase, iron-sulfur subunit [EC:1.3.5.1 1.3.5.4] |
| K00241 | KSG66\_13065 | sdhC, frdC; succinate dehydrogenase / fumarate reductase, cytochrome b subunit |
| K00625 | KSG66\_17785 | E2.3.1.8, pta; phosphate acetyltransferase [EC:2.3.1.8] |
| K00626 | KSG66\_05195 | E2.3.1.9, atoB; acetyl-CoA C-acetyltransferase [EC:2.3.1.9] |
| K00626 | KSG66\_11405 | E2.3.1.9, atoB; acetyl-CoA C-acetyltransferase [EC:2.3.1.9] |
| K00925 | KSG66\_13520 | ackA; acetate kinase [EC:2.7.2.1] |
| K01491 | KSG66\_11505 | folD; methylenetetrahydrofolate dehydrogenase (NADP+) / methenyltetrahydrofolate cyclohydrolase [EC:1.5.1.5 3.5.4.9] |
| K01679 | KSG66\_15415 | E4.2.1.2B, fumC, FH; fumarate hydratase, class II [EC:4.2.1.2] |
| K01681 | KSG66\_08990 | ACO, acnA; aconitate hydratase [EC:4.2.1.3] |
| K01895 | KSG66\_13565 | ACSS, acs; acetyl-CoA synthetase [EC:6.2.1.1] |
| K01895 | KSG66\_13725 | ACSS, acs; acetyl-CoA synthetase [EC:6.2.1.1] |
| K01902 | KSG66\_08045 | sucD; succinyl-CoA synthetase alpha subunit [EC:6.2.1.5] |
| K01903 | KSG66\_08040 | sucC; succinyl-CoA synthetase beta subunit [EC:6.2.1.5] |
| K01958 | KSG66\_07440 | PC, pyc; pyruvate carboxylase [EC:6.4.1.1] |
| K01961 | KSG66\_09265 | accC; acetyl-CoA carboxylase, biotin carboxylase subunit [EC:6.4.1.2 6.3.4.14] |
| K01961 | KSG66\_11520 | accC; acetyl-CoA carboxylase, biotin carboxylase subunit [EC:6.4.1.2 6.3.4.14] |
| K01962 | KSG66\_13445 | accA; acetyl-CoA carboxylase carboxyl transferase subunit alpha [EC:6.4.1.2 2.1.3.15] |
| K01963 | KSG66\_13450 | accD; acetyl-CoA carboxylase carboxyl transferase subunit beta [EC:6.4.1.2 2.1.3.15] |
| K02160 | KSG66\_09260 | accB, bccP; acetyl-CoA carboxylase biotin carboxyl carrier protein |
| K02160 | KSG66\_11525 | accB, bccP; acetyl-CoA carboxylase biotin carboxyl carrier protein |
| K05606 | KSG66\_11290 | MCEE, epi; methylmalonyl-CoA/ethylmalonyl-CoA epimerase [EC:5.1.99.1] |

| 32 | Arginine and proline metabolism | ko00330 || K00128 | KSG66\_09600 | ALDH; aldehyde dehydrogenase (NAD+) [EC:1.2.1.3] |
| K00128 | KSG66\_13620 | ALDH; aldehyde dehydrogenase (NAD+) [EC:1.2.1.3] |
| K00128 | KSG66\_18800 | ALDH; aldehyde dehydrogenase (NAD+) [EC:1.2.1.3] |
| K00147 | KSG66\_06570 | proA; glutamate-5-semialdehyde dehydrogenase [EC:1.2.1.41] |
| K00286 | KSG66\_09400 | proC; pyrroline-5-carboxylate reductase [EC:1.5.1.2] |
| K00286 | KSG66\_11140 | proC; pyrroline-5-carboxylate reductase [EC:1.5.1.2] |
| K00294 | KSG66\_01730 | E1.2.1.88; 1-pyrroline-5-carboxylate dehydrogenase [EC:1.2.1.88] |
| K00294 | KSG66\_17850 | E1.2.1.88; 1-pyrroline-5-carboxylate dehydrogenase [EC:1.2.1.88] |
| K00318 | KSG66\_01725 | PRODH; proline dehydrogenase [EC:1.5.-.-] |
| K00318 | KSG66\_15300 | PRODH; proline dehydrogenase [EC:1.5.-.-] |
| K00491 | KSG66\_03800 | nos; nitric-oxide synthase, bacterial [EC:1.14.14.47] |
| K00657 | KSG66\_02940 | speG, SAT; diamine N-acetyltransferase [EC:2.3.1.57] |
| K00797 | KSG66\_17670 | speE, SRM; spermidine synthase [EC:2.5.1.16] |
| K00812 | KSG66\_10425 | aspB; aspartate aminotransferase [EC:2.6.1.1] |
| K00819 | KSG66\_18965 | rocD, OAT; ornithine--oxo-acid transaminase [EC:2.6.1.13] |
| K00824 | KSG66\_04865 | dat; D-alanine transaminase [EC:2.6.1.21] |
| K00931 | KSG66\_06565 | proB; glutamate 5-kinase [EC:2.7.2.11] |
| K00931 | KSG66\_09395 | proB; glutamate 5-kinase [EC:2.7.2.11] |
| K01476 | KSG66\_18955 | E3.5.3.1, rocF, arg; arginase [EC:3.5.3.1] |
| K01480 | KSG66\_17665 | speB; agmatinase [EC:3.5.3.11] |
| K01585 | KSG66\_07340 | speA; arginine decarboxylase [EC:4.1.1.19] |
| K01611 | KSG66\_13330 | speD, AMD1; S-adenosylmethionine decarboxylase [EC:4.1.1.50] |
| K11358 | KSG66\_04815 | yhdR; aspartate aminotransferase [EC:2.6.1.1] |

| 33 | Folate biosynthesis | ko00790 || K00287 | KSG66\_10125 | DHFR, folA; dihydrofolate reductase [EC:1.5.1.3] |
| K00796 | KSG66\_00460 | folP; dihydropteroate synthase [EC:2.5.1.15] |
| K00950 | KSG66\_00470 | folK; 2-amino-4-hydroxy-6-hydroxymethyldihydropteridine diphosphokinase [EC:2.7.6.3] |
| K01077 | KSG66\_04750 | E3.1.3.1, phoA, phoB; alkaline phosphatase [EC:3.1.3.1] |
| K01113 | KSG66\_01470 | phoD; alkaline phosphatase D [EC:3.1.3.1] |
| K01495 | KSG66\_10635 | GCH1, folE; GTP cyclohydrolase IA [EC:3.5.4.16] |
| K01633 | KSG66\_00465 | folB; 7,8-dihydroneopterin aldolase/epimerase/oxygenase [EC:4.1.2.25 5.1.99.8 1.13.11.81] |
| K01664 | KSG66\_00450 | pabA; para-aminobenzoate synthetase component II [EC:2.6.1.85] |
| K01665 | KSG66\_00445 | pabB; para-aminobenzoate synthetase component I [EC:2.6.1.85] |
| K01737 | KSG66\_06845 | queD, ptpS, PTS; 6-pyruvoyltetrahydropterin/6-carboxytetrahydropterin synthase [EC:4.2.3.12 4.1.2.50] |
| K02619 | KSG66\_00455 | pabC; 4-amino-4-deoxychorismate lyase [EC:4.1.3.38] |
| K03635 | KSG66\_07125 | MOCS2B, moaE; molybdopterin synthase catalytic subunit [EC:2.8.1.12] |
| K03637 | KSG66\_03130 | moaC, CNX3; cyclic pyranopterin monophosphate synthase [EC:4.6.1.17] |
| K03638 | KSG66\_13515 | moaB; molybdopterin adenylyltransferase [EC:2.7.7.75] |
| K03639 | KSG66\_17320 | moaA, CNX2; GTP 3',8-cyclase [EC:4.1.99.22] |
| K03750 | KSG66\_07115 | moeA; molybdopterin molybdotransferase [EC:2.10.1.1] |
| K03752 | KSG66\_07105 | mobA; molybdenum cofactor guanylyltransferase [EC:2.7.7.77] |
| K06920 | KSG66\_06840 | queC; 7-cyano-7-deazaguanine synthase [EC:6.3.4.20] |
| K07141 | KSG66\_18875 | mocA; molybdenum cofactor cytidylyltransferase [EC:2.7.7.76] |
| K09457 | KSG66\_06855 | queF; 7-cyano-7-deazaguanine reductase [EC:1.7.1.13] |
| K10026 | KSG66\_06850 | queE; 7-carboxy-7-deazaguanine synthase [EC:4.3.99.3] |
| K11754 | KSG66\_12870 | folC; dihydrofolate synthase / folylpolyglutamate synthase [EC:6.3.2.12 6.3.2.17] |
| K14652 | KSG66\_10870 | ribBA; 3,4-dihydroxy 2-butanone 4-phosphate synthase / GTP cyclohydrolase II [EC:4.1.99.12 3.5.4.25] |

| 34 | Phosphotransferase system (PTS) | ko02060 || K00882 | KSG66\_07160 | fruK; 1-phosphofructokinase [EC:2.7.1.56] |
| K02750 | KSG66\_04090 | PTS-Glv-EIIC, glvC, malP, aglA; PTS system, alpha-glucoside-specific IIC component |
| K02759 | KSG66\_18230 | PTS-Cel-EIIA, celC, chbA; PTS system, cellobiose-specific IIA component [EC:2.7.1.196 2.7.1.205] |
| K02759 | KSG66\_18285 | PTS-Cel-EIIA, celC, chbA; PTS system, cellobiose-specific IIA component [EC:2.7.1.196 2.7.1.205] |
| K02760 | KSG66\_18240 | PTS-Cel-EIIB, celA, chbB; PTS system, cellobiose-specific IIB component [EC:2.7.1.196 2.7.1.205] |
| K02760 | KSG66\_18280 | PTS-Cel-EIIB, celA, chbB; PTS system, cellobiose-specific IIB component [EC:2.7.1.196 2.7.1.205] |
| K02761 | KSG66\_18170 | PTS-Cel-EIIC, celB, chbC; PTS system, cellobiose-specific IIC component |
| K02761 | KSG66\_18235 | PTS-Cel-EIIC, celB, chbC; PTS system, cellobiose-specific IIC component |
| K02761 | KSG66\_18290 | PTS-Cel-EIIC, celB, chbC; PTS system, cellobiose-specific IIC component |
| K02770 | KSG66\_07165 | PTS-Fru-EIIC, fruA; PTS system, fructose-specific IIC component |
| K02770 | KSG66\_12390 | PTS-Fru-EIIC, fruA; PTS system, fructose-specific IIC component |
| K02777 | KSG66\_10355 | PTS-Glc-EIIA, crr; PTS system, sugar-specific IIA component [EC:2.7.1.-] |
| K02784 | KSG66\_06935 | PTS-HPR.PTSH, ptsH; phosphocarrier protein HPr |
| K02786 | KSG66\_06005 | PTS-Lac-EIIA, lacF; PTS system, lactose-specific IIA component [EC:2.7.1.207] |
| K02788 | KSG66\_06000 | PTS-Lac-EIIC, lacE; PTS system, lactose-specific IIC component |
| K02798 | KSG66\_02110 | PTS-Mtl-EIIA, mtlA, cmtB; PTS system, mannitol-specific IIA component [EC:2.7.1.197] |
| K02800 | KSG66\_02105 | PTS-Mtl-EIIC, mtlA, cmtA; PTS system, mannitol-specific IIC component |
| K02804 | KSG66\_03835 | PTS-Nag-EIIC, nagE; PTS system, N-acetylglucosamine-specific IIC component |
| K02810 | KSG66\_01070 | PTS-Scr-EIIC, scrA, sacP, sacX, ptsS; PTS system, sucrose-specific IIC component |
| K02810 | KSG66\_18000 | PTS-Scr-EIIC, scrA, sacP, sacX, ptsS; PTS system, sucrose-specific IIC component |
| K02819 | KSG66\_03870 | PTS-Tre-EIIC, treB; PTS system, trehalose-specific IIC component |
| K08483 | KSG66\_06940 | PTS-EI.PTSI, ptsI; phosphotransferase system, enzyme I, PtsI [EC:2.7.3.9] |
| K20118 | KSG66\_06930 | PTS-Glc1-EIIC, ptsG, glcA, glcB; PTS system, glucose-specific IIC component |

| 35 | Citrate cycle (TCA cycle) | ko00020 || K00024 | KSG66\_13385 | mdh; malate dehydrogenase [EC:1.1.1.37] |
| K00031 | KSG66\_13390 | IDH1, IDH2, icd; isocitrate dehydrogenase [EC:1.1.1.42] |
| K00161 | KSG66\_07305 | PDHA, pdhA; pyruvate dehydrogenase E1 component alpha subunit [EC:1.2.4.1] |
| K00162 | KSG66\_07310 | PDHB, pdhB; pyruvate dehydrogenase E1 component beta subunit [EC:1.2.4.1] |
| K00164 | KSG66\_09635 | OGDH, sucA; 2-oxoglutarate dehydrogenase E1 component [EC:1.2.4.2] |
| K00239 | KSG66\_13060 | sdhA, frdA; succinate dehydrogenase / fumarate reductase, flavoprotein subunit [EC:1.3.5.1 1.3.5.4] |
| K00240 | KSG66\_13055 | sdhB, frdB; succinate dehydrogenase / fumarate reductase, iron-sulfur subunit [EC:1.3.5.1 1.3.5.4] |
| K00241 | KSG66\_13065 | sdhC, frdC; succinate dehydrogenase / fumarate reductase, cytochrome b subunit |
| K00382 | KSG66\_04065 | DLD, lpd, pdhD; dihydrolipoamide dehydrogenase [EC:1.8.1.4] |
| K00382 | KSG66\_07320 | DLD, lpd, pdhD; dihydrolipoamide dehydrogenase [EC:1.8.1.4] |
| K00382 | KSG66\_11350 | DLD, lpd, pdhD; dihydrolipoamide dehydrogenase [EC:1.8.1.4] |
| K00627 | KSG66\_04060 | DLAT, aceF, pdhC; pyruvate dehydrogenase E2 component (dihydrolipoamide acetyltransferase) [EC:2.3.1.12] |
| K00627 | KSG66\_07315 | DLAT, aceF, pdhC; pyruvate dehydrogenase E2 component (dihydrolipoamide acetyltransferase) [EC:2.3.1.12] |
| K00658 | KSG66\_09630 | DLST, sucB; 2-oxoglutarate dehydrogenase E2 component (dihydrolipoamide succinyltransferase) [EC:2.3.1.61] |
| K01610 | KSG66\_14085 | E4.1.1.49, pckA; phosphoenolpyruvate carboxykinase (ATP) [EC:4.1.1.49] |
| K01647 | KSG66\_04765 | CS, gltA; citrate synthase [EC:2.3.3.1] |
| K01647 | KSG66\_11390 | CS, gltA; citrate synthase [EC:2.3.3.1] |
| K01679 | KSG66\_15415 | E4.2.1.2B, fumC, FH; fumarate hydratase, class II [EC:4.2.1.2] |
| K01681 | KSG66\_08990 | ACO, acnA; aconitate hydratase [EC:4.2.1.3] |
| K01902 | KSG66\_08045 | sucD; succinyl-CoA synthetase alpha subunit [EC:6.2.1.5] |
| K01903 | KSG66\_08040 | sucC; succinyl-CoA synthetase beta subunit [EC:6.2.1.5] |
| K01958 | KSG66\_07440 | PC, pyc; pyruvate carboxylase [EC:6.4.1.1] |

| 36 | Arginine biosynthesis | ko00220 || K00145 | KSG66\_05535 | argC; N-acetyl-gamma-glutamyl-phosphate reductase [EC:1.2.1.38] |
| K00260 | KSG66\_10725 | gudB, rocG; glutamate dehydrogenase [EC:1.4.1.2] |
| K00260 | KSG66\_17855 | gudB, rocG; glutamate dehydrogenase [EC:1.4.1.2] |
| K00491 | KSG66\_03800 | nos; nitric-oxide synthase, bacterial [EC:1.14.14.47] |
| K00611 | KSG66\_05565 | OTC, argF, argI; ornithine carbamoyltransferase [EC:2.1.3.3] |
| K00620 | KSG66\_05540 | argJ; glutamate N-acetyltransferase / amino-acid N-acetyltransferase [EC:2.3.1.35 2.3.1.1] |
| K00812 | KSG66\_10425 | aspB; aspartate aminotransferase [EC:2.6.1.1] |
| K00821 | KSG66\_05550 | argD; acetylornithine/N-succinyldiaminopimelate aminotransferase [EC:2.6.1.11 2.6.1.17] |
| K00930 | KSG66\_05545 | argB; acetylglutamate kinase [EC:2.7.2.8] |
| K01425 | KSG66\_01400 | glsA, GLS; glutaminase [EC:3.5.1.2] |
| K01425 | KSG66\_07425 | glsA, GLS; glutaminase [EC:3.5.1.2] |
| K01428 | KSG66\_17290 | ureC; urease subunit alpha [EC:3.5.1.5] |
| K01429 | KSG66\_17295 | ureB; urease subunit beta [EC:3.5.1.5] |
| K01430 | KSG66\_17300 | ureA; urease subunit gamma [EC:3.5.1.5] |
| K01438 | KSG66\_09840 | argE; acetylornithine deacetylase [EC:3.5.1.16] |
| K01476 | KSG66\_18955 | E3.5.3.1, rocF, arg; arginase [EC:3.5.3.1] |
| K01755 | KSG66\_13505 | argH, ASL; argininosuccinate lyase [EC:4.3.2.1] |
| K01915 | KSG66\_08725 | glnA, GLUL; glutamine synthetase [EC:6.3.1.2] |
| K01940 | KSG66\_13510 | argG, ASS1; argininosuccinate synthase [EC:6.3.4.5] |
| K11358 | KSG66\_04815 | yhdR; aspartate aminotransferase [EC:2.6.1.1] |
| K23265 | KSG66\_03370 |  |

| 37 | Phenylalanine, tyrosine and tryptophan biosynthesis | ko00400 || K00014 | KSG66\_12170 | aroE; shikimate dehydrogenase [EC:1.1.1.25] |
| K00766 | KSG66\_10580 | trpD; anthranilate phosphoribosyltransferase [EC:2.4.2.18] |
| K00800 | KSG66\_10545 | aroA; 3-phosphoshikimate 1-carboxyvinyltransferase [EC:2.5.1.19] |
| K00812 | KSG66\_10425 | aspB; aspartate aminotransferase [EC:2.6.1.1] |
| K00817 | KSG66\_10555 | hisC; histidinol-phosphate aminotransferase [EC:2.6.1.9] |
| K00891 | KSG66\_01710 | E2.7.1.71, aroK, aroL; shikimate kinase [EC:2.7.1.71] |
| K01609 | KSG66\_10575 | trpC; indole-3-glycerol phosphate synthase [EC:4.1.1.48] |
| K01657 | KSG66\_10585 | trpE; anthranilate synthase component I [EC:4.1.3.27] |
| K01695 | KSG66\_10560 | trpA; tryptophan synthase alpha chain [EC:4.2.1.20] |
| K01696 | KSG66\_10565 | trpB; tryptophan synthase beta chain [EC:4.2.1.20] |
| K01735 | KSG66\_10595 | aroB; 3-dehydroquinate synthase [EC:4.2.3.4] |
| K01736 | KSG66\_10600 | aroC; chorismate synthase [EC:4.2.3.5] |
| K01817 | KSG66\_10570 | trpF; phosphoribosylanthranilate isomerase [EC:5.3.1.24] |
| K03785 | KSG66\_03920 | aroD; 3-dehydroquinate dehydratase I [EC:4.2.1.10] |
| K04517 | KSG66\_10550 | tyrA2; prephenate dehydrogenase [EC:1.3.1.12] |
| K04518 | KSG66\_12780 | pheA2; prephenate dehydratase [EC:4.2.1.51] |
| K05887 | KSG66\_03915 | ydiB; quinate/shikimate dehydrogenase [EC:1.1.1.282] |
| K06208 | KSG66\_10590 | aroH; chorismate mutase [EC:5.4.99.5] |
| K06209 | KSG66\_12785 | pheB; chorismate mutase [EC:5.4.99.5] |
| K11358 | KSG66\_04815 | yhdR; aspartate aminotransferase [EC:2.6.1.1] |
| K13853 | KSG66\_13760 | aroG, aroA; 3-deoxy-7-phosphoheptulonate synthase / chorismate mutase [EC:2.5.1.54 5.4.99.5] |

| 38 | Methane metabolism | ko00680 || K00024 | KSG66\_13385 | mdh; malate dehydrogenase [EC:1.1.1.37] |
| K00058 | KSG66\_09435 | serA, PHGDH; D-3-phosphoglycerate dehydrogenase / 2-oxoglutarate reductase [EC:1.1.1.95 1.1.1.399] |
| K00058 | KSG66\_10775 | serA, PHGDH; D-3-phosphoglycerate dehydrogenase / 2-oxoglutarate reductase [EC:1.1.1.95 1.1.1.399] |
| K00121 | KSG66\_01785 | frmA, ADH5, adhC; S-(hydroxymethyl)glutathione dehydrogenase / alcohol dehydrogenase [EC:1.1.1.284 1.1.1.1] |
| K00121 | KSG66\_02865 | frmA, ADH5, adhC; S-(hydroxymethyl)glutathione dehydrogenase / alcohol dehydrogenase [EC:1.1.1.284 1.1.1.1] |
| K00123 | KSG66\_06060 | fdoG, fdhF, fdwA; formate dehydrogenase major subunit [EC:1.17.1.9] |
| K00123 | KSG66\_12455 | fdoG, fdhF, fdwA; formate dehydrogenase major subunit [EC:1.17.1.9] |
| K00600 | KSG66\_17420 | glyA, SHMT; glycine hydroxymethyltransferase [EC:2.1.2.1] |
| K00625 | KSG66\_17785 | E2.3.1.8, pta; phosphate acetyltransferase [EC:2.3.1.8] |
| K00831 | KSG66\_05055 | serC, PSAT1; phosphoserine aminotransferase [EC:2.6.1.52] |
| K00850 | KSG66\_13440 | pfkA, PFK; 6-phosphofructokinase 1 [EC:2.7.1.11] |
| K00925 | KSG66\_13520 | ackA; acetate kinase [EC:2.7.2.1] |
| K01624 | KSG66\_17530 | FBA, fbaA; fructose-bisphosphate aldolase, class II [EC:4.1.2.13] |
| K01689 | KSG66\_15970 | ENO, eno; enolase [EC:4.2.1.11] |
| K01895 | KSG66\_13565 | ACSS, acs; acetyl-CoA synthetase [EC:6.2.1.1] |
| K01895 | KSG66\_13725 | ACSS, acs; acetyl-CoA synthetase [EC:6.2.1.1] |
| K02446 | KSG66\_17515 | glpX; fructose-1,6-bisphosphatase II [EC:3.1.3.11] |
| K04041 | KSG66\_18855 | fbp3; fructose-1,6-bisphosphatase III [EC:3.1.3.11] |
| K08093 | KSG66\_01825 | hxlA; 3-hexulose-6-phosphate synthase [EC:4.1.2.43] |
| K08094 | KSG66\_01820 | hxlB; 6-phospho-3-hexuloisomerase [EC:5.3.1.27] |
| K15633 | KSG66\_15975 | gpmI; 2,3-bisphosphoglycerate-independent phosphoglycerate mutase [EC:5.4.2.12] |

| 39 | Porphyrin and chlorophyll metabolism | ko00860 || K00231 | KSG66\_05110 | PPOX, hemY; protoporphyrinogen/coproporphyrinogen III oxidase [EC:1.3.3.4 1.3.3.15] |
| K00435 | KSG66\_17790 | hemQ; Fe-coproporphyrin III decarboxylase [EC:1.11.1.-] |
| K00798 | KSG66\_15475 | MMAB, pduO; cob(I)alamin adenosyltransferase [EC:2.5.1.17] |
| K01599 | KSG66\_05100 | hemE, UROD; uroporphyrinogen decarboxylase [EC:4.1.1.37] |
| K01698 | KSG66\_12900 | hemB, ALAD; porphobilinogen synthase [EC:4.2.1.24] |
| K01719 | KSG66\_06085 | hemD, UROS; uroporphyrinogen-III synthase [EC:4.2.1.75] |
| K01719 | KSG66\_12905 | hemD, UROS; uroporphyrinogen-III synthase [EC:4.2.1.75] |
| K01749 | KSG66\_12910 | hemC, HMBS; hydroxymethylbilane synthase [EC:2.5.1.61] |
| K01772 | KSG66\_05105 | hemH, FECH; protoporphyrin/coproporphyrin ferrochelatase [EC:4.99.1.1 4.99.1.9] |
| K01845 | KSG66\_04300 | hemL; glutamate-1-semialdehyde 2,1-aminomutase [EC:5.4.3.8] |
| K01845 | KSG66\_12895 | hemL; glutamate-1-semialdehyde 2,1-aminomutase [EC:5.4.3.8] |
| K02257 | KSG66\_04120 | COX10, ctaB, cyoE; heme o synthase [EC:2.5.1.141] |
| K02257 | KSG66\_07450 | COX10, ctaB, cyoE; heme o synthase [EC:2.5.1.141] |
| K02259 | KSG66\_07445 | COX15, ctaA; cytochrome c oxidase assembly protein subunit 15 |
| K02304 | KSG66\_07810 | MET8; precorrin-2 dehydrogenase / sirohydrochlorin ferrochelatase [EC:1.3.1.76 4.99.1.4] |
| K02492 | KSG66\_12920 | hemA; glutamyl-tRNA reductase [EC:1.2.1.70] |
| K02495 | KSG66\_04940 | hemN, hemZ; oxygen-independent coproporphyrinogen III oxidase [EC:1.3.98.3] |
| K02495 | KSG66\_12080 | hemN, hemZ; oxygen-independent coproporphyrinogen III oxidase [EC:1.3.98.3] |
| K03794 | KSG66\_07805 | sirB; sirohydrochlorin ferrochelatase [EC:4.99.1.4] |
| K07145 | KSG66\_03595 | isdG, isdI; heme oxygenase (staphylobilin-producing) [EC:1.14.99.48] |
| K13542 | KSG66\_01750 | cobA-hemD; uroporphyrinogen III methyltransferase / synthase [EC:2.1.1.107 4.2.1.75] |

| 40 | Bacterial chemotaxis | ko02030 || K00575 | KSG66\_10605 | cheR; chemotaxis protein methyltransferase CheR [EC:2.1.1.80] |
| K02410 | KSG66\_08105 | fliG; flagellar motor switch protein FliG |
| K02416 | KSG66\_08155 | fliM; flagellar motor switch protein FliM |
| K02417 | KSG66\_08160 | fliNY, fliN; flagellar motor switch protein FliN/FliY |
| K02556 | KSG66\_06820 | motA; chemotaxis protein MotA |
| K02556 | KSG66\_13750 | motA; chemotaxis protein MotA |
| K02557 | KSG66\_06815 | motB; chemotaxis protein MotB |
| K03406 | KSG66\_06955 | mcp; methyl-accepting chemotaxis protein |
| K03406 | KSG66\_14435 | mcp; methyl-accepting chemotaxis protein |
| K03406 | KSG66\_14440 | mcp; methyl-accepting chemotaxis protein |
| K03406 | KSG66\_14445 | mcp; methyl-accepting chemotaxis protein |
| K03406 | KSG66\_14450 | mcp; methyl-accepting chemotaxis protein |
| K03407 | KSG66\_08215 | cheA; two-component system, chemotaxis family, sensor kinase CheA [EC:2.7.13.3] |
| K03408 | KSG66\_08220 | cheW; purine-binding chemotaxis protein CheW |
| K03410 | KSG66\_08225 | cheC; chemotaxis protein CheC |
| K03411 | KSG66\_08230 | cheD; chemotaxis protein CheD [EC:3.5.1.44] |
| K03412 | KSG66\_08210 | cheB; two-component system, chemotaxis family, protein-glutamate methylesterase/glutaminase [EC:3.1.1.61 3.5.1.44] |
| K03413 | KSG66\_08165 | cheY; two-component system, chemotaxis family, chemotaxis protein CheY |
| K03413 | KSG66\_08960 | cheY; two-component system, chemotaxis family, chemotaxis protein CheY |
| K03415 | KSG66\_06990 | cheV; two-component system, chemotaxis family, chemotaxis protein CheV |
| K10439 | KSG66\_16960 | rbsB; ribose transport system substrate-binding protein |

| 41 | Galactose metabolism | ko00052 || K00845 | KSG66\_11770 | glk; glucokinase [EC:2.7.1.2] |
| K00849 | KSG66\_05995 | galK; galactokinase [EC:2.7.1.6] |
| K00849 | KSG66\_18075 | galK; galactokinase [EC:2.7.1.6] |
| K00850 | KSG66\_13440 | pfkA, PFK; 6-phosphofructokinase 1 [EC:2.7.1.11] |
| K00963 | KSG66\_16805 | UGP2, galU, galF; UTP--glucose-1-phosphate uridylyltransferase [EC:2.7.7.9] |
| K00965 | KSG66\_05985 | galT, GALT; UDPglucose--hexose-1-phosphate uridylyltransferase [EC:2.7.7.12] |
| K00965 | KSG66\_18070 | galT, GALT; UDPglucose--hexose-1-phosphate uridylyltransferase [EC:2.7.7.12] |
| K01182 | KSG66\_01555 | IMA, malL; oligo-1,6-glucosidase [EC:3.2.1.10] |
| K01182 | KSG66\_16290 | IMA, malL; oligo-1,6-glucosidase [EC:3.2.1.10] |
| K01187 | KSG66\_14475 | malZ; alpha-glucosidase [EC:3.2.1.20] |
| K01193 | KSG66\_16265 | INV, sacA; beta-fructofuranosidase [EC:3.2.1.26] |
| K01193 | KSG66\_17995 | INV, sacA; beta-fructofuranosidase [EC:3.2.1.26] |
| K01220 | KSG66\_06010 | E3.2.1.85, lacG; 6-phospho-beta-galactosidase [EC:3.2.1.85] |
| K01784 | KSG66\_05990 | galE, GALE; UDP-glucose 4-epimerase [EC:5.1.3.2] |
| K01784 | KSG66\_18420 | galE, GALE; UDP-glucose 4-epimerase [EC:5.1.3.2] |
| K01785 | KSG66\_09320 | galM, GALM; aldose 1-epimerase [EC:5.1.3.3] |
| K01835 | KSG66\_04700 | pgm; phosphoglucomutase [EC:5.4.2.2] |
| K02786 | KSG66\_06005 | PTS-Lac-EIIA, lacF; PTS system, lactose-specific IIA component [EC:2.7.1.207] |
| K02788 | KSG66\_06000 | PTS-Lac-EIIC, lacE; PTS system, lactose-specific IIC component |
| K07406 | KSG66\_13935 | melA; alpha-galactosidase [EC:3.2.1.22] |

| 42 | Glycerophospholipid metabolism | ko00564 || K00057 | KSG66\_10660 | gpsA; glycerol-3-phosphate dehydrogenase (NAD(P)+) [EC:1.1.1.94] |
| K00096 | KSG66\_13215 | araM, egsA; glycerol-1-phosphate dehydrogenase [NAD(P)+] [EC:1.1.1.261] |
| K00111 | KSG66\_04695 | glpA, glpD; glycerol-3-phosphate dehydrogenase [EC:1.1.5.3] |
| K00655 | KSG66\_04800 | plsC; 1-acyl-sn-glycerol-3-phosphate acyltransferase [EC:2.3.1.51] |
| K00655 | KSG66\_10120 | plsC; 1-acyl-sn-glycerol-3-phosphate acyltransferase [EC:2.3.1.51] |
| K00980 | KSG66\_16830 | tagD; glycerol-3-phosphate cytidylyltransferase [EC:2.7.7.39] |
| K00981 | KSG66\_08270 | E2.7.7.41, CDS1, CDS2, cdsA; phosphatidate cytidylyltransferase [EC:2.7.7.41] |
| K00995 | KSG66\_08460 | pgsA, PGS1; CDP-diacylglycerol---glycerol-3-phosphate 3-phosphatidyltransferase [EC:2.7.8.5] |
| K01048 | KSG66\_14060 | pldB; lysophospholipase [EC:3.1.1.5] |
| K01126 | KSG66\_01285 | E3.1.4.46, glpQ, ugpQ; glycerophosphoryl diester phosphodiesterase [EC:3.1.4.46] |
| K01126 | KSG66\_04840 | E3.1.4.46, glpQ, ugpQ; glycerophosphoryl diester phosphodiesterase [EC:3.1.4.46] |
| K01126 | KSG66\_11410 | E3.1.4.46, glpQ, ugpQ; glycerophosphoryl diester phosphodiesterase [EC:3.1.4.46] |
| K01126 | KSG66\_14050 | E3.1.4.46, glpQ, ugpQ; glycerophosphoryl diester phosphodiesterase [EC:3.1.4.46] |
| K01613 | KSG66\_01360 | psd, PISD; phosphatidylserine decarboxylase [EC:4.1.1.65] |
| K06131 | KSG66\_17265 | clsA\_B; cardiolipin synthase A/B [EC:2.7.8.-] |
| K06131 | KSG66\_17560 | clsA\_B; cardiolipin synthase A/B [EC:2.7.8.-] |
| K06131 | KSG66\_17580 | clsA\_B; cardiolipin synthase A/B [EC:2.7.8.-] |
| K07029 | KSG66\_03490 | dagK; diacylglycerol kinase (ATP) [EC:2.7.1.107] |
| K08591 | KSG66\_09030 | plsY; acyl phosphate:glycerol-3-phosphate acyltransferase [EC:2.3.1.275] |
| K17103 | KSG66\_01350 | CHO1, pssA; CDP-diacylglycerol---serine O-phosphatidyltransferase [EC:2.7.8.8] |

| 43 | Pantothenate and CoA biosynthesis | ko00770 || K00053 | KSG66\_12980 | ilvC; ketol-acid reductoisomerase [EC:1.1.1.86] |
| K00077 | KSG66\_07185 | panE, apbA; 2-dehydropantoate 2-reductase [EC:1.1.1.169] |
| K00077 | KSG66\_07565 | panE, apbA; 2-dehydropantoate 2-reductase [EC:1.1.1.169] |
| K00077 | KSG66\_15355 | panE, apbA; 2-dehydropantoate 2-reductase [EC:1.1.1.169] |
| K00606 | KSG66\_10455 | panB; 3-methyl-2-oxobutanoate hydroxymethyltransferase [EC:2.1.2.11] |
| K00826 | KSG66\_01380 | E2.6.1.42, ilvE; branched-chain amino acid aminotransferase [EC:2.6.1.42] |
| K00826 | KSG66\_18220 | E2.6.1.42, ilvE; branched-chain amino acid aminotransferase [EC:2.6.1.42] |
| K00859 | KSG66\_13355 | coaE; dephospho-CoA kinase [EC:2.7.1.24] |
| K00867 | KSG66\_11120 | coaA; type I pantothenate kinase [EC:2.7.1.33] |
| K00954 | KSG66\_07520 | E2.7.7.3A, coaD, kdtB; pantetheine-phosphate adenylyltransferase [EC:2.7.7.3] |
| K00997 | KSG66\_02465 | acpS; holo-[acyl-carrier protein] synthase [EC:2.7.8.7] |
| K01579 | KSG66\_10445 | panD; aspartate 1-decarboxylase [EC:4.1.1.11] |
| K01652 | KSG66\_12990 | E2.2.1.6L, ilvB, ilvG, ilvI; acetolactate synthase I/II/III large subunit [EC:2.2.1.6] |
| K01652 | KSG66\_16980 | E2.2.1.6L, ilvB, ilvG, ilvI; acetolactate synthase I/II/III large subunit [EC:2.2.1.6] |
| K01653 | KSG66\_12985 | E2.2.1.6S, ilvH, ilvN; acetolactate synthase I/III small subunit [EC:2.2.1.6] |
| K01687 | KSG66\_10150 | ilvD; dihydroxy-acid dehydratase [EC:4.2.1.9] |
| K01918 | KSG66\_10450 | panC; pantoate--beta-alanine ligase [EC:6.3.2.1] |
| K03525 | KSG66\_00425 | coaX; type III pantothenate kinase [EC:2.7.1.33] |
| K06133 | KSG66\_01860 | LYS5, acpT; 4'-phosphopantetheinyl transferase [EC:2.7.8.-] |
| K13038 | KSG66\_07845 | coaBC, dfp; phosphopantothenoylcysteine decarboxylase / phosphopantothenate---cysteine ligase [EC:4.1.1.36 6.3.2.5] |

| 44 | Fructose and mannose metabolism | ko00051 || K00008 | KSG66\_03200 | SORD, gutB; L-iditol 2-dehydrogenase [EC:1.1.1.14] |
| K00009 | KSG66\_02115 | mtlD; mannitol-1-phosphate 5-dehydrogenase [EC:1.1.1.17] |
| K00847 | KSG66\_03210 | E2.7.1.4, scrK; fructokinase [EC:2.7.1.4] |
| K00850 | KSG66\_13440 | pfkA, PFK; 6-phosphofructokinase 1 [EC:2.7.1.11] |
| K00882 | KSG66\_07160 | fruK; 1-phosphofructokinase [EC:2.7.1.56] |
| K01218 | KSG66\_18310 | gmuG; mannan endo-1,4-beta-mannosidase [EC:3.2.1.78] |
| K01624 | KSG66\_17530 | FBA, fbaA; fructose-bisphosphate aldolase, class II [EC:4.1.2.13] |
| K01803 | KSG66\_15980 | TPI, tpiA; triosephosphate isomerase (TIM) [EC:5.3.1.1] |
| K01805 | KSG66\_08780 | xylA; xylose isomerase [EC:5.3.1.5] |
| K01808 | KSG66\_17430 | rpiB; ribose 5-phosphate isomerase B [EC:5.3.1.6] |
| K01809 | KSG66\_12385 | manA, MPI; mannose-6-phosphate isomerase [EC:5.3.1.8] |
| K01809 | KSG66\_16850 | manA, MPI; mannose-6-phosphate isomerase [EC:5.3.1.8] |
| K01809 | KSG66\_18305 | manA, MPI; mannose-6-phosphate isomerase [EC:5.3.1.8] |
| K02446 | KSG66\_17515 | glpX; fructose-1,6-bisphosphatase II [EC:3.1.3.11] |
| K02770 | KSG66\_07165 | PTS-Fru-EIIC, fruA; PTS system, fructose-specific IIC component |
| K02770 | KSG66\_12390 | PTS-Fru-EIIC, fruA; PTS system, fructose-specific IIC component |
| K02798 | KSG66\_02110 | PTS-Mtl-EIIA, mtlA, cmtB; PTS system, mannitol-specific IIA component [EC:2.7.1.197] |
| K02800 | KSG66\_02105 | PTS-Mtl-EIIC, mtlA, cmtA; PTS system, mannitol-specific IIC component |
| K04041 | KSG66\_18855 | fbp3; fructose-1,6-bisphosphatase III [EC:3.1.3.11] |

| 45 | Thiamine metabolism | ko00730 || K00788 | KSG66\_18120 | thiE; thiamine-phosphate pyrophosphorylase [EC:2.5.1.3] |
| K00878 | KSG66\_18125 | thiM; hydroxyethylthiazole kinase [EC:2.7.1.50] |
| K00939 | KSG66\_00835 | adk, AK; adenylate kinase [EC:2.7.4.3] |
| K00941 | KSG66\_05805 | thiD; hydroxymethylpyrimidine/phosphomethylpyrimidine kinase [EC:2.7.1.49 2.7.4.7] |
| K00946 | KSG66\_03100 | thiL; thiamine-monophosphate kinase [EC:2.7.4.16] |
| K00949 | KSG66\_07895 | thiN, TPK1, THI80; thiamine pyrophosphokinase [EC:2.7.6.2] |
| K01077 | KSG66\_04750 | E3.1.3.1, phoA, phoB; alkaline phosphatase [EC:3.1.3.1] |
| K01662 | KSG66\_11485 | dxs; 1-deoxy-D-xylulose-5-phosphate synthase [EC:2.2.1.7] |
| K03147 | KSG66\_04430 | thiC; phosphomethylpyrimidine synthase [EC:4.1.99.17] |
| K03148 | KSG66\_05800 | thiF; sulfur carrier protein ThiS adenylyltransferase [EC:2.7.7.73] |
| K03149 | KSG66\_05795 | thiG; thiazole synthase [EC:2.8.1.10] |
| K03151 | KSG66\_13575 | thiI; tRNA uracil 4-sulfurtransferase [EC:2.8.1.4] |
| K03153 | KSG66\_05785 | thiO; glycine oxidase [EC:1.4.3.19] |
| K03707 | KSG66\_05775 | tenA; thiaminase (transcriptional activator TenA) [EC:3.5.99.2] |
| K04487 | KSG66\_12610 | iscS, NFS1; cysteine desulfurase [EC:2.8.1.7] |
| K04487 | KSG66\_12770 | iscS, NFS1; cysteine desulfurase [EC:2.8.1.7] |
| K06949 | KSG66\_07885 | rsgA, engC; ribosome biogenesis GTPase / thiamine phosphate phosphatase [EC:3.6.1.- 3.1.3.100] |
| K10810 | KSG66\_05780 | tenI; thiazole tautomerase (transcriptional regulator TenI) [EC:5.3.99.10] |
| K20895 | KSG66\_07670 | ylmB; formylaminopyrimidine deformylase [EC:3.5.1.-] |

| 46 | Biotin metabolism | ko00780 || K00059 | KSG66\_07950 | fabG; 3-oxoacyl-[acyl-carrier protein] reductase [EC:1.1.1.100] |
| K00059 | KSG66\_08440 | fabG; 3-oxoacyl-[acyl-carrier protein] reductase [EC:1.1.1.100] |
| K00059 | KSG66\_09410 | fabG; 3-oxoacyl-[acyl-carrier protein] reductase [EC:1.1.1.100] |
| K00059 | KSG66\_11195 | fabG; 3-oxoacyl-[acyl-carrier protein] reductase [EC:1.1.1.100] |
| K00059 | KSG66\_13500 | fabG; 3-oxoacyl-[acyl-carrier protein] reductase [EC:1.1.1.100] |
| K00059 | KSG66\_15330 | fabG; 3-oxoacyl-[acyl-carrier protein] reductase [EC:1.1.1.100] |
| K00059 | KSG66\_15750 | fabG; 3-oxoacyl-[acyl-carrier protein] reductase [EC:1.1.1.100] |
| K00208 | KSG66\_05810 | fabI; enoyl-[acyl-carrier protein] reductase I [EC:1.3.1.9 1.3.1.10] |
| K00652 | KSG66\_09215 | bioF; 8-amino-7-oxononanoate synthase [EC:2.3.1.47] |
| K01012 | KSG66\_09205 | bioB; biotin synthase [EC:2.8.1.6] |
| K01906 | KSG66\_09225 | bioW; 6-carboxyhexanoate--CoA ligase [EC:6.2.1.14] |
| K01935 | KSG66\_09210 | bioD; dethiobiotin synthetase [EC:6.3.3.3] |
| K02372 | KSG66\_02120 | fabZ; 3-hydroxyacyl-[acyl-carrier-protein] dehydratase [EC:4.2.1.59] |
| K02372 | KSG66\_17165 | fabZ; 3-hydroxyacyl-[acyl-carrier-protein] dehydratase [EC:4.2.1.59] |
| K03524 | KSG66\_10460 | birA; BirA family transcriptional regulator, biotin operon repressor / biotin---[acetyl-CoA-carboxylase] ligase [EC:6.3.4.15] |
| K09458 | KSG66\_02925 | fabF; 3-oxoacyl-[acyl-carrier-protein] synthase II [EC:2.3.1.179] |
| K09458 | KSG66\_05610 | fabF; 3-oxoacyl-[acyl-carrier-protein] synthase II [EC:2.3.1.179] |
| K16593 | KSG66\_09200 | bioI, CYP107H; pimeloyl-[acyl-carrier protein] synthase [EC:1.14.14.46] |
| K19563 | KSG66\_09220 | bioA, bioK; lysine---8-amino-7-oxononanoate aminotransferase [EC:2.6.1.105] |

| 47 | Protein export | ko03060 || K03070 | KSG66\_16620 | secA; preprotein translocase subunit SecA |
| K03073 | KSG66\_00650 | secE; preprotein translocase subunit SecE |
| K03075 | KSG66\_15800 | secG; preprotein translocase subunit SecG |
| K03076 | KSG66\_00830 | secY; preprotein translocase subunit SecY |
| K03100 | KSG66\_05280 | lepB; signal peptidase I [EC:3.4.21.89] |
| K03100 | KSG66\_07170 | lepB; signal peptidase I [EC:3.4.21.89] |
| K03100 | KSG66\_10890 | lepB; signal peptidase I [EC:3.4.21.89] |
| K03101 | KSG66\_07720 | lspA; signal peptidase II [EC:3.4.23.36] |
| K03106 | KSG66\_07985 | SRP54, ffh; signal recognition particle subunit SRP54 [EC:3.6.5.4] |
| K03110 | KSG66\_07970 | ftsY; fused signal recognition particle receptor |
| K03116 | KSG66\_01475 | tatA; sec-independent protein translocase protein TatA |
| K03116 | KSG66\_03140 | tatA; sec-independent protein translocase protein TatA |
| K03118 | KSG66\_01480 | tatC; sec-independent protein translocase protein TatC |
| K03118 | KSG66\_03145 | tatC; sec-independent protein translocase protein TatC |
| K03210 | KSG66\_12710 | yajC; preprotein translocase subunit YajC |
| K03217 | KSG66\_11275 | yidC, spoIIIJ, OXA1, ccfA; YidC/Oxa1 family membrane protein insertase |
| K03217 | KSG66\_19405 | yidC, spoIIIJ, OXA1, ccfA; YidC/Oxa1 family membrane protein insertase |
| K12257 | KSG66\_12685 | secDF; SecD/SecF fusion protein |
| K13280 | KSG66\_11660 | SEC11, sipW; signal peptidase I [EC:3.4.21.89] |

| 48 | Homologous recombination | ko03440 || K02335 | KSG66\_13370 | polA; DNA polymerase I [EC:2.7.7.7] |
| K02337 | KSG66\_13460 | dnaE; DNA polymerase III subunit alpha [EC:2.7.7.7] |
| K02338 | KSG66\_00010 | dnaN; DNA polymerase III subunit beta [EC:2.7.7.7] |
| K02340 | KSG66\_12110 | holA; DNA polymerase III subunit delta [EC:2.7.7.7] |
| K02341 | KSG66\_00220 | holB; DNA polymerase III subunit delta' [EC:2.7.7.7] |
| K02343 | KSG66\_00135 | dnaX; DNA polymerase III subunit gamma/tau [EC:2.7.7.7] |
| K03111 | KSG66\_17145 | ssb; single-strand DNA-binding protein |
| K03111 | KSG66\_19330 | ssb; single-strand DNA-binding protein |
| K03550 | KSG66\_12735 | ruvA; holliday junction DNA helicase RuvA [EC:3.6.4.12] |
| K03551 | KSG66\_12730 | ruvB; holliday junction DNA helicase RuvB [EC:3.6.4.12] |
| K03553 | KSG66\_08470 | recA; recombination protein RecA |
| K03581 | KSG66\_12595 | recD; exodeoxyribonuclease V alpha subunit [EC:3.1.11.5] |
| K03584 | KSG66\_11965 | recO; DNA repair protein RecO (recombination protein O) |
| K03629 | KSG66\_00020 | recF; DNA replication and repair protein RecF |
| K03655 | KSG66\_07930 | recG; ATP-dependent DNA helicase RecG [EC:3.6.4.12] |
| K03763 | KSG66\_08290 | polC; DNA polymerase III subunit alpha, Gram-positive type [EC:2.7.7.7] |
| K04066 | KSG66\_07850 | priA; primosomal protein N' (replication factor Y) (superfamily II helicase) [EC:3.6.4.-] |
| K06187 | KSG66\_00145 | recR; recombination protein RecR |
| K07462 | KSG66\_12670 | recJ; single-stranded-DNA-specific exonuclease [EC:3.1.-.-] |

| 49 | Nitrogen metabolism | ko00910 || K00260 | KSG66\_10725 | gudB, rocG; glutamate dehydrogenase [EC:1.4.1.2] |
| K00260 | KSG66\_17855 | gudB, rocG; glutamate dehydrogenase [EC:1.4.1.2] |
| K00265 | KSG66\_09385 | gltB; glutamate synthase (NADPH) large chain [EC:1.4.1.13] |
| K00266 | KSG66\_09380 | gltD; glutamate synthase (NADPH) small chain [EC:1.4.1.13] |
| K00362 | KSG66\_01760 | nirB; nitrite reductase (NADH) large subunit [EC:1.7.1.15] |
| K00362 | KSG66\_01770 | nirB; nitrite reductase (NADH) large subunit [EC:1.7.1.15] |
| K00363 | KSG66\_01755 | nirD; nitrite reductase (NADH) small subunit [EC:1.7.1.15] |
| K00370 | KSG66\_17605 | narG, narZ, nxrA; nitrate reductase / nitrite oxidoreductase, alpha subunit [EC:1.7.5.1 1.7.99.-] |
| K00371 | KSG66\_17600 | narH, narY, nxrB; nitrate reductase / nitrite oxidoreductase, beta subunit [EC:1.7.5.1 1.7.99.-] |
| K00372 | KSG66\_01765 | nasA; assimilatory nitrate reductase catalytic subunit [EC:1.7.99.-] |
| K00374 | KSG66\_17590 | narI, narV; nitrate reductase gamma subunit [EC:1.7.5.1 1.7.99.-] |
| K00459 | KSG66\_14465 | ncd2, npd; nitronate monooxygenase [EC:1.13.12.16] |
| K01673 | KSG66\_14150 | cynT, can; carbonic anhydrase [EC:4.2.1.1] |
| K01673 | KSG66\_16345 | cynT, can; carbonic anhydrase [EC:4.2.1.1] |
| K01743 | KSG66\_08820 | E4.2.1.1; carbonic anhydrase [EC:4.2.1.1] |
| K01915 | KSG66\_08725 | glnA, GLUL; glutamine synthetase [EC:6.3.1.2] |
| K02575 | KSG66\_01775 | NRT, narK, nrtP, nasA; MFS transporter, NNP family, nitrate/nitrite transporter |
| K02575 | KSG66\_17625 | NRT, narK, nrtP, nasA; MFS transporter, NNP family, nitrate/nitrite transporter |

| 50 | Mismatch repair | ko03430 || K01972 | KSG66\_03440 | E6.5.1.2, ligA, ligB; DNA ligase (NAD+) [EC:6.5.1.2] |
| K02337 | KSG66\_13460 | dnaE; DNA polymerase III subunit alpha [EC:2.7.7.7] |
| K02338 | KSG66\_00010 | dnaN; DNA polymerase III subunit beta [EC:2.7.7.7] |
| K02340 | KSG66\_12110 | holA; DNA polymerase III subunit delta [EC:2.7.7.7] |
| K02341 | KSG66\_00220 | holB; DNA polymerase III subunit delta' [EC:2.7.7.7] |
| K02343 | KSG66\_00135 | dnaX; DNA polymerase III subunit gamma/tau [EC:2.7.7.7] |
| K03111 | KSG66\_17145 | ssb; single-strand DNA-binding protein |
| K03111 | KSG66\_19330 | ssb; single-strand DNA-binding protein |
| K03555 | KSG66\_08520 | mutS; DNA mismatch repair protein MutS |
| K03572 | KSG66\_08525 | mutL; DNA mismatch repair protein MutL |
| K03601 | KSG66\_11500 | xseA; exodeoxyribonuclease VII large subunit [EC:3.1.11.6] |
| K03602 | KSG66\_11495 | xseB; exodeoxyribonuclease VII small subunit [EC:3.1.11.6] |
| K03657 | KSG66\_03435 | uvrD, pcrA; DNA helicase II / ATP-dependent DNA helicase PcrA [EC:3.6.4.12] |
| K03657 | KSG66\_05865 | uvrD, pcrA; DNA helicase II / ATP-dependent DNA helicase PcrA [EC:3.6.4.12] |
| K03657 | KSG66\_15645 | uvrD, pcrA; DNA helicase II / ATP-dependent DNA helicase PcrA [EC:3.6.4.12] |
| K03763 | KSG66\_08290 | polC; DNA polymerase III subunit alpha, Gram-positive type [EC:2.7.7.7] |
| K07456 | KSG66\_13130 | mutS2; DNA mismatch repair protein MutS2 |
| K07462 | KSG66\_12670 | recJ; single-stranded-DNA-specific exonuclease [EC:3.1.-.-] |

| 51 | Pentose and glucuronate interconversions | ko00040 || K00008 | KSG66\_03200 | SORD, gutB; L-iditol 2-dehydrogenase [EC:1.1.1.14] |
| K00012 | KSG66\_16760 | UGDH, ugd; UDPglucose 6-dehydrogenase [EC:1.1.1.22] |
| K00012 | KSG66\_17100 | UGDH, ugd; UDPglucose 6-dehydrogenase [EC:1.1.1.22] |
| K00041 | KSG66\_06135 | uxaB; tagaturonate reductase [EC:1.1.1.58] |
| K00853 | KSG66\_13230 | araB; L-ribulokinase [EC:2.7.1.16] |
| K00854 | KSG66\_08785 | xylB, XYLB; xylulokinase [EC:2.7.1.17] |
| K00963 | KSG66\_16805 | UGP2, galU, galF; UTP--glucose-1-phosphate uridylyltransferase [EC:2.7.7.9] |
| K01685 | KSG66\_06140 | uxaA; altronate hydrolase [EC:4.2.1.7] |
| K01686 | KSG66\_09075 | uxuA; mannonate dehydratase [EC:4.2.1.8] |
| K01728 | KSG66\_03750 | pel; pectate lyase [EC:4.2.2.2] |
| K01783 | KSG66\_07890 | rpe, RPE; ribulose-phosphate 3-epimerase [EC:5.1.3.1] |
| K01804 | KSG66\_13235 | araA; L-arabinose isomerase [EC:5.3.1.4] |
| K01805 | KSG66\_08780 | xylA; xylose isomerase [EC:5.3.1.5] |
| K01812 | KSG66\_06120 | uxaC; glucuronate isomerase [EC:5.3.1.12] |
| K03077 | KSG66\_13225 | araD, ulaF, sgaE, sgbE; L-ribulose-5-phosphate 4-epimerase [EC:5.1.3.4] |
| K09988 | KSG66\_02205 | lyxA; D-lyxose ketol-isomerase [EC:5.3.1.15] |
| K14274 | KSG66\_15500 | xylC; xylonolactonase [EC:3.1.1.-] |

| 52 | Fatty acid degradation | ko00071 || K00121 | KSG66\_01785 | frmA, ADH5, adhC; S-(hydroxymethyl)glutathione dehydrogenase / alcohol dehydrogenase [EC:1.1.1.284 1.1.1.1] |
| K00121 | KSG66\_02865 | frmA, ADH5, adhC; S-(hydroxymethyl)glutathione dehydrogenase / alcohol dehydrogenase [EC:1.1.1.284 1.1.1.1] |
| K00128 | KSG66\_09600 | ALDH; aldehyde dehydrogenase (NAD+) [EC:1.2.1.3] |
| K00128 | KSG66\_13620 | ALDH; aldehyde dehydrogenase (NAD+) [EC:1.2.1.3] |
| K00128 | KSG66\_18800 | ALDH; aldehyde dehydrogenase (NAD+) [EC:1.2.1.3] |
| K00626 | KSG66\_05195 | E2.3.1.9, atoB; acetyl-CoA C-acetyltransferase [EC:2.3.1.9] |
| K00626 | KSG66\_11405 | E2.3.1.9, atoB; acetyl-CoA C-acetyltransferase [EC:2.3.1.9] |
| K00632 | KSG66\_15285 | fadA, fadI; acetyl-CoA acyltransferase [EC:2.3.1.16] |
| K01897 | KSG66\_02195 | ACSL, fadD; long-chain acyl-CoA synthetase [EC:6.2.1.3] |
| K01897 | KSG66\_05165 | ACSL, fadD; long-chain acyl-CoA synthetase [EC:6.2.1.3] |
| K01897 | KSG66\_05200 | ACSL, fadD; long-chain acyl-CoA synthetase [EC:6.2.1.3] |
| K01897 | KSG66\_13115 | ACSL, fadD; long-chain acyl-CoA synthetase [EC:6.2.1.3] |
| K07516 | KSG66\_15290 | fadN; 3-hydroxyacyl-CoA dehydrogenase [EC:1.1.1.35] |
| K13767 | KSG66\_13105 | fadB; enoyl-CoA hydratase [EC:4.2.1.17] |
| K13953 | KSG66\_08815 | adhP; alcohol dehydrogenase, propanol-preferring [EC:1.1.1.1] |
| K14338 | KSG66\_03640 | cypD\_E, CYP102A2\_3; cytochrome P450 / NADPH-cytochrome P450 reductase [EC:1.14.14.1 1.6.2.4] |
| K14338 | KSG66\_12420 | cypD\_E, CYP102A2\_3; cytochrome P450 / NADPH-cytochrome P450 reductase [EC:1.14.14.1 1.6.2.4] |

| 53 | Lysine biosynthesis | ko00300 || K00133 | KSG66\_08375 | asd; aspartate-semialdehyde dehydrogenase [EC:1.2.1.11] |
| K00215 | KSG66\_10485 | dapB; 4-hydroxy-tetrahydrodipicolinate reductase [EC:1.17.1.8] |
| K00821 | KSG66\_05550 | argD; acetylornithine/N-succinyldiaminopimelate aminotransferase [EC:2.6.1.11 2.6.1.17] |
| K00841 | KSG66\_06980 | patA; aminotransferase [EC:2.6.1.-] |
| K00928 | KSG66\_02010 | lysC; aspartate kinase [EC:2.7.2.4] |
| K00928 | KSG66\_08380 | lysC; aspartate kinase [EC:2.7.2.4] |
| K00928 | KSG66\_13075 | lysC; aspartate kinase [EC:2.7.2.4] |
| K01439 | KSG66\_13865 | dapE; succinyl-diaminopimelate desuccinylase [EC:3.5.1.18] |
| K01586 | KSG66\_10910 | lysA; diaminopimelate decarboxylase [EC:4.1.1.20] |
| K01714 | KSG66\_02785 | dapA; 4-hydroxy-tetrahydrodipicolinate synthase [EC:4.3.3.7] |
| K01714 | KSG66\_08385 | dapA; 4-hydroxy-tetrahydrodipicolinate synthase [EC:4.3.3.7] |
| K01778 | KSG66\_14955 | dapF; diaminopimelate epimerase [EC:5.1.1.7] |
| K01928 | KSG66\_07600 | murE; UDP-N-acetylmuramoyl-L-alanyl-D-glutamate--2,6-diaminopimelate ligase [EC:6.3.2.13] |
| K01929 | KSG66\_02440 | murF; UDP-N-acetylmuramoyl-tripeptide--D-alanyl-D-alanine ligase [EC:6.3.2.10] |
| K05822 | KSG66\_07070 | dapH, dapD; tetrahydrodipicolinate N-acetyltransferase [EC:2.3.1.89] |
| K05823 | KSG66\_07075 | dapL; N-acetyldiaminopimelate deacetylase [EC:3.5.1.47] |
| K05825 | KSG66\_16095 | LYSN; 2-aminoadipate transaminase [EC:2.6.1.-] |

| 54 | Histidine metabolism | ko00340 || K00013 | KSG66\_16445 | hisD; histidinol dehydrogenase [EC:1.1.1.23] |
| K00128 | KSG66\_09600 | ALDH; aldehyde dehydrogenase (NAD+) [EC:1.2.1.3] |
| K00128 | KSG66\_13620 | ALDH; aldehyde dehydrogenase (NAD+) [EC:1.2.1.3] |
| K00128 | KSG66\_18800 | ALDH; aldehyde dehydrogenase (NAD+) [EC:1.2.1.3] |
| K00765 | KSG66\_16450 | hisG; ATP phosphoribosyltransferase [EC:2.4.2.17] |
| K00817 | KSG66\_10555 | hisC; histidinol-phosphate aminotransferase [EC:2.6.1.9] |
| K01468 | KSG66\_18585 | hutI, AMDHD1; imidazolonepropionase [EC:3.5.2.7] |
| K01479 | KSG66\_18590 | hutG; formiminoglutamase [EC:3.5.3.8] |
| K01693 | KSG66\_16440 | hisB; imidazoleglycerol-phosphate dehydratase [EC:4.2.1.19] |
| K01712 | KSG66\_18580 | hutU, UROC1; urocanate hydratase [EC:4.2.1.49] |
| K01745 | KSG66\_18575 | hutH, HAL; histidine ammonia-lyase [EC:4.3.1.3] |
| K01814 | KSG66\_16430 | hisA; phosphoribosylformimino-5-aminoimidazole carboxamide ribotide isomerase [EC:5.3.1.16] |
| K02500 | KSG66\_16425 | hisF; imidazole glycerol-phosphate synthase subunit HisF [EC:4.3.2.10] |
| K02501 | KSG66\_16435 | hisH; imidazole glycerol-phosphate synthase subunit HisH [EC:4.3.2.10] |
| K02502 | KSG66\_16455 | hisZ; ATP phosphoribosyltransferase regulatory subunit |
| K04486 | KSG66\_13595 | E3.1.3.15B; histidinol-phosphatase (PHP family) [EC:3.1.3.15] |
| K11755 | KSG66\_16420 | hisIE; phosphoribosyl-ATP pyrophosphohydrolase / phosphoribosyl-AMP cyclohydrolase [EC:3.6.1.31 3.5.4.19] |

| 55 | Glycerolipid metabolism | ko00561 || K00005 | KSG66\_02915 | gldA; glycerol dehydrogenase [EC:1.1.1.6] |
| K00128 | KSG66\_09600 | ALDH; aldehyde dehydrogenase (NAD+) [EC:1.2.1.3] |
| K00128 | KSG66\_13620 | ALDH; aldehyde dehydrogenase (NAD+) [EC:1.2.1.3] |
| K00128 | KSG66\_18800 | ALDH; aldehyde dehydrogenase (NAD+) [EC:1.2.1.3] |
| K00655 | KSG66\_04800 | plsC; 1-acyl-sn-glycerol-3-phosphate acyltransferase [EC:2.3.1.51] |
| K00655 | KSG66\_10120 | plsC; 1-acyl-sn-glycerol-3-phosphate acyltransferase [EC:2.3.1.51] |
| K00864 | KSG66\_04690 | glpK, GK; glycerol kinase [EC:2.7.1.30] |
| K00865 | KSG66\_00095 | glxK, garK; glycerate 2-kinase [EC:2.7.1.165] |
| K01046 | KSG66\_01510 | lip, TGL2; triacylglycerol lipase [EC:3.1.1.3] |
| K03429 | KSG66\_10175 | ugtP; processive 1,2-diacylglycerol beta-glucosyltransferase [EC:2.4.1.315] |
| K03621 | KSG66\_07940 | plsX; phosphate acyltransferase [EC:2.3.1.274] |
| K07029 | KSG66\_03490 | dagK; diacylglycerol kinase (ATP) [EC:2.7.1.107] |
| K07406 | KSG66\_13935 | melA; alpha-galactosidase [EC:3.2.1.22] |
| K08591 | KSG66\_09030 | plsY; acyl phosphate:glycerol-3-phosphate acyltransferase [EC:2.3.1.275] |
| K19005 | KSG66\_03645 | ltaS; lipoteichoic acid synthase [EC:2.7.8.20] |
| K19005 | KSG66\_03845 | ltaS; lipoteichoic acid synthase [EC:2.7.8.20] |
| K19005 | KSG66\_15575 | ltaS; lipoteichoic acid synthase [EC:2.7.8.20] |

| 56 | Lysine degradation | ko00310 || K00128 | KSG66\_09600 | ALDH; aldehyde dehydrogenase (NAD+) [EC:1.2.1.3] |
| K00128 | KSG66\_13620 | ALDH; aldehyde dehydrogenase (NAD+) [EC:1.2.1.3] |
| K00128 | KSG66\_18800 | ALDH; aldehyde dehydrogenase (NAD+) [EC:1.2.1.3] |
| K00135 | KSG66\_02070 | gabD; succinate-semialdehyde dehydrogenase / glutarate-semialdehyde dehydrogenase [EC:1.2.1.16 1.2.1.79 1.2.1.20] |
| K00382 | KSG66\_04065 | DLD, lpd, pdhD; dihydrolipoamide dehydrogenase [EC:1.8.1.4] |
| K00382 | KSG66\_07320 | DLD, lpd, pdhD; dihydrolipoamide dehydrogenase [EC:1.8.1.4] |
| K00382 | KSG66\_11350 | DLD, lpd, pdhD; dihydrolipoamide dehydrogenase [EC:1.8.1.4] |
| K00626 | KSG66\_05195 | E2.3.1.9, atoB; acetyl-CoA C-acetyltransferase [EC:2.3.1.9] |
| K00626 | KSG66\_11405 | E2.3.1.9, atoB; acetyl-CoA C-acetyltransferase [EC:2.3.1.9] |
| K00658 | KSG66\_09630 | DLST, sucB; 2-oxoglutarate dehydrogenase E2 component (dihydrolipoamide succinyltransferase) [EC:2.3.1.61] |
| K00824 | KSG66\_04865 | dat; D-alanine transaminase [EC:2.6.1.21] |
| K01034 | KSG66\_09850 | atoD; acetate CoA/acetoacetate CoA-transferase alpha subunit [EC:2.8.3.8 2.8.3.9] |
| K01035 | KSG66\_09845 | atoA; acetate CoA/acetoacetate CoA-transferase beta subunit [EC:2.8.3.8 2.8.3.9] |
| K01843 | KSG66\_09830 | kamA; lysine 2,3-aminomutase [EC:5.4.3.2] |
| K03897 | KSG66\_04945 | iucD; lysine N6-hydroxylase [EC:1.14.13.59] |
| K07250 | KSG66\_02065 | gabT; 4-aminobutyrate aminotransferase / (S)-3-amino-2-methylpropionate transaminase / 5-aminovalerate transaminase [EC:2.6.1.19 2.6.1.22 2.6.1.48] |

| 57 | Sulfur metabolism | ko00920 || K00380 | KSG66\_15640 | cysJ; sulfite reductase (NADPH) flavoprotein alpha-component [EC:1.8.1.2] |
| K00381 | KSG66\_15635 | cysI; sulfite reductase (NADPH) hemoprotein beta-component [EC:1.8.1.2] |
| K00390 | KSG66\_07780 | cysH; phosphoadenosine phosphosulfate reductase [EC:1.8.4.8 1.8.4.10] |
| K00640 | KSG66\_00615 | cysE; serine O-acetyltransferase [EC:2.3.1.30] |
| K00651 | KSG66\_10170 | metA; homoserine O-succinyltransferase/O-acetyltransferase [EC:2.3.1.46 2.3.1.31] |
| K00860 | KSG66\_07795 | cysC; adenylylsulfate kinase [EC:2.7.1.25] |
| K00958 | KSG66\_07790 | sat, met3; sulfate adenylyltransferase [EC:2.7.7.4] |
| K01738 | KSG66\_00440 | cysK; cysteine synthase [EC:2.5.1.47] |
| K01738 | KSG66\_13860 | cysK; cysteine synthase [EC:2.5.1.47] |
| K01739 | KSG66\_05910 | metB; cystathionine gamma-synthase [EC:2.5.1.48] |
| K04091 | KSG66\_04495 | ssuD; alkanesulfonate monooxygenase [EC:1.14.14.5] |
| K06881 | KSG66\_13480 | nrnA; bifunctional oligoribonuclease and PAP phosphatase NrnA [EC:3.1.3.7 3.1.13.3] |
| K15553 | KSG66\_04485 | ssuA; sulfonate transport system substrate-binding protein |
| K15554 | KSG66\_04490 | ssuC; sulfonate transport system permease protein |
| K15555 | KSG66\_04480 | ssuB; sulfonate transport system ATP-binding protein [EC:3.6.3.-] |
| K17217 | KSG66\_12470 | mccB; cystathionine gamma-lyase / homocysteine desulfhydrase [EC:4.4.1.1 4.4.1.2] |

| 58 | DNA replication | ko03030 || K01972 | KSG66\_03440 | E6.5.1.2, ligA, ligB; DNA ligase (NAD+) [EC:6.5.1.2] |
| K02314 | KSG66\_19080 | dnaB; replicative DNA helicase [EC:3.6.4.12] |
| K02316 | KSG66\_11935 | dnaG; DNA primase [EC:2.7.7.101] |
| K02335 | KSG66\_13370 | polA; DNA polymerase I [EC:2.7.7.7] |
| K02337 | KSG66\_13460 | dnaE; DNA polymerase III subunit alpha [EC:2.7.7.7] |
| K02338 | KSG66\_00010 | dnaN; DNA polymerase III subunit beta [EC:2.7.7.7] |
| K02340 | KSG66\_12110 | holA; DNA polymerase III subunit delta [EC:2.7.7.7] |
| K02341 | KSG66\_00220 | holB; DNA polymerase III subunit delta' [EC:2.7.7.7] |
| K02343 | KSG66\_00135 | dnaX; DNA polymerase III subunit gamma/tau [EC:2.7.7.7] |
| K03111 | KSG66\_17145 | ssb; single-strand DNA-binding protein |
| K03111 | KSG66\_19330 | ssb; single-strand DNA-binding protein |
| K03469 | KSG66\_10205 | rnhA, RNASEH1; ribonuclease HI [EC:3.1.26.4] |
| K03469 | KSG66\_10215 | rnhA, RNASEH1; ribonuclease HI [EC:3.1.26.4] |
| K03470 | KSG66\_08025 | rnhB; ribonuclease HII [EC:3.1.26.4] |
| K03471 | KSG66\_13150 | rnhC; ribonuclease HIII [EC:3.1.26.4] |
| K03763 | KSG66\_08290 | polC; DNA polymerase III subunit alpha, Gram-positive type [EC:2.7.7.7] |

| 59 | Nicotinate and nicotinamide metabolism | ko00760 || K00135 | KSG66\_02070 | gabD; succinate-semialdehyde dehydrogenase / glutarate-semialdehyde dehydrogenase [EC:1.2.1.16 1.2.1.79 1.2.1.20] |
| K00278 | KSG66\_12765 | nadB; L-aspartate oxidase [EC:1.4.3.16] |
| K00763 | KSG66\_14725 | pncB, NAPRT1; nicotinate phosphoribosyltransferase [EC:6.3.4.21] |
| K00767 | KSG66\_12760 | nadC, QPRT; nicotinate-nucleotide pyrophosphorylase (carboxylating) [EC:2.4.2.19] |
| K00858 | KSG66\_05755 | ppnK, NADK; NAD+ kinase [EC:2.7.1.23] |
| K00858 | KSG66\_13555 | ppnK, NADK; NAD+ kinase [EC:2.7.1.23] |
| K00969 | KSG66\_12160 | nadD; nicotinate-nucleotide adenylyltransferase [EC:2.7.7.18] |
| K01081 | KSG66\_04640 | E3.1.3.5; 5'-nucleotidase [EC:3.1.3.5] |
| K01081 | KSG66\_15045 | E3.1.3.5; 5'-nucleotidase [EC:3.1.3.5] |
| K01916 | KSG66\_01685 | nadE; NAD+ synthase [EC:6.3.1.5] |
| K03517 | KSG66\_12755 | nadA; quinolinate synthase [EC:2.5.1.72] |
| K03742 | KSG66\_08465 | pncC; nicotinamide-nucleotide amidase [EC:3.5.1.42] |
| K03783 | KSG66\_10970 | punA, PNP; purine-nucleoside phosphorylase [EC:2.4.2.1] |
| K03784 | KSG66\_09785 | deoD; purine-nucleoside phosphorylase [EC:2.4.2.1] |
| K08693 | KSG66\_03900 | yfkN; 2',3'-cyclic-nucleotide 2'-phosphodiesterase / 3'-nucleotidase / 5'-nucleotidase [EC:3.1.4.16 3.1.3.6 3.1.3.5] |

| 60 | Terpenoid backbone biosynthesis | ko00900 || K00099 | KSG66\_08275 | dxr; 1-deoxy-D-xylulose-5-phosphate reductoisomerase [EC:1.1.1.267] |
| K00626 | KSG66\_05195 | E2.3.1.9, atoB; acetyl-CoA C-acetyltransferase [EC:2.3.1.9] |
| K00626 | KSG66\_11405 | E2.3.1.9, atoB; acetyl-CoA C-acetyltransferase [EC:2.3.1.9] |
| K00805 | KSG66\_10615 | hepST; heptaprenyl diphosphate synthase [EC:2.5.1.30] |
| K00805 | KSG66\_10625 | hepST; heptaprenyl diphosphate synthase [EC:2.5.1.30] |
| K00806 | KSG66\_08265 | uppS; undecaprenyl diphosphate synthase [EC:2.5.1.31] |
| K00919 | KSG66\_00295 | ispE; 4-diphosphocytidyl-2-C-methyl-D-erythritol kinase [EC:2.7.1.148] |
| K00991 | KSG66\_00600 | ispD; 2-C-methyl-D-erythritol 4-phosphate cytidylyltransferase [EC:2.7.7.60] |
| K01662 | KSG66\_11485 | dxs; 1-deoxy-D-xylulose-5-phosphate synthase [EC:2.2.1.7] |
| K01770 | KSG66\_00605 | ispF; 2-C-methyl-D-erythritol 2,4-cyclodiphosphate synthase [EC:4.6.1.12] |
| K01823 | KSG66\_10680 | idi, IDI; isopentenyl-diphosphate Delta-isomerase [EC:5.3.3.2] |
| K03526 | KSG66\_11865 | gcpE, ispG; (E)-4-hydroxy-3-methylbut-2-enyl-diphosphate synthase [EC:1.17.7.1 1.17.7.3] |
| K03527 | KSG66\_11910 | ispH, lytB; 4-hydroxy-3-methylbut-2-en-1-yl diphosphate reductase [EC:1.17.7.4] |
| K06013 | KSG66\_05175 | STE24; STE24 endopeptidase [EC:3.4.24.84] |
| K13789 | KSG66\_11490 | GGPS; geranylgeranyl diphosphate synthase, type II [EC:2.5.1.1 2.5.1.10 2.5.1.29] |

| 61 | RNA degradation | ko03018 || K00850 | KSG66\_13440 | pfkA, PFK; 6-phosphofructokinase 1 [EC:2.7.1.11] |
| K00962 | KSG66\_08345 | pnp, PNPT1; polyribonucleotide nucleotidyltransferase [EC:2.7.7.8] |
| K01689 | KSG66\_15970 | ENO, eno; enolase [EC:4.2.1.11] |
| K03628 | KSG66\_17510 | rho; transcription termination factor Rho |
| K03654 | KSG66\_09540 | recQ; ATP-dependent DNA helicase RecQ [EC:3.6.4.12] |
| K03654 | KSG66\_10755 | recQ; ATP-dependent DNA helicase RecQ [EC:3.6.4.12] |
| K03666 | KSG66\_08665 | hfq; host factor-I protein |
| K04043 | KSG66\_12065 | dnaK, HSPA9; molecular chaperone DnaK |
| K04077 | KSG66\_03165 | groEL, HSPD1; chaperonin GroEL |
| K05592 | KSG66\_02445 | deaD, cshA; ATP-dependent RNA helicase DeaD [EC:3.6.4.13] |
| K05592 | KSG66\_18490 | deaD, cshA; ATP-dependent RNA helicase DeaD [EC:3.6.4.13] |
| K12573 | KSG66\_15790 | rnr, vacB; ribonuclease R [EC:3.1.-.-] |
| K12574 | KSG66\_07230 | rnj; ribonuclease J [EC:3.1.-.-] |
| K12574 | KSG66\_08390 | rnj; ribonuclease J [EC:3.1.-.-] |
| K18682 | KSG66\_08480 | rny; ribonucrease Y [EC:3.1.-.-] |

| 62 | Tryptophan metabolism | ko00380 || K00128 | KSG66\_09600 | ALDH; aldehyde dehydrogenase (NAD+) [EC:1.2.1.3] |
| K00128 | KSG66\_13620 | ALDH; aldehyde dehydrogenase (NAD+) [EC:1.2.1.3] |
| K00128 | KSG66\_18800 | ALDH; aldehyde dehydrogenase (NAD+) [EC:1.2.1.3] |
| K00382 | KSG66\_04065 | DLD, lpd, pdhD; dihydrolipoamide dehydrogenase [EC:1.8.1.4] |
| K00382 | KSG66\_07320 | DLD, lpd, pdhD; dihydrolipoamide dehydrogenase [EC:1.8.1.4] |
| K00382 | KSG66\_11350 | DLD, lpd, pdhD; dihydrolipoamide dehydrogenase [EC:1.8.1.4] |
| K00626 | KSG66\_05195 | E2.3.1.9, atoB; acetyl-CoA C-acetyltransferase [EC:2.3.1.9] |
| K00626 | KSG66\_11405 | E2.3.1.9, atoB; acetyl-CoA C-acetyltransferase [EC:2.3.1.9] |
| K00658 | KSG66\_09630 | DLST, sucB; 2-oxoglutarate dehydrogenase E2 component (dihydrolipoamide succinyltransferase) [EC:2.3.1.61] |
| K03781 | KSG66\_04470 | katE, CAT, catB, srpA; catalase [EC:1.11.1.6] |
| K03781 | KSG66\_18315 | katE, CAT, catB, srpA; catalase [EC:1.11.1.6] |
| K03781 | KSG66\_18465 | katE, CAT, catB, srpA; catalase [EC:1.11.1.6] |
| K14338 | KSG66\_03640 | cypD\_E, CYP102A2\_3; cytochrome P450 / NADPH-cytochrome P450 reductase [EC:1.14.14.1 1.6.2.4] |
| K14338 | KSG66\_12420 | cypD\_E, CYP102A2\_3; cytochrome P450 / NADPH-cytochrome P450 reductase [EC:1.14.14.1 1.6.2.4] |

| 63 | Inositol phosphate metabolism | ko00562 || K00010 | KSG66\_18720 | iolG; myo-inositol 2-dehydrogenase / D-chiro-inositol 1-dehydrogenase [EC:1.1.1.18 1.1.1.369] |
| K00140 | KSG66\_18750 | mmsA, iolA, ALDH6A1; malonate-semialdehyde dehydrogenase (acetylating) / methylmalonate-semialdehyde dehydrogenase [EC:1.2.1.18 1.2.1.27] |
| K01083 | KSG66\_09895 | E3.1.3.8; 3-phytase [EC:3.1.3.8] |
| K01092 | KSG66\_01010 | E3.1.3.25, IMPA, suhB; myo-inositol-1(or 4)-monophosphatase [EC:3.1.3.25] |
| K01092 | KSG66\_07360 | E3.1.3.25, IMPA, suhB; myo-inositol-1(or 4)-monophosphatase [EC:3.1.3.25] |
| K01803 | KSG66\_15980 | TPI, tpiA; triosephosphate isomerase (TIM) [EC:5.3.1.1] |
| K03335 | KSG66\_18730 | iolE; inosose dehydratase [EC:4.2.1.44] |
| K03336 | KSG66\_18735 | iolD; 3D-(3,5/4)-trihydroxycyclohexane-1,2-dione acylhydrolase (decyclizing) [EC:3.7.1.22] |
| K03337 | KSG66\_18745 | iolB; 5-deoxy-glucuronate isomerase [EC:5.3.1.30] |
| K03338 | KSG66\_18740 | iolC; 5-dehydro-2-deoxygluconokinase [EC:2.7.1.92] |
| K03339 | KSG66\_18705 | iolJ; 6-phospho-5-dehydro-2-deoxy-D-gluconate aldolase [EC:4.1.2.29] |
| K06606 | KSG66\_18710 | iolI; 2-keto-myo-inositol isomerase [EC:5.3.99.11] |
| K16044 | KSG66\_15690 | iolW; scyllo-inositol 2-dehydrogenase (NADP+) [EC:1.1.1.371] |
| K22230 | KSG66\_14425 | iolU; scyllo-inositol 2-dehydrogenase (NADP+) [EC:1.1.1.-] |

| 64 | Bacterial secretion system | ko03070 || K03070 | KSG66\_16620 | secA; preprotein translocase subunit SecA |
| K03073 | KSG66\_00650 | secE; preprotein translocase subunit SecE |
| K03075 | KSG66\_15800 | secG; preprotein translocase subunit SecG |
| K03076 | KSG66\_00830 | secY; preprotein translocase subunit SecY |
| K03106 | KSG66\_07985 | SRP54, ffh; signal recognition particle subunit SRP54 [EC:3.6.5.4] |
| K03110 | KSG66\_07970 | ftsY; fused signal recognition particle receptor |
| K03116 | KSG66\_01475 | tatA; sec-independent protein translocase protein TatA |
| K03116 | KSG66\_03140 | tatA; sec-independent protein translocase protein TatA |
| K03118 | KSG66\_01480 | tatC; sec-independent protein translocase protein TatC |
| K03118 | KSG66\_03145 | tatC; sec-independent protein translocase protein TatC |
| K03210 | KSG66\_12710 | yajC; preprotein translocase subunit YajC |
| K03217 | KSG66\_11275 | yidC, spoIIIJ, OXA1, ccfA; YidC/Oxa1 family membrane protein insertase |
| K03217 | KSG66\_19405 | yidC, spoIIIJ, OXA1, ccfA; YidC/Oxa1 family membrane protein insertase |
| K12257 | KSG66\_12685 | secDF; SecD/SecF fusion protein |

| 65 | Valine, leucine and isoleucine biosynthesis | ko00290 || K00052 | KSG66\_12970 | leuB, IMDH; 3-isopropylmalate dehydrogenase [EC:1.1.1.85] |
| K00053 | KSG66\_12980 | ilvC; ketol-acid reductoisomerase [EC:1.1.1.86] |
| K00263 | KSG66\_11360 | E1.4.1.9; leucine dehydrogenase [EC:1.4.1.9] |
| K00826 | KSG66\_01380 | E2.6.1.42, ilvE; branched-chain amino acid aminotransferase [EC:2.6.1.42] |
| K00826 | KSG66\_18220 | E2.6.1.42, ilvE; branched-chain amino acid aminotransferase [EC:2.6.1.42] |
| K01649 | KSG66\_12975 | leuA, IMS; 2-isopropylmalate synthase [EC:2.3.3.13] |
| K01652 | KSG66\_12990 | E2.2.1.6L, ilvB, ilvG, ilvI; acetolactate synthase I/II/III large subunit [EC:2.2.1.6] |
| K01652 | KSG66\_16980 | E2.2.1.6L, ilvB, ilvG, ilvI; acetolactate synthase I/II/III large subunit [EC:2.2.1.6] |
| K01653 | KSG66\_12985 | E2.2.1.6S, ilvH, ilvN; acetolactate synthase I/III small subunit [EC:2.2.1.6] |
| K01687 | KSG66\_10150 | ilvD; dihydroxy-acid dehydratase [EC:4.2.1.9] |
| K01703 | KSG66\_12965 | leuC, IPMI-L; 3-isopropylmalate/(R)-2-methylmalate dehydratase large subunit [EC:4.2.1.33 4.2.1.35] |
| K01704 | KSG66\_12960 | leuD, IPMI-S; 3-isopropylmalate/(R)-2-methylmalate dehydratase small subunit [EC:4.2.1.33 4.2.1.35] |
| K01754 | KSG66\_10105 | E4.3.1.19, ilvA, tdcB; threonine dehydratase [EC:4.3.1.19] |

| 66 | Cell cycle - Caulobacter | ko04112 || K01338 | KSG66\_12935 | lon; ATP-dependent Lon protease [EC:3.4.21.53] |
| K01358 | KSG66\_16280 | clpP, CLPP; ATP-dependent Clp protease, protease subunit [EC:3.4.21.92] |
| K02313 | KSG66\_00005 | dnaA; chromosomal replication initiator protein |
| K02314 | KSG66\_19080 | dnaB; replicative DNA helicase [EC:3.6.4.12] |
| K02563 | KSG66\_07620 | murG; UDP-N-acetylglucosamine--N-acetylmuramyl-(pentapeptide) pyrophosphoryl-undecaprenol N-acetylglucosamine transferase [EC:2.4.1.227] |
| K03531 | KSG66\_07640 | ftsZ; cell division protein FtsZ |
| K03544 | KSG66\_12945 | clpX, CLPX; ATP-dependent Clp protease ATP-binding subunit ClpX |
| K03588 | KSG66\_07435 | ftsW, spoVE; cell division protein FtsW |
| K03588 | KSG66\_07615 | ftsW, spoVE; cell division protein FtsW |
| K03588 | KSG66\_17230 | ftsW, spoVE; cell division protein FtsW |
| K03589 | KSG66\_07630 | ftsQ; cell division protein FtsQ |
| K03590 | KSG66\_07635 | ftsA; cell division protein FtsA |
| K11749 | KSG66\_08280 | rseP; regulator of sigma E protease [EC:3.4.24.-] |

| 67 | Sulfur relay system | ko04122 || K00566 | KSG66\_12605 | mnmA, trmU; tRNA-uridine 2-sulfurtransferase [EC:2.8.1.13] |
| K03148 | KSG66\_05800 | thiF; sulfur carrier protein ThiS adenylyltransferase [EC:2.7.7.73] |
| K03151 | KSG66\_13575 | thiI; tRNA uracil 4-sulfurtransferase [EC:2.8.1.4] |
| K03154 | KSG66\_05790 | thiS; sulfur carrier protein |
| K03635 | KSG66\_07125 | MOCS2B, moaE; molybdopterin synthase catalytic subunit [EC:2.8.1.12] |
| K03636 | KSG66\_07130 | moaD, cysO; sulfur-carrier protein |
| K03637 | KSG66\_03130 | moaC, CNX3; cyclic pyranopterin monophosphate synthase [EC:4.6.1.17] |
| K03639 | KSG66\_17320 | moaA, CNX2; GTP 3',8-cyclase [EC:4.1.99.22] |
| K04085 | KSG66\_02700 | tusA, sirA; tRNA 2-thiouridine synthesizing protein A [EC:2.8.1.-] |
| K04487 | KSG66\_12610 | iscS, NFS1; cysteine desulfurase [EC:2.8.1.7] |
| K04487 | KSG66\_12770 | iscS, NFS1; cysteine desulfurase [EC:2.8.1.7] |
| K21029 | KSG66\_07110 | moeB; molybdopterin-synthase adenylyltransferase [EC:2.7.7.80] |
| K21147 | KSG66\_19005 | moeZR, moeBR; sulfur-carrier protein adenylyltransferase/sulfurtransferase [EC:2.7.7.80 2.7.7.- 2.8.1.11 2.8.1.-] |

| 68 | Ubiquinone and other terpenoid-quinone biosynthesis | ko00130 || K00355 | KSG66\_04130 | NQO1; NAD(P)H dehydrogenase (quinone) [EC:1.6.5.2] |
| K01661 | KSG66\_14190 | menB; naphthoate synthase [EC:4.1.3.36] |
| K01911 | KSG66\_14185 | menE; O-succinylbenzoic acid---CoA ligase [EC:6.2.1.26] |
| K02361 | KSG66\_14835 | entC; isochorismate synthase [EC:5.4.4.2] |
| K02548 | KSG66\_18190 | menA; 1,4-dihydroxy-2-naphthoate octaprenyltransferase [EC:2.5.1.74 2.5.1.-] |
| K02549 | KSG66\_14180 | menC; O-succinylbenzoate synthase [EC:4.2.1.113] |
| K02551 | KSG66\_14200 | menD; 2-succinyl-5-enolpyruvyl-6-hydroxy-3-cyclohexene-1-carboxylate synthase [EC:2.2.1.9] |
| K03183 | KSG66\_10620 | ubiE; demethylmenaquinone methyltransferase / 2-methoxy-6-polyprenyl-1,4-benzoquinol methylase [EC:2.1.1.163 2.1.1.201] |
| K03186 | KSG66\_01890 | ubiX, bsdB, PAD1; flavin prenyltransferase [EC:2.5.1.129] |
| K03809 | KSG66\_04580 | wrbA; NAD(P)H dehydrogenase (quinone) [EC:1.6.5.2] |
| K08680 | KSG66\_14195 | menH; 2-succinyl-6-hydroxy-2,4-cyclohexadiene-1-carboxylate synthase [EC:4.2.99.20] |
| K19222 | KSG66\_14680 | menI, DHNAT; 1,4-dihydroxy-2-naphthoyl-CoA hydrolase [EC:3.1.2.28] |

| 69 | One carbon pool by folate | ko00670 || K00287 | KSG66\_10125 | DHFR, folA; dihydrofolate reductase [EC:1.5.1.3] |
| K00560 | KSG66\_10130 | thyA, TYMS; thymidylate synthase [EC:2.1.1.45] |
| K00600 | KSG66\_17420 | glyA, SHMT; glycine hydroxymethyltransferase [EC:2.1.2.1] |
| K00602 | KSG66\_03395 | purH; phosphoribosylaminoimidazolecarboxamide formyltransferase / IMP cyclohydrolase [EC:2.1.2.3 3.5.4.10] |
| K00604 | KSG66\_07860 | MTFMT, fmt; methionyl-tRNA formyltransferase [EC:2.1.2.9] |
| K00605 | KSG66\_11630 | gcvT, AMT; aminomethyltransferase [EC:2.1.2.10] |
| K01433 | KSG66\_06560 | purU; formyltetrahydrofolate deformylase [EC:3.5.1.10] |
| K01491 | KSG66\_11505 | folD; methylenetetrahydrofolate dehydrogenase (NADP+) / methenyltetrahydrofolate cyclohydrolase [EC:1.5.1.5 3.5.4.9] |
| K01934 | KSG66\_11790 | MTHFS; 5-formyltetrahydrofolate cyclo-ligase [EC:6.3.3.2] |
| K08289 | KSG66\_01345 | purT; phosphoribosylglycinamide formyltransferase 2 [EC:2.1.2.2] |
| K11175 | KSG66\_03390 | purN; phosphoribosylglycinamide formyltransferase 1 [EC:2.1.2.2] |
| K24042 | KSG66\_05445 |  |

| 70 | Selenocompound metabolism | ko00450 || K00384 | KSG66\_10720 | trxB, TRR; thioredoxin reductase (NADPH) [EC:1.8.1.9] |
| K00384 | KSG66\_16390 | trxB, TRR; thioredoxin reductase (NADPH) [EC:1.8.1.9] |
| K00549 | KSG66\_06615 | metE; 5-methyltetrahydropteroyltriglutamate--homocysteine methyltransferase [EC:2.1.1.14] |
| K00549 | KSG66\_18440 | metE; 5-methyltetrahydropteroyltriglutamate--homocysteine methyltransferase [EC:2.1.1.14] |
| K00958 | KSG66\_07790 | sat, met3; sulfate adenylyltransferase [EC:2.7.7.4] |
| K01739 | KSG66\_05910 | metB; cystathionine gamma-synthase [EC:2.5.1.48] |
| K01760 | KSG66\_05915 | metC; cysteine-S-conjugate beta-lyase [EC:4.4.1.13] |
| K01874 | KSG66\_00255 | MARS, metG; methionyl-tRNA synthetase [EC:6.1.1.10] |
| K11717 | KSG66\_15210 | sufS; cysteine desulfurase / selenocysteine lyase [EC:2.8.1.7 4.4.1.16] |
| K14155 | KSG66\_14570 | patB, malY; cysteine-S-conjugate beta-lyase [EC:4.4.1.13] |
| K24042 | KSG66\_05445 |  |

| 71 | Carbon fixation in photosynthetic organisms | ko00710 || K00024 | KSG66\_13385 | mdh; malate dehydrogenase [EC:1.1.1.37] |
| K00134 | KSG66\_13335 | GAPDH, gapA; glyceraldehyde 3-phosphate dehydrogenase [EC:1.2.1.12] |
| K00134 | KSG66\_15990 | GAPDH, gapA; glyceraldehyde 3-phosphate dehydrogenase [EC:1.2.1.12] |
| K00615 | KSG66\_08935 | E2.2.1.1, tktA, tktB; transketolase [EC:2.2.1.1] |
| K00927 | KSG66\_15985 | PGK, pgk; phosphoglycerate kinase [EC:2.7.2.3] |
| K01610 | KSG66\_14085 | E4.1.1.49, pckA; phosphoenolpyruvate carboxykinase (ATP) [EC:4.1.1.49] |
| K01624 | KSG66\_17530 | FBA, fbaA; fructose-bisphosphate aldolase, class II [EC:4.1.2.13] |
| K01783 | KSG66\_07890 | rpe, RPE; ribulose-phosphate 3-epimerase [EC:5.1.3.1] |
| K01803 | KSG66\_15980 | TPI, tpiA; triosephosphate isomerase (TIM) [EC:5.3.1.1] |
| K01808 | KSG66\_17430 | rpiB; ribose 5-phosphate isomerase B [EC:5.3.1.6] |
| K02446 | KSG66\_17515 | glpX; fructose-1,6-bisphosphatase II [EC:3.1.3.11] |

| 72 | Base excision repair | ko03410 || K01142 | KSG66\_19310 | E3.1.11.2, xthA; exodeoxyribonuclease III [EC:3.1.11.2] |
| K01151 | KSG66\_11895 | nfo; deoxyribonuclease IV [EC:3.1.21.2] |
| K01247 | KSG66\_04025 | alkA; DNA-3-methyladenine glycosylase II [EC:3.2.2.21] |
| K01972 | KSG66\_03440 | E6.5.1.2, ligA, ligB; DNA ligase (NAD+) [EC:6.5.1.2] |
| K02335 | KSG66\_13370 | polA; DNA polymerase I [EC:2.7.7.7] |
| K03575 | KSG66\_04260 | mutY; A/G-specific adenine glycosylase [EC:3.2.2.31] |
| K03648 | KSG66\_17960 | UNG, UDG; uracil-DNA glycosylase [EC:3.2.2.27] |
| K03652 | KSG66\_18250 | MPG; DNA-3-methyladenine glycosylase [EC:3.2.2.21] |
| K07462 | KSG66\_12670 | recJ; single-stranded-DNA-specific exonuclease [EC:3.1.-.-] |
| K10563 | KSG66\_13365 | mutM, fpg; formamidopyrimidine-DNA glycosylase [EC:3.2.2.23 4.2.99.18] |
| K10773 | KSG66\_10410 | NTH; endonuclease III [EC:4.2.99.18] |

| 73 | Prodigiosin biosyntheses | ko00333 || K00059 | KSG66\_07950 | fabG; 3-oxoacyl-[acyl-carrier protein] reductase [EC:1.1.1.100] |
| K00059 | KSG66\_08440 | fabG; 3-oxoacyl-[acyl-carrier protein] reductase [EC:1.1.1.100] |
| K00059 | KSG66\_09410 | fabG; 3-oxoacyl-[acyl-carrier protein] reductase [EC:1.1.1.100] |
| K00059 | KSG66\_11195 | fabG; 3-oxoacyl-[acyl-carrier protein] reductase [EC:1.1.1.100] |
| K00059 | KSG66\_13500 | fabG; 3-oxoacyl-[acyl-carrier protein] reductase [EC:1.1.1.100] |
| K00059 | KSG66\_15330 | fabG; 3-oxoacyl-[acyl-carrier protein] reductase [EC:1.1.1.100] |
| K00059 | KSG66\_15750 | fabG; 3-oxoacyl-[acyl-carrier protein] reductase [EC:1.1.1.100] |
| K00208 | KSG66\_05810 | fabI; enoyl-[acyl-carrier protein] reductase I [EC:1.3.1.9 1.3.1.10] |
| K00645 | KSG66\_07945 | fabD; [acyl-carrier-protein] S-malonyltransferase [EC:2.3.1.39] |
| K00645 | KSG66\_09175 | fabD; [acyl-carrier-protein] S-malonyltransferase [EC:2.3.1.39] |

| 74 | Riboflavin metabolism | ko00740 || K00793 | KSG66\_10875 | ribE, RIB5; riboflavin synthase [EC:2.5.1.9] |
| K00794 | KSG66\_10865 | ribH, RIB4; 6,7-dimethyl-8-ribityllumazine synthase [EC:2.5.1.78] |
| K11752 | KSG66\_10880 | ribD; diaminohydroxyphosphoribosylaminopyrimidine deaminase / 5-amino-6-(5-phosphoribosylamino)uracil reductase [EC:3.5.4.26 1.1.1.193] |
| K11753 | KSG66\_08335 | ribF; riboflavin kinase / FMN adenylyltransferase [EC:2.7.1.26 2.7.7.2] |
| K14652 | KSG66\_10870 | ribBA; 3,4-dihydroxy 2-butanone 4-phosphate synthase / GTP cyclohydrolase II [EC:4.1.99.12 3.5.4.25] |
| K19285 | KSG66\_18025 | nfrA1; FMN reductase (NADPH) [EC:1.5.1.38] |
| K19286 | KSG66\_02045 | nfrA2; FMN reductase [NAD(P)H] [EC:1.5.1.39] |
| K21064 | KSG66\_02130 | ycsE, yitU, ywtE; 5-amino-6-(5-phospho-D-ribitylamino)uracil phosphatase [EC:3.1.3.104] |
| K21064 | KSG66\_05505 | ycsE, yitU, ywtE; 5-amino-6-(5-phospho-D-ribitylamino)uracil phosphatase [EC:3.1.3.104] |
| K21064 | KSG66\_16885 | ycsE, yitU, ywtE; 5-amino-6-(5-phospho-D-ribitylamino)uracil phosphatase [EC:3.1.3.104] |

| 75 | Nonribosomal peptide structures | ko01054 || K01779 | KSG66\_02710 | racD; aspartate racemase [EC:5.1.1.13] |
| K01779 | KSG66\_12225 | racD; aspartate racemase [EC:5.1.1.13] |
| K15655 | KSG66\_01840 | srfAB, lchAB; surfactin family lipopeptide synthetase B |
| K15656 | KSG66\_01845 | srfAC, lchAC; surfactin family lipopeptide synthetase C |
| K15661 | KSG66\_09170 | ituA, mycA, bmyA; iturin family lipopeptide synthetase A |
| K15662 | KSG66\_09165 | ituB, mycB, bmyB; iturin family lipopeptide synthetase B |
| K15663 | KSG66\_09160 | ituC, mycC, bmyC; iturin family lipopeptide synthetase C |
| K15665 | KSG66\_09305 | K15665, ppsB, fenD; fengycin family lipopeptide synthetase B |
| K15666 | KSG66\_09300 | K15666, ppsC, fenE; fengycin family lipopeptide synthetase C |
| K15668 | KSG66\_09290 | K15668, ppsE, fenB; fengycin family lipopeptide synthetase E |

| 76 | Degradation of aromatic compounds | ko01220 || K00121 | KSG66\_01785 | frmA, ADH5, adhC; S-(hydroxymethyl)glutathione dehydrogenase / alcohol dehydrogenase [EC:1.1.1.284 1.1.1.1] |
| K00121 | KSG66\_02865 | frmA, ADH5, adhC; S-(hydroxymethyl)glutathione dehydrogenase / alcohol dehydrogenase [EC:1.1.1.284 1.1.1.1] |
| K00483 | KSG66\_01300 | hpaB; 4-hydroxyphenylacetate 3-monooxygenase [EC:1.14.14.9] |
| K01607 | KSG66\_19235 | pcaC; 4-carboxymuconolactone decarboxylase [EC:4.1.1.44] |
| K01612 | KSG66\_01895 | bsdC; vanillate/4-hydroxybenzoate decarboxylase subunit C [EC:4.1.1.- 4.1.1.61] |
| K01821 | KSG66\_17695 | praC, xylH; 4-oxalocrotonate tautomerase [EC:5.3.2.6] |
| K03186 | KSG66\_01890 | ubiX, bsdB, PAD1; flavin prenyltransferase [EC:2.5.1.129] |
| K07104 | KSG66\_04115 | catE; catechol 2,3-dioxygenase [EC:1.13.11.2] |
| K13953 | KSG66\_08815 | adhP; alcohol dehydrogenase, propanol-preferring [EC:1.1.1.1] |
| K21759 | KSG66\_01900 | bsdD; vanillate/4-hydroxybenzoate decarboxylase subunit D [EC:4.1.1.- 4.1.1.61] |

| 77 | Monobactam biosynthesis | ko00261 || K00133 | KSG66\_08375 | asd; aspartate-semialdehyde dehydrogenase [EC:1.2.1.11] |
| K00215 | KSG66\_10485 | dapB; 4-hydroxy-tetrahydrodipicolinate reductase [EC:1.17.1.8] |
| K00928 | KSG66\_02010 | lysC; aspartate kinase [EC:2.7.2.4] |
| K00928 | KSG66\_08380 | lysC; aspartate kinase [EC:2.7.2.4] |
| K00928 | KSG66\_13075 | lysC; aspartate kinase [EC:2.7.2.4] |
| K00958 | KSG66\_07790 | sat, met3; sulfate adenylyltransferase [EC:2.7.7.4] |
| K01714 | KSG66\_02785 | dapA; 4-hydroxy-tetrahydrodipicolinate synthase [EC:4.3.3.7] |
| K01714 | KSG66\_08385 | dapA; 4-hydroxy-tetrahydrodipicolinate synthase [EC:4.3.3.7] |
| K05375 | KSG66\_14815 | mbtH, nocI; MbtH protein |

| 78 | Benzoate degradation | ko00362 || K00074 | KSG66\_11400 | paaH, hbd, fadB, mmgB; 3-hydroxybutyryl-CoA dehydrogenase [EC:1.1.1.157] |
| K00626 | KSG66\_05195 | E2.3.1.9, atoB; acetyl-CoA C-acetyltransferase [EC:2.3.1.9] |
| K00626 | KSG66\_11405 | E2.3.1.9, atoB; acetyl-CoA C-acetyltransferase [EC:2.3.1.9] |
| K00632 | KSG66\_15285 | fadA, fadI; acetyl-CoA acyltransferase [EC:2.3.1.16] |
| K01607 | KSG66\_19235 | pcaC; 4-carboxymuconolactone decarboxylase [EC:4.1.1.44] |
| K01821 | KSG66\_17695 | praC, xylH; 4-oxalocrotonate tautomerase [EC:5.3.2.6] |
| K07104 | KSG66\_04115 | catE; catechol 2,3-dioxygenase [EC:1.13.11.2] |
| K07516 | KSG66\_15290 | fadN; 3-hydroxyacyl-CoA dehydrogenase [EC:1.1.1.35] |
| K13767 | KSG66\_13105 | fadB; enoyl-CoA hydratase [EC:4.2.1.17] |

| 79 | Glutathione metabolism | ko00480 || K00031 | KSG66\_13390 | IDH1, IDH2, icd; isocitrate dehydrogenase [EC:1.1.1.42] |
| K00033 | KSG66\_11260 | PGD, gnd, gntZ; 6-phosphogluconate dehydrogenase [EC:1.1.1.44 1.1.1.343] |
| K00036 | KSG66\_11255 | G6PD, zwf; glucose-6-phosphate 1-dehydrogenase [EC:1.1.1.49 1.1.1.363] |
| K00432 | KSG66\_10165 | gpx; glutathione peroxidase [EC:1.11.1.9] |
| K00681 | KSG66\_09350 | ggt; gamma-glutamyltranspeptidase / glutathione hydrolase [EC:2.3.2.2 3.4.19.13] |
| K00797 | KSG66\_17670 | speE, SRM; spermidine synthase [EC:2.5.1.16] |
| K01255 | KSG66\_14865 | CARP, pepA; leucyl aminopeptidase [EC:3.4.11.1] |
| K07160 | KSG66\_02135 | K07160; UPF0271 protein |
| K07232 | KSG66\_07215 | CHAC, chaC; glutathione-specific gamma-glutamylcyclotransferase [EC:4.3.2.7] |

| 80 | Streptomycin biosynthesis | ko00521 || K00010 | KSG66\_18720 | iolG; myo-inositol 2-dehydrogenase / D-chiro-inositol 1-dehydrogenase [EC:1.1.1.18 1.1.1.369] |
| K00067 | KSG66\_17890 | rfbD, rmlD; dTDP-4-dehydrorhamnose reductase [EC:1.1.1.133] |
| K00845 | KSG66\_11770 | glk; glucokinase [EC:2.7.1.2] |
| K00973 | KSG66\_17900 | E2.7.7.24, rfbA, rffH; glucose-1-phosphate thymidylyltransferase [EC:2.7.7.24] |
| K01092 | KSG66\_01010 | E3.1.3.25, IMPA, suhB; myo-inositol-1(or 4)-monophosphatase [EC:3.1.3.25] |
| K01092 | KSG66\_07360 | E3.1.3.25, IMPA, suhB; myo-inositol-1(or 4)-monophosphatase [EC:3.1.3.25] |
| K01710 | KSG66\_17895 | E4.2.1.46, rfbB, rffG; dTDP-glucose 4,6-dehydratase [EC:4.2.1.46] |
| K01790 | KSG66\_17885 | rfbC, rmlC; dTDP-4-dehydrorhamnose 3,5-epimerase [EC:5.1.3.13] |
| K01835 | KSG66\_04700 | pgm; phosphoglucomutase [EC:5.4.2.2] |

| 81 | Aminobenzoate degradation | ko00627 || K01034 | KSG66\_09850 | atoD; acetate CoA/acetoacetate CoA-transferase alpha subunit [EC:2.8.3.8 2.8.3.9] |
| K01035 | KSG66\_09845 | atoA; acetate CoA/acetoacetate CoA-transferase beta subunit [EC:2.8.3.8 2.8.3.9] |
| K01101 | KSG66\_15015 | E3.1.3.41; 4-nitrophenyl phosphatase [EC:3.1.3.41] |
| K01512 | KSG66\_03805 | acyP; acylphosphatase [EC:3.6.1.7] |
| K01612 | KSG66\_01895 | bsdC; vanillate/4-hydroxybenzoate decarboxylase subunit C [EC:4.1.1.- 4.1.1.61] |
| K03186 | KSG66\_01890 | ubiX, bsdB, PAD1; flavin prenyltransferase [EC:2.5.1.129] |
| K14338 | KSG66\_03640 | cypD\_E, CYP102A2\_3; cytochrome P450 / NADPH-cytochrome P450 reductase [EC:1.14.14.1 1.6.2.4] |
| K14338 | KSG66\_12420 | cypD\_E, CYP102A2\_3; cytochrome P450 / NADPH-cytochrome P450 reductase [EC:1.14.14.1 1.6.2.4] |
| K21759 | KSG66\_01900 | bsdD; vanillate/4-hydroxybenzoate decarboxylase subunit D [EC:4.1.1.- 4.1.1.61] |

| 82 | C5-Branched dibasic acid metabolism | ko00660 || K00052 | KSG66\_12970 | leuB, IMDH; 3-isopropylmalate dehydrogenase [EC:1.1.1.85] |
| K01575 | KSG66\_16975 | alsD, budA, aldC; acetolactate decarboxylase [EC:4.1.1.5] |
| K01652 | KSG66\_12990 | E2.2.1.6L, ilvB, ilvG, ilvI; acetolactate synthase I/II/III large subunit [EC:2.2.1.6] |
| K01652 | KSG66\_16980 | E2.2.1.6L, ilvB, ilvG, ilvI; acetolactate synthase I/II/III large subunit [EC:2.2.1.6] |
| K01653 | KSG66\_12985 | E2.2.1.6S, ilvH, ilvN; acetolactate synthase I/III small subunit [EC:2.2.1.6] |
| K01703 | KSG66\_12965 | leuC, IPMI-L; 3-isopropylmalate/(R)-2-methylmalate dehydratase large subunit [EC:4.2.1.33 4.2.1.35] |
| K01704 | KSG66\_12960 | leuD, IPMI-S; 3-isopropylmalate/(R)-2-methylmalate dehydratase small subunit [EC:4.2.1.33 4.2.1.35] |
| K01902 | KSG66\_08045 | sucD; succinyl-CoA synthetase alpha subunit [EC:6.2.1.5] |
| K01903 | KSG66\_08040 | sucC; succinyl-CoA synthetase beta subunit [EC:6.2.1.5] |

| 83 | Nucleotide excision repair | ko03420 || K01972 | KSG66\_03440 | E6.5.1.2, ligA, ligB; DNA ligase (NAD+) [EC:6.5.1.2] |
| K02335 | KSG66\_13370 | polA; DNA polymerase I [EC:2.7.7.7] |
| K03657 | KSG66\_03435 | uvrD, pcrA; DNA helicase II / ATP-dependent DNA helicase PcrA [EC:3.6.4.12] |
| K03657 | KSG66\_05865 | uvrD, pcrA; DNA helicase II / ATP-dependent DNA helicase PcrA [EC:3.6.4.12] |
| K03657 | KSG66\_15645 | uvrD, pcrA; DNA helicase II / ATP-dependent DNA helicase PcrA [EC:3.6.4.12] |
| K03701 | KSG66\_16535 | uvrA; excinuclease ABC subunit A |
| K03702 | KSG66\_16540 | uvrB; excinuclease ABC subunit B |
| K03703 | KSG66\_13080 | uvrC; excinuclease ABC subunit C |
| K03723 | KSG66\_00340 | mfd; transcription-repair coupling factor (superfamily II helicase) [EC:3.6.4.-] |

| 84 | Biofilm formation - Vibrio cholerae | ko05111 || K00640 | KSG66\_00615 | cysE; serine O-acetyltransferase [EC:2.3.1.30] |
| K01791 | KSG66\_16800 | wecB; UDP-N-acetylglucosamine 2-epimerase (non-hydrolysing) [EC:5.1.3.14] |
| K02405 | KSG66\_08235 | fliA; RNA polymerase sigma factor for flagellar operon FliA |
| K02777 | KSG66\_10355 | PTS-Glc-EIIA, crr; PTS system, sugar-specific IIA component [EC:2.7.1.-] |
| K03092 | KSG66\_16110 | rpoN; RNA polymerase sigma-54 factor |
| K03563 | KSG66\_16655 | csrA; carbon storage regulator |
| K03666 | KSG66\_08665 | hfq; host factor-I protein |
| K05946 | KSG66\_16835 | tagA, tarA; N-acetylglucosaminyldiphosphoundecaprenol N-acetyl-beta-D-mannosaminyltransferase [EC:2.4.1.187] |
| K07173 | KSG66\_14140 | luxS; S-ribosylhomocysteine lyase [EC:4.4.1.21] |

| 85 | Synthesis and degradation of ketone bodies | ko00072 || K00019 | KSG66\_09180 | E1.1.1.30, bdh; 3-hydroxybutyrate dehydrogenase [EC:1.1.1.30] |
| K00019 | KSG66\_19245 | E1.1.1.30, bdh; 3-hydroxybutyrate dehydrogenase [EC:1.1.1.30] |
| K00626 | KSG66\_05195 | E2.3.1.9, atoB; acetyl-CoA C-acetyltransferase [EC:2.3.1.9] |
| K00626 | KSG66\_11405 | E2.3.1.9, atoB; acetyl-CoA C-acetyltransferase [EC:2.3.1.9] |
| K01028 | KSG66\_09190 | E2.8.3.5A, scoA; 3-oxoacid CoA-transferase subunit A [EC:2.8.3.5] |
| K01029 | KSG66\_09185 | E2.8.3.5B, scoB; 3-oxoacid CoA-transferase subunit B [EC:2.8.3.5] |
| K01574 | KSG66\_15350 | adc; acetoacetate decarboxylase [EC:4.1.1.4] |
| K01640 | KSG66\_09255 | E4.1.3.4, HMGCL, hmgL; hydroxymethylglutaryl-CoA lyase [EC:4.1.3.4] |

| 86 | Photosynthesis | ko00195 || K02108 | KSG66\_17405 | ATPF0A, atpB; F-type H+-transporting ATPase subunit a |
| K02109 | KSG66\_17395 | ATPF0B, atpF; F-type H+-transporting ATPase subunit b |
| K02110 | KSG66\_17400 | ATPF0C, atpE; F-type H+-transporting ATPase subunit c |
| K02111 | KSG66\_17385 | ATPF1A, atpA; F-type H+/Na+-transporting ATPase subunit alpha [EC:7.1.2.2 7.2.2.1] |
| K02112 | KSG66\_17375 | ATPF1B, atpD; F-type H+/Na+-transporting ATPase subunit beta [EC:7.1.2.2 7.2.2.1] |
| K02113 | KSG66\_17390 | ATPF1D, atpH; F-type H+-transporting ATPase subunit delta |
| K02114 | KSG66\_17370 | ATPF1E, atpC; F-type H+-transporting ATPase subunit epsilon |
| K02115 | KSG66\_17380 | ATPF1G, atpG; F-type H+-transporting ATPase subunit gamma |

| 87 | Tyrosine metabolism | ko00350 || K00121 | KSG66\_01785 | frmA, ADH5, adhC; S-(hydroxymethyl)glutathione dehydrogenase / alcohol dehydrogenase [EC:1.1.1.284 1.1.1.1] |
| K00121 | KSG66\_02865 | frmA, ADH5, adhC; S-(hydroxymethyl)glutathione dehydrogenase / alcohol dehydrogenase [EC:1.1.1.284 1.1.1.1] |
| K00135 | KSG66\_02070 | gabD; succinate-semialdehyde dehydrogenase / glutarate-semialdehyde dehydrogenase [EC:1.2.1.16 1.2.1.79 1.2.1.20] |
| K00483 | KSG66\_01300 | hpaB; 4-hydroxyphenylacetate 3-monooxygenase [EC:1.14.14.9] |
| K00812 | KSG66\_10425 | aspB; aspartate aminotransferase [EC:2.6.1.1] |
| K00817 | KSG66\_10555 | hisC; histidinol-phosphate aminotransferase [EC:2.6.1.9] |
| K11358 | KSG66\_04815 | yhdR; aspartate aminotransferase [EC:2.6.1.1] |
| K13953 | KSG66\_08815 | adhP; alcohol dehydrogenase, propanol-preferring [EC:1.1.1.1] |

| 88 | Drug metabolism - other enzymes | ko00983 || K00088 | KSG66\_00070 | IMPDH, guaB; IMP dehydrogenase [EC:1.1.1.205] |
| K00760 | KSG66\_00415 | hprT, hpt, HPRT1; hypoxanthine phosphoribosyltransferase [EC:2.4.2.8] |
| K00857 | KSG66\_17500 | tdk, TK; thymidine kinase [EC:2.7.1.21] |
| K00876 | KSG66\_12510 | udk, UCK; uridine kinase [EC:2.7.1.48] |
| K00940 | KSG66\_10610 | ndk, NME; nucleoside-diphosphate kinase [EC:2.7.4.6] |
| K01489 | KSG66\_11980 | cdd, CDA; cytidine deaminase [EC:3.5.4.5] |
| K01520 | KSG66\_08885 | dut, DUT; dUTP pyrophosphatase [EC:3.6.1.23] |
| K01951 | KSG66\_03300 | guaA, GMPS; GMP synthase (glutamine-hydrolysing) [EC:6.3.5.2] |

| 89 | Biosynthesis of various secondary metabolites - part 2 | ko00998 || K02078 | KSG66\_07955 | acpP; acyl carrier protein |
| K02078 | KSG66\_11205 | acpP; acyl carrier protein |
| K13037 | KSG66\_17810 | bacD; L-alanine-L-anticapsin ligase [EC:6.3.2.49] |
| K19546 | KSG66\_17825 | bacA; prephenate decarboxylase [EC:4.1.1.100] |
| K19547 | KSG66\_17820 | bacB; 3-[(4R)-4-hydroxycyclohexa-1,5-dien-1-yl]-2-oxopropanoate isomerase [EC:5.3.3.19] |
| K19548 | KSG66\_17815 | bacC; dihydroanticapsin dehydrogenase [EC:1.1.1.385] |
| K19549 | KSG66\_17800 | bacF; bacilysin biosynthesis transaminase BacF [EC:2.6.1.-] |
| K19550 | KSG66\_17795 | bacG; bacilysin biosynthesis oxidoreductase BacG [EC:1.3.1.-] |

| 90 | Phenylalanine metabolism | ko00360 || K00074 | KSG66\_11400 | paaH, hbd, fadB, mmgB; 3-hydroxybutyryl-CoA dehydrogenase [EC:1.1.1.157] |
| K00285 | KSG66\_15175 | dadA; D-amino-acid dehydrogenase [EC:1.4.5.1] |
| K00812 | KSG66\_10425 | aspB; aspartate aminotransferase [EC:2.6.1.1] |
| K00817 | KSG66\_10555 | hisC; histidinol-phosphate aminotransferase [EC:2.6.1.9] |
| K00824 | KSG66\_04865 | dat; D-alanine transaminase [EC:2.6.1.21] |
| K11358 | KSG66\_04815 | yhdR; aspartate aminotransferase [EC:2.6.1.1] |
| K15866 | KSG66\_04965 | paaG; 2-(1,2-epoxy-1,2-dihydrophenyl)acetyl-CoA isomerase [EC:5.3.3.18] |

| 91 | beta-Alanine metabolism | ko00410 || K00128 | KSG66\_09600 | ALDH; aldehyde dehydrogenase (NAD+) [EC:1.2.1.3] |
| K00128 | KSG66\_13620 | ALDH; aldehyde dehydrogenase (NAD+) [EC:1.2.1.3] |
| K00128 | KSG66\_18800 | ALDH; aldehyde dehydrogenase (NAD+) [EC:1.2.1.3] |
| K00140 | KSG66\_18750 | mmsA, iolA, ALDH6A1; malonate-semialdehyde dehydrogenase (acetylating) / methylmalonate-semialdehyde dehydrogenase [EC:1.2.1.18 1.2.1.27] |
| K01579 | KSG66\_10445 | panD; aspartate 1-decarboxylase [EC:4.1.1.11] |
| K01918 | KSG66\_10450 | panC; pantoate--beta-alanine ligase [EC:6.3.2.1] |
| K07250 | KSG66\_02065 | gabT; 4-aminobutyrate aminotransferase / (S)-3-amino-2-methylpropionate transaminase / 5-aminovalerate transaminase [EC:2.6.1.19 2.6.1.22 2.6.1.48] |

| 92 | Taurine and hypotaurine metabolism | ko00430 || K00259 | KSG66\_14805 | ald; alanine dehydrogenase [EC:1.4.1.1] |
| K00260 | KSG66\_10725 | gudB, rocG; glutamate dehydrogenase [EC:1.4.1.2] |
| K00260 | KSG66\_17855 | gudB, rocG; glutamate dehydrogenase [EC:1.4.1.2] |
| K00625 | KSG66\_17785 | E2.3.1.8, pta; phosphate acetyltransferase [EC:2.3.1.8] |
| K00681 | KSG66\_09350 | ggt; gamma-glutamyltranspeptidase / glutathione hydrolase [EC:2.3.2.2 3.4.19.13] |
| K00925 | KSG66\_13520 | ackA; acetate kinase [EC:2.7.2.1] |
| K03851 | KSG66\_04675 | tpa; taurine-pyruvate aminotransferase [EC:2.6.1.77] |

| 93 | Chloroalkane and chloroalkene degradation | ko00625 || K00121 | KSG66\_01785 | frmA, ADH5, adhC; S-(hydroxymethyl)glutathione dehydrogenase / alcohol dehydrogenase [EC:1.1.1.284 1.1.1.1] |
| K00121 | KSG66\_02865 | frmA, ADH5, adhC; S-(hydroxymethyl)glutathione dehydrogenase / alcohol dehydrogenase [EC:1.1.1.284 1.1.1.1] |
| K00128 | KSG66\_09600 | ALDH; aldehyde dehydrogenase (NAD+) [EC:1.2.1.3] |
| K00128 | KSG66\_13620 | ALDH; aldehyde dehydrogenase (NAD+) [EC:1.2.1.3] |
| K00128 | KSG66\_18800 | ALDH; aldehyde dehydrogenase (NAD+) [EC:1.2.1.3] |
| K01560 | KSG66\_01210 | E3.8.1.2; 2-haloacid dehalogenase [EC:3.8.1.2] |
| K13953 | KSG66\_08815 | adhP; alcohol dehydrogenase, propanol-preferring [EC:1.1.1.1] |

| 94 | Biofilm formation - Escherichia coli | ko02026 || K02398 | KSG66\_16685 | flgM; negative regulator of flagellin synthesis FlgM |
| K02405 | KSG66\_08235 | fliA; RNA polymerase sigma factor for flagellar operon FliA |
| K02777 | KSG66\_10355 | PTS-Glc-EIIA, crr; PTS system, sugar-specific IIA component [EC:2.7.1.-] |
| K03563 | KSG66\_16655 | csrA; carbon storage regulator |
| K06204 | KSG66\_07715 | dksA; DnaK suppressor protein |
| K06204 | KSG66\_09555 | dksA; DnaK suppressor protein |
| K07173 | KSG66\_14140 | luxS; S-ribosylhomocysteine lyase [EC:4.4.1.21] |

| 95 | Ascorbate and aldarate metabolism | ko00053 || K00012 | KSG66\_16760 | UGDH, ugd; UDPglucose 6-dehydrogenase [EC:1.1.1.22] |
| K00012 | KSG66\_17100 | UGDH, ugd; UDPglucose 6-dehydrogenase [EC:1.1.1.22] |
| K00128 | KSG66\_09600 | ALDH; aldehyde dehydrogenase (NAD+) [EC:1.2.1.3] |
| K00128 | KSG66\_13620 | ALDH; aldehyde dehydrogenase (NAD+) [EC:1.2.1.3] |
| K00128 | KSG66\_18800 | ALDH; aldehyde dehydrogenase (NAD+) [EC:1.2.1.3] |
| K03077 | KSG66\_13225 | araD, ulaF, sgaE, sgbE; L-ribulose-5-phosphate 4-epimerase [EC:5.1.3.4] |

| 96 | D-Glutamine and D-glutamate metabolism | ko00471 || K01425 | KSG66\_01400 | glsA, GLS; glutaminase [EC:3.5.1.2] |
| K01425 | KSG66\_07425 | glsA, GLS; glutaminase [EC:3.5.1.2] |
| K01776 | KSG66\_13035 | murI; glutamate racemase [EC:5.1.1.3] |
| K01924 | KSG66\_13780 | murC; UDP-N-acetylmuramate--alanine ligase [EC:6.3.2.8] |
| K01925 | KSG66\_07610 | murD; UDP-N-acetylmuramoylalanine--D-glutamate ligase [EC:6.3.2.9] |
| K23265 | KSG66\_03370 |  |

| 97 | D-Alanine metabolism | ko00473 || K00824 | KSG66\_04865 | dat; D-alanine transaminase [EC:2.6.1.21] |
| K01775 | KSG66\_02475 | alr; alanine racemase [EC:5.1.1.1] |
| K01775 | KSG66\_11455 | alr; alanine racemase [EC:5.1.1.1] |
| K01921 | KSG66\_02435 | ddl; D-alanine-D-alanine ligase [EC:6.3.2.4] |
| K03367 | KSG66\_18200 | dltA; D-alanine--poly(phosphoribitol) ligase subunit 1 [EC:6.1.1.13] |
| K14188 | KSG66\_18210 | dltC; D-alanine--poly(phosphoribitol) ligase subunit 2 [EC:6.1.1.13] |

| 98 | Vitamin B6 metabolism | ko00750 || K00831 | KSG66\_05055 | serC, PSAT1; phosphoserine aminotransferase [EC:2.6.1.52] |
| K00868 | KSG66\_17985 | pdxK, pdxY; pyridoxine kinase [EC:2.7.1.35] |
| K01733 | KSG66\_14995 | thrC; threonine synthase [EC:4.2.3.1] |
| K06215 | KSG66\_00080 | pdxS, pdx1; pyridoxal 5'-phosphate synthase pdxS subunit [EC:4.3.3.6] |
| K08681 | KSG66\_00085 | pdxT, pdx2; 5'-phosphate synthase pdxT subunit [EC:4.3.3.6] |

| 99 | Biosynthesis of siderophore group nonribosomal peptides | ko01053 || K00216 | KSG66\_14840 | entA; 2,3-dihydro-2,3-dihydroxybenzoate dehydrogenase [EC:1.3.1.28] |
| K02361 | KSG66\_14835 | entC; isochorismate synthase [EC:5.4.4.2] |
| K02363 | KSG66\_14830 | entE, dhbE, vibE, mxcE; 2,3-dihydroxybenzoate-AMP ligase [EC:6.3.2.14 2.7.7.58] |
| K04780 | KSG66\_14820 | dhbF; nonribosomal peptide synthetase DhbF |
| K15652 | KSG66\_01700 | asbF; 3-dehydroshikimate dehydratase [EC:4.2.1.118] |

| 100 | RNA polymerase | ko03020 || K03040 | KSG66\_00865 | rpoA; DNA-directed RNA polymerase subunit alpha [EC:2.7.7.6] |
| K03043 | KSG66\_00685 | rpoB; DNA-directed RNA polymerase subunit beta [EC:2.7.7.6] |
| K03046 | KSG66\_00690 | rpoC; DNA-directed RNA polymerase subunit beta' [EC:2.7.7.6] |
| K03048 | KSG66\_17550 | rpoE; DNA-directed RNA polymerase subunit delta |
| K03060 | KSG66\_07840 | rpoZ; DNA-directed RNA polymerase subunit omega [EC:2.7.7.6] |

| 101 | Novobiocin biosynthesis | ko00401 || K00812 | KSG66\_10425 | aspB; aspartate aminotransferase [EC:2.6.1.1] |
| K00817 | KSG66\_10555 | hisC; histidinol-phosphate aminotransferase [EC:2.6.1.9] |
| K04517 | KSG66\_10550 | tyrA2; prephenate dehydrogenase [EC:1.3.1.12] |
| K11358 | KSG66\_04815 | yhdR; aspartate aminotransferase [EC:2.6.1.1] |

| 102 | Cyanoamino acid metabolism | ko00460 || K00600 | KSG66\_17420 | glyA, SHMT; glycine hydroxymethyltransferase [EC:2.1.2.1] |
| K00681 | KSG66\_09350 | ggt; gamma-glutamyltranspeptidase / glutathione hydrolase [EC:2.3.2.2 3.4.19.13] |
| K01424 | KSG66\_01505 | E3.5.1.1, ansA, ansB; L-asparaginase [EC:3.5.1.1] |
| K01424 | KSG66\_11025 | E3.5.1.1, ansA, ansB; L-asparaginase [EC:3.5.1.1] |

| 103 | Polyketide sugar unit biosynthesis | ko00523 || K00067 | KSG66\_17890 | rfbD, rmlD; dTDP-4-dehydrorhamnose reductase [EC:1.1.1.133] |
| K00973 | KSG66\_17900 | E2.7.7.24, rfbA, rffH; glucose-1-phosphate thymidylyltransferase [EC:2.7.7.24] |
| K01710 | KSG66\_17895 | E4.2.1.46, rfbB, rffG; dTDP-glucose 4,6-dehydratase [EC:4.2.1.46] |
| K01790 | KSG66\_17885 | rfbC, rmlC; dTDP-4-dehydrorhamnose 3,5-epimerase [EC:5.1.3.13] |

| 104 | Lipoic acid metabolism | ko00785 || K03644 | KSG66\_15030 | lipA; lipoyl synthase [EC:2.8.1.8] |
| K03800 | KSG66\_05155 | lplA, lplJ; lipoate---protein ligase [EC:6.3.1.20] |
| K16869 | KSG66\_17770 | lipL; octanoyl-[GcvH]:protein N-octanoyltransferase [EC:2.3.1.204] |
| K23734 | KSG66\_11610 |  |

| 105 | Biofilm formation - Pseudomonas aeruginosa | ko02025 || K01657 | KSG66\_10585 | trpE; anthranilate synthase component I [EC:4.1.3.27] |
| K02398 | KSG66\_16685 | flgM; negative regulator of flagellin synthesis FlgM |
| K02405 | KSG66\_08235 | fliA; RNA polymerase sigma factor for flagellar operon FliA |
| K03563 | KSG66\_16655 | csrA; carbon storage regulator |

| 106 | Plant-pathogen interaction | ko04626 || K00864 | KSG66\_04690 | glpK, GK; glycerol kinase [EC:2.7.1.30] |
| K02358 | KSG66\_00715 | tuf, TUFM; elongation factor Tu |
| K02406 | KSG66\_16650 | fliC; flagellin |
| K04079 | KSG66\_18770 | HSP90A, htpG; molecular chaperone HtpG |

| 107 | Penicillin and cephalosporin biosynthesis | ko00311 || K01060 | KSG66\_01715 | cah; cephalosporin-C deacetylase [EC:3.1.1.41] |
| K17836 | KSG66\_05960 | penP; beta-lactamase class A [EC:3.5.2.6] |
| K24041 | KSG66\_18320 |  |

| 108 | Carbapenem biosynthesis | ko00332 || K00147 | KSG66\_06570 | proA; glutamate-5-semialdehyde dehydrogenase [EC:1.2.1.41] |
| K00931 | KSG66\_06565 | proB; glutamate 5-kinase [EC:2.7.2.11] |
| K00931 | KSG66\_09395 | proB; glutamate 5-kinase [EC:2.7.2.11] |

| 109 | Naphthalene degradation | ko00626 || K00121 | KSG66\_01785 | frmA, ADH5, adhC; S-(hydroxymethyl)glutathione dehydrogenase / alcohol dehydrogenase [EC:1.1.1.284 1.1.1.1] |
| K00121 | KSG66\_02865 | frmA, ADH5, adhC; S-(hydroxymethyl)glutathione dehydrogenase / alcohol dehydrogenase [EC:1.1.1.284 1.1.1.1] |
| K13953 | KSG66\_08815 | adhP; alcohol dehydrogenase, propanol-preferring [EC:1.1.1.1] |

| 110 | Atrazine degradation | ko00791 || K01428 | KSG66\_17290 | ureC; urease subunit alpha [EC:3.5.1.5] |
| K01429 | KSG66\_17295 | ureB; urease subunit beta [EC:3.5.1.5] |
| K01430 | KSG66\_17300 | ureA; urease subunit gamma [EC:3.5.1.5] |

| 111 | Limonene and pinene degradation | ko00903 || K00128 | KSG66\_09600 | ALDH; aldehyde dehydrogenase (NAD+) [EC:1.2.1.3] |
| K00128 | KSG66\_13620 | ALDH; aldehyde dehydrogenase (NAD+) [EC:1.2.1.3] |
| K00128 | KSG66\_18800 | ALDH; aldehyde dehydrogenase (NAD+) [EC:1.2.1.3] |

| 112 | NOD-like receptor signaling pathway | ko04621 || K02406 | KSG66\_16650 | fliC; flagellin |
| K03671 | KSG66\_13085 | trxA; thioredoxin 1 |
| K04079 | KSG66\_18770 | HSP90A, htpG; molecular chaperone HtpG |

| 113 | Geraniol degradation | ko00281 || K00632 | KSG66\_15285 | fadA, fadI; acetyl-CoA acyltransferase [EC:2.3.1.16] |
| K01640 | KSG66\_09255 | E4.1.3.4, HMGCL, hmgL; hydroxymethylglutaryl-CoA lyase [EC:4.1.3.4] |

| 114 | Chlorocyclohexane and chlorobenzene degradation | ko00361 || K01560 | KSG66\_01210 | E3.8.1.2; 2-haloacid dehalogenase [EC:3.8.1.2] |
| K07104 | KSG66\_04115 | catE; catechol 2,3-dioxygenase [EC:1.13.11.2] |

| 115 | Other glycan degradation | ko00511 || K01201 | KSG66\_09750 | GBA, srfJ; glucosylceramidase [EC:3.2.1.45] |
| K23989 | KSG66\_16845 |  |

| 116 | Acarbose and validamycin biosynthesis | ko00525 || K00973 | KSG66\_17900 | E2.7.7.24, rfbA, rffH; glucose-1-phosphate thymidylyltransferase [EC:2.7.7.24] |
| K01710 | KSG66\_17895 | E4.2.1.46, rfbB, rffG; dTDP-glucose 4,6-dehydratase [EC:4.2.1.46] |

| 117 | Sphingolipid metabolism | ko00600 || K01201 | KSG66\_09750 | GBA, srfJ; glucosylceramidase [EC:3.2.1.45] |
| K07406 | KSG66\_13935 | melA; alpha-galactosidase [EC:3.2.1.22] |

| 118 | Xylene degradation | ko00622 || K01821 | KSG66\_17695 | praC, xylH; 4-oxalocrotonate tautomerase [EC:5.3.2.6] |
| K07104 | KSG66\_04115 | catE; catechol 2,3-dioxygenase [EC:1.13.11.2] |

| 119 | Non-homologous end-joining | ko03450 || K01971 | KSG66\_06685 | ligD; bifunctional non-homologous end joining protein LigD [EC:6.5.1.1] |
| K10979 | KSG66\_06690 | ku; DNA end-binding protein Ku |

| 120 | Phenazine biosynthesis | ko00405 | K01657 | KSG66\_10585 | trpE; anthranilate synthase component I [EC:4.1.3.27] |
| 121 | Phosphonate and phosphinate metabolism | ko00440 | K03823 | KSG66\_17250 | pat; phosphinothricin acetyltransferase [EC:2.3.1.183] |
| 122 | D-Arginine and D-ornithine metabolism | ko00472 | K00824 | KSG66\_04865 | dat; D-alanine transaminase [EC:2.6.1.21] |
| 123 | Neomycin, kanamycin and gentamicin biosynthesis | ko00524 | K00845 | KSG66\_11770 | glk; glucokinase [EC:2.7.1.2] |
| 124 | Arabinogalactan biosynthesis - Mycobacterium | ko00572 | K02851 | KSG66\_16735 | wecA, tagO, rfe; UDP-GlcNAc:undecaprenyl-phosphate/decaprenyl-phosphate GlcNAc-1-phosphate transferase [EC:2.7.8.33 2.7.8.35] |
| 125 | Arachidonic acid metabolism | ko00590 | K00432 | KSG66\_10165 | gpx; glutathione peroxidase [EC:1.11.1.9] |
| 126 | alpha-Linolenic acid metabolism | ko00592 | K00632 | KSG66\_15285 | fadA, fadI; acetyl-CoA acyltransferase [EC:2.3.1.16] |
| 127 | Dioxin degradation | ko00621 | K01821 | KSG66\_17695 | praC, xylH; 4-oxalocrotonate tautomerase [EC:5.3.2.6] |
| 128 | Ethylbenzene degradation | ko00642 | K00632 | KSG66\_15285 | fadA, fadI; acetyl-CoA acyltransferase [EC:2.3.1.16] |
| 129 | Styrene degradation | ko00643 | K07104 | KSG66\_04115 | catE; catechol 2,3-dioxygenase [EC:1.13.11.2] |
| 130 | Carotenoid biosynthesis | ko00906 | K02291 | KSG66\_05400 | crtB; 15-cis-phytoene synthase [EC:2.5.1.32] |
| 131 | Biosynthesis of ansamycins | ko01051 | K00615 | KSG66\_08935 | E2.2.1.1, tktA, tktB; transketolase [EC:2.2.1.1] |
| 132 | Biosynthesis of vancomycin group antibiotics | ko01055 | K01710 | KSG66\_17895 | E4.2.1.46, rfbB, rffG; dTDP-glucose 4,6-dehydratase [EC:4.2.1.46] |

Back Top
