# Supplementary material for: Isolation, identification, and whole-genome sequencing of high-yield protease bacteria from Daqu of ZhangGong Laojiu
Source: PLoS One. 2022 Apr 26;17(4):e0264677. doi: 10.1371/journal.pone.0264677 (PMC9041807; doi:10.1371/journal.pone.0264677)
Supplement: S1 Raw data — (ZIP) [file pone.0264677.s002.zip › Raw data/GDD20120317-1_Bacillus_velezensis_Genome_result/4_Basic_Annot/KEGG/Bac_map/map00333.html]

KEGG PATHWAY: Prodigiosin biosynthesis - Reference pathway


|  |  |
| --- | --- |
| **Prodigiosin biosynthesis - Reference pathway** |  |

[
Pathway menu
| Organism menu
| Pathway entry
| Show description
| User data mapping
]

|  |
| --- |
| Prodiginines are red-pigmented natural antibiotics that are produced as secondary metabolites and have received renewed attention because of potential clinical interests. In Serratia sp. or actinomycetes such as Streptomyces coelicolor, the tripyrrole molecules of prodigiosin and undecylprodigiosin are formed by the condensation of 4-methoxy-2,2'-bipyrrole-5-carbaldehyde and either 2-methyl-3-n-amyl-pyrrole [MD:M00837] or 2-undecylpyrrole [MD:M00838]. The Serratia pig gene cluster and the Streptomyces red gene cluster responsible for these biosynthesis pathways have been identified. |

|  |  |
| --- | --- |
| Reference pathway Reference pathway (KO) Reference pathway (EC) Reference pathway (Reaction) -----< Sort below by alphabet >----- Serratia marcescens WW4 Serratia plymuthica AS9 Serratia sp. AS12 Serratia sp. AS13 Serratia sp. FS14 Serratia sp. ATCC 39006 Serratia sp. ATCC 39006 Serratia sp. MYb239 Vibrio gazogenes Pseudoalteromonas rubra Pseudoalteromonas tunicata Hahella chejuensis Hahella sp. KA22 Cupriavidus sp. USMAHM13 Pseudohalocynthiibacter aestuariivivens Nocardia terpenica Streptomyces coelicolor Streptomyces lividans Streptomyces sp. CCM\_MD2014 Streptomyces alfalfae Streptomyces alboflavus Nocardiopsis gilva | 184% 150% 122% 100% 82% 67% 55% |
