# Supplementary material for: Isolation, identification, and whole-genome sequencing of high-yield protease bacteria from Daqu of ZhangGong Laojiu
Source: PLoS One. 2022 Apr 26;17(4):e0264677. doi: 10.1371/journal.pone.0264677 (PMC9041807; doi:10.1371/journal.pone.0264677)
Supplement: S1 Raw data — (ZIP) [file pone.0264677.s002.zip › Raw data/GDD20120317-1_Bacillus_velezensis_Genome_result/4_Basic_Annot/KEGG/Bac_map/map00405.html]

KEGG PATHWAY: Phenazine biosynthesis - Reference pathway


|  |  |
| --- | --- |
| **Phenazine biosynthesis - Reference pathway** |  |

[
Pathway menu
| Organism menu
| Pathway entry
| User data mapping
]

|  |  |
| --- | --- |
| Reference pathway Reference pathway (KO) Reference pathway (EC) Reference pathway (Reaction) -----< Sort below by alphabet >----- Enterobacter cloacae subsp. cloacae ENHKU01 Enterobacter cloacae ECNIH2 Enterobacter cloacae ECNIH4 Enterobacter hormaechei CAV1176 Enterobacter asburiae L1 Enterobacter kobei Enterobacter ludwigii EN-119 Enterobacter chengduensis Enterobacter soli Enterobacter sp. ODB01 Cronobacter muytjensii Klebsiella oxytoca CAV1374 Raoultella ornithinolytica B6 Raoultella planticola Raoultella sp. X13 Cedecea neteri M006 Leclercia adecarboxylata Leclercia sp. LSNIH3 Metakosakonia sp. MRY16-398 Enterobacteriaceae bacterium FGI 57 Enterobacteriaceae bacterium ENNIH2 Serratia sp. FGI94 Serratia fonticola DSM 4576 Serratia sp. MYb239 Pectobacterium atrosepticum SCRI1043 Pectobacterium atrosepticum JG10-08 Pectobacterium atrosepticum 21A Pantoea ananatis AJ13355 Pantoea sp. At-9b Xenorhabdus bovienii CS03 Xenorhabdus doucetiae Xenorhabdus poinarii Xenorhabdus hominickii Phytobacter ursingii Glaesserella sp. 15-184 Pasteurella multocida ATCC 43137 Pasteurella dagmatis Mannheimia haemolytica 89010807N Mannheimia haemolytica 89010807N lktA- Mannheimia sp. USDA-ARS-USMARC-1261 Mannheimia varigena USDA-ARS-USMARC-1296 Mannheimia varigena USDA-ARS-USMARC-1388 Mannheimia sp. ZY170218 Actinobacillus porcitonsillarum Lysobacter antibioticus 76 Lysobacter antibioticus ATCC 29479 Pseudolysobacter antarcticus Vibrio mimicus Pseudomonas aeruginosa PAO1 Pseudomonas aeruginosa PAO1-VE13 Pseudomonas aeruginosa PAO1-VE2 Pseudomonas aeruginosa UCBPP-PA14 Pseudomonas aeruginosa PA7 Pseudomonas aeruginosa LESB58 Pseudomonas aeruginosa M18 Pseudomonas aeruginosa NCGM2.S1 Pseudomonas aeruginosa NCGM 1900 Pseudomonas aeruginosa DK2 Pseudomonas aeruginosa B136-33 Pseudomonas aeruginosa RP73 Pseudomonas aeruginosa PA1 Pseudomonas aeruginosa PA1R Pseudomonas aeruginosa MTB-1 Pseudomonas aeruginosa LES431 Pseudomonas aeruginosa SCV20265 Pseudomonas aeruginosa PA38182 Pseudomonas aeruginosa YL84 Pseudomonas aeruginosa c7447m Pseudomonas aeruginosa PAO581 Pseudomonas citronellolis Pseudomonas synxantha LBUM223 Pseudomonas orientalis Pseudomonas knackmussii Pseudomonas chlororaphis PA23 Pseudomonas chlororaphis subsp. aurantiaca Pseudomonas sp. TCU-HL1 Pseudomonas yamanorum Moraxella osloensis Moraxellaceae bacterium HYN0046 Shewanella violacea Shewanella psychrophila Catenovulum sp. CCB-QB4 Spongiibacter sp. IMCC21906 Zhongshania aliphaticivorans Microbulbifer hydrolyticus Methylomonas methanica Methylomonas denitrificans Methylomonas sp. DH-1 Methylomonas koyamae Methylomonas sp. LW13 Methylomicrobium alcaliphilum Methylomicrobium buryatense Methylovulum psychrotolerans Cycloclasticus sp. P1 Cycloclasticus zancles Cycloclasticus sp. PY97N Woeseia oceani Granulosicoccus antarcticus Halomonas elongata Halomonas campaniensis Halomonas sp. KO116 Halomonas sp. R57-5 Halomonas huangheensis Halomonas chromatireducens Halomonas hydrothermalis Halomonas beimenensis Halomonas alkaliphila Halomonas venusta Halomonas olivaria Halomonas sulfidaeris Halotalea alkalilenta Kushneria konosiri Kushneria marisflavi Pistricoccus aurantiacus Alcanivorax borkumensis Alcanivorax sp. NBRC 101098 Marinomonas mediterranea Marinomonas posidonica Marinobacterium aestuarii Neptunomonas concharum Nitrincola sp. KXZD1103 Oleiphilus messinensis Sinimarinibacterium sp. NLF-5-8 Immundisolibacter cernigliae Sulfuricaulis limicola Sulfurifustis variabilis Salinisphaera sp. LB1 Neisseria weaveri Neisseria chenwenguii Neisseria animalis Chromobacterium vaccinii Chromobacterium sp. ATCC 53434 Chromobacterium rhizoryzae Chromobacterium sp. 257-1 Iodobacter sp. H11R3 Jeongeupia sp. USM3 Aquaspirillum sp. LM1 Aquitalea magnusonii Aquitalea sp. USM4 Ralstonia solanacearum CFBP2957 Ralstonia solanacearum PSI07 Ralstonia solanacearum Po82 Ralstonia solanacearum UY031 Ralstonia pickettii 12J Ralstonia pickettii 12D Ralstonia pickettii DTP0602 Ralstonia mannitolilytica Ralstonia insidiosa Ralstonia pseudosolanacearum Cupriavidus necator H16 Cupriavidus necator N-1 Cupriavidus necator NH9 Cupriavidus taiwanensis Cupriavidus basilensis Cupriavidus gilardii Cupriavidus sp. USMAHM13 Cupriavidus malaysiensis Cupriavidus sp. USMAA2-4 Cupriavidus pauculus Cupriavidus oxalaticus Burkholderia mallei ATCC 23344 Burkholderia mallei SAVP1 Burkholderia mallei NCTC 10229 Burkholderia mallei NCTC 10247 Burkholderia mallei 23344 Burkholderia mallei 6 Burkholderia mallei BMQ Burkholderia mallei 2000031063 Burkholderia mallei FMH 23344 Burkholderia mallei NCTC 10247 Burkholderia mallei 2002734299 Burkholderia pseudomallei K96243 Burkholderia pseudomallei 1710b Burkholderia pseudomallei 1106a Burkholderia pseudomallei 668 Burkholderia pseudomallei MSHR305 Burkholderia pseudomallei MSHR511 Burkholderia pseudomallei MSHR146 Burkholderia pseudomallei MSHR520 Burkholderia pseudomallei 1026b Burkholderia pseudomallei BPC006 Burkholderia pseudomallei NCTC 13179 Burkholderia pseudomallei HBPUB10134a Burkholderia pseudomallei NAU35A-3 Burkholderia pseudomallei A79A Burkholderia pseudomallei TSV202 Burkholderia thailandensis E264 Burkholderia thailandensis 2002721723 Burkholderia thailandensis E444 Burkholderia thailandensis H0587 Burkholderia thailandensis MSMB121 Burkholderia thailandensis MSMB59 Burkholderia thailandensis E254 Burkholderia thailandensis USAMRU Malaysia #20 Burkholderia thailandensis 2003015869 Burkholderia thailandensis 2002721643 Burkholderia oklahomensis E0147 Burkholderia oklahomensis C6786 Burkholderia sp. BDU6 Burkholderia lata Burkholderia cenocepacia DDS 22E-1 Burkholderia cenocepacia H111 Burkholderia ambifaria AMMD Burkholderia ambifaria MC40-6 Burkholderia cepacia DDS 7H-2 Burkholderia cepacia ATCC 25416 Burkholderia pyrrocinia Burkholderia contaminans Burkholderia ubonensis Burkholderia seminalis Burkholderia stagnalis Burkholderia stabilis Burkholderia glumae BGR1 Burkholderia glumae LMG 2196 = ATCC 33617 Burkholderia sp. 2002721687 Burkholderia plantarii PG1 Burkholderia plantarii ATCC 43733 Burkholderia sp. Bp5365 Burkholderia sp. OLGA172 Burkholderia sp. PAMC 26561 Burkholderia sp. PAMC 28687 Paraburkholderia hospita Paraburkholderia caffeinilytica Pandoraea pnomenusa RB38 Pandoraea pnomenusa DSM 16536 Pandoraea pulmonicola Pandoraea sputorum Pandoraea apista Pandoraea vervacti Pandoraea oxalativorans Pandoraea thiooxydans Pandoraea faecigallinarum Pandoraea norimbergensis Pandoraea sp. XY-2 Pandoraea fibrosis Ephemeroptericola cinctiostellae Caballeronia sp. SBC2 Bordetella pertussis B1917 Bordetella pertussis 137 Bordetella bronchiseptica S798 Bordetella petrii Bordetella holmesii ATCC 51541 Bordetella holmesii 44057 Bordetella trematum Bordetella bronchialis Bordetella flabilis Bordetella pseudohinzii Bordetella sp. H567 Bordetella genomosp. 13 Achromobacter xylosoxidans A8 Achromobacter xylosoxidans NH44784-1996 Achromobacter xylosoxidans NBRC 15126 = ATCC 27061 Achromobacter xylosoxidans NCTC10807 Achromobacter denitrificans Achromobacter insolitus Achromobacter spanius Achromobacter sp. AONIH1 Achromobacter sp. B7 Pusillimonas sp. T7-7 Pusillimonas sp. ye3 Orrella dioscoreae Pigmentiphaga sp. H8 Rhodoferax sp. CHu59-6-5 Polaromonas sp. JS666 Polaromonas sp. SP1 Polaromonas sp. Pch-P Acidovorax citrulli Acidovorax avenae Acidovorax sp. KKS102 Acidovorax sp. RAC01 Acidovorax sp. 1608163 Delftia acidovorans Delftia sp. Cs1-4 Delftia tsuruhatensis Delftia sp. HK171 Variovorax paradoxus S110 Variovorax paradoxus EPS Variovorax paradoxus B4 Variovorax boronicumulans Variovorax sp. PMC12 Ramlibacter tataouinensis Limnohabitans sp. 103DPR2 Hydrogenophaga sp. RAC07 Hydrogenophaga sp. PBC Janthinobacterium sp. B9-8 Herbaspirillum seropedicae Z67 Herbaspirillum rubrisubalbicans Herbaspirillum huttiense Collimonas arenae Collimonas pratensis Roseateles depolymerans Paucibacter sp. KCTC 42545 Mitsuaria sp. 7 Rhizobacter gummiphilus Thauera sp. K11 Betaproteobacteria bacterium GR16-43 Nitratiruptor sp. SB155-2 Geobacter metallireducens Desulfovibrio ferrophilus Desulfococcus oleovorans Desulfococcus multivorans Desulfatibacillum alkenivorans Desulfosarcina ovata Desulfosarcina widdelii Sorangium cellulosum So ce56 Sorangium cellulosum So0157-2 Chondromyces crocatus Haliangium ochraceum Desulfomonile tiedjei Desulfarculus baarsii Mesorhizobium loti NZP2037 Mesorhizobium ciceri (biovar Biserrulae) Mesorhizobium opportunistum Mesorhizobium sp. B7 Mesorhizobium sp. WSM1497 Mesorhizobium sp. M9A.F.Ca.ET.002.03.1.2 Mesorhizobium sp. Pch-S Chelativorans sp. BNC1 Hoeflea sp. IMCC20628 Aminobacter aminovorans Neorhizobium galegae bv. officinalis bv. officinalis HAMBI 1141 Neorhizobium galegae bv. orientalis HAMBI 540 Neorhizobium sp. SOG26 Brucella suis bv. 2 Bs143CITA Brucella suis bv. 2 PT09143 Brucella suis bv. 2 PT09172 Brucella suis bv. 2 Bs364CITA Ochrobactrum anthropi ATCC 49188 Ochrobactrum anthropi OAB Bradyrhizobium diazoefficiens USDA 110 Bradyrhizobium japonicum USDA 6 Bradyrhizobium japonicum E109 Bradyrhizobium sp. ORS 278 Bradyrhizobium sp. BTAi1 Bradyrhizobium sp. S23321 Bradyrhizobium oligotrophicum Bradyrhizobium sp. CCGE-LA001 Bradyrhizobium sp. BF49 Bradyrhizobium icense Bradyrhizobium sp. ORS 285 Bradyrhizobium sp. SK17 Bradyrhizobium ottawaense Bradyrhizobium amphicarpaeae Bradyrhizobium guangdongense Bradyrhizobium guangzhouense Bradyrhizobium symbiodeficiens Bradyrhizobium betae Rhodopseudomonas palustris CGA009 Rhodopseudomonas palustris HaA2 Rhodopseudomonas palustris TIE-1 Rhodopseudomonas palustris DX-1 Bosea sp. PAMC 26642 Bosea sp. RAC05 Bosea vaviloviae Bosea sp. Tri-49 Bosea sp. F3-2 Variibacter gotjawalensis Xanthobacter autotrophicus Azorhizobium caulinodans Starkeya novella Labrys neptuniae Ancylobacter sp. TS-1 Ancylobacter pratisalsi Methylorubrum extorquens AM1 Methylorubrum extorquens DM4 Methylorubrum extorquens PA1 Methylorubrum extorquens CM4 Methylorubrum populi Methylorubrum zatmanii Methylobacterium radiotolerans Methylobacterium sp. 4-46 Methylobacterium nodulans Methylobacterium oryzae Methylobacterium sp. AMS5 Methylobacterium aquaticum Methylobacterium phyllosphaerae Methylobacterium currus Methylobacterium sp. DM1 Methylobacterium sp. XJLW Methylobacterium sp. 17Sr1-43 Methylobacterium mesophilicum Methylobacterium terrae Microvirga ossetica Microvirga sp. 17 mud 1-3 Microvirga sp. HR1 Methylovirgula ligni Beijerinckiaceae bacterium RH AL1 Chelatococcus sp. CO-6 Chelatococcus daeguensis Hyphomicrobium denitrificans ATCC 51888 Hyphomicrobium denitrificans 1NES1 Hyphomicrobium sp. MC1 Hyphomicrobium nitrativorans Rhodomicrobium vannielii Pelagibacterium halotolerans Candidatus Filomicrobium marinum W Candidatus Filomicrobium marinum Y Devosia sp. H5989 Devosia sp. I507 Devosia ginsengisoli Blastochloris viridis Rhodoplanes sp. Z2-YC6860 Maritalea myrionectae Youhaiella tibetensis Methylocystis heyeri Methylocystis parvus Methylosinus trichosporium Breoghania sp. L-A4 Methyloceanibacter caenitepidi Pseudorhodoplanes sinuspersici Nordella sp. HKS 07 Rhizobiales bacterium NRL2 Caulobacteraceae bacterium Ruegeria pomeroyi Ruegeria sp. TM1040 Ruegeria sp. AD91A Ruegeria sp. THAF33 Epibacterium mobile Rhodobacter blasticus Jannaschia sp. CCS1 Paracoccus zhejiangensis Paracoccus sp. BM15 Paracoccus sp. SC2-6 Paracoccus sp. Arc7-R13 Dinoroseobacter shibae Phaeobacter inhibens DSM 17395 Phaeobacter inhibens 2.10 Phaeobacter gallaeciensis DSM 26640 Phaeobacter porticola Phaeobacter piscinae Phaeobacter sp. LSS9 Leisingera methylohalidivorans Leisingera sp. NJS204 Leisingera aquaemixtae Roseibacterium elongatum Planktomarina temperata Celeribacter indicus Celeribacter ethanolicus Confluentimicrobium sp. EMB200-NS6 Pannonibacter phragmitetus Yangia sp. CCB-MM3 Yangia pacifica Sulfitobacter sp. AM1-D1 Sulfitobacter pseudonitzschiae Sulfitobacter sp. JL08 Sulfitobacter sp. D7 Tateyamaria omphalii Pelagibaca abyssi Roseovarius indicus Antarctobacter heliothermus Sagittula sp. P11 Thalassococcus sp. S3 Silicimonas algicola Sedimentitalea sp. W43 Boseongicola sp. CCM32 Pseudorhodobacter sp. S12M18 Litoreibacter sp. LN3S51 Oceanicola sp. D3 Maribius sp. THAF1 Roseivivax sp. THAF197b Paraoceanicella profunda Stappia indica Halovulum dunhuangense Rhodobacteraceae bacterium SH-1 Maricaulis maris Novosphingobium aromaticivorans Novosphingobium sp. THN1 Sphingomonas wittichii Sphingomonas wittichii DC-6 Sphingomonas hengshuiensis Sphingomonas sp. NIC1 Sphingomonas panacis Sphingomonas koreensis Sphingomonas sp. Cra20 Sphingosinicella sp. BN140058 Tardibacter chloracetimidivorans Sphingosinithalassobacter sp. zrk23 Altererythrobacter sp. B11 Gluconobacter oxydans H24 Gluconobacter thailandicus Acidiphilium cryptum Acidiphilium multivorum Roseomonas sp. FDAARGOS\_362 Roseomonas mucosa Swingsia samuiensis Tistrella mobilis Thalassospira indica Ferrovibrio terrae Polymorphum gilvum Phreatobacter cathodiphilus Phreatobacter stygius Candidatus Puniceispirillum marinum Bacillus licheniformis ATCC 14580 Bacillus licheniformis DSM 13 = ATCC 14580 Bacillus paralicheniformis Bacillus halodurans Bacillus anthracis A16 Bacillus anthracis A16R Bacillus anthracis SVA11 Bacillus anthracis HYU01 Bacillus anthracis Vollum Bacillus thuringiensis HD1011 Bacillus pseudomycoides 219298 Bacillus megaterium QM B1551 Bacillus megaterium DSM 319 Bacillus megaterium WSH-002 Bacillus megaterium NBRC 15308 = ATCC 14581 Bacillus sp. 1NLA3E Bacillus filamentosus Bacillus oceanisediminis Bacillus krulwichiae Oceanobacillus sp. 160 Anoxybacillus sp. PDR2 Lysinibacillus fusiformis Lysinibacillus sp. YS11 Lentibacillus amyloliquefaciens Brevibacillus formosus Paenibacillus sp. JDR-2 Paenibacillus sp. FSL R7-0331 Cohnella candidum Tumebacillus avium Tumebacillus algifaecis Planococcus sp. PAMC 21323 Planococcus kocurii Planococcus antarcticus Planococcus donghaensis Planococcus halocryophilus Planococcus maritimus Planococcus faecalis Paenisporosarcina antarctica Planomicrobium sp. Y50 Clostridium kluyveri DSM 555 Clostridium kluyveri NBRC 12016 Geosporobacter ferrireducens Syntrophothermus lipocalidus Desulfitobacterium metallireducens Desulfallas gibsoniae Sulfobacillus acidophilus TPY Sulfobacillus acidophilus DSM 10332 Sulfobacillus thermotolerans Christensenella minuta Pelosinus sp. UFO1 Pelosinus fermentans Sporomusa termitida Mycobacterium sp. JS623 Mycobacterium sp. VKM Ac-1817D Mycobacterium sp. NRRL B-3805 Mycobacterium dioxanotrophicus Mycolicibacterium smegmatis MC2 155 Mycolicibacterium smegmatis MC2 155 Mycolicibacterium smegmatis MC2 155 Mycolicibacterium smegmatis INHR1 Mycolicibacterium smegmatis INHR2 Mycolicibacterium neoaurum Mycolicibacterium goodii Mycolicibacterium fortuitum Mycobacteroides abscessus ATCC 19977 Mycobacteroides abscessus subsp. massiliense GO 06 Mycobacteroides abscessus subsp. bolletii 50594 Mycobacteroides abscessus subsp. massiliense CCUG 48898 = JCM 15300 Mycobacteroides chelonae Mycobacteroides immunogenum Mycobacterium stephanolepidis Mycobacteroides saopaulense Mycobacteroides salmoniphilum Corynebacterium glyciniphilum Corynebacterium mustelae Nocardia cyriacigeorgica Nocardia brasiliensis Nocardia nova Nocardia terpenica Nocardia sp. CS682 Rhodococcus erythropolis PR4 Rhodococcus fascians Rhodococcus sp. PBTS 2 Tsukamurella paurometabola Streptomyces coelicolor Streptomyces albidoflavus Streptomyces avermitilis Streptomyces griseus Streptomyces globisporus Streptomyces scabiei Streptomyces sp. SirexAA-E Streptomyces violaceusniger Streptomyces cattleya NRRL 8057 = DSM 46488 Streptomyces cattleya NRRL 8057 = DSM 46488 Streptomyces pratensis Streptomyces bingchenggensis Streptomyces hygroscopicus subsp. jinggangensis 5008 Streptomyces hygroscopicus subsp. jinggangensis TL01 Streptomyces venezuelae Streptomyces davaonensis Streptomyces albus DSM 41398 Streptomyces sp. PAMC 26508 Streptomyces fulvissimus Streptomyces collinus Streptomyces rapamycinicus Streptomyces albulus ZPM Streptomyces lividans Streptomyces glaucescens Streptomyces vietnamensis Streptomyces sp. 769 Streptomyces cyaneogriseus Streptomyces lydicus A02 Streptomyces lydicus 103 Streptomyces xiamenensis Streptomyces sp. Mg1 Streptomyces sp. CNQ-509 Streptomyces ambofaciens Streptomyces pristinaespiralis Streptomyces sp. CFMR 7 Streptomyces sp. CdTB01 Streptomyces reticuli Streptomyces sp. 4F Streptomyces leeuwenhoekii Streptomyces rubrolavendulae Streptomyces parvulus Streptomyces sp. SAT1 Streptomyces clavuligerus Streptomyces griseochromogenes Streptomyces qaidamensis Streptomyces lincolnensis Streptomyces noursei Streptomyces pluripotens Streptomyces sp. CCM\_MD2014 Streptomyces niveus Streptomyces autolyticus Streptomyces alfalfae Streptomyces violaceoruber Streptomyces fodineus Streptomyces gilvosporeus Streptomyces malaysiensis Streptomyces laurentii Streptomyces alboflavus Streptomyces albireticuli Streptomyces lavendulae Streptomyces sp. MOE7 Streptomyces formicae Streptomyces griseorubiginosus Streptomyces rochei Streptomyces lunaelactis Streptomyces koyangensis Kitasatospora setae Kitasatospora albolonga Kitasatospora aureofaciens Kitasatospora sp. MMS16-BH015 Streptacidiphilus sp. DSM 106435 Microbacterium sp. 1.5R Microbacterium foliorum Rathayibacter toxicus WAC3373 Curtobacterium sp. MR\_MD2014 Curtobacterium sp. BH-2-1-1 Curtobacterium sp. SGAir0471 Curtobacterium flaccumfaciens Microterricola viridarii Frondihabitans sp. PAMC 28766 Agromyces aureus Agromyces sp. MF30-A Cnuibacter physcomitrellae Salinibacterium sp. CGMCC 1.16371 Salinibacterium sp. dk2585 Humibacter sp. BT305 Humibacter sp. WJ7-1 Gryllotalpicola sp. 2DFW10M-5 Plantibacter sp. PA-3-X8 Leucobacter sp. DSM 101948 Leucobacter triazinivorans Arthrobacter sp. Hiyo8 Arthrobacter sp. PGP41 Dermacoccus abyssi Luteipulveratus mongoliensis Isoptericola dokdonensis Cellulomonas flavigena Cellulomonas sp. PSBB021 Cellulomonas sp. H30R-01 Arsenicicoccus sp. oral taxon 190 Tetrasphaera sp. HKS02 Brevibacterium sp. CS2 Dermatophilus congolensis Tessaracoccus flavescens Nocardioides sp. JS614 Nocardioides dokdonensis Nocardioides sp. MMS17-SY117 Nocardioides sp. dk3136 Nocardioides sp. S-1144 Nocardioides daphniae Pimelobacter simplex Nocardiopsis dassonvillei Streptomonospora sp. M2 Streptosporangium roseum Nonomuraea sp. ATCC 55076 Nonomuraea sp. WYY166 Frankia sp. EAN1pec Frankia alni Geodermatophilus obscurus Blastococcus saxobsidens Modestobacter marinus Amycolatopsis sp. AA4 Amycolatopsis sp. YIM 10 Pseudonocardia dioxanivorans Pseudonocardia sp. EC080625-04 Pseudonocardia sp. HH130629-09 Pseudonocardia sp. EC080610-09 Pseudonocardia sp EC080619-01 Pseudonocardia sp. HH130630-07 Pseudonocardia autotrophica Actinosynnema pretiosum Saccharothrix espanaensis Saccharothrix syringae Kutzneria albida Kibdelosporangium phytohabitans Actinoalloteichus sp. AHMU CJ021 Alloactinosynnema sp. L-07 Prauserella marina Salinispora tropica Salinispora arenicola Micromonospora aurantiaca Micromonospora sp. L5 Micromonospora sp. B006 Micromonospora tulbaghiae Micromonospora sp. HM134 Micromonospora terminaliae Verrucosispora maris Actinoplanes sp. SE50/110 Actinoplanes missouriensis Actinoplanes sp. N902-109 Actinoplanes friuliensis Actinoplanes sp. SE50 Plantactinospora sp. KBS50 Plantactinospora sp. BC1 Plantactinospora sp. BB1 Catenulispora acidiphila Stackebrandtia nassauensis Actinomyces sp. Chiba101 Bifidobacterium thermophilum Actinopolyspora erythraea Ilumatobacter coccineus Synechocystis sp. PCC 6803 Synechocystis sp. PCC 6803 Synechocystis sp. PCC 6803 GT-S Synechocystis sp. PCC 6803 GT-I Synechocystis sp. PCC 6803 PCC-N Synechocystis sp. PCC 6803 PCC-P Synechocystis sp. PCC 6714 Synechocystis sp. IPPAS B-1465 Synechococcus sp. PCC7002 Synechococcus sp. PCC 73109 Synechococcus sp. PCC 7003 Cyanobium gracile Dactylococcopsis salina Chamaesiphon minutus Leptolyngbya sp. PCC 7376 Leptolyngbya sp. NIES-3755 Leptolyngbya boryana Gloeocapsa sp. PCC 7428 Geminocystis sp. NIES-3709 Geminocystis sp. NIES-3708 Microcystis aeruginosa Microcystis panniformis Microcystis sp. MC19 Microcystis viridis Cyanobacterium aponinum Cyanobacterium stanieri Cyanobacterium sp. HL-69 Halothece sp. PCC 7418 Euhalothece natronophila Crocosphaera subtropica Crocosphaera watsonii Rippkaea orientalis PCC 8801 Rippkaea orientalis PCC 8802 Gloeothece citriformis Gloeothece verrucosa Trichodesmium erythraeum Arthrospira platensis Planktothrix agardhii Geitlerinema sp. PCC 7407 Oscillatoria acuminata Oscillatoria nigro-viridis Crinalium epipsammum Gloeobacter kilaueensis Fischerella sp. NIES-3754 Pleurocapsa sp. PCC 7327 Cyanobacterium endosymbiont of Epithemia turgida Cyanobacterium endosymbiont of Rhopalodia gibberula Chloroflexus aurantiacus Chloroflexus aurantiacus Y-400-fl Sphaerobacter thermophilus Caldilinea aerophila Ktedonobacterales bacterium SCAWS-G2 Tepidiforma bonchosmolovskayae Deinococcus maricopensis Truepera radiovictrix Candidatus Koribacter versatilis Acidobacterium capsulatum Granulicella tundricola Granulicella mallensis Granulicella sp. WH15 Terriglobus saanensis Terriglobus roseus Terriglobus albidus Acidisarcina polymorpha Edaphobacter sp. 12200R-103 Gemmatimonas aurantiaca Gemmatimonas phototrophica Gemmatirosa kalamazoonesis Panacibacter ginsenosidivorans Haliscomenobacter hydrossis Mucilaginibacter ginsenosidivorax Spirosoma linguale Spirosoma radiotolerans Spirosoma montaniterrae Spirosoma pollinicola Runella slithyformis Runella sp. HYN0085 Runella sp. SP2 Emticicia oligotrophica Fibrella aestuarina Fibrella sp. ES10-3-2-2 Hymenobacter swuensis Hymenobacter sp. APR13 Hymenobacter sedentarius Hymenobacter sp. sh-6 Hymenobacter sp. 17J68-5 Rufibacter sp. DG31D Rufibacter tibetensis Rufibacter sp. DG15C Nibribacter sp. BT10 Marivirga tractuosa Flammeovirga sp. MY04 Chryseolinea sp. KIS68-18 Flavobacterium johnsoniae UW101 Flavobacterium anhuiense Flavobacterium gilvum Chryseobacterium sp. StRB126 Flavobacteriaceae bacterium UJ101 Thermosulfidibacter takaii Nitrospira defluvii Nitrospira moscoviensis Candidatus Nitrospira inopinata Nitrospira japonica Ferroglobus placidus Geoglobus acetivorans Geoglobus ahangari Methanocella paludicola Methanocella arvoryzae Halobacterium salinarum NRC-1 Halobacterium salinarum Halalkalicoccus jeotgali Haloarcula marismortui Haloarcula hispanica ATCC 33960 Haloarcula hispanica N601 Haloarcula sp. CBA1115 Haloarcula taiwanensis Halomicrobium mukohataei JP60 Haloarculaceae archaeon HArcel1 Haloferax volcanii Haloferax mediterranei Haloferax gibbonsii Haloferax alexandrinus Halogeometricum borinquense Haloplanus sp. CBA1112 Haloplanus sp. CBA1113 Haloplanus aerogenes Haloplanus rallus Halobellus limi Haloprofundus sp. MHR1 Halorubrum sp. PV6 Halorubrum sp. BOL3-1 Halorubrum ezzemoulense Salinigranum rubrum Halohasta litchfieldiae Lokiarchaeum sp. GC14\_75 | 184% 150% 122% 100% 82% 67% 55% |
