# Supplementary material for: Isolation, identification, and whole-genome sequencing of high-yield protease bacteria from Daqu of ZhangGong Laojiu
Source: PLoS One. 2022 Apr 26;17(4):e0264677. doi: 10.1371/journal.pone.0264677 (PMC9041807; doi:10.1371/journal.pone.0264677)
Supplement: S1 Raw data — (ZIP) [file pone.0264677.s002.zip › Raw data/GDD20120317-1_Bacillus_velezensis_Genome_result/4_Basic_Annot/KEGG/Bac_map/map00520.html]

KEGG PATHWAY: Amino sugar and nucleotide sugar metabolism - Reference pathway


|  |  |
| --- | --- |
| **Amino sugar and nucleotide sugar metabolism - Reference pathway** |  |

[
Pathway menu
| Organism menu
| Pathway entry
| User data mapping
]

|  |  |
| --- | --- |
| Reference pathway Reference pathway (KO) Reference pathway (EC) Reference pathway (Reaction) -----< Sort below by alphabet >----- Homo sapiens (human) Homo sapiens (human) + Disease/drug Pan troglodytes (chimpanzee) Pan paniscus (bonobo) Gorilla gorilla gorilla (western lowland gorilla) Pongo abelii (Sumatran orangutan) Nomascus leucogenys (northern white-cheeked gibbon) Macaca mulatta (rhesus monkey) Macaca fascicularis (crab-eating macaque) Chlorocebus sabaeus (green monkey) Rhinopithecus roxellana (golden snub-nosed monkey) Rhinopithecus bieti (black snub-nosed monkey) Callithrix jacchus (white-tufted-ear marmoset) Saimiri boliviensis boliviensis (Bolivian squirrel monkey) Mus musculus (mouse) Mus caroli (Ryukyu mouse) Mus pahari (shrew mouse) Rattus norvegicus (rat) Meriones unguiculatus (Mongolian gerbil) Cricetulus griseus (Chinese hamster) Nannospalax galili (Upper Galilee mountains blind mole rat) Heterocephalus glaber (naked mole rat) Castor canadensis (American beaver) Oryctolagus cuniculus (rabbit) Tupaia chinensis (Chinese tree shrew) Canis familiaris (dog) Vulpes vulpes (red fox) Ailuropoda melanoleuca (giant panda) Ursus maritimus (polar bear) Ursus arctos horribilis Odobenus rosmarus divergens (Pacific walrus) Enhydra lutris kenyoni (northern sea otter) Felis catus (domestic cat) Panthera tigris altaica (Amur tiger) Panthera pardus (leopard) Acinonyx jubatus (cheetah) Bos taurus (cow) Bos mutus (wild yak) Bos indicus (zebu cattle) Bubalus bubalis (water buffalo) Capra hircus (goat) Ovis aries (sheep) Sus scrofa (pig) Camelus ferus (Wild Bactrian camel) Camelus dromedarius (Arabian camel) Balaenoptera acutorostrata scammoni (minke whale) Lipotes vexillifer (Yangtze River dolphin) Orcinus orca (killer whale) Delphinapterus leucas (beluga whale) Physeter catodon (sperm whale) Equus caballus (horse) Equus przewalskii (Przewalski's horse) Equus asinus (ass) Myotis brandtii (Brandt's bat) Myotis davidii Miniopterus natalensis Hipposideros armiger (great roundleaf bat) Desmodus rotundus (common vampire bat) Pteropus alecto (black flying fox) Rousettus aegyptiacus (Egyptian rousette) Manis javanica (Malayan pangolin) Loxodonta africana (African savanna elephant) Trichechus manatus latirostris (Florida manatee) Monodelphis domestica (opossum) Sarcophilus harrisii (Tasmanian devil) Phascolarctos cinereus (koala) Ornithorhynchus anatinus (platypus) Gallus gallus (chicken) Meleagris gallopavo (turkey) Coturnix japonica (Japanese quail) Numida meleagris (helmeted guineafowl) Anas platyrhynchos (mallard) Anser cygnoides domesticus (swan goose) Taeniopygia guttata (zebra finch) Lonchura striata domestica (Bengalese finch) Serinus canaria (common canary) Geospiza fortis (medium ground-finch) Ficedula albicollis (collared flycatcher) Pseudopodoces humilis (Tibetan ground-tit) Parus major (Great Tit) Cyanistes caeruleus (blue tit) Corvus cornix (hooded crow) Empidonax traillii (willow flycatcher) Falco peregrinus (peregrine falcon) Falco cherrug (Saker falcon) Columba livia (rock pigeon) Egretta garzetta (little egret) Nipponia nippon (crested ibis) Athene cunicularia (burrowing owl) Pygoscelis adeliae (Adelie penguin) Apteryx mantelli mantelli (brown kiwi) Alligator sinensis (Chinese alligator) Alligator mississippiensis (American alligator) Pelodiscus sinensis (Chinese soft-shelled turtle) Chelonia mydas (green sea turtle) Chrysemys picta (western painted turtle) Anolis carolinensis (green anole) Pogona vitticeps (central bearded dragon) Python bivittatus (Burmese python) Protobothrops mucrosquamatus (Taiwan habu) Thamnophis sirtalis Podarcis muralis (common wall lizard) Gekko japonicus Xenopus laevis (African clawed frog) Xenopus tropicalis (western clawed frog) Nanorana parkeri Danio rerio (zebrafish) Sinocyclocheilus rhinocerous Sinocyclocheilus anshuiensis Sinocyclocheilus grahami Cyprinus carpio (common carp) Ictalurus punctatus (channel catfish) Pangasianodon hypophthalmus (striped catfish) Astyanax mexicanus (Mexican tetra) Electrophorus electricus (electric eel) Takifugu rubripes (torafugu) Tetraodon nigroviridis (spotted green pufferfish) Larimichthys crocea (large yellow croaker) Notothenia coriiceps (black rockcod) Maylandia zebra (zebra mbuna) Oreochromis niloticus (Nile tilapia) Oryzias latipes (Japanese medaka) Xiphophorus maculatus (southern platyfish) Xiphophorus couchianus (Monterrey platyfish) Poecilia reticulata (guppy) Cyprinodon variegatus (sheepshead minnow) Nothobranchius furzeri (turquoise killifish) Kryptolebias marmoratus (mangrove rivulus) Austrofundulus limnaeus (annual killifish) Amphiprion ocellaris (clown anemonefish) Cynoglossus semilaevis (tongue sole) Paralichthys olivaceus (Japanese flounder) Lates calcarifer (barramundi perch) Seriola dumerili (greater amberjack) Seriola lalandi dorsalis (Yellowtail amberjack) Hippocampus comes (tiger tail seahorse) Boleophthalmus pectinirostris (great blue-spotted mudskipper) Monopterus albus (swamp eel) Salmo salar (Atlantic salmon) Oncorhynchus tshawytscha (Chinook salmon) Salvelinus alpinus (Arctic char) Esox lucius (northern pike) Scleropages formosus (Asian bonytongue) Paramormyrops kingsleyae Latimeria chalumnae (coelacanth) Callorhinchus milii (elephant shark) Rhincodon typus (whale shark) Branchiostoma floridae (Florida lancelet) Ciona intestinalis (sea squirt) Strongylocentrotus purpuratus (purple sea urchin) Acanthaster planci (crown-of-thorns starfish) Saccoglossus kowalevskii (acorn worm) Drosophila melanogaster (fruit fly) Drosophila erecta Drosophila sechellia Drosophila simulans Drosophila yakuba Drosophila ananassae Drosophila serrata Drosophila pseudoobscura pseudoobscura Drosophila persimilis Drosophila miranda Drosophila willistoni Drosophila grimshawi Drosophila mojavensis Drosophila arizonae Drosophila navojoa Drosophila hydei Drosophila virilis Musca domestica (house fly) Lucilia cuprina (Australian sheep blowfly) Anopheles gambiae (mosquito) Aedes aegypti (yellow fever mosquito) Aedes albopictus (Asian tiger mosquito) Culex quinquefasciatus (southern house mosquito) Apis mellifera (honey bee) Bombus impatiens (common eastern bumble bee) Bombus terrestris (buff-tailed bumblebee) Ceratina calcarata (carpenter bee) Osmia bicornis bicornis Solenopsis invicta (red fire ant) Monomorium pharaonis (pharaoh ant) Acromyrmex echinatior (Panamanian leafcutter ant) Atta cephalotes (leaf cutting ant) Pogonomyrmex barbatus (red harvester ant) Vollenhovia emeryi Harpegnathos saltator (Jerdon's jumping ant) Dinoponera quadriceps Camponotus floridanus (Florida carpenter ant) Linepithema humile (Argentine ant) Pseudomyrmex gracilis Ooceraea biroi (clonal raider ant) Polistes canadensis Nasonia vitripennis (jewel wasp) Ceratosolen solmsi marchali Microplitis demolitor Tribolium castaneum (red flour beetle) Dendroctonus ponderosae (mountain pine beetle) Aethina tumida (small hive beetle) Nicrophorus vespilloides Bombyx mori (domestic silkworm) Bombyx mandarina (wild silkworm) Danaus plexippus (monarch butterfly) Papilio machaon (common yellow swallowtail) Pieris rapae (cabbage white) Helicoverpa armigera (cotton bollworm) Trichoplusia ni (cabbage looper) Plutella xylostella (diamondback moth) Acyrthosiphon pisum (pea aphid) Diuraphis noxia (Russian wheat aphid) Aphis gossypii (cotton aphid) Rhopalosiphum maidis (corn leaf aphid) Bemisia tabaci (sweet potato whitefly) Cimex lectularius (bed bug) Pediculus humanus corporis (human body louse) Zootermopsis nevadensis Folsomia candida Daphnia pulex (common water flea) Penaeus vannamei (Pacific white shrimp) Ixodes scapularis (black-legged tick) Tetranychus urticae (two-spotted spider mite) Dermatophagoides pteronyssinus (European house dust mite) Centruroides sculpturatus (bark scorpion) Parasteatoda tepidariorum (common house spider) Caenorhabditis elegans (nematode) Caenorhabditis briggsae Brugia malayi (filaria) Loa loa (eye worm) Necator americanus Trichinella spiralis Helobdella robusta Lottia gigantea (owl limpet) Pomacea canaliculata (golden apple snail) Crassostrea gigas (Pacific oyster) Mizuhopecten yessoensis (Yesso scallop) Octopus bimaculoides Lingula anatina Schistosoma mansoni Schistosoma haematobium Opisthorchis viverrini Echinococcus granulosus Nematostella vectensis (sea anemone) Exaiptasia pallida Acropora digitifera (stony coral) Acropora millepora Pocillopora damicornis (lace coral) Stylophora pistillata Dendronephthya gigantea (soft corals) Hydra vulgaris Trichoplax adhaerens Amphimedon queenslandica (sponge) Arabidopsis thaliana (thale cress) Arabidopsis lyrata (lyrate rockcress) Capsella rubella Camelina sativa (false flax) Eutrema salsugineum Brassica rapa (field mustard) Brassica napus (rape) Brassica oleracea (wild cabbage) Raphanus sativus (radish) Tarenaya hassleriana (spider flower) Carica papaya (papaya) Citrus sinensis (Valencia orange) Citrus clementina (mandarin orange) Theobroma cacao (cacao) Gossypium raimondii Gossypium hirsutum (upland cotton) Gossypium arboreum Durio zibethinus (durian) Eucalyptus grandis (rose gum) Glycine max (soybean) Glycine soja (wild soybean) Phaseolus vulgaris (common bean) Vigna radiata (mung bean) Vigna angularis (adzuki bean) Vigna unguiculata (cowpea) Cajanus cajan (pigeon pea) Medicago truncatula (barrel medic) Cicer arietinum (chickpea) Lotus japonicus Arachis duranensis Arachis ipaensis Lupinus angustifolius (narrow-leaved blue lupine) Fragaria vesca (woodland strawberry) Rosa chinensis (China rose) Prunus persica (peach) Prunus mume (Japanese apricot) Prunus avium (sweet cherry) Malus domestica (apple) Pyrus x bretschneideri (Chinese white pear) Ziziphus jujuba (Chinese jujube) Cucumis sativus (cucumber) Cucumis melo (muskmelon) Momordica charantia (bitter melon) Cucurbita maxima (winter squash) Cucurbita moschata (crookneck pumpkin) Cucurbita pepo subsp. pepo (vegetable marrow) Ricinus communis (castor bean) Jatropha curcas Hevea brasiliensis (rubber tree) Manihot esculenta (cassava) Populus trichocarpa (black cottonwood) Populus euphratica (Euphrates poplar) Juglans regia (English walnut) Quercus suber (cork oak) Vitis vinifera (wine grape) Solanum lycopersicum (tomato) Solanum pennellii Solanum tuberosum (potato) Capsicum annuum Nicotiana tabacum (common tobacco) Nicotiana sylvestris Nicotiana tomentosiformis Nicotiana attenuata Ipomoea nil (Japanese morning glory) Sesamum indicum (sesame) Olea europaea var. sylvestris (wild olive) Helianthus annuus (common sunflower) Lactuca sativa (garden lettuce) Cynara cardunculus var. scolymus (artichoke) Daucus carota (carrot) Beta vulgaris (sugar beet) Spinacia oleracea (spinach) Chenopodium quinoa (quinoa) Nelumbo nucifera (sacred lotus) Papaver somniferum (opium poppy) Oryza sativa japonica (Japanese rice) (RefSeq) Oryza sativa japonica (Japanese rice) (RAPDB) Oryza brachyantha (malo sina) Brachypodium distachyon Aegilops tauschii (wheat D) Sorghum bicolor (sorghum) Zea mays (maize) Setaria italica (foxtail millet) Phoenix dactylifera (date palm) Elaeis guineensis (African oil palm) Musa acuminata (wild Malaysian banana) Dendrobium catenatum Phalaenopsis equestris Asparagus officinalis (garden asparagus) Amborella trichopoda Selaginella moellendorffii Physcomitrella patens subsp. patens Chlamydomonas reinhardtii Volvox carteri f. nagariensis Monoraphidium neglectum Coccomyxa subellipsoidea Chlorella variabilis Auxenochlorella protothecoides Ostreococcus lucimarinus Ostreococcus tauri Bathycoccus prasinos Micromonas commoda Micromonas pusilla Cyanidioschyzon merolae Galdieria sulphuraria Chondrus crispus (carragheen) Saccharomyces cerevisiae (budding yeast) Ashbya gossypii (Eremothecium gossypii) Eremothecium cymbalariae Kluyveromyces lactis Kluyveromyces marxianus Lachancea thermotolerans Vanderwaltozyma polyspora Zygosaccharomyces rouxii Candida glabrata Naumovozyma castellii Naumovozyma dairenensis Tetrapisispora phaffii Tetrapisispora blattae Torulaspora delbrueckii Kazachstania africana Komagataella phaffii Debaryomyces hansenii Scheffersomyces stipitis Meyerozyma guilliermondii Spathaspora passalidarum Lodderomyces elongisporus Candida albicans Candida tropicalis Candida orthopsilosis Candida dubliniensis Yamadazyma tenuis Yarrowia lipolytica Clavispora lusitaniae ATCC 42720 Clavispora lusitaniae CBS 6936 Candida auris Sugiyamaella lignohabitans Pichia kudriavzevii Neurospora crassa Neurospora tetrasperma Sordaria macrospora Podospora anserina Thermothielavioides terrestris Thermothelomyces thermophilus Chaetomium thermophilum Pyricularia oryzae Phaeoacremonium minimum Sporothrix schenckii Fusarium graminearum Fusarium pseudograminearum Fusarium verticillioides Fusarium oxysporum Nectria haematococca Trichoderma reesei QM6a Trichoderma reesei RUT C-30 Metarhizium acridum Metarhizium robertsii Cordyceps militaris Purpureocillium lilacinum Verticillium alfalfae Verticillium dahliae Colletotrichum fioriniae Scedosporium apiospermum Eutypa lata Pestalotiopsis fici Sclerotinia sclerotiorum Botrytis cinerea Marssonina brunnea Phialocephala scopiformis Glarea lozoyensis Aspergillus nidulans Aspergillus fumigatus Aspergillus clavatus Aspergillus fischeri Aspergillus oryzae Aspergillus niger Aspergillus flavus Penicillium rubens Penicillium digitatum Talaromyces marneffei Coccidioides immitis Coccidioides posadasii Paracoccidioides lutzii Pb01 Paracoccidioides brasiliensis Uncinocarpus reesii Trichophyton benhamiae Trichophyton verrucosum Histoplasma capsulatum Parastagonospora nodorum Pyrenophora teres Bipolaris zeicola Bipolaris sorokiniana Bipolaris oryzae Alternaria alternata Zymoseptoria tritici Pseudocercospora fijiensis Baudoinia panamericana Neofusicoccum parvum Tuber melanosporum Schizosaccharomyces pombe (fission yeast) Cryptococcus neoformans var. neoformans JEC21 Cryptococcus neoformans var. neoformans B-3501A Cryptococcus gattii Tremella mesenterica Postia placenta Trametes versicolor Dichomitus squalens Phanerochaete carnosa Stereum hirsutum Heterobasidion irregulare Punctularia strigosozonata Auricularia subglabra Fomitiporia mediterranea Gloeophyllum trabeum Laccaria bicolor Moniliophthora perniciosa Moniliophthora roreri Coprinopsis cinerea Schizophyllum commune Agaricus bisporus var. burnettii JB137-S8 Agaricus bisporus var. bisporus H97 Coniophora puteana Serpula lacrymans Wallemia mellicola Wallemia ichthyophaga Ustilago maydis Anthracocystis flocculosa Malassezia globosa Malassezia restricta Puccinia graminis Melampsora larici-populina Encephalitozoon cuniculi Encephalitozoon intestinalis Encephalitozoon hellem Encephalitozoon romaleae Nosema ceranae Monosiga brevicollis Salpingoeca rosetta Dictyostelium discoideum (cellular slime mold) Dictyostelium purpureum (cellular slime mold) Cavenderia fasciculata (cellular slime mold) Entamoeba histolytica Entamoeba dispar Entamoeba invadens Acanthamoeba castellanii Plasmodium falciparum 3D7 Plasmodium falciparum Dd2 Plasmodium falciparum HB3 Plasmodium yoelii Plasmodium chabaudi Plasmodium berghei Plasmodium knowlesi Plasmodium vivax Plasmodium cynomolgi Theileria annulata Theileria parva Theileria orientalis Theileria equi Babesia bovis Babesia microti Cryptosporidium parvum Cryptosporidium hominis Toxoplasma gondii Tetrahymena thermophila Paramecium tetraurelia Breviolum minutum Phaeodactylum tricornutum Fragilariopsis cylindrus Thalassiosira pseudonana Nannochloropsis gaditana Aureococcus anophagefferens Phytophthora infestans Phytophthora sojae Saprolegnia parasitica Emiliania huxleyi Guillardia theta Trypanosoma brucei brucei Trypanosoma cruzi Leishmania major Leishmania infantum Leishmania donovani Leishmania mexicana Leishmania braziliensis Naegleria gruberi Trichomonas vaginalis Giardia lamblia Escherichia coli K-12 MG1655 Escherichia coli K-12 W3110 Escherichia coli K-12 DH10B Escherichia coli K-12 BW2952 Escherichia coli K-12 MDS42 Escherichia coli O157:H7 EDL933 (EHEC) Escherichia coli O157:H7 Sakai (EHEC) Escherichia coli O157:H7 EC4115 (EHEC) Escherichia coli O157:H7 TW14359 (EHEC) Escherichia coli O157:H7 Xuzhou21 (EHEC) Escherichia coli O111:H- 11128 (EHEC) Escherichia coli O26:H11 11368 (EHEC) Escherichia coli O103:H2 12009 (EHEC) Escherichia coli O145:H28 RM13514 (EHEC) Escherichia coli O145:H28 RM13516 (EHEC) Escherichia coli O104:H4 2011C-3493 (EAEC) Escherichia coli O104:H4 2009EL-2071 (EAEC) Escherichia coli O104:H4 2009EL-2050 (EAEC) Escherichia coli 55989 (EAEC) Escherichia coli O127:H6 E2348/69 (EPEC) Escherichia coli O55:H7 CB9615 (EPEC) Escherichia coli O55:H7 RM12579 (EPEC) Escherichia coli O78:H11:K80 H10407 (ETEC) Escherichia coli O139:H28 E24377A (ETEC) Escherichia coli UMNK88 (ETEC, porcine) Escherichia coli O6:K15:H31 536 (UPEC) Escherichia coli NA114 (UPEC) Escherichia coli O25b:K100:H4-ST131 EC958 (UPEC) Escherichia coli APEC O1 (APEC) Escherichia coli APEC O78 (APEC) Escherichia coli O9 HS (commensal) Escherichia coli SMS-3-5 (environmental) Escherichia coli O152:H28 SE11 (commensal) Escherichia coli O8 IAI1 (commensal) Escherichia coli O81 ED1a (commensal) Escherichia coli O17:K52:H18 UMN026 (ExPEC) Escherichia coli O7:K1 IAI39 (ExPEC) Escherichia coli O7:K1 CE10 Escherichia coli B REL606 Escherichia coli BL21(DE3) Escherichia coli BL21(DE3) Escherichia coli BL21-Gold(DE3)pLysS AG Escherichia coli O18:K1:H7 UTI89 (UPEC) Escherichia coli O18:K1:H7 IHE3034 (ExPEC) Escherichia coli O45:K1:H7 S88 (ExPEC) Escherichia coli O6:K2:H1 CFT073 (UPEC) Escherichia coli O44:H18 042 (EAEC) Escherichia coli O83:H1 NRG 857C (AIEC) Escherichia coli O150:H5 SE15 (commensal) Escherichia coli ATCC 8739 Escherichia coli KO11FL Escherichia coli KO11FL Escherichia coli ABU 83972 Escherichia coli DH1 Escherichia coli DH1 Escherichia coli UM146 Escherichia coli W Escherichia coli W Escherichia coli clone D i14 Escherichia coli clone D i2 Escherichia coli P12b Escherichia coli LF82 Escherichia coli LY180 Escherichia coli PMV-1 Escherichia coli JJ1886 Escherichia fergusonii Escherichia albertii Escherichia marmotae Escherichia sp. E4742 Salmonella enterica subsp. enterica serovar Typhi CT18 Salmonella enterica subsp. enterica serovar Typhi Ty2 Salmonella enterica subsp. enterica serovar Typhi P-stx-12 Salmonella enterica subsp. enterica serovar Typhi Ty21a Salmonella enterica subsp. enterica serovar Typhimurium LT2 Salmonella enterica subsp. enterica serovar Typhimurium 14028S Salmonella enterica subsp. enterica serovar Typhimurium D23580 Salmonella enterica subsp. enterica serovar Typhimurium SL1344 Salmonella enterica subsp. enterica serovar Typhimurium T000240 Salmonella enterica subsp. enterica serovar Typhimurium UK-1 Salmonella enterica subsp. enterica serovar Typhimurium ST4/74 Salmonella enterica subsp. enterica serovar Typhimurium 798 Salmonella enterica subsp. enterica serovar Typhimurium U288 Salmonella enterica subsp. enterica serovar Typhimurium var. 5- CFSAN001921 Salmonella enterica subsp. enterica serovar Typhimurium DT2 Salmonella enterica subsp. enterica serovar Typhimurium DT104 Salmonella enterica subsp. enterica serovar Typhimurium 138736 Salmonella enterica subsp. enterica serovar 4,[5],12:i:- str. 08-1736 Salmonella enterica subsp. enterica serovar Paratyphi A ATCC9150 Salmonella enterica subsp. enterica serovar Paratyphi A AKU12601 Salmonella enterica subsp. enterica serovar Paratyphi B Salmonella enterica subsp. enterica serovar Paratyphi C Salmonella enterica subsp. enterica serovar Choleraesuis Salmonella enterica subsp. enterica serovar Heidelberg SL476 Salmonella enterica subsp. enterica serovar Heidelberg B182 Salmonella enterica subsp. enterica serovar Heidelberg CFSAN002069 Salmonella enterica subsp. enterica serovar Heidelberg 41578 Salmonella enterica subsp. enterica serovar Newport SL254 Salmonella enterica subsp. enterica serovar Newport USMARC-S3124.1 Salmonella enterica subsp. enterica serovar Schwarzengrund Salmonella enterica subsp. enterica serovar Agona SL483 Salmonella enterica subsp. enterica serovar Agona 24249 Salmonella enterica subsp. enterica serovar Dublin Salmonella enterica subsp. enterica serovar Gallinarum 287/91 Salmonella enterica subsp. enterica serovar Gallinarum/pullorum RKS5078 Salmonella enterica subsp. enterica serovar Gallinarum/pullorum CDC1983-67 Salmonella enterica subsp. enterica serovar Enteritidis P125109 Salmonella enterica subsp. enterica serovar Enteritidis EC20090135 Salmonella enterica subsp. enterica serovar Enteritidis EC20090193 Salmonella enterica subsp. enterica serovar Enteritidis EC20090332 Salmonella enterica subsp. enterica serovar Enteritidis EC20090531 Salmonella enterica subsp. enterica serovar Enteritidis OLF-SE1-1019-1 Salmonella enterica subsp. enterica serovar Javiana Salmonella enterica subsp. enterica serovar Cubana Salmonella enterica subsp. enterica serovar Bareilly Salmonella enterica subsp. enterica serovar Pullorum Salmonella enterica subsp. enterica serovar Bovismorbificans Salmonella enterica subsp. enterica serovar Thompson Salmonella enterica subsp. enterica serovar Tennessee Salmonella enterica subsp. arizonae Salmonella bongori NCTC 12419 Salmonella bongori N268-08 Salmonella bongori serovar 48:z41:-- Salmonella sp. SSDFZ69 Shigella flexneri 301 (serotype 2a) Shigella flexneri 2457T (serotype 2a) Shigella flexneri 8401 (serotype 5b) Shigella flexneri 2002017 (serotype Fxv) Shigella flexneri 2003036 Shigella flexneri Shi06HN006 (serotype Yv) Shigella flexneri NCTC1 (serotype 2a) Shigella sonnei Ss046 Shigella boydii Sb227 (serotype 4) Shigella boydii CDC 3083-94 (serotype 18) Shigella dysenteriae Sd197 Shigella dysenteriae 1617 (serotype 1) Shigella sp. PAMC 28760 Enterobacter cloacae subsp. cloacae ATCC 13047 Enterobacter cloacae subsp. cloacae ENHKU01 Enterobacter cloacae subsp. cloacae NCTC 9394 Enterobacter cloacae subsp. dissolvens SDM Enterobacter cloacae GGT036 Enterobacter cloacae ECNIH2 Enterobacter cloacae ECNIH4 Enterobacter cloacae ECNIH5 Enterobacter hormaechei subsp. xiangfangensis Enterobacter hormaechei subsp. steigerwaltii Enterobacter hormaechei subsp. hormaechei Enterobacter hormaechei CAV1176 Enterobacter hormaechei subsp. xiangfangensis Enterobacter hormaechei subsp. hoffmannii ECNIH3 Enterobacter hormaechei subsp. hoffmannii ECR091 Enterobacter asburiae L1 Enterobacter kobei Enterobacter ludwigii EcWSU1 Enterobacter ludwigii EN-119 Enterobacter cancerogenus Enterobacter roggenkampii Enterobacter cloacae complex sp. 35734 Enterobacter chengduensis Enterobacter sp. 638 Enterobacter soli Enterobacter sp. R4-368 Enterobacter sp. E20 Enterobacter sp. FY-07 Enterobacter bugandensis Enterobacter sp. ODB01 Cronobacter sakazakii ATCC BAA-894 Cronobacter sakazakii ES15 Cronobacter sakazakii Sp291 Cronobacter sakazakii ATCC 29544 Cronobacter condimenti Cronobacter dublinensis Cronobacter malonaticus CMCC45402 Cronobacter malonaticus LMG 23826 Cronobacter universalis Cronobacter muytjensii Cronobacter turicensis Klebsiella pneumoniae subsp. pneumoniae MGH 78578 (serotype K52) Klebsiella pneumoniae subsp. pneumoniae NTUH-K2044 (serotype K1) Klebsiella pneumoniae subsp. pneumoniae HS11286 Klebsiella pneumoniae subsp. pneumoniae 1084 (serotype K1) Klebsiella pneumoniae subsp. pneumoniae KPNIH24 Klebsiella pneumoniae subsp. pneumoniae KPNIH27 Klebsiella pneumoniae subsp. pneumoniae KPNIH29 Klebsiella pneumoniae subsp. pneumoniae KPNIH30 Klebsiella pneumoniae subsp. pneumoniae KPNIH31 Klebsiella pneumoniae subsp. pneumoniae KPNIH32 Klebsiella pneumoniae subsp. pneumoniae KPNIH10 Klebsiella pneumoniae subsp. pneumoniae KPR0928 Klebsiella pneumoniae subsp. pneumoniae ATCC 43816 KPPR1 Klebsiella pneumoniae 342 Klebsiella pneumoniae KCTC 2242 Klebsiella pneumoniae subsp. rhinoscleromatis SB3432 Klebsiella pneumoniae JM45 Klebsiella pneumoniae CG43 Klebsiella pneumoniae 30660/NJST258\_1 Klebsiella pneumoniae 30684/NJST258\_2 Klebsiella pneumoniae PMK1 Klebsiella pneumoniae blaNDM-1 Klebsiella pneumoniae 32192 Klebsiella pneumoniae 34618 Klebsiella pneumoniae Kp52.145 Klebsiella variicola At-22 Klebsiella variicola KP5-1 Klebsiella variicola DX120E Klebsiella variicola DSM 15968 Klebsiella michiganensis KCTC 1686 Klebsiella michiganensis E718 Klebsiella michiganensis HKOPL1 Klebsiella michiganensis M1 Klebsiella michiganensis RC10 Klebsiella oxytoca KONIH1 Klebsiella oxytoca CAV1374 Klebsiella quasipneumoniae Klebsiella aerogenes KCTC 2190 Klebsiella aerogenes EA1509E Klebsiella quasivariicola Klebsiella sp. LTGPAF-6F Klebsiella huaxiensis Citrobacter rodentium Citrobacter koseri Citrobacter freundii Citrobacter braakii Citrobacter werkmanii Citrobacter youngae Citrobacter portucalensis Citrobacter freundii complex sp. CFNIH3 Citrobacter amalonaticus Y19 Citrobacter amalonaticus FDAARGOS\_165 Citrobacter sp. FDAARGOS\_156 Citrobacter farmeri Citrobacter sp. CFNIH10 Citrobacter sp. CRE-46 Citrobacter pasteurii Gibbsiella quercinecans Candidatus Blochmannia floridanus (Camponotus floridanus) Candidatus Blochmannia pennsylvanicus (Camponotus pennsylvanicus) Candidatus Blochmannia vafer (Camponotus vafer) Candidatus Blochmannia chromaiodes (Camponotus chromaiodes) Blochmannia endosymbiont of Camponotus (Colobopsis) obliquus 757 Blochmannia endosymbiont of Polyrhachis (Hedomyrma) turneri 675 Candidatus Hamiltonella defensa (Acyrthosiphon pisum) Secondary endosymbiont of Ctenarytaina eucalypti Secondary endosymbiont of Heteropsylla cubana Candidatus Riesia pediculicola Candidatus Riesia sp. GBBU Candidatus Moranella endobia PCIT Candidatus Moranella endobia PCVAL Shimwellia blattae Raoultella ornithinolytica B6 Raoultella ornithinolytica S12 Raoultella planticola Raoultella sp. X13 Raoultella terrigena Raoultella electrica Cedecea neteri SSMD04 Cedecea neteri M006 Cedecea neteri ND14a Cedecea lapagei Pluralibacter gergoviae Enterobacter lignolyticus SCF1 Enterobacter lignolyticus G5 Kosakonia sacchari Kosakonia oryzae Kosakonia radicincitans Kosakonia cowanii Kosakonia sp. CCTCC M2018092 Kluyvera georgiana Kluyvera intermedia Candidatus Ishikawaella capsulata Leclercia adecarboxylata Leclercia sp. LSNIH1 Leclercia sp. LSNIH3 Leclercia sp. W17 Leclercia sp. 1106151 Leclercia sp. J807 Lelliottia amnigena Lelliottia jeotgali Lelliottia nimipressuralis Lelliottia sp. WB101 Limnobaculum parvum Buttiauxella sp. 3AFRM03 Candidatus Tachikawaea gelatinosa Candidatus Doolittlea endobia Candidatus Hoaglandella endobia Candidatus Gullanella endobia Candidatus Purcelliella pentastirinorum Metakosakonia sp. MRY16-398 Atlantibacter hermannii Izhakiella sp. KSNA2 Enterobacteriaceae bacterium FGI 57 Enterobacteriaceae bacterium ENNIH2 Enterobacteriaceae bacterium S05 Plautia stali symbiont Yersinia pestis CO92 (biovar Orientalis) Yersinia pestis KIM10+ (biovar Mediaevalis) Yersinia pestis Harbin 35 (biovar Medievalis) Yersinia pestis Antiqua (biovar Antiqua) Yersinia pestis Nepal516 (biovar Antiqua) Yersinia pestis 91001 (biovar Microtus) Yersinia pestis Pestoides F (biovar Antiqua) Yersinia pestis Angola Yersinia pestis Z176003 Yersinia pestis A1122 Yersinia pestis D106004 Yersinia pestis D182038 Yersinia pestis PBM19 Yersinia pestis Harbin35 Yersinia pestis Shasta Yersinia pestis El Dorado Yersinia pseudotuberculosis IP 32953 (serotype I) Yersinia pseudotuberculosis IP 32953 (serotype 1) Yersinia pseudotuberculosis IP 31758 (serotype O:1b) Yersinia pseudotuberculosis YPIII (serotype O:3) Yersinia pseudotuberculosis PB1/+ (serotype 1B) Yersinia pseudotuberculosis ATCC 6904 Yersinia pseudotuberculosis MD67 Yersinia pseudotuberculosis EP2/+ Yersinia pseudotuberculosis 1 Yersinia pseudotuberculosis PA3606 Yersinia enterocolitica subsp. enterocolitica 8081 (serotype 0:8) Yersinia enterocolitica subsp. palearctica 105.5R(r) (serotype:O:9) Yersinia enterocolitica subsp. palearctica Y11 (serotype:0:3) Yersinia enterocolitica LC20 Yersinia enterocolitica WA Yersinia enterocolitica 2516-87 Yersinia enterocolitica FORC\_002 Yersinia enterocolitica (type O:5) YE53/03 Yersinia similis Yersinia aldovae Yersinia frederiksenii Yersinia intermedia Yersinia kristensenii Yersinia rohdei Yersinia ruckeri YRB Yersinia ruckeri Big Creek 74 (serotype O2) Yersinia aleksiciae Yersinia massiliensis Yersinia hibernica Yersinia canariae Serratia marcescens SM39 Serratia marcescens subsp. marcescens Db11 Serratia marcescens WW4 Serratia proteamaculans Serratia plymuthica AS9 Serratia plymuthica 4Rx13 Serratia plymuthica S13 Serratia plymuthica PRI-2C Serratia sp. AS12 Serratia sp. AS13 Serratia symbiotica Serratia sp. FGI94 Serratia liquefaciens Serratia sp. FS14 Serratia sp. SCBI Serratia fonticola DSM 4576 Serratia fonticola GS2 Serratia rubidaea Serratia sp. ATCC 39006 Serratia sp. ATCC 39006 Serratia sp. MYb239 Serratia quinivorans Serratia ficaria Chania multitudinisentens Rahnella sp. Y9602 Rahnella aquatilis CIP 78.65 = ATCC 33071 Rahnella aquatilis HX2 Rahnella sp. ERMR1:05 Candidatus Fukatsuia symbiotica Pectobacterium atrosepticum SCRI1043 Pectobacterium atrosepticum JG10-08 Pectobacterium atrosepticum 21A Pectobacterium carotovorum subsp. carotovorum PC1 Pectobacterium carotovorum subsp. carotovorum PCC21 Pectobacterium odoriferum Pectobacterium parmentieri WPP163 Pectobacterium parmentieri RNS08.42.1A Pectobacterium parmentieri SCC3193 Pectobacterium wasabiae Pectobacterium polaris Pectobacterium brasiliense Sodalis glossinidius (Glossina spp.) Sodalis praecaptivus Candidatus Sodalis pierantonius Sodalis endosymbiont of Henestaris halophilus Dickeya dadantii 3937 Dickeya paradisiaca Dickeya chrysanthemi Dickeya zeae Ech586 Dickeya zeae EC1 Dickeya solani IPO 2222 Dickeya fangzhongdai ND14b Dickeya fangzhongdai DSM 101947 Dickeya dianthicola Dickeya aquatica Dickeya sp. NCPPB 569 Brenneria goodwinii Brenneria rubrifaciens Brenneria nigrifluens Lonsdalea britannica Erwinia amylovora CFBP1430 Erwinia amylovora ATCC 49946 Erwinia tasmaniensis Erwinia pyrifoliae Ep1/96 Erwinia pyrifoliae DSM 12163 Erwinia billingiae Erwinia sp. Ejp617 Erwinia gerundensis Erwinia persicina Candidatus Erwinia haradaeae Erwinia sp. J780 Buchnera aphidicola APS (Acyrthosiphon pisum) Buchnera aphidicola 5A (Acyrthosiphon pisum) Buchnera aphidicola Tuc7 (Acyrthosiphon pisum) Buchnera aphidicola JF98 (Acyrthosiphon pisum) Buchnera aphidicola JF99 (Acyrthosiphon pisum) Buchnera aphidicola LL01 (Acyrthosiphon pisum) Buchnera aphidicola TLW03 (Acyrthosiphon pisum) Buchnera aphidicola Ak (Acyrthosiphon kondoi) Buchnera aphidicola Ua (Uroleucon ambrosiae) Buchnera aphidicola F009 (Myzus persicae) Buchnera aphidicola G002 (Myzus persicae) Buchnera aphidicola USDA (Myzus persicae) Buchnera aphidicola W106 (Myzus persicae) Buchnera aphidicola Sg (Schizaphis graminum) Buchnera aphidicola Bp (Baizongia pistaciae) Buchnera aphidicola (Aphis glycines) Wigglesworthia glossinidia brevipalpis (Glossina brevipalpis) Wigglesworthia glossinidia morsitans (Glossina morsitans) Pantoea ananatis LMG 20103 Pantoea ananatis LMG 5342 Pantoea ananatis AJ13355 Pantoea ananatis PA13 Pantoea vagans C9-1 Pantoea vagans FDAARGOS\_160 Pantoea sp. At-9b Pantoea rwandensis Pantoea sp. PSNIH1 Pantoea sp. PSNIH2 Candidatus Pantoea carbekii Candidatus Pantoea carbekii US Pantoea agglomerans C410P1 Pantoea stewartii subsp. stewartii Pantoea alhagi Pantoea sp. SO10 Pantoea eucalypti Mixta gaviniae Mixta calida Mixta intestinalis Mixta theicola Tatumella citrea Tatumella ptyseos Photorhabdus laumondii subsp. laumondii TTO1 Photorhabdus laumondii subsp. laumondii DSPV002N Photorhabdus asymbiotica Photorhabdus thracensis Proteus mirabilis HI4320 Proteus mirabilis BB2000 Proteus mirabilis CYPV1 Proteus vulgaris Proteus hauseri Proteus sp. CD3 Proteus cibarius Proteus columbae Xenorhabdus bovienii SS-2004 Xenorhabdus bovienii CS03 Xenorhabdus nematophila ATCC 19061 Xenorhabdus nematophila AN6/1 Xenorhabdus doucetiae Xenorhabdus poinarii Xenorhabdus hominickii Providencia stuartii MRSN 2154 Providencia stuartii ATCC 33672 Providencia stuartii BE2467 Providencia rettgeri Providencia alcalifaciens Providencia heimbachae Providencia sp. WCHPHu000369 Providencia rustigianii Providencia vermicola Morganella morganii Candidatus Arsenophonus lipoptenae Arsenophonus nasoniae Edwardsiella ictaluri Edwardsiella tarda EIB202 Edwardsiella tarda FL6-60 Edwardsiella anguillarum Edwardsiella piscicida C07-087 Edwardsiella sp. EA181011 Edwardsiella sp. LADL05-105 Edwardsiella hoshinae Hafnia alvei Hafnia paralvei Obesumbacterium proteus Pragia fontium Pragia sp. CF-458 Leminorella richardii Phytobacter ursingii Plesiomonas shigelloides Haemophilus influenzae Rd KW20 (serotype d) Haemophilus influenzae 86-028NP (nontypeable) Haemophilus influenzae PittEE (nontypeable) Haemophilus influenzae PittGG (nontypeable) Haemophilus influenzae F3031 (nontypeable) Haemophilus influenzae F3047 (nontypeable) Haemophilus influenzae 10810 (serotype b) Haemophilus influenzae R2846 (nontypeable) Haemophilus influenzae R2866 (nontypeable) Haemophilus influenzae KR494 (serotype f) Haemophilus influenzae CGSHiCZ412602 Haemophilus influenzae Hi375 Haemophilus influenzae 477 Haemophilus influenzae C486 Haemophilus influenzae 723 Haemophilus parainfluenzae Haemophilus ducreyi Haemophilus sp. oral taxon 036 Haemophilus pittmaniae Haemophilus haemolyticus Glaesserella parasuis SH0165 Glaesserella parasuis ZJ0906 Glaesserella parasuis SH03 Glaesserella parasuis KL0318 Glaesserella sp. 15-184 Histophilus somni 129PT Histophilus somni 2336 Pasteurella multocida subsp. multocida Pm70 Pasteurella multocida subsp. multocida HN06 Pasteurella multocida subsp. multocida 3480 Pasteurella multocida 36950 Pasteurella multocida ATCC 43137 Pasteurella dagmatis Pasteurella aerogenes Mannheimia succiniciproducens Mannheimia haemolytica USDA-ARS-USMARC-185 Mannheimia haemolytica USDA-ARS-USMARC-183 Mannheimia haemolytica USDA-ARS-USMARC-184 Mannheimia haemolytica M42548 Mannheimia haemolytica D153 Mannheimia haemolytica D171 Mannheimia haemolytica D174 Mannheimia haemolytica USMARC\_2286 Mannheimia haemolytica 89010807N Mannheimia haemolytica 89010807N lktA- Mannheimia sp. USDA-ARS-USMARC-1261 Mannheimia varigena USDA-ARS-USMARC-1296 Mannheimia varigena USDA-ARS-USMARC-1312 Mannheimia varigena USDA-ARS-USMARC-1388 Mannheimia sp. ZY170218 Actinobacillus pleuropneumoniae L20 (serotype 5b) Actinobacillus pleuropneumoniae JL03 (serotype 3) Actinobacillus pleuropneumoniae AP76 (serotype 7) Actinobacillus succinogenes Actinobacillus suis H91-0380 Actinobacillus suis ATCC 33415 Actinobacillus equuli Actinobacillus porcitonsillarum Actinobacillus indolicus Aggregatibacter aphrophilus NJ8700 Aggregatibacter aphrophilus W10433 Aggregatibacter actinomycetemcomitans D11S-1 Aggregatibacter actinomycetemcomitans ANH9381 Aggregatibacter actinomycetemcomitans D7S-1 Aggregatibacter actinomycetemcomitans HK1651 Aggregatibacter actinomycetemcomitans NUM4039 Aggregatibacter actinomycetemcomitans 624 Aggregatibacter segnis Gallibacterium anatis Bibersteinia trehalosi USDA-ARS-USMARC-192 Bibersteinia trehalosi USDA-ARS-USMARC-188 Bibersteinia trehalosi USDA-ARS-USMARC-189 Bibersteinia trehalosi USDA-ARS-USMARC-190 Avibacterium paragallinarum Rodentibacter pneumotropicus Rodentibacter heylii Otariodibacter oris Xylella fastidiosa 9a5c Xylella fastidiosa Temecula1 Xylella fastidiosa M12 Xylella fastidiosa M23 Xylella fastidiosa subsp. fastidiosa GB514 Xylella fastidiosa MUL0034 Xylella fastidiosa subsp. sandyi Ann-1 Xylella fastidiosa Hib4 Xylella taiwanensis Xanthomonas campestris pv. campestris ATCC 33913 Xanthomonas campestris pv. campestris 8004 Xanthomonas campestris pv. campestris B100 Xanthomonas campestris pv. raphani Xanthomonas campestris pv. vesicatoria Xanthomonas axonopodis pv. citrumelo F1 Xanthomonas citri pv. citri 306 Xanthomonas citri subsp. citri Aw12879 Xanthomonas citri subsp. citri A306 Xanthomonas citri subsp. citri UI6 Xanthomonas citri pv. citri UI7 Xanthomonas citri pv. citri NT17 Xanthomonas citri pv. citri MN10 Xanthomonas citri pv. citri MN11 Xanthomonas citri pv. citri MN12 Xanthomonas citri pv. citri mf20 Xanthomonas citri pv. fuscans Xanthomonas axonopodis Xac29-1 Xanthomonas oryzae pv. oryzae MAFF 311018 Xanthomonas oryzae pv. oryzae KACC 10331 Xanthomonas oryzae pv. oryzae PXO99A Xanthomonas oryzae pv. oryzae PXO86 Xanthomonas oryzae pv. oryzicola BLS256 Xanthomonas oryzae pv. oryzicola CFBP7342 Xanthomonas albilineans Xanthomonas sacchari Xanthomonas translucens Xanthomonas fragariae Xanthomonas gardneri Xanthomonas vesicatoria Xanthomonas perforans Xanthomonas hortorum Xanthomonas phaseoli Xanthomonas vasicola Xanthomonas sp. ISO98C4 Xanthomonas arboricola Xanthomonas hyacinthi Stenotrophomonas maltophilia K279a Stenotrophomonas maltophilia R551-3 Stenotrophomonas maltophilia JV3 Stenotrophomonas maltophilia D457 Stenotrophomonas acidaminiphila Stenotrophomonas sp. KCTC 12332 Stenotrophomonas rhizophila Stenotrophomonas sp. LM091 Stenotrophomonas sp. WZN-1 Stenotrophomonas sp. MYb57 Stenotrophomonas sp. ASS1 Pseudoxanthomonas suwonensis 11-1 Pseudoxanthomonas suwonensis J1 Pseudoxanthomonas spadix Lysobacter antibioticus 76 Lysobacter antibioticus ATCC 29479 Lysobacter capsici Lysobacter gummosus Lysobacter enzymogenes C3 Lysobacter enzymogenes M497-1 Lysobacter maris Lysobacter sp. TY2-98 Lysobacter oculi Lysobacter sp. SJ-36 Lysobacter soli Luteimonas sp. 100111 Luteimonas sp. S-1072 Luteimonas sp. Gr-4 Thermomonas sp. SY21 Pseudolysobacter antarcticus Frateuria aurantia Rhodanobacter denitrificans Rhodanobacter glycinis Dyella jiangningensis Dyella japonica Dyella thiooxydans Dyella sp. M7H15-1 Dokdonella koreensis Luteibacter rhizovicinus DSM 16549 Luteibacter pinisoli Ahniella affigens Aerosticca soli Vibrio cholerae O1 El Tor N16961 Vibrio cholerae O1 El Tor FJ147 Vibrio cholerae O1 El Tor MS6 Vibrio cholerae O1 2010EL-1786 Vibrio cholerae O1 2012EL-2176 Vibrio cholerae O1 MJ-1236 Vibrio cholerae O1 IEC224 Vibrio cholerae O1 O395 Vibrio cholerae O1 O395 Vibrio cholerae O1 M66-2 Vibrio cholerae O1 LMA3984-4 Vibrio cholerae O49 1154-74 Vibrio cholerae O27 10432-62 Vibrio vulnificus CMCP6 Vibrio vulnificus YJ016 Vibrio vulnificus MO6-24/O Vibrio vulnificus 93U204 Vibrio parahaemolyticus RIMD 2210633 Vibrio parahaemolyticus BB22OP Vibrio parahaemolyticus O1:K33 CDC\_K4557 Vibrio parahaemolyticus O1:Kuk FDA\_R31 Vibrio parahaemolyticus UCM-V493 Vibrio campbellii Vibrio campbellii Vibrio alginolyticus Vibrio antiquarius Vibrio diabolicus Vibrio harveyi Vibrio natriegens Vibrio owensii Vibrio rotiferianus Vibrio tasmaniensis Vibrio sp. EJY3 Vibrio furnissii Vibrio nigripulchritudo Vibrio anguillarum 775 Vibrio anguillarum M3 Vibrio anguillarum NB10 Vibrio coralliilyticus RE98 Vibrio coralliilyticus OCN014 Vibrio tubiashii Vibrio fluvialis Vibrio mimicus Vibrio breoganii Vibrio scophthalmi Vibrio gazogenes Vibrio mediterranei Vibrio qinghaiensis Vibrio tapetis Vibrio alfacsensis Vibrio neocaledonicus Vibrio cyclitrophicus Aliivibrio fischeri ES114 Aliivibrio fischeri MJ11 Aliivibrio salmonicida Aliivibrio wodanis Photobacterium profundum Photobacterium gaetbulicola Photobacterium damselae subsp. damselae Grimontia hollisae Paraphotobacterium marinum Salinivibrio sp. YCSC6 Salinivibrio kushneri Pseudomonas aeruginosa PAO1 Pseudomonas aeruginosa PAO1-VE13 Pseudomonas aeruginosa PAO1-VE2 Pseudomonas aeruginosa UCBPP-PA14 Pseudomonas aeruginosa PA7 Pseudomonas aeruginosa LESB58 Pseudomonas aeruginosa M18 Pseudomonas aeruginosa NCGM2.S1 Pseudomonas aeruginosa NCGM 1900 Pseudomonas aeruginosa DK2 Pseudomonas aeruginosa B136-33 Pseudomonas aeruginosa RP73 Pseudomonas aeruginosa PA1 Pseudomonas aeruginosa PA1R Pseudomonas aeruginosa MTB-1 Pseudomonas aeruginosa LES431 Pseudomonas aeruginosa SCV20265 Pseudomonas aeruginosa PA38182 Pseudomonas aeruginosa YL84 Pseudomonas aeruginosa c7447m Pseudomonas aeruginosa PAO581 Pseudomonas mendocina ymp Pseudomonas mendocina NK-01 Pseudomonas resinovorans Pseudomonas pseudoalcaligenes Pseudomonas alcaligenes Pseudomonas citronellolis Pseudomonas putida KT2440 Pseudomonas putida F1 Pseudomonas putida GB-1 Pseudomonas putida W619 Pseudomonas putida S16 Pseudomonas putida BIRD-1 Pseudomonas putida ND6 Pseudomonas putida DOT-T1E Pseudomonas putida HB3267 Pseudomonas putida H8234 Pseudomonas putida NBRC 14164 Pseudomonas putida DLL-E4 Pseudomonas fulva Pseudomonas monteilii SB3078 Pseudomonas monteilii SB3101 Pseudomonas soli Pseudomonas plecoglossicida Pseudomonas oryzihabitans Pseudomonas syringae pv. tomato DC3000 Pseudomonas syringae pv. syringae B728a Pseudomonas syringae CC1557 Pseudomonas savastanoi pv. phaseolicola 1448A Pseudomonas amygdali Pseudomonas cichorii Pseudomonas avellanae Pseudomonas protegens Pf-5 Pseudomonas protegens CHA0 Pseudomonas protegens Cab57 Pseudomonas fluorescens Pf0-1 Pseudomonas fluorescens SBW25 Pseudomonas fluorescens F113 Pseudomonas fluorescens A506 Pseudomonas fluorescens UK4 Pseudomonas poae Pseudomonas synxantha LBUM223 Pseudomonas mandelii Pseudomonas trivialis Pseudomonas corrugata Pseudomonas veronii Pseudomonas azotoformans Pseudomonas orientalis Pseudomonas simiae PCL1751 Pseudomonas simiae PICF7 Pseudomonas lurida Pseudomonas entomophila Pseudomonas stutzeri A1501 Pseudomonas stutzeri ATCC 17588 Pseudomonas stutzeri DSM 4166 Pseudomonas stutzeri CCUG 29243 Pseudomonas stutzeri DSM 10701 Pseudomonas stutzeri RCH2 Pseudomonas stutzeri 19SMN4 Pseudomonas stutzeri 28a24 Pseudomonas balearica Pseudomonas brassicacearum subsp. brassicacearum NFM421 Pseudomonas brassicacearum DF41 Pseudomonas sp. UW4 Pseudomonas sp. ATCC 13867 Pseudomonas sp. VLB120 Pseudomonas sp. TKP Pseudomonas knackmussii Pseudomonas chlororaphis PA23 Pseudomonas chlororaphis PCL1606 Pseudomonas chlororaphis subsp. aurantiaca Pseudomonas fragi Pseudomonas lundensis Pseudomonas alkylphenolica Pseudomonas rhizosphaerae Pseudomonas cremoricolorata Pseudomonas parafulva Pseudomonas sp. StFLB209 Pseudomonas sp. MRSN12121 Pseudomonas sp. CCOS 191 Pseudomonas versuta Pseudomonas sp. Os17 Pseudomonas koreensis Pseudomonas frederiksbergensis Pseudomonas antarctica Pseudomonas psychrotolerans Pseudomonas sp. TCU-HL1 Pseudomonas silesiensis Pseudomonas yamanorum Candidatus Pseudomonas adelgestsugas (Adelges tsugae) Pseudomonas sp. R2A2 Pseudomonas kribbensis Azotobacter vinelandii DJ Azotobacter vinelandii CA Azotobacter vinelandii CA6 Azotobacter chroococcum Oblitimonas alkaliphila Permianibacter aggregans Psychrobacter arcticus Psychrobacter cryohalolentis Psychrobacter sp. PRwf-1 Psychrobacter sp. G Psychrobacter urativorans Psychrobacter alimentarius Psychrobacter sp. P2G3 Psychrobacter sp. P11G5 Psychrobacter sp. DAB\_AL43B Psychrobacter sp. AntiMn-1 Psychrobacter sp. YP14 Psychrobacter sp. PAMC27889 Acinetobacter baumannii ATCC 17978 Acinetobacter baumannii SDF Acinetobacter baumannii AYE Acinetobacter baumannii ACICU Acinetobacter baumannii AB0057 Acinetobacter baumannii AB307-0294 Acinetobacter baumannii 1656-2 Acinetobacter baumannii MDR-ZJ06 Acinetobacter baumannii MDR-TJ Acinetobacter baumannii TCDC-AB0715 Acinetobacter baumannii TYTH-1 Acinetobacter baumannii D1279779 Acinetobacter baumannii BJAB07104 Acinetobacter baumannii BJAB0715 Acinetobacter baumannii BJAB0868 Acinetobacter baumannii ZW85-1 Acinetobacter baumannii AbH12O-A2 Acinetobacter baumannii AB030 Acinetobacter baumannii AB031 Acinetobacter baumannii AC29 Acinetobacter baumannii LAC-4 Acinetobacter pittii Acinetobacter nosocomialis Acinetobacter lactucae Acinetobacter calcoaceticus Acinetobacter oleivorans Acinetobacter baylyi Acinetobacter sp. TTH0-4 Acinetobacter equi Acinetobacter johnsonii Acinetobacter sp. DUT-2 Acinetobacter sp. TGL-Y2 Acinetobacter haemolyticus Acinetobacter junii Acinetobacter soli Acinetobacter larvae Acinetobacter schindleri Acinetobacter indicus Acinetobacter defluvii Acinetobacter radioresistens Acinetobacter wuhouensis Acinetobacter cumulans Acinetobacter guillouiae Acinetobacter ursingii Acinetobacter lwoffii Moraxella catarrhalis BBH18 Moraxella catarrhalis 25240 Moraxella catarrhalis 25239 Moraxella ovis Moraxella osloensis Moraxella bovoculi Moraxella bovis Moraxellaceae bacterium HYN0046 Shewanella oneidensis Shewanella denitrificans Shewanella frigidimarina Shewanella amazonensis Shewanella baltica OS155 Shewanella baltica OS185 Shewanella baltica OS195 Shewanella baltica OS223 Shewanella baltica OS678 Shewanella baltica OS117 Shewanella baltica BA175 Shewanella loihica Shewanella putrefaciens CN-32 Shewanella putrefaciens 200 Shewanella sediminis Shewanella pealeana Shewanella sp. MR-4 Shewanella sp. MR-7 Shewanella sp. ANA-3 Shewanella sp. W3-18-1 Shewanella halifaxensis Shewanella woodyi Shewanella piezotolerans Shewanella violacea Shewanella sp. FDAARGOS\_354 Shewanella japonica Shewanella psychrophila Shewanella bicestrii Shewanella marisflavi Shewanella sp. WE21 Shewanella algae Shewanella livingstonensis Shewanella maritima Idiomarina loihiensis L2TR Idiomarina loihiensis GSL 199 Idiomarina piscisalsi Idiomarina sp. X4 Idiomarina sp. OT37-5b Colwellia psychrerythraea Colwellia sp. MT41 Colwellia sp. PAMC 20917 Colwellia sp. PAMC 21821 Colwellia sp. Arc7-D Colwellia beringensis Colwellia sp. Arc7-635 Litorilituus sediminis Thalassotalea sp. HSM 43 Thalassotalea sp. PS06 Pseudoalteromonas haloplanktis Pseudoalteromonas atlantica Pseudoalteromonas sp. SM9913 Pseudoalteromonas piratica OCN003 Pseudoalteromonas issachenkonii Pseudoalteromonas phenolica Pseudoalteromonas sp. Bsw20308 Pseudoalteromonas rubra Pseudoalteromonas translucida Pseudoalteromonas luteoviolacea Pseudoalteromonas aliena Pseudoalteromonas piscicida Pseudoalteromonas espejiana Pseudoalteromonas spongiae Pseudoalteromonas arctica Pseudoalteromonas tunicata Pseudoalteromonas nigrifaciens Pseudoalteromonas tetraodonis Pseudoalteromonas sp. NC201 Pseudoalteromonas donghaensis Pseudoalteromonas agarivorans Marinobacter hydrocarbonoclasticus VT8 Marinobacter hydrocarbonoclasticus ATCC 49840 Marinobacter adhaerens Marinobacter sp. BSs20148 Marinobacter salarius Marinobacter similis Marinobacter psychrophilus Marinobacter sp. CP1 Marinobacter sp. LQ44 Marinobacter salinus Marinobacter sp. Arc7-DN-1 Marinobacter sp. JH2 Alteromonas mediterranea DE Alteromonas mediterranea 615 Alteromonas mediterranea DE1 Alteromonas mediterranea U4 Alteromonas mediterranea U7 Alteromonas mediterranea U8 Alteromonas mediterranea UM4b Alteromonas mediterranea UM7 Alteromonas mediterranea MED64 Alteromonas macleodii ATCC 27126 Alteromonas macleodii Balearic Sea AD45 Alteromonas macleodii English Channel 673 Alteromonas macleodii Black Sea 11 Alteromonas naphthalenivorans Alteromonas australica H 17 Alteromonas australica DE170 Alteromonas stellipolaris LMG 21856 Alteromonas stellipolaris LMG 21861 Alteromonas addita Alteromonas sp. RKMC-009 Alteromonas sp. Mac1 Alteromonas sp. Mac2 Glaciecola sp. 4H-3-7+YE-5 Glaciecola nitratireducens Paraglaciecola psychrophila Paraglaciecola mesophila Lacimicrobium alkaliphilum Catenovulum sp. CCB-QB4 Saliniradius amylolyticus Salinimonas sediminis Salinimonas sp. KX18D6 Hydrocarboniclastica marina Psychromonas ingrahamii Psychromonas sp. CNPT3 Ferrimonas balearica Moritella viscosa Moritella yayanosii Moritella marina Cellvibrio japonicus Cellvibrio sp. PSBB023 Cellvibrio sp. PSBB006 Cellvibrio sp. KY-YJ-3 Cellvibrio sp. KY-GH-1 Saccharophagus degradans Teredinibacter turnerae Simiduia agarivorans Spongiibacter sp. IMCC21906 Zhongshania aliphaticivorans Oceanicoccus sagamiensis Microbulbifer thermotolerans Microbulbifer aggregans Microbulbifer agarilyticus Microbulbifer sp. A4B17 Microbulbifer sp. THAF38 Microbulbifer hydrolyticus Halioglobus japonicus Halioglobus sp. IMCC14385 Kineobactrum sp. M2 Coxiella burnetii RSA 493 Coxiella burnetii RSA 331 Coxiella burnetii Dugway 5J108-111 Coxiella burnetii CbuG\_Q212 Coxiella burnetii CbuK\_Q154 Coxiella endosymbiont of Amblyomma americanum Coxiella mudrowiae Coxiella endosymbiont of Amblyomma sculptum Candidatus Rickettsiella viridis Aquicella lusitana Aquicella siphonis Legionella pneumophila subsp. pneumophila Philadelphia 1 (serogroup 1) Legionella pneumophila subsp. pneumophila HL06041035 (serogroup 1) Legionella pneumophila subsp. pneumophila Lorraine (serogroup 1) Legionella pneumophila subsp. pneumophila LPE509 Legionella pneumophila subsp. pneumophila Thunder Bay Legionella pneumophila Lens (serogroup 1) Legionella pneumophila Paris (serogroup 1) Legionella pneumophila Corby Legionella pneumophila 2300/99 Alcoy (serogroup 1) Legionella pneumophila subsp. pneumophila ATCC 43290 (serogroup 12) Legionella longbeachae Legionella fallonii Legionella hackeliae Legionella oakridgensis Legionella clemsonensis Legionella endosymbiont of Polyplax serrata Legionella sainthelensi Legionella lansingensis Legionella israelensis Legionella geestiana Legionella jordanis Tatlockia micdadei Methylococcus capsulatus Methylomonas methanica Methylomonas denitrificans Methylomonas sp. DH-1 Methylomonas koyamae Methylomonas sp. LW13 Methylomicrobium alcaliphilum Methylomicrobium buryatense Methylovulum psychrotolerans Methylocaldum marinum Candidatus Methylospira mobilis Francisella tularensis subsp. tularensis SCHU S4 Francisella tularensis subsp. tularensis SCHU S4 NR-28534 Francisella tularensis subsp. tularensis FSC198 Francisella tularensis subsp. tularensis WY96-3418 Francisella tularensis subsp. tularensis NE061598 Francisella tularensis subsp. tularensis TI0902 Francisella tularensis subsp. tularensis TIGB03 Francisella tularensis subsp. holarctica LVS Francisella tularensis subsp. holarctica OSU18 Francisella tularensis subsp. holarctica FTNF002-00 Francisella tularensis subsp. holarctica F92 Francisella tularensis subsp. holarctica FSC200 Francisella tularensis subsp. holarctica PHIT-FT049 Francisella tularensis subsp. holarctica FTT\_1 Francisella tularensis subsp. holarctica VT68 Francisella tularensis subsp. holarctica 425 Francisella tularensis subsp. mediasiatica FSC147 Francisella tularensis subsp. novicida U112 Francisella tularensis subsp. novicida U112 Francisella tularensis subsp. novicida F6168 Francisella tularensis subsp. novicida DPG 3A-IS Francisella cf. novicida Fx1 Francisella hispaniensis 3523 Francisella hispaniensis FSC454 Francisella philomiragia subsp. philomiragia ATCC 25017 Francisella philomiragia subsp. philomiragia ATCC 25015 O#319L Francisella philomiragia O#319-029 Francisella philomiragia O#319-036 Francisella philomiragia O#319-067 Francisella philomiragia GA01-2794 Francisella philomiragia GA01-2801 Francisella salina Francisella noatunensis subsp. orientalis Toba 04 Francisella noatunensis subsp. orientalis LADL--07-285A Francisella sp. FSC1006 Francisella persica Francisella halioticida Francisella uliginis Francisella sp. MA067296 Francisella frigiditurris Francisella adeliensis Francisella marina Francisella opportunistica Allofrancisella guangzhouensis Hydrogenovibrio crunogenus Hydrogenovibrio thermophilus Thiomicrospira cyclica Thiomicrospira aerophila Thiomicrospira sp. S5 Methylophaga nitratireducenticrescens Methylophaga frappieri Cycloclasticus sp. P1 Cycloclasticus zancles Cycloclasticus sp. PY97N Piscirickettsia salmonis Thiomicrorhabdus sp. G1 Thioploca ingrica Beggiatoa leptomitiformis Nitrosococcus oceani Nitrosococcus halophilus Nitrosococcus watsonii Nitrosococcus wardiae Allochromatium vinosum Thiocystis violascens Thioflavicoccus mobilis Marichromatium purpuratum Candidatus Tenderia electrophaga Candidatus Nitrosoglobus terrae Candidatus Thiodictyon syntrophicum Rheinheimera sp. D18 Alkalilimnicola ehrlichii Halorhodospira halophila Halorhodospira halochloris Ectothiorhodospira haloalkaliphila Ectothiorhodospira sp. BSL-9 Thioalkalivibrio sulfidiphilus Thioalkalivibrio sp. K90mix Thioalkalivibrio nitratireducens Thioalkalivibrio paradoxus Thioalkalivibrio versutus Spiribacter salinus Spiribacter curvatus Spiribacter roseus Spiribacter sp. 2438 Acidihalobacter prosperus Halothiobacillus neapolitanus Halothiobacillus sp. LS2 Wenzhouxiangella marina Woeseia oceani Granulosicoccus antarcticus Guyparkeria halophila Thiohalobacter thiocyanaticus Hahella chejuensis Hahella sp. KA22 Chromohalobacter salexigens Halomonas elongata Halomonas campaniensis Halomonas sp. KO116 Halomonas sp. R57-5 Halomonas huangheensis Halomonas chromatireducens Halomonas aestuarii Halomonas sp. 1513 Halomonas hydrothermalis Halomonas beimenensis Halomonas sp. GFAJ-1 Halomonas sp. SF2003 Halomonas alkaliphila Halomonas venusta Halomonas olivaria Halomonas sulfidaeris Halomonas meridiana Candidatus Portiera aleyrodidarum BT-B Candidatus Portiera aleyrodidarum BT-B-HRs Candidatus Portiera aleyrodidarum BT-QVLC Candidatus Portiera aleyrodidarum BT-QVLC Candidatus Portiera aleyrodidarum TV Candidatus Portiera aleyrodidarum TV-BCN Candidatus Portiera aleyrodidarum AD-CAI Candidatus Portiera aleyrodidarum AF-CAI Candidatus Portiera aleyrodidarum MED Candidatus Evansia muelleri Zymobacter palmae Halotalea alkalilenta Cobetia marina Kushneria konosiri Kushneria marisflavi Kushneria sp. YCWA18 Pistricoccus aurantiacus Alcanivorax borkumensis Alcanivorax dieselolei Alcanivorax pacificus Alcanivorax sp. NBRC 101098 Alcanivorax xenomutans Ketobacter alkanivorans Kangiella koreensis Kangiella geojedonensis Kangiella sediminilitoris Kangiella profundi Marinomonas sp. MWYL1 Marinomonas mediterranea Marinomonas posidonica Thalassolituus oleivorans MIL-1 Thalassolituus oleivorans R6-15 Oleispira antarctica Marinobacterium aestuarii Bacterioplanes sanyensis Neptunomonas concharum Nitrincola sp. KXZD1103 Gynuella sunshinyii Reinekea forsetii Oleiphilus messinensis Litoricola lipolytica Aeromonas hydrophila subsp. hydrophila ATCC 7966 Aeromonas hydrophila ML09-119 Aeromonas hydrophila YL17 Aeromonas hydrophila AL09-71 Aeromonas hydrophila pc104A Aeromonas hydrophila J-1 Aeromonas hydrophila AL06-06 Aeromonas hydrophila AH10 Aeromonas hydrophila MX16A Aeromonas salmonicida subsp. salmonicida A449 Aeromonas salmonicida O23A Aeromonas veronii B565 Aeromonas veronii TH0426 Aeromonas media Aeromonas schubertii Aeromonas dhakensis Aeromonas caviae Aeromonas sp. CU5 Aeromonas sp. ASNIH3 Aeromonas rivipollensis Aeromonas sp. ASNIH5 Tolumonas auensis Oceanimonas sp. GK1 Oceanisphaera avium Oceanisphaera profunda Zobellella denitrificans Dichelobacter nodosus Cardiobacterium hominis Gilliamella apicola Frischella perrara Steroidobacter denitrificans Solimonas sp. K1W22B-7 Sinimarinibacterium sp. NLF-5-8 Immundisolibacter cernigliae Sulfuricaulis limicola Sulfurifustis variabilis Acidiferrobacter sp. SPIII\_3 Salinisphaera sp. LB1 Thiolapillus brandeum Sedimenticola thiotaurini Candidatus Thioglobus singularis PS1 Candidatus Thioglobus sp. NP1 Candidatus Thioglobus autotrophicus Pseudohongiella spirulinae Endosymbiont of Sipalinus gigas Candidatus Ruthia magnifica (Calyptogena magnifica) Candidatus Vesicomyosocius okutanii (Calyptogena okutanii) Endosymbiont of Bathymodiolus septemdierum Baumannia cicadellinicola Hc (Homalodisca coagulata) Candidatus Baumannia cicadellinicola BGSS Candidatus Baumannia cicadellinicola B-GSS Gamma proteobacterium HdN1 Endosymbiont of unidentified scaly snail isolate Monju Neisseria meningitidis MC58 (serogroup B) Neisseria meningitidis alpha710 (serogroup B) Neisseria meningitidis H44/76 (serogroup B) Neisseria meningitidis G2136 (serogroup B) Neisseria meningitidis M01-240149 (serogroup B) Neisseria meningitidis M01-240355 (serogroup B) Neisseria meningitidis M04-240196 (serogroup B) Neisseria meningitidis NZ-05/33 (serogroup B) Neisseria meningitidis Z2491 (serogroup A) Neisseria meningitidis WUE 2594 (serogroup A) Neisseria meningitidis 510612 (serogroup A) Neisseria meningitidis FAM18 (serogroup C) Neisseria meningitidis 053442 (serogroup C) Neisseria meningitidis 8013 (serogroup C) Neisseria meningitidis alpha14 (cnl strain) Neisseria gonorrhoeae FA 1090 Neisseria gonorrhoeae NCCP11945 Neisseria lactamica Neisseria elongata Neisseria weaveri Neisseria mucosa FDAARGOS\_260 Neisseria mucosa ATCC 19696 Neisseria chenwenguii Neisseria sp. KEM232 Neisseria flavescens Neisseria subflava Neisseria zalophi Neisseria animalis Snodgrassella alvi Kingella kingae Vitreoscilla filiformis Vitreoscilla sp. C1 Eikenella corrodens Eikenella exigua Simonsiella muelleri Neisseriaceae bacterium DSM 100970 Chromobacterium violaceum Chromobacterium vaccinii Chromobacterium sp. ATCC 53434 Chromobacterium sp. IIBBL 112-1 Chromobacterium rhizoryzae Chromobacterium sp. IIBBL 274-1 Chromobacterium sp. 257-1 Iodobacter sp. H11R3 Laribacter hongkongensis Pseudogulbenkiania sp. NH8B Jeongeupia sp. USM3 Aquaspirillum sp. LM1 Aquitalea magnusonii Aquitalea sp. USM4 Ralstonia solanacearum GMI1000 Ralstonia solanacearum CFBP2957 Ralstonia solanacearum PSI07 Ralstonia solanacearum Po82 Ralstonia solanacearum CMR15 Ralstonia solanacearum FQY\_4 Ralstonia solanacearum UY031 Ralstonia pickettii 12J Ralstonia pickettii 12D Ralstonia pickettii DTP0602 Ralstonia mannitolilytica Ralstonia insidiosa Ralstonia pseudosolanacearum Cupriavidus necator H16 Cupriavidus necator N-1 Cupriavidus necator NH9 Cupriavidus pinatubonensis JMP134 Cupriavidus metallidurans Cupriavidus taiwanensis Cupriavidus basilensis Cupriavidus gilardii Cupriavidus sp. USMAHM13 Cupriavidus malaysiensis Cupriavidus sp. USMAA2-4 Cupriavidus pauculus Cupriavidus oxalaticus Burkholderia mallei ATCC 23344 Burkholderia mallei SAVP1 Burkholderia mallei NCTC 10229 Burkholderia mallei NCTC 10247 Burkholderia mallei 23344 Burkholderia mallei 6 Burkholderia mallei BMQ Burkholderia mallei 2000031063 Burkholderia mallei FMH 23344 Burkholderia mallei NCTC 10247 Burkholderia mallei 2002734299 Burkholderia pseudomallei K96243 Burkholderia pseudomallei 1710b Burkholderia pseudomallei 1106a Burkholderia pseudomallei 668 Burkholderia pseudomallei MSHR346 Burkholderia pseudomallei MSHR305 Burkholderia pseudomallei MSHR511 Burkholderia pseudomallei MSHR146 Burkholderia pseudomallei MSHR520 Burkholderia pseudomallei 1026b Burkholderia pseudomallei BPC006 Burkholderia pseudomallei NCTC 13179 Burkholderia pseudomallei HBPUB10134a Burkholderia pseudomallei NAU35A-3 Burkholderia pseudomallei A79A Burkholderia pseudomallei TSV202 Burkholderia thailandensis E264 Burkholderia thailandensis 2002721723 Burkholderia thailandensis E444 Burkholderia thailandensis H0587 Burkholderia thailandensis MSMB121 Burkholderia thailandensis MSMB59 Burkholderia thailandensis E254 Burkholderia thailandensis USAMRU Malaysia #20 Burkholderia thailandensis 2003015869 Burkholderia thailandensis 2002721643 Burkholderia oklahomensis E0147 Burkholderia oklahomensis C6786 Burkholderia sp. BDU6 Burkholderia vietnamiensis G4 Burkholderia vietnamiensis LMG 10929 Burkholderia lata Burkholderia cenocepacia AU1054 Burkholderia cenocepacia HI2424 Burkholderia cenocepacia MC0-3 Burkholderia cenocepacia J2315 Burkholderia cenocepacia DDS 22E-1 Burkholderia cenocepacia DWS 37E-2 Burkholderia cenocepacia H111 Burkholderia ambifaria AMMD Burkholderia ambifaria MC40-6 Burkholderia multivorans ATCC 17616 (Tohoku) Burkholderia multivorans ATCC 17616 (JGI) Burkholderia multivorans DDS 15A-1 Burkholderia multivorans ATCC BAA-247 Burkholderia cepacia GG4 Burkholderia cepacia DDS 7H-2 Burkholderia cepacia ATCC 25416 Burkholderia dolosa Burkholderia pyrrocinia Burkholderia contaminans Burkholderia ubonensis Burkholderia diffusa Burkholderia latens Burkholderia territorii Burkholderia seminalis Burkholderia pseudomultivorans Burkholderia metallica Burkholderia stagnalis Burkholderia stabilis Burkholderia glumae BGR1 Burkholderia glumae LMG 2196 = ATCC 33617 Burkholderia sp. CCGE1001 Burkholderia sp. CCGE1003 Burkholderia gladioli BSR3 Burkholderia gladioli ATCC 10248 Burkholderia sp. YI23 Burkholderia sp. KJ006 Burkholderia insecticola Burkholderia sp. RPE67 Burkholderia sp. 2002721687 Burkholderia sp. HB1 Burkholderia plantarii PG1 Burkholderia plantarii ATCC 43733 Burkholderia sp. Bp5365 Burkholderia sp. OLGA172 Burkholderia sp. PAMC 26561 Burkholderia sp. PAMC 28687 Paraburkholderia xenovorans LB400 Paraburkholderia xenovorans LB400 Paraburkholderia phymatum Paraburkholderia sp. CCGE1002 Paraburkholderia phenoliruptrix Paraburkholderia phytofirmans Paraburkholderia fungorum Paraburkholderia caribensis Paraburkholderia sprentiae Paraburkholderia sp. SOS3 Paraburkholderia aromaticivorans Paraburkholderia hospita Paraburkholderia terrae Paraburkholderia graminis Paraburkholderia caledonica Paraburkholderia terricola Paraburkholderia caffeinilytica Paraburkholderia rhizoxinica Polynucleobacter asymbioticus Polynucleobacter necessarius Polynucleobacter duraquae Polynucleobacter paneuropaeus Pandoraea pnomenusa 3kgm Pandoraea pnomenusa RB38 Pandoraea pnomenusa DSM 16536 Pandoraea pnomenusa Pandoraea pulmonicola Pandoraea sputorum Pandoraea apista Pandoraea vervacti Pandoraea oxalativorans Pandoraea thiooxydans Pandoraea faecigallinarum Pandoraea norimbergensis Pandoraea sp. XY-2 Pandoraea fibrosis Paucimonas lemoignei Ephemeroptericola cinctiostellae Lautropia mirabilis Mycoavidus cysteinexigens Caballeronia sp. SBC2 Bordetella pertussis Tohama I Bordetella pertussis CS Bordetella pertussis 18323 Bordetella pertussis B1917 Bordetella pertussis 137 Bordetella parapertussis Bpp5 Bordetella parapertussis 12822 Bordetella bronchiseptica 253 Bordetella bronchiseptica RB50 Bordetella bronchiseptica MO149 Bordetella bronchiseptica S798 Bordetella petrii Bordetella avium Bordetella holmesii ATCC 51541 Bordetella holmesii 44057 Bordetella hinzii Bordetella trematum Bordetella bronchialis Bordetella flabilis Bordetella pseudohinzii Bordetella sp. H567 Bordetella genomosp. 13 Bordetella sp. HZ20 Bordetella sp. J329 Achromobacter xylosoxidans A8 Achromobacter xylosoxidans NH44784-1996 Achromobacter xylosoxidans NBRC 15126 = ATCC 27061 Achromobacter xylosoxidans NCTC10807 Achromobacter denitrificans Achromobacter insolitus Achromobacter spanius Achromobacter sp. AONIH1 Achromobacter sp. B7 Taylorella equigenitalis MCE9 Taylorella equigenitalis ATCC 35865 Taylorella equigenitalis 14/56 Taylorella asinigenitalis MCE3 Taylorella asinigenitalis 14/45 Pusillimonas sp. T7-7 Pusillimonas sp. ye3 Advenella kashmirensis Advenella mimigardefordensis Castellaniella defragrans Basilea psittacipulmonis Alcaligenes faecalis ZD02 Alcaligenes faecalis JQ135 Alcaligenes aquatilis Paenalcaligenes hominis Orrella dioscoreae Oligella urethralis Pigmentiphaga sp. H8 Pigmentiphaga aceris Kerstersia gyiorum Rhodoferax ferrireducens Rhodoferax saidenbachensis Rhodoferax antarcticus Rhodoferax koreense Rhodoferax sp. CHu59-6-5 Rhodoferax sp. Gr-4 Polaromonas sp. JS666 Polaromonas naphthalenivorans Polaromonas sp. SP1 Polaromonas sp. Pch-P Acidovorax citrulli Acidovorax sp. JS42 Acidovorax ebreus Acidovorax avenae Acidovorax sp. KKS102 Acidovorax sp. RAC01 Acidovorax carolinensis NA2 Acidovorax carolinensis P4 Acidovorax carolinensis NA3 Acidovorax carolinensis P3 Acidovorax sp. 1608163 Verminephrobacter eiseniae Delftia acidovorans Delftia sp. Cs1-4 Delftia tsuruhatensis Delftia sp. HK171 Variovorax paradoxus S110 Variovorax paradoxus EPS Variovorax paradoxus B4 Variovorax sp. PAMC 28711 Variovorax boronicumulans Variovorax sp. PMC12 Comamonas thiooxydans Comamonas testosteroni TK102 Comamonas kerstersii Comamonas serinivorans Comamonas sp. NLF 7-7 Alicycliphilus denitrificans BC Alicycliphilus denitrificans K601 Ramlibacter tataouinensis Candidatus Symbiobacter mobilis Ottowia sp. oral taxon 894 Ottowia oryzae Limnohabitans sp. 103DPR2 Limnohabitans sp. 63ED37-2 Hydrogenophaga sp. RAC07 Hydrogenophaga sp. PBC Hydrogenophaga sp. LPB0072 Hydrogenophaga sp. PAMC20947 Hydrogenophaga pseudoflava Hydrogenophaga sp. BPS33 Diaphorobacter polyhydroxybutyrativorans Simplicispira suum Pulveribacter suum Melaminivora sp. SC2-9 Serpentinomonas raichei Serpentinomonas mccroryi Methylibium petroleiphilum Methylibium sp. Pch-M Herminiimonas arsenicoxydans Janthinobacterium sp. Marseille Janthinobacterium agaricidamnosum Janthinobacterium sp. B9-8 Janthinobacterium sp. 1\_2014MBL\_MicDiv Janthinobacterium sp. LM6 Janthinobacterium svalbardensis Janthinobacterium sp. 17J80-10 Janthinobacterium sp. SNU WT3 Herbaspirillum seropedicae SmR1 Herbaspirillum seropedicae Z67 Herbaspirillum hiltneri Herbaspirillum rubrisubalbicans Herbaspirillum sp. meg3 Herbaspirillum huttiense Collimonas fungivorans Collimonas arenae Collimonas pratensis Massilia sp. NR 4-1 Massilia sp. WG5 Massilia violaceinigra Massilia armeniaca Massilia oculi Massilia sp. YMA4 Massilia albidiflava Massilia umbonata Massilia flava Oxalobacter formigenes Undibacterium parvum Noviherbaspirillum sp. UKPF54 Sutterella megalosphaeroides Sutterella sp. KGMB03119 Leptothrix cholodnii Thiomonas intermedia Thiomonas arsenitoxydans Rubrivivax gelatinosus Rubrivivax benzoatilyticus Roseateles depolymerans Paucibacter sp. KCTC 42545 Mitsuaria sp. 7 Rhizobacter gummiphilus Aquabacterium olei Sphaerotilus natans Xylophilus sp. KACC 21265 Burkholderiales bacterium GJ-E10 Burkholderiales bacterium YL45 Polyangium brachysporum Nitrosomonas europaea Nitrosomonas eutropha Nitrosomonas sp. AL212 Nitrosomonas sp. Is79A3 Nitrosomonas communis Nitrosomonas ureae Nitrosomonas stercoris Nitrosospira multiformis Nitrosospira lacus Sulfuritalea hydrogenivorans Methyloversatilis sp. RAC08 Thiobacillus denitrificans Methylobacillus flagellatus Methylotenera mobilis Methylotenera versatilis Methylovorus glucosetrophus Methylovorus sp. MP688 Candidatus Methylopumilus turicensis Candidatus Methylopumilus planktonicus Methylophilus sp. TWE2 Sideroxydans lithotrophicus Gallionella capsiferriformans Sulfuricella denitrificans Sulfuriferula sp. AH1 Ferriphaselus amnicola Aromatoleum aromaticum Azospira oryzae Rugosibacter aromaticivorans Oryzomicrobium terrae Rhodocyclaceae bacterium Thauera-like Dechloromonas aromatica Dechloromonas sp. HYN0024 Azoarcus sp. BH72 Azoarcus sp. KH32C Azoarcus sp. CIB Azoarcus olearius Azoarcus sp. SY39 Azoarcus communis Azoarcus sp. DN11 Azoarcus sp. DD4 Thauera sp. MZ1T Thauera humireducens Thauera chlorobenzoica Thauera sp. K11 Thauera aromatica Zoogloeaceae bacteirum Par-f-2 Accumulibacter phosphatis Candidatus Kinetoplastibacterium crithidii (ex Angomonas deanei ATCC 30255) Candidatus Kinetoplastibacterium crithidii TCC036E Candidatus Kinetoplastibacterium blastocrithidii (ex Strigomonas culicis) Candidatus Kinetoplastibacterium blastocrithidii TCC012E Candidatus Kinetoplastibacterium desouzaii Candidatus Kinetoplastibacterium galatii Candidatus Kinetoplastibacterium oncopeltii Candidatus Kinetoplastibacterium sorsogonicusi Beta proteobacterium CB Betaproteobacteria bacterium UKL13-2 Betaproteobacteria bacterium GR16-43 Helicobacter pylori 26695 Helicobacter pylori 26695 Helicobacter pylori J99 Helicobacter pylori HPAG1 Helicobacter pylori Shi470 Helicobacter pylori Shi112 Helicobacter pylori Shi169 Helicobacter pylori Shi417 Helicobacter pylori G27 Helicobacter pylori P12 Helicobacter pylori B38 Helicobacter pylori B8 Helicobacter pylori PeCan4 Helicobacter pylori PeCan18 Helicobacter pylori SJM180 Helicobacter pylori ELS37 Helicobacter pylori 35A Helicobacter pylori 908 Helicobacter pylori 2017 Helicobacter pylori 2018 Helicobacter pylori Cuz20 Helicobacter pylori F16 Helicobacter pylori F30 Helicobacter pylori F32 Helicobacter pylori F57 Helicobacter pylori Sat464 Helicobacter pylori 52 Helicobacter pylori v225d Helicobacter pylori 83 Helicobacter pylori SNT49 Helicobacter pylori Lithuania75 Helicobacter pylori Gambia94/24 Helicobacter pylori India7 Helicobacter pylori Puno120 Helicobacter pylori Puno135 Helicobacter pylori SouthAfrica7 Helicobacter pylori SouthAfrica20 Helicobacter pylori HUP-B14 Helicobacter pylori 51 Helicobacter pylori XZ274 Helicobacter pylori Rif1 Helicobacter pylori Rif2 Helicobacter pylori Aklavik117 Helicobacter pylori Aklavik86 Helicobacter pylori OK113 Helicobacter pylori OK310 Helicobacter pylori oki102 Helicobacter pylori oki112 Helicobacter pylori oki128 Helicobacter pylori oki154 Helicobacter pylori oki422 Helicobacter pylori oki673 Helicobacter pylori oki828 Helicobacter pylori oki898 Helicobacter pylori UM032 Helicobacter pylori UM037 Helicobacter pylori UM066 Helicobacter pylori UM299 Helicobacter pylori UM298 Helicobacter pylori BM012A Helicobacter pylori BM012S Helicobacter hepaticus Helicobacter acinonychis Helicobacter mustelae Helicobacter felis Helicobacter bizzozeronii Helicobacter cetorum MIT 00-7128 Helicobacter cetorum MIT 99-5656 Helicobacter cinaedi PAGU611 Helicobacter cinaedi CCUG 18818 = ATCC BAA-847 Helicobacter heilmannii Helicobacter typhlonius Helicobacter bilis Helicobacter apodemus Helicobacter sp. MIT 01-6242 Wolinella succinogenes Sulfurimonas denitrificans Sulfurimonas autotrophica Sulfurimonas sp. 1-1N Sulfurimonas sp. GYSZ\_1 Sulfurimonas sp. CVO Sulfuricurvum kujiense Candidatus Sulfuricurvum sp. RIFRC-1 Campylobacter jejuni subsp. jejuni NCTC 11168 = ATCC 700819 Campylobacter jejuni subsp. jejuni NCTC 11168-BN148 Campylobacter jejuni subsp. jejuni 81-176 Campylobacter jejuni subsp. jejuni 81116 Campylobacter jejuni subsp. jejuni ICDCCJ07001 Campylobacter jejuni subsp. jejuni IA3902 Campylobacter jejuni subsp. jejuni M1 Campylobacter jejuni subsp. jejuni S3 Campylobacter jejuni subsp. jejuni PT14 Campylobacter jejuni subsp. jejuni 00-2426 Campylobacter jejuni subsp. jejuni 00-2538 Campylobacter jejuni subsp. jejuni 00-2544 Campylobacter jejuni subsp. jejuni 00-2425 Campylobacter jejuni subsp. jejuni R14 Campylobacter jejuni subsp. jejuni MTVDSCj20 Campylobacter jejuni subsp. jejuni YH001 Campylobacter jejuni subsp. jejuni 35925B2 Campylobacter jejuni subsp. jejuni 00-1597 Campylobacter jejuni subsp. jejuni 00-6200 Campylobacter jejuni RM1221 Campylobacter jejuni subsp. doylei 269.97 Campylobacter jejuni 32488 Campylobacter jejuni 4031 Campylobacter fetus subsp. fetus 82-40 Campylobacter fetus subsp. fetus 04/554 Campylobacter fetus subsp. venerealis cfvi03/293 Campylobacter fetus subsp. venerealis 97/608 Campylobacter fetus subsp. venerealis 84-112 Campylobacter fetus subsp. testudinum 03-427 Campylobacter fetus subsp. testudinum pet-3 Campylobacter curvus Campylobacter hominis Campylobacter concisus 13826 Campylobacter concisus ATCC 33237 Campylobacter lari RM2100 Campylobacter lari RM16701 Campylobacter lari RM16712 Campylobacter lari CCUG 22395 Campylobacter lari NCTC 11845 Campylobacter lari subsp. concheus LMG 11760 Campylobacter coli 76339 Campylobacter coli CVM N29710 Campylobacter coli 15-537360 Campylobacter coli RM1875 Campylobacter coli RM4661 Campylobacter coli RM5611 Campylobacter coli FB1 Campylobacter coli OR12 Campylobacter iguaniorum Campylobacter insulaenigrae Campylobacter volucris Campylobacter peloridis Campylobacter sp. RM16704 Campylobacter subantarcticus LMG 24374 Campylobacter subantarcticus LMG 24377 Campylobacter gracilis Campylobacter ureolyticus Campylobacter hyointestinalis Campylobacter helveticus Campylobacter sputorum Campylobacter pinnipediorum Campylobacter cuniculorum Campylobacter lanienae Campylobacter avium Campylobacter hepaticus Campylobacter sp. RM12175 Campylobacter sp. RM6137 Arcobacter butzleri RM4018 Arcobacter butzleri ED-1 Arcobacter butzleri 7h1h Arcobacter nitrofigilis Arcobacter skirrowii Arcobacter halophilus Arcobacter mytili Arcobacter marinus Arcobacter trophiarum Arcobacter porcinus Halarcobacter bivalviorum Arcobacter sp. L Arcobacter sp. LPB0137 Sulfurospirillum deleyianum Sulfurospirillum barnesii Sulfurospirillum multivorans Sulfurospirillum halorespirans Sulfurospirillum sp. SL2-1 Sulfurospirillum sp. JPD-1 Hydrogenimonas sp. MAG Nitratifractor salsuginis Nitratiruptor sp. SB155-2 Sulfurovum sp. NBC37-1 Sulfurovum lithotrophicum Nautilia profundicola Nautilia sp. PV-1 Caminibacter mediatlanticus Cetia pacifica Geobacter sulfurreducens PCA Geobacter sulfurreducens KN400 Geobacter metallireducens Geobacter uraniireducens Geobacter lovleyi Geobacter bemidjiensis Geobacter daltonii FRC-32 Geobacter sp. M21 Geobacter sp. M18 Geobacter pickeringii Geobacter anodireducens Geoalkalibacter subterraneus Pelobacter carbinolicus Pelobacter propionicus Pelobacter acetylenicus Pelobacter sp. SFB93 Desulfuromonas soudanensis Desulfuromonas sp. DDH964 Desulfovibrio vulgaris Hildenborough Desulfovibrio vulgaris DP4 Desulfovibrio vulgaris Miyazaki F Desulfovibrio vulgaris RCH1 Desulfovibrio alaskensis Desulfovibrio desulfuricans ATCC 27774 Desulfovibrio desulfuricans ND132 Desulfovibrio magneticus Desulfovibrio salexigens Desulfovibrio hydrothermalis Desulfovibrio gigas Desulfovibrio fairfieldensis Desulfovibrio piger Desulfovibrio sp. G11 Candidatus Desulfovibrio trichonymphae Desulfovibrio ferrophilus Desulfovibrio africanus Pseudodesulfovibrio aespoeensis Pseudodesulfovibrio piezophilus Pseudodesulfovibrio indicus Pseudodesulfovibrio profundus Pseudodesulfovibrio sp. SRB007 Lawsonia intracellularis PHE/MN1-00 Lawsonia intracellularis N343 Desulfomicrobium baculatum Desulfomicrobium orale Desulfohalobium retbaense Desulfotalea psychrophila Desulfurivibrio alkaliphilus Desulfobulbus propionicus Desulfobulbus oralis Desulfocapsa sulfexigens Desulfococcus oleovorans Desulfococcus multivorans Desulfatibacillum alkenivorans Desulfobacterium autotrophicum Desulfobacula toluolica Desulfosarcina ovata Desulfosarcina widdelii Anaeromyxobacter dehalogenans 2CP-C Anaeromyxobacter dehalogenans 2CP-1 Anaeromyxobacter sp. Fw109-5 Anaeromyxobacter sp. K Myxococcus xanthus Myxococcus fulvus Myxococcus stipitatus Myxococcus hansupus Myxococcus macrosporus Corallococcus coralloides Stigmatella aurantiaca Archangium gephyra Melittangium boletus Cystobacter fuscus Vulgatibacter incomptus Sorangium cellulosum So ce56 Sorangium cellulosum So0157-2 Chondromyces crocatus Sandaracinus amylolyticus Labilithrix luteola Minicystis rosea Haliangium ochraceum Syntrophus aciditrophicus Desulfobacca acetoxidans Desulfomonile tiedjei Syntrophobacter fumaroxidans Desulfoglaeba alkanexedens Desulfarculus baarsii Hippea maritima Desulfurella acetivorans Bradymonas sediminis Bdellovibrio bacteriovorus HD100 Bdellovibrio bacteriovorus Tiberius Bdellovibrio bacteriovorus W Bdellovibrio bacteriovorus 109J Bdellovibrio exovorus Bdellovibrio sp. qaytius Bdellovibrio sp. NC01 Bdellovibrio sp. ZAP7 Halobacteriovorax marinus Halobacteriovorax sp. BALOs\_7 Bacteriovorax stolpii Silvanigrellales bacterium RF1110005 Rickettsia prowazekii Madrid E Rickettsia prowazekii BuV67-CWPP Rickettsia prowazekii Chernikova Rickettsia prowazekii Dachau Rickettsia prowazekii GvV257 Rickettsia prowazekii Katsinyian Rickettsia prowazekii RpGvF24 Rickettsia prowazekii Rp22 Rickettsia prowazekii Breinl Rickettsia prowazekii NMRC Madrid E Rickettsia typhi Wilmington Rickettsia typhi TH1527 Rickettsia typhi B9991CWPP Rickettsia canadensis McKiel Rickettsia canadensis CA410 Rickettsia bellii RML369-C Rickettsia bellii OSU 85-389 Rickettsia conorii Rickettsia felis Rickettsia akari Rickettsia rickettsii Sheila Smith Rickettsia rickettsii Iowa Rickettsia rickettsii Arizona Rickettsia rickettsii Colombia Rickettsia rickettsii Hauke Rickettsia rickettsii Brazil Rickettsia rickettsii Hino Rickettsia rickettsii Hlp#2 Rickettsia rickettsii Morgan Rickettsia rickettsii R Rickettsia massiliae MTU5 Rickettsia massiliae AZT80 Rickettsia peacockii Rickettsia africae Rickettsia heilongjiangensis Rickettsia japonica Rickettsia slovaca 13-B Rickettsia slovaca D-CWPP Rickettsia philipii Rickettsia australis Rickettsia montanensis Rickettsia parkeri Rickettsia rhipicephali Rickettsia amblyommatis GAT-30V Rickettsia amblyommatis Ac37 Rickettsia monacensis Rickettsia asiatica Rickettsia sp. MEAM1 (Bemisia tabaci) Orientia tsutsugamushi Boryong Orientia tsutsugamushi Ikeda Candidatus Phycorickettsia trachydisci Wolbachia wMel (Drosophila melanogaster) Wolbachia wRi (Drosophila simulans) Wolbachia wHa (Drosophila simulans) Wolbachia wNo (Drosophila simulans) Wolbachia wPip (Culex quinquefasciatus) Wolbachia wBm (Brugia malayi) Wolbachia wOo (Onchocerca ochengi) Wolbachia wCle (Cimex lectularius) Wolbachia wFol (Folsomia candida) Wolbachia pipientis Anaplasma marginale St. Maries Anaplasma marginale Florida Anaplasma marginale Dawn Anaplasma marginale Gypsy Plains Anaplasma centrale Anaplasma ovis Neorickettsia helminthoeca Candidatus Midichloria mitochondrii Candidatus Fokinia solitaria Rickettsiales bacterium Ac37b Rickettsiales endosymbiont of Stachyamoeba lipophora Candidatus Paracaedibacter acanthamoebae Candidatus Paracaedimonas acanthamoebae Candidatus Nucleicultrix amoebiphila Endosymbiont of Acanthamoeba sp. UWC8 Mesorhizobium japonicum MAFF 303099 Mesorhizobium loti NZP2037 Mesorhizobium ciceri (biovar Biserrulae) Mesorhizobium opportunistum Mesorhizobium australicum Mesorhizobium amorphae Mesorhizobium sp. B7 Mesorhizobium sp. WSM1497 Mesorhizobium sp. M9A.F.Ca.ET.002.03.1.2 Mesorhizobium sp. Pch-S Mesorhizobium huakuii Chelativorans sp. BNC1 Hoeflea sp. IMCC20628 Aminobacter aminovorans Aminobacter sp. MSH1 Phyllobacterium zundukense Roseitalea porphyridii Parvibaculum lavamentivorans Rhodobiaceae bacterium SMS8 Sinorhizobium meliloti 1021 Sinorhizobium meliloti AK83 Sinorhizobium meliloti BL225C Sinorhizobium meliloti SM11 Sinorhizobium meliloti Rm41 Sinorhizobium meliloti GR4 Sinorhizobium meliloti 2011 Sinorhizobium meliloti RMO17 Sinorhizobium medicae Sinorhizobium fredii NGR234 Sinorhizobium fredii HH103 Sinorhizobium fredii USDA 257 Sinorhizobium sp. RAC02 Sinorhizobium americanum Sinorhizobium sp. CCBAU 05631 Ensifer adhaerens OV14 Ensifer adhaerens Casida A Ensifer sojae Ensifer alkalisoli Agrobacterium fabrum Agrobacterium radiobacter Agrobacterium tumefaciens Ach5 Agrobacterium tumefaciens S33 Agrobacterium vitis Agrobacterium sp. H13-3 Agrobacterium sp. RAC06 Agrobacterium rhizogenes Agrobacterium sp. 33MFTa1.1 Agrobacterium larrymoorei Rhizobium etli CFN 42 Rhizobium etli CIAT 652 Rhizobium etli bv. mimosae Mim1 Rhizobium etli bv. phaseoli IE4803 Rhizobium sp. IE4771 Rhizobium leguminosarum bv. viciae 3841 Rhizobium leguminosarum bv. trifolii WSM2304 Rhizobium leguminosarum bv. trifolii WSM1325 Rhizobium leguminosarum bv. trifolii WSM1689 Rhizobium leguminosarum bv. trifolii CB782 Rhizobium tropici Rhizobium sp. IRBG74 Rhizobium favelukesii Rhizobium gallicum Rhizobium sp. N1341 Rhizobium phaseoli Rhizobium sp. NT-26 Rhizobium sp. N731 Rhizobium sp. S41 Rhizobium sp. Kim5 Rhizobium esperanzae Rhizobium jaguaris Rhizobium sp. 11515TR Rhizobium grahamii Rhizobium acidisoli Neorhizobium galegae bv. officinalis bv. officinalis HAMBI 1141 Neorhizobium galegae bv. orientalis HAMBI 540 Neorhizobium sp. SOG26 Neorhizobium sp. NCHU2750 Candidatus Liberibacter asiaticus psy62 Candidatus Liberibacter asiaticus gxpsy Candidatus Liberibacter asiaticus Ishi-1 Candidatus Liberibacter solanacearum Liberibacter crescens Candidatus Liberibacter americanus Candidatus Liberibacter africanus Shinella sp. HZN7 Georhizobium profundi Brucella melitensis bv. 1 16M Brucella melitensis bv. 1 16M Brucella melitensis ATCC 23457 Brucella melitensis M28 Brucella melitensis M5-90 Brucella melitensis NI Brucella melitensis bv. 3 Ether Brucella abortus 2308 Brucella abortus bv. 1 9-941 Brucella abortus S19 Brucella abortus A13334 Brucella abortus bv. 2 86/8/59 Brucella abortus bv. 6 870 Brucella abortus 63 75 Brucella abortus BDW Brucella abortus bv. 9 C68 Brucella abortus BER Brucella abortus NCTC 10505 Brucella suis 1330 Brucella suis 1330 Brucella suis bv. 1 Brucella suis bv. 2 Bs143CITA Brucella suis bv. 2 PT09143 Brucella suis bv. 2 PT09172 Brucella suis bv. 2 Bs364CITA Brucella suis ATCC 23445 Brucella suis bv. 3 Brucella suis VBI22 Brucella suis ZW043 Brucella suis ZW046 Brucella ovis Brucella canis ATCC 23365 Brucella canis HSK A52141 Brucella canis Oliveri Brucella canis RM6/66 Brucella canis SVA13 Brucella microti Brucella pinnipedialis B2/94 Brucella pinnipedialis 6/566 Brucella ceti TE10759-12 Brucella ceti TE28753-12 Brucella vulpis Brucella sp. 2002734562 Brucella sp. 09RB8471 Ochrobactrum anthropi ATCC 49188 Ochrobactrum anthropi OAB Ochrobactrum pseudogrignonense Ochrobactrum sp. A44 Bradyrhizobium diazoefficiens USDA 110 Bradyrhizobium japonicum USDA 6 Bradyrhizobium japonicum E109 Bradyrhizobium sp. ORS 278 Bradyrhizobium sp. BTAi1 Bradyrhizobium sp. S23321 Bradyrhizobium oligotrophicum Bradyrhizobium sp. CCGE-LA001 Bradyrhizobium sp. BF49 Bradyrhizobium icense Bradyrhizobium sp. ORS 285 Bradyrhizobium sp. SK17 Bradyrhizobium ottawaense Bradyrhizobium amphicarpaeae Bradyrhizobium guangdongense Bradyrhizobium guangzhouense Bradyrhizobium symbiodeficiens Bradyrhizobium betae Rhodopseudomonas palustris CGA009 Rhodopseudomonas palustris HaA2 Rhodopseudomonas palustris BisB18 Rhodopseudomonas palustris BisB5 Rhodopseudomonas palustris BisA53 Rhodopseudomonas palustris TIE-1 Rhodopseudomonas palustris DX-1 Nitrobacter winogradskyi Nitrobacter hamburgensis Oligotropha carboxidovorans OM5 (Mississippi) Oligotropha carboxidovorans OM5 (Goettingen) Oligotropha carboxidovorans OM4 Bosea sp. PAMC 26642 Bosea sp. RAC05 Bosea vaviloviae Bosea sp. Tri-49 Bosea sp. F3-2 Variibacter gotjawalensis Bartonella henselae Houston-1 Bartonella henselae BM1374163 Bartonella henselae BM1374165 Bartonella quintana Toulouse Bartonella quintana RM-11 Bartonella bacilliformis Bartonella tribocorum CIP 105476 Bartonella tribocorum BM1374166 Bartonella grahamii Bartonella clarridgeiae Bartonella australis Bartonella vinsonii Bartonella ancashensis Bartonella apis Bartonella sp. JB15 Bartonella sp. A1379B Bartonella sp. WD16.2 Bartonella sp. Raccoon60 Bartonella sp. 1-1C Bartonella sp. JB63 Bartonella elizabethae Bartonella krasnovii OE 1-1 Xanthobacter autotrophicus Azorhizobium caulinodans Starkeya novella Labrys neptuniae Ancylobacter sp. TS-1 Ancylobacter pratisalsi Methylorubrum extorquens AM1 Methylorubrum extorquens DM4 Methylorubrum extorquens PA1 Methylorubrum extorquens CM4 Methylorubrum populi Methylorubrum zatmanii Methylobacterium radiotolerans Methylobacterium sp. 4-46 Methylobacterium nodulans Methylobacterium oryzae Methylobacterium sp. AMS5 Methylobacterium aquaticum Methylobacterium phyllosphaerae Methylobacterium currus Methylobacterium sp. DM1 Methylobacterium sp. XJLW Methylobacterium sp. 17SD2-17 Methylobacterium sp. 17Sr1-43 Methylobacterium mesophilicum Methylobacterium terrae Microvirga ossetica Microvirga sp. 17 mud 1-3 Microvirga sp. HR1 Beijerinckia indica Methylocella silvestris Methylocella tundrae Methylovirgula ligni Beijerinckiaceae bacterium RH AL1 Chelatococcus sp. CO-6 Chelatococcus daeguensis Hyphomicrobium denitrificans ATCC 51888 Hyphomicrobium denitrificans 1NES1 Hyphomicrobium sp. MC1 Hyphomicrobium nitrativorans Rhodomicrobium vannielii Pelagibacterium halotolerans Candidatus Filomicrobium marinum W Candidatus Filomicrobium marinum Y Devosia sp. H5989 Devosia sp. I507 Devosia ginsengisoli Blastochloris viridis Blastochloris sp. GI Rhodoplanes sp. Z2-YC6860 Maritalea myrionectae Youhaiella tibetensis Methylocystis sp. SC2 Methylocystis bryophila Methylocystis rosea Methylocystis heyeri Methylocystis parvus Methylosinus trichosporium Pleomorphomonas sp. SM30 Martelella endophytica Martelella sp. AD-3 Martelella mediterranea Aureimonas sp. AU20 Breoghania sp. L-A4 Methyloceanibacter caenitepidi Candidatus Tokpelaia hoelldoblerii Pseudorhodoplanes sinuspersici Hartmannibacter diazotrophicus Nordella sp. HKS 07 Rhizobiales bacterium NRL2 Caulobacter vibrioides CB15 Caulobacter vibrioides NA1000 Caulobacter sp. K31 Caulobacter segnis Caulobacter henricii Caulobacter mirabilis Caulobacter flavus Caulobacter sp. FWC26 Phenylobacterium zucineum Phenylobacterium sp. HYN0004 Brevundimonas subvibrioides Brevundimonas sp. DS20 Brevundimonas naejangsanensis Brevundimonas sp. GW460-12-10-14-LB2 Brevundimonas sp. LM2 Brevundimonas vesicularis Brevundimonas diminuta Brevundimonas sp. MF30-B Brevundimonas sp. SGAir0440 Asticcacaulis excentricus Caulobacteraceae bacterium Ruegeria pomeroyi Ruegeria sp. TM1040 Ruegeria sp. AD91A Ruegeria sp. THAF33 Epibacterium mobile Rhodobacter sphaeroides 2.4.1 Rhodobacter sphaeroides ATCC 17029 Rhodobacter sphaeroides ATCC 17025 Rhodobacter sphaeroides KD131 Rhodobacter capsulatus Rhodobacter sp. LPB0142 Rhodobacter blasticus Jannaschia sp. CCS1 Roseobacter denitrificans Roseobacter litoralis Paracoccus denitrificans Paracoccus aminophilus Paracoccus yeei Paracoccus contaminans Paracoccus zhejiangensis Paracoccus sp. BM15 Paracoccus jeotgali Paracoccus aminovorans Paracoccus mutanolyticus Paracoccus sp. SC2-6 Paracoccus sp. Arc7-R13 Paracoccus kondratievae Dinoroseobacter shibae Ketogulonicigenium vulgare WSH-001 Ketogulonicigenium vulgare Y25 Ketogulonicigenium robustum Pseudovibrio sp. FO-BEG1 Phaeobacter inhibens DSM 17395 Phaeobacter inhibens 2.10 Phaeobacter gallaeciensis DSM 26640 Phaeobacter porticola Phaeobacter piscinae Phaeobacter sp. LSS9 Octadecabacter antarcticus Octadecabacter arcticus Octadecabacter temperatus Octadecabacter sp. SW4 Leisingera methylohalidivorans Leisingera sp. NJS204 Leisingera aquaemixtae Roseibacterium elongatum Planktomarina temperata Celeribacter indicus Celeribacter marinus Celeribacter ethanolicus Celeribacter manganoxidans Marinovum algicola Confluentimicrobium sp. EMB200-NS6 Rhodovulum sulfidophilum Rhodovulum sp. MB263 Rhodovulum sp. P5 Pannonibacter phragmitetus Halocynthiibacter arcticus Labrenzia sp. CP4 Labrenzia aggregata Labrenzia sp. VG12 Labrenzia sp. PHM005 Labrenzia sp. THAF35 Defluviimonas alba Yangia sp. CCB-MM3 Yangia pacifica Sulfitobacter sp. AM1-D1 Sulfitobacter pseudonitzschiae Sulfitobacter sp. SK012 Sulfitobacter sp. JL08 Sulfitobacter sp. D7 Sulfitobacter donghicola Marivivens sp. JLT3646 Salipiger profundus Tateyamaria omphalii Pelagibaca abyssi Thioclava nitratireducens Roseovarius mucosus Roseovarius sp. AK1035 Roseovarius indicus Roseovarius sp. MME-070 Roseovarius sp. THAF27 Yoonia vestfoldensis Antarctobacter heliothermus Rhodobaca barguzinensis Sagittula sp. P11 Thalassococcus sp. S3 Gemmobacter sp. HYN0069 Tabrizicola piscis Silicimonas algicola Sedimentitalea sp. W43 Haematobacter massiliensis Boseongicola sp. CCM32 Pseudorhodobacter sp. S12M18 Litoreibacter sp. LN3S51 Oceanicola sp. D3 Maribius sp. THAF1 Roseivivax sp. THAF197b Paraoceanicella profunda Stappia indica Fluviibacterium aquatile Halovulum dunhuangense Pseudohalocynthiibacter aestuariivivens Rhodobacteraceae bacterium G7 Rhodobacteraceae bacterium SH-1 Maricaulis maris Hyphomonas neptunium Hirschia baltica Glycocaulis alkaliphilus Hyphomonadaceae bacterium UKL13-1 Zymomonas mobilis subsp. mobilis ZM4 Zymomonas mobilis subsp. mobilis NCIMB 11163 Zymomonas mobilis subsp. mobilis ATCC 10988 Zymomonas mobilis subsp. mobilis ATCC 29191 Zymomonas mobilis subsp. mobilis CP4 = NRRL B-14023 Zymomonas mobilis subsp. mobilis CP4 = NRRL B-14023 Zymomonas mobilis subsp. mobilis NRRL B-12526 Zymomonas mobilis subsp. pomaceae ATCC 29192 Novosphingobium aromaticivorans Novosphingobium sp. PP1Y Novosphingobium pentaromativorans Novosphingobium resinovorum Novosphingobium sp. P6W Novosphingobium sp. THN1 Novosphingobium tardaugens Novosphingobium sp. ABRDHK2 Novosphingobium ginsenosidimutans Novosphingobium sp. Gsoil 351 Sphingopyxis alaskensis Sphingopyxis fribergensis Sphingopyxis sp. 113P3 Sphingopyxis macrogoltabida EY-1 Sphingopyxis macrogoltabida 203 Sphingopyxis terrae Sphingopyxis granuli Sphingopyxis sp. LPB0140 Sphingopyxis sp. QXT-31 Sphingopyxis sp. MG Sphingopyxis sp. PAMC25046 Uncultured Sphingopyxis sp. UC10 Sphingomonas wittichii Sphingomonas wittichii DC-6 Sphingomonas sp. MM-1 Sphingomonas taxi Sphingomonas hengshuiensis Sphingomonas sanxanigenens Sphingomonas sp. NIC1 Sphingomonas melonis Sphingomonas panacis Sphingomonas koreensis Sphingomonas sp. LM7 Sphingomonas sp. LK11 Sphingomonas sp. KC8 Sphingomonas sp. Cra20 Sphingomonas sp. FARSPH Sphingomonas sp. YZ-8 Sphingomonas paucimobilis Sphingobium japonicum Sphingobium chlorophenolicum Sphingobium sp. SYK-6 Sphingobium sp. YBL2 Sphingobium baderi Sphingobium sp. MI1205 Sphingobium sp. EP60837 Sphingobium sp. RAC03 Sphingobium indicum Sphingobium sp. TKS Sphingobium hydrophobicum Sphingobium yanoikuyae Sphingobium cloacae Sphingobium sp. YG1 Sphingobium fuliginis Citromicrobium sp. JL477 Sphingorhabdus sp. M41 Sphingorhabdus sp. SMR4y Sphingorhabdus sp. YGSMI21 Blastomonas sp. RAC04 Blastomonas fulva Rhizorhabdus dicambivorans Sphingosinicella microcystinivorans Sphingosinicella sp. BN140058 Tardibacter chloracetimidivorans Sphingosinithalassobacter sp. zrk23 Hankyongella ginsenosidimutans Erythrobacter litoralis HTCC2594 Erythrobacter litoralis DSM 8509 Erythrobacter atlanticus Erythrobacter gangjinensis Erythrobacter flavus Erythrobacter sp. KY5 Erythrobacter sp. YH-07 Erythrobacter sp. THAF29 Altererythrobacter atlanticus Altererythrobacter marensis Altererythrobacter epoxidivorans Altererythrobacter namhicola Altererythrobacter dongtanensis Altererythrobacter sp. B11 Altererythrobacter sp. BO-6 Croceicoccus naphthovorans Croceicoccus marinus Porphyrobacter neustonensis Porphyrobacter sp. LM 6 Porphyrobacter sp. HT-58-2 Porphyrobacter sp. YT40 Gluconobacter oxydans 621H Gluconobacter oxydans H24 Gluconobacter oxydans DSM 3504 Gluconobacter albidus Gluconobacter thailandicus Granulibacter bethesdensis CGDNIH1 Granulibacter bethesdensis CGDNIH2 Granulibacter bethesdensis NIH3.1 Granulibacter bethesdensis CGDNIH4 Acidiphilium cryptum Acidiphilium multivorum Gluconacetobacter diazotrophicus PA1 5 (Brazil) Gluconacetobacter diazotrophicus PA1 5 (JGI) Komagataeibacter medellinensis Komagataeibacter xylinus Komagataeibacter nataicola Komagataeibacter europaeus Komagataeibacter saccharivorans Acetobacter pasteurianus IFO 3283-01 Acetobacter pasteurianus IFO 3283-01-42C Acetobacter pasteurianus IFO 3283-03 Acetobacter pasteurianus IFO 3283-07 Acetobacter pasteurianus IFO 3283-12 Acetobacter pasteurianus IFO 3283-22 Acetobacter pasteurianus IFO 3283-26 Acetobacter pasteurianus IFO 3283-32 Acetobacter pasteurianus 386B Acetobacter senegalensis Acetobacter oryzifermentans Acetobacter aceti Acetobacter persici Acetobacter pomorum Acetobacter tropicalis Acetobacter ascendens Acetobacter sp. JWB Acetobacter orientalis Acetobacter oryzoeni Asaia bogorensis Kozakia baliensis Roseomonas gilardii Roseomonas sp. FDAARGOS\_362 Roseomonas mucosa Neoasaia chiangmaiensis Commensalibacter sp. AMU001 Commensalibacter sp. ESL0284 Stella humosa Stella vacuolata Neokomagataea tanensis Neokomagataea sp. Ha5 Swingsia samuiensis Swingsia sp. F3b2 Bombella sp. ESL0368 Bombella sp. KACC 21507 Rhodospirillum rubrum ATCC 11170 Rhodospirillum rubrum F11 Rhodospirillum centenum Pararhodospirillum photometricum Magnetospirillum magneticum Magnetospirillum gryphiswaldense MSR-1 v2 Magnetospirillum gryphiswaldense MSR-1 Magnetospirillum sp. XM-1 Magnetospirillum sp. ME-1 Azospirillum sp. B510 Azospirillum lipoferum Azospirillum brasilense Sp245 Azospirillum brasilense Az39 Azospirillum brasilense Sp7 Azospirillum thiophilum Azospirillum humicireducens Azospirillum sp. TSH58 Azospirillum sp. M2T2B2 Azospirillum sp. CFH 70021 Tistrella mobilis Candidatus Endolissoclinum faulkneri L2 Candidatus Endolissoclinum faulkneri L5 Thalassospira xiamenensis Thalassospira marina Thalassospira indica Magnetospira sp. QH-2 Haematospirillum jordaniae Nitrospirillum amazonense Niveispirillum cyanobacteriorum Ferrovibrio terrae Hypericibacter terrae Hypericibacter adhaerens Parvularcula bermudensis Magnetococcus marinus Candidatus Pelagibacter ubique Candidatus Pelagibacter sp. IMCC9063 Alpha proteobacterium HIMB59 Alpha proteobacterium HIMB5 Emcibacter congregatus Micavibrio aeruginosavorus ARL-13 Micavibrio aeruginosavorus EPB Polymorphum gilvum Phreatobacter cathodiphilus Phreatobacter stygius Candidatus Puniceispirillum marinum Acidithiobacillus ferrooxidans ATCC 23270 Acidithiobacillus ferrooxidans ATCC 53993 Acidithiobacillus caldus SM-1 Acidithiobacillus caldus ATCC 51756 Acidithiobacillus ferrivorans Acidithiobacillus ferridurans Acidithiobacillus thiooxidans Mariprofundus aestuarium Mariprofundus ferrinatatus Hydrogenophilus thermoluteolus Bacillus subtilis subsp. subtilis 168 Bacillus subtilis subsp. subtilis RO-NN-1 Bacillus subtilis subsp. subtilis BSP1 Bacillus subtilis subsp. subtilis 6051-HGW Bacillus subtilis subsp. subtilis BAB-1 Bacillus subtilis subsp. subtilis AG1839 Bacillus subtilis subsp. subtilis JH642 Bacillus subtilis subsp. subtilis OH 131.1 Bacillus subtilis subsp. spizizenii W23 Bacillus subtilis subsp. spizizenii TU-B-10 Bacillus subtilis subsp. natto BEST195 Bacillus subtilis BSn5 Bacillus subtilis QB928 Bacillus subtilis XF-1 Bacillus subtilis PY79 Bacillus licheniformis ATCC 14580 Bacillus licheniformis DSM 13 = ATCC 14580 Bacillus paralicheniformis Bacillus velezensis FZB42 Bacillus velezensis CAU B946 Bacillus velezensis YAU B9601-Y2 Bacillus velezensis AS43.3 Bacillus velezensis UCMB5036 Bacillus velezensis UCMB5033 Bacillus velezensis UCMB5113 Bacillus velezensis NAU-B3 Bacillus velezensis TrigoCor1448 Bacillus velezensis SQR9 Bacillus velezensis JS25R Bacillus amyloliquefaciens DSM 7 Bacillus amyloliquefaciens TA208 Bacillus amyloliquefaciens LL3 Bacillus amyloliquefaciens XH7 Bacillus amyloliquefaciens Y2 Bacillus amyloliquefaciens IT-45 Bacillus amyloliquefaciens CC178 Bacillus amyloliquefaciens LFB112 Bacillus atrophaeus Bacillus vallismortis Bacillus halodurans Bacillus anthracis Ames Bacillus anthracis Ames Ancestor Bacillus anthracis Sterne Bacillus anthracis CDC 684 Bacillus anthracis A0248 Bacillus anthracis H9401 Bacillus anthracis A16 Bacillus anthracis A16R Bacillus anthracis SVA11 Bacillus anthracis HYU01 Bacillus anthracis Vollum Bacillus cereus ATCC 14579 Bacillus cereus ATCC 10987 Bacillus cereus E33L Bacillus cereus AH187 Bacillus cereus B4264 Bacillus cereus AH820 Bacillus cereus G9842 Bacillus cereus Q1 Bacillus cereus 03BB102 Bacillus cereus biovar anthracis CI Bacillus cereus NC7401 Bacillus cereus F837/76 Bacillus cereus FRI-35 Bacillus cereus FT9 Bacillus cytotoxicus Bacillus thuringiensis serovar konkukian 97-27 Bacillus thuringiensis Al Hakam Bacillus thuringiensis BMB171 Bacillus thuringiensis serovar kurstaki HD73 Bacillus thuringiensis serovar kurstaki YBT-1520 Bacillus thuringiensis serovar kurstaki HD-1 Bacillus thuringiensis serovar chinensis CT-43 Bacillus thuringiensis serovar finitimus YBT-020 Bacillus thuringiensis MC28 Bacillus thuringiensis Bt407 Bacillus thuringiensis HD-771 Bacillus thuringiensis HD-789 Bacillus thuringiensis serovar thuringiensis IS5056 Bacillus thuringiensis YBT-1518 Bacillus thuringiensis HD1011 Bacillus thuringiensis YWC2-8 Bacillus mycoides KBAB4 Bacillus mycoides WSBC 10204 Bacillus mycoides ATCC 6462 Bacillus toyonensis Bacillus pseudomycoides 219298 Bacillus bombysepticus Bacillus wiedmannii Bacillus clausii Bacillus pumilus SAFR-032 Bacillus pumilus MTCC B6033 Bacillus pumilus SH-B9 Bacillus pseudofirmus Bacillus megaterium QM B1551 Bacillus megaterium DSM 319 Bacillus megaterium WSH-002 Bacillus megaterium NBRC 15308 = ATCC 14581 Bacillus cellulosilyticus Bacillus coagulans 2-6 Bacillus coagulans 36D1 Bacillus coagulans DSM 1 = ATCC 7050 Bacillus sp. JS Bacillus sp. 1NLA3E Bacillus infantis Bacillus lehensis Bacillus methanolicus Bacillus sp. X1(2014) Bacillus sp. WP8 Bacillus sp. Pc3 Bacillus sp. BH072 Bacillus sp. OxB-1 Bacillus sp. YP1 Bacillus sp. BS34A Bacillus sp. LM 4-2 Bacillus filamentosus Bacillus smithii Bacillus simplex Bacillus oceanisediminis Bacillus glycinifermentans Bacillus flexus Bacillus gibsonii Bacillus weihaiensis Bacillus xiamenensis Bacillus horikoshii Bacillus krulwichiae Bacillus beveridgei Bacillus kochii Bacillus altitudinis Bacillus sp. SDLI1 Bacillus muralis Bacillus safensis Bacillus intestinalis Bacillus sp. Y1 Bacillus thermoamylovorans Bacillus circulans Oceanobacillus iheyensis Oceanobacillus sp. 160 Oceanobacillus sp. 143 Geobacillus kaustophilus Geobacillus thermoleovorans CCB\_US3\_UF5 Geobacillus thermoleovorans KCTC 3570 Geobacillus thermocatenulatus Geobacillus lituanicus Geobacillus thermodenitrificans Geobacillus sp. WCH70 Geobacillus sp. Y412MC61 Geobacillus sp. Y412MC52 Geobacillus sp. C56-T3 Geobacillus sp. Y4.1MC1 Geobacillus sp. GHH01 Geobacillus genomosp. 3 Geobacillus sp. 12AMOR1 Geobacillus sp. LC300 Geobacillus stearothermophilus Geobacillus subterraneus Geobacillus sp. JS12 Parageobacillus thermoglucosidasius C56-YS93 Parageobacillus thermoglucosidasius DSM 2542 Anoxybacillus flavithermus Anoxybacillus gonensis Anoxybacillus sp. B2M1 Anoxybacillus amylolyticus Anoxybacillus sp. B7M1 Anoxybacillus sp. PDR2 Amphibacillus xylanus Lysinibacillus sphaericus Lysinibacillus varians Lysinibacillus fusiformis Lysinibacillus sp. YS11 Lysinibacillus sp. B2A1 Lysinibacillus sp. 2017 Lysinibacillus sp. SGAir0095 Lysinibacillus pakistanensis Halobacillus halophilus Halobacillus mangrovi Halobacillus litoralis Terribacillus goriensis Virgibacillus sp. SK37 Virgibacillus halodenitrificans Virgibacillus sp. 6R Virgibacillus phasianinus Virgibacillus necropolis Virgibacillus dokdonensis Virgibacillus sp. MSP4-1 Lentibacillus amyloliquefaciens Fictibacillus phosphorivorans Fictibacillus arsenicus Salimicrobium jeotgali Aeribacillus pallidus Aquibacillus sp. TKL69 Psychrobacillus sp. AK 1817 Psychrobacillus sp. PB01 Pradoshia sp. D12 Gracilibacillus sp. SCU50 Salicibibacter kimchii Salicibibacter halophilus Pontibacillus sp. HMF3514 Sporolactobacillus terrae Bacillus selenitireducens Staphylococcus aureus subsp. aureus N315 (MRSA/VSSA) Staphylococcus aureus subsp. aureus Mu50 (MRSA/VISA) Staphylococcus aureus subsp. aureus Mu3 (MRSA/hetero-VISA) Staphylococcus aureus subsp. aureus JH1 (MRSA/VSSA) Staphylococcus aureus subsp. aureus JH9 (MRSA/VISA) Staphylococcus aureus subsp. aureus MW2 (CA-MRSA) Staphylococcus aureus subsp. aureus MSSA476 (MSSA) Staphylococcus aureus subsp. aureus MRSA252 (MRSA) Staphylococcus aureus subsp. aureus COL (MRSA) Staphylococcus aureus subsp. aureus USA300\_TCH1516 (CA-MRSA) Staphylococcus aureus subsp. aureus USA300\_FPR3757 (CA-MRSA) Staphylococcus aureus subsp. aureus NCTC8325 Staphylococcus aureus subsp. aureus Newman Staphylococcus aureus subsp. aureus ED98 Staphylococcus aureus subsp. aureus M013 (CA-MRSA) Staphylococcus aureus subsp. aureus VC40 Staphylococcus aureus subsp. aureus ED133 Staphylococcus aureus subsp. aureus JKD6159 (CA-MRSA) Staphylococcus aureus subsp. aureus JKD6008 (MRSA/VISA) Staphylococcus aureus subsp. aureus ECT-R 2 Staphylococcus aureus subsp. aureus T0131 (MRSA) Staphylococcus aureus subsp. aureus TCH60 Staphylococcus aureus subsp. aureus 11819-97 (CA-MRSA) Staphylococcus aureus subsp. aureus 71193 (MSSA) Staphylococcus aureus subsp. aureus HO 5096 0412 (MRSA) Staphylococcus aureus subsp. aureus TW20 (MRSA) Staphylococcus aureus subsp. aureus ST398 (MRSA) Staphylococcus aureus subsp. aureus LGA251 (MRSA) Staphylococcus aureus subsp. aureus 55/2053 Staphylococcus aureus subsp. aureus 6850 (MSSA) Staphylococcus aureus subsp. aureus CN1 (CA-MRSA) Staphylococcus aureus subsp. aureus SA40 (CA-MRSA) Staphylococcus aureus subsp. aureus SA957 (CA-MRSA) Staphylococcus aureus subsp. aureus SA268 (CA-MRSA) Staphylococcus aureus subsp. aureus Z172 (MRSA/VISA) Staphylococcus aureus subsp. aureus ST228/10388 (MRSA) Staphylococcus aureus subsp. aureus ST228/10497 (MRSA) Staphylococcus aureus subsp. aureus ST228/15532 (MRSA) Staphylococcus aureus subsp. aureus ST228/16035 (MRSA) Staphylococcus aureus subsp. aureus ST228/18412 (MRSA) Staphylococcus aureus subsp. aureus ST228/16125 (MRSA) Staphylococcus aureus subsp. aureus ST228/18341 (MRSA) Staphylococcus aureus subsp. aureus ST228/18583 (MRSA) Staphylococcus aureus subsp. aureus FDAARGOS\_5 Staphylococcus aureus RF122 Staphylococcus aureus 04-02981 (MRSA) Staphylococcus aureus 08BA02176 (LA-MRSA) Staphylococcus aureus M1 (MRSA) Staphylococcus aureus CA-347 (MRSA) Staphylococcus aureus Bmb9393 (MRSA) Staphylococcus aureus USA300-ISMMS1 (MRSA) Staphylococcus aureus 502A Staphylococcus aureus MS4 Staphylococcus argenteus Staphylococcus epidermidis RP62A (MRSE) Staphylococcus epidermidis ATCC 12228 Staphylococcus epidermidis PM221 Staphylococcus epidermidis SEI Staphylococcus haemolyticus JCSC1435 Staphylococcus haemolyticus Sh29/312/L2 Staphylococcus saprophyticus Staphylococcus carnosus Staphylococcus lugdunensis HKU09-01 Staphylococcus lugdunensis N920143 Staphylococcus pseudintermedius HKU10-03 Staphylococcus pseudintermedius ED99 Staphylococcus warneri Staphylococcus pasteuri Staphylococcus xylosus HKUOPL8 Staphylococcus xylosus SMQ-121 Staphylococcus xylosus C2a Staphylococcus hyicus Staphylococcus capitis Staphylococcus schleiferi 1360-13 Staphylococcus schleiferi 2317-03 Staphylococcus agnetis Staphylococcus equorum Staphylococcus simulans Staphylococcus condimenti Staphylococcus pettenkoferi Staphylococcus lutrae Staphylococcus cohnii Staphylococcus sciuri Staphylococcus nepalensis Staphylococcus kloosii Staphylococcus felis Staphylococcus hominis Staphylococcus muscae Staphylococcus caprae Staphylococcus fleurettii Macrococcus caseolyticus Macrococcus canis Macrococcus sp. IME1552 Salinicoccus halodurans Auricoccus indicus Listeria monocytogenes EGD-e (serotype 1/2a) Listeria monocytogenes 08-5578 (serotype 1/2a) Listeria monocytogenes 08-5923 (serotype 1/2a) Listeria monocytogenes 10403S (serotype 1/2a) Listeria monocytogenes SLCC5850 (serotype 1/2a) Listeria monocytogenes La111 (serotype 1/2a) Listeria monocytogenes N53-1 (serotype 1/2a) Listeria monocytogenes EGD (serotype 1/2a) Listeria monocytogenes WSLC1001 (serotype 1/2a) Listeria monocytogenes 6179 (serotype 1/2a) Listeria monocytogenes R479a (serotype 1/2a) Listeria monocytogenes Lm60 (serotype 1/2a) Listeria monocytogenes F2365 (serotype 4b) Listeria monocytogenes CLIP 80459 (serotype 4b) Listeria monocytogenes serotype 4b LL195 Listeria monocytogenes 07PF0776 (serotype 4b) Listeria monocytogenes L312 (serotype 4b) Listeria monocytogenes J1816 (serotype 4b) Listeria monocytogenes J1-220 (serotype 4b) Listeria monocytogenes WSLC1042 (serotype 4b) Listeria monocytogenes HCC23 (serotype 4a) Listeria monocytogenes M7 (serotype 4a) Listeria monocytogenes L99 (serotype 4a) Listeria monocytogenes FSL R2-561 Listeria monocytogenes Finland 1998 Listeria monocytogenes J0161 Listeria monocytogenes SLCC2755 (serotype 1/2b) Listeria monocytogenes SLCC2372 (serotype 1/2c) Listeria monocytogenes serotype 7 SLCC2482 Listeria monocytogenes SLCC2376 (serotype 4c) Listeria monocytogenes SLCC7179 (serotype 3a) Listeria monocytogenes SLCC2378 (serotype 4e) Listeria monocytogenes SLCC2479 (serotype 3c) Listeria monocytogenes SLCC2540 (serotype 3b) Listeria monocytogenes ATCC 19117 (serotype 4d) Listeria monocytogenes NE dc2014 Listeria monocytogenes CFSAN006122 Listeria innocua (serotype 6a) Listeria welshimeri Listeria seeligeri Listeria ivanovii subsp. ivanovii PAM 55 Listeria ivanovii subsp. ivanovii WSLC 3010 Listeria ivanovii WSLC3009 Listeria ivanovii subsp. londoniensis WSLC 30167 Listeria ivanovii subsp. londoniensis WSLC 30151 Listeria weihenstephanensis Listeria grayi Brochothrix thermosphacta Exiguobacterium sibiricum Exiguobacterium sp. AT1b Exiguobacterium antarcticum Exiguobacterium sp. MH3 Exiguobacterium sp. U13-1 Gemella sp. oral taxon 928 Gemella morbillorum Gemella sp. ND 6198 Gemella sanguinis Brevibacillus brevis Brevibacillus laterosporus Brevibacillus formosus Brevibacillus agri Brevibacillus sp. 7WMA2 Paenibacillus sp. JDR-2 Paenibacillus sp. Y412MC10 Paenibacillus polymyxa E681 Paenibacillus polymyxa SC2 Paenibacillus polymyxa M1 Paenibacillus polymyxa CR1 Paenibacillus polymyxa SQR-21 Paenibacillus polymyxa Sb3-1 Paenibacillus mucilaginosus KNP414 Paenibacillus mucilaginosus 3016 Paenibacillus mucilaginosus K02 Paenibacillus terrae Paenibacillus larvae Paenibacillus sabinae Paenibacillus durus Paenibacillus borealis Paenibacillus graminis Paenibacillus odorifer Paenibacillus sp. FSL P4-0081 Paenibacillus sp. FSL R5-0345 Paenibacillus sp. FSL R5-0912 Paenibacillus stellifer Paenibacillus sp. FSL R7-0273 Paenibacillus sp. FSL R7-0331 Paenibacillus sp. FSL H7-0357 Paenibacillus sp. FSL H7-0737 Paenibacillus beijingensis Paenibacillus sp. IHBB 10380 Paenibacillus riograndensis Paenibacillus peoriae Paenibacillus naphthalenovorans Paenibacillus sp. 32O-W Paenibacillus bovis Paenibacillus xylanexedens Paenibacillus yonginensis Paenibacillus swuensis Paenibacillus donghaensis Paenibacillus ihbetae Paenibacillus crassostreae Paenibacillus kribbensis Paenibacillus sp. IHB B 3084 Paenibacillus vortex Paenibacillus lautus Paenibacillus lentus Paenibacillus psychroresistens Thermobacillus composti Aneurinibacillus sp. XH2 Aneurinibacillus soli Cohnella candidum Cohnella sp. HS21 Saccharibacillus sp. ATSA2 Alicyclobacillus acidocaldarius subsp. acidocaldarius DSM 446 Alicyclobacillus acidocaldarius subsp. acidocaldarius Tc-4-1 Kyrpidia tusciae Kyrpidia spormannii Tumebacillus avium Tumebacillus algifaecis Solibacillus silvestris StLB046 Solibacillus silvestris DSM 12223 Solibacillus sp. R5-41 Planococcus sp. PAMC 21323 Planococcus kocurii Planococcus rifietoensis Planococcus versutus Planococcus antarcticus Planococcus donghaensis Planococcus halocryophilus Planococcus maritimus Planococcus plakortidis Planococcus faecalis Planococcus sp. MB-3u-03 Jeotgalibacillus malaysiensis Kurthia sp. 11kri321 Kurthia zopfii Sporosarcina psychrophila Sporosarcina sp. P33 Sporosarcina sp. P37 Sporosarcina ureae Sporosarcina sp. PTS2304 Sporosarcina pasteurii Rummeliibacillus stabekisii Paenisporosarcina sp. K2R23-3 Paenisporosarcina antarctica Planomicrobium glaciei Planomicrobium sp. Y50 Novibacillus thermophilus Laceyella sacchari Thermoactinomyces vulgaris Lactococcus lactis subsp. lactis Il1403 Lactococcus lactis subsp. lactis KF147 Lactococcus lactis subsp. lactis CV56 Lactococcus lactis subsp. lactis IO-1 Lactococcus lactis subsp. lactis KLDS 4.0325 Lactococcus lactis subsp. lactis NCDO 2118 Lactococcus lactis subsp. cremoris MG1363 Lactococcus lactis subsp. cremoris SK11 Lactococcus lactis subsp. cremoris A76 Lactococcus lactis subsp. cremoris NZ9000 Lactococcus lactis subsp. cremoris UC509.9 Lactococcus lactis subsp. cremoris KW2 Lactococcus lactis AI06 Lactococcus garvieae ATCC 49156 Lactococcus garvieae Lg2 Lactococcus piscium Lactococcus raffinolactis Lactococcus sp. 1JSPR-7 Lactococcus sp. KACC 19320 Streptococcus pyogenes M1 GAS (serotype M1) Streptococcus pyogenes MGAS5005 (serotype M1) Streptococcus pyogenes M1 476 (serotype M1) Streptococcus pyogenes A20 (serotype M1) Streptococcus pyogenes MGAS8232 (serotype M18) Streptococcus pyogenes MGAS315 (serotype M3) Streptococcus pyogenes SSI-1 (serotype M3) Streptococcus pyogenes MGAS10270 (serotype M2) Streptococcus pyogenes MGAS10750 (serotype M4) Streptococcus pyogenes MGAS2096 (serotype M12) Streptococcus pyogenes MGAS9429 (serotype M12) Streptococcus pyogenes Manfredo (serotype M5) Streptococcus pyogenes MGAS10394 (serotype M6) Streptococcus pyogenes MGAS6180 (serotype M28) Streptococcus pyogenes MGAS15252 (serotype M59) Streptococcus pyogenes MGAS1882 (serotype M59) Streptococcus pyogenes NZ131 (serotype M49) Streptococcus pyogenes Alab49 (serotype M53) Streptococcus pyogenes HSC5 (serotype M14) Streptococcus pyogenes STAB901 (serotype M44) Streptococcus pneumoniae TIGR4 (virulent serotype 4) Streptococcus pneumoniae D39 (virulent serotype 2) Streptococcus pneumoniae R6 (avirulent serotype 2) Streptococcus pneumoniae CGSP14 (serotype 14) Streptococcus pneumoniae JJA (serotype 14) Streptococcus pneumoniae INV200 (serotype 14) Streptococcus pneumoniae G54 (serotype 19F) Streptococcus pneumoniae Taiwan19F-14 (serotype 19F) Streptococcus pneumoniae ST556 (serotype 19F) Streptococcus pneumoniae A026 (serotype 19F) Streptococcus pneumoniae ATCC 700669 (serotype 23F ST81 lineage) Streptococcus pneumoniae Hungary19A 6 (serotype 19A) Streptococcus pneumoniae TCH8431/19A (serotype 19A) Streptococcus pneumoniae 70585 (serotype 5) Streptococcus pneumoniae P1031 (serotype 1) Streptococcus pneumoniae INV104 (serotype 1) Streptococcus pneumoniae gamPNI0373 (virulent serotype 1) Streptococcus pneumoniae 670-6B (serotype 6B) Streptococcus pneumoniae AP200 (serotype 11A) Streptococcus pneumoniae OXC141 (serotype 3) Streptococcus pneumoniae SPNA45 (serotype 3) Streptococcus pneumoniae SPN034156 (serotype 3) Streptococcus pneumoniae SPN034183 (serotype 3) Streptococcus pneumoniae SPN994038 (serotype 3) Streptococcus pneumoniae SPN994039 (serotype 3) Streptococcus agalactiae 2603 (serotype V) Streptococcus agalactiae NEM316 (serotype III) Streptococcus agalactiae A909 (serotype Ia) Streptococcus agalactiae GD201008-001 (serotype Ia) Streptococcus agalactiae SA20 Streptococcus agalactiae 2-22 (serotype Ib) Streptococcus agalactiae 09mas018883 Streptococcus agalactiae ILRI005 Streptococcus agalactiae ILRI112 Streptococcus agalactiae 138P Streptococcus agalactiae 138spar Streptococcus agalactiae COH1 Streptococcus agalactiae NGBS061 Streptococcus agalactiae NGBS572 Streptococcus agalactiae CNCTC 10/84 Streptococcus mutans UA159 (serotype c) Streptococcus mutans NN2025 (serotype c) Streptococcus mutans GS-5 (serotype c) Streptococcus mutans LJ23 (serotype k) Streptococcus mutans UA159-FR Streptococcus thermophilus CNRZ1066 Streptococcus thermophilus LMG 18311 Streptococcus thermophilus LMD-9 Streptococcus thermophilus ND03 Streptococcus thermophilus JIM 8232 Streptococcus thermophilus MN-ZLW-002 Streptococcus thermophilus ASCC 1275 Streptococcus thermophilus S9 Streptococcus sanguinis Streptococcus suis BM407 (serotype 2) Streptococcus suis 05ZYH33 (serotype 2) Streptococcus suis 98HAH33 (serotype 2) Streptococcus suis P1/7 (serotype 2) Streptococcus suis SC84 (serotype 2) Streptococcus suis A7 (serotype 2) Streptococcus suis GZ1 (serotype 2) Streptococcus suis S735 (serotype 2) Streptococcus suis SC070731 (serotype 2) Streptococcus suis ST3 (serotype 3) Streptococcus suis YB51 (serotype 3) Streptococcus suis D12 (serotype 9) Streptococcus suis D9 (serotype 7) Streptococcus suis JS14 (serotype 14) Streptococcus suis SS12 (serotype 1/2) Streptococcus suis ST1 (serotype 1) Streptococcus suis TL13 (serotype 16) Streptococcus suis T15 Streptococcus gordonii Streptococcus equi subsp. zooepidemicus MGCS10565 Streptococcus equi subsp. zooepidemicus H70 Streptococcus equi subsp. zooepidemicus ATCC 35246 Streptococcus equi subsp. zooepidemicus CY Streptococcus equi subsp. equi 4047 Streptococcus uberis Streptococcus dysgalactiae subsp. equisimilis GGS\_124 Streptococcus dysgalactiae subsp. equisimilis ATCC 12394 Streptococcus dysgalactiae subsp. equisimilis RE378 Streptococcus dysgalactiae subsp. equisimilis AC-2713 Streptococcus dysgalactiae subsp. equisimilis 167 Streptococcus gallolyticus UCN34 Streptococcus gallolyticus subsp. gallolyticus ATCC BAA-2069 Streptococcus gallolyticus subsp. gallolyticus ATCC 43143 Streptococcus mitis Streptococcus oralis Streptococcus parauberis Streptococcus pasteurianus Streptococcus parasanguinis ATCC 15912 Streptococcus parasanguinis FW213 Streptococcus salivarius CCHSS3 Streptococcus salivarius 57.I Streptococcus salivarius JIM8777 Streptococcus salivarius NCTC 8618 Streptococcus salivarius HSISS4 Streptococcus pseudopneumoniae Streptococcus macedonicus Streptococcus infantarius Streptococcus intermedius JTH08 Streptococcus intermedius B196 Streptococcus intermedius C270 Streptococcus anginosus C1051 Streptococcus anginosus C238 Streptococcus anginosus SA1 Streptococcus constellatus subsp. pharyngis C1050 Streptococcus constellatus subsp. pharyngis C232 Streptococcus constellatus subsp. pharyngis C818 Streptococcus cristatus Streptococcus iniae SF1 Streptococcus iniae ISET0901 Streptococcus iniae ISNO Streptococcus iniae SFST01-82 Streptococcus lutetiensis Streptococcus sp. I-G2 Streptococcus sp. I-P16 Streptococcus sp. VT 162 Streptococcus pantholopis Streptococcus sp. A12 Streptococcus sp. NPS 308 Streptococcus sobrinus Streptococcus ruminantium Streptococcus equinus Streptococcus koreensis Lactobacillus plantarum WCFS1 Lactobacillus plantarum JDM1 Lactobacillus plantarum ZJ316 Lactobacillus plantarum subsp. plantarum ST-III Lactobacillus plantarum subsp. plantarum P-8 Lactobacillus plantarum 16 Lactobacillus plantarum B21 Lactobacillus johnsonii NCC 533 Lactobacillus johnsonii FI9785 Lactobacillus johnsonii DPC 6026 Lactobacillus johnsonii N6.2 Lactobacillus acidophilus NCFM Lactobacillus acidophilus 30SC Lactobacillus acidophilus La-14 Lactobacillus acidophilus FSI4 Lactobacillus sakei Lactobacillus salivarius UCC118 Lactobacillus salivarius CECT 5713 Lactobacillus salivarius JCM1046 Lactobacillus delbrueckii subsp. bulgaricus ATCC 11842 Lactobacillus delbrueckii subsp. bulgaricus ATCC BAA-365 Lactobacillus delbrueckii subsp. bulgaricus ND02 Lactobacillus delbrueckii subsp. bulgaricus 2038 Lactobacillus brevis ATCC 367 Lactobacillus brevis KB290 Lactobacillus paracasei ATCC 334 Lactobacillus paracasei Zhang Lactobacillus paracasei N1115 Lactobacillus paracasei subsp. paracasei 8700:2 Lactobacillus paracasei subsp. paracasei JCM 8130 Lactobacillus casei BL23 Lactobacillus casei BD-II Lactobacillus casei LC2W Lactobacillus casei W56 Lactobacillus casei LOCK919 Lactobacillus casei 12A Lactobacillus gasseri ATCC 33323 Lactobacillus reuteri DSM 20016 Lactobacillus reuteri JCM 1112 Lactobacillus reuteri SD2112 Lactobacillus reuteri I5007 Lactobacillus reuteri TD1 Lactobacillus helveticus DPC 4571 Lactobacillus helveticus H10 Lactobacillus helveticus R0052 Lactobacillus helveticus CNRZ32 Lactobacillus helveticus H9 Lactobacillus helveticus KLDS1.8701 Lactobacillus fermentum IFO 3956 Lactobacillus fermentum CECT 5716 Lactobacillus fermentum F-6 Lactobacillus rhamnosus GG Lactobacillus rhamnosus GG Lactobacillus rhamnosus Lc 705 Lactobacillus rhamnosus ATCC 8530 Lactobacillus rhamnosus LOCK900 Lactobacillus rhamnosus LOCK908 Lactobacillus crispatus Lactobacillus amylovorus GRL 1112 Lactobacillus amylovorus GRL1118 Lactobacillus buchneri NRRL B-30929 Lactobacillus buchneri CD034 Lactobacillus kefiranofaciens Lactobacillus ruminis Lactobacillus sanfranciscensis Lactobacillus sp. wkB8 Lactobacillus hokkaidonensis Lactobacillus mucosae Lactobacillus acetotolerans Lactobacillus ginsenosidimutans Lactobacillus koreensis Lactobacillus heilongjiangensis Lactobacillus kunkeei Lactobacillus gallinarum Lactobacillus paraplantarum Lactobacillus oris Lactobacillus parabuchneri Lactobacillus lindneri Lactobacillus paracollinoides Lactobacillus jensenii Lactobacillus curieae Lactobacillus crustorum Lactobacillus backii Lactobacillus curvatus Lactobacillus amylophilus Lactobacillus amylolyticus Lactobacillus agilis Lactobacillus zymae Lactobacillus pentosus Lactobacillus coryniformis Lactobacillus acidipiscis Lactobacillus allii Lactobacillus alimentarius Lactobacillus paragasseri Lactobacillus farciminis Lactobacillus nagelii Lactobacillus dextrinicus Lactobacillus kullabergensis Pediococcus pentosaceus ATCC 25745 Pediococcus pentosaceus SL4 Pediococcus claussenii Pediococcus damnosus Pediococcus acidilactici Pediococcus inopinatus Enterococcus faecalis V583 Enterococcus faecalis 62 Enterococcus faecalis OG1RF Enterococcus faecalis D32 Enterococcus faecalis Symbioflor 1 Enterococcus faecalis DENG1 Enterococcus faecalis ATCC 29212 Enterococcus faecalis 7L76 Enterococcus faecium Aus0004 Enterococcus faecium Aus0085 Enterococcus faecium DO Enterococcus faecium ATCC 8459 = NRRL B-2354 Enterococcus faecium T110 Enterococcus hirae Enterococcus casseliflavus Enterococcus mundtii Enterococcus durans Enterococcus gallinarum Enterococcus silesiacus Enterococcus thailandicus Enterococcus gilvus Enterococcus avium Melissococcus plutonius ATCC 35311 Melissococcus plutonius DAT561 Tetragenococcus halophilus NBRC 12172 Tetragenococcus halophilus YJ1 Tetragenococcus osmophilus Tetragenococcus koreensis Vagococcus teuberi Vagococcus penaei Vagococcus sp. CF-49 Vagococcus sp. MN-17 Oenococcus oeni Oenococcus sp. UCMA 16435 Oenococcus sicerae Leuconostoc mesenteroides subsp. mesenteroides ATCC 8293 Leuconostoc mesenteroides subsp. mesenteroides J18 Leuconostoc mesenteroides KFRI-MG Leuconostoc citreum Leuconostoc kimchii Leuconostoc sp. C2 Leuconostoc carnosum Leuconostoc gelidum subsp. gasicomitatum Leuconostoc gelidum JB7 Leuconostoc lactis Leuconostoc garlicum Leuconostoc mesenteroides subsp. suionicum Leuconostoc pseudomesenteroides Weissella koreensis Weissella ceti WS08 Weissella ceti WS74 Weissella ceti WS105 Weissella cibaria Weissella jogaejeotgali Weissella paramesenteroides Weissella confusa Weissella soli Weissella hellenica Weissella sp. 26KH-42 Aerococcus urinae ACS-120-V-Col10a Aerococcus urinae CCUG36881 Aerococcus urinaeequi Aerococcus sanguinicola Aerococcus christensenii Aerococcus viridans Aerococcus urinaehominis Aerococcaceae bacterium ZY16052 Carnobacterium sp. 17-4 Carnobacterium maltaromaticum Carnobacterium inhibens Carnobacterium sp. CP1 Carnobacterium divergens Carnobacterium sp. PL17GRE32 Marinilactibacillus sp. 15R Jeotgalibaca sp. PTS2502 Jeotgalibaca dankookensis Jeotgalibaca sp. H21T32 Jeotgalibaca arthritidis Dolosigranulum pigrum Clostridium acetobutylicum ATCC 824 Clostridium acetobutylicum DSM 1731 Clostridium acetobutylicum EA 2018 Clostridium perfringens 13 Clostridium perfringens ATCC 13124 Clostridium perfringens SM101 Clostridium tetani E88 Clostridium tetani 12124569 Clostridium novyi Clostridium botulinum A ATCC 3502 Clostridium botulinum A ATCC 19397 Clostridium botulinum A Hall Clostridium botulinum A2 Clostridium botulinum A3 Loch Maree Clostridium botulinum B Eklund 17B (NRP) Clostridium botulinum B1 Okra Clostridium botulinum Ba4 Clostridium botulinum BKT015925 Clostridium botulinum E3 Clostridium botulinum F Langeland Clostridium botulinum F 230613 Clostridium botulinum H04402 065 Clostridium beijerinckii NCIMB 8052 Clostridium beijerinckii ATCC 35702 Clostridium beijerinckii NCIMB 14988 Clostridium kluyveri DSM 555 Clostridium kluyveri NBRC 12016 Clostridium ljungdahlii Clostridium cellulovorans Clostridium sp. SY8519 Clostridium sp. BNL1100 Clostridium saccharoperbutylacetonicum Clostridium pasteurianum BC1 Clostridium pasteurianum DSM 525 = ATCC 6013 Clostridium pasteurianum DSM 525 = ATCC 6013 Clostridium saccharobutylicum Clostridium autoethanogenum Clostridium bornimense Clostridium baratii Clostridium scatologenes Clostridium sporogenes Clostridium aceticum Clostridium carboxidivorans Clostridium butyricum Clostridium tyrobutyricum Clostridium estertheticum Clostridium taeniosporum Clostridium formicaceticum Clostridium chauvoei Clostridium argentinense Clostridium drakei Clostridium isatidis Clostridium septicum Clostridium diolis Alkaliphilus metalliredigens Alkaliphilus oremlandii Candidatus Arthromitus sp. SFB-mouse-Japan Candidatus Arthromitus sp. SFB-mouse-Yit Candidatus Arthromitus sp. SFB-mouse-NL Candidatus Arthromitus sp. SFB-rat-Yit Geosporobacter ferrireducens Hathewaya histolytica Caloramator sp. E03 Crassaminicella sp. SY095 Caloranaerobacter azorensis Mageeibacillus indolicus Fastidiosipila sanguinis Hungateiclostridium thermocellum ATCC 27405 Hungateiclostridium thermocellum DSM 1313 Hungateiclostridium clariflavum Hungateiclostridium saccincola Hungateiclostridiaceae bacterium KB18 Ruminiclostridium cellulolyticum Thermoclostridium stercorarium subsp. stercorarium DSM 8532 Thermoclostridium stercorarium subsp. stercorarium DSM 8532 Pseudoclostridium thermosuccinogenes Eubacterium siraeum V10Sc8a Eubacterium siraeum 70/3 Clostridium cellulosi Ruminococcaceae bacterium CPB6 Flavonifractor plautii Ethanoligenens harbinense Ruminococcus albus Ruminococcus champanellensis Ruminococcus sp. SR1/5 Ruminococcus bicirculans Ruminococcus sp. JE7A12 Faecalibacterium prausnitzii L2-6 Faecalibacterium prausnitzii SL3/3 Caproiciproducens sp. NJN-50 Butyrivibrio proteoclasticus Butyrivibrio fibrisolvens Butyrivibrio hungatei Cellulosilyticum lentocellum Cellulosilyticum sp. WCF-2 Roseburia hominis Roseburia intestinalis XB6B4 Roseburia intestinalis M50/1 Coprococcus sp. ART55/1 Coprococcus catus Blautia obeum Blautia sp. YL58 Ruminococcus torques Blautia hansenii Blautia sp. N6H1-15 Blautia producta Blautia sp. SC05B48 Lachnoclostridium phytofermentans Lachnoclostridium sp. YL32 Clostridium saccharolyticum WM1 Clostridium cf. saccharolyticum K10 Clostridium bolteae Anaerostipes hadrus Anaerostipes rhamnosivorans Herbinix luporum Anaerotignum propionicum Lachnoanaerobaculum umeaense Anaerobutyricum hallii Pseudobutyrivibrio xylanivorans Anaerocolumna sp. CBA3638 Eubacterium eligens Eubacterium rectale ATCC 33656 Eubacterium rectale DSM 17629 Eubacterium rectale M104/1 Lachnospiraceae bacterium oral taxon 500 Clostridioides difficile 630 Clostridioides difficile 630 Clostridioides difficile CD196 Clostridioides difficile R20291 Clostridioides difficile 630Derm Peptoclostridium acidaminophilum Acetoanaerobium sticklandii Filifactor alocis Paeniclostridium sordellii Peptostreptococcaceae bacterium oral taxon 929 Symbiobacterium thermophilum Syntrophomonas wolfei Syntrophothermus lipocalidus Candidatus Syntrophocurvum alkaliphilum Desulfitobacterium hafniense Y51 Desulfitobacterium hafniense DCB-2 Desulfitobacterium dehalogenans Desulfitobacterium dichloroeliminans Desulfitobacterium metallireducens Desulfotomaculum reducens Desulfotomaculum nigrificans Desulfotomaculum ruminis Desulfotomaculum ferrireducens Desulfofarcimen acetoxidans Desulfofundulus kuznetsovii Desulfallas gibsoniae Pelotomaculum thermopropionicum Candidatus Desulforudis audaxviator Thermincola potens Syntrophobotulus glycolicus Desulfosporosinus orientis Desulfosporosinus acidiphilus Desulfosporosinus meridiei Dehalobacter sp. DCA Dehalobacter sp. CF Dehalobacter restrictus Heliobacterium modesticaldum Heliorestis convoluta Eubacterium limosum Eubacterium maltosivorans Acetobacterium woodii Oscillibacter valericigenes Oscillospiraceae bacterium J115 Thermaerobacter marianensis Thermaerobacter sp. FW80 Thermaerobacter sp. PB12/4term Sulfobacillus acidophilus TPY Sulfobacillus acidophilus DSM 10332 Sulfobacillus thermotolerans Carboxydocella thermautotrophica Christensenella minuta Intestinimonas butyriciproducens Mogibacterium diversum Aminipila sp. JN-39 Aminipila sp. CBA3637 Aminipila butyrica Eubacterium sulci Butyrate-producing bacterium SM4/1 Butyrate-producing bacterium SS3/4 Clostridiales bacterium 70B-A Caldanaerobacter subterraneus subsp. tengcongensis Thermoanaerobacter sp. X514 Thermoanaerobacter sp. X513 Thermoanaerobacter pseudethanolicus Thermoanaerobacter italicus Thermoanaerobacter mathranii Thermoanaerobacter brockii Thermoanaerobacter wiegelii Thermoanaerobacter kivui Carboxydothermus hydrogenoformans Tepidanaerobacter acetatoxydans Re1 Tepidanaerobacter acetatoxydans Re1 Moorella thermoacetica ATCC 39073 Moorella thermoacetica DSM 521 Moorella thermoacetica DSM 2955 Ammonifex degensii Thermacetogenium phaeum Caldicellulosiruptor saccharolyticus Caldicellulosiruptor bescii Caldicellulosiruptor obsidiansis Caldicellulosiruptor hydrothermalis Caldicellulosiruptor owensensis Caldicellulosiruptor kristjanssonii Caldicellulosiruptor kronotskyensis Caldicellulosiruptor lactoaceticus Caldicellulosiruptor changbaiensis Thermosediminibacter oceani Thermoanaerobacterium thermosaccharolyticum DSM 571 Thermoanaerobacterium thermosaccharolyticum M0795 Thermoanaerobacterium xylanolyticum Thermoanaerobacterium saccharolyticum Thermodesulfobium narugense Thermodesulfobium acidiphilum Mahella australiensis Natranaerobius thermophilus Halothermothrix orenii Halanaerobium hydrogeniformans Halanaerobium praevalens Halocella sp. SP3-1 Acetohalobium arabaticum Halobacteroides halobius Anoxybacter fermentans Finegoldia magna Anaerococcus prevotii Parvimonas micra Peptoniphilus sp. ING2-D1G Peptoniphilus harei Gottschalkia acidurici Sporanaerobacter sp. NJN-17 Veillonella parvula Veillonella atypica Veillonella rodentium Veillonella dispar Megasphaera elsdenii Megasphaera hexanoica Megasphaera sp. AJH120 Dialister pneumosintes Dialister hominis Selenomonas sputigena Selenomonas ruminantium Selenomonas sp. oral taxon 478 Selenomonas sp. oral taxon 136 Selenomonas sp. oral taxon 920 Megamonas hypermegale Megamonas funiformis Pelosinus sp. UFO1 Pelosinus fermentans Methylomusa anaerophila Sporomusa termitida Acidaminococcus fermentans Acidaminococcus intestini Phascolarctobacterium faecium Erysipelothrix rhusiopathiae Fujisawa Erysipelothrix rhusiopathiae SY1027 Erysipelothrix larvae Erysipelothrix sp. 15TAL0474 Faecalitalea cylindroides Turicibacter sp. H121 Faecalibaculum rodentium Absiella argi Absiella sp. 9CBEGH2 Erysipelotrichaceae bacterium I46 Erysipelotrichaceae bacterium SG0102 Limnochorda pilosa Coprothermobacter proteolyticus Mycoplasma genitalium G37 Mycoplasma genitalium M2288 Mycoplasma genitalium M2321 Mycoplasma genitalium M6282 Mycoplasma genitalium M6320 Mycoplasma pneumoniae M129 Mycoplasma pneumoniae 309 Mycoplasma pneumoniae FH Mycoplasma pneumoniae M129-B7 Mycoplasma pulmonis Mycoplasma penetrans Mycoplasma gallisepticum R(low) Mycoplasma gallisepticum R(high) Mycoplasma gallisepticum F Mycoplasma gallisepticum NC06\_2006.080-5-2P Mycoplasma gallisepticum NC95\_13295-2-2P Mycoplasma gallisepticum NY01\_2001.047-5-1P Mycoplasma gallisepticum VA94\_7994-1-7P Mycoplasma gallisepticum WI01\_2001.043-13-2P Mycoplasma gallisepticum CA06\_2006.052-5-2P Mycoplasma gallisepticum NC08\_2008.031-4-3P Mycoplasma gallisepticum NC96\_1596-4-2P Mycoplasma gallisepticum S6 Mycoplasma mycoides subsp. mycoides SC PG1 Mycoplasma mycoides subsp. mycoides SC Gladysdale Mycoplasma mycoides subsp. mycoides izsam\_mm5713 Mycoplasma mycoides subsp. capri LC 95010 Mycoplasma capricolum subsp. capricolum ATCC 27343 Mycoplasma capricolum subsp. capripneumoniae 9231-Abomsa Mycoplasma capricolum subsp. capripneumoniae F38 Mycoplasma capricolum subsp. capripneumoniae ILRI181 Mycoplasma capricolum subsp. capripneumoniae 87001 Mycoplasma leachii PG50 Mycoplasma leachii 99/014/6 Mycoplasma mobile Mycoplasma hyopneumoniae 232 Mycoplasma hyopneumoniae J Mycoplasma hyopneumoniae 7448 Mycoplasma hyopneumoniae 168 Mycoplasma hyopneumoniae 168-L Mycoplasma hyopneumoniae 7422 Mycoplasma synoviae 53 Mycoplasma synoviae ATCC 25204 Mycoplasma agalactiae PG2 Mycoplasma agalactiae 5632 Mycoplasma arthritidis Mycoplasma conjunctivae Mycoplasma crocodyli Mycoplasma hyorhinis HUB-1 Mycoplasma hyorhinis GDL-1 Mycoplasma hyorhinis MCLD Mycoplasma hyorhinis SK76 Mycoplasma hyorhinis DBS 1050 Mycoplasma fermentans JER Mycoplasma fermentans M64 Mycoplasma fermentans PG18 Mycoplasma bovis PG45 Mycoplasma bovis Hubei-1 Mycoplasma bovis HB0801 Mycoplasma bovis CQ-W70 Mycoplasma haemofelis Langford 1 Mycoplasma haemofelis Ohio2 Mycoplasma suis Illinois Mycoplasma suis KI3806 Mycoplasma putrefaciens KS1 Mycoplasma putrefaciens Mput9231 Mycoplasma haemocanis Mycoplasma wenyonii Candidatus Mycoplasma haemolamae Mycoplasma cynos Candidatus Mycoplasma haemominutum Mycoplasma parvum Mycoplasma ovis Mycoplasma bovoculi Mycoplasma californicum ST-6 Mycoplasma californicum HAZ160\_1 Candidatus Mycoplasma girerdii Mycoplasma flocculare Mycoplasma yeatsii Mycoplasma dispar Mycoplasma gallinaceum Mycoplasma canis LV Mycoplasma canis PG 14 Mycoplasma arginini Mycoplasma sp. (ex Biomphalaria glabrata) Mycoplasma pullorum Mycoplasma bovigenitalium Mycoplasma phocidae Mycoplasma hyosynoviae Mycoplasma bovirhinis Mycoplasma cloacale Mycoplasma amphoriforme Candidatus Hepatoplasma crinochetorum Acholeplasma laidlawii Acholeplasma brassicae Acholeplasma palmae Acholeplasma oculi Acholeplasma axanthum Mesoplasma florum L1 Mesoplasma florum W37 Mesoplasma chauliocola Mesoplasma lactucae Mesoplasma entomophilum Mesoplasma syrphidae Mesoplasma tabanidae Mesoplasma coleopterae Entomoplasma luminosum Entomoplasma somnilux Entomoplasma freundtii Entomoplasma melaleucae Spiroplasma chrysopicola Spiroplasma syrphidicola Spiroplasma diminutum Spiroplasma taiwanense Spiroplasma apis Spiroplasma mirum ATCC 29335 Spiroplasma mirum ATCC 29335 SMCA Spiroplasma culicicola Spiroplasma sabaudiense Spiroplasma atrichopogonis Spiroplasma eriocheiris Spiroplasma turonicum Spiroplasma litorale Spiroplasma kunkelii Spiroplasma cantharicola Spiroplasma helicoides Spiroplasma citri Spiroplasma floricola Spiroplasma corruscae Spiroplasma clarkii Spiroplasma sp. NBRC 100390 Spiroplasma sp. TU-14 Spiroplasma tabanidicola Spiroplasma phoeniceum Candidatus Izimaplasma sp. HR1 Tenericutes bacterium MO-XQ Tenericutes bacterium MZ-XQ Mycobacterium tuberculosis H37Rv Mycobacterium tuberculosis H37Rv Mycobacterium tuberculosis CDC1551 Mycobacterium tuberculosis H37Ra Mycobacterium tuberculosis F11 Mycobacterium tuberculosis KZN 1435 Mycobacterium tuberculosis KZN 4207 Mycobacterium tuberculosis KZN 605 Mycobacterium tuberculosis RGTB327 Mycobacterium tuberculosis RGTB423 Mycobacterium tuberculosis CCDC5079 Mycobacterium tuberculosis CCDC5079 Mycobacterium tuberculosis CCDC5180 Mycobacterium tuberculosis CTRI-2 Mycobacterium tuberculosis UT205 Mycobacterium tuberculosis Erdman = ATCC 35801 Mycobacterium tuberculosis Beijing/NITR203 Mycobacterium tuberculosis 7199-99 Mycobacterium tuberculosis CAS/NITR204 Mycobacterium tuberculosis EAI5/NITR206 Mycobacterium tuberculosis EAI5 Mycobacterium tuberculosis Haarlem/NITR202 Mycobacterium tuberculosis Haarlem Mycobacterium tuberculosis BT1 Mycobacterium tuberculosis BT2 Mycobacterium tuberculosis HKBS1 Mycobacterium tuberculosis variant bovis AF2122/97 Mycobacterium tuberculosis variant bovis BCG Pasteur 1173P2 Mycobacterium tuberculosis variant bovis BCG Tokyo 172 Mycobacterium tuberculosis variant bovis BCG Mexico Mycobacterium tuberculosis variant bovis BCG Korea 1168P Mycobacterium tuberculosis variant bovis BCG ATCC 35743 Mycobacterium tuberculosis variant africanum Mycobacterium tuberculosis variant microti Mycobacterium canettii CIPT 140010059 Mycobacterium canettii CIPT 140060008 Mycobacterium canettii CIPT 140070008 Mycobacterium canettii CIPT 140070010 Mycobacterium canettii CIPT 140070017 Mycobacterium leprae TN Mycobacterium leprae Br4923 Mycobacterium avium subsp. paratuberculosis K-10 Mycobacterium avium subsp. paratuberculosis MAP4 Mycobacterium avium subsp. paratuberculosis E1 Mycobacterium avium subsp. paratuberculosis E93 Mycobacterium avium 104 Mycobacterium intracellulare MOTT-02 Mycobacterium intracellulare ATCC 13950 Mycobacterium intracellulare subsp. intracellulare Mycobacterium intracellulare subsp. yongonense Mycobacterium paraintracellulare Mycobacterium chimaera Mycobacterium marseillense Mycobacterium lepraemurium Mycobacterium sp. JS623 Mycobacterium ulcerans Mycobacterium sp. MCS Mycobacterium sp. KMS Mycobacterium sp. JLS Mycobacterium marinum M Mycobacterium marinum E11 Mycobacterium sp. MOTT36Y Mycobacterium liflandii Mycobacterium kansasii ATCC 12478 Mycobacterium sp. VKM Ac-1817D Mycobacterium sp. EPa45 Mycobacterium haemophilum Mycobacterium sp. NRRL B-3805 Mycobacterium dioxanotrophicus Mycobacterium shigaense Mycolicibacterium smegmatis MC2 155 Mycolicibacterium smegmatis MC2 155 Mycolicibacterium smegmatis MC2 155 Mycolicibacterium smegmatis INHR1 Mycolicibacterium smegmatis INHR2 Mycolicibacterium vanbaalenii Mycolicibacterium gilvum PYR-GCK Mycolicibacterium gilvum Spyr1 Mycolicibacterium chubuense Mycolicibacterium neoaurum Mycolicibacterium goodii Mycolicibacterium fortuitum Mycolicibacterium phlei Mycolicibacterium vaccae Mycolicibacterium litorale Mycolicibacterium rhodesiae Mycolicibacterium thermoresistibile Mycolicibacterium hassiacum Mycobacteroides abscessus ATCC 19977 Mycobacteroides abscessus subsp. massiliense GO 06 Mycobacteroides abscessus subsp. bolletii 50594 Mycobacteroides abscessus subsp. massiliense CCUG 48898 = JCM 15300 Mycobacteroides chelonae Mycobacteroides immunogenum Mycobacterium stephanolepidis Mycobacteroides saopaulense Mycobacteroides salmoniphilum Mycolicibacter sinensis Mycolicibacter terrae Hoyosella subflava Mycolicibacillus koreensis Corynebacterium glutamicum ATCC 13032 (Kyowa Hakko) Corynebacterium glutamicum ATCC 13032 (Bielefeld) Corynebacterium glutamicum K051 Corynebacterium glutamicum R Corynebacterium glutamicum SCgG1 Corynebacterium glutamicum SCgG2 Corynebacterium glutamicum MB001 Corynebacterium glutamicum ATCC 21831 Corynebacterium glutamicum AR1 Corynebacterium glutamicum B253 Corynebacterium efficiens Corynebacterium diphtheriae NCTC 13129 Corynebacterium diphtheriae 241 Corynebacterium diphtheriae INCA 402 Corynebacterium diphtheriae HC01 Corynebacterium diphtheriae HC02 Corynebacterium diphtheriae HC03 Corynebacterium diphtheriae HC04 Corynebacterium diphtheriae 31A Corynebacterium diphtheriae BH8 Corynebacterium diphtheriae C7 (beta) Corynebacterium diphtheriae CDCE 8392 Corynebacterium diphtheriae PW8 Corynebacterium diphtheriae VA01 Corynebacterium diphtheriae NCTC11397 Corynebacterium jeikeium Corynebacterium urealyticum DSM 7109 Corynebacterium urealyticum DSM 7111 Corynebacterium aurimucosum Corynebacterium kroppenstedtii Corynebacterium pseudotuberculosis 3/99-5 Corynebacterium pseudotuberculosis 316 Corynebacterium pseudotuberculosis P54B96 Corynebacterium pseudotuberculosis 1002 Corynebacterium pseudotuberculosis C231 Corynebacterium pseudotuberculosis I19 Corynebacterium pseudotuberculosis PAT10 Corynebacterium pseudotuberculosis 267 Corynebacterium pseudotuberculosis 31 Corynebacterium pseudotuberculosis 1/06-A Corynebacterium pseudotuberculosis 42/02-A Corynebacterium pseudotuberculosis CIP 52.97 Corynebacterium pseudotuberculosis 258 Corynebacterium pseudotuberculosis Cp162 Corynebacterium pseudotuberculosis 48252 Corynebacterium pseudotuberculosis CS\_10 Corynebacterium pseudotuberculosis Ft\_2193/67 Corynebacterium resistens Corynebacterium ulcerans BR-AD22 Corynebacterium ulcerans 809 Corynebacterium ulcerans 0102 Corynebacterium ulcerans 210932 Corynebacterium ulcerans FRC11 Corynebacterium ulcerans 210931 Corynebacterium ulcerans 05146 Corynebacterium ulcerans 131002 Corynebacterium variabile Corynebacterium halotolerans Corynebacterium callunae Corynebacterium terpenotabidum Corynebacterium maris Corynebacterium argentoratense Corynebacterium falsenii Corynebacterium casei Corynebacterium vitaeruminis Corynebacterium glyciniphilum Corynebacterium atypicum Corynebacterium imitans Corynebacterium ureicelerivorans Corynebacterium sp. ATCC 6931 Corynebacterium doosanense Corynebacterium humireducens Corynebacterium singulare Corynebacterium marinum Corynebacterium kutscheri Corynebacterium camporealensis Corynebacterium mustelae Corynebacterium epidermidicanis Corynebacterium testudinoris Corynebacterium uterequi Corynebacterium lactis Corynebacterium deserti Corynebacterium simulans Corynebacterium stationis Corynebacterium crudilactis Corynebacterium frankenforstense Corynebacterium phocae Corynebacterium flavescens Corynebacterium glaucum Corynebacterium striatum Corynebacterium aquilae Corynebacterium sphenisci Corynebacterium ammoniagenes Corynebacterium minutissimum Corynebacterium pelargi Brevibacterium flavum Nocardia farcinica IFM 10152 Nocardia farcinica NCTC11134 Nocardia cyriacigeorgica Nocardia brasiliensis Nocardia nova Nocardia mangyaensis Nocardia seriolae Nocardia terpenica Nocardia sp. CS682 Nocardia otitidiscaviarum Rhodococcus jostii Rhodococcus erythropolis PR4 Rhodococcus erythropolis CCM2595 Rhodococcus erythropolis BG43 Rhodococcus opacus B4 Rhodococcus opacus PD630 Rhodococcus hoagii Rhodococcus pyridinivorans Rhodococcus sp. B7740 Rhodococcus aetherivorans Rhodococcus fascians Rhodococcus sp. WMMA185 Rhodococcus sp. PBTS 2 Rhodococcus ruber Rhodococcus sp. PBTS 1 Rhodococcus qingshengii Rhodococcus sp. p52 Rhodococcus sp. 008 Rhodococcus rhodochrous Rhodococcus biphenylivorans Gordonia bronchialis Gordonia polyisoprenivorans Gordonia sp. KTR9 Gordonia phthalatica Gordonia terrae Gordonia sp. YC-JH1 Gordonia iterans Gordonia rubripertincta Gordonia sp. MMS17-SY073 Gordonia alkanivorans Gordonia sp. 135 Tsukamurella paurometabola Tsukamurella tyrosinosolvens Segniliparus rotundus Dietzia timorensis Dietzia sp. oral taxon 368 Dietzia sp. JS16-p6b Dietzia psychralcaliphila Dietzia lutea Lawsonella clevelandensis Tomitella sp. HY188 Streptomyces coelicolor Streptomyces albidoflavus Streptomyces avermitilis Streptomyces griseus Streptomyces globisporus Streptomyces scabiei Streptomyces sp. SirexAA-E Streptomyces violaceusniger Streptomyces cattleya NRRL 8057 = DSM 46488 Streptomyces cattleya NRRL 8057 = DSM 46488 Streptomyces pratensis Streptomyces bingchenggensis Streptomyces hygroscopicus subsp. jinggangensis 5008 Streptomyces hygroscopicus subsp. jinggangensis TL01 Streptomyces venezuelae Streptomyces davaonensis Streptomyces albus DSM 41398 Streptomyces sp. PAMC 26508 Streptomyces fulvissimus Streptomyces collinus Streptomyces rapamycinicus Streptomyces albulus NK660 Streptomyces albulus ZPM Streptomyces lividans Streptomyces glaucescens Streptomyces vietnamensis Streptomyces sp. 769 Streptomyces cyaneogriseus Streptomyces lydicus A02 Streptomyces lydicus 103 Streptomyces xiamenensis Streptomyces sp. Mg1 Streptomyces sp. CNQ-509 Streptomyces ambofaciens Streptomyces pristinaespiralis Streptomyces sp. CFMR 7 Streptomyces sp. CdTB01 Streptomyces reticuli Streptomyces sp. 4F Streptomyces leeuwenhoekii Streptomyces rubrolavendulae Streptomyces parvulus Streptomyces sp. SAT1 Streptomyces clavuligerus Streptomyces griseochromogenes Streptomyces qaidamensis Streptomyces lincolnensis Streptomyces noursei Streptomyces pluripotens Streptomyces sp. CCM\_MD2014 Streptomyces niveus Streptomyces autolyticus Streptomyces alfalfae Streptomyces violaceoruber Streptomyces fodineus Streptomyces gilvosporeus Streptomyces malaysiensis Streptomyces laurentii Streptomyces alboflavus Streptomyces albireticuli Streptomyces lavendulae Streptomyces sp. MOE7 Streptomyces formicae Streptomyces nigra Streptomyces griseorubiginosus Streptomyces rochei Streptomyces lunaelactis Streptomyces koyangensis Kitasatospora setae Kitasatospora albolonga Kitasatospora aureofaciens Kitasatospora sp. MMS16-BH015 Streptacidiphilus sp. DSM 106435 Tropheryma whipplei Twist Tropheryma whipplei TW08/27 Luteimicrobium xylanilyticum Leifsonia xyli subsp. xyli CTCB07 Leifsonia xyli subsp. cynodontis DSM 46306 Clavibacter michiganensis subsp. michiganensis Clavibacter michiganensis subsp. sepedonicus Clavibacter michiganensis subsp. nebraskensis Clavibacter michiganensis subsp. insidiosus Clavibacter michiganensis subsp. capsici Microbacterium testaceum Microbacterium sp. CGR1 Microbacterium sp. No. 7 Microbacterium sp. XT11 Microbacterium sp. PAMC 28756 Microbacterium chocolatum Microbacterium paludicola Microbacterium sp. BH-3-3-3 Microbacterium sp. 1.5R Microbacterium aurum Microbacterium hominis Microbacterium foliorum Microbacterium oleivorans Microbacterium lemovicicum Rhodoluna lacicola Candidatus Rhodoluna planktonica Rathayibacter toxicus 70137 Rathayibacter toxicus WAC3373 Rathayibacter tritici Rathayibacter rathayi Rathayibacter iranicus Rathayibacter festucae Rathayibacter tanaceti Curtobacterium sp. MR\_MD2014 Curtobacterium sp. BH-2-1-1 Curtobacterium sp. SGAir0471 Curtobacterium flaccumfaciens Microterricola viridarii Frondihabitans sp. PAMC 28766 Agromyces aureus Agromyces sp. MF30-A Agromyces sp. FW100M-8 Cryobacterium arcticum Cryobacterium sp. LW097 Cnuibacter physcomitrellae Aurantimicrobium minutum Aurantimicrobium sp. MWH-Mo1 Aurantimicrobium sp. MWH-Uga1 Pontimonas salivibrio Microcella alkaliphila Mycetocola sp. 449 Salinibacterium sp. CGMCC 1.16371 Salinibacterium sp. UTAS2018 Salinibacterium sp. dk2585 Humibacter sp. BT305 Humibacter sp. WJ7-1 Gryllotalpicola sp. 2DFW10M-5 Protaetiibacter intestinalis Lysinimonas sp. KACC 19322 Plantibacter sp. PA-3-X8 Leucobacter sp. DSM 101948 Leucobacter triazinivorans Agrococcus sp. SGAir0287 Marisediminicola antarctica Arthrobacter sp. FB24 Arthrobacter sp. Rue61a Arthrobacter sp. PAMC 25486 Arthrobacter sp. LS16 Arthrobacter sp. ERGS1:01 Arthrobacter alpinus R3.8 Arthrobacter alpinus A3 Arthrobacter sp. Hiyo8 Arthrobacter sp. YC-RL1 Arthrobacter sp. ATCC 21022 Arthrobacter sp. U41 Arthrobacter sp. QXT-31 Arthrobacter sp. YN Arthrobacter sp. ZXY-2 Arthrobacter crystallopoietes Arthrobacter sp. PGP41 Arthrobacter sp. PAMC25564 Psychromicrobium lacuslunae Paenarthrobacter aurescens Pseudarthrobacter chlorophenolicus Pseudarthrobacter phenanthrenivorans Pseudarthrobacter sulfonivorans Pseudarthrobacter sp. NIBRBAC000502771 Pseudarthrobacter sp. YJ56 Glutamicibacter arilaitensis Glutamicibacter halophytocola Glutamicibacter creatinolyticus Glutamicibacter sp. ZJUTW Renibacterium salmoninarum Kocuria rhizophila Kocuria palustris Kocuria flava Kocuria indica Kocuria rosea Micrococcus luteus Micrococcus sp. KBS0714 Rothia mucilaginosa Rothia dentocariosa Rothia aeria Sinomonas atrocyanea Neomicrococcus aestuarii Auritidibacter sp. NML130574 Citricoccus sp. SGAir0453 Beutenbergia cavernae Brachybacterium faecium Brachybacterium sp. P6-10-X1 Brachybacterium sp. VR2415 Brachybacterium ginsengisoli Brachybacterium sp. VM2412 Brachybacterium saurashtrense Brachybacterium sp. SGAir0954 Dermabacter vaginalis Dermabacter jinjuensis Jonesia denitrificans Kytococcus sedentarius Dermacoccus nishinomiyaensis Dermacoccus abyssi Luteipulveratus mongoliensis Xylanimonas cellulosilytica Xylanimonas allomyrinae Isoptericola variabilis Isoptericola dokdonensis Cellulosimicrobium sp. TH-20 Cellulosimicrobium cellulans Xylanimicrobium sp. FW10M-9 Sanguibacter keddieii Cellulomonas flavigena Cellulomonas fimi Cellulomonas gilvus Cellulomonas sp. PSBB021 Cellulomonas sp. Z28 Cellulomonas sp. JZ18 Cellulomonas sp. H30R-01 Oerskovia sp. KBS0722 Pseudactinotalea sp. HY158 Intrasporangium calvum Arsenicicoccus sp. oral taxon 190 Serinicoccus sp. JLT9 Serinicoccus sp. W204 Janibacter indicus Janibacter limosus Janibacter melonis Ornithinimicrobium sp. AMA3305 Ornithinimicrobium sp. H23M54 Tetrasphaera sp. HKS02 Brevibacterium linens BS258 Brevibacterium aurantiacum Brevibacterium sp. CS2 Dermatophilus congolensis Georgenia sp. Z294 Cutibacterium acnes KPA171202 Cutibacterium acnes SK137 Cutibacterium acnes TypeIA2 P.acn17 Cutibacterium acnes TypeIA2 P.acn31 Cutibacterium acnes TypeIA2 P.acn33 Cutibacterium acnes 266 Cutibacterium acnes subsp. defendens ATCC 11828 Cutibacterium acnes 6609 Cutibacterium acnes C1 Cutibacterium acnes HL096PA1 Cutibacterium acnes hdn-1 Cutibacterium acnes KCOM 1861 (= ChDC B594) Cutibacterium avidum Cutibacterium granulosum Propionibacterium freudenreichii subsp. shermanii CIRM-BIA1 Propionibacterium freudenreichii subsp. freudenreichii DSM 20271 Propionibacterium sp. oral taxon 193 Propionibacterium acidifaciens Pseudopropionibacterium propionicum Acidipropionibacterium acidipropionici ATCC 4875 Acidipropionibacterium acidipropionici CGMCC 1.2230 Acidipropionibacterium virtanenii Acidipropionibacterium jensenii Microlunatus phosphovorus Microlunatus sp. KUDC0627 Microlunatus sp. Gsoil 973 Tessaracoccus flavus Tessaracoccus flavescens Tessaracoccus aquimaris Tessaracoccus sp. T2.5-30 Raineyella sp. CBA3103 Nocardioides sp. JS614 Nocardioides dokdonensis Nocardioides sp. MMS17-SY117 Nocardioides sp. dk3136 Nocardioides sp. S-1144 Nocardioides daphniae Kribbella flavida Pimelobacter simplex Aeromicrobium erythreum Aeromicrobium sp. 592 Aeromicrobium sp. A1-2 Aeromicrobium sp. MF47 Micropruina glycogenica Thermobifida fusca Nocardiopsis dassonvillei Nocardiopsis alba Nocardiopsis gilva Streptomonospora sp. M2 Thermomonospora curvata Actinomadura sp. WMMB499 Streptosporangium roseum Nonomuraea sp. ATCC 55076 Nonomuraea sp. WYY166 Frankia casuarinae Frankia sp. EAN1pec Frankia inefficax Frankia alni Candidatus Frankia datiscae Acidothermus cellulolyticus Nakamurella multipartita Nakamurella sp. s14-144 Geodermatophilus obscurus Blastococcus saxobsidens Modestobacter marinus Kineococcus radiotolerans Saccharopolyspora erythraea Saccharopolyspora sp. E2A Saccharomonospora viridis Saccharomonospora sp. 31sw Amycolatopsis mediterranei U32 Amycolatopsis mediterranei S699 Amycolatopsis mediterranei S699 Amycolatopsis mediterranei RB Amycolatopsis orientalis Amycolatopsis japonica Amycolatopsis methanolica Amycolatopsis sp. AA4 Amycolatopsis sp. BJA-103 Amycolatopsis albispora Amycolatopsis sp. YIM 10 Pseudonocardia dioxanivorans Pseudonocardia sp. AL041005-10 Pseudonocardia sp. EC080625-04 Pseudonocardia sp. HH130629-09 Pseudonocardia sp. EC080610-09 Pseudonocardia sp EC080619-01 Pseudonocardia sp. HH130630-07 Pseudonocardia autotrophica Actinosynnema pretiosum Actinosynnema mirum Saccharothrix espanaensis Saccharothrix syringae Kutzneria albida Kibdelosporangium phytohabitans Lentzea guizhouensis Actinoalloteichus hymeniacidonis Actinoalloteichus sp. GBA129-24 Actinoalloteichus fjordicus Actinoalloteichus hoggarensis Actinoalloteichus sp. AHMU CJ021 Alloactinosynnema sp. L-07 Prauserella marina Salinispora tropica Salinispora arenicola Micromonospora aurantiaca Micromonospora sp. L5 Micromonospora sp. B006 Micromonospora tulbaghiae Micromonospora sp. HM134 Micromonospora terminaliae Verrucosispora maris Actinoplanes sp. SE50/110 Actinoplanes missouriensis Actinoplanes sp. N902-109 Actinoplanes friuliensis Actinoplanes sp. SE50 Plantactinospora sp. KBS50 Plantactinospora sp. BC1 Plantactinospora sp. BB1 Catenulispora acidiphila Stackebrandtia nassauensis Arcanobacterium haemolyticum Mobiluncus curtisii Trueperella pyogenes TP6375 Trueperella pyogenes TP8 Trueperella bialowiezensis Actinotignum schaalii Actinobaculum sp. 313 Schaalia meyeri Schaalia odontolytica Actinomyces sp. oral taxon 414 Actinomyces oris Actinomyces radicidentis Actinomyces tangfeifanii Actinomyces gaoshouyii Actinomyces sp. Chiba101 Actinomyces sp. oral taxon 897 Actinomyces naeslundii Actinomyces howellii Actinomyces sp. 432 Flaviflexus salsibiostraticola Flaviflexus sp. H23T48 Bifidobacterium longum NCC2705 Bifidobacterium longum DJO10A Bifidobacterium longum subsp. infantis ATCC 15697 (JGI) Bifidobacterium longum subsp. infantis ATCC 15697 (Tokyo) Bifidobacterium longum subsp. infantis 157F Bifidobacterium longum subsp. longum JDM301 Bifidobacterium longum subsp. longum BBMN68 Bifidobacterium longum subsp. longum JCM 1217 Bifidobacterium longum subsp. longum KACC 91563 Bifidobacterium longum subsp. longum F8 Bifidobacterium longum subsp. longum GT15 Bifidobacterium longum BXY01 Bifidobacterium adolescentis ATCC 15703 Bifidobacterium adolescentis 22L Bifidobacterium adolescentis BBMN23 Bifidobacterium animalis subsp. lactis AD011 Bifidobacterium animalis subsp. lactis Bl-04 Bifidobacterium animalis subsp. lactis DSM 10140 Bifidobacterium animalis subsp. lactis BB-12 Bifidobacterium animalis subsp. lactis BLC1 Bifidobacterium animalis subsp. lactis CNCM I-2494 Bifidobacterium animalis subsp. lactis V9 Bifidobacterium animalis subsp. lactis B420 Bifidobacterium animalis subsp. lactis Bi-07 Bifidobacterium animalis subsp. lactis Bl12 Bifidobacterium animalis subsp. lactis ATCC 27673 Bifidobacterium animalis subsp. animalis ATCC 25527 Bifidobacterium animalis RH Bifidobacterium dentium Bd1 Bifidobacterium dentium JCM 1195 = DSM 20436 Bifidobacterium bifidum PRL2010 Bifidobacterium bifidum S17 Bifidobacterium bifidum BGN4 Bifidobacterium breve ACS-071-V-Sch8b Bifidobacterium breve UCC2003 Bifidobacterium breve 12L Bifidobacterium breve 689b Bifidobacterium breve JCM 7017 Bifidobacterium breve JCM 7019 Bifidobacterium breve NCFB 2258 Bifidobacterium breve S27 Bifidobacterium breve DSM 20213 = JCM 1192 Bifidobacterium asteroides Bifidobacterium thermophilum Bifidobacterium coryneforme Bifidobacterium kashiwanohense PV20-2 Bifidobacterium kashiwanohense JCM 15439 = DSM 21854 Bifidobacterium catenulatum Bifidobacterium pseudolongum Bifidobacterium indicum Bifidobacterium angulatum Bifidobacterium pseudocatenulatum Bifidobacterium scardovii Bifidobacterium actinocoloniiforme Bifidobacterium choerinum Gardnerella vaginalis ATCC 14019 Gardnerella vaginalis 409-05 Gardnerella vaginalis HMP9231 Scardovia inopinata Parascardovia denticolens Thermobispora bispora Actinopolyspora erythraea Candidatus Planktophila lacus Candidatus Planktophila dulcis Candidatus Planktophila limnetica Candidatus Planktophila sulfonica Candidatus Planktophila versatilis Candidatus Planktophila vernalis Candidatus Nanopelagicus limnes Candidatus Nanopelagicus hibericus Candidatus Nanopelagicus abundans Epidermidibacterium keratini Actinobacteria bacterium Rubrobacter xylanophilus Rubrobacter radiotolerans Baekduia soli Conexibacter woesei Acidimicrobium ferrooxidans Ilumatobacter coccineus Cryptobacterium curtum Slackia heliotrinireducens Eggerthella lenta Eggerthella sp. YY7918 Gordonibacter pamelaeae Adlercreutzia equolifaciens Denitrobacterium detoxificans Atopobium parvulum Olsenella uli Olsenella sp. oral taxon 807 Parolsenella catena Coriobacterium glomerans Collinsella aerofaciens Coriobacteriaceae bacterium 68-1-3 Egibacter rhizosphaerae Euzebya sp. DY32-46 Synechocystis sp. PCC 6803 Synechocystis sp. PCC 6803 Synechocystis sp. PCC 6803 GT-S Synechocystis sp. PCC 6803 GT-I Synechocystis sp. PCC 6803 PCC-N Synechocystis sp. PCC 6803 PCC-P Synechocystis sp. PCC 6714 Synechocystis sp. IPPAS B-1465 Synechococcus elongatus PCC6301 Synechococcus elongatus PCC7942 Synechococcus sp. WH8102 Synechococcus sp. CC9605 Synechococcus sp. CC9902 Synechococcus sp. CC9311 Synechococcus sp. RCC307 Synechococcus sp. WH7803 Synechococcus sp. PCC7002 Synechococcus sp. JA-3-3Ab Synechococcus sp. JA-2-3B'a(2-13) Synechococcus sp. PCC 6312 Synechococcus sp. PCC 7502 Synechococcus sp. KORDI-100 Synechococcus sp. KORDI-49 Synechococcus sp. KORDI-52 Synechococcus sp. UTEX 2973 Synechococcus sp. WH 8109 Synechococcus sp. WH 8103 Synechococcus lividus Synechococcus sp. PCC 73109 Synechococcus sp. PCC 7003 Synechococcus sp. CB0101 Thermosynechococcus elongatus Thermosynechococcus sp. NK55 Thermosynechococcus vulcanus Thermosynechococcus sp. CL-1 Cyanobium gracile Cyanobium sp. NIES-981 Dactylococcopsis salina Chamaesiphon minutus Leptolyngbya sp. PCC 7376 Leptolyngbya sp. NIES-3755 Leptolyngbya sp. O-77 Leptolyngbya boryana Halomicronema hongdechloris Pseudanabaena sp. PCC 7367 Pseudanabaena sp. ABRG5-3 Prochlorococcus marinus subsp. marinus CCMP1375 Prochlorococcus marinus subsp. pastoris CCMP1986 Prochlorococcus marinus MIT 9313 Prochlorococcus marinus NATL2A Prochlorococcus marinus MIT 9312 Prochlorococcus marinus AS9601 Prochlorococcus marinus MIT 9515 Prochlorococcus marinus MIT 9303 Prochlorococcus marinus MIT 9301 Prochlorococcus marinus MIT 9215 Prochlorococcus marinus MIT 9211 Prochlorococcus marinus NATL1A Prochlorococcus sp. MIT 0604 Prochlorococcus sp. MIT 0801 Acaryochloris marina Gloeocapsa sp. PCC 7428 Geminocystis sp. NIES-3709 Geminocystis sp. NIES-3708 Chondrocystis sp. NIES-4102 Microcystis aeruginosa Microcystis panniformis Microcystis sp. MC19 Microcystis viridis Cyanobacterium aponinum Cyanobacterium stanieri Cyanobacterium sp. HL-69 Halothece sp. PCC 7418 Euhalothece natronophila Candidatus Atelocyanobacterium thalassa Crocosphaera subtropica Crocosphaera watsonii Rippkaea orientalis PCC 8801 Rippkaea orientalis PCC 8802 Gloeothece citriformis Gloeothece verrucosa Cyanothece sp. PCC 7425 Trichodesmium erythraeum Microcoleus sp. PCC 7113 Arthrospira platensis Planktothrix agardhii Geitlerinema sp. PCC 7407 Oscillatoria acuminata Oscillatoria nigro-viridis Moorea producens Crinalium epipsammum Gloeobacter violaceus Gloeobacter kilaueensis Nostoc sp. PCC 7120 Nostoc punctiforme Nostoc sp. PCC 7107 Nostoc sp. PCC 7524 Nostoc sp. NIES-3756 Nostoc flagelliforme Nostoc sp. CENA543 Nostoc sphaeroides Trichormus variabilis 'Nostoc azollae' 0708 Anabaena sp. 90 Anabaena cylindrica Anabaena sp. WA102 Anabaena sp. YBS01 Cylindrospermum stagnale Calothrix sp. PCC 7507 Calothrix sp. PCC 6303 Calothrix sp. 336/3 Rivularia sp. PCC 7116 Fischerella sp. NIES-3754 Nodularia spumigena Dolichospermum sp. UHCC 0315A Nostocales cyanobacterium HT-58-2 Chroococcidiopsis thermalis Pleurocapsa sp. PCC 7327 Stanieria cyanosphaera Stanieria sp. NIES-3757 Cyanobacterium endosymbiont of Epithemia turgida Cyanobacterium endosymbiont of Rhopalodia gibberula Candidatus Melainabacteria bacterium MEL.A1 Dehalococcoides mccartyi 195 Dehalococcoides mccartyi CBDB1 Dehalococcoides mccartyi BAV1 Dehalococcoides mccartyi VS Dehalococcoides mccartyi GT Dehalococcoides mccartyi BTF08 Dehalococcoides mccartyi DCMB5 Dehalococcoides mccartyi GY50 Dehalococcoides mccartyi CG1 Dehalococcoides mccartyi CG4 Dehalococcoides mccartyi CG5 Dehalococcoides sp. UCH007 Dehalogenimonas lykanthroporepellens Dehalogenimonas sp. WBC-2 Dehalogenimonas formicexedens Roseiflexus sp. RS-1 Roseiflexus castenholzii Chloroflexus aurantiacus Chloroflexus aurantiacus Y-400-fl Chloroflexus aggregans Herpetosiphon aurantiacus Thermomicrobium roseum Sphaerobacter thermophilus Anaerolinea thermophila Brevefilum fermentans Pelolinea submarina Anaerolineaceae bacterium oral taxon 439 Caldilinea aerophila Candidatus Promineofilum breve Ktedonobacterales bacterium SCAWS-G2 Tepidiforma bonchosmolovskayae Deinococcus radiodurans Deinococcus geothermalis Deinococcus deserti Deinococcus maricopensis Deinococcus proteolyticus Deinococcus gobiensis Deinococcus peraridilitoris Deinococcus swuensis Deinococcus soli Deinococcus actinosclerus Deinococcus puniceus Deinococcus irradiatisoli Deinococcus wulumuqiensis Deinococcus ficus Deinococcus sp. AJ005 Truepera radiovictrix Thermus thermophilus HB27 Thermus thermophilus HB8 Thermus thermophilus SG0.5JP17-16 Thermus thermophilus JL-18 Thermus scotoductus Thermus sp. CCB\_US3\_UF1 Thermus oshimai Thermus aquaticus Thermus parvatiensis Thermus brockianus Meiothermus ruber DSM 1279 Meiothermus ruber DSM 1279 Meiothermus silvanus Meiothermus taiwanensis Oceanithermus profundus Marinithermus hydrothermalis Chthonomonas calidirosea Fimbriimonas ginsengisoli Thermobaculum terrenum Chlamydia trachomatis D/UW-3/CX Chlamydia trachomatis D-EC Chlamydia trachomatis D-LC Chlamydia trachomatis D/SotonD1 Chlamydia trachomatis D/SotonD5 Chlamydia trachomatis D/SotonD6 Chlamydia trachomatis A/HAR-13 Chlamydia trachomatis A2497 Chlamydia trachomatis A2497 Chlamydia trachomatis A/363 Chlamydia trachomatis A/5291 Chlamydia trachomatis A/7249 Chlamydia trachomatis L1/1322/p2 Chlamydia trachomatis L1/115 Chlamydia trachomatis L1/224 Chlamydia trachomatis L1/440/LN Chlamydia trachomatis 434/Bu Chlamydia trachomatis L2/25667R Chlamydia trachomatis L2/434/Bu(f) Chlamydia trachomatis L2/434/Bu(i) Chlamydia trachomatis L2b/UCH-1/proctitis Chlamydia trachomatis L2b/UCH-2 Chlamydia trachomatis L2b/LST Chlamydia trachomatis L2b/CV204 Chlamydia trachomatis L2b/Ams1 Chlamydia trachomatis L2b/Ams2 Chlamydia trachomatis L2b/Ams3 Chlamydia trachomatis L2b/Ams4 Chlamydia trachomatis L2b/Ams5 Chlamydia trachomatis L2b/Canada1 Chlamydia trachomatis L2b/Canada2 Chlamydia trachomatis L2b/795 Chlamydia trachomatis L2b/8200/07 Chlamydia trachomatis L2c Chlamydia trachomatis L3/404/LN Chlamydia trachomatis B/Jali20/OT Chlamydia trachomatis B/TZ1A828/OT Chlamydia trachomatis E/11023 Chlamydia trachomatis E/150 Chlamydia trachomatis Sweden2 Chlamydia trachomatis E/SW3 Chlamydia trachomatis E/Bour Chlamydia trachomatis E/SotonE4 Chlamydia trachomatis E/SotonE8 Chlamydia trachomatis E/C599 Chlamydia trachomatis F/SW4 Chlamydia trachomatis F/SW5 Chlamydia trachomatis F/SWFPminus Chlamydia trachomatis F/SotonF3 Chlamydia trachomatis F/11-96 Chlamydia trachomatis G/11074 Chlamydia trachomatis G/11222 Chlamydia trachomatis G/9301 Chlamydia trachomatis G/9768 Chlamydia trachomatis G/SotonG1 Chlamydia trachomatis IU824 Chlamydia trachomatis IU888 Chlamydia trachomatis Ia/SotonIa1 Chlamydia trachomatis Ia/SotonIa3 Chlamydia trachomatis K/SotonK1 Chlamydia trachomatis J/6276tet1 Chlamydia trachomatis RC-F/69 Chlamydia trachomatis RC-F(s)/342 Chlamydia trachomatis RC-F(s)/852 Chlamydia trachomatis RC-J/943 Chlamydia trachomatis RC-J/953 Chlamydia trachomatis RC-J/966 Chlamydia trachomatis RC-J/971 Chlamydia trachomatis RC-J(s)/122 Chlamydia trachomatis RC-L2/55 Chlamydia trachomatis RC-L2(s)/3 Chlamydia trachomatis RC-L2(s)/46 Chlamydia trachomatis C/TW-3 Chlamydia muridarum Nigg Chlamydia muridarum Nigg CM972 Chlamydia muridarum Nigg 2 MCR Chlamydia muridarum Nigg3 clone G0.1.1 Chlamydia muridarum Nigg3 clone G28.38.1 Chlamydia muridarum Nigg3 Chlamydia muridarum Nigg3 CMUT3-5 Chlamydia pneumoniae CWL029 Chlamydia pneumoniae AR39 Chlamydia pneumoniae J138 Chlamydia pneumoniae TW-183 Chlamydia pneumoniae LPCoLN Chlamydia pecorum E58 Chlamydia pecorum P787 Chlamydia pecorum PV3056/3 Chlamydia pecorum W73 Chlamydia psittaci 6BC Chlamydia psittaci 6BC Chlamydia psittaci 01DC11 Chlamydia psittaci 02DC15 Chlamydia psittaci 08DC60 Chlamydia psittaci C19/98 Chlamydia psittaci RD1 Chlamydia psittaci CP3 Chlamydia psittaci NJ1 Chlamydia psittaci 84/55 Chlamydia psittaci GR9 Chlamydia psittaci M56 Chlamydia psittaci MN Chlamydia psittaci VS225 Chlamydia psittaci WC Chlamydia psittaci WS/RT/E30 Chlamydia psittaci 01DC12 Chlamydia psittaci Mat116 Chlamydia avium Chlamydia caviae Chlamydia abortus S26/3 Chlamydia abortus AB7 Chlamydia felis Fe/C-56 Chlamydia gallinacea Chlamydia sp. S15-834C Candidatus Protochlamydia amoebophila Candidatus Protochlamydia naegleriophila Parachlamydia acanthamoebae Neochlamydia sp. S13 Waddlia chondrophila Simkania negevensis Opitutus terrae Lacunisphaera limnophila Nibricoccus aquaticus HZ-65 Opitutaceae bacterium TAV5 Coraliomargarita akajimensis Akkermansia muciniphila Akkermansia glycaniphila Roseimicrobium sp. ORNL1 Candidatus Xiphinematobacter sp. Idaho Grape Methylacidiphilum infernorum Methylacidiphilum kamchatkense Verrucomicrobia bacterium IMCC26134 Verrucomicrobia bacterium S94 Rhodopirellula baltica Pirellula staleyi Pirellula sp. SH-Sr6A Roseimaritima ulvae Mariniblastus fucicola Thermogutta terrifontis Planctopirus limnophila Planctopirus ephydatiae Rubinisphaera brasiliensis Planctomyces sp. SH-PL14 Planctomyces sp. SH-PL62 Fuerstia marisgermanicae Gimesia maris Gimesia sp. E7 Bremerella volcania Gemmata sp. SH-PL17 Gemmata obscuriglobus Tuwongella immobilis Isosphaera pallida Singulisphaera acidiphila Paludisphaera borealis Aquisphaera giovannonii Candidatus Kuenenia stuttgartiensis Phycisphaera mikurensis Sedimentisphaera cyanobacteriorum L21-RPul-D3 Sedimentisphaera salicampi Limihaloglobus sulfuriphilus Anaerohalosphaera lusitana Kiritimatiella glycovorans Victivallales bacterium CCUG 44730 Borreliella burgdorferi B31 Borreliella burgdorferi ZS7 Borreliella burgdorferi N40 Borreliella burgdorferi JD1 Borrelia burgdorferi CA382 Borreliella bavariensis Borreliella garinii BgVir Borreliella garinii NMJW1 Borreliella garinii SZ Borreliella garinii CIP 103362 Borrelia afzelii (FLI) Borrelia afzelii (Maryland) Borrelia afzelii HLJ01 Borreliella afzelii Tom3107 Borreliella afzelii K78 Borreliella bissettii Borreliella valaisiana Borreliella chilensis Borrelia mayonii Borrelia turicatae Borrelia hermsii DAH Borrelia hermsii CC1 Borrelia duttonii Borrelia recurrentis Borrelia crocidurae Borrelia miyamotoi LB-2001 Borrelia miyamotoi CT14D4 Borrelia parkeri Borrelia anserina Borrelia turcica Treponema pallidum subsp. pallidum Nichols Treponema pallidum subsp. pallidum Nichols Treponema pallidum subsp. pallidum SS14 Treponema pallidum subsp. pallidum DAL-1 Treponema pallidum subsp. pallidum Chicago Treponema pallidum subsp. pallidum Mexico A Treponema pallidum subsp. pallidum Sea 81-4 Treponema pallidum subsp. pertenue CDC2 Treponema pallidum subsp. pertenue Gauthier Treponema pallidum subsp. pertenue SamoaD Treponema pallidum Fribourg-Blanc Treponema denticola Treponema succinifaciens Treponema brennaborense Treponema azotonutricium Treponema primitia Treponema paraluiscuniculi Treponema pedis Treponema caldarium Treponema putidum Treponema sp. OMZ 838 Treponema phagedenis Treponema sp. OMZ 804 Sediminispirochaeta smaragdinae Spirochaeta thermophila DSM 6192 Spirochaeta thermophila DSM 6578 Spirochaeta africana Spirochaeta perfilievii Sphaerochaeta globosa Sphaerochaeta coccoides Sphaerochaeta pleomorpha Salinispira pacifica Oceanispirochaeta sp. K2 Leptospira interrogans serovar Lai 56601 Leptospira interrogans serovar Lai IPAV Leptospira interrogans serovar Copenhageni Leptospira interrogans serovar Linhai Leptospira borgpetersenii JB197 Leptospira borgpetersenii L550 Leptospira biflexa serovar Patoc Patoc 1 (Paris) Leptospira biflexa serovar Patoc Patoc 1 (Ames) Leptospira santarosai Leptospira tipperaryensis Leptospira mayottensis Leptospira kmetyi Leptospira weilii Turneriella parva Brachyspira hyodysenteriae WA1 Brachyspira hyodysenteriae ATCC 27164 Brachyspira murdochii Brachyspira pilosicoli 95/1000 Brachyspira pilosicoli B2904 Brachyspira pilosicoli P43/6/78 Brachyspira pilosicoli WesB Brachyspira intermedia Brachyspira hampsonii Candidatus Koribacter versatilis Acidobacterium capsulatum Granulicella tundricola Granulicella mallensis Granulicella sp. WH15 Terriglobus saanensis Terriglobus roseus Terriglobus albidus Acidisarcina polymorpha Edaphobacter sp. 12200R-103 Candidatus Solibacter usitatus Chloracidobacterium thermophilum Luteitalea pratensis Elusimicrobium minutum Endomicrobium proavitum Candidatus Endomicrobium trichonymphae Uncultured Termite group 1 bacterium phylotype Rs-D17 Fusobacterium nucleatum subsp. nucleatum ATCC 25586 Fusobacterium nucleatum subsp. vincentii 3\_1\_36A2 Fusobacterium nucleatum subsp. vincentii 3\_1\_27 Fusobacterium nucleatum subsp. animalis 4\_8 Fusobacterium nucleatum subsp. animalis 7\_1 Fusobacterium hwasookii Fusobacterium pseudoperiodonticum Fusobacterium varium Fusobacterium ulcerans Fusobacterium mortiferum Fusobacterium gonidiaformans Fusobacterium necrophorum subsp. funduliforme Fusobacterium periodonticum Ilyobacter polytropus Leptotrichia buccalis Leptotrichia sp. oral taxon 212 Leptotrichia sp. oral taxon 847 Leptotrichia sp. oral taxon 498 Leptotrichia hofstadii Sebaldella termitidis Streptobacillus moniliformis Sneathia amnii Thermanaerovibrio acidaminovorans Aminobacterium colombiense Thermovirga lienii Acetomicrobium mobile Fretibacterium fastidiosum Cloacibacillus porcorum Fibrobacter succinogenes Fibrobacter succinogenes Gemmatimonas aurantiaca Gemmatimonas phototrophica Gemmatirosa kalamazoonesis Bacteroides thetaiotaomicron VPI-5482 Bacteroides thetaiotaomicron 7330 Bacteroides fragilis YCH46 Bacteroides fragilis NCTC 9343 Bacteroides fragilis 638R Bacteroides fragilis BOB25 Bacteroides vulgatus Bacteroides helcogenes Bacteroides salanitronis Bacteroides xylanisolvens Bacteroides dorei HS1\_L\_1\_B\_010 Bacteroides dorei HS1\_L\_3\_B\_079 Bacteroides ovatus Bacteroides cellulosilyticus Bacteroides caccae Bacteroides caecimuris Bacteroides zoogleoformans Bacteroides heparinolyticus Bacteroides intestinalis Porphyromonas gingivalis W83 Porphyromonas gingivalis ATCC 33277 Porphyromonas gingivalis TDC60 Porphyromonas asaccharolytica Porphyromonas crevioricanis Fermentimonas caenicola Petrimonas mucosa Petrimonas sp. IBARAKI Paludibacter propionicigenes Parabacteroides distasonis Parabacteroides sp. CT06 Tannerella forsythia Tannerella sp. oral taxon HOT-286 Muribaculum intestinale Duncaniella sp. B8 Barnesiella viscericola Proteiniphilum saccharofermentans Odoribacter splanchnicus Butyricimonas sp. H184 Candidatus Azobacteroides pseudotrichonymphae (Coptotermes formosanus) Prevotella ruminicola Prevotella melaninogenica Prevotella denticola Prevotella intermedia Prevotella dentalis Prevotella sp. oral taxon 299 Prevotella fusca Prevotella enoeca Prevotella jejuni Prevotella oris Alloprevotella sp. E39 Alistipes finegoldii Alistipes shahii Alistipes sp. dk3624 Alistipes onderdonkii subsp. vulgaris Mucinivorans hirudinis Bacteroidales bacterium CF Draconibacterium orientale Draconibacterium sp. M1 Salinivirga cyanobacteriivorans Alkalitalea saponilacus Labilibaculum antarcticum Salinibacter ruber DSM 13855 Salinibacter ruber M8 Rhodothermus marinus DSM 4252 Rhodothermus marinus SG0.5JP17-172 Rhodothermaceae bacterium RA Chitinophaga pinensis Chitinophaga caeni Chitinophaga sp. XS-30 Chitinophaga sp. H33E-04 Niastella koreensis Niabella soli Niabella ginsenosidivorans Flavisolibacter tropicus Flavisolibacter ginsenosidimutans Arachidicoccus sp. BS20 Arachidicoccus sp. KIS59-12 Arachidicoccus ginsenosidivorans Arachidicoccus sp. B3-10 Filimonas lacunae Pseudoflavitalea sp. 5GH32-13 Panacibacter ginsenosidivorans Pseudobacter ginsenosidimutans Flavihumibacter sp. SB-02 Haliscomenobacter hydrossis Saprospira grandis Pedobacter heparinus Pedobacter sp. PACM 27299 Pedobacter cryoconitis Pedobacter steynii Pedobacter ginsengisoli Pedobacter sp. CJ43 Pedobacter sp. KBS0701 Pseudopedobacter saltans Sphingobacterium sp. 21 Sphingobacterium sp. ML3W Sphingobacterium sp. B29 Sphingobacterium mizutaii Sphingobacterium psychroaquaticum Sphingobacterium sp. CZ-2 Sphingobacterium sp. dk4302 Sphingobacterium sp. DR205 Solitalea canadensis Mucilaginibacter sp. PAMC 26640 Mucilaginibacter sp. BJC16-A31 Mucilaginibacter gotjawali Mucilaginibacter sp. HYN0043 Mucilaginibacter ginsenosidivorans Mucilaginibacter ginsenosidivorax Mucilaginibacter rubeus Mucilaginibacter gossypii Anseongella ginsenosidimutans Olivibacter sp. LS-1 Sphingobacteriaceae bacterium GW460-11-11-14-LB5 Cyclobacterium marinum Cyclobacterium amurskyense Belliella baltica Echinicola vietnamensis Echinicola strongylocentroti Echinicola sp. LN3S3 Algoriphagus sanaruensis Cytophaga hutchinsonii Dyadobacter fermentans Spirosoma linguale Spirosoma radiotolerans Spirosoma montaniterrae Spirosoma pollinicola Spirosoma sp. KCTC 42546 Leadbetterella byssophila Runella slithyformis Runella sp. HYN0085 Runella sp. SP2 Emticicia oligotrophica Fibrella aestuarina Fibrella sp. ES10-3-2-2 Allopseudarcicella aquatilis Arcticibacterium luteifluviistationis Rhodocytophaga sp. 172606-1 Bernardetia litoralis Hymenobacter swuensis Hymenobacter sp. APR13 Hymenobacter sp. DG25B Hymenobacter sp. DG25A Hymenobacter sedentarius Hymenobacter sp. PAMC 26554 Hymenobacter sp. PAMC 26628 Hymenobacter nivis Hymenobacter sp. sh-6 Hymenobacter sp. 17J68-5 Pontibacter korlensis Pontibacter actiniarum Rufibacter sp. DG31D Rufibacter tibetensis Rufibacter sp. DG15C Nibribacter sp. BT10 Marivirga tractuosa Flammeovirga sp. MY04 Flammeovirga sp. L12M1 Fabibacter pacificus Flammeovirgaceae bacterium 311 Candidatus Amoebophilus asiaticus Cardinium endosymbiont cEper1 (Encarsia pergandiella) Cardinium endosymbiont of Sogatella furcifera Candidatus Cardinium hertigii Chryseolinea sp. KIS68-18 Gramella forsetii Gramella salexigens Gramella flava Gramella fulva Flavobacterium psychrophilum JIP02/86 Flavobacterium psychrophilum CSF259-93 Flavobacterium psychrophilum FPG101 Flavobacterium psychrophilum FPG3 Flavobacterium psychrophilum 950106-1/1 Flavobacterium psychrophilum V3-5 Flavobacterium psychrophilum V4-24 Flavobacterium psychrophilum v4-33 Flavobacterium psychrophilum Z2 Flavobacterium johnsoniae UW101 Flavobacterium anhuiense Flavobacterium branchiophilum Flavobacterium columnare Flavobacterium indicum Flavobacterium gilvum Flavobacterium commune Flavobacterium faecale Flavobacterium arcticum Flavobacterium kingsejongi Flavobacterium pallidum Flavobacterium magnum Capnocytophaga ochracea Capnocytophaga canimorsus Capnocytophaga sp. oral taxon 323 Capnocytophaga haemolytica Capnocytophaga endodontalis Capnocytophaga gingivalis Capnocytophaga leadbetteri Capnocytophaga sputigena Capnocytophaga cynodegmi Capnocytophaga sp. H4358 Capnocytophaga stomatis Capnocytophaga sp. H2931 Capnocytophaga sp. FDAARGOS\_737 Robiginitalea biformata Zunongwangia profunda Croceibacter atlanticus Riemerella anatipestifer ATCC 11845 = DSM 15868 Riemerella anatipestifer ATCC 11845 = DSM 15868 Riemerella anatipestifer RA-GD Riemerella anatipestifer RA-CH-1 Riemerella anatipestifer RA-CH-2 Riemerella anatipestifer CH3 Maribacter sp. HTCC2170 Maribacter sp. 1\_2014MBL\_MicDiv Maribacter sp. T28 Maribacter cobaltidurans Maribacter sp. MJ134 Cellulophaga algicola Cellulophaga lytica DSM 7489 Cellulophaga lytica HI1 Cellulophaga baltica NN016038 Cellulophaga baltica 18 Weeksella virosa Dokdonia sp. 4H-3-7-5 Dokdonia sp. MED134 Dokdonia donghaensis Dokdonia sp. Dokd-P16 Lacinutrix sp. 5H-3-7-4 Lacinutrix venerupis Lacinutrix sp. Bg11-31 Zobellia galactanivorans Muricauda ruestringensis Muricauda lutaonensis Aequorivita sublithincola Aequorivita sp. H23M31 Ornithobacterium rhinotracheale DSM 15997 Ornithobacterium rhinotracheale ORT-UMN 88 Psychroflexus torquis Nonlabens dokdonensis Nonlabens sp. MIC269 Nonlabens sediminis Nonlabens sp. MB-3u-79 Nonlabens sp. MJ115 Polaribacter sp. MED152 Polaribacter vadi Polaribacter reichenbachii Polaribacter sp. BM10 Polaribacter sp. ALD11 Elizabethkingia anophelis NUHP1 Elizabethkingia anophelis FMS-007 Elizabethkingia anophelis JM-87 Elizabethkingia miricola Elizabethkingia meningoseptica Elizabethkingia ursingii Elizabethkingia bruuniana Elizabethkingia sp. 2-6 Elizabethkingia sp. JS20170427COW Myroides sp. A21 Myroides profundi Myroides odoratimimus Myroides sp. ZB35 Chryseobacterium sp. StRB126 Chryseobacterium gallinarum Chryseobacterium sp. IHB B 17019 Chryseobacterium glaciei Chryseobacterium indologenes Chryseobacterium sp. T16E-39 Chryseobacterium piperi Chryseobacterium taklimakanense Chryseobacterium sp. 3008163 Chryseobacterium sp. 6424 Chryseobacterium arthrosphaerae Chryseobacterium shandongense Chryseobacterium nakagawai Winogradskyella sp. PG-2 Winogradskyella sp. J14-2 Siansivirga zeaxanthinifaciens Algibacter alginicilyticus Sediminicola sp. YIK13 Tenacibaculum dicentrarchi Tenacibaculum sp. LPB0136 Tenacibaculum jejuense Tenacibaculum maritimum Tenacibaculum mesophilum Lutibacter profundi Urechidicola croceus Wenyingzhuangia fucanilytica Formosa sp. Hel1\_33\_131 Formosa sp. Hel3\_A1\_48 Formosa sp. PS13 Salegentibacter sp. T436 Seonamhaeicola sp. S2-3 Arenibacter algicola Olleya sp. Bg11-27 Olleya aquimaris Flavivirga eckloniae Tamlana sp. UJ94 Aureitalea sp. RR4-38 Flagellimonas sp. HME9304 Kordia sp. SMS9 Kordia antarctica Mariniflexile sp. TRM1-10 Aquimarina sp. BL5 Aquimarina sp. AD1 Aquimarina sp. AD10 Euzebyella marina Cloacibacterium normanense Muriicola sp. MMS17-SY002 Psychroserpens sp. NJDZ02 Antarcticibacterium flavum Antarcticibacterium sp. PAMC 28998 Empedobacter brevis Oceanihabitans sp. IOP\_32 Bergeyella cardium Marinirhabdus gelatinilytica Apibacter sp. B2966 Mesoflavibacter sp. HG96 Flavobacteriaceae bacterium 3519-10 Flavobacteriaceae bacterium UJ101 Flavobacteriaceae bacterium 10Alg115 Blattabacterium sp. (Blattella germanica) Bge Blattabacterium sp. BPLAN (Periplaneta americana) Blattabacterium sp. (Mastotermes darwiniensis) Blattabacterium Cpu (Cryptocercus punctulatus) Blattabacterium sp. (Blaberus giganteus) Blattabacterium sp. (Blatta orientalis) Blattabacterium cuenoti Blattabacterium sp. (Nauphoeta cinerea) Blattabacterium sp. (Cryptocercus kyebangensis) Fluviicola taffensis Candidatus Fluviicola riflensis Owenweeksia hongkongensis Ichthyobacterium seriolicida Bacteroidetes bacterium Chlorobaculum tepidum Chlorobaculum parvum Chlorobaculum limnaeum Chlorobium chlorochromatii Chlorobium phaeobacteroides DSM 266 Chlorobium phaeobacteroides BS1 Chlorobium limicola Chlorobium phaeovibrioides Chlorobium luteolum Pelodictyon phaeoclathratiforme Prosthecochloris aestuarii Prosthecochloris sp. CIB 2401 Prosthecochloris sp. HL-130-GSB Prosthecochloris sp. GSB1 Chloroherpeton thalassium Ignavibacterium album Melioribacter roseus Candidatus Cyclonatronum proteinivorum Candidatus Cloacimonas acidaminovorans Aquifex aeolicus Hydrogenobaculum sp. Y04AAS1 Hydrogenobaculum sp. HO Hydrogenobaculum sp. SN Hydrogenobacter thermophilus Hydrogenobacter thermophilus Thermocrinis albus Thermocrinis ruber Sulfurihydrogenibium sp. YO3AOP1 Sulfurihydrogenibium azorense Persephonella marina Thermosulfidibacter takaii Thermovibrio ammonificans Desulfurobacterium thermolithotrophum Thermotoga maritima MSB8 Thermotoga maritima MSB8 Thermotoga maritima MSB8 Thermotoga maritima MSB8 Thermotoga maritima Tma100 Thermotoga maritima Tma200 Thermotoga petrophila Thermotoga sp. RQ2 Thermotoga neapolitana Thermotoga naphthophila Thermotoga sp. 2812B Thermotoga sp. Cell2 Thermotoga sp. RQ7 Pseudothermotoga lettingae Pseudothermotoga thermarum Pseudothermotoga hypogea Thermosipho melanesiensis Thermosipho africanus Thermosipho sp. 1063 Thermosipho sp. 1070 Fervidobacterium nodosum Fervidobacterium pennivorans Fervidobacterium islandicum Petrotoga mobilis Marinitoga piezophila Marinitoga sp. 1137 Defluviitoga tunisiensis Kosmotoga olearia Kosmotoga pacifica Mesotoga prima Mesotoga infera Athalassotoga saccharophila Caldisericum exile Desulfurispirillum indicum Deferribacter desulfuricans Denitrovibrio acetiphilus Calditerrivibrio nitroreducens Flexistipes sinusarabici Geovibrio thiophilus Caldithrix abyssi Dictyoglomus thermophilum Dictyoglomus turgidum Thermodesulfovibrio yellowstonii Nitrospira defluvii Nitrospira moscoviensis Candidatus Nitrospira inopinata Nitrospira japonica Leptospirillum ferrooxidans Leptospirillum ferriphilum ML-04 Leptospirillum ferriphilum YSK Leptospirillum sp. Group II Thermodesulfatator indicus Thermodesulfobacterium geofontis Thermodesulfobacterium commune Thermodesulfobacterium sp. TA1 Caldimicrobium thiodismutans Thermosulfuriphilus ammonigenes Candidatus Saccharimonas aalborgensis Candidatus Saccharibacteria bacterium RAAC3\_TM7\_1 Candidatus Saccharibacteria bacterium GW2011\_GWC2\_44\_17 Candidatus Saccharibacteria oral Candidatus Peribacter riflensis Candidatus Bipolaricaulis anaerobius Candidatus Bipolaricaulis sp. Ch78 Vampirococcus sp. LiM Candidatus Methylomirabilis oxyfera Candidatus Chazhemtobacterium aquaticus Candidatus Babela massiliensis Candidate division TM6 bacterium GW2011\_GWF2\_28\_16 Candidate division SR1 bacterium RAAC1\_SR1\_1 Candidate division SR1 bacterium Aalborg\_AAW-1 Candidate division WWE3 bacterium RAAC2\_WWE3\_1 Candidate division Kazan bacterium GW2011\_GWA1\_50\_15 Berkelbacteria bacterium GW2011\_GWE1\_39\_12 Candidatus Beckwithbacteria bacterium GW2011\_GWC1\_49\_16 Candidatus Woesebacteria bacterium GW2011\_GWF1\_31\_35 Candidatus Wolfebacteria bacterium GW2011\_GWB1\_47\_1 Candidatus Campbellbacteria bacterium GW2011\_OD1\_34\_28 Methanocaldococcus jannaschii Methanocaldococcus fervens Methanocaldococcus vulcanius Methanocaldococcus sp. FS406-22 Methanocaldococcus infernus Methanocaldococcus bathoardescens Methanotorris igneus Methanococcus maripaludis S2 Methanococcus maripaludis C5 Methanococcus maripaludis C6 Methanococcus maripaludis C7 Methanococcus maripaludis X1 Methanococcus maripaludis KA1 Methanococcus maripaludis OS7 Methanococcus maripaludis DSM 2067 Methanococcus aeolicus Methanococcus vannielii Methanococcus voltae Methanothermococcus okinawensis Methanofervidicoccus sp. A16 Methanothermobacter thermautotrophicus Methanothermobacter marburgensis Methanothermobacter sp. CaT2 Methanothermobacter wolfeii Methanothermobacter sp. EMTCatA1 Methanothermobacter sp. MT-2 Methanothermobacter sp. KEPCO-1 Methanothermobacter sp. THM-1 Methanosphaera stadtmanae Methanosphaera sp. BMS Methanobrevibacter ruminantium Methanobrevibacter smithii Methanobrevibacter sp. AbM4 Methanobrevibacter millerae Methanobrevibacter sp. YE315 Methanobrevibacter olleyae Methanobacterium lacus Methanobacterium paludis Methanobacterium sp. MB1 Methanobacterium formicicum BRM9 Methanobacterium formicicum DSM1535 Methanobacterium congolense Methanobacterium subterraneum Methanobacterium sp. MZ-A1 Methanobacterium sp. BRmetb2 Methanobacterium sp. BAmetb5 Methanothermus fervidus Methanopyrus kandleri Archaeoglobus fulgidus DSM 4304 Archaeoglobus fulgidus DSM 8774 Archaeoglobus profundus Archaeoglobus veneficus Archaeoglobus sulfaticallidus Ferroglobus placidus Geoglobus acetivorans Geoglobus ahangari Thermoplasma acidophilum Thermoplasma volcanium Picrophilus torridus Ferroplasma acidarmanus Ferroplasma acidiphilum Cuniculiplasma divulgatum Thermoplasmatales archaeon BRNA1 Candidatus Methanomethylophilus alvus Candidatus Methanomassiliicoccus intestinalis Issoire-Mx1 Candidatus Methanoplasma termitum Methanogenic archaeon ISO4-H5 Aciduliprofundum boonei Aciduliprofundum sp. MAR08-339 Pyrococcus furiosus DSM 3638 Pyrococcus furiosus COM1 Pyrococcus horikoshii Pyrococcus abyssi Pyrococcus sp. NA2 Pyrococcus yayanosii Pyrococcus sp. ST04 Pyrococcus kukulkanii Thermococcus kodakarensis Thermococcus onnurineus Thermococcus gammatolerans Thermococcus sibiricus Thermococcus barophilus Thermococcus sp. 4557 Thermococcus sp. AM4 Thermococcus cleftensis Thermococcus litoralis Thermococcus paralvinellae Thermococcus nautili Thermococcus eurythermalis Thermococcus guaymasensis Thermococcus sp. 2319x1 Thermococcus chitonophagus Thermococcus peptonophilus Thermococcus piezophilus Thermococcus gorgonarius Thermococcus celer Thermococcus barossii Thermococcus sp. 5-4 Thermococcus siculi Thermococcus thioreducens Thermococcus profundus Thermococcus radiotolerans Thermococcus pacificus Thermococcus sp. P6 Palaeococcus pacificus Methanosarcina barkeri Fusaro Methanosarcina barkeri MS Methanosarcina barkeri Wiesmoor Methanosarcina barkeri 227 Methanosarcina barkeri 3 Methanosarcina acetivorans Methanosarcina mazei Go1 Methanosarcina mazei Tuc01 Methanosarcina mazei S-6 Methanosarcina mazei C16 Methanosarcina vacuolata Methanosarcina sp. Kolksee Methanosarcina lacustris Methanosarcina sp. MTP4 Methanosarcina sp. WH1 Methanosarcina sp. WWM596 Methanosarcina siciliae C2J Methanosarcina siciliae HI350 Methanosarcina siciliae T4/M Methanosarcina thermophila TM-1 Methanosarcina thermophila CHTI-55 Methanosarcina horonobensis Methanosarcina flavescens Methanococcoides burtonii Methanococcoides methylutens Methanohalophilus mahii Methanohalophilus halophilus Methanohalobium evestigatum Methanosalsum zhilinae Methanolobus psychrophilus Methanomethylovorans hollandica Methanothrix thermoacetophila Methanothrix soehngenii Methanosaeta harundinacea Methanospirillum hungatei Methanocorpusculum labreanum Methanoculleus marisnigri Methanoculleus bourgensis Methanoculleus sp. MAB1 Methanolacinia petrolearia Methanoregula boonei Methanoregula formicica Methanosphaerula palustris Methanocella paludicola Methanocella conradii Methanocella arvoryzae Halobacterium salinarum NRC-1 Halobacterium salinarum Halobacterium sp. DL1 Halobacterium hubeiense Halalkalicoccus jeotgali Halodesulfurarchaeum formicicum HTSR1 Haloarchaeon HSR6 Halanaeroarchaeum sulfurireducens HSR2 Halanaeroarchaeum sulfurireducens M27-SA2 Salarchaeum sp. JOR-1 Haloarcula marismortui Haloarcula hispanica ATCC 33960 Haloarcula hispanica N601 Haloarcula sp. CBA1115 Haloarcula taiwanensis Natronomonas pharaonis Natronomonas moolapensis Halorhabdus utahensis Halorhabdus tiamatea Halorhabdus sp. CBA1104 Halomicrobium mukohataei Halomicrobium mukohataei JP60 Halomicrobium sp. LC1Hm Halorientalis sp. IM1011 Halapricum salinum Haloarculaceae archaeon HArcel1 Haloquadratum walsbyi DSM 16790 Haloquadratum walsbyi C23 Haloferax volcanii Haloferax mediterranei Haloferax gibbonsii Haloferax alexandrinus Halogeometricum borinquense Haloplanus sp. CBA1112 Haloplanus sp. CBA1113 Haloplanus aerogenes Haloplanus rallus Halobellus limi Haloprofundus sp. MHR1 Halorubrum lacusprofundi Halorubrum sp. PV6 Halorubrum sp. BOL3-1 Halorubrum ezzemoulense Salinigranum rubrum Halohasta litchfieldiae Halalkaliarchaeum desulfuricum Halophilic archaeon DL31 Haloterrigena turkmenica Haloterrigena daqingensis Haloterrigena jeotgali Natrialba magadii Halopiger xanaduensis Natrinema sp. J7-2 Natrinema pellirubrum Natrinema versiforme Natrinema pallidum Natronobacterium gregoryi Halovivax ruber Natronococcus occultus Salinarchaeum sp. Harcht-Bsk1 Halostagnicola larsenii Halobiforma lacisalsi Natronolimnobius aegyptiacus Natronolimnobius sulfurireducens AArc-Mg Natronolimnobius sulfurireducens AArc1 Natronorubrum bangense Natronorubrum aibiense Nanohaloarchaea archaeon SG9 Aeropyrum pernix Aeropyrum camini Staphylothermus marinus Staphylothermus hellenicus Ignicoccus hospitalis Ignicoccus islandicus Desulfurococcus amylolyticus 1221n Desulfurococcus amylolyticus DSM 16532 Desulfurococcus mucosus Thermosphaera aggregans Ignisphaera aggregans Thermogladius calderae Hyperthermus butylicus Pyrolobus fumarii Pyrodictium delaneyi Sulfurisphaera tokodaii Sulfurisphaera ohwakuensis Saccharolobus solfataricus P2 Saccharolobus solfataricus 98/2 Saccharolobus solfataricus SULA Saccharolobus solfataricus SARC-B Saccharolobus solfataricus SARC-C Sulfolobus acidocaldarius DSM 639 Sulfolobus acidocaldarius N8 Sulfolobus acidocaldarius Ron12/I Sulfolobus acidocaldarius SUSAZ Sulfolobus islandicus L.S.2.15 Sulfolobus islandicus M.14.25 Sulfolobus islandicus M.16.27 Sulfolobus islandicus M.16.4 Sulfolobus islandicus Y.G.57.14 Sulfolobus islandicus Y.N.15.51 Sulfolobus islandicus L.D.8.5 Sulfolobus islandicus HVE10/4 Sulfolobus islandicus REY15A Sulfolobus islandicus LAL14/1 Sulfolobus sp. A20 Metallosphaera sedula Metallosphaera cuprina Metallosphaera hakonensis JCM 8857 = DSM 7519 Metallosphaera prunae Acidianus hospitalis Acidianus manzaensis Acidianus brierleyi Acidianus sulfidivorans Acidianus ambivalens Sulfodiicoccus acidiphilus Stygiolobus azoricus Sulfuracidifex tepidarius Pyrobaculum aerophilum Pyrobaculum islandicum Pyrobaculum calidifontis Pyrobaculum arsenaticum Pyrobaculum ferrireducens Pyrobaculum oguniense Pyrobaculum neutrophilum Pyrobaculum sp. WP30 Caldivirga maquilingensis Thermoproteus tenax Thermoproteus uzoniensis Vulcanisaeta distributa Vulcanisaeta moutnovskia Thermofilum pendens Thermofilum adornatus Thermofilum adornatus 1505 Thermofilum uzonense Acidilobus saccharovorans Acidilobus sp. 7A Caldisphaera lagunensis Fervidicoccus fontis Nitrosopumilus maritimus Candidatus Nitrosopumilus sediminis Candidatus Nitrosopumilus koreensis Nitrosopumilus piranensis Nitrosopumilus adriaticus Candidatus Nitrosopumilus sp. SW Candidatus Nitrosomarinus catalina Cenarchaeum symbiosum Candidatus Nitrososphaera gargensis Nitrososphaera viennensis Candidatus Nitrososphaera evergladensis Candidatus Nitrosocosmicus oleophilus Candidatus Nitrosocosmicus franklandus Candidatus Nitrosocaldus cavascurensis Candidatus Caldiarchaeum subterraneum Candidatus Nitrosopelagicus brevis Candidatus Nitrosotenuis cloacae Candidatus Nitrosotalea devanaterra Candidatus Nanopusillus acidilobi Candidatus Micrarchaeota archaeon Mia14 Candidatus Korarchaeum cryptofilum Candidatus Bathyarchaeota archaeon BA1 Candidatus Bathyarchaeota archaeon BA2 Lokiarchaeum sp. GC14\_75 Archaeon GW2011\_AR10 Archaeon GW2011\_AR20 | 184% 150% 122% 100% 82% 67% 55% |
