# Supplementary material for: Isolation, identification, and whole-genome sequencing of high-yield protease bacteria from Daqu of ZhangGong Laojiu
Source: PLoS One. 2022 Apr 26;17(4):e0264677. doi: 10.1371/journal.pone.0264677 (PMC9041807; doi:10.1371/journal.pone.0264677)
Supplement: S1 Raw data — (ZIP) [file pone.0264677.s002.zip › Raw data/GDD20120317-1_Bacillus_velezensis_Genome_result/4_Basic_Annot/KEGG/Bac_map/map00621.html]

KEGG PATHWAY: Dioxin degradation - Reference pathway


|  |  |
| --- | --- |
| **Dioxin degradation - Reference pathway** |  |

[
Pathway menu
| Organism menu
| Pathway entry
| Show description
| User data mapping
]

|  |
| --- |
| Dioxins and dioxin-like compounds, including PCDDs, PCDFs and PCBs, are persistent organic pollutants (POPs) that are resistant to environmental degradation. However, POPs can be degraded by certain microorganisms with acquisition of a novel set of enzymes, such as biphenyl degradation by Paraburkholderia xenovorans LB400 involving four novel enzymes encoded in operon-like structures [MD:M00543]. DDT, another POP, and carbazol are also known to be degraded in similar pathways. |

|  |  |
| --- | --- |
| Reference pathway Reference pathway (KO) Reference pathway (EC) Reference pathway (Reaction) -----< Sort below by alphabet >----- Escherichia coli K-12 MG1655 Escherichia coli K-12 W3110 Escherichia coli K-12 BW2952 Escherichia coli K-12 MDS42 Escherichia coli O157:H7 EDL933 (EHEC) Escherichia coli O157:H7 Sakai (EHEC) Escherichia coli O157:H7 EC4115 (EHEC) Escherichia coli O157:H7 TW14359 (EHEC) Escherichia coli O157:H7 Xuzhou21 (EHEC) Escherichia coli O111:H- 11128 (EHEC) Escherichia coli O26:H11 11368 (EHEC) Escherichia coli O103:H2 12009 (EHEC) Escherichia coli O145:H28 RM13514 (EHEC) Escherichia coli O145:H28 RM13516 (EHEC) Escherichia coli O104:H4 2011C-3493 (EAEC) Escherichia coli O104:H4 2009EL-2071 (EAEC) Escherichia coli O104:H4 2009EL-2050 (EAEC) Escherichia coli 55989 (EAEC) Escherichia coli O55:H7 CB9615 (EPEC) Escherichia coli O55:H7 RM12579 (EPEC) Escherichia coli O78:H11:K80 H10407 (ETEC) Escherichia coli O139:H28 E24377A (ETEC) Escherichia coli UMNK88 (ETEC, porcine) Escherichia coli NA114 (UPEC) Escherichia coli O25b:K100:H4-ST131 EC958 (UPEC) Escherichia coli APEC O78 (APEC) Escherichia coli O9 HS (commensal) Escherichia coli SMS-3-5 (environmental) Escherichia coli O152:H28 SE11 (commensal) Escherichia coli O8 IAI1 (commensal) Escherichia coli O81 ED1a (commensal) Escherichia coli O17:K52:H18 UMN026 (ExPEC) Escherichia coli O7:K1 IAI39 (ExPEC) Escherichia coli O7:K1 CE10 Escherichia coli B REL606 Escherichia coli BL21(DE3) Escherichia coli BL21(DE3) Escherichia coli BL21-Gold(DE3)pLysS AG Escherichia coli O44:H18 042 (EAEC) Escherichia coli O150:H5 SE15 (commensal) Escherichia coli ATCC 8739 Escherichia coli KO11FL Escherichia coli KO11FL Escherichia coli DH1 Escherichia coli DH1 Escherichia coli W Escherichia coli W Escherichia coli P12b Escherichia coli LY180 Escherichia coli JJ1886 Escherichia marmotae Escherichia sp. E4742 Shigella sonnei Ss046 Shigella sp. PAMC 28760 Klebsiella pneumoniae subsp. pneumoniae MGH 78578 (serotype K52) Klebsiella pneumoniae subsp. pneumoniae NTUH-K2044 (serotype K1) Klebsiella pneumoniae subsp. pneumoniae HS11286 Klebsiella pneumoniae subsp. pneumoniae 1084 (serotype K1) Klebsiella pneumoniae subsp. pneumoniae KPNIH24 Klebsiella pneumoniae subsp. pneumoniae KPNIH27 Klebsiella pneumoniae subsp. pneumoniae KPNIH29 Klebsiella pneumoniae subsp. pneumoniae KPNIH30 Klebsiella pneumoniae subsp. pneumoniae KPNIH31 Klebsiella pneumoniae subsp. pneumoniae KPNIH32 Klebsiella pneumoniae subsp. pneumoniae KPNIH10 Klebsiella pneumoniae subsp. pneumoniae KPR0928 Klebsiella pneumoniae subsp. pneumoniae ATCC 43816 KPPR1 Klebsiella pneumoniae 342 Klebsiella pneumoniae KCTC 2242 Klebsiella pneumoniae subsp. rhinoscleromatis SB3432 Klebsiella pneumoniae JM45 Klebsiella pneumoniae CG43 Klebsiella pneumoniae 30660/NJST258\_1 Klebsiella pneumoniae 30684/NJST258\_2 Klebsiella pneumoniae PMK1 Klebsiella pneumoniae blaNDM-1 Klebsiella pneumoniae 32192 Klebsiella pneumoniae 34618 Klebsiella pneumoniae Kp52.145 Klebsiella variicola At-22 Klebsiella variicola KP5-1 Klebsiella variicola DX120E Klebsiella variicola DSM 15968 Klebsiella michiganensis KCTC 1686 Klebsiella michiganensis E718 Klebsiella michiganensis HKOPL1 Klebsiella michiganensis M1 Klebsiella oxytoca KONIH1 Klebsiella oxytoca CAV1374 Klebsiella quasipneumoniae Klebsiella quasivariicola Klebsiella sp. LTGPAF-6F Klebsiella huaxiensis Citrobacter freundii Citrobacter werkmanii Citrobacter youngae Citrobacter portucalensis Citrobacter freundii complex sp. CFNIH3 Citrobacter sp. FDAARGOS\_156 Citrobacter sp. CRE-46 Citrobacter pasteurii Gibbsiella quercinecans Shimwellia blattae Raoultella ornithinolytica B6 Raoultella planticola Raoultella sp. X13 Raoultella terrigena Raoultella electrica Pluralibacter gergoviae Kluyvera georgiana Metakosakonia sp. MRY16-398 Izhakiella sp. KSNA2 Enterobacteriaceae bacterium FGI 57 Enterobacteriaceae bacterium ENNIH2 Enterobacteriaceae bacterium S05 Serratia proteamaculans Serratia liquefaciens Serratia sp. FS14 Serratia fonticola DSM 4576 Serratia fonticola GS2 Serratia sp. ATCC 39006 Serratia sp. ATCC 39006 Serratia quinivorans Pectobacterium atrosepticum SCRI1043 Pectobacterium atrosepticum JG10-08 Pectobacterium atrosepticum 21A Dickeya zeae EC1 Dickeya aquatica Photorhabdus laumondii subsp. laumondii TTO1 Photorhabdus laumondii subsp. laumondii DSPV002N Xenorhabdus nematophila ATCC 19061 Xenorhabdus nematophila AN6/1 Xenorhabdus hominickii Leminorella richardii Phytobacter ursingii Xanthomonas sacchari Xanthomonas perforans Xanthomonas arboricola Stenotrophomonas sp. LM091 Pseudoxanthomonas spadix Lysobacter antibioticus 76 Lysobacter antibioticus ATCC 29479 Lysobacter capsici Lysobacter gummosus Lysobacter sp. SJ-36 Lysobacter soli Rhodanobacter glycinis Aerosticca soli Vibrio natriegens Vibrio sp. EJY3 Vibrio furnissii Vibrio alfacsensis Pseudomonas resinovorans Pseudomonas pseudoalcaligenes Pseudomonas citronellolis Pseudomonas putida KT2440 Pseudomonas putida F1 Pseudomonas putida GB-1 Pseudomonas putida W619 Pseudomonas putida S16 Pseudomonas putida BIRD-1 Pseudomonas putida ND6 Pseudomonas putida DOT-T1E Pseudomonas putida HB3267 Pseudomonas putida H8234 Pseudomonas putida NBRC 14164 Pseudomonas monteilii SB3078 Pseudomonas monteilii SB3101 Pseudomonas soli Pseudomonas plecoglossicida Pseudomonas fluorescens A506 Pseudomonas fluorescens UK4 Pseudomonas corrugata Pseudomonas veronii Pseudomonas lurida Pseudomonas entomophila Pseudomonas stutzeri CCUG 29243 Pseudomonas stutzeri 19SMN4 Pseudomonas stutzeri 28a24 Pseudomonas balearica Pseudomonas brassicacearum subsp. brassicacearum NFM421 Pseudomonas sp. UW4 Pseudomonas sp. ATCC 13867 Pseudomonas sp. VLB120 Pseudomonas sp. TKP Pseudomonas knackmussii Pseudomonas chlororaphis PA23 Pseudomonas chlororaphis PCL1606 Pseudomonas fragi Pseudomonas lundensis Pseudomonas alkylphenolica Pseudomonas rhizosphaerae Pseudomonas sp. StFLB209 Pseudomonas versuta Pseudomonas sp. Os17 Pseudomonas koreensis Pseudomonas frederiksbergensis Pseudomonas sp. TCU-HL1 Pseudomonas silesiensis Pseudomonas kribbensis Azotobacter vinelandii DJ Azotobacter vinelandii CA Azotobacter vinelandii CA6 Azotobacter chroococcum Psychrobacter urativorans Acinetobacter lactucae Acinetobacter oleivorans Acinetobacter equi Acinetobacter johnsonii Acinetobacter schindleri Acinetobacter defluvii Acinetobacter radioresistens Acinetobacter wuhouensis Acinetobacter cumulans Acinetobacter lwoffii Moraxellaceae bacterium HYN0046 Shewanella denitrificans Shewanella pealeana Shewanella halifaxensis Shewanella woodyi Colwellia psychrerythraea Colwellia sp. PAMC 21821 Colwellia beringensis Thalassotalea sp. HSM 43 Pseudoalteromonas haloplanktis Pseudoalteromonas rubra Pseudoalteromonas translucida Pseudoalteromonas nigrifaciens Marinobacter adhaerens Marinobacter salarius Marinobacter sp. LQ44 Marinobacter salinus Marinobacter sp. JH2 Alteromonas mediterranea MED64 Lacimicrobium alkaliphilum Catenovulum sp. CCB-QB4 Saliniradius amylolyticus Hydrocarboniclastica marina Moritella marina Cellvibrio japonicus Teredinibacter turnerae Spongiibacter sp. IMCC21906 Zhongshania aliphaticivorans Halioglobus japonicus Halioglobus sp. IMCC14385 Kineobactrum sp. M2 Tatlockia micdadei Cycloclasticus sp. P1 Cycloclasticus zancles Cycloclasticus sp. PY97N Beggiatoa leptomitiformis Marichromatium purpuratum Alkalilimnicola ehrlichii Halomonas campaniensis Halomonas sp. KO116 Halomonas sp. 1513 Halomonas hydrothermalis Halomonas beimenensis Halomonas alkaliphila Halomonas venusta Alcanivorax pacificus Alcanivorax xenomutans Ketobacter alkanivorans Marinomonas sp. MWYL1 Marinomonas posidonica Oleispira antarctica Marinobacterium aestuarii Neptunomonas concharum Nitrincola sp. KXZD1103 Reinekea forsetii Oceanimonas sp. GK1 Oceanisphaera avium Oceanisphaera profunda Zobellella denitrificans Solimonas sp. K1W22B-7 Sinimarinibacterium sp. NLF-5-8 Immundisolibacter cernigliae Thiolapillus brandeum Gamma proteobacterium HdN1 Vitreoscilla filiformis Chromobacterium sp. ATCC 53434 Chromobacterium rhizoryzae Pseudogulbenkiania sp. NH8B Aquaspirillum sp. LM1 Aquitalea sp. USM4 Ralstonia solanacearum GMI1000 Ralstonia solanacearum CMR15 Ralstonia solanacearum FQY\_4 Ralstonia pickettii 12J Ralstonia pickettii 12D Ralstonia pickettii DTP0602 Ralstonia insidiosa Ralstonia pseudosolanacearum Cupriavidus necator H16 Cupriavidus necator N-1 Cupriavidus necator NH9 Cupriavidus pinatubonensis JMP134 Cupriavidus metallidurans Cupriavidus taiwanensis Cupriavidus basilensis Cupriavidus sp. USMAHM13 Cupriavidus malaysiensis Cupriavidus sp. USMAA2-4 Cupriavidus oxalaticus Burkholderia pseudomallei K96243 Burkholderia pseudomallei 1710b Burkholderia pseudomallei 1106a Burkholderia pseudomallei 668 Burkholderia pseudomallei MSHR305 Burkholderia pseudomallei MSHR511 Burkholderia pseudomallei MSHR146 Burkholderia pseudomallei MSHR520 Burkholderia pseudomallei 1026b Burkholderia pseudomallei BPC006 Burkholderia pseudomallei NCTC 13179 Burkholderia pseudomallei HBPUB10134a Burkholderia pseudomallei NAU35A-3 Burkholderia pseudomallei A79A Burkholderia pseudomallei TSV202 Burkholderia thailandensis E264 Burkholderia thailandensis 2002721723 Burkholderia thailandensis E444 Burkholderia thailandensis H0587 Burkholderia thailandensis MSMB121 Burkholderia thailandensis MSMB59 Burkholderia thailandensis E254 Burkholderia thailandensis USAMRU Malaysia #20 Burkholderia thailandensis 2003015869 Burkholderia thailandensis 2002721643 Burkholderia oklahomensis E0147 Burkholderia oklahomensis C6786 Burkholderia sp. BDU6 Burkholderia vietnamiensis G4 Burkholderia vietnamiensis LMG 10929 Burkholderia lata Burkholderia cenocepacia AU1054 Burkholderia cenocepacia HI2424 Burkholderia cenocepacia MC0-3 Burkholderia cenocepacia J2315 Burkholderia cenocepacia DDS 22E-1 Burkholderia cenocepacia DWS 37E-2 Burkholderia cenocepacia H111 Burkholderia ambifaria AMMD Burkholderia ambifaria MC40-6 Burkholderia multivorans ATCC 17616 (Tohoku) Burkholderia multivorans ATCC 17616 (JGI) Burkholderia multivorans DDS 15A-1 Burkholderia multivorans ATCC BAA-247 Burkholderia cepacia GG4 Burkholderia cepacia DDS 7H-2 Burkholderia cepacia ATCC 25416 Burkholderia dolosa Burkholderia pyrrocinia Burkholderia contaminans Burkholderia ubonensis Burkholderia diffusa Burkholderia latens Burkholderia territorii Burkholderia seminalis Burkholderia pseudomultivorans Burkholderia metallica Burkholderia stagnalis Burkholderia stabilis Burkholderia glumae BGR1 Burkholderia glumae LMG 2196 = ATCC 33617 Burkholderia sp. CCGE1001 Burkholderia sp. CCGE1003 Burkholderia gladioli BSR3 Burkholderia gladioli ATCC 10248 Burkholderia sp. YI23 Burkholderia sp. KJ006 Burkholderia insecticola Burkholderia sp. RPE67 Burkholderia sp. 2002721687 Burkholderia sp. HB1 Burkholderia plantarii PG1 Burkholderia plantarii ATCC 43733 Burkholderia sp. Bp5365 Burkholderia sp. OLGA172 Burkholderia sp. PAMC 26561 Burkholderia sp. PAMC 28687 Paraburkholderia xenovorans LB400 Paraburkholderia xenovorans LB400 Paraburkholderia phymatum Paraburkholderia sp. CCGE1002 Paraburkholderia phenoliruptrix Paraburkholderia phytofirmans Paraburkholderia fungorum Paraburkholderia caribensis Paraburkholderia sprentiae Paraburkholderia sp. SOS3 Paraburkholderia aromaticivorans Paraburkholderia hospita Paraburkholderia terrae Paraburkholderia graminis Paraburkholderia caledonica Paraburkholderia terricola Paraburkholderia caffeinilytica Paraburkholderia rhizoxinica Pandoraea pnomenusa 3kgm Pandoraea pnomenusa RB38 Pandoraea pnomenusa DSM 16536 Pandoraea pnomenusa Pandoraea pulmonicola Pandoraea sputorum Pandoraea apista Pandoraea vervacti Pandoraea oxalativorans Pandoraea thiooxydans Pandoraea faecigallinarum Pandoraea norimbergensis Pandoraea sp. XY-2 Pandoraea fibrosis Caballeronia sp. SBC2 Bordetella pertussis 18323 Bordetella parapertussis Bpp5 Bordetella parapertussis 12822 Bordetella bronchiseptica 253 Bordetella bronchiseptica RB50 Bordetella bronchiseptica MO149 Bordetella bronchiseptica S798 Bordetella avium Bordetella hinzii Bordetella bronchialis Bordetella flabilis Bordetella pseudohinzii Bordetella sp. H567 Bordetella genomosp. 13 Bordetella sp. HZ20 Bordetella sp. J329 Achromobacter xylosoxidans A8 Achromobacter insolitus Achromobacter spanius Achromobacter sp. AONIH1 Achromobacter sp. B7 Pusillimonas sp. T7-7 Pusillimonas sp. ye3 Advenella kashmirensis Advenella mimigardefordensis Castellaniella defragrans Alcaligenes faecalis ZD02 Alcaligenes faecalis JQ135 Alcaligenes aquatilis Paenalcaligenes hominis Pigmentiphaga sp. H8 Kerstersia gyiorum Rhodoferax ferrireducens Rhodoferax saidenbachensis Rhodoferax koreense Rhodoferax sp. CHu59-6-5 Rhodoferax sp. Gr-4 Polaromonas sp. JS666 Polaromonas naphthalenivorans Polaromonas sp. SP1 Polaromonas sp. Pch-P Acidovorax citrulli Acidovorax sp. JS42 Acidovorax ebreus Acidovorax avenae Acidovorax sp. KKS102 Acidovorax sp. RAC01 Acidovorax carolinensis NA2 Acidovorax carolinensis P4 Acidovorax carolinensis NA3 Acidovorax carolinensis P3 Acidovorax sp. 1608163 Verminephrobacter eiseniae Delftia acidovorans Delftia sp. Cs1-4 Delftia tsuruhatensis Delftia sp. HK171 Variovorax paradoxus S110 Variovorax paradoxus EPS Variovorax paradoxus B4 Variovorax boronicumulans Variovorax sp. PMC12 Comamonas thiooxydans Comamonas testosteroni TK102 Comamonas kerstersii Comamonas serinivorans Comamonas sp. NLF 7-7 Alicycliphilus denitrificans BC Alicycliphilus denitrificans K601 Ramlibacter tataouinensis Ottowia oryzae Limnohabitans sp. 63ED37-2 Hydrogenophaga sp. RAC07 Hydrogenophaga sp. PBC Hydrogenophaga sp. LPB0072 Hydrogenophaga sp. PAMC20947 Hydrogenophaga pseudoflava Hydrogenophaga sp. BPS33 Diaphorobacter polyhydroxybutyrativorans Simplicispira suum Pulveribacter suum Melaminivora sp. SC2-9 Methylibium petroleiphilum Methylibium sp. Pch-M Herminiimonas arsenicoxydans Janthinobacterium sp. Marseille Janthinobacterium agaricidamnosum Janthinobacterium sp. 17J80-10 Herbaspirillum hiltneri Herbaspirillum rubrisubalbicans Herbaspirillum sp. meg3 Collimonas fungivorans Collimonas arenae Collimonas pratensis Massilia sp. WG5 Leptothrix cholodnii Roseateles depolymerans Paucibacter sp. KCTC 42545 Mitsuaria sp. 7 Rhizobacter gummiphilus Sphaerotilus natans Xylophilus sp. KACC 21265 Polyangium brachysporum Rugosibacter aromaticivorans Oryzomicrobium terrae Dechloromonas aromatica Dechloromonas sp. HYN0024 Azoarcus sp. BH72 Azoarcus sp. CIB Azoarcus olearius Azoarcus sp. SY39 Azoarcus communis Azoarcus sp. DN11 Azoarcus sp. DD4 Thauera sp. MZ1T Thauera humireducens Thauera sp. K11 Campylobacter jejuni subsp. doylei 269.97 Campylobacter lari CCUG 22395 Campylobacter coli RM1875 Arcobacter butzleri RM4018 Arcobacter butzleri ED-1 Arcobacter butzleri 7h1h Arcobacter nitrofigilis Arcobacter skirrowii Arcobacter halophilus Arcobacter marinus Arcobacter trophiarum Arcobacter porcinus Halarcobacter bivalviorum Arcobacter sp. L Arcobacter sp. LPB0137 Sulfurospirillum halorespirans Pelobacter sp. SFB93 Desulfovibrio magneticus Pseudodesulfovibrio piezophilus Myxococcus stipitatus Corallococcus coralloides Desulfomonile tiedjei Mesorhizobium japonicum MAFF 303099 Mesorhizobium loti NZP2037 Mesorhizobium ciceri (biovar Biserrulae) Mesorhizobium opportunistum Mesorhizobium australicum Mesorhizobium amorphae Mesorhizobium sp. B7 Mesorhizobium sp. WSM1497 Mesorhizobium sp. M9A.F.Ca.ET.002.03.1.2 Mesorhizobium sp. Pch-S Mesorhizobium huakuii Chelativorans sp. BNC1 Hoeflea sp. IMCC20628 Aminobacter aminovorans Aminobacter sp. MSH1 Phyllobacterium zundukense Sinorhizobium meliloti 1021 Sinorhizobium meliloti AK83 Sinorhizobium meliloti BL225C Sinorhizobium meliloti SM11 Sinorhizobium meliloti Rm41 Sinorhizobium meliloti GR4 Sinorhizobium meliloti 2011 Sinorhizobium meliloti RMO17 Sinorhizobium medicae Sinorhizobium fredii NGR234 Sinorhizobium fredii HH103 Sinorhizobium fredii USDA 257 Sinorhizobium sp. RAC02 Sinorhizobium americanum Sinorhizobium sp. CCBAU 05631 Ensifer adhaerens OV14 Ensifer adhaerens Casida A Ensifer alkalisoli Agrobacterium fabrum Agrobacterium radiobacter Agrobacterium tumefaciens S33 Agrobacterium sp. H13-3 Agrobacterium sp. RAC06 Agrobacterium rhizogenes Agrobacterium sp. 33MFTa1.1 Rhizobium etli CFN 42 Rhizobium etli CIAT 652 Rhizobium etli bv. mimosae Mim1 Rhizobium leguminosarum bv. viciae 3841 Rhizobium leguminosarum bv. trifolii WSM2304 Rhizobium leguminosarum bv. trifolii WSM1325 Rhizobium leguminosarum bv. trifolii WSM1689 Rhizobium tropici Rhizobium sp. IRBG74 Rhizobium favelukesii Rhizobium gallicum Rhizobium sp. NT-26 Rhizobium sp. S41 Rhizobium jaguaris Rhizobium sp. 11515TR Rhizobium grahamii Rhizobium acidisoli Neorhizobium galegae bv. officinalis bv. officinalis HAMBI 1141 Neorhizobium galegae bv. orientalis HAMBI 540 Neorhizobium sp. SOG26 Neorhizobium sp. NCHU2750 Shinella sp. HZN7 Georhizobium profundi Ochrobactrum anthropi ATCC 49188 Bradyrhizobium diazoefficiens USDA 110 Bradyrhizobium japonicum USDA 6 Bradyrhizobium japonicum E109 Bradyrhizobium sp. ORS 278 Bradyrhizobium sp. BTAi1 Bradyrhizobium sp. S23321 Bradyrhizobium oligotrophicum Bradyrhizobium sp. CCGE-LA001 Bradyrhizobium sp. BF49 Bradyrhizobium icense Bradyrhizobium sp. ORS 285 Bradyrhizobium sp. SK17 Bradyrhizobium ottawaense Bradyrhizobium amphicarpaeae Bradyrhizobium guangdongense Bradyrhizobium guangzhouense Bradyrhizobium symbiodeficiens Bradyrhizobium betae Rhodopseudomonas palustris CGA009 Rhodopseudomonas palustris HaA2 Rhodopseudomonas palustris BisB18 Rhodopseudomonas palustris BisB5 Rhodopseudomonas palustris BisA53 Rhodopseudomonas palustris TIE-1 Rhodopseudomonas palustris DX-1 Oligotropha carboxidovorans OM5 (Mississippi) Oligotropha carboxidovorans OM5 (Goettingen) Oligotropha carboxidovorans OM4 Bosea sp. PAMC 26642 Bosea sp. RAC05 Bosea vaviloviae Bosea sp. Tri-49 Bosea sp. F3-2 Variibacter gotjawalensis Bartonella tribocorum CIP 105476 Bartonella tribocorum BM1374166 Bartonella grahamii Bartonella australis Bartonella apis Bartonella elizabethae Xanthobacter autotrophicus Azorhizobium caulinodans Starkeya novella Labrys neptuniae Ancylobacter sp. TS-1 Ancylobacter pratisalsi Methylorubrum extorquens DM4 Methylorubrum extorquens PA1 Methylorubrum extorquens CM4 Methylorubrum populi Methylorubrum zatmanii Methylobacterium radiotolerans Methylobacterium sp. 4-46 Methylobacterium nodulans Methylobacterium aquaticum Methylobacterium currus Methylobacterium sp. DM1 Methylobacterium sp. 17SD2-17 Methylobacterium sp. 17Sr1-43 Methylobacterium mesophilicum Methylobacterium terrae Microvirga ossetica Beijerinckia indica Methylocella silvestris Methylocella tundrae Beijerinckiaceae bacterium RH AL1 Chelatococcus sp. CO-6 Chelatococcus daeguensis Hyphomicrobium sp. MC1 Rhodomicrobium vannielii Pelagibacterium halotolerans Devosia sp. H5989 Devosia ginsengisoli Blastochloris viridis Rhodoplanes sp. Z2-YC6860 Maritalea myrionectae Youhaiella tibetensis Methylocystis bryophila Methylocystis parvus Methylosinus trichosporium Pleomorphomonas sp. SM30 Martelella endophytica Martelella sp. AD-3 Martelella mediterranea Breoghania sp. L-A4 Pseudorhodoplanes sinuspersici Hartmannibacter diazotrophicus Nordella sp. HKS 07 Caulobacter vibrioides CB15 Caulobacter vibrioides NA1000 Caulobacter mirabilis Caulobacter flavus Caulobacter sp. FWC26 Caulobacteraceae bacterium Ruegeria pomeroyi Ruegeria sp. TM1040 Epibacterium mobile Roseobacter denitrificans Paracoccus denitrificans Paracoccus aminophilus Paracoccus contaminans Paracoccus sp. BM15 Paracoccus aminovorans Paracoccus sp. SC2-6 Paracoccus kondratievae Dinoroseobacter shibae Ketogulonicigenium vulgare WSH-001 Ketogulonicigenium vulgare Y25 Ketogulonicigenium robustum Pseudovibrio sp. FO-BEG1 Phaeobacter inhibens 2.10 Phaeobacter gallaeciensis DSM 26640 Phaeobacter porticola Phaeobacter piscinae Phaeobacter sp. LSS9 Octadecabacter arcticus Octadecabacter sp. SW4 Leisingera methylohalidivorans Leisingera sp. NJS204 Leisingera aquaemixtae Celeribacter indicus Celeribacter ethanolicus Marinovum algicola Confluentimicrobium sp. EMB200-NS6 Pannonibacter phragmitetus Halocynthiibacter arcticus Labrenzia sp. CP4 Labrenzia aggregata Labrenzia sp. VG12 Labrenzia sp. PHM005 Labrenzia sp. THAF35 Defluviimonas alba Sulfitobacter sp. AM1-D1 Sulfitobacter sp. SK012 Sulfitobacter sp. JL08 Pelagibaca abyssi Roseovarius mucosus Roseovarius sp. AK1035 Roseovarius indicus Roseovarius sp. MME-070 Antarctobacter heliothermus Rhodobaca barguzinensis Sagittula sp. P11 Thalassococcus sp. S3 Sedimentitalea sp. W43 Litoreibacter sp. LN3S51 Paraoceanicella profunda Stappia indica Halovulum dunhuangense Pseudohalocynthiibacter aestuariivivens Rhodobacteraceae bacterium SH-1 Hirschia baltica Hyphomonadaceae bacterium UKL13-1 Novosphingobium aromaticivorans Novosphingobium sp. PP1Y Novosphingobium pentaromativorans Novosphingobium resinovorum Novosphingobium sp. P6W Novosphingobium sp. THN1 Novosphingobium tardaugens Novosphingobium sp. Gsoil 351 Sphingopyxis alaskensis Sphingopyxis fribergensis Sphingopyxis macrogoltabida EY-1 Sphingopyxis macrogoltabida 203 Sphingopyxis terrae Sphingopyxis granuli Sphingopyxis sp. QXT-31 Sphingopyxis sp. MG Sphingopyxis sp. PAMC25046 Uncultured Sphingopyxis sp. UC10 Sphingomonas wittichii Sphingomonas wittichii DC-6 Sphingomonas sp. MM-1 Sphingomonas taxi Sphingomonas hengshuiensis Sphingomonas sanxanigenens Sphingomonas sp. NIC1 Sphingomonas melonis Sphingomonas panacis Sphingomonas sp. KC8 Sphingomonas sp. FARSPH Sphingomonas sp. YZ-8 Sphingomonas paucimobilis Sphingobium japonicum Sphingobium chlorophenolicum Sphingobium sp. SYK-6 Sphingobium sp. YBL2 Sphingobium baderi Sphingobium sp. EP60837 Sphingobium sp. RAC03 Sphingobium indicum Sphingobium sp. TKS Sphingobium hydrophobicum Sphingobium yanoikuyae Sphingobium fuliginis Sphingorhabdus sp. SMR4y Sphingorhabdus sp. YGSMI21 Blastomonas sp. RAC04 Blastomonas fulva Rhizorhabdus dicambivorans Sphingosinicella sp. BN140058 Tardibacter chloracetimidivorans Erythrobacter atlanticus Erythrobacter sp. KY5 Altererythrobacter epoxidivorans Altererythrobacter namhicola Altererythrobacter sp. BO-6 Croceicoccus naphthovorans Croceicoccus marinus Porphyrobacter sp. HT-58-2 Gluconacetobacter diazotrophicus PA1 5 (Brazil) Gluconacetobacter diazotrophicus PA1 5 (JGI) Komagataeibacter saccharivorans Roseomonas gilardii Roseomonas sp. FDAARGOS\_362 Roseomonas mucosa Stella humosa Stella vacuolata Neokomagataea tanensis Azospirillum sp. B510 Azospirillum lipoferum Azospirillum brasilense Sp245 Azospirillum brasilense Az39 Azospirillum brasilense Sp7 Azospirillum thiophilum Azospirillum humicireducens Azospirillum sp. TSH58 Azospirillum sp. M2T2B2 Tistrella mobilis Thalassospira xiamenensis Magnetospira sp. QH-2 Nitrospirillum amazonense Niveispirillum cyanobacteriorum Ferrovibrio terrae Hypericibacter terrae Hypericibacter adhaerens Candidatus Pelagibacter ubique Candidatus Pelagibacter sp. IMCC9063 Alpha proteobacterium HIMB5 Polymorphum gilvum Phreatobacter stygius Hydrogenophilus thermoluteolus Bacillus subtilis subsp. spizizenii W23 Bacillus licheniformis ATCC 14580 Bacillus licheniformis DSM 13 = ATCC 14580 Bacillus paralicheniformis Bacillus cereus ATCC 10987 Bacillus cereus 03BB102 Bacillus cereus F837/76 Bacillus cereus FRI-35 Bacillus cereus FT9 Bacillus thuringiensis Al Hakam Bacillus pseudomycoides 219298 Bacillus pseudofirmus Bacillus megaterium QM B1551 Bacillus megaterium DSM 319 Bacillus megaterium WSH-002 Bacillus megaterium NBRC 15308 = ATCC 14581 Bacillus coagulans 2-6 Bacillus coagulans 36D1 Bacillus sp. 1NLA3E Bacillus sp. X1(2014) Bacillus sp. OxB-1 Bacillus filamentosus Bacillus smithii Bacillus simplex Bacillus glycinifermentans Bacillus horikoshii Bacillus krulwichiae Bacillus kochii Bacillus muralis Bacillus intestinalis Bacillus sp. Y1 Oceanobacillus sp. 160 Oceanobacillus sp. 143 Geobacillus thermodenitrificans Geobacillus sp. Y412MC61 Geobacillus sp. Y412MC52 Geobacillus sp. C56-T3 Geobacillus sp. Y4.1MC1 Geobacillus sp. GHH01 Geobacillus genomosp. 3 Geobacillus sp. JS12 Parageobacillus thermoglucosidasius C56-YS93 Parageobacillus thermoglucosidasius DSM 2542 Lysinibacillus varians Lysinibacillus sp. B2A1 Lysinibacillus sp. 2017 Lysinibacillus sp. SGAir0095 Lysinibacillus pakistanensis Terribacillus goriensis Virgibacillus sp. 6R Virgibacillus phasianinus Lentibacillus amyloliquefaciens Fictibacillus phosphorivorans Fictibacillus arsenicus Aeribacillus pallidus Pradoshia sp. D12 Salicibibacter kimchii Salicibibacter halophilus Staphylococcus epidermidis ATCC 12228 Staphylococcus epidermidis PM221 Staphylococcus haemolyticus Sh29/312/L2 Staphylococcus carnosus Staphylococcus lugdunensis HKU09-01 Staphylococcus lugdunensis N920143 Staphylococcus capitis Staphylococcus simulans Staphylococcus condimenti Staphylococcus pettenkoferi Staphylococcus hominis Staphylococcus muscae Staphylococcus caprae Brevibacillus brevis Brevibacillus formosus Paenibacillus sp. JDR-2 Paenibacillus sp. Y412MC10 Paenibacillus mucilaginosus KNP414 Paenibacillus mucilaginosus 3016 Paenibacillus mucilaginosus K02 Paenibacillus sp. FSL R7-0331 Paenibacillus beijingensis Paenibacillus naphthalenovorans Paenibacillus bovis Paenibacillus xylanexedens Paenibacillus donghaensis Paenibacillus vortex Paenibacillus lautus Paenibacillus psychroresistens Aneurinibacillus sp. XH2 Aneurinibacillus soli Cohnella candidum Cohnella sp. HS21 Alicyclobacillus acidocaldarius subsp. acidocaldarius DSM 446 Alicyclobacillus acidocaldarius subsp. acidocaldarius Tc-4-1 Kyrpidia tusciae Kyrpidia spormannii Solibacillus silvestris StLB046 Solibacillus silvestris DSM 12223 Solibacillus sp. R5-41 Planococcus rifietoensis Planococcus versutus Planococcus maritimus Planococcus plakortidis Planococcus sp. MB-3u-03 Kurthia sp. 11kri321 Kurthia zopfii Sporosarcina psychrophila Sporosarcina pasteurii Planomicrobium sp. Y50 Lactobacillus reuteri DSM 20016 Lactobacillus reuteri JCM 1112 Lactobacillus reuteri SD2112 Lactobacillus reuteri TD1 Lactobacillus fermentum IFO 3956 Lactobacillus fermentum F-6 Lactobacillus mucosae Enterococcus faecium Aus0004 Leuconostoc kimchii Leuconostoc sp. C2 Leuconostoc pseudomesenteroides Weissella soli Carnobacterium sp. 17-4 Carnobacterium sp. CP1 Clostridium sp. BNL1100 Clostridium pasteurianum BC1 Clostridium aceticum Clostridium formicaceticum Geosporobacter ferrireducens Hungateiclostridium clariflavum Flavonifractor plautii Lachnoclostridium sp. YL32 Clostridium cf. saccharolyticum K10 Desulfitobacterium hafniense DCB-2 Desulfotomaculum nigrificans Desulfofundulus kuznetsovii Pelotomaculum thermopropionicum Desulfosporosinus orientis Dehalobacter sp. DCA Dehalobacter sp. CF Dehalobacter restrictus Thermaerobacter marianensis Sulfobacillus acidophilus TPY Sulfobacillus acidophilus DSM 10332 Carboxydothermus hydrogenoformans Moorella thermoacetica ATCC 39073 Moorella thermoacetica DSM 521 Moorella thermoacetica DSM 2955 Thermosediminibacter oceani Sporanaerobacter sp. NJN-17 Megasphaera hexanoica Sporomusa termitida Limnochorda pilosa Mycobacterium tuberculosis H37Rv Mycobacterium tuberculosis H37Rv Mycobacterium tuberculosis CDC1551 Mycobacterium tuberculosis H37Ra Mycobacterium tuberculosis F11 Mycobacterium tuberculosis KZN 1435 Mycobacterium tuberculosis KZN 4207 Mycobacterium tuberculosis KZN 605 Mycobacterium tuberculosis RGTB327 Mycobacterium tuberculosis RGTB423 Mycobacterium tuberculosis CCDC5079 Mycobacterium tuberculosis CCDC5079 Mycobacterium tuberculosis CCDC5180 Mycobacterium tuberculosis CTRI-2 Mycobacterium tuberculosis UT205 Mycobacterium tuberculosis Erdman = ATCC 35801 Mycobacterium tuberculosis Beijing/NITR203 Mycobacterium tuberculosis 7199-99 Mycobacterium tuberculosis CAS/NITR204 Mycobacterium tuberculosis EAI5/NITR206 Mycobacterium tuberculosis EAI5 Mycobacterium tuberculosis Haarlem/NITR202 Mycobacterium tuberculosis Haarlem Mycobacterium tuberculosis BT1 Mycobacterium tuberculosis BT2 Mycobacterium tuberculosis HKBS1 Mycobacterium tuberculosis variant bovis AF2122/97 Mycobacterium tuberculosis variant bovis BCG Pasteur 1173P2 Mycobacterium tuberculosis variant bovis BCG Tokyo 172 Mycobacterium tuberculosis variant bovis BCG Mexico Mycobacterium tuberculosis variant bovis BCG Korea 1168P Mycobacterium tuberculosis variant bovis BCG ATCC 35743 Mycobacterium tuberculosis variant africanum Mycobacterium tuberculosis variant microti Mycobacterium canettii CIPT 140010059 Mycobacterium canettii CIPT 140060008 Mycobacterium canettii CIPT 140070008 Mycobacterium canettii CIPT 140070010 Mycobacterium canettii CIPT 140070017 Mycobacterium avium subsp. paratuberculosis K-10 Mycobacterium avium subsp. paratuberculosis MAP4 Mycobacterium avium subsp. paratuberculosis E1 Mycobacterium avium subsp. paratuberculosis E93 Mycobacterium avium 104 Mycobacterium intracellulare MOTT-02 Mycobacterium intracellulare ATCC 13950 Mycobacterium intracellulare subsp. intracellulare Mycobacterium intracellulare subsp. yongonense Mycobacterium paraintracellulare Mycobacterium chimaera Mycobacterium marseillense Mycobacterium lepraemurium Mycobacterium sp. JS623 Mycobacterium ulcerans Mycobacterium sp. MCS Mycobacterium sp. KMS Mycobacterium sp. JLS Mycobacterium marinum M Mycobacterium marinum E11 Mycobacterium sp. MOTT36Y Mycobacterium liflandii Mycobacterium kansasii ATCC 12478 Mycobacterium sp. VKM Ac-1817D Mycobacterium sp. EPa45 Mycobacterium haemophilum Mycobacterium sp. NRRL B-3805 Mycobacterium dioxanotrophicus Mycobacterium shigaense Mycolicibacterium smegmatis MC2 155 Mycolicibacterium smegmatis MC2 155 Mycolicibacterium smegmatis MC2 155 Mycolicibacterium smegmatis INHR1 Mycolicibacterium smegmatis INHR2 Mycolicibacterium vanbaalenii Mycolicibacterium gilvum PYR-GCK Mycolicibacterium gilvum Spyr1 Mycolicibacterium chubuense Mycolicibacterium neoaurum Mycolicibacterium goodii Mycolicibacterium fortuitum Mycolicibacterium phlei Mycolicibacterium vaccae Mycolicibacterium litorale Mycolicibacterium rhodesiae Mycolicibacterium thermoresistibile Mycolicibacterium hassiacum Mycobacteroides abscessus ATCC 19977 Mycobacteroides abscessus subsp. massiliense GO 06 Mycobacteroides abscessus subsp. bolletii 50594 Mycobacteroides abscessus subsp. massiliense CCUG 48898 = JCM 15300 Mycobacteroides chelonae Mycobacteroides immunogenum Mycobacterium stephanolepidis Mycobacteroides saopaulense Mycobacteroides salmoniphilum Mycolicibacter sinensis Mycolicibacter terrae Hoyosella subflava Mycolicibacillus koreensis Corynebacterium diphtheriae HC03 Corynebacterium halotolerans Corynebacterium argentoratense Corynebacterium glyciniphilum Corynebacterium imitans Corynebacterium mustelae Corynebacterium ammoniagenes Nocardia farcinica IFM 10152 Nocardia farcinica NCTC11134 Nocardia cyriacigeorgica Nocardia brasiliensis Nocardia nova Nocardia mangyaensis Nocardia seriolae Nocardia terpenica Nocardia sp. CS682 Nocardia otitidiscaviarum Rhodococcus jostii Rhodococcus erythropolis PR4 Rhodococcus erythropolis CCM2595 Rhodococcus erythropolis BG43 Rhodococcus opacus B4 Rhodococcus opacus PD630 Rhodococcus hoagii Rhodococcus pyridinivorans Rhodococcus aetherivorans Rhodococcus sp. WMMA185 Rhodococcus sp. PBTS 2 Rhodococcus ruber Rhodococcus qingshengii Rhodococcus sp. p52 Rhodococcus sp. 008 Rhodococcus rhodochrous Rhodococcus biphenylivorans Gordonia bronchialis Gordonia polyisoprenivorans Gordonia sp. KTR9 Gordonia phthalatica Gordonia terrae Gordonia sp. YC-JH1 Gordonia iterans Gordonia rubripertincta Gordonia sp. MMS17-SY073 Gordonia alkanivorans Gordonia sp. 135 Tsukamurella paurometabola Tsukamurella tyrosinosolvens Segniliparus rotundus Dietzia timorensis Dietzia sp. oral taxon 368 Dietzia sp. JS16-p6b Dietzia psychralcaliphila Dietzia lutea Tomitella sp. HY188 Streptomyces coelicolor Streptomyces griseus Streptomyces scabiei Streptomyces sp. SirexAA-E Streptomyces violaceusniger Streptomyces bingchenggensis Streptomyces davaonensis Streptomyces albus DSM 41398 Streptomyces collinus Streptomyces rapamycinicus Streptomyces albulus NK660 Streptomyces albulus ZPM Streptomyces lividans Streptomyces vietnamensis Streptomyces sp. 769 Streptomyces lydicus A02 Streptomyces lydicus 103 Streptomyces sp. Mg1 Streptomyces pristinaespiralis Streptomyces sp. CFMR 7 Streptomyces sp. 4F Streptomyces rubrolavendulae Streptomyces parvulus Streptomyces sp. SAT1 Streptomyces griseochromogenes Streptomyces qaidamensis Streptomyces lincolnensis Streptomyces pluripotens Streptomyces niveus Streptomyces autolyticus Streptomyces violaceoruber Streptomyces fodineus Streptomyces gilvosporeus Streptomyces malaysiensis Streptomyces laurentii Streptomyces lavendulae Streptomyces sp. MOE7 Streptomyces griseorubiginosus Streptomyces rochei Streptomyces koyangensis Kitasatospora setae Kitasatospora aureofaciens Kitasatospora sp. MMS16-BH015 Streptacidiphilus sp. DSM 106435 Microbacterium sp. No. 7 Rathayibacter rathayi Rathayibacter iranicus Microterricola viridarii Agromyces aureus Salinibacterium sp. CGMCC 1.16371 Salinibacterium sp. dk2585 Leucobacter triazinivorans Arthrobacter sp. LS16 Arthrobacter sp. Hiyo8 Arthrobacter sp. YC-RL1 Arthrobacter sp. ATCC 21022 Arthrobacter sp. QXT-31 Arthrobacter crystallopoietes Arthrobacter sp. PAMC25564 Pseudarthrobacter chlorophenolicus Pseudarthrobacter sulfonivorans Glutamicibacter sp. ZJUTW Neomicrococcus aestuarii Xylanimonas allomyrinae Intrasporangium calvum Janibacter indicus Janibacter limosus Janibacter melonis Microlunatus sp. KUDC0627 Raineyella sp. CBA3103 Nocardioides sp. JS614 Nocardioides dokdonensis Pimelobacter simplex Aeromicrobium sp. 592 Aeromicrobium sp. A1-2 Nocardiopsis dassonvillei Streptomonospora sp. M2 Thermomonospora curvata Actinomadura sp. WMMB499 Streptosporangium roseum Nonomuraea sp. ATCC 55076 Nonomuraea sp. WYY166 Frankia sp. EAN1pec Frankia inefficax Frankia alni Candidatus Frankia datiscae Nakamurella multipartita Saccharopolyspora sp. E2A Amycolatopsis mediterranei U32 Amycolatopsis mediterranei S699 Amycolatopsis mediterranei S699 Amycolatopsis mediterranei RB Amycolatopsis orientalis Amycolatopsis japonica Amycolatopsis methanolica Amycolatopsis sp. AA4 Amycolatopsis sp. BJA-103 Amycolatopsis albispora Amycolatopsis sp. YIM 10 Pseudonocardia dioxanivorans Pseudonocardia sp. AL041005-10 Pseudonocardia sp. EC080625-04 Pseudonocardia sp. EC080610-09 Pseudonocardia sp EC080619-01 Pseudonocardia sp. HH130630-07 Pseudonocardia autotrophica Actinosynnema pretiosum Kutzneria albida Kibdelosporangium phytohabitans Lentzea guizhouensis Alloactinosynnema sp. L-07 Prauserella marina Salinispora tropica Salinispora arenicola Micromonospora aurantiaca Micromonospora sp. L5 Micromonospora sp. B006 Micromonospora tulbaghiae Micromonospora terminaliae Verrucosispora maris Actinoplanes missouriensis Actinoplanes sp. N902-109 Actinoplanes friuliensis Plantactinospora sp. KBS50 Actinopolyspora erythraea Conexibacter woesei Egibacter rhizosphaerae Euzebya sp. DY32-46 Crocosphaera subtropica Crocosphaera watsonii Rippkaea orientalis PCC 8801 Rippkaea orientalis PCC 8802 Trichodesmium erythraeum Nostoc sp. PCC 7524 Calothrix sp. PCC 6303 Calothrix sp. 336/3 Rivularia sp. PCC 7116 Roseiflexus sp. RS-1 Roseiflexus castenholzii Chloroflexus aurantiacus Chloroflexus aurantiacus Y-400-fl Chloroflexus aggregans Thermomicrobium roseum Caldilinea aerophila Ktedonobacterales bacterium SCAWS-G2 Thermus thermophilus HB8 Thermus thermophilus JL-18 Thermus scotoductus Thermus sp. CCB\_US3\_UF1 Thermus oshimai Thermus brockianus Gemmata sp. SH-PL17 Paludisphaera borealis Treponema primitia Sediminispirochaeta smaragdinae Leptospira tipperaryensis Acidobacterium capsulatum Terriglobus roseus Terriglobus albidus Aminobacterium colombiense Cloacibacillus porcorum Bacteroides salanitronis Bacteroides dorei HS1\_L\_1\_B\_010 Bacteroides dorei HS1\_L\_3\_B\_079 Chryseolinea sp. KIS68-18 Flavobacterium psychrophilum Z2 Halobacterium sp. DL1 Halobellus limi Natronolimnobius aegyptiacus Nitrosopumilus maritimus | 184% 150% 122% 100% 82% 67% 55% |
