# Supplementary material for: Isolation, identification, and whole-genome sequencing of high-yield protease bacteria from Daqu of ZhangGong Laojiu
Source: PLoS One. 2022 Apr 26;17(4):e0264677. doi: 10.1371/journal.pone.0264677 (PMC9041807; doi:10.1371/journal.pone.0264677)
Supplement: S1 Raw images — (PDF) [file pone.0264677.s003.pdf]

Raw images

The following figure is the original gel electrophoresis picture of Fig. 3. The DNA fragment of the strain DW-7 was amplified by PCR, detected by agarose gel electrophoresis, and photographed by a gel imaging system. The marker used is DL2000, so it can be seen that The size of the amplified product is about 1500 bp, which is consistent with the size of the target product. Figure 3 captures the first two lanes of this photo.

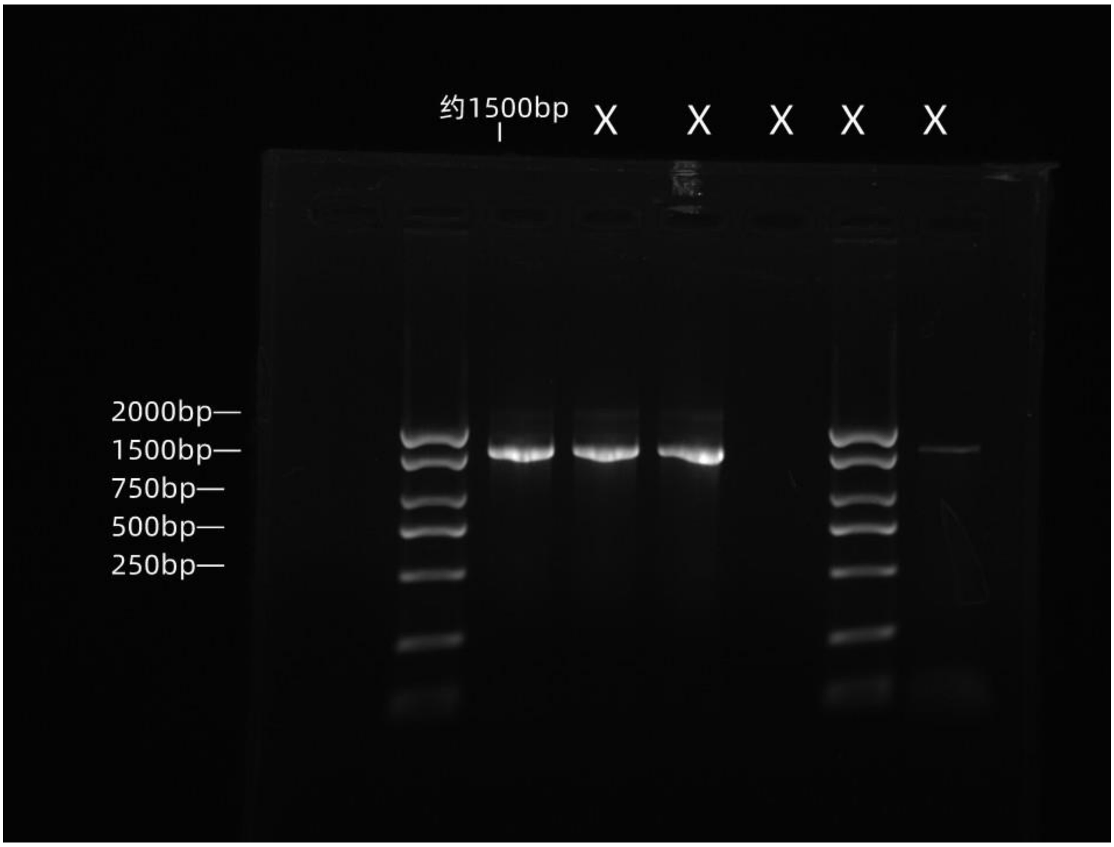

Table 1 Diameter of transparent ring and strain diameter ratio

No.	d/cm	D/cm	D/d
**DW-2**	0.60±0.06	2.30±0.10	3.84±0.25
**DW-7**	0.57±0.15	2.50±0.06	4.39±0.98
**DW-8**	0.50±0.06	1.90±0.06	3.80±0.34
**DW-22**	0.60±0.06	2.20±0.12	3.69±0.62
**DW-32**	0.60±0.06	2.40±0.10	4.00±0.25
**DW-40**	0.50±0.00	1.80±0.06	3.60±0.12

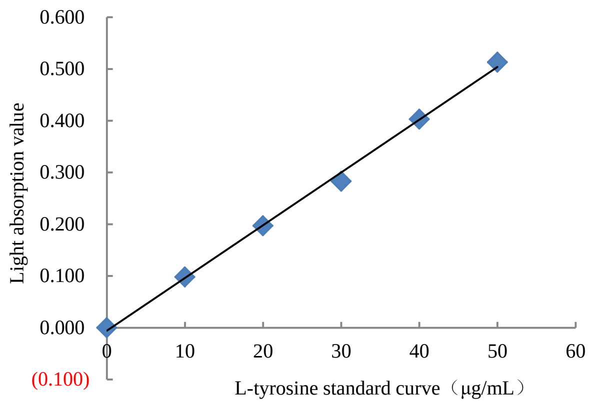

Fig 1 L-tyrosine standard curve

Table 2 Determination of protease activity

**No.**	**OD-value**	**protease activity（U/mL）**
**DW-2**	0.555±0.01	87.56±1.79
**DW-7**	0.631±0.01	99.54±2.23
**DW-8**	0.540±0.00	85.19±0.11
**DW-22**	0.525±0.00	82.82±0.00
**DW-32**	0.574±0.01	90.55±1.90
**DW-40**	0.497±0.03	78.41±4.24

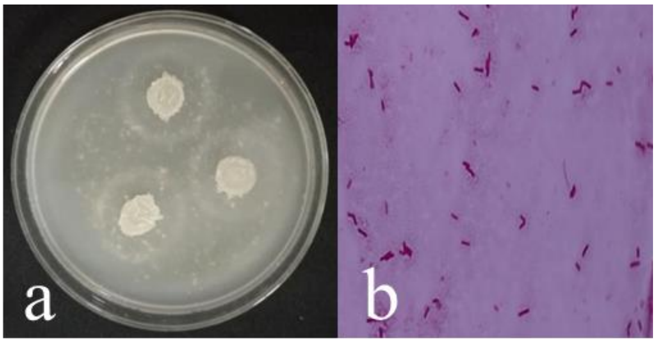

Fig 2 (a) Colony morphology of DW-7; (b) Morphology of DW-7 cells (magnification 1000X)

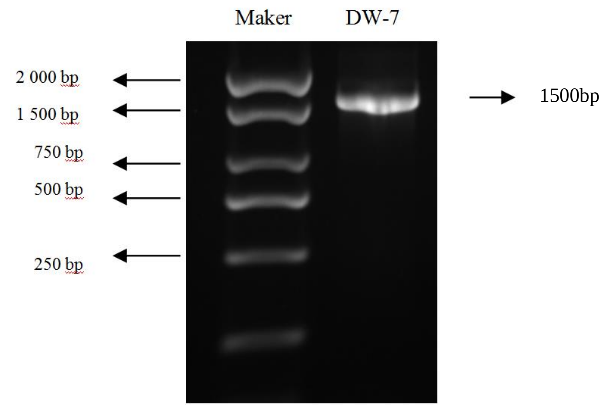

Fig 3 Electrophoresis results of DW-7

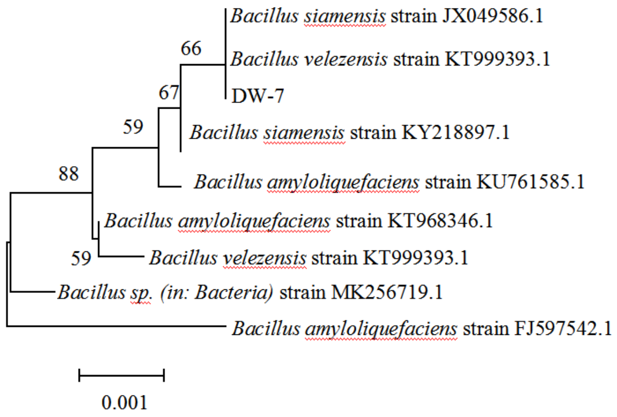

Fig 4 Phylogenetic tree of the DW-7 bacteria

Table 3. Physiological and biochemical analysis of the DW-7 bacterial strain

**Experiment**	**Result**
**Tolerance to 10% NaCl**	+
**Tolerance to pH 5.0**	+
**D-mannitol acid production**	+
**Catalase test**	+
**Propionate**	+
**Citrate**	-

+ indicates a positive result, and − indicates a negative result.

Table 4 Genome statistics of the DW-7 strain

**Type**	**Length**	**Amount**
**Sequence**	3942829 bp	1
**Gene**	3402822 bp	3662
**GC content**	46.46%	

**Total transposon length**	4515 bp	64
**Long scattered sequence**	1869 bp	24
**Short scattered sequence**	1474 bp	22
**Long terminal repeat sequence**	185 bp	3
**tandem repeat sequences**	10086 bp	147
**tRNA**	6639 bp	86
**rRNA**	41367 bp	37
**sRNA**	2227 bp	17
**Gene Islands**	28970 bp	1
**prophage**	90882 bp	1

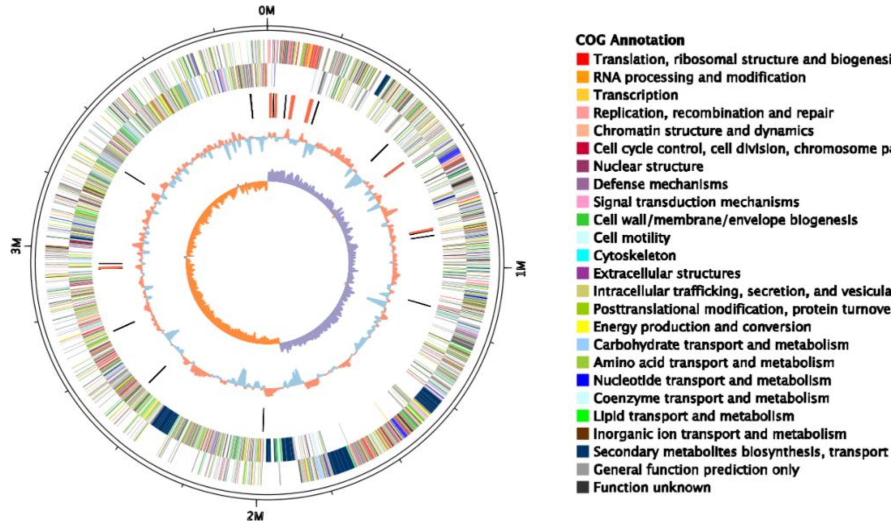

Fig 5 Complete map of the genome of DW-7 bacteria

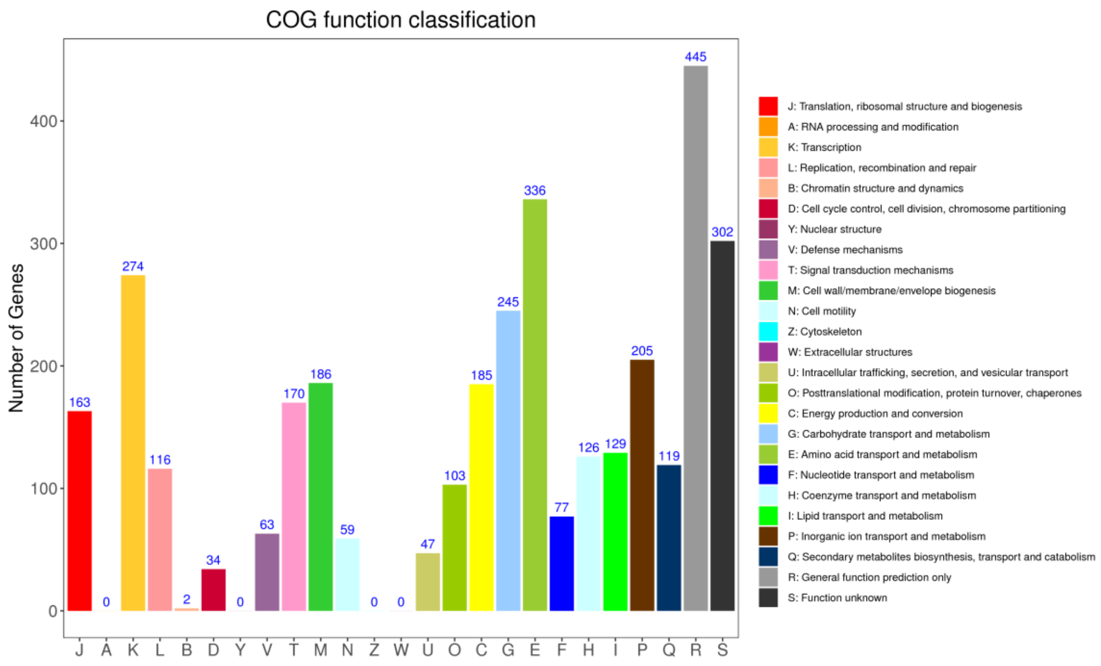

Fig 6 Functional classification of COG

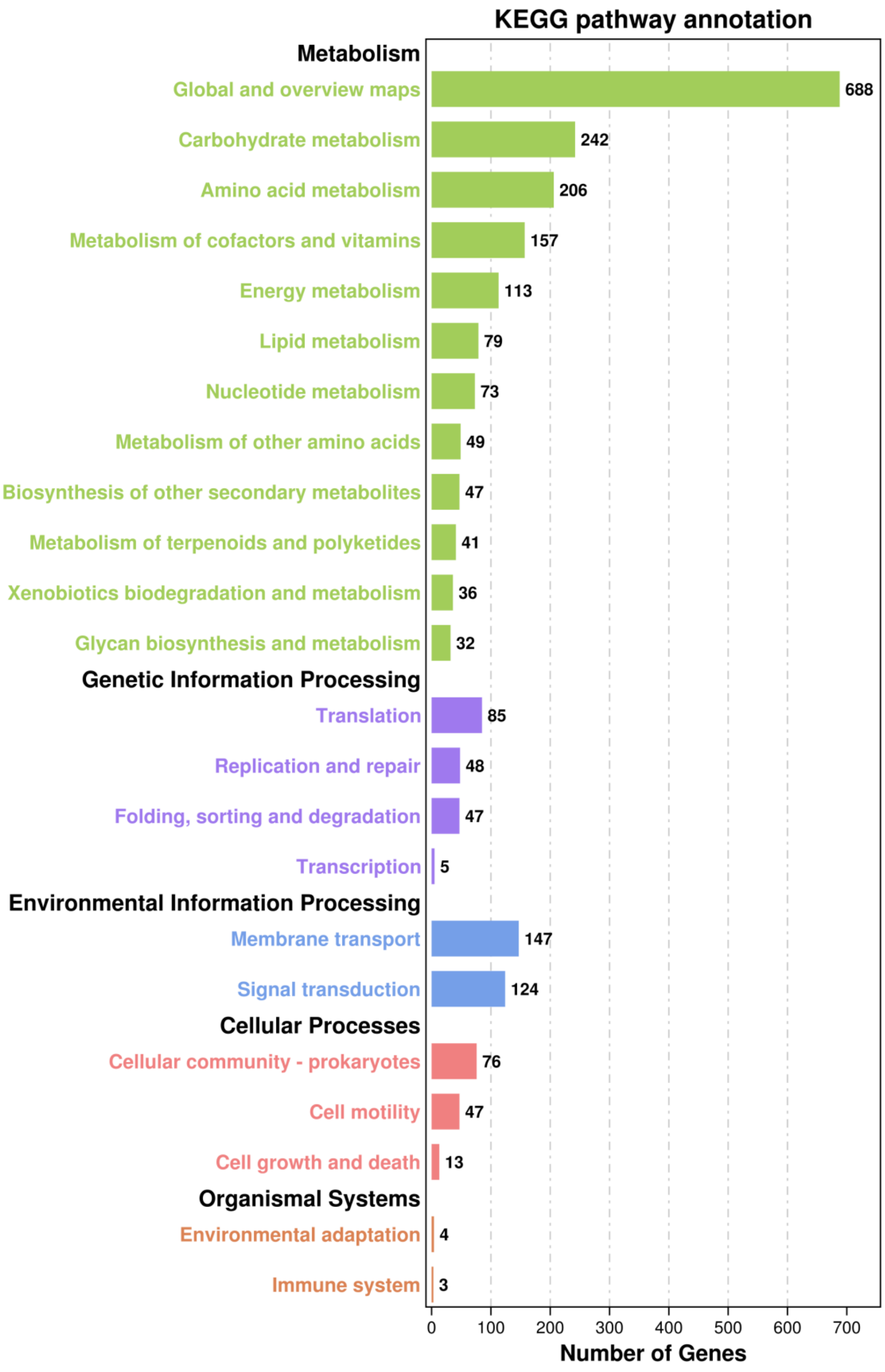

Fig 7 Bacterial gene functional annotation KEGG metabolic pathway

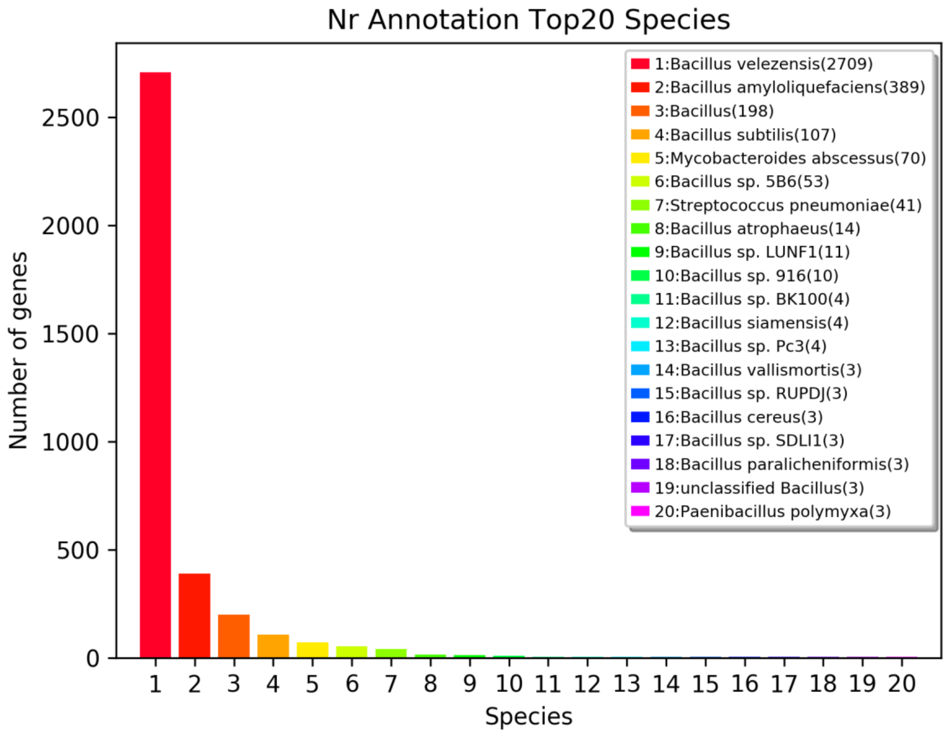

Fig 8 Annotated species statistics of Nr database (top 20 species)

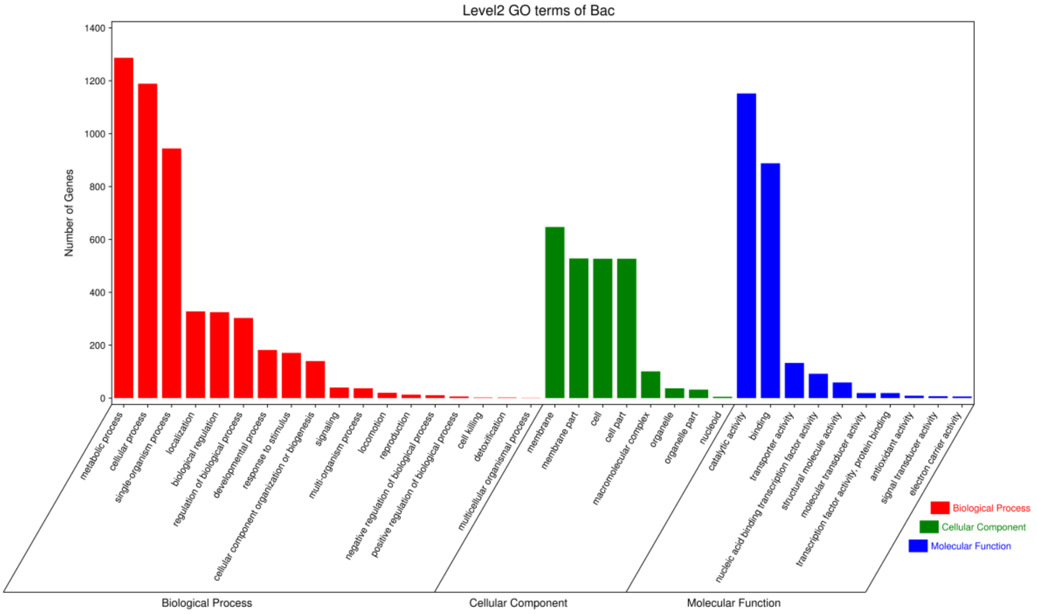

Fig 9 GO classification of bacterial gene function annotation
