# Supplementary material for: Isolation, identification, and whole-genome sequencing of high-yield protease bacteria from Daqu of ZhangGong Laojiu
Source: PLoS One. 2022 Apr 26;17(4):e0264677. doi: 10.1371/journal.pone.0264677 (PMC9041807; doi:10.1371/journal.pone.0264677)
Supplement: S1 Raw data — (ZIP) [file pone.0264677.s002.zip › Raw data/GDD20120317-1_Bacillus_velezensis_Genome_result/4_Basic_Annot/KEGG/Bac_map/map00642.html]

KEGG PATHWAY: Ethylbenzene degradation - Reference pathway


|  |  |
| --- | --- |
| **Ethylbenzene degradation - Reference pathway** |  |

[
Pathway menu
| Organism menu
| Pathway entry
| User data mapping
]

|  |  |
| --- | --- |
| Reference pathway Reference pathway (KO) Reference pathway (EC) Reference pathway (Reaction) -----< Sort below by alphabet >----- Shimwellia blattae Pseudoxanthomonas spadix Pseudomonas putida S16 Pseudomonas putida ND6 Pseudomonas putida DLL-E4 Pseudomonas plecoglossicida Pseudomonas veronii Pseudomonas stutzeri CCUG 29243 Pseudomonas stutzeri 19SMN4 Pseudomonas balearica Pseudomonas frederiksbergensis Azotobacter vinelandii DJ Azotobacter vinelandii CA Azotobacter vinelandii CA6 Psychrobacter urativorans Pseudoalteromonas translucida Alteromonas naphthalenivorans Halioglobus sp. IMCC14385 Cycloclasticus sp. P1 Cycloclasticus zancles Cycloclasticus sp. PY97N Marinomonas sp. MWYL1 Marinomonas mediterranea Marinomonas posidonica Candidatus Ruthia magnifica (Calyptogena magnifica) Candidatus Vesicomyosocius okutanii (Calyptogena okutanii) Pseudogulbenkiania sp. NH8B Ralstonia solanacearum GMI1000 Ralstonia solanacearum PSI07 Ralstonia solanacearum Po82 Ralstonia solanacearum CMR15 Ralstonia solanacearum FQY\_4 Ralstonia solanacearum UY031 Ralstonia pickettii DTP0602 Ralstonia mannitolilytica Ralstonia pseudosolanacearum Cupriavidus necator H16 Cupriavidus necator N-1 Cupriavidus necator NH9 Cupriavidus pinatubonensis JMP134 Cupriavidus basilensis Cupriavidus sp. USMAHM13 Cupriavidus malaysiensis Cupriavidus sp. USMAA2-4 Cupriavidus oxalaticus Burkholderia multivorans DDS 15A-1 Burkholderia cepacia ATCC 25416 Burkholderia pyrrocinia Burkholderia ubonensis Burkholderia glumae BGR1 Burkholderia glumae LMG 2196 = ATCC 33617 Burkholderia sp. CCGE1001 Burkholderia sp. HB1 Burkholderia sp. OLGA172 Paraburkholderia xenovorans LB400 Paraburkholderia xenovorans LB400 Paraburkholderia phenoliruptrix Paraburkholderia fungorum Paraburkholderia sprentiae Paraburkholderia aromaticivorans Paraburkholderia hospita Paraburkholderia terrae Paraburkholderia caffeinilytica Pandoraea pnomenusa 3kgm Pandoraea pnomenusa RB38 Pandoraea pnomenusa DSM 16536 Pandoraea pnomenusa Pandoraea pulmonicola Pandoraea sputorum Pandoraea apista Pandoraea vervacti Pandoraea oxalativorans Pandoraea faecigallinarum Pandoraea norimbergensis Pandoraea sp. XY-2 Pandoraea fibrosis Bordetella parapertussis Bpp5 Bordetella parapertussis 12822 Bordetella bronchiseptica 253 Bordetella bronchiseptica RB50 Bordetella bronchiseptica MO149 Bordetella bronchiseptica S798 Bordetella petrii Achromobacter xylosoxidans A8 Achromobacter xylosoxidans NH44784-1996 Achromobacter xylosoxidans NBRC 15126 = ATCC 27061 Achromobacter xylosoxidans NCTC10807 Achromobacter denitrificans Achromobacter insolitus Achromobacter spanius Achromobacter sp. AONIH1 Achromobacter sp. B7 Pusillimonas sp. ye3 Alcaligenes faecalis ZD02 Alcaligenes faecalis JQ135 Alcaligenes aquatilis Pigmentiphaga sp. H8 Polaromonas sp. JS666 Polaromonas naphthalenivorans Acidovorax citrulli Acidovorax sp. JS42 Acidovorax avenae Acidovorax sp. KKS102 Acidovorax carolinensis P4 Acidovorax carolinensis P3 Delftia sp. Cs1-4 Variovorax paradoxus S110 Variovorax paradoxus EPS Variovorax paradoxus B4 Variovorax boronicumulans Variovorax sp. PMC12 Comamonas testosteroni TK102 Limnohabitans sp. 103DPR2 Limnohabitans sp. 63ED37-2 Hydrogenophaga sp. PBC Hydrogenophaga sp. LPB0072 Hydrogenophaga pseudoflava Janthinobacterium sp. 17J80-10 Massilia sp. WG5 Massilia sp. YMA4 Leptothrix cholodnii Thiomonas intermedia Thiomonas arsenitoxydans Rhizobacter gummiphilus Aromatoleum aromaticum Dechloromonas aromatica Azoarcus sp. BH72 Azoarcus sp. KH32C Azoarcus olearius Azoarcus sp. DD4 Sinorhizobium sp. RAC02 Rhizobium sp. S41 Shinella sp. HZN7 Ochrobactrum anthropi ATCC 49188 Ochrobactrum anthropi OAB Bradyrhizobium sp. SK17 Bosea sp. PAMC 26642 Methylobacterium radiotolerans Methylobacterium nodulans Methylobacterium phyllosphaerae Methylobacterium sp. XJLW Microvirga ossetica Methylocella silvestris Chelatococcus daeguensis Hyphomicrobium sp. MC1 Nordella sp. HKS 07 Rhizobiales bacterium NRL2 Caulobacter flavus Jannaschia sp. CCS1 Paracoccus yeei Paracoccus sp. Arc7-R13 Paracoccus kondratievae Pseudovibrio sp. FO-BEG1 Celeribacter manganoxidans Confluentimicrobium sp. EMB200-NS6 Pannonibacter phragmitetus Labrenzia sp. CP4 Labrenzia aggregata Labrenzia sp. VG12 Labrenzia sp. THAF35 Boseongicola sp. CCM32 Stappia indica Novosphingobium aromaticivorans Novosphingobium sp. PP1Y Novosphingobium pentaromativorans Novosphingobium sp. P6W Sphingopyxis fribergensis Sphingopyxis macrogoltabida EY-1 Sphingopyxis granuli Sphingomonas panacis Sphingobium chlorophenolicum Croceicoccus marinus Stella humosa Azospirillum sp. B510 Azospirillum lipoferum Tistrella mobilis Thalassospira indica Paenibacillus naphthalenovorans Mycobacterium sp. MCS Mycobacterium sp. KMS Mycobacterium sp. JLS Mycobacterium sp. EPa45 Mycolicibacterium vanbaalenii Mycolicibacterium gilvum PYR-GCK Rhodococcus jostii Rhodococcus opacus B4 Streptomyces bingchenggensis Thermomonospora curvata Actinomadura sp. WMMB499 Conexibacter woesei Ilumatobacter coccineus Thermus oshimai Aequorivita sublithincola Candidatus Bipolaricaulis anaerobius Nitrososphaera viennensis Candidatus Nitrososphaera evergladensis | 184% 150% 122% 100% 82% 67% 55% |
