# Supplementary material for: Isolation, identification, and whole-genome sequencing of high-yield protease bacteria from Daqu of ZhangGong Laojiu
Source: PLoS One. 2022 Apr 26;17(4):e0264677. doi: 10.1371/journal.pone.0264677 (PMC9041807; doi:10.1371/journal.pone.0264677)
Supplement: S1 Raw data — (ZIP) [file pone.0264677.s002.zip › Raw data/GDD20120317-1_Bacillus_velezensis_Genome_result/4_Basic_Annot/KEGG/Bac_map/map00791.html]

KEGG PATHWAY: Atrazine degradation - Reference pathway


|  |  |
| --- | --- |
| **Atrazine degradation - Reference pathway** |  |

[
Pathway menu
| Organism menu
| Pathway entry
| User data mapping
]

|  |  |
| --- | --- |
| Reference pathway Reference pathway (KO) Reference pathway (EC) Reference pathway (Reaction) -----< Sort below by alphabet >----- Saccharomyces cerevisiae (budding yeast) Ashbya gossypii (Eremothecium gossypii) Eremothecium cymbalariae Kluyveromyces lactis Kluyveromyces marxianus Lachancea thermotolerans Vanderwaltozyma polyspora Zygosaccharomyces rouxii Candida glabrata Naumovozyma castellii Naumovozyma dairenensis Tetrapisispora phaffii Torulaspora delbrueckii Komagataella phaffii Debaryomyces hansenii Scheffersomyces stipitis Meyerozyma guilliermondii Spathaspora passalidarum Lodderomyces elongisporus Candida albicans Candida tropicalis Candida orthopsilosis Candida dubliniensis Yamadazyma tenuis Yarrowia lipolytica Clavispora lusitaniae ATCC 42720 Clavispora lusitaniae CBS 6936 Candida auris Sugiyamaella lignohabitans Pichia kudriavzevii Neurospora crassa Neurospora tetrasperma Podospora anserina Thermothelomyces thermophilus Chaetomium thermophilum Sporothrix schenckii Fusarium graminearum Fusarium pseudograminearum Fusarium verticillioides Fusarium oxysporum Nectria haematococca Trichoderma reesei QM6a Trichoderma reesei RUT C-30 Metarhizium acridum Metarhizium robertsii Cordyceps militaris Purpureocillium lilacinum Verticillium alfalfae Verticillium dahliae Colletotrichum fioriniae Eutypa lata Pestalotiopsis fici Sclerotinia sclerotiorum Botrytis cinerea Phialocephala scopiformis Glarea lozoyensis Aspergillus nidulans Aspergillus fumigatus Aspergillus clavatus Aspergillus fischeri Aspergillus oryzae Aspergillus niger Aspergillus flavus Penicillium rubens Talaromyces marneffei Coccidioides immitis Coccidioides posadasii Paracoccidioides lutzii Pb01 Paracoccidioides brasiliensis Uncinocarpus reesii Trichophyton benhamiae Trichophyton verrucosum Histoplasma capsulatum Parastagonospora nodorum Pyrenophora teres Bipolaris zeicola Bipolaris sorokiniana Bipolaris oryzae Alternaria alternata Pseudocercospora fijiensis Baudoinia panamericana Neofusicoccum parvum Cryptococcus gattii Postia placenta Trametes versicolor Dichomitus squalens Phanerochaete carnosa Stereum hirsutum Heterobasidion irregulare Punctularia strigosozonata Auricularia subglabra Fomitiporia mediterranea Gloeophyllum trabeum Moniliophthora roreri Schizophyllum commune Agaricus bisporus var. burnettii JB137-S8 Agaricus bisporus var. bisporus H97 Coniophora puteana Serpula lacrymans Anthracocystis flocculosa Escherichia coli UMNK88 (ETEC, porcine) Salmonella enterica subsp. enterica serovar Typhi P-stx-12 Enterobacter cloacae subsp. cloacae ATCC 13047 Enterobacter cloacae subsp. dissolvens SDM Enterobacter cloacae GGT036 Enterobacter cloacae ECNIH5 Enterobacter hormaechei subsp. hoffmannii ECNIH3 Enterobacter ludwigii EcWSU1 Enterobacter ludwigii EN-119 Enterobacter soli Enterobacter sp. FY-07 Cronobacter sakazakii ATCC BAA-894 Cronobacter sakazakii ES15 Cronobacter sakazakii Sp291 Cronobacter sakazakii ATCC 29544 Cronobacter condimenti Cronobacter dublinensis Cronobacter malonaticus CMCC45402 Cronobacter malonaticus LMG 23826 Cronobacter universalis Cronobacter muytjensii Cronobacter turicensis Klebsiella pneumoniae 342 Klebsiella variicola At-22 Klebsiella variicola KP5-1 Klebsiella variicola DX120E Klebsiella variicola DSM 15968 Klebsiella michiganensis KCTC 1686 Klebsiella michiganensis E718 Klebsiella michiganensis HKOPL1 Klebsiella michiganensis M1 Klebsiella michiganensis RC10 Klebsiella oxytoca KONIH1 Klebsiella oxytoca CAV1374 Klebsiella quasipneumoniae Klebsiella sp. LTGPAF-6F Klebsiella huaxiensis Citrobacter youngae Citrobacter portucalensis Gibbsiella quercinecans Raoultella ornithinolytica B6 Raoultella ornithinolytica S12 Raoultella sp. X13 Raoultella terrigena Raoultella electrica Cedecea neteri SSMD04 Cedecea neteri M006 Cedecea neteri ND14a Cedecea lapagei Kosakonia oryzae Kosakonia radicincitans Kluyvera intermedia Leclercia adecarboxylata Leclercia sp. LSNIH1 Leclercia sp. W17 Leclercia sp. 1106151 Leclercia sp. J807 Buttiauxella sp. 3AFRM03 Metakosakonia sp. MRY16-398 Atlantibacter hermannii Izhakiella sp. KSNA2 Enterobacteriaceae bacterium FGI 57 Enterobacteriaceae bacterium ENNIH2 Enterobacteriaceae bacterium S05 Plautia stali symbiont Serratia marcescens SM39 Serratia marcescens subsp. marcescens Db11 Serratia marcescens WW4 Serratia proteamaculans Serratia plymuthica AS9 Serratia plymuthica 4Rx13 Serratia plymuthica S13 Serratia plymuthica PRI-2C Serratia sp. AS12 Serratia sp. AS13 Serratia sp. FGI94 Serratia liquefaciens Serratia sp. FS14 Serratia sp. SCBI Serratia rubidaea Serratia sp. ATCC 39006 Serratia sp. ATCC 39006 Serratia sp. MYb239 Serratia quinivorans Serratia ficaria Rahnella sp. Y9602 Rahnella aquatilis CIP 78.65 = ATCC 33071 Rahnella aquatilis HX2 Rahnella sp. ERMR1:05 Pectobacterium atrosepticum SCRI1043 Pectobacterium atrosepticum JG10-08 Pectobacterium atrosepticum 21A Pectobacterium carotovorum subsp. carotovorum PC1 Pectobacterium carotovorum subsp. carotovorum PCC21 Pectobacterium parmentieri WPP163 Pectobacterium parmentieri RNS08.42.1A Pectobacterium parmentieri SCC3193 Pectobacterium wasabiae Pectobacterium polaris Pectobacterium brasiliense Dickeya dadantii 3937 Dickeya chrysanthemi Dickeya zeae Ech586 Dickeya zeae EC1 Dickeya solani IPO 2222 Dickeya fangzhongdai ND14b Dickeya fangzhongdai DSM 101947 Dickeya dianthicola Dickeya aquatica Dickeya sp. NCPPB 569 Brenneria goodwinii Brenneria nigrifluens Erwinia tasmaniensis Erwinia billingiae Erwinia gerundensis Erwinia persicina Erwinia sp. J780 Pantoea ananatis LMG 20103 Pantoea ananatis LMG 5342 Pantoea ananatis AJ13355 Pantoea ananatis PA13 Pantoea vagans C9-1 Pantoea vagans FDAARGOS\_160 Pantoea sp. At-9b Pantoea rwandensis Pantoea agglomerans C410P1 Pantoea stewartii subsp. stewartii Pantoea sp. SO10 Pantoea eucalypti Tatumella citrea Leminorella richardii Phytobacter ursingii Pasteurella dagmatis Xanthomonas citri pv. citri 306 Xanthomonas citri subsp. citri Aw12879 Xanthomonas citri subsp. citri A306 Xanthomonas citri subsp. citri UI6 Xanthomonas citri pv. citri UI7 Xanthomonas citri pv. citri NT17 Xanthomonas citri pv. citri MN10 Xanthomonas citri pv. citri MN11 Xanthomonas citri pv. citri MN12 Xanthomonas citri pv. citri mf20 Xanthomonas axonopodis Xac29-1 Xanthomonas phaseoli Xanthomonas sp. ISO98C4 Pseudoxanthomonas suwonensis J1 Pseudoxanthomonas spadix Vibrio owensii Vibrio nigripulchritudo Vibrio coralliilyticus OCN014 Vibrio breoganii Vibrio gazogenes Grimontia hollisae Pseudomonas aeruginosa PAO1-VE13 Pseudomonas aeruginosa PAO1-VE2 Pseudomonas aeruginosa UCBPP-PA14 Pseudomonas aeruginosa PA7 Pseudomonas aeruginosa LESB58 Pseudomonas aeruginosa M18 Pseudomonas aeruginosa NCGM2.S1 Pseudomonas aeruginosa NCGM 1900 Pseudomonas aeruginosa DK2 Pseudomonas aeruginosa B136-33 Pseudomonas aeruginosa RP73 Pseudomonas aeruginosa PA1 Pseudomonas aeruginosa PA1R Pseudomonas aeruginosa MTB-1 Pseudomonas aeruginosa LES431 Pseudomonas aeruginosa SCV20265 Pseudomonas aeruginosa PA38182 Pseudomonas aeruginosa YL84 Pseudomonas aeruginosa c7447m Pseudomonas aeruginosa PAO581 Pseudomonas mendocina ymp Pseudomonas mendocina NK-01 Pseudomonas resinovorans Pseudomonas pseudoalcaligenes Pseudomonas alcaligenes Pseudomonas citronellolis Pseudomonas putida F1 Pseudomonas putida GB-1 Pseudomonas putida W619 Pseudomonas putida S16 Pseudomonas putida BIRD-1 Pseudomonas putida ND6 Pseudomonas putida DOT-T1E Pseudomonas putida HB3267 Pseudomonas putida H8234 Pseudomonas putida NBRC 14164 Pseudomonas putida DLL-E4 Pseudomonas fulva Pseudomonas monteilii SB3078 Pseudomonas monteilii SB3101 Pseudomonas soli Pseudomonas plecoglossicida Pseudomonas oryzihabitans Pseudomonas syringae pv. tomato DC3000 Pseudomonas syringae pv. syringae B728a Pseudomonas syringae CC1557 Pseudomonas savastanoi pv. phaseolicola 1448A Pseudomonas amygdali Pseudomonas cichorii Pseudomonas avellanae Pseudomonas protegens Pf-5 Pseudomonas protegens CHA0 Pseudomonas protegens Cab57 Pseudomonas fluorescens Pf0-1 Pseudomonas fluorescens SBW25 Pseudomonas fluorescens F113 Pseudomonas fluorescens A506 Pseudomonas fluorescens UK4 Pseudomonas poae Pseudomonas synxantha LBUM223 Pseudomonas mandelii Pseudomonas trivialis Pseudomonas corrugata Pseudomonas veronii Pseudomonas azotoformans Pseudomonas simiae PCL1751 Pseudomonas simiae PICF7 Pseudomonas lurida Pseudomonas stutzeri A1501 Pseudomonas stutzeri ATCC 17588 Pseudomonas stutzeri DSM 4166 Pseudomonas stutzeri CCUG 29243 Pseudomonas stutzeri RCH2 Pseudomonas stutzeri 19SMN4 Pseudomonas balearica Pseudomonas brassicacearum subsp. brassicacearum NFM421 Pseudomonas brassicacearum DF41 Pseudomonas sp. UW4 Pseudomonas sp. ATCC 13867 Pseudomonas sp. VLB120 Pseudomonas sp. TKP Pseudomonas knackmussii Pseudomonas chlororaphis PA23 Pseudomonas chlororaphis PCL1606 Pseudomonas chlororaphis subsp. aurantiaca Pseudomonas cremoricolorata Pseudomonas sp. StFLB209 Pseudomonas sp. MRSN12121 Pseudomonas sp. CCOS 191 Pseudomonas sp. Os17 Pseudomonas koreensis Pseudomonas frederiksbergensis Pseudomonas antarctica Pseudomonas psychrotolerans Pseudomonas sp. TCU-HL1 Pseudomonas silesiensis Pseudomonas yamanorum Pseudomonas sp. R2A2 Pseudomonas kribbensis Acinetobacter baumannii ATCC 17978 Acinetobacter baumannii AYE Acinetobacter baumannii ACICU Acinetobacter baumannii AB0057 Acinetobacter baumannii AB307-0294 Acinetobacter baumannii 1656-2 Acinetobacter baumannii MDR-ZJ06 Acinetobacter baumannii MDR-TJ Acinetobacter baumannii TCDC-AB0715 Acinetobacter baumannii TYTH-1 Acinetobacter baumannii D1279779 Acinetobacter baumannii BJAB07104 Acinetobacter baumannii BJAB0715 Acinetobacter baumannii BJAB0868 Acinetobacter baumannii ZW85-1 Acinetobacter baumannii AbH12O-A2 Acinetobacter baumannii AB030 Acinetobacter baumannii AC29 Acinetobacter baumannii LAC-4 Acinetobacter pittii Acinetobacter nosocomialis Acinetobacter lactucae Acinetobacter calcoaceticus Acinetobacter oleivorans Acinetobacter johnsonii Acinetobacter haemolyticus Acinetobacter junii Acinetobacter larvae Acinetobacter schindleri Acinetobacter defluvii Acinetobacter radioresistens Acinetobacter wuhouensis Acinetobacter cumulans Acinetobacter guillouiae Acinetobacter ursingii Acinetobacter lwoffii Moraxellaceae bacterium HYN0046 Marinobacter adhaerens Marinobacter sp. BSs20148 Marinobacter salarius Marinobacter psychrophilus Marinobacter sp. CP1 Marinobacter sp. LQ44 Marinobacter salinus Marinobacter sp. Arc7-DN-1 Salinimonas sediminis Hydrocarboniclastica marina Psychromonas ingrahamii Cellvibrio japonicus Cellvibrio sp. PSBB023 Cellvibrio sp. PSBB006 Cellvibrio sp. KY-YJ-3 Cellvibrio sp. KY-GH-1 Saccharophagus degradans Teredinibacter turnerae Spongiibacter sp. IMCC21906 Zhongshania aliphaticivorans Microbulbifer thermotolerans Microbulbifer sp. A4B17 Methylomonas sp. DH-1 Methylomonas koyamae Methylomonas sp. LW13 Methylovulum psychrotolerans Methylocaldum marinum Hydrogenovibrio crunogenus Hydrogenovibrio thermophilus Thiomicrospira sp. S5 Thiomicrorhabdus sp. G1 Candidatus Thiodictyon syntrophicum Halorhodospira halophila Thioalkalivibrio sulfidiphilus Spiribacter roseus Acidihalobacter prosperus Halothiobacillus neapolitanus Halothiobacillus sp. LS2 Halomonas sp. KO116 Halomonas sp. R57-5 Halomonas sp. 1513 Halomonas hydrothermalis Halomonas sp. SF2003 Halomonas alkaliphila Halomonas olivaria Halotalea alkalilenta Cobetia marina Alcanivorax borkumensis Alcanivorax dieselolei Alcanivorax pacificus Alcanivorax sp. NBRC 101098 Alcanivorax xenomutans Ketobacter alkanivorans Marinomonas posidonica Thalassolituus oleivorans MIL-1 Thalassolituus oleivorans R6-15 Marinobacterium aestuarii Bacterioplanes sanyensis Gynuella sunshinyii Reinekea forsetii Oleiphilus messinensis Tolumonas auensis Solimonas sp. K1W22B-7 Immundisolibacter cernigliae Acidiferrobacter sp. SPIII\_3 Salinisphaera sp. LB1 Sedimenticola thiotaurini Laribacter hongkongensis Aquitalea magnusonii Cupriavidus necator NH9 Cupriavidus pinatubonensis JMP134 Cupriavidus basilensis Cupriavidus gilardii Cupriavidus malaysiensis Cupriavidus sp. USMAA2-4 Burkholderia mallei ATCC 23344 Burkholderia mallei SAVP1 Burkholderia mallei NCTC 10229 Burkholderia mallei NCTC 10247 Burkholderia mallei 23344 Burkholderia mallei 6 Burkholderia mallei BMQ Burkholderia mallei 2000031063 Burkholderia mallei FMH 23344 Burkholderia mallei NCTC 10247 Burkholderia mallei 2002734299 Burkholderia pseudomallei K96243 Burkholderia pseudomallei 1710b Burkholderia pseudomallei 1106a Burkholderia pseudomallei 668 Burkholderia pseudomallei MSHR305 Burkholderia pseudomallei MSHR511 Burkholderia pseudomallei MSHR146 Burkholderia pseudomallei MSHR520 Burkholderia pseudomallei 1026b Burkholderia pseudomallei BPC006 Burkholderia pseudomallei NCTC 13179 Burkholderia pseudomallei HBPUB10134a Burkholderia pseudomallei NAU35A-3 Burkholderia pseudomallei A79A Burkholderia pseudomallei TSV202 Burkholderia thailandensis E264 Burkholderia thailandensis 2002721723 Burkholderia thailandensis E444 Burkholderia thailandensis H0587 Burkholderia thailandensis MSMB121 Burkholderia thailandensis MSMB59 Burkholderia thailandensis E254 Burkholderia thailandensis USAMRU Malaysia #20 Burkholderia thailandensis 2003015869 Burkholderia thailandensis 2002721643 Burkholderia oklahomensis E0147 Burkholderia oklahomensis C6786 Burkholderia sp. BDU6 Burkholderia vietnamiensis G4 Burkholderia vietnamiensis LMG 10929 Burkholderia lata Burkholderia cenocepacia AU1054 Burkholderia cenocepacia HI2424 Burkholderia cenocepacia MC0-3 Burkholderia cenocepacia J2315 Burkholderia cenocepacia DDS 22E-1 Burkholderia cenocepacia DWS 37E-2 Burkholderia cenocepacia H111 Burkholderia multivorans ATCC 17616 (Tohoku) Burkholderia multivorans ATCC 17616 (JGI) Burkholderia multivorans ATCC BAA-247 Burkholderia cepacia DDS 7H-2 Burkholderia cepacia ATCC 25416 Burkholderia dolosa Burkholderia pyrrocinia Burkholderia contaminans Burkholderia diffusa Burkholderia latens Burkholderia territorii Burkholderia seminalis Burkholderia pseudomultivorans Burkholderia metallica Burkholderia stagnalis Burkholderia stabilis Burkholderia glumae BGR1 Burkholderia glumae LMG 2196 = ATCC 33617 Burkholderia sp. CCGE1001 Burkholderia gladioli BSR3 Burkholderia gladioli ATCC 10248 Burkholderia sp. YI23 Burkholderia sp. KJ006 Burkholderia insecticola Burkholderia sp. RPE67 Burkholderia sp. 2002721687 Burkholderia sp. HB1 Burkholderia plantarii PG1 Burkholderia plantarii ATCC 43733 Burkholderia sp. Bp5365 Burkholderia sp. OLGA172 Burkholderia sp. PAMC 26561 Burkholderia sp. PAMC 28687 Paraburkholderia xenovorans LB400 Paraburkholderia xenovorans LB400 Paraburkholderia phymatum Paraburkholderia sp. CCGE1002 Paraburkholderia phenoliruptrix Paraburkholderia phytofirmans Paraburkholderia fungorum Paraburkholderia caribensis Paraburkholderia sprentiae Paraburkholderia sp. SOS3 Paraburkholderia aromaticivorans Paraburkholderia hospita Paraburkholderia terrae Paraburkholderia graminis Paraburkholderia caledonica Paraburkholderia terricola Paraburkholderia caffeinilytica Pandoraea pnomenusa RB38 Pandoraea pnomenusa Pandoraea apista Pandoraea norimbergensis Paucimonas lemoignei Caballeronia sp. SBC2 Bordetella petrii Bordetella avium Bordetella holmesii ATCC 51541 Bordetella holmesii 44057 Bordetella hinzii Bordetella pseudohinzii Bordetella genomosp. 13 Bordetella sp. J329 Achromobacter xylosoxidans A8 Achromobacter xylosoxidans NH44784-1996 Achromobacter xylosoxidans NBRC 15126 = ATCC 27061 Achromobacter xylosoxidans NCTC10807 Achromobacter denitrificans Achromobacter insolitus Achromobacter spanius Achromobacter sp. AONIH1 Achromobacter sp. B7 Advenella mimigardefordensis Orrella dioscoreae Pigmentiphaga aceris Kerstersia gyiorum Rhodoferax ferrireducens Rhodoferax saidenbachensis Rhodoferax koreense Polaromonas naphthalenivorans Polaromonas sp. SP1 Polaromonas sp. Pch-P Acidovorax avenae Verminephrobacter eiseniae Delftia acidovorans Delftia sp. Cs1-4 Delftia sp. HK171 Variovorax paradoxus S110 Variovorax paradoxus EPS Variovorax paradoxus B4 Variovorax sp. PAMC 28711 Variovorax boronicumulans Variovorax sp. PMC12 Comamonas testosteroni TK102 Comamonas serinivorans Hydrogenophaga sp. RAC07 Hydrogenophaga sp. PBC Hydrogenophaga sp. LPB0072 Hydrogenophaga sp. PAMC20947 Hydrogenophaga pseudoflava Serpentinomonas raichei Methylibium petroleiphilum Methylibium sp. Pch-M Janthinobacterium sp. Marseille Herbaspirillum seropedicae SmR1 Herbaspirillum seropedicae Z67 Herbaspirillum hiltneri Herbaspirillum rubrisubalbicans Herbaspirillum sp. meg3 Herbaspirillum huttiense Massilia umbonata Leptothrix cholodnii Thiomonas arsenitoxydans Rhizobacter gummiphilus Sphaerotilus natans Xylophilus sp. KACC 21265 Polyangium brachysporum Nitrosomonas sp. AL212 Nitrosomonas sp. Is79A3 Nitrosomonas communis Nitrosomonas ureae Nitrosospira multiformis Nitrosospira lacus Methylotenera versatilis Methylovorus glucosetrophus Methylovorus sp. MP688 Candidatus Methylopumilus turicensis Methylophilus sp. TWE2 Sideroxydans lithotrophicus Gallionella capsiferriformans Sulfuriferula sp. AH1 Ferriphaselus amnicola Dechloromonas aromatica Azoarcus sp. BH72 Azoarcus olearius Azoarcus communis Azoarcus sp. DD4 Betaproteobacteria bacterium GR16-43 Helicobacter felis Helicobacter bizzozeronii Helicobacter bilis Wolinella succinogenes Sulfuricurvum kujiense Arcobacter butzleri 7h1h Arcobacter nitrofigilis Arcobacter sp. L Sulfurospirillum multivorans Sulfurospirillum halorespirans Sulfurospirillum sp. SL2-1 Sulfurospirillum sp. JPD-1 Desulfovibrio desulfuricans ND132 Stigmatella aurantiaca Sorangium cellulosum So ce56 Sorangium cellulosum So0157-2 Haliangium ochraceum Mesorhizobium japonicum MAFF 303099 Mesorhizobium loti NZP2037 Mesorhizobium ciceri (biovar Biserrulae) Mesorhizobium opportunistum Mesorhizobium australicum Mesorhizobium amorphae Mesorhizobium sp. B7 Mesorhizobium sp. WSM1497 Mesorhizobium sp. M9A.F.Ca.ET.002.03.1.2 Mesorhizobium sp. Pch-S Mesorhizobium huakuii Hoeflea sp. IMCC20628 Phyllobacterium zundukense Parvibaculum lavamentivorans Rhodobiaceae bacterium SMS8 Sinorhizobium meliloti AK83 Sinorhizobium meliloti BL225C Sinorhizobium meliloti SM11 Sinorhizobium meliloti Rm41 Sinorhizobium meliloti GR4 Sinorhizobium meliloti 2011 Sinorhizobium meliloti RMO17 Sinorhizobium medicae Sinorhizobium fredii NGR234 Sinorhizobium fredii USDA 257 Sinorhizobium sp. RAC02 Ensifer adhaerens OV14 Ensifer adhaerens Casida A Ensifer sojae Ensifer alkalisoli Agrobacterium radiobacter Agrobacterium tumefaciens S33 Agrobacterium vitis Agrobacterium sp. RAC06 Agrobacterium sp. 33MFTa1.1 Agrobacterium larrymoorei Rhizobium etli CFN 42 Rhizobium etli CIAT 652 Rhizobium etli bv. mimosae Mim1 Rhizobium etli bv. phaseoli IE4803 Rhizobium sp. IE4771 Rhizobium leguminosarum bv. viciae 3841 Rhizobium leguminosarum bv. trifolii WSM2304 Rhizobium leguminosarum bv. trifolii WSM1325 Rhizobium leguminosarum bv. trifolii WSM1689 Rhizobium leguminosarum bv. trifolii CB782 Rhizobium tropici Rhizobium sp. IRBG74 Rhizobium favelukesii Rhizobium gallicum Rhizobium phaseoli Rhizobium sp. S41 Rhizobium sp. Kim5 Rhizobium jaguaris Rhizobium sp. 11515TR Rhizobium grahamii Rhizobium acidisoli Neorhizobium galegae bv. officinalis bv. officinalis HAMBI 1141 Neorhizobium galegae bv. orientalis HAMBI 540 Neorhizobium sp. NCHU2750 Shinella sp. HZN7 Bradyrhizobium diazoefficiens USDA 110 Bradyrhizobium japonicum USDA 6 Bradyrhizobium japonicum E109 Bradyrhizobium sp. ORS 278 Bradyrhizobium sp. BTAi1 Bradyrhizobium sp. S23321 Bradyrhizobium oligotrophicum Bradyrhizobium sp. CCGE-LA001 Bradyrhizobium sp. BF49 Bradyrhizobium icense Bradyrhizobium sp. ORS 285 Bradyrhizobium sp. SK17 Bradyrhizobium ottawaense Bradyrhizobium amphicarpaeae Bradyrhizobium guangdongense Bradyrhizobium guangzhouense Bradyrhizobium symbiodeficiens Bradyrhizobium betae Rhodopseudomonas palustris CGA009 Rhodopseudomonas palustris HaA2 Rhodopseudomonas palustris BisB18 Rhodopseudomonas palustris BisB5 Rhodopseudomonas palustris BisA53 Rhodopseudomonas palustris TIE-1 Rhodopseudomonas palustris DX-1 Bosea sp. PAMC 26642 Bosea sp. RAC05 Bosea vaviloviae Bosea sp. Tri-49 Bosea sp. F3-2 Variibacter gotjawalensis Xanthobacter autotrophicus Azorhizobium caulinodans Starkeya novella Labrys neptuniae Ancylobacter sp. TS-1 Ancylobacter pratisalsi Methylorubrum extorquens AM1 Methylorubrum extorquens DM4 Methylorubrum extorquens PA1 Methylorubrum extorquens CM4 Methylorubrum populi Methylorubrum zatmanii Methylobacterium radiotolerans Methylobacterium sp. 4-46 Methylobacterium oryzae Methylobacterium sp. AMS5 Methylobacterium aquaticum Methylobacterium phyllosphaerae Methylobacterium sp. DM1 Methylobacterium sp. XJLW Methylobacterium sp. 17SD2-17 Methylobacterium sp. 17Sr1-43 Methylobacterium mesophilicum Methylobacterium terrae Microvirga ossetica Methylocella silvestris Methylocella tundrae Beijerinckiaceae bacterium RH AL1 Hyphomicrobium sp. MC1 Pelagibacterium halotolerans Devosia sp. I507 Blastochloris viridis Blastochloris sp. GI Rhodoplanes sp. Z2-YC6860 Pleomorphomonas sp. SM30 Martelella endophytica Martelella mediterranea Aureimonas sp. AU20 Breoghania sp. L-A4 Pseudorhodoplanes sinuspersici Hartmannibacter diazotrophicus Nordella sp. HKS 07 Rhizobiales bacterium NRL2 Caulobacter vibrioides CB15 Caulobacter vibrioides NA1000 Caulobacter sp. K31 Caulobacter sp. FWC26 Asticcacaulis excentricus Ruegeria sp. TM1040 Ruegeria sp. AD91A Epibacterium mobile Rhodobacter sphaeroides 2.4.1 Jannaschia sp. CCS1 Roseobacter litoralis Pseudovibrio sp. FO-BEG1 Phaeobacter piscinae Octadecabacter antarcticus Octadecabacter arcticus Celeribacter indicus Celeribacter ethanolicus Celeribacter manganoxidans Confluentimicrobium sp. EMB200-NS6 Rhodovulum sp. P5 Pannonibacter phragmitetus Labrenzia sp. CP4 Labrenzia aggregata Labrenzia sp. VG12 Labrenzia sp. THAF35 Defluviimonas alba Sulfitobacter sp. AM1-D1 Sulfitobacter sp. SK012 Salipiger profundus Pelagibaca abyssi Thioclava nitratireducens Roseovarius indicus Roseovarius sp. THAF27 Yoonia vestfoldensis Antarctobacter heliothermus Sagittula sp. P11 Thalassococcus sp. S3 Gemmobacter sp. HYN0069 Tabrizicola piscis Silicimonas algicola Sedimentitalea sp. W43 Litoreibacter sp. LN3S51 Paraoceanicella profunda Fluviibacterium aquatile Rhodobacteraceae bacterium G7 Hirschia baltica Novosphingobium aromaticivorans Novosphingobium sp. PP1Y Novosphingobium pentaromativorans Novosphingobium resinovorum Novosphingobium sp. P6W Novosphingobium tardaugens Novosphingobium sp. ABRDHK2 Sphingomonas hengshuiensis Sphingomonas sanxanigenens Sphingobium japonicum Sphingobium sp. YBL2 Sphingobium baderi Sphingobium sp. MI1205 Sphingobium cloacae Sphingobium fuliginis Sphingorhabdus sp. M41 Sphingorhabdus sp. YGSMI21 Blastomonas sp. RAC04 Blastomonas fulva Erythrobacter flavus Altererythrobacter sp. B11 Croceicoccus marinus Porphyrobacter sp. LM 6 Porphyrobacter sp. HT-58-2 Porphyrobacter sp. YT40 Gluconobacter oxydans H24 Granulibacter bethesdensis CGDNIH1 Granulibacter bethesdensis CGDNIH2 Granulibacter bethesdensis NIH3.1 Granulibacter bethesdensis CGDNIH4 Acidiphilium cryptum Acidiphilium multivorum Komagataeibacter nataicola Komagataeibacter saccharivorans Acetobacter pasteurianus IFO 3283-01 Acetobacter pasteurianus IFO 3283-03 Acetobacter pasteurianus IFO 3283-07 Acetobacter pasteurianus IFO 3283-12 Acetobacter pasteurianus IFO 3283-22 Acetobacter pasteurianus IFO 3283-26 Acetobacter pasteurianus IFO 3283-32 Acetobacter pasteurianus 386B Acetobacter senegalensis Acetobacter oryzifermentans Acetobacter aceti Acetobacter pomorum Acetobacter tropicalis Acetobacter ascendens Acetobacter sp. JWB Acetobacter orientalis Stella humosa Stella vacuolata Neokomagataea tanensis Magnetospirillum gryphiswaldense MSR-1 v2 Magnetospirillum gryphiswaldense MSR-1 Azospirillum sp. B510 Azospirillum lipoferum Azospirillum thiophilum Azospirillum sp. CFH 70021 Tistrella mobilis Magnetospira sp. QH-2 Nitrospirillum amazonense Niveispirillum cyanobacteriorum Ferrovibrio terrae Hypericibacter terrae Hypericibacter adhaerens Polymorphum gilvum Phreatobacter cathodiphilus Phreatobacter stygius Acidithiobacillus ferrooxidans ATCC 23270 Acidithiobacillus ferrooxidans ATCC 53993 Acidithiobacillus caldus SM-1 Acidithiobacillus ferrivorans Acidithiobacillus ferridurans Bacillus megaterium DSM 319 Bacillus megaterium WSH-002 Bacillus megaterium NBRC 15308 = ATCC 14581 Bacillus cellulosilyticus Bacillus coagulans 36D1 Bacillus sp. 1NLA3E Bacillus weihaiensis Bacillus sp. Y1 Oceanobacillus sp. 160 Oceanobacillus sp. 143 Halobacillus mangrovi Aeribacillus pallidus Psychrobacillus sp. PB01 Paenibacillus sp. JDR-2 Paenibacillus polymyxa CR1 Paenibacillus mucilaginosus KNP414 Paenibacillus mucilaginosus 3016 Paenibacillus mucilaginosus K02 Paenibacillus terrae Paenibacillus borealis Paenibacillus sp. FSL P4-0081 Paenibacillus sp. FSL R5-0912 Paenibacillus stellifer Paenibacillus sp. FSL R7-0273 Paenibacillus beijingensis Paenibacillus naphthalenovorans Paenibacillus sp. 32O-W Paenibacillus donghaensis Paenibacillus psychroresistens Saccharibacillus sp. ATSA2 Alicyclobacillus acidocaldarius subsp. acidocaldarius DSM 446 Alicyclobacillus acidocaldarius subsp. acidocaldarius Tc-4-1 Kyrpidia tusciae Sporosarcina psychrophila Clostridium bornimense Clostridium tyrobutyricum Ethanoligenens harbinense Cellulosilyticum lentocellum Cellulosilyticum sp. WCF-2 Roseburia intestinalis XB6B4 Roseburia intestinalis M50/1 Lachnoclostridium sp. YL32 Clostridium saccharolyticum WM1 Clostridium bolteae Syntrophobotulus glycolicus Desulfosporosinus orientis Desulfosporosinus meridiei Sulfobacillus acidophilus TPY Sulfobacillus acidophilus DSM 10332 Mycobacterium sp. JS623 Mycobacterium sp. MCS Mycobacterium sp. KMS Mycobacterium sp. JLS Mycobacterium marinum M Mycobacterium marinum E11 Mycobacterium liflandii Mycobacterium kansasii ATCC 12478 Mycobacterium sp. VKM Ac-1817D Mycobacterium sp. EPa45 Mycobacterium sp. NRRL B-3805 Mycobacterium dioxanotrophicus Mycolicibacterium smegmatis MC2 155 Mycolicibacterium smegmatis MC2 155 Mycolicibacterium smegmatis MC2 155 Mycolicibacterium smegmatis INHR1 Mycolicibacterium smegmatis INHR2 Mycolicibacterium vanbaalenii Mycolicibacterium neoaurum Mycolicibacterium goodii Mycolicibacterium fortuitum Mycolicibacterium phlei Mycolicibacterium vaccae Mycolicibacterium litorale Mycolicibacterium rhodesiae Mycolicibacterium thermoresistibile Mycolicibacterium hassiacum Mycobacteroides abscessus ATCC 19977 Mycobacteroides abscessus subsp. massiliense GO 06 Mycobacteroides abscessus subsp. bolletii 50594 Mycobacteroides abscessus subsp. massiliense CCUG 48898 = JCM 15300 Mycobacteroides chelonae Mycobacteroides immunogenum Mycobacterium stephanolepidis Mycobacteroides saopaulense Mycobacteroides salmoniphilum Mycolicibacter sinensis Mycolicibacter terrae Hoyosella subflava Corynebacterium efficiens Corynebacterium glyciniphilum Nocardia farcinica IFM 10152 Nocardia farcinica NCTC11134 Nocardia cyriacigeorgica Nocardia brasiliensis Nocardia nova Nocardia mangyaensis Nocardia seriolae Nocardia sp. CS682 Nocardia otitidiscaviarum Rhodococcus jostii Rhodococcus erythropolis PR4 Rhodococcus erythropolis CCM2595 Rhodococcus erythropolis BG43 Rhodococcus opacus B4 Rhodococcus opacus PD630 Rhodococcus hoagii Rhodococcus pyridinivorans Rhodococcus sp. B7740 Rhodococcus aetherivorans Rhodococcus fascians Rhodococcus sp. WMMA185 Rhodococcus sp. PBTS 2 Rhodococcus ruber Rhodococcus sp. PBTS 1 Rhodococcus qingshengii Rhodococcus sp. p52 Rhodococcus sp. 008 Rhodococcus rhodochrous Rhodococcus biphenylivorans Gordonia bronchialis Gordonia polyisoprenivorans Gordonia sp. KTR9 Gordonia terrae Gordonia rubripertincta Gordonia sp. MMS17-SY073 Gordonia alkanivorans Gordonia sp. 135 Tsukamurella paurometabola Tsukamurella tyrosinosolvens Segniliparus rotundus Dietzia sp. oral taxon 368 Dietzia sp. JS16-p6b Dietzia psychralcaliphila Dietzia lutea Streptomyces avermitilis Streptomyces scabiei Streptomyces pratensis Streptomyces bingchenggensis Streptomyces hygroscopicus subsp. jinggangensis 5008 Streptomyces hygroscopicus subsp. jinggangensis TL01 Streptomyces albus DSM 41398 Streptomyces sp. PAMC 26508 Streptomyces collinus Streptomyces lydicus A02 Streptomyces sp. CdTB01 Streptomyces lincolnensis Streptomyces autolyticus Streptomyces malaysiensis Streptomyces griseorubiginosus Streptomyces rochei Streptomyces lunaelactis Kitasatospora setae Clavibacter michiganensis subsp. michiganensis Clavibacter michiganensis subsp. sepedonicus Clavibacter michiganensis subsp. nebraskensis Clavibacter michiganensis subsp. capsici Rathayibacter festucae Agromyces sp. MF30-A Cryobacterium arcticum Salinibacterium sp. CGMCC 1.16371 Gryllotalpicola sp. 2DFW10M-5 Leucobacter triazinivorans Arthrobacter sp. FB24 Arthrobacter sp. Rue61a Arthrobacter sp. Hiyo8 Arthrobacter sp. ATCC 21022 Arthrobacter sp. QXT-31 Arthrobacter sp. YN Arthrobacter sp. ZXY-2 Arthrobacter crystallopoietes Arthrobacter sp. PGP41 Arthrobacter sp. PAMC25564 Paenarthrobacter aurescens Pseudarthrobacter phenanthrenivorans Pseudarthrobacter sp. NIBRBAC000502771 Kocuria flava Kocuria rosea Sinomonas atrocyanea Isoptericola dokdonensis Cellulosimicrobium cellulans Sanguibacter keddieii Cellulomonas flavigena Cellulomonas sp. PSBB021 Cellulomonas sp. Z28 Cellulomonas sp. H30R-01 Georgenia sp. Z294 Nocardioides daphniae Nocardiopsis gilva Actinomadura sp. WMMB499 Nonomuraea sp. ATCC 55076 Nonomuraea sp. WYY166 Frankia sp. EAN1pec Frankia inefficax Frankia alni Nakamurella multipartita Geodermatophilus obscurus Kineococcus radiotolerans Amycolatopsis mediterranei U32 Amycolatopsis mediterranei S699 Amycolatopsis mediterranei S699 Amycolatopsis mediterranei RB Amycolatopsis orientalis Amycolatopsis japonica Amycolatopsis methanolica Amycolatopsis sp. AA4 Amycolatopsis sp. BJA-103 Amycolatopsis albispora Amycolatopsis sp. YIM 10 Pseudonocardia dioxanivorans Pseudonocardia sp. AL041005-10 Pseudonocardia sp. EC080625-04 Pseudonocardia sp. HH130629-09 Pseudonocardia sp. EC080610-09 Pseudonocardia sp EC080619-01 Pseudonocardia autotrophica Actinosynnema mirum Kibdelosporangium phytohabitans Alloactinosynnema sp. L-07 Micromonospora sp. B006 Micromonospora tulbaghiae Micromonospora sp. HM134 Actinoplanes sp. SE50/110 Actinoplanes friuliensis Actinoplanes sp. SE50 Catenulispora acidiphila Baekduia soli Conexibacter woesei Synechococcus sp. JA-3-3Ab Synechococcus sp. JA-2-3B'a(2-13) Synechococcus sp. PCC 7502 Cyanobium sp. NIES-981 Dactylococcopsis salina Chamaesiphon minutus Leptolyngbya sp. NIES-3755 Leptolyngbya sp. O-77 Leptolyngbya boryana Halomicronema hongdechloris Pseudanabaena sp. PCC 7367 Pseudanabaena sp. ABRG5-3 Acaryochloris marina Gloeocapsa sp. PCC 7428 Microcystis aeruginosa Microcystis panniformis Microcystis viridis Halothece sp. PCC 7418 Euhalothece natronophila Gloeothece citriformis Gloeothece verrucosa Cyanothece sp. PCC 7425 Trichodesmium erythraeum Microcoleus sp. PCC 7113 Oscillatoria nigro-viridis Moorea producens Gloeobacter violaceus Gloeobacter kilaueensis Nostoc sp. PCC 7107 Nostoc sp. PCC 7524 Nostoc sp. NIES-3756 Nostoc flagelliforme Nostoc sp. CENA543 Nostoc sphaeroides Anabaena cylindrica Anabaena sp. YBS01 Cylindrospermum stagnale Calothrix sp. PCC 7507 Calothrix sp. PCC 6303 Calothrix sp. 336/3 Rivularia sp. PCC 7116 Fischerella sp. NIES-3754 Nostocales cyanobacterium HT-58-2 Chroococcidiopsis thermalis Pleurocapsa sp. PCC 7327 Stanieria cyanosphaera Stanieria sp. NIES-3757 Thermomicrobium roseum Anaerolineaceae bacterium oral taxon 439 Lacunisphaera limnophila Nibricoccus aquaticus HZ-65 Opitutaceae bacterium TAV5 Coraliomargarita akajimensis Roseimicrobium sp. ORNL1 Verrucomicrobia bacterium IMCC26134 Verrucomicrobia bacterium S94 Victivallales bacterium CCUG 44730 Sediminispirochaeta smaragdinae Sphaerochaeta globosa Sphaerochaeta pleomorpha Oceanispirochaeta sp. K2 Granulicella tundricola Granulicella mallensis Candidatus Solibacter usitatus Ornithobacterium rhinotracheale DSM 15997 Ornithobacterium rhinotracheale ORT-UMN 88 Halobacterium sp. DL1 Halalkalicoccus jeotgali Haloterrigena daqingensis Natrinema versiforme Halostagnicola larsenii Natronorubrum bangense Nitrososphaera viennensis Candidatus Nitrososphaera evergladensis Candidatus Nitrosocosmicus franklandus | 184% 150% 122% 100% 82% 67% 55% |
