# Supplementary material for: Isolation, identification, and whole-genome sequencing of high-yield protease bacteria from Daqu of ZhangGong Laojiu
Source: PLoS One. 2022 Apr 26;17(4):e0264677. doi: 10.1371/journal.pone.0264677 (PMC9041807; doi:10.1371/journal.pone.0264677)
Supplement: S1 Raw data — (ZIP) [file pone.0264677.s002.zip › Raw data/GDD20120317-1_Bacillus_velezensis_Genome_result/4_Basic_Annot/KEGG/Bac_map/map00998.html]

KEGG PATHWAY: Biosynthesis of various secondary metabolites - part 2 - Reference pathway


|  |  |
| --- | --- |
| **Biosynthesis of various secondary metabolites - part 2 - Reference pathway** |  |

[
Pathway menu
| Organism menu
| Pathway entry
| User data mapping
]

|  |  |
| --- | --- |
| Reference pathway Reference pathway (KO) Reference pathway (EC) Reference pathway (Reaction) -----< Sort below by alphabet >----- Arabidopsis thaliana (thale cress) Arabidopsis lyrata (lyrate rockcress) Capsella rubella Camelina sativa (false flax) Eutrema salsugineum Brassica rapa (field mustard) Brassica napus (rape) Brassica oleracea (wild cabbage) Raphanus sativus (radish) Tarenaya hassleriana (spider flower) Carica papaya (papaya) Citrus sinensis (Valencia orange) Citrus clementina (mandarin orange) Theobroma cacao (cacao) Gossypium raimondii Gossypium hirsutum (upland cotton) Gossypium arboreum Durio zibethinus (durian) Eucalyptus grandis (rose gum) Glycine max (soybean) Glycine soja (wild soybean) Phaseolus vulgaris (common bean) Vigna radiata (mung bean) Vigna angularis (adzuki bean) Vigna unguiculata (cowpea) Cajanus cajan (pigeon pea) Medicago truncatula (barrel medic) Cicer arietinum (chickpea) Lotus japonicus Arachis duranensis Arachis ipaensis Lupinus angustifolius (narrow-leaved blue lupine) Fragaria vesca (woodland strawberry) Rosa chinensis (China rose) Prunus persica (peach) Prunus mume (Japanese apricot) Prunus avium (sweet cherry) Malus domestica (apple) Pyrus x bretschneideri (Chinese white pear) Ziziphus jujuba (Chinese jujube) Cucumis sativus (cucumber) Cucumis melo (muskmelon) Momordica charantia (bitter melon) Cucurbita maxima (winter squash) Cucurbita moschata (crookneck pumpkin) Cucurbita pepo subsp. pepo (vegetable marrow) Ricinus communis (castor bean) Jatropha curcas Hevea brasiliensis (rubber tree) Manihot esculenta (cassava) Populus trichocarpa (black cottonwood) Populus euphratica (Euphrates poplar) Juglans regia (English walnut) Quercus suber (cork oak) Vitis vinifera (wine grape) Solanum lycopersicum (tomato) Solanum pennellii Solanum tuberosum (potato) Capsicum annuum Nicotiana tabacum (common tobacco) Nicotiana sylvestris Nicotiana tomentosiformis Nicotiana attenuata Ipomoea nil (Japanese morning glory) Sesamum indicum (sesame) Olea europaea var. sylvestris (wild olive) Helianthus annuus (common sunflower) Lactuca sativa (garden lettuce) Cynara cardunculus var. scolymus (artichoke) Daucus carota (carrot) Beta vulgaris (sugar beet) Spinacia oleracea (spinach) Chenopodium quinoa (quinoa) Nelumbo nucifera (sacred lotus) Papaver somniferum (opium poppy) Oryza sativa japonica (Japanese rice) (RefSeq) Oryza sativa japonica (Japanese rice) (RAPDB) Oryza brachyantha (malo sina) Brachypodium distachyon Aegilops tauschii (wheat D) Sorghum bicolor (sorghum) Zea mays (maize) Setaria italica (foxtail millet) Phoenix dactylifera (date palm) Elaeis guineensis (African oil palm) Musa acuminata (wild Malaysian banana) Dendrobium catenatum Phalaenopsis equestris Asparagus officinalis (garden asparagus) Amborella trichopoda Selaginella moellendorffii Sclerotinia sclerotiorum Botrytis cinerea Marssonina brunnea Glarea lozoyensis Aspergillus fumigatus Aspergillus clavatus Aspergillus fischeri Aspergillus oryzae Aspergillus flavus Penicillium digitatum Tetrahymena thermophila Paramecium tetraurelia Escherichia coli APEC O1 (APEC) Enterobacter sp. R4-368 Klebsiella quasivariicola Candidatus Hamiltonella defensa (Acyrthosiphon pisum) Raoultella electrica Kosakonia oryzae Kosakonia radicincitans Yersinia enterocolitica LC20 Yersinia rohdei Serratia plymuthica 4Rx13 Serratia plymuthica S13 Serratia rubidaea Serratia ficaria Candidatus Fukatsuia symbiotica Pectobacterium atrosepticum SCRI1043 Pectobacterium atrosepticum JG10-08 Pectobacterium atrosepticum 21A Pectobacterium carotovorum subsp. carotovorum PC1 Pectobacterium carotovorum subsp. carotovorum PCC21 Pectobacterium parmentieri WPP163 Pectobacterium parmentieri RNS08.42.1A Pectobacterium parmentieri SCC3193 Pectobacterium wasabiae Pectobacterium polaris Sodalis glossinidius (Glossina spp.) Sodalis praecaptivus Dickeya aquatica Dickeya sp. NCPPB 569 Brenneria goodwinii Lonsdalea britannica Erwinia amylovora CFBP1430 Erwinia amylovora ATCC 49946 Erwinia pyrifoliae Ep1/96 Erwinia pyrifoliae DSM 12163 Erwinia sp. Ejp617 Erwinia persicina Pantoea ananatis LMG 20103 Pantoea ananatis LMG 5342 Pantoea ananatis AJ13355 Pantoea ananatis PA13 Pantoea eucalypti Mixta gaviniae Tatumella ptyseos Photorhabdus laumondii subsp. laumondii TTO1 Photorhabdus laumondii subsp. laumondii DSPV002N Proteus mirabilis HI4320 Proteus mirabilis BB2000 Proteus mirabilis CYPV1 Proteus vulgaris Proteus sp. CD3 Proteus cibarius Proteus columbae Xenorhabdus bovienii CS03 Xenorhabdus doucetiae Providencia stuartii MRSN 2154 Providencia stuartii ATCC 33672 Providencia stuartii BE2467 Providencia rettgeri Providencia alcalifaciens Providencia heimbachae Providencia sp. WCHPHu000369 Providencia rustigianii Providencia vermicola Edwardsiella ictaluri Pragia fontium Pragia sp. CF-458 Pasteurella multocida subsp. multocida Pm70 Pasteurella multocida subsp. multocida HN06 Pasteurella multocida subsp. multocida 3480 Pasteurella multocida 36950 Pasteurella multocida ATCC 43137 Rodentibacter heylii Xanthomonas campestris pv. campestris ATCC 33913 Xanthomonas campestris pv. campestris 8004 Xanthomonas campestris pv. campestris B100 Xanthomonas campestris pv. raphani Xanthomonas campestris pv. vesicatoria Xanthomonas axonopodis pv. citrumelo F1 Xanthomonas citri pv. citri 306 Xanthomonas citri subsp. citri Aw12879 Xanthomonas citri subsp. citri A306 Xanthomonas citri subsp. citri UI6 Xanthomonas citri pv. citri UI7 Xanthomonas citri pv. citri NT17 Xanthomonas citri pv. citri MN10 Xanthomonas citri pv. citri MN11 Xanthomonas citri pv. citri MN12 Xanthomonas citri pv. citri mf20 Xanthomonas citri pv. fuscans Xanthomonas axonopodis Xac29-1 Xanthomonas oryzae pv. oryzae MAFF 311018 Xanthomonas oryzae pv. oryzae KACC 10331 Xanthomonas oryzae pv. oryzae PXO99A Xanthomonas oryzae pv. oryzae PXO86 Xanthomonas oryzae pv. oryzicola BLS256 Xanthomonas oryzae pv. oryzicola CFBP7342 Xanthomonas albilineans Xanthomonas sacchari Xanthomonas gardneri Xanthomonas vesicatoria Xanthomonas perforans Xanthomonas hortorum Xanthomonas phaseoli Xanthomonas vasicola Xanthomonas sp. ISO98C4 Xanthomonas arboricola Xanthomonas hyacinthi Lysobacter gummosus Vibrio nigripulchritudo Vibrio gazogenes Aliivibrio fischeri ES114 Grimontia hollisae Paraphotobacterium marinum Pseudomonas aeruginosa PA7 Pseudomonas citronellolis Pseudomonas putida KT2440 Pseudomonas putida F1 Pseudomonas putida W619 Pseudomonas putida H8234 Pseudomonas fulva Pseudomonas soli Pseudomonas oryzihabitans Pseudomonas syringae pv. tomato DC3000 Pseudomonas syringae pv. syringae B728a Pseudomonas syringae CC1557 Pseudomonas savastanoi pv. phaseolicola 1448A Pseudomonas amygdali Pseudomonas avellanae Pseudomonas protegens Pf-5 Pseudomonas protegens CHA0 Pseudomonas protegens Cab57 Pseudomonas fluorescens SBW25 Pseudomonas fluorescens F113 Pseudomonas fluorescens A506 Pseudomonas poae Pseudomonas trivialis Pseudomonas corrugata Pseudomonas veronii Pseudomonas azotoformans Pseudomonas entomophila Pseudomonas brassicacearum subsp. brassicacearum NFM421 Pseudomonas brassicacearum DF41 Pseudomonas sp. UW4 Pseudomonas sp. TKP Pseudomonas chlororaphis PA23 Pseudomonas chlororaphis PCL1606 Pseudomonas chlororaphis subsp. aurantiaca Pseudomonas fragi Pseudomonas sp. StFLB209 Pseudomonas sp. MRSN12121 Pseudomonas sp. Os17 Pseudomonas psychrotolerans Azotobacter vinelandii DJ Azotobacter vinelandii CA Azotobacter vinelandii CA6 Azotobacter chroococcum Psychrobacter sp. PRwf-1 Psychrobacter alimentarius Psychrobacter sp. YP14 Psychrobacter sp. PAMC27889 Acinetobacter baumannii ATCC 17978 Acinetobacter baumannii AYE Acinetobacter baumannii AB0057 Acinetobacter baumannii AB307-0294 Acinetobacter baumannii BJAB0715 Acinetobacter baumannii ZW85-1 Acinetobacter baumannii AB030 Acinetobacter baumannii AB031 Acinetobacter baumannii LAC-4 Acinetobacter pittii Acinetobacter nosocomialis Acinetobacter lactucae Acinetobacter calcoaceticus Acinetobacter oleivorans Acinetobacter baylyi Acinetobacter equi Acinetobacter sp. DUT-2 Acinetobacter haemolyticus Acinetobacter soli Acinetobacter defluvii Acinetobacter wuhouensis Acinetobacter ursingii Shewanella denitrificans Shewanella sediminis Colwellia psychrerythraea Thalassotalea sp. PS06 Pseudoalteromonas piratica OCN003 Pseudoalteromonas spongiae Pseudoalteromonas tunicata Marinobacter sp. Arc7-DN-1 Alteromonas sp. RKMC-009 Glaciecola nitratireducens Moritella yayanosii Oceanicoccus sagamiensis Candidatus Rickettsiella viridis Legionella pneumophila 2300/99 Alcoy (serogroup 1) Legionella longbeachae Legionella sainthelensi Francisella tularensis subsp. tularensis SCHU S4 NR-28534 Francisella tularensis subsp. tularensis NE061598 Francisella tularensis subsp. tularensis TI0902 Francisella tularensis subsp. tularensis TIGB03 Francisella tularensis subsp. holarctica LVS Francisella tularensis subsp. holarctica OSU18 Francisella tularensis subsp. holarctica FTNF002-00 Francisella tularensis subsp. holarctica F92 Francisella tularensis subsp. holarctica FSC200 Francisella tularensis subsp. holarctica PHIT-FT049 Francisella tularensis subsp. holarctica FTT\_1 Francisella tularensis subsp. holarctica VT68 Francisella tularensis subsp. holarctica 425 Francisella tularensis subsp. mediasiatica FSC147 Francisella tularensis subsp. novicida U112 Francisella tularensis subsp. novicida U112 Francisella tularensis subsp. novicida F6168 Francisella tularensis subsp. novicida DPG 3A-IS Francisella cf. novicida Fx1 Francisella hispaniensis 3523 Francisella hispaniensis FSC454 Francisella philomiragia subsp. philomiragia ATCC 25017 Francisella philomiragia O#319-029 Francisella philomiragia O#319-036 Francisella philomiragia O#319-067 Francisella philomiragia GA01-2794 Francisella philomiragia GA01-2801 Francisella salina Francisella sp. FSC1006 Francisella persica Francisella halioticida Francisella uliginis Francisella sp. MA067296 Francisella frigiditurris Francisella adeliensis Francisella marina Francisella opportunistica Piscirickettsia salmonis Nitrosococcus oceani Nitrosococcus halophilus Nitrosococcus watsonii Nitrosococcus wardiae Candidatus Nitrosoglobus terrae Spiribacter salinus Spiribacter sp. 2438 Chromohalobacter salexigens Halomonas sp. R57-5 Halomonas olivaria Halomonas sulfidaeris Halomonas meridiana Zymobacter palmae Gynuella sunshinyii Frischella perrara Immundisolibacter cernigliae Salinisphaera sp. LB1 Vitreoscilla sp. C1 Neisseriaceae bacterium DSM 100970 Chromobacterium violaceum Chromobacterium sp. ATCC 53434 Chromobacterium sp. IIBBL 112-1 Chromobacterium sp. IIBBL 274-1 Ralstonia solanacearum GMI1000 Ralstonia solanacearum PSI07 Ralstonia solanacearum Po82 Ralstonia solanacearum CMR15 Ralstonia solanacearum FQY\_4 Ralstonia solanacearum UY031 Ralstonia pickettii DTP0602 Ralstonia pseudosolanacearum Cupriavidus pinatubonensis JMP134 Cupriavidus metallidurans Cupriavidus basilensis Cupriavidus gilardii Cupriavidus sp. USMAHM13 Cupriavidus malaysiensis Cupriavidus sp. USMAA2-4 Cupriavidus pauculus Cupriavidus oxalaticus Burkholderia mallei ATCC 23344 Burkholderia mallei SAVP1 Burkholderia mallei NCTC 10229 Burkholderia mallei NCTC 10247 Burkholderia mallei 23344 Burkholderia mallei 6 Burkholderia mallei BMQ Burkholderia mallei 2000031063 Burkholderia mallei FMH 23344 Burkholderia mallei NCTC 10247 Burkholderia mallei 2002734299 Burkholderia pseudomallei K96243 Burkholderia pseudomallei 1710b Burkholderia pseudomallei 1106a Burkholderia pseudomallei 668 Burkholderia pseudomallei MSHR305 Burkholderia pseudomallei MSHR511 Burkholderia pseudomallei MSHR146 Burkholderia pseudomallei MSHR520 Burkholderia pseudomallei 1026b Burkholderia pseudomallei BPC006 Burkholderia pseudomallei NCTC 13179 Burkholderia pseudomallei HBPUB10134a Burkholderia pseudomallei NAU35A-3 Burkholderia pseudomallei A79A Burkholderia pseudomallei TSV202 Burkholderia thailandensis E264 Burkholderia thailandensis 2002721723 Burkholderia thailandensis E444 Burkholderia thailandensis H0587 Burkholderia thailandensis MSMB121 Burkholderia thailandensis MSMB59 Burkholderia thailandensis E254 Burkholderia thailandensis USAMRU Malaysia #20 Burkholderia thailandensis 2003015869 Burkholderia thailandensis 2002721643 Burkholderia oklahomensis E0147 Burkholderia oklahomensis C6786 Burkholderia sp. BDU6 Burkholderia vietnamiensis G4 Burkholderia vietnamiensis LMG 10929 Burkholderia lata Burkholderia cenocepacia AU1054 Burkholderia cenocepacia HI2424 Burkholderia cenocepacia MC0-3 Burkholderia cenocepacia J2315 Burkholderia cenocepacia DDS 22E-1 Burkholderia cenocepacia DWS 37E-2 Burkholderia cenocepacia H111 Burkholderia ambifaria AMMD Burkholderia ambifaria MC40-6 Burkholderia multivorans ATCC 17616 (JGI) Burkholderia multivorans DDS 15A-1 Burkholderia multivorans ATCC BAA-247 Burkholderia cepacia GG4 Burkholderia cepacia DDS 7H-2 Burkholderia cepacia ATCC 25416 Burkholderia dolosa Burkholderia pyrrocinia Burkholderia contaminans Burkholderia ubonensis Burkholderia diffusa Burkholderia latens Burkholderia territorii Burkholderia seminalis Burkholderia pseudomultivorans Burkholderia metallica Burkholderia stagnalis Burkholderia stabilis Burkholderia glumae BGR1 Burkholderia glumae LMG 2196 = ATCC 33617 Burkholderia sp. CCGE1001 Burkholderia sp. CCGE1003 Burkholderia gladioli BSR3 Burkholderia gladioli ATCC 10248 Burkholderia sp. YI23 Burkholderia sp. KJ006 Burkholderia insecticola Burkholderia sp. RPE67 Burkholderia sp. 2002721687 Burkholderia sp. HB1 Burkholderia plantarii PG1 Burkholderia plantarii ATCC 43733 Burkholderia sp. Bp5365 Burkholderia sp. OLGA172 Burkholderia sp. PAMC 26561 Burkholderia sp. PAMC 28687 Paraburkholderia xenovorans LB400 Paraburkholderia xenovorans LB400 Paraburkholderia phymatum Paraburkholderia sp. CCGE1002 Paraburkholderia phenoliruptrix Paraburkholderia phytofirmans Paraburkholderia fungorum Paraburkholderia caribensis Paraburkholderia sprentiae Paraburkholderia sp. SOS3 Paraburkholderia aromaticivorans Paraburkholderia hospita Paraburkholderia terrae Paraburkholderia graminis Paraburkholderia caledonica Paraburkholderia terricola Paraburkholderia caffeinilytica Paraburkholderia rhizoxinica Pandoraea pnomenusa 3kgm Pandoraea pnomenusa RB38 Pandoraea pnomenusa DSM 16536 Pandoraea pnomenusa Pandoraea pulmonicola Pandoraea sputorum Pandoraea apista Pandoraea vervacti Pandoraea oxalativorans Pandoraea thiooxydans Pandoraea faecigallinarum Pandoraea norimbergensis Pandoraea sp. XY-2 Pandoraea fibrosis Caballeronia sp. SBC2 Bordetella parapertussis Bpp5 Bordetella parapertussis 12822 Bordetella bronchiseptica 253 Bordetella bronchiseptica RB50 Bordetella bronchiseptica MO149 Bordetella bronchiseptica S798 Bordetella holmesii ATCC 51541 Bordetella holmesii 44057 Bordetella hinzii Bordetella bronchialis Bordetella flabilis Bordetella pseudohinzii Bordetella sp. H567 Bordetella genomosp. 13 Achromobacter xylosoxidans A8 Achromobacter xylosoxidans NH44784-1996 Achromobacter xylosoxidans NBRC 15126 = ATCC 27061 Achromobacter xylosoxidans NCTC10807 Achromobacter denitrificans Achromobacter insolitus Achromobacter sp. AONIH1 Achromobacter sp. B7 Pusillimonas sp. ye3 Advenella kashmirensis Castellaniella defragrans Alcaligenes faecalis ZD02 Alcaligenes faecalis JQ135 Alcaligenes aquatilis Paenalcaligenes hominis Pigmentiphaga sp. H8 Pigmentiphaga aceris Rhodoferax saidenbachensis Rhodoferax antarcticus Rhodoferax sp. CHu59-6-5 Acidovorax citrulli Acidovorax avenae Acidovorax sp. RAC01 Acidovorax carolinensis NA2 Acidovorax carolinensis P4 Acidovorax carolinensis P3 Delftia acidovorans Delftia sp. Cs1-4 Delftia tsuruhatensis Delftia sp. HK171 Comamonas testosteroni TK102 Hydrogenophaga sp. PBC Hydrogenophaga sp. BPS33 Melaminivora sp. SC2-9 Janthinobacterium sp. B9-8 Massilia sp. WG5 Massilia violaceinigra Massilia albidiflava Massilia umbonata Massilia flava Noviherbaspirillum sp. UKPF54 Roseateles depolymerans Sulfuritalea hydrogenivorans Methylophilus sp. TWE2 Azoarcus sp. BH72 Azoarcus olearius Helicobacter cinaedi PAGU611 Helicobacter cinaedi CCUG 18818 = ATCC BAA-847 Campylobacter jejuni subsp. jejuni 81116 Campylobacter hyointestinalis Campylobacter hepaticus Arcobacter nitrofigilis Nitratifractor salsuginis Sulfurovum sp. NBC37-1 Pelobacter carbinolicus Pelobacter propionicus Pelobacter acetylenicus Desulfurivibrio alkaliphilus Desulfatibacillum alkenivorans Myxococcus xanthus Myxococcus fulvus Myxococcus stipitatus Myxococcus hansupus Corallococcus coralloides Stigmatella aurantiaca Archangium gephyra Melittangium boletus Cystobacter fuscus Sorangium cellulosum So ce56 Sorangium cellulosum So0157-2 Labilithrix luteola Minicystis rosea Desulfobacca acetoxidans Bacteriovorax stolpii Rickettsia slovaca 13-B Rickettsia slovaca D-CWPP Mesorhizobium japonicum MAFF 303099 Mesorhizobium loti NZP2037 Mesorhizobium amorphae Mesorhizobium sp. B7 Mesorhizobium sp. Pch-S Mesorhizobium huakuii Chelativorans sp. BNC1 Phyllobacterium zundukense Sinorhizobium meliloti AK83 Sinorhizobium meliloti Rm41 Sinorhizobium medicae Sinorhizobium fredii NGR234 Sinorhizobium fredii USDA 257 Sinorhizobium americanum Ensifer adhaerens Casida A Ensifer alkalisoli Agrobacterium radiobacter Agrobacterium vitis Agrobacterium sp. RAC06 Rhizobium etli CIAT 652 Rhizobium etli bv. mimosae Mim1 Rhizobium etli bv. phaseoli IE4803 Rhizobium sp. IE4771 Rhizobium leguminosarum bv. viciae 3841 Rhizobium leguminosarum bv. trifolii WSM2304 Rhizobium leguminosarum bv. trifolii WSM1325 Rhizobium leguminosarum bv. trifolii WSM1689 Rhizobium leguminosarum bv. trifolii CB782 Rhizobium tropici Rhizobium favelukesii Rhizobium sp. N1341 Rhizobium phaseoli Rhizobium sp. NT-26 Rhizobium sp. N731 Rhizobium esperanzae Rhizobium jaguaris Rhizobium grahamii Rhizobium acidisoli Neorhizobium galegae bv. officinalis bv. officinalis HAMBI 1141 Neorhizobium galegae bv. orientalis HAMBI 540 Shinella sp. HZN7 Ochrobactrum sp. A44 Bradyrhizobium japonicum USDA 6 Bradyrhizobium japonicum E109 Bradyrhizobium sp. S23321 Bradyrhizobium oligotrophicum Bradyrhizobium sp. CCGE-LA001 Bradyrhizobium sp. BF49 Bradyrhizobium icense Bradyrhizobium sp. ORS 285 Bradyrhizobium sp. SK17 Bradyrhizobium ottawaense Bradyrhizobium amphicarpaeae Bradyrhizobium guangdongense Bradyrhizobium guangzhouense Bradyrhizobium symbiodeficiens Rhodopseudomonas palustris BisB18 Bosea sp. RAC05 Bosea sp. F3-2 Bartonella apis Starkeya novella Ancylobacter pratisalsi Methylorubrum extorquens CM4 Methylorubrum populi Methylobacterium sp. 4-46 Methylobacterium sp. AMS5 Methylobacterium aquaticum Rhodoplanes sp. Z2-YC6860 Methylocystis parvus Pseudorhodoplanes sinuspersici Nordella sp. HKS 07 Caulobacter vibrioides CB15 Caulobacter vibrioides NA1000 Caulobacter sp. K31 Caulobacter segnis Brevundimonas subvibrioides Brevundimonas sp. LM2 Brevundimonas sp. MF30-B Ruegeria sp. THAF33 Epibacterium mobile Rhodobacter sphaeroides ATCC 17029 Paracoccus yeei Pseudovibrio sp. FO-BEG1 Phaeobacter porticola Phaeobacter piscinae Phaeobacter sp. LSS9 Octadecabacter antarcticus Confluentimicrobium sp. EMB200-NS6 Roseovarius indicus Roseovarius sp. MME-070 Thalassococcus sp. S3 Silicimonas algicola Boseongicola sp. CCM32 Stappia indica Pseudohalocynthiibacter aestuariivivens Rhodobacteraceae bacterium SH-1 Hirschia baltica Novosphingobium aromaticivorans Novosphingobium sp. PP1Y Novosphingobium pentaromativorans Novosphingobium resinovorum Novosphingobium sp. THN1 Novosphingobium sp. ABRDHK2 Sphingomonas sp. MM-1 Sphingomonas hengshuiensis Sphingomonas sanxanigenens Sphingomonas panacis Sphingomonas koreensis Sphingomonas sp. LM7 Sphingomonas sp. KC8 Sphingomonas sp. Cra20 Sphingobium japonicum Sphingobium chlorophenolicum Sphingobium sp. SYK-6 Sphingobium sp. YBL2 Sphingobium sp. MI1205 Sphingobium sp. EP60837 Sphingobium sp. RAC03 Sphingobium indicum Sphingobium hydrophobicum Sphingobium sp. YG1 Sphingobium fuliginis Sphingorhabdus sp. M41 Sphingorhabdus sp. SMR4y Sphingorhabdus sp. YGSMI21 Blastomonas sp. RAC04 Blastomonas fulva Rhizorhabdus dicambivorans Sphingosinicella sp. BN140058 Erythrobacter atlanticus Altererythrobacter atlanticus Altererythrobacter sp. B11 Croceicoccus marinus Roseomonas gilardii Roseomonas sp. FDAARGOS\_362 Roseomonas mucosa Stella humosa Stella vacuolata Pararhodospirillum photometricum Magnetospirillum sp. XM-1 Azospirillum sp. B510 Azospirillum sp. CFH 70021 Tistrella mobilis Nitrospirillum amazonense Niveispirillum cyanobacteriorum Hypericibacter terrae Hypericibacter adhaerens Alpha proteobacterium HIMB59 Emcibacter congregatus Phreatobacter stygius Candidatus Puniceispirillum marinum Bacillus subtilis subsp. subtilis 168 Bacillus subtilis subsp. subtilis RO-NN-1 Bacillus subtilis subsp. subtilis BSP1 Bacillus subtilis subsp. subtilis 6051-HGW Bacillus subtilis subsp. subtilis BAB-1 Bacillus subtilis subsp. subtilis AG1839 Bacillus subtilis subsp. subtilis JH642 Bacillus subtilis subsp. subtilis OH 131.1 Bacillus subtilis subsp. spizizenii W23 Bacillus subtilis subsp. spizizenii TU-B-10 Bacillus subtilis subsp. natto BEST195 Bacillus subtilis BSn5 Bacillus subtilis QB928 Bacillus subtilis XF-1 Bacillus subtilis PY79 Bacillus licheniformis ATCC 14580 Bacillus licheniformis DSM 13 = ATCC 14580 Bacillus paralicheniformis Bacillus velezensis FZB42 Bacillus velezensis CAU B946 Bacillus velezensis YAU B9601-Y2 Bacillus velezensis AS43.3 Bacillus velezensis UCMB5036 Bacillus velezensis UCMB5033 Bacillus velezensis UCMB5113 Bacillus velezensis NAU-B3 Bacillus velezensis TrigoCor1448 Bacillus velezensis SQR9 Bacillus velezensis JS25R Bacillus amyloliquefaciens DSM 7 Bacillus amyloliquefaciens TA208 Bacillus amyloliquefaciens LL3 Bacillus amyloliquefaciens XH7 Bacillus amyloliquefaciens Y2 Bacillus amyloliquefaciens IT-45 Bacillus amyloliquefaciens CC178 Bacillus amyloliquefaciens LFB112 Bacillus atrophaeus Bacillus vallismortis Bacillus anthracis Ames Bacillus anthracis Ames Ancestor Bacillus anthracis Sterne Bacillus anthracis CDC 684 Bacillus anthracis A0248 Bacillus anthracis H9401 Bacillus anthracis A16 Bacillus anthracis A16R Bacillus anthracis SVA11 Bacillus anthracis HYU01 Bacillus anthracis Vollum Bacillus cereus ATCC 10987 Bacillus cereus AH187 Bacillus cereus B4264 Bacillus cereus AH820 Bacillus cereus G9842 Bacillus cereus Q1 Bacillus cereus 03BB102 Bacillus cereus NC7401 Bacillus cereus F837/76 Bacillus cereus FRI-35 Bacillus cereus FT9 Bacillus cytotoxicus Bacillus thuringiensis Al Hakam Bacillus thuringiensis serovar kurstaki HD73 Bacillus thuringiensis serovar kurstaki YBT-1520 Bacillus thuringiensis serovar kurstaki HD-1 Bacillus thuringiensis serovar chinensis CT-43 Bacillus thuringiensis Bt407 Bacillus thuringiensis HD-771 Bacillus thuringiensis HD-789 Bacillus thuringiensis serovar thuringiensis IS5056 Bacillus thuringiensis YBT-1518 Bacillus thuringiensis HD1011 Bacillus thuringiensis YWC2-8 Bacillus mycoides KBAB4 Bacillus toyonensis Bacillus pseudomycoides 219298 Bacillus bombysepticus Bacillus wiedmannii Bacillus clausii Bacillus pumilus SAFR-032 Bacillus pumilus MTCC B6033 Bacillus pumilus SH-B9 Bacillus pseudofirmus Bacillus megaterium QM B1551 Bacillus megaterium DSM 319 Bacillus megaterium WSH-002 Bacillus megaterium NBRC 15308 = ATCC 14581 Bacillus coagulans 2-6 Bacillus coagulans 36D1 Bacillus sp. JS Bacillus sp. 1NLA3E Bacillus lehensis Bacillus sp. WP8 Bacillus sp. Pc3 Bacillus sp. BH072 Bacillus sp. YP1 Bacillus sp. BS34A Bacillus sp. LM 4-2 Bacillus filamentosus Bacillus simplex Bacillus glycinifermentans Bacillus flexus Bacillus gibsonii Bacillus xiamenensis Bacillus kochii Bacillus altitudinis Bacillus sp. SDLI1 Bacillus muralis Bacillus safensis Bacillus intestinalis Bacillus thermoamylovorans Oceanobacillus iheyensis Oceanobacillus sp. 160 Lysinibacillus sphaericus Lysinibacillus fusiformis Lysinibacillus sp. YS11 Lysinibacillus sp. B2A1 Lysinibacillus sp. SGAir0095 Terribacillus goriensis Virgibacillus necropolis Virgibacillus sp. MSP4-1 Salicibibacter halophilus Staphylococcus muscae Staphylococcus fleurettii Macrococcus canis Brochothrix thermosphacta Exiguobacterium sp. AT1b Brevibacillus laterosporus Brevibacillus sp. 7WMA2 Paenibacillus sp. Y412MC10 Paenibacillus polymyxa E681 Paenibacillus polymyxa SC2 Paenibacillus polymyxa M1 Paenibacillus polymyxa CR1 Paenibacillus polymyxa SQR-21 Paenibacillus polymyxa Sb3-1 Paenibacillus terrae Paenibacillus larvae Paenibacillus sabinae Paenibacillus borealis Paenibacillus odorifer Paenibacillus sp. FSL P4-0081 Paenibacillus sp. FSL R5-0912 Paenibacillus sp. FSL R7-0273 Paenibacillus sp. FSL R7-0331 Paenibacillus sp. FSL H7-0357 Paenibacillus sp. FSL H7-0737 Paenibacillus beijingensis Paenibacillus sp. IHBB 10380 Paenibacillus riograndensis Paenibacillus peoriae Paenibacillus naphthalenovorans Paenibacillus bovis Paenibacillus xylanexedens Paenibacillus donghaensis Paenibacillus ihbetae Paenibacillus kribbensis Paenibacillus sp. IHB B 3084 Paenibacillus vortex Paenibacillus lautus Paenibacillus psychroresistens Cohnella candidum Cohnella sp. HS21 Tumebacillus avium Tumebacillus algifaecis Solibacillus silvestris StLB046 Solibacillus silvestris DSM 12223 Planococcus sp. PAMC 21323 Planococcus kocurii Planococcus rifietoensis Planococcus versutus Planococcus antarcticus Planococcus donghaensis Planococcus halocryophilus Planococcus maritimus Planococcus plakortidis Planococcus faecalis Planococcus sp. MB-3u-03 Jeotgalibacillus malaysiensis Sporosarcina psychrophila Rummeliibacillus stabekisii Paenisporosarcina antarctica Planomicrobium glaciei Planomicrobium sp. Y50 Streptococcus pneumoniae OXC141 (serotype 3) Streptococcus pneumoniae SPN034156 (serotype 3) Streptococcus pneumoniae SPN034183 (serotype 3) Streptococcus pneumoniae SPN994038 (serotype 3) Streptococcus pneumoniae SPN994039 (serotype 3) Lactobacillus sp. wkB8 Lactobacillus heilongjiangensis Lactobacillus parabuchneri Lactobacillus allii Lactobacillus farciminis Leuconostoc citreum Leuconostoc lactis Leuconostoc garlicum Weissella paramesenteroides Weissella hellenica Aerococcus urinaeequi Aerococcus sanguinicola Carnobacterium inhibens Clostridium tetani E88 Clostridium tetani 12124569 Clostridium botulinum H04402 065 Clostridium beijerinckii NCIMB 8052 Clostridium beijerinckii ATCC 35702 Clostridium beijerinckii NCIMB 14988 Clostridium saccharoperbutylacetonicum Clostridium saccharobutylicum Clostridium butyricum Clostridium argentinense Clostridium diolis Ruminococcus albus Ruminococcus champanellensis Cellulosilyticum lentocellum Roseburia intestinalis XB6B4 Blautia producta Lachnoclostridium sp. YL32 Paeniclostridium sordellii Oscillibacter valericigenes Aminipila butyrica Ammonifex degensii Halanaerobium hydrogeniformans Gottschalkia acidurici Veillonella parvula Veillonella atypica Veillonella rodentium Veillonella dispar Megasphaera elsdenii Megasphaera hexanoica Selenomonas sputigena Selenomonas sp. oral taxon 478 Selenomonas sp. oral taxon 136 Selenomonas sp. oral taxon 920 Megamonas hypermegale Megamonas funiformis Pelosinus sp. UFO1 Pelosinus fermentans Candidatus Izimaplasma sp. HR1 Mycobacterium avium subsp. paratuberculosis K-10 Mycobacterium avium subsp. paratuberculosis MAP4 Mycobacterium avium subsp. paratuberculosis E1 Mycobacterium avium subsp. paratuberculosis E93 Mycobacterium avium 104 Mycobacterium intracellulare MOTT-02 Mycobacterium intracellulare ATCC 13950 Mycobacterium intracellulare subsp. intracellulare Mycobacterium intracellulare subsp. yongonense Mycobacterium paraintracellulare Mycobacterium chimaera Mycobacterium marseillense Mycobacterium sp. JS623 Mycobacterium sp. MCS Mycobacterium sp. KMS Mycobacterium sp. JLS Mycobacterium marinum M Mycobacterium marinum E11 Mycobacterium sp. MOTT36Y Mycobacterium kansasii ATCC 12478 Mycobacterium shigaense Mycolicibacterium smegmatis MC2 155 Mycolicibacterium smegmatis MC2 155 Mycolicibacterium smegmatis INHR1 Mycolicibacterium smegmatis INHR2 Mycolicibacterium gilvum PYR-GCK Mycolicibacterium gilvum Spyr1 Mycolicibacterium chubuense Mycolicibacterium goodii Mycolicibacterium phlei Mycolicibacterium vaccae Mycolicibacterium litorale Mycolicibacterium rhodesiae Mycolicibacterium hassiacum Mycobacteroides abscessus ATCC 19977 Mycobacteroides abscessus subsp. massiliense GO 06 Mycobacteroides abscessus subsp. bolletii 50594 Mycobacteroides abscessus subsp. massiliense CCUG 48898 = JCM 15300 Mycobacteroides immunogenum Mycobacteroides saopaulense Mycolicibacillus koreensis Nocardia cyriacigeorgica Nocardia brasiliensis Nocardia nova Nocardia seriolae Nocardia terpenica Nocardia sp. CS682 Nocardia otitidiscaviarum Rhodococcus erythropolis PR4 Rhodococcus erythropolis CCM2595 Rhodococcus erythropolis BG43 Rhodococcus opacus B4 Rhodococcus opacus PD630 Rhodococcus pyridinivorans Rhodococcus aetherivorans Rhodococcus qingshengii Rhodococcus sp. 008 Rhodococcus rhodochrous Gordonia terrae Gordonia rubripertincta Gordonia sp. MMS17-SY073 Dietzia lutea Streptomyces coelicolor Streptomyces avermitilis Streptomyces scabiei Streptomyces violaceusniger Streptomyces cattleya NRRL 8057 = DSM 46488 Streptomyces bingchenggensis Streptomyces hygroscopicus subsp. jinggangensis 5008 Streptomyces hygroscopicus subsp. jinggangensis TL01 Streptomyces davaonensis Streptomyces albus DSM 41398 Streptomyces collinus Streptomyces rapamycinicus Streptomyces albulus NK660 Streptomyces albulus ZPM Streptomyces lydicus A02 Streptomyces lydicus 103 Streptomyces sp. Mg1 Streptomyces sp. CNQ-509 Streptomyces sp. CdTB01 Streptomyces sp. SAT1 Streptomyces clavuligerus Streptomyces griseochromogenes Streptomyces sp. CCM\_MD2014 Streptomyces alfalfae Streptomyces violaceoruber Streptomyces gilvosporeus Streptomyces laurentii Streptomyces alboflavus Streptomyces albireticuli Streptomyces sp. MOE7 Streptomyces formicae Streptomyces rochei Kitasatospora setae Kitasatospora albolonga Kitasatospora aureofaciens Streptacidiphilus sp. DSM 106435 Clavibacter michiganensis subsp. michiganensis Clavibacter michiganensis subsp. nebraskensis Clavibacter michiganensis subsp. insidiosus Clavibacter michiganensis subsp. capsici Microbacterium sp. CGR1 Microbacterium sp. XT11 Rathayibacter toxicus 70137 Rathayibacter toxicus WAC3373 Curtobacterium sp. MR\_MD2014 Arthrobacter sp. Rue61a Arthrobacter sp. LS16 Arthrobacter sp. Hiyo8 Arthrobacter sp. ATCC 21022 Arthrobacter sp. YN Arthrobacter sp. ZXY-2 Arthrobacter sp. PGP41 Psychromicrobium lacuslunae Paenarthrobacter aurescens Sinomonas atrocyanea Cellulomonas sp. H30R-01 Intrasporangium calvum Tessaracoccus aquimaris Aeromicrobium sp. A1-2 Aeromicrobium sp. MF47 Actinomadura sp. WMMB499 Streptosporangium roseum Nonomuraea sp. ATCC 55076 Frankia casuarinae Frankia alni Nakamurella sp. s14-144 Modestobacter marinus Saccharopolyspora erythraea Saccharomonospora viridis Amycolatopsis orientalis Amycolatopsis japonica Amycolatopsis methanolica Amycolatopsis sp. AA4 Amycolatopsis sp. BJA-103 Amycolatopsis albispora Amycolatopsis sp. YIM 10 Pseudonocardia dioxanivorans Pseudonocardia sp. EC080625-04 Pseudonocardia sp. EC080610-09 Pseudonocardia sp EC080619-01 Pseudonocardia sp. HH130630-07 Pseudonocardia autotrophica Actinosynnema pretiosum Actinosynnema mirum Saccharothrix espanaensis Saccharothrix syringae Kutzneria albida Kibdelosporangium phytohabitans Actinoalloteichus sp. AHMU CJ021 Alloactinosynnema sp. L-07 Salinispora arenicola Micromonospora tulbaghiae Stackebrandtia nassauensis Actinomyces radicidentis Flaviflexus salsibiostraticola Actinopolyspora erythraea Actinobacteria bacterium Baekduia soli Ilumatobacter coccineus Olsenella uli Synechocystis sp. PCC 6803 Synechocystis sp. PCC 6803 GT-S Synechocystis sp. PCC 6803 GT-I Synechocystis sp. PCC 6803 PCC-N Synechocystis sp. PCC 6803 PCC-P Synechocystis sp. IPPAS B-1465 Acaryochloris marina Microcystis aeruginosa Microcystis panniformis Microcystis viridis Rippkaea orientalis PCC 8801 Rippkaea orientalis PCC 8802 Gloeothece citriformis Arthrospira platensis Planktothrix agardhii Moorea producens Nostoc sp. PCC 7107 Anabaena sp. 90 Anabaena cylindrica Anabaena sp. WA102 Nodularia spumigena Dolichospermum sp. UHCC 0315A Nostocales cyanobacterium HT-58-2 Anaerolinea thermophila Caldilinea aerophila Ktedonobacterales bacterium SCAWS-G2 Deinococcus deserti Deinococcus ficus Chthonomonas calidirosea Opitutus terrae Lacunisphaera limnophila Fuerstia marisgermanicae Gimesia maris Gimesia sp. E7 Singulisphaera acidiphila Treponema denticola Treponema brennaborense Treponema azotonutricium Treponema primitia Treponema caldarium Treponema putidum Treponema phagedenis Sediminispirochaeta smaragdinae Spirochaeta thermophila DSM 6192 Sphaerochaeta globosa Sphaerochaeta pleomorpha Leptospira borgpetersenii JB197 Leptospira borgpetersenii L550 Leptospira weilii Turneriella parva Brachyspira intermedia Granulicella tundricola Acidisarcina polymorpha Candidatus Solibacter usitatus Thermovirga lienii Acetomicrobium mobile Fretibacterium fastidiosum Cloacibacillus porcorum Fibrobacter succinogenes Fibrobacter succinogenes Gemmatirosa kalamazoonesis Bacteroides thetaiotaomicron VPI-5482 Bacteroides thetaiotaomicron 7330 Bacteroides fragilis YCH46 Bacteroides fragilis NCTC 9343 Bacteroides fragilis 638R Bacteroides dorei HS1\_L\_1\_B\_010 Parabacteroides sp. CT06 Butyricimonas sp. H184 Draconibacterium sp. M1 Chitinophaga pinensis Chitinophaga sp. H33E-04 Flavisolibacter tropicus Panacibacter ginsenosidivorans Pedobacter cryoconitis Pedobacter sp. KBS0701 Sphingobacterium mizutaii Sphingobacterium sp. CZ-2 Mucilaginibacter sp. PAMC 26640 Echinicola sp. LN3S3 Algoriphagus sanaruensis Spirosoma pollinicola Runella sp. HYN0085 Fibrella sp. ES10-3-2-2 Rhodocytophaga sp. 172606-1 Hymenobacter sedentarius Hymenobacter sp. 17J68-5 Pontibacter actiniarum Rufibacter sp. DG31D Rufibacter tibetensis Flammeovirgaceae bacterium 311 Maribacter sp. HTCC2170 Maribacter cobaltidurans Muricauda ruestringensis Euzebyella marina Antarcticibacterium sp. PAMC 28998 Prosthecochloris sp. CIB 2401 Nitrospira defluvii Nitrospira moscoviensis Candidatus Nitrospira inopinata Nitrospira japonica Methanosphaera sp. BMS Thermoplasmatales archaeon BRNA1 Archaeon GW2011\_AR20 | 184% 150% 122% 100% 82% 67% 55% |
