# Supplementary material for: Isolation, identification, and whole-genome sequencing of high-yield protease bacteria from Daqu of ZhangGong Laojiu
Source: PLoS One. 2022 Apr 26;17(4):e0264677. doi: 10.1371/journal.pone.0264677 (PMC9041807; doi:10.1371/journal.pone.0264677)
Supplement: S1 Raw data — (ZIP) [file pone.0264677.s002.zip › Raw data/GDD20120317-1_Bacillus_velezensis_Genome_result/4_Basic_Annot/KEGG/Bac_map/map01051.html]

KEGG PATHWAY: Biosynthesis of ansamycins - Reference pathway


|  |  |
| --- | --- |
| **Biosynthesis of ansamycins - Reference pathway** |  |

[
Pathway menu
| Organism menu
| Pathway entry
| User data mapping
]

|  |  |
| --- | --- |
| Reference pathway Reference pathway (KO) Reference pathway (EC) Reference pathway (Reaction) -----< Sort below by alphabet >----- Serratia sp. ATCC 39006 Serratia sp. ATCC 39006 Sodalis praecaptivus Xenorhabdus bovienii CS03 Xenorhabdus nematophila ATCC 19061 Xenorhabdus nematophila AN6/1 Xenorhabdus doucetiae Xenorhabdus poinarii Photobacterium gaetbulicola Pseudomonas sp. CCOS 191 Pseudomonas sp. Os17 Colwellia sp. MT41 Thioploca ingrica Ectothiorhodospira haloalkaliphila Ectothiorhodospira sp. BSL-9 Massilia sp. NR 4-1 Geobacter bemidjiensis Geobacter sp. M21 Myxococcus stipitatus Chondromyces crocatus Sandaracinus amylolyticus Haliangium ochraceum Rhizobium jaguaris Neorhizobium sp. SOG26 Rhodopseudomonas palustris HaA2 Rhodopseudomonas palustris BisB18 Oligotropha carboxidovorans OM5 (Mississippi) Oligotropha carboxidovorans OM5 (Goettingen) Oligotropha carboxidovorans OM4 Methylorubrum zatmanii Blastochloris viridis Paracoccus sp. BM15 Paracoccus aminovorans Paracoccus kondratievae Tabrizicola piscis Stappia indica Pseudohalocynthiibacter aestuariivivens Sphingosinithalassobacter sp. zrk23 Stella vacuolata Rhodospirillum centenum Azospirillum sp. CFH 70021 Candidatus Puniceispirillum marinum Bacillus thuringiensis serovar kurstaki YBT-1520 Bacillus thuringiensis serovar kurstaki HD-1 Bacillus thuringiensis serovar thuringiensis IS5056 Bacillus bombysepticus Bacillus pumilus SAFR-032 Bacillus pumilus SH-B9 Bacillus sp. JS Bacillus safensis Lysinibacillus varians Brevibacillus formosus Brevibacillus agri Paenibacillus mucilaginosus KNP414 Paenibacillus mucilaginosus 3016 Paenibacillus mucilaginosus K02 Paenibacillus larvae Paenibacillus durus Paenibacillus graminis Paenibacillus sp. FSL H7-0357 Paenibacillus riograndensis Paenibacillus naphthalenovorans Paenibacillus xylanexedens Paenibacillus donghaensis Paenibacillus lentus Tumebacillus avium Tumebacillus algifaecis Enterococcus silesiacus Clostridium cellulovorans Clostridium sp. BNL1100 Ruminiclostridium cellulolyticum Coprococcus sp. ART55/1 Coprococcus catus Blautia sp. YL58 Nocardia brasiliensis Nocardia seriolae Nocardia terpenica Nocardia sp. CS682 Nocardia otitidiscaviarum Rhodococcus opacus B4 Rhodococcus opacus PD630 Rhodococcus sp. WMMA185 Streptomyces globisporus Streptomyces scabiei Streptomyces violaceusniger Streptomyces cattleya NRRL 8057 = DSM 46488 Streptomyces bingchenggensis Streptomyces hygroscopicus subsp. jinggangensis 5008 Streptomyces hygroscopicus subsp. jinggangensis TL01 Streptomyces davaonensis Streptomyces rapamycinicus Streptomyces albulus NK660 Streptomyces albulus ZPM Streptomyces cyaneogriseus Streptomyces ambofaciens Streptomyces sp. CFMR 7 Streptomyces reticuli Streptomyces leeuwenhoekii Streptomyces parvulus Streptomyces sp. SAT1 Streptomyces clavuligerus Streptomyces griseochromogenes Streptomyces lincolnensis Streptomyces pluripotens Streptomyces niveus Streptomyces autolyticus Streptomyces alfalfae Streptomyces violaceoruber Streptomyces fodineus Streptomyces gilvosporeus Streptomyces malaysiensis Streptomyces laurentii Streptomyces albireticuli Kitasatospora setae Kitasatospora albolonga Kitasatospora sp. MMS16-BH015 Beutenbergia cavernae Nocardiopsis alba Nocardiopsis gilva Streptosporangium roseum Nonomuraea sp. WYY166 Frankia casuarinae Frankia sp. EAN1pec Frankia inefficax Frankia alni Saccharopolyspora erythraea Saccharomonospora sp. 31sw Amycolatopsis mediterranei U32 Amycolatopsis mediterranei S699 Amycolatopsis mediterranei S699 Amycolatopsis mediterranei RB Amycolatopsis methanolica Amycolatopsis sp. BJA-103 Amycolatopsis albispora Amycolatopsis sp. YIM 10 Pseudonocardia sp. EC080625-04 Pseudonocardia sp EC080619-01 Actinosynnema pretiosum Actinosynnema mirum Saccharothrix espanaensis Saccharothrix syringae Kutzneria albida Kibdelosporangium phytohabitans Lentzea guizhouensis Actinoalloteichus hymeniacidonis Actinoalloteichus sp. GBA129-24 Actinoalloteichus hoggarensis Actinoalloteichus sp. AHMU CJ021 Salinispora arenicola Micromonospora aurantiaca Micromonospora sp. L5 Micromonospora sp. B006 Micromonospora tulbaghiae Micromonospora sp. HM134 Actinoplanes sp. SE50/110 Actinoplanes missouriensis Actinoplanes sp. N902-109 Actinoplanes sp. SE50 Plantactinospora sp. KBS50 Plantactinospora sp. BC1 Plantactinospora sp. BB1 Catenulispora acidiphila Acidimicrobium ferrooxidans Ilumatobacter coccineus Halomicronema hongdechloris Microcoleus sp. PCC 7113 Nostoc sp. PCC 7107 Nostoc flagelliforme Cylindrospermum stagnale Nostocales cyanobacterium HT-58-2 Roseiflexus sp. RS-1 Roseiflexus castenholzii Herpetosiphon aurantiacus Sphaerobacter thermophilus Brevefilum fermentans Caldilinea aerophila Chthonomonas calidirosea Roseimaritima ulvae Thermogutta terrifontis Planctopirus limnophila Fuerstia marisgermanicae Gimesia sp. E7 Bremerella volcania Gemmata obscuriglobus Paludisphaera borealis Aquisphaera giovannonii Spirochaeta perfilievii Luteitalea pratensis Petrimonas sp. IBARAKI Proteiniphilum saccharofermentans Chitinophaga pinensis Chitinophaga sp. XS-30 Niabella soli Anseongella ginsenosidimutans Cyclobacterium amurskyense Dyadobacter fermentans Spirosoma montaniterrae Chryseolinea sp. KIS68-18 Elizabethkingia anophelis FMS-007 Euzebyella marina | 184% 150% 122% 100% 82% 67% 55% |
