# Supplementary material for: Isolation, identification, and whole-genome sequencing of high-yield protease bacteria from Daqu of ZhangGong Laojiu
Source: PLoS One. 2022 Apr 26;17(4):e0264677. doi: 10.1371/journal.pone.0264677 (PMC9041807; doi:10.1371/journal.pone.0264677)
Supplement: S1 Raw data — (ZIP) [file pone.0264677.s002.zip › Raw data/GDD20120317-1_Bacillus_velezensis_Genome_result/4_Basic_Annot/KEGG/Bac_map/map01055.html]

KEGG PATHWAY: Biosynthesis of vancomycin group antibiotics - Reference pathway


|  |  |
| --- | --- |
| **Biosynthesis of vancomycin group antibiotics - Reference pathway** |  |

[
Pathway menu
| Organism menu
| Pathway entry
| User data mapping
]

|  |  |
| --- | --- |
| Reference pathway Reference pathway (KO) Reference pathway (EC) Reference pathway (Reaction) -----< Sort below by alphabet >----- Serratia sp. FGI94 Serratia sp. MYb239 Dickeya paradisiaca Erwinia billingiae Pantoea sp. At-9b Pantoea rwandensis Pantoea sp. SO10 Photorhabdus laumondii subsp. laumondii TTO1 Photorhabdus laumondii subsp. laumondii DSPV002N Xanthomonas albilineans Xanthomonas translucens Pseudoxanthomonas suwonensis J1 Lysobacter gummosus Lysobacter enzymogenes C3 Lysobacter enzymogenes M497-1 Dyella thiooxydans Ahniella affigens Vibrio cholerae O49 1154-74 Pseudomonas mendocina ymp Pseudomonas resinovorans Pseudomonas pseudoalcaligenes Pseudomonas putida F1 Pseudomonas fluorescens F113 Pseudomonas brassicacearum subsp. brassicacearum NFM421 Pseudomonas brassicacearum DF41 Pseudomonas sp. TCU-HL1 Pseudomonas yamanorum Azotobacter vinelandii DJ Azotobacter vinelandii CA Azotobacter vinelandii CA6 Azotobacter chroococcum Psychrobacter sp. YP14 Acinetobacter sp. TTH0-4 Acinetobacter johnsonii Acinetobacter schindleri Acinetobacter wuhouensis Acinetobacter guillouiae Pseudoalteromonas luteoviolacea Pseudoalteromonas tunicata Paraglaciecola mesophila Saccharophagus degradans Zhongshania aliphaticivorans Legionella fallonii Legionella hackeliae Francisella tularensis subsp. tularensis SCHU S4 NR-28534 Granulosicoccus antarcticus Hahella chejuensis Hahella sp. KA22 Halomonas hydrothermalis Marinobacterium aestuarii Neptunomonas concharum Gynuella sunshinyii Oleiphilus messinensis Sinimarinibacterium sp. NLF-5-8 Vitreoscilla filiformis Vitreoscilla sp. C1 Aquaspirillum sp. LM1 Cupriavidus gilardii Cupriavidus pauculus Burkholderia oklahomensis E0147 Burkholderia oklahomensis C6786 Burkholderia sp. BDU6 Burkholderia glumae BGR1 Burkholderia glumae LMG 2196 = ATCC 33617 Burkholderia gladioli BSR3 Burkholderia gladioli ATCC 10248 Burkholderia plantarii PG1 Burkholderia plantarii ATCC 43733 Pandoraea sp. XY-2 Pusillimonas sp. ye3 Alcaligenes faecalis ZD02 Alcaligenes faecalis JQ135 Oligella urethralis Pigmentiphaga sp. H8 Pigmentiphaga aceris Rhodoferax koreense Polaromonas naphthalenivorans Acidovorax sp. KKS102 Acidovorax sp. RAC01 Acidovorax carolinensis NA2 Acidovorax carolinensis P4 Acidovorax carolinensis NA3 Acidovorax carolinensis P3 Acidovorax sp. 1608163 Verminephrobacter eiseniae Delftia tsuruhatensis Delftia sp. HK171 Comamonas kerstersii Comamonas serinivorans Comamonas sp. NLF 7-7 Ottowia sp. oral taxon 894 Ottowia oryzae Limnohabitans sp. 63ED37-2 Hydrogenophaga sp. RAC07 Hydrogenophaga sp. PBC Hydrogenophaga sp. LPB0072 Hydrogenophaga sp. PAMC20947 Hydrogenophaga pseudoflava Hydrogenophaga sp. BPS33 Diaphorobacter polyhydroxybutyrativorans Simplicispira suum Pulveribacter suum Melaminivora sp. SC2-9 Herbaspirillum huttiense Massilia sp. NR 4-1 Massilia violaceinigra Massilia albidiflava Massilia umbonata Rubrivivax gelatinosus Paucibacter sp. KCTC 42545 Aquabacterium olei Sphaerotilus natans Burkholderiales bacterium YL45 Nitrosomonas stercoris Methyloversatilis sp. RAC08 Methylophilus sp. TWE2 Rugosibacter aromaticivorans Oryzomicrobium terrae Azoarcus olearius Azoarcus communis Thauera humireducens Thauera sp. K11 Zoogloeaceae bacteirum Par-f-2 Geobacter uraniireducens Geobacter bemidjiensis Geobacter daltonii FRC-32 Geobacter sp. M21 Desulfovibrio vulgaris DP4 Desulfovibrio vulgaris RCH1 Desulfovibrio alaskensis Desulfovibrio magneticus Desulfovibrio salexigens Desulfovibrio hydrothermalis Desulfovibrio gigas Desulfovibrio fairfieldensis Desulfovibrio piger Desulfovibrio ferrophilus Pseudodesulfovibrio aespoeensis Pseudodesulfovibrio indicus Pseudodesulfovibrio sp. SRB007 Desulfomicrobium baculatum Desulfotalea psychrophila Desulfobulbus propionicus Desulfobulbus oralis Desulfobacterium autotrophicum Myxococcus stipitatus Stigmatella aurantiaca Archangium gephyra Melittangium boletus Cystobacter fuscus Sorangium cellulosum So ce56 Sorangium cellulosum So0157-2 Sandaracinus amylolyticus Labilithrix luteola Minicystis rosea Haliangium ochraceum Desulfarculus baarsii Bacteriovorax stolpii Hoeflea sp. IMCC20628 Parvibaculum lavamentivorans Bradyrhizobium sp. BF49 Bradyrhizobium guangzhouense Rhodopseudomonas palustris BisA53 Nitrobacter winogradskyi Bosea sp. PAMC 26642 Bosea sp. Tri-49 Azorhizobium caulinodans Ancylobacter sp. TS-1 Ancylobacter pratisalsi Microvirga sp. 17 mud 1-3 Rhodoplanes sp. Z2-YC6860 Youhaiella tibetensis Methylocystis parvus Nordella sp. HKS 07 Caulobacter mirabilis Rhodobacter blasticus Paracoccus denitrificans Ketogulonicigenium robustum Sulfitobacter sp. D7 Pelagibaca abyssi Thalassococcus sp. S3 Maricaulis maris Hyphomonas neptunium Novosphingobium aromaticivorans Novosphingobium resinovorum Novosphingobium sp. P6W Sphingomonas wittichii Sphingomonas panacis Sphingomonas sp. YZ-8 Roseomonas gilardii Roseomonas sp. FDAARGOS\_362 Roseomonas mucosa Stella humosa Stella vacuolata Azospirillum lipoferum Azospirillum brasilense Sp245 Azospirillum brasilense Az39 Azospirillum brasilense Sp7 Azospirillum thiophilum Azospirillum sp. TSH58 Azospirillum sp. M2T2B2 Hypericibacter terrae Hydrogenophilus thermoluteolus Lysinibacillus sp. SGAir0095 Lactobacillus amylovorus GRL 1112 Lactobacillus amylovorus GRL1118 Leuconostoc lactis Clostridium perfringens 13 Clostridium perfringens ATCC 13124 Clostridium ljungdahlii Clostridium sp. SY8519 Clostridium autoethanogenum Clostridium aceticum Clostridium carboxidivorans Geosporobacter ferrireducens Crassaminicella sp. SY095 Hungateiclostridium clariflavum Ruminiclostridium cellulolyticum Ruminococcus albus Faecalibacterium prausnitzii L2-6 Anaerobutyricum hallii Lachnospiraceae bacterium oral taxon 500 Clostridioides difficile CD196 Clostridioides difficile R20291 Acetoanaerobium sticklandii Desulfotomaculum reducens Desulfotomaculum nigrificans Desulfotomaculum ruminis Desulfofundulus kuznetsovii Desulfosporosinus orientis Desulfosporosinus acidiphilus Desulfosporosinus meridiei Dehalobacter sp. DCA Dehalobacter sp. CF Acetobacterium woodii Carboxydothermus hydrogenoformans Ammonifex degensii Caldicellulosiruptor obsidiansis Caldicellulosiruptor changbaiensis Thermodesulfobium narugense Thermodesulfobium acidiphilum Megasphaera elsdenii Pelosinus sp. UFO1 Sporomusa termitida Acidaminococcus intestini Absiella argi Erysipelotrichaceae bacterium I46 Mycobacterium avium subsp. paratuberculosis K-10 Mycobacterium avium subsp. paratuberculosis MAP4 Mycobacterium avium subsp. paratuberculosis E1 Mycobacterium avium 104 Mycobacterium intracellulare MOTT-02 Mycobacterium intracellulare ATCC 13950 Mycobacterium intracellulare subsp. intracellulare Mycobacterium intracellulare subsp. yongonense Mycobacterium paraintracellulare Mycobacterium chimaera Mycobacterium sp. JS623 Mycobacterium ulcerans Mycobacterium marinum M Mycobacterium marinum E11 Mycobacterium sp. MOTT36Y Mycobacterium liflandii Mycobacterium sp. NRRL B-3805 Mycobacterium shigaense Mycolicibacterium neoaurum Mycolicibacterium rhodesiae Nocardia brasiliensis Nocardia mangyaensis Nocardia seriolae Nocardia terpenica Nocardia sp. CS682 Rhodococcus jostii Rhodococcus opacus PD630 Rhodococcus sp. B7740 Rhodococcus sp. PBTS 1 Gordonia polyisoprenivorans Gordonia sp. KTR9 Gordonia sp. MMS17-SY073 Tsukamurella paurometabola Tsukamurella tyrosinosolvens Dietzia psychralcaliphila Streptomyces coelicolor Streptomyces albidoflavus Streptomyces avermitilis Streptomyces griseus Streptomyces globisporus Streptomyces scabiei Streptomyces violaceusniger Streptomyces cattleya NRRL 8057 = DSM 46488 Streptomyces cattleya NRRL 8057 = DSM 46488 Streptomyces bingchenggensis Streptomyces hygroscopicus subsp. jinggangensis 5008 Streptomyces hygroscopicus subsp. jinggangensis TL01 Streptomyces venezuelae Streptomyces davaonensis Streptomyces albus DSM 41398 Streptomyces fulvissimus Streptomyces collinus Streptomyces rapamycinicus Streptomyces albulus NK660 Streptomyces albulus ZPM Streptomyces lividans Streptomyces vietnamensis Streptomyces sp. 769 Streptomyces cyaneogriseus Streptomyces lydicus A02 Streptomyces lydicus 103 Streptomyces xiamenensis Streptomyces sp. CNQ-509 Streptomyces ambofaciens Streptomyces pristinaespiralis Streptomyces sp. CdTB01 Streptomyces reticuli Streptomyces sp. 4F Streptomyces leeuwenhoekii Streptomyces rubrolavendulae Streptomyces parvulus Streptomyces sp. SAT1 Streptomyces clavuligerus Streptomyces griseochromogenes Streptomyces qaidamensis Streptomyces lincolnensis Streptomyces noursei Streptomyces pluripotens Streptomyces niveus Streptomyces autolyticus Streptomyces alfalfae Streptomyces violaceoruber Streptomyces fodineus Streptomyces gilvosporeus Streptomyces malaysiensis Streptomyces laurentii Streptomyces alboflavus Streptomyces albireticuli Streptomyces lavendulae Streptomyces sp. MOE7 Streptomyces formicae Streptomyces griseorubiginosus Streptomyces rochei Streptomyces lunaelactis Streptomyces koyangensis Kitasatospora setae Kitasatospora albolonga Kitasatospora aureofaciens Kitasatospora sp. MMS16-BH015 Streptacidiphilus sp. DSM 106435 Rathayibacter festucae Frondihabitans sp. PAMC 28766 Cryobacterium sp. LW097 Luteipulveratus mongoliensis Xylanimicrobium sp. FW10M-9 Cellulomonas flavigena Serinicoccus sp. JLT9 Microlunatus sp. Gsoil 973 Tessaracoccus flavus Nocardioides sp. JS614 Nocardioides sp. MMS17-SY117 Nocardioides sp. S-1144 Kribbella flavida Aeromicrobium erythreum Aeromicrobium sp. 592 Aeromicrobium sp. MF47 Nocardiopsis dassonvillei Nocardiopsis alba Nocardiopsis gilva Streptomonospora sp. M2 Thermomonospora curvata Actinomadura sp. WMMB499 Streptosporangium roseum Nonomuraea sp. ATCC 55076 Nonomuraea sp. WYY166 Frankia casuarinae Frankia sp. EAN1pec Frankia inefficax Frankia alni Nakamurella multipartita Blastococcus saxobsidens Modestobacter marinus Saccharopolyspora erythraea Saccharomonospora viridis Saccharomonospora sp. 31sw Amycolatopsis mediterranei U32 Amycolatopsis mediterranei S699 Amycolatopsis mediterranei S699 Amycolatopsis mediterranei RB Amycolatopsis orientalis Amycolatopsis japonica Amycolatopsis methanolica Amycolatopsis sp. AA4 Amycolatopsis sp. BJA-103 Amycolatopsis albispora Amycolatopsis sp. YIM 10 Pseudonocardia sp. HH130629-09 Pseudonocardia sp. EC080610-09 Pseudonocardia sp EC080619-01 Pseudonocardia sp. HH130630-07 Actinosynnema pretiosum Actinosynnema mirum Saccharothrix espanaensis Saccharothrix syringae Kutzneria albida Kibdelosporangium phytohabitans Lentzea guizhouensis Actinoalloteichus hymeniacidonis Actinoalloteichus sp. GBA129-24 Actinoalloteichus fjordicus Actinoalloteichus hoggarensis Actinoalloteichus sp. AHMU CJ021 Alloactinosynnema sp. L-07 Prauserella marina Salinispora tropica Salinispora arenicola Micromonospora aurantiaca Micromonospora sp. L5 Micromonospora sp. B006 Micromonospora tulbaghiae Micromonospora sp. HM134 Micromonospora terminaliae Verrucosispora maris Actinoplanes sp. SE50/110 Actinoplanes missouriensis Actinoplanes sp. N902-109 Actinoplanes friuliensis Actinoplanes sp. SE50 Plantactinospora sp. KBS50 Catenulispora acidiphila Bifidobacterium thermophilum Actinopolyspora erythraea Candidatus Planktophila vernalis Actinobacteria bacterium Rubrobacter xylanophilus Egibacter rhizosphaerae Cyanobium sp. NIES-981 Pseudanabaena sp. ABRG5-3 Crocosphaera subtropica Gloeothece verrucosa Trichodesmium erythraeum Microcoleus sp. PCC 7113 Moorea producens Crinalium epipsammum Nostoc sp. PCC 7120 Nostoc punctiforme Nostoc sp. PCC 7107 Nostoc sp. PCC 7524 Nostoc sp. NIES-3756 Nostoc flagelliforme Nostoc sp. CENA543 Nostoc sphaeroides Trichormus variabilis Anabaena sp. 90 Anabaena sp. YBS01 Cylindrospermum stagnale Calothrix sp. PCC 7507 Calothrix sp. 336/3 Dolichospermum sp. UHCC 0315A Nostocales cyanobacterium HT-58-2 Chroococcidiopsis thermalis Pleurocapsa sp. PCC 7327 Stanieria sp. NIES-3757 Herpetosiphon aurantiacus Candidatus Promineofilum breve Deinococcus deserti Deinococcus wulumuqiensis Deinococcus sp. AJ005 Truepera radiovictrix Nibricoccus aquaticus HZ-65 Opitutaceae bacterium TAV5 Roseimicrobium sp. ORNL1 Verrucomicrobia bacterium IMCC26134 Planctomyces sp. SH-PL14 Tuwongella immobilis Treponema azotonutricium Treponema primitia Sediminispirochaeta smaragdinae Brachyspira pilosicoli P43/6/78 Acidisarcina polymorpha Edaphobacter sp. 12200R-103 Candidatus Solibacter usitatus Luteitalea pratensis Fusobacterium varium Fusobacterium gonidiaformans Fusobacterium necrophorum subsp. funduliforme Ilyobacter polytropus Acetomicrobium mobile Gemmatimonas aurantiaca Gemmatimonas phototrophica Gemmatirosa kalamazoonesis Bacteroides fragilis YCH46 Bacteroides fragilis BOB25 Bacteroides cellulosilyticus Prevotella melaninogenica Prevotella fusca Prevotella jejuni Alkalitalea saponilacus Panacibacter ginsenosidivorans Spirosoma sp. KCTC 42546 Runella sp. HYN0085 Rufibacter sp. DG31D Gramella forsetii Flavobacteriaceae bacterium UJ101 Prosthecochloris sp. CIB 2401 Thermodesulfovibrio yellowstonii Thermodesulfobacterium commune Thermodesulfobacterium sp. TA1 Candidatus Methylomirabilis oxyfera Methanobacterium lacus Methanobacterium sp. MB1 Candidatus Nitrosopelagicus brevis | 184% 150% 122% 100% 82% 67% 55% |
