# Supplementary material for: Isolation, identification, and whole-genome sequencing of high-yield protease bacteria from Daqu of ZhangGong Laojiu
Source: PLoS One. 2022 Apr 26;17(4):e0264677. doi: 10.1371/journal.pone.0264677 (PMC9041807; doi:10.1371/journal.pone.0264677)
Supplement: S1 Raw data — (ZIP) [file pone.0264677.s002.zip › Raw data/GDD20120317-1_Bacillus_velezensis_Genome_result/4_Basic_Annot/KEGG/Bac_map/map01100.html]

KEGG PATHWAY: Metabolic pathways - Reference pathway


|  |  |
| --- | --- |
| **Metabolic pathways - Reference pathway** |  |

[
Pathway menu
| Organism menu
| Pathway entry
|

Hide module list

| User data mapping
|

Image (png) file

]

|  |  |
| --- | --- |
| Reference pathway Reference pathway (KO) Reference pathway (EC) Reference pathway (Reaction) -----< Sort below by alphabet >----- Homo sapiens (human) Homo sapiens (human) + Disease/drug Mus musculus (mouse) Rattus norvegicus (rat) Bos taurus (cow) Sus scrofa (pig) Gallus gallus (chicken) Xenopus laevis (African clawed frog) Danio rerio (zebrafish) Oryzias latipes (Japanese medaka) Drosophila melanogaster (fruit fly) Caenorhabditis elegans (nematode) Arabidopsis thaliana (thale cress) Glycine max (soybean) Solanum lycopersicum (tomato) Oryza sativa japonica (Japanese rice) (RefSeq) Zea mays (maize) Chlamydomonas reinhardtii Saccharomyces cerevisiae (budding yeast) Candida albicans Neurospora crassa Aspergillus nidulans Aspergillus fumigatus Aspergillus flavus Schizosaccharomyces pombe (fission yeast) Cryptococcus neoformans var. neoformans JEC21 Dictyostelium discoideum (cellular slime mold) Entamoeba histolytica Plasmodium falciparum 3D7 Phaeodactylum tricornutum Thalassiosira pseudonana Trypanosoma brucei brucei Trypanosoma cruzi Leishmania major Escherichia coli K-12 MG1655 Escherichia coli K-12 W3110 Escherichia coli O157:H7 EDL933 (EHEC) Escherichia coli O157:H7 Sakai (EHEC) Salmonella enterica subsp. enterica serovar Typhi CT18 Salmonella enterica subsp. enterica serovar Typhimurium LT2 Salmonella enterica subsp. enterica serovar Typhimurium 14028S Shigella flexneri 301 (serotype 2a) Enterobacter cloacae subsp. cloacae ATCC 13047 Klebsiella pneumoniae subsp. pneumoniae MGH 78578 (serotype K52) Citrobacter rodentium Yersinia pestis CO92 (biovar Orientalis) Yersinia pestis KIM10+ (biovar Mediaevalis) Yersinia pseudotuberculosis IP 32953 (serotype I) Yersinia enterocolitica subsp. enterocolitica 8081 (serotype 0:8) Serratia marcescens SM39 Pectobacterium atrosepticum SCRI1043 Dickeya dadantii 3937 Erwinia amylovora CFBP1430 Buchnera aphidicola APS (Acyrthosiphon pisum) Haemophilus influenzae Rd KW20 (serotype d) Haemophilus ducreyi Pasteurella multocida subsp. multocida Pm70 Actinobacillus pleuropneumoniae L20 (serotype 5b) Xylella fastidiosa 9a5c Xanthomonas campestris pv. campestris ATCC 33913 Xanthomonas citri pv. citri 306 Xanthomonas oryzae pv. oryzae MAFF 311018 Vibrio cholerae O1 El Tor N16961 Vibrio vulnificus CMCP6 Vibrio parahaemolyticus RIMD 2210633 Aliivibrio fischeri ES114 Pseudomonas aeruginosa PAO1 Pseudomonas putida KT2440 Pseudomonas syringae pv. tomato DC3000 Pseudomonas syringae pv. syringae B728a Azotobacter vinelandii DJ Acinetobacter baumannii ATCC 17978 Acinetobacter baylyi Shewanella oneidensis Legionella pneumophila subsp. pneumophila Philadelphia 1 (serogroup 1) Methylococcus capsulatus Francisella tularensis subsp. tularensis SCHU S4 Neisseria meningitidis MC58 (serogroup B) Neisseria gonorrhoeae FA 1090 Ralstonia solanacearum GMI1000 Cupriavidus necator H16 Burkholderia mallei ATCC 23344 Burkholderia pseudomallei K96243 Paraburkholderia xenovorans LB400 Bordetella pertussis Tohama I Aromatoleum aromaticum Helicobacter pylori 26695 Helicobacter pylori J99 Campylobacter jejuni subsp. jejuni NCTC 11168 = ATCC 700819 Geobacter sulfurreducens PCA Desulfovibrio vulgaris Hildenborough Rickettsia prowazekii Madrid E Rickettsia conorii Wolbachia wMel (Drosophila melanogaster) Mesorhizobium japonicum MAFF 303099 Sinorhizobium meliloti 1021 Agrobacterium fabrum Rhizobium etli CFN 42 Rhizobium leguminosarum bv. viciae 3841 Brucella melitensis bv. 1 16M Brucella abortus 2308 Brucella suis 1330 Bradyrhizobium diazoefficiens USDA 110 Rhodopseudomonas palustris CGA009 Caulobacter vibrioides CB15 Rhodobacter sphaeroides 2.4.1 Paracoccus denitrificans Rhodospirillum rubrum ATCC 11170 Bacillus subtilis subsp. subtilis 168 Bacillus anthracis Ames Bacillus cereus ATCC 14579 Bacillus thuringiensis serovar konkukian 97-27 Staphylococcus aureus subsp. aureus N315 (MRSA/VSSA) Staphylococcus aureus subsp. aureus NCTC8325 Staphylococcus epidermidis RP62A (MRSE) Listeria monocytogenes EGD-e (serotype 1/2a) Listeria innocua (serotype 6a) Lactococcus lactis subsp. lactis Il1403 Lactococcus lactis subsp. cremoris MG1363 Streptococcus pyogenes M1 GAS (serotype M1) Streptococcus pneumoniae TIGR4 (virulent serotype 4) Streptococcus pneumoniae R6 (avirulent serotype 2) Streptococcus mutans UA159 (serotype c) Lactobacillus plantarum WCFS1 Lactobacillus johnsonii NCC 533 Lactobacillus casei BL23 Enterococcus faecalis V583 Clostridium acetobutylicum ATCC 824 Clostridium perfringens 13 Clostridium botulinum A ATCC 3502 Clostridioides difficile 630 Moorella thermoacetica ATCC 39073 Mycoplasma genitalium G37 Mycoplasma pneumoniae M129 Mycobacterium tuberculosis H37Rv Mycobacterium tuberculosis CDC1551 Mycobacterium leprae TN Mycolicibacterium smegmatis MC2 155 Mycobacteroides abscessus ATCC 19977 Corynebacterium glutamicum ATCC 13032 (Kyowa Hakko) Corynebacterium diphtheriae NCTC 13129 Rhodococcus jostii Streptomyces coelicolor Streptomyces avermitilis Streptomyces griseus Streptomyces cattleya NRRL 8057 = DSM 46488 Streptomyces glaucescens Kitasatospora setae Amycolatopsis mediterranei U32 Bifidobacterium longum NCC2705 Synechocystis sp. PCC 6803 Synechococcus elongatus PCC6301 Synechococcus elongatus PCC7942 Thermosynechococcus elongatus Prochlorococcus marinus subsp. marinus CCMP1375 Gloeobacter violaceus Nostoc sp. PCC 7120 Nostoc punctiforme Chloroflexus aurantiacus Deinococcus radiodurans Thermus thermophilus HB27 Chlamydia trachomatis D/UW-3/CX Chlamydia trachomatis 434/Bu Chlamydia pneumoniae CWL029 Borreliella burgdorferi B31 Treponema pallidum subsp. pallidum Nichols Treponema denticola Leptospira interrogans serovar Lai 56601 Fusobacterium nucleatum subsp. nucleatum ATCC 25586 Bacteroides thetaiotaomicron VPI-5482 Bacteroides fragilis YCH46 Flavobacterium psychrophilum JIP02/86 Chlorobaculum tepidum Aquifex aeolicus Hydrogenobacter thermophilus Thermotoga maritima MSB8 Methanocaldococcus jannaschii Methanococcus maripaludis S2 Methanothermobacter thermautotrophicus Archaeoglobus fulgidus DSM 4304 Thermoplasma acidophilum Pyrococcus furiosus DSM 3638 Thermococcus kodakarensis Methanosarcina barkeri Fusaro Halobacterium salinarum NRC-1 Haloarcula marismortui Haloferax volcanii Aeropyrum pernix Saccharolobus solfataricus P2 Sulfolobus acidocaldarius DSM 639 Pyrobaculum aerophilum Nitrosopumilus maritimus | 100% 70% 35% |

- **Category title**

- **Pathway module**
  - Carbohydrate metabolism
    - Central carbohydrate metabolism
      - M00001 Glycolysis (Embden-Meyerhof pathway), glucose => pyruvate
        - M00002 Glycolysis, core module involving three-carbon compounds
        - M00003 Gluconeogenesis, oxaloacetate => fructose-6P
        - M00307 Pyruvate oxidation, pyruvate => acetyl-CoA
        - M00009 Citrate cycle (TCA cycle, Krebs cycle)
        - M00010 Citrate cycle, first carbon oxidation, oxaloacetate => 2-oxoglutarate
        - M00011 Citrate cycle, second carbon oxidation, 2-oxoglutarate => oxaloacetate
        - M00004 Pentose phosphate pathway (Pentose phosphate cycle)
        - M00006 Pentose phosphate pathway, oxidative phase, glucose 6P => ribulose 5P
        - M00007 Pentose phosphate pathway, non-oxidative phase, fructose 6P => ribose 5P
        - M00580 Pentose phosphate pathway, archaea, fructose 6P => ribose 5P
        - M00005 PRPP biosynthesis, ribose 5P => PRPP
        - M00008 Entner-Doudoroff pathway, glucose-6P => glyceraldehyde-3P + pyruvate
        - M00308 Semi-phosphorylative Entner-Doudoroff pathway, gluconate => glycerate-3P
        - M00633 Semi-phosphorylative Entner-Doudoroff pathway, gluconate/galactonate => glycerate-3P
        - M00309 Non-phosphorylative Entner-Doudoroff pathway, gluconate/galactonate => glycerate
    - Other carbohydrate metabolism
      - M00014 Glucuronate pathway (uronate pathway)
        - M00630 D-Galacturonate degradation (fungi), D-galacturonate => glycerol
        - M00631 D-Galacturonate degradation (bacteria), D-galacturonate => pyruvate + D-glyceraldehyde 3P
        - M00061 D-Glucuronate degradation, D-glucuronate => pyruvate + D-glyceraldehyde 3P
        - M00081 Pectin degradation
        - M00632 Galactose degradation, Leloir pathway, galactose => alpha-D-glucose-1P
        - M00552 D-galactonate degradation, De Ley-Doudoroff pathway, D-galactonate => glycerate-3P
        - M00129 Ascorbate biosynthesis, animals, glucose-1P => ascorbate
        - M00114 Ascorbate biosynthesis, plants, glucose-6P => ascorbate
        - M00550 Ascorbate degradation, ascorbate => D-xylulose-5P
        - M00854 Glycogen biosynthesis, glucose-1P => glycogen/starch
        - M00855 Glycogen degradation, glycogen => glucose-6P
        - M00565 Trehalose biosynthesis, D-glucose 1P => trehalose
        - M00549 Nucleotide sugar biosynthesis, glucose => UDP-glucose
        - M00554 Nucleotide sugar biosynthesis, galactose => UDP-galactose
        - M00761 Undecaprenylphosphate alpha-L-Ara4N biosynthesis, UDP-GlcA => undecaprenyl phosphate alpha-L-Ara4N
        - M00012 Glyoxylate cycle
        - M00373 Ethylmalonyl pathway
        - M00740 Methylaspartate cycle
        - M00532 Photorespiration
        - M00013 Malonate semialdehyde pathway, propanoyl-CoA => acetyl-CoA
        - M00741 Propanoyl-CoA metabolism, propanoyl-CoA => succinyl-CoA
        - M00130 Inositol phosphate metabolism, PI=> PIP2 => Ins(1,4,5)P3 => Ins(1,3,4,5)P4
        - M00131 Inositol phosphate metabolism, Ins(1,3,4,5)P4 => Ins(1,3,4)P3 => myo-inositol
        - M00132 Inositol phosphate metabolism, Ins(1,3,4)P3 => phytate
  - Energy metabolism
    - Carbon fixation
      - M00165 Reductive pentose phosphate cycle (Calvin cycle)
        - M00166 Reductive pentose phosphate cycle, ribulose-5P => glyceraldehyde-3P
        - M00167 Reductive pentose phosphate cycle, glyceraldehyde-3P => ribulose-5P
        - M00168 CAM (Crassulacean acid metabolism), dark
        - M00169 CAM (Crassulacean acid metabolism), light
        - M00172 C4-dicarboxylic acid cycle, NADP - malic enzyme type
        - M00173 Reductive citrate cycle (Arnon-Buchanan cycle)
        - M00376 3-Hydroxypropionate bi-cycle
        - M00375 Hydroxypropionate-hydroxybutylate cycle
        - M00374 Dicarboxylate-hydroxybutyrate cycle
        - M00377 Reductive acetyl-CoA pathway (Wood-Ljungdahl pathway)
        - M00579 Phosphate acetyltransferase-acetate kinase pathway, acetyl-CoA => acetate
        - M00620 Incomplete reductive citrate cycle, acetyl-CoA => oxoglutarate
    - Methane metabolism
      - M00567 Methanogenesis, CO2 => methane
        - M00357 Methanogenesis, acetate => methane
        - M00356 Methanogenesis, methanol => methane
        - M00563 Methanogenesis, methylamine/dimethylamine/trimethylamine => methane
        - M00358 Coenzyme M biosynthesis
        - M00608 2-Oxocarboxylic acid chain extension, 2-oxoglutarate => 2-oxoadipate => 2-oxopimelate => 2-oxosuberate
        - M00174 Methane oxidation, methanotroph, methane => formaldehyde
        - M00346 Formaldehyde assimilation, serine pathway
        - M00345 Formaldehyde assimilation, ribulose monophosphate pathway
        - M00344 Formaldehyde assimilation, xylulose monophosphate pathway
        - M00378 F420 biosynthesis
        - M00422 Acetyl-CoA pathway, CO2 => acetyl-CoA
    - Nitrogen metabolism
      - M00175 Nitrogen fixation, nitrogen => ammonia
        - M00531 Assimilatory nitrate reduction, nitrate => ammonia
        - M00530 Dissimilatory nitrate reduction, nitrate => ammonia
        - M00529 Denitrification, nitrate => nitrogen
        - M00528 Nitrification, ammonia => nitrite
        - M00804 Complete nitrification, comammox, ammonia => nitrite => nitrate
    - Sulfur metabolism
      - M00176 Assimilatory sulfate reduction, sulfate => H2S
        - M00596 Dissimilatory sulfate reduction, sulfate => H2S
        - M00595 Thiosulfate oxidation by SOX complex, thiosulfate => sulfate
  - Lipid metabolism
    - Fatty acid metabolism
      - M00082 Fatty acid biosynthesis, initiation
        - M00083 Fatty acid biosynthesis, elongation
        - M00873 Fatty acid biosynthesis in mitochondria, animals
        - M00874 Fatty acid biosynthesis in mitochondria, fungi
        - M00085 Fatty acid elongation in mitochondria
        - M00415 Fatty acid elongation in endoplasmic reticulum
        - M00086 beta-Oxidation, acyl-CoA synthesis
        - M00087 beta-Oxidation
        - M00861 beta-Oxidation, peroxisome, VLCFA
    - Sterol biosynthesis
      - M00101 Cholesterol biosynthesis, squalene 2,3-epoxide => cholesterol
        - M00102 Ergocalciferol biosynthesis
        - M00103 Cholecalciferol biosynthesis
        - M00104 Bile acid biosynthesis, cholesterol => cholate/chenodeoxycholate
        - M00106 Conjugated bile acid biosynthesis, cholate => taurocholate/glycocholate
        - M00862 beta-Oxidation, peroxisome, tri/dihydroxycholestanoyl-CoA => choloyl/chenodeoxycholoyl-CoA
        - M00107 Steroid hormone biosynthesis, cholesterol => prognenolone => progesterone
        - M00108 C21-Steroid hormone biosynthesis, progesterone => corticosterone/aldosterone
        - M00109 C21-Steroid hormone biosynthesis, progesterone => cortisol/cortisone
        - M00110 C19/C18-Steroid hormone biosynthesis, pregnenolone => androstenedione => estrone
    - Lipid metabolism
      - M00088 Ketone body biosynthesis, acetyl-CoA => acetoacetate/3-hydroxybutyrate/acetone
        - M00089 Triacylglycerol biosynthesis
        - M00098 Acylglycerol degradation
        - M00090 Phosphatidylcholine (PC) biosynthesis, choline => PC
        - M00091 Phosphatidylcholine (PC) biosynthesis, PE => PC
        - M00092 Phosphatidylethanolamine (PE) biosynthesis, ethanolamine => PE
        - M00093 Phosphatidylethanolamine (PE) biosynthesis, PA => PS => PE
        - M00094 Ceramide biosynthesis
        - M00066 Lactosylceramide biosynthesis
        - M00067 Sulfoglycolipids biosynthesis, ceramide/1-alkyl-2-acylglycerol => sulfatide/seminolipid
        - M00099 Sphingosine biosynthesis
        - M00100 Sphingosine degradation
        - M00113 Jasmonic acid biosynthesis
  - Nucleotide metabolism
    - Purine metabolism
      - M00048 Inosine monophosphate biosynthesis, PRPP + glutamine => IMP
        - M00049 Adenine ribonucleotide biosynthesis, IMP => ADP,ATP
        - M00050 Guanine ribonucleotide biosynthesis IMP => GDP,GTP
        - M00546 Purine degradation, xanthine => urea
    - Pyrimidine metabolism
      - M00051 Uridine monophosphate biosynthesis, glutamine (+ PRPP) => UMP
        - M00052 Pyrimidine ribonucleotide biosynthesis, UMP => UDP/UTP,CDP/CTP
        - M00053 Pyrimidine deoxyribonuleotide biosynthesis, CDP/CTP => dCDP/dCTP,dTDP/dTTP
        - M00046 Pyrimidine degradation, uracil => beta-alanine, thymine => 3-aminoisobutanoate
  - Amino acid metabolism
    - Serine and threonine metabolism
      - M00020 Serine biosynthesis, glycerate-3P => serine
        - M00018 Threonine biosynthesis, aspartate => homoserine => threonine
        - M00555 Betaine biosynthesis, choline => betaine
        - M00033 Ectoine biosynthesis, aspartate => ectoine
    - Cysteine and methionine metabolism
      - M00021 Cysteine biosynthesis, serine => cysteine
        - M00338 Cysteine biosynthesis, homocysteine + serine => cysteine
        - M00609 Cysteine biosynthesis, methionine => cysteine
        - M00017 Methionine biosynthesis, apartate => homoserine => methionine
        - M00034 Methionine salvage pathway
        - M00035 Methionine degradation
        - M00368 Ethylene biosynthesis, methionine => ethylene
    - Branched-chain amino acid metabolism
      - M00019 Valine/isoleucine biosynthesis, pyruvate => valine / 2-oxobutanoate => isoleucine
        - M00535 Isoleucine biosynthesis, pyruvate => 2-oxobutanoate
        - M00570 Isoleucine biosynthesis, threonine => 2-oxobutanoate => isoleucine
        - M00432 Leucine biosynthesis, 2-oxoisovalerate => 2-oxoisocaproate
        - M00036 Leucine degradation, leucine => acetoacetate + acetyl-CoA
    - Lysine metabolism
      - M00016 Lysine biosynthesis, succinyl-DAP pathway, aspartate => lysine
        - M00525 Lysine biosynthesis, acetyl-DAP pathway, aspartate => lysine
        - M00526 Lysine biosynthesis, DAP dehydrogenase pathway, aspartate => lysine
        - M00527 Lysine biosynthesis, DAP aminotransferase pathway, aspartate => lysine
        - M00030 Lysine biosynthesis, AAA pathway, 2-oxoglutarate => 2-aminoadipate => lysine
        - M00433 Lysine biosynthesis, 2-oxoglutarate => 2-oxoadipate
        - M00031 Lysine biosynthesis, mediated by LysW, 2-aminoadipate => lysine
        - M00032 Lysine degradation, lysine => saccharopine => acetoacetyl-CoA
    - Arginine and proline metabolism
      - M00028 Ornithine biosynthesis, glutamate => ornithine
        - M00763 Ornithine biosynthesis, mediated by LysW, glutamate => ornithine
        - M00844 Arginine biosynthesis, ornithine => arginine
        - M00845 Arginine biosynthesis, glutamate => acetylcitrulline => arginine
        - M00029 Urea cycle
        - M00015 Proline biosynthesis, glutamate => proline
        - M00047 Creatine pathway
        - M00879 Arginine succinyltransferase pathway, arginine => glutamate
    - Polyamine biosynthesis
      - M00133 Polyamine biosynthesis, arginine => agmatine => putrescine => spermidine
        - M00134 Polyamine biosynthesis, arginine => ornithine => putrescine
        - M00135 GABA biosynthesis, eukaryotes, putrescine => GABA
        - M00136 GABA biosynthesis, prokaryotes, putrescine => GABA
    - Histidine metabolism
      - M00026 Histidine biosynthesis, PRPP => histidine
        - M00045 Histidine degradation, histidine => N-formiminoglutamate => glutamate
    - Aromatic amino acid metabolism
      - M00022 Shikimate pathway, phosphoenolpyruvate + erythrose-4P => chorismate
        - M00023 Tryptophan biosynthesis, chorismate => tryptophan
        - M00024 Phenylalanine biosynthesis, chorismate => phenylalanine
        - M00025 Tyrosine biosynthesis, chorismate => tyrosine
        - M00040 Tyrosine biosynthesis, prephanate => pretyrosine => tyrosine
        - M00042 Catecholamine biosynthesis, tyrosine => dopamine => noradrenaline => adrenaline
        - M00043 Thyroid hormone biosynthesis, tyrosine => triiodothyronine/thyroxine
        - M00044 Tyrosine degradation, tyrosine => homogentisate
        - M00533 Homoprotocatechuate degradation, homoprotocatechuate => 2-oxohept-3-enedioate
        - M00545 Trans-cinnamate degradation, trans-cinnamate => acetyl-CoA
        - M00037 Melatonin biosynthesis, tryptophan => serotonin => melatonin
        - M00038 Tryptophan metabolism, tryptophan => kynurenine => 2-aminomuconate
    - Other amino acid metabolism
      - M00027 GABA (gamma-Aminobutyrate) shunt
        - M00369 Cyanogenic glycoside biosynthesis, tyrosine => dhurrin
        - M00118 Glutathione biosynthesis, glutamate => glutathione
  - Glycan metabolism
    - Glycan biosynthesis
      - M00055 N-glycan precursor biosynthesis
        - M00072 N-glycosylation by oligosaccharyltransferase
        - M00073 N-glycan precursor trimming
        - M00074 N-glycan biosynthesis, high-mannose type
        - M00075 N-glycan biosynthesis, complex type
        - M00056 O-glycan biosynthesis, mucin type core
        - M00872 O-glycan biosynthesis, mannose type (core M3)
        - M00065 GPI-anchor biosynthesis, core oligosaccharide
        - M00070 Glycosphingolipid biosynthesis, lacto-series, LacCer => Lc4Cer
        - M00071 Glycosphingolipid biosynthesis, neolacto-series, LacCer => nLc4Cer
        - M00068 Glycosphingolipid biosynthesis, globo-series, LacCer => Gb4Cer
        - M00069 Glycosphingolipid biosynthesis, ganglio series, LacCer => GT3
    - Glycosaminoglycan metabolism
      - M00057 Glycosaminoglycan biosynthesis, linkage tetrasaccharide
        - M00058 Glycosaminoglycan biosynthesis, chondroitin sulfate backbone
        - M00059 Glycosaminoglycan biosynthesis, heparan sulfate backbone
        - M00076 Dermatan sulfate degradation
        - M00077 Chondroitin sulfate degradation
        - M00078 Heparan sulfate degradation
        - M00079 Keratan sulfate degradation
    - Lipopolysaccharide metabolism
      - M00060 KDO2-lipid A biosynthesis, Raetz pathway, LpxL-LpxM type
        - M00866 KDO2-lipid A biosynthesis, Raetz pathway, non-LpxL-LpxM type
        - M00867 KDO2-lipid A modification pathway
        - M00063 CMP-KDO biosynthesis
        - M00064 ADP-L-glycero-D-manno-heptose biosynthesis
  - Metabolism of cofactors and vitamins
    - Cofactor and vitamin metabolism
      - M00127 Thiamine biosynthesis, AIR => thiamine-P/thiamine-2P
        - M00125 Riboflavin biosynthesis, GTP => riboflavin/FMN/FAD
        - M00124 Pyridoxal biosynthesis, erythrose-4P => pyridoxal-5P
        - M00115 NAD biosynthesis, aspartate => NAD
        - M00810 Nicotine degradation, pyridine pathway, nicotine => 2,6-dihydroxypyridine/succinate semialdehyde
        - M00811 Nicotine degradation, pyrrolidine pathway, nicotine => succinate semialdehyde
        - M00622 Nicotinate degradation, nicotinate => fumarate
        - M00119 Pantothenate biosynthesis, valine/L-aspartate => pantothenate
        - M00120 Coenzyme A biosynthesis, pantothenate => CoA
        - M00572 Pimeloyl-ACP biosynthesis, BioC-BioH pathway, malonyl-ACP => pimeloyl-ACP
        - M00123 Biotin biosynthesis, pimeloyl-ACP/CoA => biotin
        - M00573 Biotin biosynthesis, BioI pathway, long-chain-acyl-ACP => pimeloyl-ACP => biotin
        - M00577 Biotin biosynthesis, BioW pathway, pimelate => pimeloyl-CoA => biotin
        - M00126 Tetrahydrofolate biosynthesis, GTP => THF
        - M00840 Tetrahydrofolate biosynthesis, mediated by ribA and trpF, GTP => THF
        - M00841 Tetrahydrofolate biosynthesis, mediated by PTPS, GTP => THF
        - M00842 Tetrahydrobiopterin biosynthesis, GTP => BH4
        - M00843 L-threo-Tetrahydrobiopterin biosynthesis, GTP => L-threo-BH4
        - M00880 Molybdenum cofactor biosynthesis, GTP => molybdenum cofactor
        - M00140 C1-unit interconversion, prokaryotes
        - M00141 C1-unit interconversion, eukaryotes
        - M00868 Heme biosynthesis, animals and fungi, glycine => heme
        - M00121 Heme biosynthesis, plants and bacteria, glutamate => heme
        - M00846 Siroheme biosynthesis, glutamate => siroheme
        - M00847 Heme biosynthesis, archaea, siroheme => heme
        - M00122 Cobalamin biosynthesis, cobinamide => cobalamin
        - M00836 Coenzyme F430 biosynthesis, sirohydrochlorin => coenzyme F430
        - M00117 Ubiquinone biosynthesis, prokaryotes, chorismate => ubiquinone
        - M00128 Ubiquinone biosynthesis, eukaryotes, 4-hydroxybenzoate => ubiquinone
        - M00116 Menaquinone biosynthesis, chorismate => menaquinol
        - M00112 Tocopherol/tocotorienol biosynthesis
  - Biosynthesis of terpenoids and polyketides
    - Terpenoid backbone biosynthesis
      - M00095 C5 isoprenoid biosynthesis, mevalonate pathway
        - M00849 C5 isoprenoid biosynthesis, mevalonate pathway, archaea
        - M00096 C5 isoprenoid biosynthesis, non-mevalonate pathway
        - M00364 C10-C20 isoprenoid biosynthesis, bacteria
        - M00365 C10-C20 isoprenoid biosynthesis, archaea
        - M00366 C10-C20 isoprenoid biosynthesis, plants
        - M00367 C10-C20 isoprenoid biosynthesis, non-plant eukaryotes
    - Plant terpenoid biosynthesis
      - M00097 beta-Carotene biosynthesis, GGAP => beta-carotene
        - M00372 Abscisic acid biosynthesis, beta-carotene => abscisic acid
        - M00371 Castasterone biosynthesis, campesterol => castasterone
    - Macrolide biosynthesis
      - M00773 Tylosin biosynthesis, methylmalonyl-CoA + malonyl-CoA => tylactone => tylosin
        - M00774 Erythromycin biosynthesis, propanoyl-CoA + methylmalonyl-CoA => deoxyerythronolide B => erythromycin A/B
        - M00775 Oleandomycin biosynthesis, malonyl-CoA + methylmalonyl-CoA => 8,8a-deoxyoleandolide => oleandomycin
        - M00776 Pikromycin/methymycin biosynthesis, methylmalonyl-CoA + malonyl-CoA => narbonolide/10-deoxymethynolide => pikromycin/methymycin
        - M00777 Avermectin biosynthesis, 2-methylbutanoyl-CoA/isobutyryl-CoA => 6,8a-Seco-6,8a-deoxy-5-oxoavermectin 1a/1b aglycone => avermectin A1a/B1a/A1b/B1b
    - Enediyne biosynthesis
      - M00824 9-membered enediyne core biosynthesis, malonyl-CoA => 3-hydroxyhexadeca-4,6,8,10,12,14-hexaenoyl-ACP => 9-membered enediyne core
        - M00825 10-membered enediyne core biosynthesis, malonyl-CoA => 3-hydroxyhexadeca-4,6,8,10,12,14-hexaenoyl-ACP => 10-membered enediyne core
        - M00826 C-1027 benzoxazolinate moiety biosynthesis, chorismate => benzoxazolinyl-CoA
        - M00827 C-1027 beta-amino acid moiety biosynthesis, tyrosine => 3-chloro-4,5-dihydroxy-beta-phenylalanyl-PCP
        - M00828 Maduropeptin beta-hydroxy acid moiety biosynthesis, tyrosine => 3-(4-hydroxyphenyl)-3-oxopropanoyl-PCP
        - M00829 3,6-Dimethylsalicylyl-CoA biosynthesis, malonyl-CoA => 6-methylsalicylate => 3,6-dimethylsalicylyl-CoA
        - M00830 Neocarzinostatin naphthoate moiety biosynthesis, malonyl-CoA => 2-hydroxy-5-methyl-1-naphthoate => 2-hydroxy-7-methoxy-5-methyl-1-naphthoyl-CoA
        - M00831 Kedarcidin 2-hydroxynaphthoate moiety biosynthesis, malonyl-CoA => 3,6,8-trihydroxy-2-naphthoate => 3-hydroxy-7,8-dimethoxy-6-isopropoxy-2-naphthoyl-CoA
        - M00832 Kedarcidin 2-aza-3-chloro-beta-tyrosine moiety biosynthesis, azatyrosine => 2-aza-3-chloro-beta-tyrosyl-PCP
        - M00834 Calicheamicin orsellinate moiety biosynthesis, malonyl-CoA => orsellinate-ACP => 5-iodo-2,3-dimethoxyorsellinate-ACP
        - M00833 Calicheamicin biosynthesis, calicheamicinone => calicheamicin
    - Type II polyketide biosynthesis
      - M00778 Type II polyketide backbone biosynthesis, acyl-CoA + malonyl-CoA => polyketide
        - M00779 Dihydrokalafungin biosynthesis, octaketide => dihydrokalafungin
        - M00780 Tetracycline/oxytetracycline biosynthesis, pretetramide => tetracycline/oxytetracycline
        - M00823 Chlortetracycline biosynthesis, pretetramide => chlortetracycline
        - M00781 Nogalavinone/aklavinone biosynthesis, deoxynogalonate/deoxyaklanonate => nogalavinone/aklavinone
        - M00782 Mithramycin biosynthesis, 4-demethylpremithramycinone => mithramycin
        - M00783 Tetracenomycin C/8-demethyltetracenomycin C biosynthesis, tetracenomycin F2 => tetracenomycin C/8-demethyltetracenomycin C
        - M00784 Elloramycin biosynthesis, 8-demethyltetracenomycin C => elloramycin A
    - Polyketide sugar unit biosynthesis
      - M00793 dTDP-L-rhamnose biosynthesis
        - M00794 dTDP-6-deoxy-D-allose biosynthesis
        - M00795 dTDP-beta-L-noviose biosynthesis
        - M00796 dTDP-D-mycaminose biosynthesis
        - M00797 dTDP-D-desosamine biosynthesis
        - M00798 dTDP-L-mycarose biosynthesis
        - M00799 dTDP-L-oleandrose biosynthesis
        - M00800 dTDP-L-megosamine biosynthesis
        - M00801 dTDP-L-olivose biosynthesis
        - M00802 dTDP-D-forosamine biosynthesis
        - M00803 dTDP-D-angolosamine biosynthesis
  - Biosynthesis of other secondary metabolites
    - Biosynthesis of phytochemical compounds
      - M00039 Monolignol biosynthesis, phenylalanine/tyrosine => monolignol
        - M00137 Flavanone biosynthesis, phenylalanine => naringenin
        - M00138 Flavonoid biosynthesis, naringenin => pelargonidin
        - M00370 Glucosinolate biosynthesis, tryptophan => glucobrassicin
        - M00900 Cronin biosynthesis, zeaxanthin => crocin
        - M00894 Cannabidiol biosynthesis, malonyl-CoA => cannabidiol/dronabinol
        - M00902 Podophyllotoxin biosynthesis, coniferyl alcohol => podophyllotoxin
    - Biosynthesis of beta-lactams
      - M00672 Penicillin biosynthesis, aminoadipate + cycteine + valine => penicillin
        - M00673 Cephamycin C biosynthesis, aminoadipate + cycteine + valine => cephamycin C
        - M00675 Carbapenem-3-carboxylate biosynthesis, pyrroline-5-carboxylate + malonyl-CoA => carbapenem-3-carboxylate
        - M00736 Nocardicin A biosynthesis, L-pHPG + arginine + serine => nocardicin A
        - M00674 Clavaminate biosynthesis, arginine + glyceraldehyde-3P => clavaminate
    - Biosynthesis of other antibiotics
      - M00877 Kanosamine biosynthesis glucose 6-phosphate => kanosamine
        - M00889 Puromycin biosynthesis, ATP => puromycin
        - M00815 Validamycin A biosynthesis, sedoheptulopyranose-7P => validamycin A
        - M00904 Dapdiamides biosynthesis, L-2,3-diaminopropanoate => dapdiamide A/B/C
        - M00785 Cycloserine biosynthesis, arginine/serine => cycloserine
        - M00787 Bacilysin biosynthesis, prephenate => bacilysin
        - M00848 Aurachin biosynthesis, anthranilate => aurachin A
        - M00788 Terpentecin biosynthesis, GGAP => terpentecin
        - M00819 Pentalenolactone biosynthesis, farnesyl-PP => pentalenolactone
        - M00903 Fosfomycin biosynthesis, phosphoenolpyruvate => fosfomycin
        - M00890 Roseoflavin biosynthesis, FMN => roseoflavin
    - Biosynthesis of other fungal compounds
      - M00661 Paspaline biosynthesis, geranylgeranyl-PP + indoleglycerol phosphate => paspaline
        - M00786 Fumitremorgin alkaloid biosynthesis, tryptophan + proline => fumitremorgin C/A
        - M00893 Lovastatin biosynthesis, malonyl-CoA => lovastatin acid
        - M00891 Ditryptophenaline biosynthesis, tryptophan + phenylalanine => ditryptophenaline
        - M00901 Fumiquinazoline biosynthesis, tryptophan + alanine + anthranilate => fumiquinazoline
    - Biosynthesis of other bacterial compounds
      - M00814 Acarbose biosynthesis, sedoheptulopyranose-7P => acarbose
        - M00789 Rebeccamycin biosynthesis, tryptophan => rebeccamycin
        - M00790 Pyrrolnitrin biosynthesis, tryptophan => pyrrolnitrin
        - M00805 Staurosporine biosynthesis, tryptophan => staurosporine
        - M00808 Violacein biosynthesis, tryptophan => violacein
        - M00835 Pyocyanine biosynthesis, chorismate => pyocyanine
        - M00837 Prodigiosin biosynthesis, L-proline => prodigiosin
        - M00838 Undecylprodigiosin biosynthesis, L-proline => undecylprodigiosin
        - M00905 Grixazone biosynthesis, aspartate 4-semialdehyde => grixazone B
        - M00876 Staphyloferrin A biosynthesis, L-ornithine => staphyloferrin A
        - M00875 Staphyloferrin B biosynthesis, L-serine => staphyloferrin B
        - M00906 Ethynylserine biosynthesis, lysine => ethynylserine
  - Xenobiotics biodegradation
    - Aromatics degradation
      - M00538 Toluene degradation, toluene => benzoate
        - M00537 Xylene degradation, xylene => methylbenzoate
        - M00419 Cymene degradation, p-cymene => p-cumate
        - M00547 Benzene/toluene degradation, benzene => catechol / toluene => 3-methylcatechol
        - M00548 Benzene degradation, benzene => catechol
        - M00551 Benzoate degradation, benzoate => catechol / methylbenzoate => methylcatechol
        - M00637 Anthranilate degradation, anthranilate => catechol
        - M00568 Catechol ortho-cleavage, catechol => 3-oxoadipate
        - M00569 Catechol meta-cleavage, catechol => acetyl-CoA / 4-methylcatechol => propanoyl-CoA
        - M00539 Cumate degradation, p-cumate => 2-oxopent-4-enoate + 2-methylpropanoate
        - M00543 Biphenyl degradation, biphenyl => 2-oxopent-4-enoate + benzoate
        - M00544 Carbazole degradation, carbazole => 2-oxopent-4-enoate + anthranilate
        - M00418 Toluene degradation, anaerobic, toluene => benzoyl-CoA
        - M00541 Benzoyl-CoA degradation, benzoyl-CoA => 3-hydroxypimeloyl-CoA
        - M00540 Benzoate degradation, cyclohexanecarboxylic acid =>pimeloyl-CoA
        - M00534 Naphthalene degradation, naphthalene => salicylate
        - M00638 Salicylate degradation, salicylate => gentisate
        - M00624 Terephthalate degradation, terephthalate => 3,4-dihydroxybenzoate
        - M00623 Phthalate degradation, phthalate => protocatechuate
        - M00636 Phthalate degradation, phthalate => protocatechuate
        - M00878 Phenylacetate degradation, phenylaxetate => acetyl-CoA/succinyl-CoA
