# Supplementary material for: Isolation, identification, and whole-genome sequencing of high-yield protease bacteria from Daqu of ZhangGong Laojiu
Source: PLoS One. 2022 Apr 26;17(4):e0264677. doi: 10.1371/journal.pone.0264677 (PMC9041807; doi:10.1371/journal.pone.0264677)
Supplement: S1 Raw data — (ZIP) [file pone.0264677.s002.zip › Raw data/GDD20120317-1_Bacillus_velezensis_Genome_result/4_Basic_Annot/KEGG/Bac_map/map01110.html]

KEGG PATHWAY: Biosynthesis of secondary metabolites - Reference pathway


|  |  |
| --- | --- |
| **Biosynthesis of secondary metabolites - Reference pathway** |  |

[
Pathway menu
| Organism menu
| Pathway entry
|

Hide module list

| User data mapping
|

Image (png) file

]

|  |  |
| --- | --- |
| Reference pathway Reference pathway (KO) Reference pathway (EC) Reference pathway (Reaction) -----< Sort below by alphabet >----- Arabidopsis thaliana (thale cress) Glycine max (soybean) Solanum lycopersicum (tomato) Oryza sativa japonica (Japanese rice) (RefSeq) Zea mays (maize) Chlamydomonas reinhardtii Saccharomyces cerevisiae (budding yeast) Candida albicans Neurospora crassa Aspergillus nidulans Aspergillus fumigatus Aspergillus flavus Schizosaccharomyces pombe (fission yeast) Cryptococcus neoformans var. neoformans JEC21 Dictyostelium discoideum (cellular slime mold) Entamoeba histolytica Plasmodium falciparum 3D7 Phaeodactylum tricornutum Thalassiosira pseudonana Trypanosoma brucei brucei Trypanosoma cruzi Leishmania major Escherichia coli K-12 MG1655 Escherichia coli K-12 W3110 Escherichia coli O157:H7 EDL933 (EHEC) Escherichia coli O157:H7 Sakai (EHEC) Salmonella enterica subsp. enterica serovar Typhi CT18 Salmonella enterica subsp. enterica serovar Typhimurium LT2 Salmonella enterica subsp. enterica serovar Typhimurium 14028S Shigella flexneri 301 (serotype 2a) Enterobacter cloacae subsp. cloacae ATCC 13047 Klebsiella pneumoniae subsp. pneumoniae MGH 78578 (serotype K52) Citrobacter rodentium Yersinia pestis CO92 (biovar Orientalis) Yersinia pestis KIM10+ (biovar Mediaevalis) Yersinia pseudotuberculosis IP 32953 (serotype I) Yersinia enterocolitica subsp. enterocolitica 8081 (serotype 0:8) Serratia marcescens SM39 Pectobacterium atrosepticum SCRI1043 Dickeya dadantii 3937 Erwinia amylovora CFBP1430 Buchnera aphidicola APS (Acyrthosiphon pisum) Haemophilus influenzae Rd KW20 (serotype d) Haemophilus ducreyi Pasteurella multocida subsp. multocida Pm70 Actinobacillus pleuropneumoniae L20 (serotype 5b) Xylella fastidiosa 9a5c Xanthomonas campestris pv. campestris ATCC 33913 Xanthomonas citri pv. citri 306 Xanthomonas oryzae pv. oryzae MAFF 311018 Vibrio cholerae O1 El Tor N16961 Vibrio vulnificus CMCP6 Vibrio parahaemolyticus RIMD 2210633 Aliivibrio fischeri ES114 Pseudomonas aeruginosa PAO1 Pseudomonas putida KT2440 Pseudomonas syringae pv. tomato DC3000 Pseudomonas syringae pv. syringae B728a Azotobacter vinelandii DJ Acinetobacter baumannii ATCC 17978 Acinetobacter baylyi Shewanella oneidensis Legionella pneumophila subsp. pneumophila Philadelphia 1 (serogroup 1) Methylococcus capsulatus Francisella tularensis subsp. tularensis SCHU S4 Neisseria meningitidis MC58 (serogroup B) Neisseria gonorrhoeae FA 1090 Ralstonia solanacearum GMI1000 Cupriavidus necator H16 Burkholderia mallei ATCC 23344 Burkholderia pseudomallei K96243 Paraburkholderia xenovorans LB400 Bordetella pertussis Tohama I Aromatoleum aromaticum Helicobacter pylori 26695 Helicobacter pylori J99 Campylobacter jejuni subsp. jejuni NCTC 11168 = ATCC 700819 Geobacter sulfurreducens PCA Desulfovibrio vulgaris Hildenborough Rickettsia prowazekii Madrid E Rickettsia conorii Wolbachia wMel (Drosophila melanogaster) Mesorhizobium japonicum MAFF 303099 Sinorhizobium meliloti 1021 Agrobacterium fabrum Rhizobium etli CFN 42 Rhizobium leguminosarum bv. viciae 3841 Brucella melitensis bv. 1 16M Brucella abortus 2308 Brucella suis 1330 Bradyrhizobium diazoefficiens USDA 110 Rhodopseudomonas palustris CGA009 Caulobacter vibrioides CB15 Rhodobacter sphaeroides 2.4.1 Paracoccus denitrificans Rhodospirillum rubrum ATCC 11170 Bacillus subtilis subsp. subtilis 168 Bacillus anthracis Ames Bacillus cereus ATCC 14579 Bacillus thuringiensis serovar konkukian 97-27 Staphylococcus aureus subsp. aureus N315 (MRSA/VSSA) Staphylococcus aureus subsp. aureus NCTC8325 Staphylococcus epidermidis RP62A (MRSE) Listeria monocytogenes EGD-e (serotype 1/2a) Listeria innocua (serotype 6a) Lactococcus lactis subsp. lactis Il1403 Lactococcus lactis subsp. cremoris MG1363 Streptococcus pyogenes M1 GAS (serotype M1) Streptococcus pneumoniae TIGR4 (virulent serotype 4) Streptococcus pneumoniae R6 (avirulent serotype 2) Streptococcus mutans UA159 (serotype c) Lactobacillus plantarum WCFS1 Lactobacillus johnsonii NCC 533 Lactobacillus casei BL23 Enterococcus faecalis V583 Clostridium acetobutylicum ATCC 824 Clostridium perfringens 13 Clostridium botulinum A ATCC 3502 Clostridioides difficile 630 Moorella thermoacetica ATCC 39073 Mycoplasma genitalium G37 Mycoplasma pneumoniae M129 Mycobacterium tuberculosis H37Rv Mycobacterium tuberculosis CDC1551 Mycobacterium leprae TN Mycolicibacterium smegmatis MC2 155 Mycobacteroides abscessus ATCC 19977 Corynebacterium glutamicum ATCC 13032 (Kyowa Hakko) Corynebacterium diphtheriae NCTC 13129 Rhodococcus jostii Streptomyces coelicolor Streptomyces avermitilis Streptomyces griseus Streptomyces cattleya NRRL 8057 = DSM 46488 Streptomyces glaucescens Kitasatospora setae Amycolatopsis mediterranei U32 Bifidobacterium longum NCC2705 Synechocystis sp. PCC 6803 Synechococcus elongatus PCC6301 Synechococcus elongatus PCC7942 Thermosynechococcus elongatus Prochlorococcus marinus subsp. marinus CCMP1375 Gloeobacter violaceus Nostoc sp. PCC 7120 Nostoc punctiforme Chloroflexus aurantiacus Deinococcus radiodurans Thermus thermophilus HB27 Chlamydia trachomatis D/UW-3/CX Chlamydia trachomatis 434/Bu Chlamydia pneumoniae CWL029 Borreliella burgdorferi B31 Treponema pallidum subsp. pallidum Nichols Treponema denticola Leptospira interrogans serovar Lai 56601 Fusobacterium nucleatum subsp. nucleatum ATCC 25586 Bacteroides thetaiotaomicron VPI-5482 Bacteroides fragilis YCH46 Flavobacterium psychrophilum JIP02/86 Chlorobaculum tepidum Aquifex aeolicus Hydrogenobacter thermophilus Thermotoga maritima MSB8 Methanocaldococcus jannaschii Methanococcus maripaludis S2 Methanothermobacter thermautotrophicus Archaeoglobus fulgidus DSM 4304 Thermoplasma acidophilum Pyrococcus furiosus DSM 3638 Thermococcus kodakarensis Methanosarcina barkeri Fusaro Halobacterium salinarum NRC-1 Haloarcula marismortui Haloferax volcanii Aeropyrum pernix Saccharolobus solfataricus P2 Sulfolobus acidocaldarius DSM 639 Pyrobaculum aerophilum Nitrosopumilus maritimus | 100% 70% 35% |

- **Category title**

- **Pathway module**
  - Carbohydrate metabolism
    - Other carbohydrate metabolism
      - M00114 Ascorbate biosynthesis, plants, glucose-6P => ascorbate
        - M00565 Trehalose biosynthesis, D-glucose 1P => trehalose
        - M00012 Glyoxylate cycle
        - M00532 Photorespiration
  - Lipid metabolism
    - Sterol biosynthesis
      - M00102 Ergocalciferol biosynthesis
    - Lipid metabolism
      - M00113 Jasmonic acid biosynthesis
  - Amino acid metabolism
    - Serine and threonine metabolism
      - M00018 Threonine biosynthesis, aspartate => homoserine => threonine
    - Cysteine and methionine metabolism
      - M00021 Cysteine biosynthesis, serine => cysteine
        - M00368 Ethylene biosynthesis, methionine => ethylene
    - Branched-chain amino acid metabolism
      - M00019 Valine/isoleucine biosynthesis, pyruvate => valine / 2-oxobutanoate => isoleucine
        - M00432 Leucine biosynthesis, 2-oxoisovalerate => 2-oxoisocaproate
    - Lysine metabolism
      - M00527 Lysine biosynthesis, DAP aminotransferase pathway, aspartate => lysine
    - Histidine metabolism
      - M00026 Histidine biosynthesis, PRPP => histidine
    - Aromatic amino acid metabolism
      - M00022 Shikimate pathway, phosphoenolpyruvate + erythrose-4P => chorismate
        - M00023 Tryptophan biosynthesis, chorismate => tryptophan
        - M00024 Phenylalanine biosynthesis, chorismate => phenylalanine
        - M00040 Tyrosine biosynthesis, prephanate => pretyrosine => tyrosine
    - Other amino acid metabolism
      - M00369 Cyanogenic glycoside biosynthesis, tyrosine => dhurrin
  - Metabolism of cofactors and vitamins
    - Cofactor and vitamin metabolism
      - M00125 Riboflavin biosynthesis, GTP => riboflavin/FMN/FAD
        - M00119 Pantothenate biosynthesis, valine/L-aspartate => pantothenate
        - M00868 Heme biosynthesis, animals and fungi, glycine => heme
        - M00121 Heme biosynthesis, plants and bacteria, glutamate => heme
        - M00846 Siroheme biosynthesis, glutamate => siroheme
        - M00847 Heme biosynthesis, archaea, siroheme => heme
        - M00117 Ubiquinone biosynthesis, prokaryotes, chorismate => ubiquinone
        - M00116 Menaquinone biosynthesis, chorismate => menaquinol
        - M00112 Tocopherol/tocotorienol biosynthesis
  - Biosynthesis of terpenoids and polyketides
    - Terpenoid backbone biosynthesis
      - M00095 C5 isoprenoid biosynthesis, mevalonate pathway
        - M00849 C5 isoprenoid biosynthesis, mevalonate pathway, archaea
        - M00096 C5 isoprenoid biosynthesis, non-mevalonate pathway
        - M00364 C10-C20 isoprenoid biosynthesis, bacteria
        - M00365 C10-C20 isoprenoid biosynthesis, archaea
        - M00366 C10-C20 isoprenoid biosynthesis, plants
        - M00367 C10-C20 isoprenoid biosynthesis, non-plant eukaryotes
    - Plant terpenoid biosynthesis
      - M00097 beta-Carotene biosynthesis, GGAP => beta-carotene
        - M00372 Abscisic acid biosynthesis, beta-carotene => abscisic acid
        - M00371 Castasterone biosynthesis, campesterol => castasterone
    - Macrolide biosynthesis
      - M00773 Tylosin biosynthesis, methylmalonyl-CoA + malonyl-CoA => tylactone => tylosin
        - M00774 Erythromycin biosynthesis, propanoyl-CoA + methylmalonyl-CoA => deoxyerythronolide B => erythromycin A/B
        - M00775 Oleandomycin biosynthesis, malonyl-CoA + methylmalonyl-CoA => 8,8a-deoxyoleandolide => oleandomycin
        - M00776 Pikromycin/methymycin biosynthesis, methylmalonyl-CoA + malonyl-CoA => narbonolide/10-deoxymethynolide => pikromycin/methymycin
        - M00777 Avermectin biosynthesis, 2-methylbutanoyl-CoA/isobutyryl-CoA => 6,8a-Seco-6,8a-deoxy-5-oxoavermectin 1a/1b aglycone => avermectin A1a/B1a/A1b/B1b
    - Enediyne biosynthesis
      - M00824 9-membered enediyne core biosynthesis, malonyl-CoA => 3-hydroxyhexadeca-4,6,8,10,12,14-hexaenoyl-ACP => 9-membered enediyne core
        - M00825 10-membered enediyne core biosynthesis, malonyl-CoA => 3-hydroxyhexadeca-4,6,8,10,12,14-hexaenoyl-ACP => 10-membered enediyne core
        - M00826 C-1027 benzoxazolinate moiety biosynthesis, chorismate => benzoxazolinyl-CoA
        - M00827 C-1027 beta-amino acid moiety biosynthesis, tyrosine => 3-chloro-4,5-dihydroxy-beta-phenylalanyl-PCP
        - M00828 Maduropeptin beta-hydroxy acid moiety biosynthesis, tyrosine => 3-(4-hydroxyphenyl)-3-oxopropanoyl-PCP
        - M00829 3,6-Dimethylsalicylyl-CoA biosynthesis, malonyl-CoA => 6-methylsalicylate => 3,6-dimethylsalicylyl-CoA
        - M00830 Neocarzinostatin naphthoate moiety biosynthesis, malonyl-CoA => 2-hydroxy-5-methyl-1-naphthoate => 2-hydroxy-7-methoxy-5-methyl-1-naphthoyl-CoA
        - M00831 Kedarcidin 2-hydroxynaphthoate moiety biosynthesis, malonyl-CoA => 3,6,8-trihydroxy-2-naphthoate => 3-hydroxy-7,8-dimethoxy-6-isopropoxy-2-naphthoyl-CoA
        - M00832 Kedarcidin 2-aza-3-chloro-beta-tyrosine moiety biosynthesis, azatyrosine => 2-aza-3-chloro-beta-tyrosyl-PCP
        - M00834 Calicheamicin orsellinate moiety biosynthesis, malonyl-CoA => orsellinate-ACP => 5-iodo-2,3-dimethoxyorsellinate-ACP
        - M00833 Calicheamicin biosynthesis, calicheamicinone => calicheamicin
    - Type II polyketide biosynthesis
      - M00778 Type II polyketide backbone biosynthesis, acyl-CoA + malonyl-CoA => polyketide
        - M00779 Dihydrokalafungin biosynthesis, octaketide => dihydrokalafungin
        - M00780 Tetracycline/oxytetracycline biosynthesis, pretetramide => tetracycline/oxytetracycline
        - M00823 Chlortetracycline biosynthesis, pretetramide => chlortetracycline
        - M00781 Nogalavinone/aklavinone biosynthesis, deoxynogalonate/deoxyaklanonate => nogalavinone/aklavinone
        - M00782 Mithramycin biosynthesis, 4-demethylpremithramycinone => mithramycin
        - M00783 Tetracenomycin C/8-demethyltetracenomycin C biosynthesis, tetracenomycin F2 => tetracenomycin C/8-demethyltetracenomycin C
        - M00784 Elloramycin biosynthesis, 8-demethyltetracenomycin C => elloramycin A
    - Polyketide sugar unit biosynthesis
      - M00793 dTDP-L-rhamnose biosynthesis
        - M00794 dTDP-6-deoxy-D-allose biosynthesis
        - M00795 dTDP-beta-L-noviose biosynthesis
        - M00796 dTDP-D-mycaminose biosynthesis
        - M00797 dTDP-D-desosamine biosynthesis
        - M00798 dTDP-L-mycarose biosynthesis
        - M00799 dTDP-L-oleandrose biosynthesis
        - M00800 dTDP-L-megosamine biosynthesis
        - M00801 dTDP-L-olivose biosynthesis
        - M00802 dTDP-D-forosamine biosynthesis
        - M00803 dTDP-D-angolosamine biosynthesis
  - Biosynthesis of other secondary metabolites
    - Biosynthesis of phytochemical compounds
      - M00039 Monolignol biosynthesis, phenylalanine/tyrosine => monolignol
        - M00137 Flavanone biosynthesis, phenylalanine => naringenin
        - M00138 Flavonoid biosynthesis, naringenin => pelargonidin
        - M00370 Glucosinolate biosynthesis, tryptophan => glucobrassicin
        - M00900 Cronin biosynthesis, zeaxanthin => crocin
        - M00894 Cannabidiol biosynthesis, malonyl-CoA => cannabidiol/dronabinol
        - M00902 Podophyllotoxin biosynthesis, coniferyl alcohol => podophyllotoxin
    - Biosynthesis of beta-lactams
      - M00672 Penicillin biosynthesis, aminoadipate + cycteine + valine => penicillin
        - M00673 Cephamycin C biosynthesis, aminoadipate + cycteine + valine => cephamycin C
        - M00675 Carbapenem-3-carboxylate biosynthesis, pyrroline-5-carboxylate + malonyl-CoA => carbapenem-3-carboxylate
        - M00736 Nocardicin A biosynthesis, L-pHPG + arginine + serine => nocardicin A
        - M00674 Clavaminate biosynthesis, arginine + glyceraldehyde-3P => clavaminate
    - Biosynthesis of other antibiotics
      - M00877 Kanosamine biosynthesis glucose 6-phosphate => kanosamine
        - M00889 Puromycin biosynthesis, ATP => puromycin
        - M00815 Validamycin A biosynthesis, sedoheptulopyranose-7P => validamycin A
        - M00904 Dapdiamides biosynthesis, L-2,3-diaminopropanoate => dapdiamide A/B/C
        - M00785 Cycloserine biosynthesis, arginine/serine => cycloserine
        - M00787 Bacilysin biosynthesis, prephenate => bacilysin
        - M00848 Aurachin biosynthesis, anthranilate => aurachin A
        - M00788 Terpentecin biosynthesis, GGAP => terpentecin
        - M00819 Pentalenolactone biosynthesis, farnesyl-PP => pentalenolactone
        - M00903 Fosfomycin biosynthesis, phosphoenolpyruvate => fosfomycin
        - M00890 Roseoflavin biosynthesis, FMN => roseoflavin
    - Biosynthesis of other fungal compounds
      - M00661 Paspaline biosynthesis, geranylgeranyl-PP + indoleglycerol phosphate => paspaline
        - M00786 Fumitremorgin alkaloid biosynthesis, tryptophan + proline => fumitremorgin C/A
        - M00893 Lovastatin biosynthesis, malonyl-CoA => lovastatin acid
        - M00891 Ditryptophenaline biosynthesis, tryptophan + phenylalanine => ditryptophenaline
        - M00901 Fumiquinazoline biosynthesis, tryptophan + alanine + anthranilate => fumiquinazoline
    - Biosynthesis of other bacterial compounds
      - M00814 Acarbose biosynthesis, sedoheptulopyranose-7P => acarbose
        - M00789 Rebeccamycin biosynthesis, tryptophan => rebeccamycin
        - M00790 Pyrrolnitrin biosynthesis, tryptophan => pyrrolnitrin
        - M00805 Staurosporine biosynthesis, tryptophan => staurosporine
        - M00808 Violacein biosynthesis, tryptophan => violacein
        - M00835 Pyocyanine biosynthesis, chorismate => pyocyanine
        - M00837 Prodigiosin biosynthesis, L-proline => prodigiosin
        - M00838 Undecylprodigiosin biosynthesis, L-proline => undecylprodigiosin
        - M00905 Grixazone biosynthesis, aspartate 4-semialdehyde => grixazone B
        - M00876 Staphyloferrin A biosynthesis, L-ornithine => staphyloferrin A
        - M00875 Staphyloferrin B biosynthesis, L-serine => staphyloferrin B
        - M00906 Ethynylserine biosynthesis, lysine => ethynylserine
