# Supplementary material for: Isolation, identification, and whole-genome sequencing of high-yield protease bacteria from Daqu of ZhangGong Laojiu
Source: PLoS One. 2022 Apr 26;17(4):e0264677. doi: 10.1371/journal.pone.0264677 (PMC9041807; doi:10.1371/journal.pone.0264677)
Supplement: S1 Raw data — (ZIP) [file pone.0264677.s002.zip › Raw data/GDD20120317-1_Bacillus_velezensis_Genome_result/4_Basic_Annot/KEGG/Bac_map/map01120.html]

KEGG PATHWAY: Microbial metabolism in diverse environments - Reference pathway


|  |  |
| --- | --- |
| **Microbial metabolism in diverse environments - Reference pathway** |  |

[
Pathway menu
| Organism menu
| Pathway entry
|

Hide module list

| User data mapping
|

Image (png) file

]

|  |  |
| --- | --- |
| Reference pathway Reference pathway (KO) Reference pathway (EC) Reference pathway (Reaction) -----< Sort below by alphabet >----- Escherichia coli K-12 MG1655 Escherichia coli K-12 W3110 Escherichia coli O157:H7 EDL933 (EHEC) Escherichia coli O157:H7 Sakai (EHEC) Salmonella enterica subsp. enterica serovar Typhi CT18 Salmonella enterica subsp. enterica serovar Typhimurium LT2 Salmonella enterica subsp. enterica serovar Typhimurium 14028S Shigella flexneri 301 (serotype 2a) Enterobacter cloacae subsp. cloacae ATCC 13047 Klebsiella pneumoniae subsp. pneumoniae MGH 78578 (serotype K52) Citrobacter rodentium Yersinia pestis CO92 (biovar Orientalis) Yersinia pestis KIM10+ (biovar Mediaevalis) Yersinia pseudotuberculosis IP 32953 (serotype I) Yersinia enterocolitica subsp. enterocolitica 8081 (serotype 0:8) Serratia marcescens SM39 Pectobacterium atrosepticum SCRI1043 Dickeya dadantii 3937 Erwinia amylovora CFBP1430 Buchnera aphidicola APS (Acyrthosiphon pisum) Haemophilus influenzae Rd KW20 (serotype d) Haemophilus ducreyi Pasteurella multocida subsp. multocida Pm70 Actinobacillus pleuropneumoniae L20 (serotype 5b) Xylella fastidiosa 9a5c Xanthomonas campestris pv. campestris ATCC 33913 Xanthomonas citri pv. citri 306 Xanthomonas oryzae pv. oryzae MAFF 311018 Vibrio cholerae O1 El Tor N16961 Vibrio vulnificus CMCP6 Vibrio parahaemolyticus RIMD 2210633 Aliivibrio fischeri ES114 Pseudomonas aeruginosa PAO1 Pseudomonas putida KT2440 Pseudomonas syringae pv. tomato DC3000 Pseudomonas syringae pv. syringae B728a Azotobacter vinelandii DJ Acinetobacter baumannii ATCC 17978 Acinetobacter baylyi Shewanella oneidensis Legionella pneumophila subsp. pneumophila Philadelphia 1 (serogroup 1) Methylococcus capsulatus Francisella tularensis subsp. tularensis SCHU S4 Neisseria meningitidis MC58 (serogroup B) Neisseria gonorrhoeae FA 1090 Ralstonia solanacearum GMI1000 Cupriavidus necator H16 Burkholderia mallei ATCC 23344 Burkholderia pseudomallei K96243 Paraburkholderia xenovorans LB400 Bordetella pertussis Tohama I Aromatoleum aromaticum Helicobacter pylori 26695 Helicobacter pylori J99 Campylobacter jejuni subsp. jejuni NCTC 11168 = ATCC 700819 Geobacter sulfurreducens PCA Desulfovibrio vulgaris Hildenborough Rickettsia prowazekii Madrid E Rickettsia conorii Wolbachia wMel (Drosophila melanogaster) Mesorhizobium japonicum MAFF 303099 Sinorhizobium meliloti 1021 Agrobacterium fabrum Rhizobium etli CFN 42 Rhizobium leguminosarum bv. viciae 3841 Brucella melitensis bv. 1 16M Brucella abortus 2308 Brucella suis 1330 Bradyrhizobium diazoefficiens USDA 110 Rhodopseudomonas palustris CGA009 Caulobacter vibrioides CB15 Rhodobacter sphaeroides 2.4.1 Paracoccus denitrificans Rhodospirillum rubrum ATCC 11170 Bacillus subtilis subsp. subtilis 168 Bacillus anthracis Ames Bacillus cereus ATCC 14579 Bacillus thuringiensis serovar konkukian 97-27 Staphylococcus aureus subsp. aureus N315 (MRSA/VSSA) Staphylococcus aureus subsp. aureus NCTC8325 Staphylococcus epidermidis RP62A (MRSE) Listeria monocytogenes EGD-e (serotype 1/2a) Listeria innocua (serotype 6a) Lactococcus lactis subsp. lactis Il1403 Lactococcus lactis subsp. cremoris MG1363 Streptococcus pyogenes M1 GAS (serotype M1) Streptococcus pneumoniae TIGR4 (virulent serotype 4) Streptococcus pneumoniae R6 (avirulent serotype 2) Streptococcus mutans UA159 (serotype c) Lactobacillus plantarum WCFS1 Lactobacillus johnsonii NCC 533 Lactobacillus casei BL23 Enterococcus faecalis V583 Clostridium acetobutylicum ATCC 824 Clostridium perfringens 13 Clostridium botulinum A ATCC 3502 Clostridioides difficile 630 Moorella thermoacetica ATCC 39073 Mycoplasma genitalium G37 Mycoplasma pneumoniae M129 Mycobacterium tuberculosis H37Rv Mycobacterium tuberculosis CDC1551 Mycobacterium leprae TN Mycolicibacterium smegmatis MC2 155 Mycobacteroides abscessus ATCC 19977 Corynebacterium glutamicum ATCC 13032 (Kyowa Hakko) Corynebacterium diphtheriae NCTC 13129 Rhodococcus jostii Streptomyces coelicolor Streptomyces avermitilis Streptomyces griseus Streptomyces cattleya NRRL 8057 = DSM 46488 Streptomyces glaucescens Kitasatospora setae Amycolatopsis mediterranei U32 Bifidobacterium longum NCC2705 Synechocystis sp. PCC 6803 Synechococcus elongatus PCC6301 Synechococcus elongatus PCC7942 Thermosynechococcus elongatus Prochlorococcus marinus subsp. marinus CCMP1375 Gloeobacter violaceus Nostoc sp. PCC 7120 Nostoc punctiforme Chloroflexus aurantiacus Deinococcus radiodurans Thermus thermophilus HB27 Chlamydia trachomatis D/UW-3/CX Chlamydia trachomatis 434/Bu Chlamydia pneumoniae CWL029 Borreliella burgdorferi B31 Treponema pallidum subsp. pallidum Nichols Treponema denticola Leptospira interrogans serovar Lai 56601 Fusobacterium nucleatum subsp. nucleatum ATCC 25586 Bacteroides thetaiotaomicron VPI-5482 Bacteroides fragilis YCH46 Flavobacterium psychrophilum JIP02/86 Chlorobaculum tepidum Aquifex aeolicus Hydrogenobacter thermophilus Thermotoga maritima MSB8 Methanocaldococcus jannaschii Methanococcus maripaludis S2 Methanothermobacter thermautotrophicus Archaeoglobus fulgidus DSM 4304 Thermoplasma acidophilum Pyrococcus furiosus DSM 3638 Thermococcus kodakarensis Methanosarcina barkeri Fusaro Halobacterium salinarum NRC-1 Haloarcula marismortui Haloferax volcanii Aeropyrum pernix Saccharolobus solfataricus P2 Sulfolobus acidocaldarius DSM 639 Pyrobaculum aerophilum Nitrosopumilus maritimus | 100% 70% 35% |

- **Category title**

- **Pathway module**
  - Carbohydrate metabolism
    - Central carbohydrate metabolism
      - M00004 Pentose phosphate pathway (Pentose phosphate cycle)
        - M00006 Pentose phosphate pathway, oxidative phase, glucose 6P => ribulose 5P
        - M00007 Pentose phosphate pathway, non-oxidative phase, fructose 6P => ribose 5P
        - M00580 Pentose phosphate pathway, archaea, fructose 6P => ribose 5P
        - M00008 Entner-Doudoroff pathway, glucose-6P => glyceraldehyde-3P + pyruvate
        - M00308 Semi-phosphorylative Entner-Doudoroff pathway, gluconate => glycerate-3P
        - M00309 Non-phosphorylative Entner-Doudoroff pathway, gluconate/galactonate => glycerate
    - Other carbohydrate metabolism
      - M00550 Ascorbate degradation, ascorbate => D-xylulose-5P
        - M00373 Ethylmalonyl pathway
        - M00740 Methylaspartate cycle
  - Energy metabolism
    - Carbon fixation
      - M00165 Reductive pentose phosphate cycle (Calvin cycle)
        - M00166 Reductive pentose phosphate cycle, ribulose-5P => glyceraldehyde-3P
        - M00167 Reductive pentose phosphate cycle, glyceraldehyde-3P => ribulose-5P
        - M00168 CAM (Crassulacean acid metabolism), dark
        - M00169 CAM (Crassulacean acid metabolism), light
        - M00172 C4-dicarboxylic acid cycle, NADP - malic enzyme type
        - M00171 C4-dicarboxylic acid cycle, NAD - malic enzyme type
        - M00170 C4-dicarboxylic acid cycle, phosphoenolpyruvate carboxykinase type
        - M00173 Reductive citrate cycle (Arnon-Buchanan cycle)
        - M00376 3-Hydroxypropionate bi-cycle
        - M00375 Hydroxypropionate-hydroxybutylate cycle
        - M00374 Dicarboxylate-hydroxybutyrate cycle
        - M00377 Reductive acetyl-CoA pathway (Wood-Ljungdahl pathway)
        - M00579 Phosphate acetyltransferase-acetate kinase pathway, acetyl-CoA => acetate
        - M00620 Incomplete reductive citrate cycle, acetyl-CoA => oxoglutarate
    - Methane metabolism
      - M00567 Methanogenesis, CO2 => methane
        - M00357 Methanogenesis, acetate => methane
        - M00356 Methanogenesis, methanol => methane
        - M00563 Methanogenesis, methylamine/dimethylamine/trimethylamine => methane
        - M00358 Coenzyme M biosynthesis
        - M00608 2-Oxocarboxylic acid chain extension, 2-oxoglutarate => 2-oxoadipate => 2-oxopimelate => 2-oxosuberate
        - M00174 Methane oxidation, methanotroph, methane => formaldehyde
        - M00346 Formaldehyde assimilation, serine pathway
        - M00345 Formaldehyde assimilation, ribulose monophosphate pathway
        - M00344 Formaldehyde assimilation, xylulose monophosphate pathway
        - M00378 F420 biosynthesis
        - M00422 Acetyl-CoA pathway, CO2 => acetyl-CoA
    - Nitrogen metabolism
      - M00175 Nitrogen fixation, nitrogen => ammonia
        - M00531 Assimilatory nitrate reduction, nitrate => ammonia
        - M00530 Dissimilatory nitrate reduction, nitrate => ammonia
        - M00529 Denitrification, nitrate => nitrogen
        - M00528 Nitrification, ammonia => nitrite
        - M00804 Complete nitrification, comammox, ammonia => nitrite => nitrate
    - Sulfur metabolism
      - M00176 Assimilatory sulfate reduction, sulfate => H2S
        - M00596 Dissimilatory sulfate reduction, sulfate => H2S
        - M00595 Thiosulfate oxidation by SOX complex, thiosulfate => sulfate
  - Amino acid metabolism
    - Serine and threonine metabolism
      - M00033 Ectoine biosynthesis, aspartate => ectoine
    - Aromatic amino acid metabolism
      - M00533 Homoprotocatechuate degradation, homoprotocatechuate => 2-oxohept-3-enedioate
        - M00545 Trans-cinnamate degradation, trans-cinnamate => acetyl-CoA
  - Metabolism of cofactors and vitamins
    - Cofactor and vitamin metabolism
      - M00810 Nicotine degradation, pyridine pathway, nicotine => 2,6-dihydroxypyridine/succinate semialdehyde
        - M00811 Nicotine degradation, pyrrolidine pathway, nicotine => succinate semialdehyde
        - M00622 Nicotinate degradation, nicotinate => fumarate
        - M00836 Coenzyme F430 biosynthesis, sirohydrochlorin => coenzyme F430
  - Xenobiotics biodegradation
    - Aromatics degradation
      - M00538 Toluene degradation, toluene => benzoate
        - M00537 Xylene degradation, xylene => methylbenzoate
        - M00419 Cymene degradation, p-cymene => p-cumate
        - M00547 Benzene/toluene degradation, benzene => catechol / toluene => 3-methylcatechol
        - M00548 Benzene degradation, benzene => catechol
        - M00551 Benzoate degradation, benzoate => catechol / methylbenzoate => methylcatechol
        - M00637 Anthranilate degradation, anthranilate => catechol
        - M00568 Catechol ortho-cleavage, catechol => 3-oxoadipate
        - M00569 Catechol meta-cleavage, catechol => acetyl-CoA / 4-methylcatechol => propanoyl-CoA
        - M00539 Cumate degradation, p-cumate => 2-oxopent-4-enoate + 2-methylpropanoate
        - M00543 Biphenyl degradation, biphenyl => 2-oxopent-4-enoate + benzoate
        - M00544 Carbazole degradation, carbazole => 2-oxopent-4-enoate + anthranilate
        - M00418 Toluene degradation, anaerobic, toluene => benzoyl-CoA
        - M00541 Benzoyl-CoA degradation, benzoyl-CoA => 3-hydroxypimeloyl-CoA
        - M00540 Benzoate degradation, cyclohexanecarboxylic acid =>pimeloyl-CoA
        - M00534 Naphthalene degradation, naphthalene => salicylate
        - M00638 Salicylate degradation, salicylate => gentisate
        - M00624 Terephthalate degradation, terephthalate => 3,4-dihydroxybenzoate
        - M00623 Phthalate degradation, phthalate => protocatechuate
        - M00636 Phthalate degradation, phthalate => protocatechuate
