# Supplementary material for: Isolation, identification, and whole-genome sequencing of high-yield protease bacteria from Daqu of ZhangGong Laojiu
Source: PLoS One. 2022 Apr 26;17(4):e0264677. doi: 10.1371/journal.pone.0264677 (PMC9041807; doi:10.1371/journal.pone.0264677)
Supplement: S1 Raw data — (ZIP) [file pone.0264677.s002.zip › Raw data/GDD20120317-1_Bacillus_velezensis_Genome_result/4_Basic_Annot/KEGG/Bac_map/map04112.html]

KEGG PATHWAY: Cell cycle - Caulobacter - Reference pathway


|  |  |
| --- | --- |
| **Cell cycle - Caulobacter - Reference pathway** |  |

[
Pathway menu
| Organism menu
| Pathway entry
| Show description
| User data mapping
]

Cell cycle progression of Caulobacter crescentus is implemented by an elaborate transcriptional regulatory network. During the life cycle, Caulobacter divides asymmetrically producing two distinct cell types with specialized development programs. The sessile stalked cell immediately initiates a new round of division cycle, whereas the motile swarmer cell remains in the G1 phase for a defined interval before differentiating into the stalked cell and entering into the S phase. The division process is centered around three master regulator proteins, DnaA, GcrA, and CtrA. DnaA and GcrA together drive the synthesis of proteins needed to complete DNA replication. CtrA controls the transcription of a diverse group of genes, which are required for essential morphological, metabolic, and cell cycle events.

|  |  |
| --- | --- |
| Reference pathway Reference pathway (KO) -----< Sort below by alphabet >----- Caulobacter vibrioides CB15 Caulobacter vibrioides NA1000 Caulobacter sp. K31 Caulobacter segnis Caulobacter henricii Caulobacter mirabilis Caulobacter flavus Caulobacter sp. FWC26 Phenylobacterium zucineum Phenylobacterium sp. HYN0004 | 184% 150% 122% 100% 82% 67% 55% |
