# Supplementary material for: Isolation, identification, and whole-genome sequencing of high-yield protease bacteria from Daqu of ZhangGong Laojiu
Source: PLoS One. 2022 Apr 26;17(4):e0264677. doi: 10.1371/journal.pone.0264677 (PMC9041807; doi:10.1371/journal.pone.0264677)
Supplement: S1 Raw data — (ZIP) [file pone.0264677.s002.zip › Raw data/GDD20120317-1_Bacillus_velezensis_Genome_result/4_Basic_Annot/KEGG/Bac_map/map05111.html]

KEGG PATHWAY: Biofilm formation - Vibrio cholerae - Reference pathway


|  |  |
| --- | --- |
| **Biofilm formation - Vibrio cholerae - Reference pathway** |  |

[
Pathway menu
| Organism menu
| Pathway entry
| Show description
| User data mapping
]

Surface colonization and subsequent biofilm formation and development provide numerous advantages to microorganisms. On the other hand, biofilm formation is an energetically costly process and therefore must be tightly regulated and plastic, enabling biofilm bacteria to be responsive to the various environmental cues. The quorum sensing (QS) pathway of Vibrio cholerae activates the expression of VpsR, VpsT and AphA (the main activators of biofilm formation) at low cell density and HapR (the main repressor) at high cell density. At low cell density, biofilm genes, including Vibrio polysaccharide (VPS) biosynthesis genes and the major extracellular matrix genes RbmA, RbmC, and Bap1, are expressed. The QS pathway also leads to induction of virulence factors such as toxin coregulated pilus (TCP) and cholera toxin (CTX), essential for colonization of the host and enterotoxicity, respectively. The VarS/VarA signaling system responds to an unknown environmental cue and represses biofilm production by process which involves the regulatory sRNAs CsrB, CsrC and CsrD and the RNA-binding protein CsrA, interfering with LuxO of the QS pathway. The second messenger cAMP acts as a repressor of biofilm formation. cAMP is generated by adenylate cyclase CyaA, and in complex with cAMP receptor protein (CRP), this nucleotide has been shown to upregulate HapR production through the positive regulation of the CAI-I synthase CqsA and the negative regulation of Fis. The second messenger c-di-GMP is a key signalling molecule controlling motility and biofilm matrix production. High cellular levels of c-di-GMP promote enhanced transcription of genes involved in biofilm formation, possibly by promoting VpsT-mediated transcriptional activation of vps genes.

|  |  |
| --- | --- |
| Reference pathway Reference pathway (KO) -----< Sort below by alphabet >----- Vibrio cholerae O1 El Tor N16961 Vibrio cholerae O1 El Tor FJ147 Vibrio cholerae O1 El Tor MS6 Vibrio cholerae O1 2010EL-1786 Vibrio cholerae O1 2012EL-2176 Vibrio cholerae O1 MJ-1236 Vibrio cholerae O1 IEC224 Vibrio cholerae O1 O395 Vibrio cholerae O1 O395 Vibrio cholerae O1 M66-2 Vibrio cholerae O1 LMA3984-4 Vibrio cholerae O49 1154-74 Vibrio cholerae O27 10432-62 Vibrio vulnificus CMCP6 Vibrio vulnificus YJ016 Vibrio vulnificus MO6-24/O Vibrio vulnificus 93U204 Vibrio parahaemolyticus RIMD 2210633 Vibrio parahaemolyticus BB22OP Vibrio parahaemolyticus O1:K33 CDC\_K4557 Vibrio parahaemolyticus O1:Kuk FDA\_R31 Vibrio parahaemolyticus UCM-V493 Vibrio campbellii Vibrio campbellii Vibrio alginolyticus Vibrio antiquarius Vibrio diabolicus Vibrio harveyi Vibrio natriegens Vibrio owensii Vibrio rotiferianus Vibrio tasmaniensis Vibrio sp. EJY3 Vibrio furnissii Vibrio nigripulchritudo Vibrio anguillarum 775 Vibrio anguillarum M3 Vibrio anguillarum NB10 Vibrio coralliilyticus RE98 Vibrio coralliilyticus OCN014 Vibrio tubiashii Vibrio fluvialis Vibrio mimicus Vibrio breoganii Vibrio scophthalmi Vibrio gazogenes Vibrio mediterranei Vibrio qinghaiensis Vibrio tapetis Vibrio alfacsensis Vibrio neocaledonicus Vibrio cyclitrophicus | 184% 150% 122% 100% 82% 67% 55% |
