# Supplementary material for: Isolation, identification, and whole-genome sequencing of high-yield protease bacteria from Daqu of ZhangGong Laojiu
Source: PLoS One. 2022 Apr 26;17(4):e0264677. doi: 10.1371/journal.pone.0264677 (PMC9041807; doi:10.1371/journal.pone.0264677)
Supplement: S1 Raw data — (ZIP) [file pone.0264677.s002.zip › Raw data/GDD20120317-1_Bacillus_velezensis_Genome_result/4_Basic_Annot/Nr/Bac.Nr.species.stat.pdf]

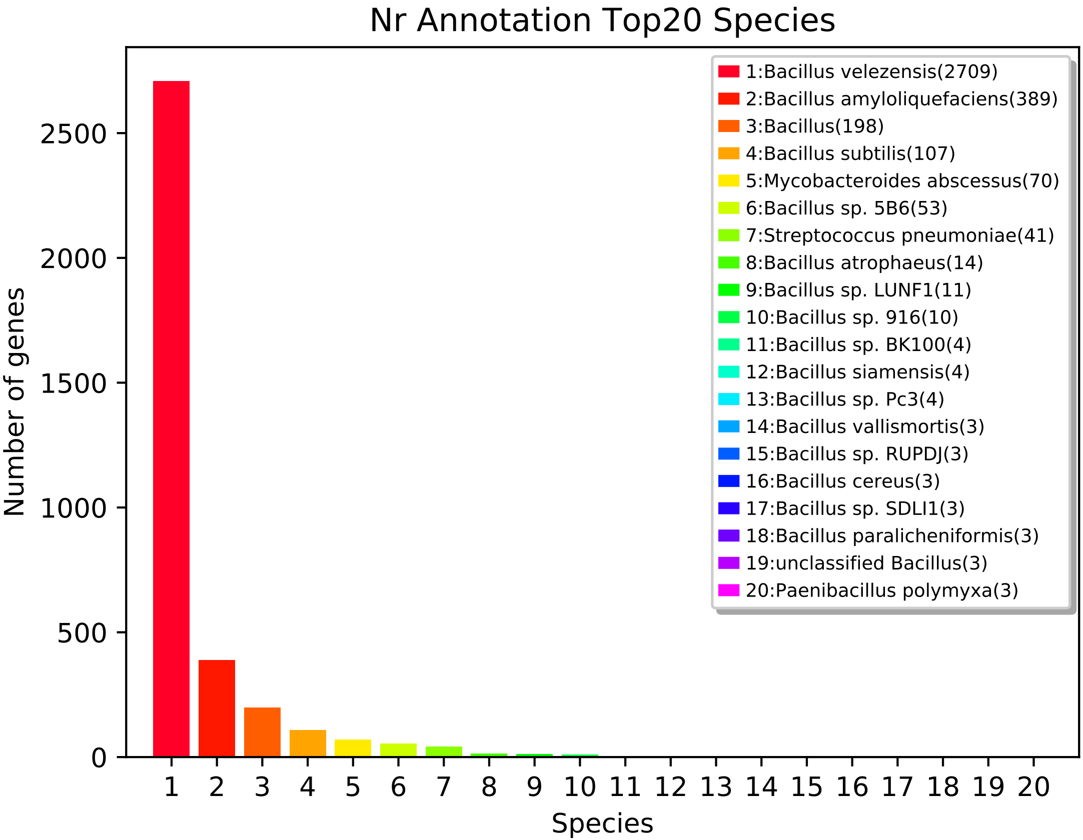

Nr Annotation Top20 Species
Number of genes
0
500
1000
1500
2000
2500
Species
1 2 3 4 5 6 7 8 9 10 11 12 13 14 15 16 17 18 19 20
1:Bacillus velezensis(2709)
2:Bacillus amyloliquefaciens(389)
3:Bacillus(198)
4:Bacillus subtilis(107)
5:Mycobacteroides abscessus(70)
6:Bacillus sp. 5B6(53)
7:Streptococcus pneumoniae(41)
8:Bacillus atrophaeus(14)
9:Bacillus sp. LUNF1(11)
10:Bacillus sp. 916(10)
11:Bacillus sp. BK100(4)
12:Bacillus siamensis(4)
13:Bacillus sp. Pc3(4)
14:Bacillus vallismortis(3)
15:Bacillus sp. RUPDJ(3)
16:Bacillus cereus(3)
17:Bacillus sp. SDLI1(3)
18:Bacillus paralicheniformis(3)
19:unclassified Bacillus(3)
20:Paenibacillus polymyxa(3)
