# Supplementary material for: Isolation, identification, and whole-genome sequencing of high-yield protease bacteria from Daqu of ZhangGong Laojiu
Source: PLoS One. 2022 Apr 26;17(4):e0264677. doi: 10.1371/journal.pone.0264677 (PMC9041807; doi:10.1371/journal.pone.0264677)
Supplement: S1 Raw data — (ZIP) [file pone.0264677.s002.zip › Raw data/GDD20120317-1_Bacillus_velezensis_Genome_result/5_Advanced_Annot/CAZy/Bac.CAZy.5class.xls.bar.pdf]

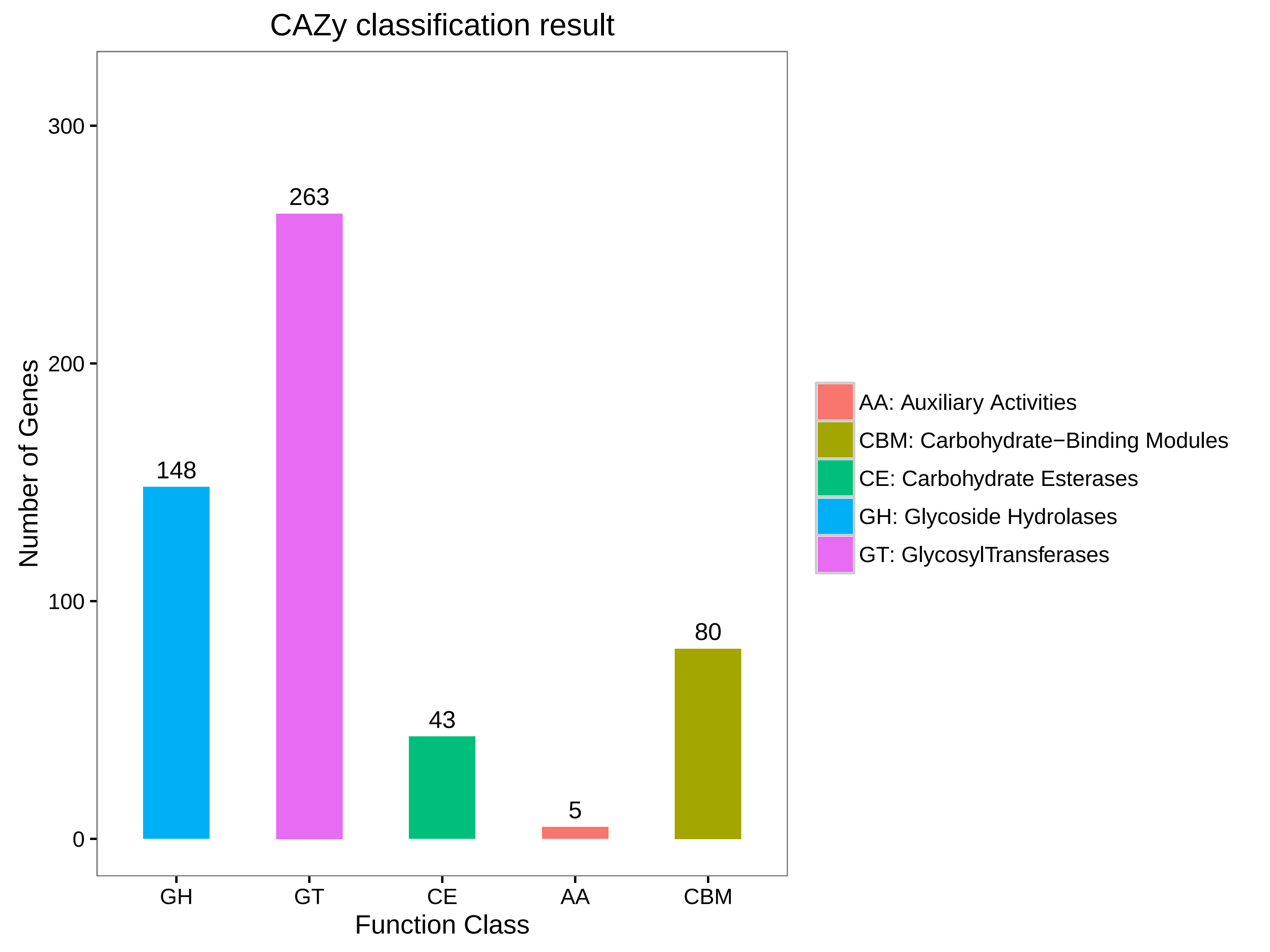

CAZy classification result
Number of Genes
300
200
100
0
148
263
43
5
80
GH
GT
CE
AA
CBM
Function Class
AA: Auxiliary Activities
CBM: Carbohydrate−Binding Modules
CE: Carbohydrate Esterases
GH: Glycoside Hydrolases
GT: GlycosylTransferases
