# Supplementary material for: Isolation, identification, and whole-genome sequencing of high-yield protease bacteria from Daqu of ZhangGong Laojiu
Source: PLoS One. 2022 Apr 26;17(4):e0264677. doi: 10.1371/journal.pone.0264677 (PMC9041807; doi:10.1371/journal.pone.0264677)
Supplement: S1 Raw data — (ZIP) [file pone.0264677.s002.zip › Raw data/GDD20120317-1_Bacillus_velezensis_Genome_result/index.html]

基迪奥生物 结题报告


基迪奥生物  细菌基因组分析  结题报告

收起
展开

### 1 项目概述

|  |  |
| --- | --- |
| 项目编号 | GDD20120317-1 |
| 物种名称 | Bacillus\_velezensis |
| 样品名称 | Bac |
| 测序策略 | PacBio + Illumina |

广州基迪奥生物科技有限公司


---

### 2 技术介绍

##### 2.1 实验流程

细菌基因组测序采用三代PacBio和二代Illumina两种技术相结合的方式。依赖三代测序长读长的特点，保证更完整的基因组组装，并使用二代测序数据校正，保证组装结果更精确可靠。实验流程如图。

Fig 2-1-1 实验流程图

  

##### 2.2 样本DNA质量检测

为保证测序质量，首先检测样品DNA质量，检测合格的样品用于构建测序文库。主要检测内容如下:

1. 浓度检测：以Qubit检测的DNA浓度为标准（包括后续的整个建库过程）， Nanodrop辅助检测（要求OD260/280在1.8-2.0之间）。若Nanodrop测的值大于Qubit值的两倍，表明样品所含杂质较高，在建库之前需要对样品进行纯化；假如两者测的值相差不大，一般可直接进行打断建库。
2. 电泳检测：进行1%的琼脂糖凝胶电泳检测样品DNA的完整性。

##### 2.3 PacBio测序

**技术简介**

目前第三代测序仪器主要是美国太平洋生物技术公司（ Pacific Biosciences）的RS II和Sequel，基于单分子实时测序系统（Single Molecule Real Time, SMRT），其专利单分子实时反应孔（SMRT Cell）含有15,0000个纳米级的零模式波导孔（zero-mode waveguides, ZMWs），每个ZMW能够包含一个DNA聚合酶及一条DNA样品链进行单分子扩增，并能够实时监测插入碱基的荧光信号（如图）。

Pacbio三代测序有以下优势：

1. 超长的测序读长：平均10-12kb，最长可超过30kb。
2. 通量高：RS II平台一个SMRT cell可产生约0.5~1Gb数据，而Sequel平台一个SMRT cell可产生5~10Gb数据，项目周期更短；
3. 无GC偏向性：不需要进行PCR扩增，避免了由扩增引起的偏向性和覆盖度不均一性。
4. 直接检测碱基修饰：可直接检测各种类型的DNA甲基化。

Fig 2-3-1 Pacbio示意图

  

**建库流程**

实验文库构建具体步骤如下：

1. 采用G-tubes 方法将基因组DNA 处理成8-10k的片段；
2. DNA片段末端修复；
3. 末端连接环状接头，得到类似哑铃（“套马环”）的结构，称为SMRT Bell；
4. 纯化DNA片段：ExoⅢ、Ⅳ酶切处理，去除有缺口或未和环状接头连接的DNA；
5. 纯化DNA片段：去除接头二聚体。为了提高测序质量，获得更长的平均插入片段，使用BluePippin（BP）核酸片段回收系统对文库进行片段选择，以去除短片段的文库分子。选择范围如下：

1. 对于浓度较低的文库或要求获得最大量的文库，BP 大小选择范围：4 k - 50 k;
2. 对于 > 2 ug的文库，BP大小选择范围一般为：7 k -50 k；
3. 对于 > 4 ug的文库且要求获得 >15 kb 的文库，BP大小选择范围是：10 k -50 k。

Fig 2-3-2 SMRT Bell示意图

  

BluePippin片段选择具体方法可参考网址: www.sagescience.com

构建好文库后，使用Qubit进行质量检测，并利用Agilent 2100评估插入片段大小，随后用PacBio平台进行测序。

**上机测序**

测序具体流程：

1. 退火，使引物与单链环结合，并固定在的零模式波导孔底部的聚合酶上。
2. 加入测序反应试剂，每个碱基配对合成后会发出相应的荧光信号，由高分辨率的光学系统实时检测。得到测序reads结果如图。

Fig 2-3-3 测序read示意图

  

##### 2.4 Illumina 测序

建库原理如图，具体步骤如下：

1. 使用DNA双链片段化酶将基因组DNA片段化；
2. 进行末端修复，磷酸化，加polyA尾；
3. 添加测序接头；
4. 使用AMPure XP磁珠纯化DNA片段，筛选目标大小（300-400bp）的插入片段；
5. PCR进行文库富集；
6. 使用AMPure XP磁珠纯化PCR产物，并使用Agilent 2100进行库检。

文库检测合格后使用Illumina 平台上机测序。

Fig 2-4-1 建库原理示意图

  

##### 2.5 信息分析流程

我们使用三代测序数据进行基因组组装，然后使用二代数据对组装结果进行校正。基于校正后的组装结果进行基因组组分分析，功能注释，比较基因组分析等，分析流程如图。

Fig 2-5-1 信息分析流程图

  

广州基迪奥生物科技有限公司


---

### 3 测序数据质控

测序的原始数据（raw data）存在一定比例的低质量数据，为了保证后续信息分析的准确可靠，需要对PacBio和Illumina测序的原始数据进行过滤处理，获得有效数据（clean data）。

##### 3.1 三代测序reads统计

对三代测序数据进行统计：

Tab 3-1-1 Reads信息统计表

| Sample | Read Bases | Number of Reads | Mean length | Reads N50 |
| --- | --- | --- | --- | --- |
| Bac | 1623703007 | 193753 | 8380.3 | 10090 |

  

##### 3.2 三代测序reads长度分布图

Fig 3-2-1 Subreads 长度分布图

  

##### 3.3 Illumina 测序reads统计

使用Fastp软件对Illumina测序数据进行质控，筛选标准：

1. 去除含有接头的reads（1.截去接头及接头后面的部分。2.如果截去后的reads长度小于50就丢弃该reads，否则保留。）；
2. 去除全部都是A碱基的reads；
3. 去除含N比例大于10%的reads；
4. 去除低质量的reads（质量值Q≤20的碱基数占整条reads的50%以上）。

过滤前后统计信息如下：

Tab 3-3-1 过滤前后信息统计

| Stat | Bases(bp) | Reads | Q20(%) | Q30(%) | N(%) | GC(%) |
| --- | --- | --- | --- | --- | --- | --- |
| Before filter | 1122772200 | 7485148 | 1094994710 (97.53%) | 1041392568 (92.75%) | 6269 (0.0%) | 520073049 (46.32%) |
| After filter | 1121596828 | 7478898 | 1094074676 (97.55%) | 1040580103 (92.78%) | 6261 (0.0%) | 519505496 (46.32%) |

  

过滤前后reads数量和碱基质量分布图如下：

|  |  |  |
| --- | --- | --- |
|  |  |  |
| Fig 3-3-1 各样品中reads分布图（reads数） |  | Fig 3-3-2 各样品中reads分布图（百分比） |
|  |  |  |

  

|  |  |  |
| --- | --- | --- |
|  |  |  |
| Fig 3-3-3 过滤前碱基组成分布图 |  | Fig 3-3-4 过滤后碱基组成分布图 |
|  |  |  |

  

广州基迪奥生物科技有限公司


---

### 4 基因组组装

##### 4.1 基因组组装

由于三代数据长度长，可以简化组装算法的复杂性，我们使用 Falcon 对三代测序reads进行拼接组装。

##### 4.2 组装结果

- 样本Bac 基因组序列： Bac.genome.fasta

##### 4.3 组装结果统计

Tab 4-3-1 组装结果统计表

| Sample | Total Number | Total length | Gap(N) | GC content |
| --- | --- | --- | --- | --- |
| Bac | 1 | 3942829 | 0 | 46.46% |

  

##### 4.4 基因组校正

三代测序reads更长，但是准确率不及二代，使用Pilon（version 1.23）将二代测序reads比对至组装好的基因组序列上，按软件默认参数对基因组结果进行校正，输出矫正后的基因组序列以及校正位点信息。

Tab 4-4-1 校正结果统计表

| Sample | Genome size(bp) | Correction(bp) | Insertion | Deletion | Substitution | Percent | Before GC(%) | After GC(%) |
| --- | --- | --- | --- | --- | --- | --- | --- | --- |
| Bac | 3942829 | 1543 | 794 | 645 | 104 | 0.04% | 46.46 | 46.46 |

  

##### 4.5 组装结果评估

1.将原始测序reads比对至组装结果，统计覆盖度和与GC分布，评估组装质量。

Fig 4-5-1 GC-Depth 分布图

  
2.组装结果末端和首端各取一段序列，两段序列拼在一起，将原始测序reads比对至拼接的序列，评估组装结果是否成环，即末端是否有缺失。

Fig 4-5-2 reads 比对图

  

##### 4.6 基因组圈图

针对组装的基因组序列，结合编码基因的预测结果，绘制基因组圈图，全面展示基因组的特征，如基因在正、反义链上的分布情况、基因的COG功能分类情况、GC含量，各种信息的综合展示可以使我们对菌株基因组的特征有更全面、更直观的认识。

Fig 4-6-1 基因组圈图

  

广州基迪奥生物科技有限公司


---

### 5 基因组组分分析

##### 5.1 编码基因预测

我们可提供2种预测方法，1）使用NCBI（National Center for Biotechnology Information Search database）进行基因预测，并提供组装结果上传NCBI数据库的服务。2）使用Prokka对基因组进行编码基因预测和注释，不提供上传服务。  
使用NCBI或Prokka进行编码基因预测，统计如下：

Tab 5-1-1 编码基因预测结果统计表

| Sample | Total Number | Total length | Maximum Length | Minimum Length | GC content |
| --- | --- | --- | --- | --- | --- |
| Bac | 3662 | 3402822 | 16299 | 75 | 47.32% |

  

- 样本Bac gene序列： Bac.gene.fasta

- 样本Bac protein序列： Bac.protein.fasta

编码基因长度分布:

Fig 5-1-1 基因序列长度分布图

  

##### 5.2 非编码RNA预测

非编码RNA（non-coding RNA，ncRNA）, 即不编码蛋白质的RNA分子，但可执行多种生物学功能，在RNA水平对生命活动发挥作用。其中tRNA、rRNA 、sRNA在微生物中的研究较为普遍。  
rRNA（ribosomal RNA），与蛋白质结合生成核糖体，在相邻物种中高度保守，包含5S，16S，23S三种类型，使用 RNAmmer 软件预测rRNA。  
tRNA（transfer RNA），携带并转运氨基酸，由70-90个核苷酸组成，使用tRNAscan 软件预测tRNA。  
sRNA（small RNA）：小RNA，细菌中，长度在50～500 nt的ncRNA通常定义为小RNA，具有多种调控功能，可调节转录本稳定性、激活/抑制蛋白表达等，如调控多种毒力因子，参与细胞的抗逆反应、碳以及氨基酸代谢等。cmscan 比对 Rfam database ，预测sRNA。结果如下：  
**rRNA预测**

通过RNAmmer软件预测rRNA

- 样本Bac rRNA注释信息： Bac.rRNA.xls

- 样本Bac rRNA 序列： Bac.rRNA.fasta

Tab 5-2-1 rRNA预测结果(前10行)

| ID | Contig | Start | End | Strand | Type |
| --- | --- | --- | --- | --- | --- |
| rRNA\_001 | CP077106 | 9350 | 10900 | + | 16S\_rRNA |
| rRNA\_002 | CP077106 | 11250 | 14179 | + | 23S\_rRNA |
| rRNA\_003 | CP077106 | 14230 | 14345 | + | 5S\_rRNA |
| rRNA\_004 | CP077106 | 31088 | 32634 | + | 16S\_rRNA |
| rRNA\_005 | CP077106 | 32984 | 35913 | + | 23S\_rRNA |
| rRNA\_006 | CP077106 | 35964 | 36079 | + | 5S\_rRNA |
| rRNA\_007 | CP077106 | 91120 | 92669 | + | 16S\_rRNA |
| rRNA\_008 | CP077106 | 92845 | 95771 | + | 23S\_rRNA |
| rRNA\_009 | CP077106 | 95822 | 95937 | + | 5S\_rRNA |
| rRNA\_010 | CP077106 | 96942 | 98491 | + | 16S\_rRNA |

  
**tRNA预测**

通过tRNAscan-SE软件预测tRNA区域和tRNA的二级结构

- 样本Bac tRNA注释信息： Bac.tRNA.xls

- 样本Bac tRNA 序列： Bac.tRNA.fasta

Tab 5-2-2 tRNA预测结果(前10行)

| ID | Contig | Begin | End | Type | Anti\_Codon | Cove\_Score |
| --- | --- | --- | --- | --- | --- | --- |
| tRNA\_001 | CP077106 | 11001 | 11077 | Ile | GAT | 102.39 |
| tRNA\_002 | CP077106 | 11092 | 11167 | Ala | TGC | 92.94 |
| tRNA\_003 | CP077106 | 23075 | 23167 | Ser | TGA | 72.49 |
| tRNA\_004 | CP077106 | 32735 | 32811 | Ile | GAT | 102.39 |
| tRNA\_005 | CP077106 | 32826 | 32901 | Ala | TGC | 92.94 |
| tRNA\_006 | CP077106 | 70804 | 70880 | Met | CAT | 83.99 |
| tRNA\_007 | CP077106 | 70890 | 70964 | Glu | TTC | 70.77 |
| tRNA\_008 | CP077106 | 95961 | 96036 | Val | TAC | 96.46 |
| tRNA\_009 | CP077106 | 96042 | 96117 | Thr | TGT | 92.93 |
| tRNA\_010 | CP077106 | 96154 | 96229 | Lys | TTT | 98.67 |

  
**sRNA预测**

- 样本Bac sRNA 预测结果： Bac.sRNA.xls

- 样本Bac sRNA 序列： Bac.sRNA.fasta

Tab 5-2-3 sRNA预测结果(前10行)

| ID | Contig | Start | End | Strand | Target\_Family | Target\_Acc | Score | E-value |
| --- | --- | --- | --- | --- | --- | --- | --- | --- |
| sRNA\_001 | CP077106 | 1464323 | 1464542 | + | SR1 | RF02376 | 198.5 | 2.2e-39 |
| sRNA\_002 | CP077106 | 2099499 | 2099711 | + | BsrG | RF01412 | 141.6 | 8.8e-33 |
| sRNA\_003 | CP077106 | 2112497 | 2112709 | + | BsrG | RF01412 | 141.6 | 8.8e-33 |
| sRNA\_004 | CP077106 | 556358 | 556556 | - | ncr1575 | RF02452 | 177.6 | 3.9e-30 |
| sRNA\_005 | CP077106 | 3525804 | 3526093 | + | 5\_ureB\_sRNA | RF02514 | 98.6 | 6.4e-24 |
| sRNA\_006 | CP077106 | 2517139 | 2517286 | + | SurC | RF02378 | 123.7 | 3e-23 |
| sRNA\_007 | CP077106 | 1119130 | 1119244 | + | RsaE | RF01820 | 123.1 | 1.1e-19 |
| sRNA\_008 | CP077106 | 1817145 | 1817264 | - | ncr1015 | RF02449 | 118.2 | 2.7e-17 |
| sRNA\_009 | CP077106 | 2021058 | 2021166 | + | BsrF | RF01411 | 101.0 | 6e-17 |
| sRNA\_010 | CP077106 | 514584 | 514670 | + | BsrC | RF01410 | 101.3 | 2.4e-16 |

  
**非编码RNA预测结果统计**

Tab 5-2-4 非编码RNA预测结果统计

| Type | Number | Average length(bp) | Total length | In genome(%) |
| --- | --- | --- | --- | --- |
| tRNA | 86 | 77 | 6639 | 0.17 |
| 5S\_rRNA | 9 | 116 | 1044 | 0.03 |
| 16S\_rRNA | 9 | 1549 | 13948 | 0.35 |
| 23S\_rRNA | 9 | 2930 | 26375 | 0.67 |
| sRNA | 17 | 131 | 2227 | 0.06 |

  

##### 5.3 散在重复序列预测

重复序列（repeated sequence）指在基因组中重复出现的DNA序列，根据重复的序列在基因组上的分布，分为串联重复序列（Tendam repeat）、散在重复序列（Interpersed repeat），其中串联重复序列成簇分布在染色体上，包括微卫星序列，小卫星序列和卫星DNA 等；散在重复序列又称转座子，分为长末端重复序列（Long terminal repeat sequence，LTR），短散在重复序列（short interspersed repeated sequences，SINEs）和长散在重复序列（long interspered repeated sequences，LINEs）。重复序列是基因调控网络的组成成分，影响生命的进化、遗传、变异，比如核算碱基的错配修复和损伤修复。很多细菌和病毒的复制起点都有同向重复、回文及简单重复序列。  
使用RepeatMasker 软件预测细菌基因组的重复序列。

- 样本Bac Repeat序列： Bac.repeat.fasta

Tab 5-3-1 散在重复序列预测结果统计

| Type | Number\_of\_elements | Length | Percentage\_of\_Sequence |
| --- | --- | --- | --- |
| SINEs | 22 | 1474 bp | 0.04 % |
| LINEs | 24 | 1869 bp | 0.05 % |
| LTR elements | 3 | 185 bp | 0.00 % |
| DNA elements | 13 | 876 bp | 0.02 % |
| Unclassified | 2 | 111 bp | 0.00 % |
| Total interspersed repeats | 64 | 4515 bp | 0.11 % |

  

Fig 5-3-1 散在重复序列柱状图

  

##### 5.4 串联重复序列预测

使用TRF 软件预测细菌基因组的串联重复序列（Tandem repeat）。

- 样本Bac Tandem Repeat预测结果表： Bac.Tandem\_Repeat.xls

Tab 5-4-1 串联重复序列预测结果(前10行)

| ID | Contig | Start | End | Repeat\_Size | Consensus\_Size | Copies\_Number | Score | Entropy | Consensus\_Pattern | Repeat\_Seq |
| --- | --- | --- | --- | --- | --- | --- | --- | --- | --- | --- |
| TR\_001 | CP077106 | 118181 | 118205 | 25 | 12 | 2.1 | 50 | 1.29 | GTTATCTTTTTT | GTTATCTTTTTTGTTATCTTTTTTG |
| TR\_002 | CP077106 | 121261 | 121291 | 31 | 15 | 2.1 | 53 | 1.64 | GCTGGCGGAGCTGCT | GCTGGCGGAGCTGCTGCTGGCGGCGCTGCTG |
| TR\_003 | CP077106 | 197739 | 197774 | 36 | 19 | 1.9 | 56 | 1.73 | AAAAGTCAAAACCAGGATC | AAAAGTCAAAACCAGGATCAAAATCAAGACCAGGAT |
| TR\_004 | CP077106 | 259900 | 259933 | 34 | 15 | 2.3 | 50 | 1.52 | AATTCAAGATAATTT | AATTCAAGATTTTTTAATTCAAGATAATTTAATT |
| TR\_005 | CP077106 | 315929 | 315977 | 49 | 23 | 2.2 | 66 | 1.97 | TGACGGCGTGAAAGTATCCCGGC | TGACGGCTGAAAGCTACCCGGCTGACGGCGTGAAAGTATTCCGGCTGAC |
| TR\_006 | CP077106 | 466439 | 466749 | 311 | 60 | 5.2 | 460 | 1.81 | AAAAAGGCGGAGAAGCAACAAGCAAAAATCATGACAAAGAATTCTATCAGGAAATCGGTC | AAAAAGGCGGAGAAGCGACAAGCAATAACCATGACAAAGAATTCTATCAGGAAATTGGTCAAAAAGGCGGAGAAGCAACAAGCAAAAATCACGACAAAGAATTCTATCAGGAAATCGGTGAAAAAGGCGGAGAAGCAACAAGCAAAAATCACGACAAAGAATTCTATCAGGAAATCGGTGAAAAAGGCGGAGAAGCAACAAGCAATAACCATGACAAGGATTTCTATCAAGAAATCGGCCAAAAAGGCGGAGAAGCAACAAGCAAAAATCATGACAAAGATTTTTATAAGGAAATCGGCCAAAAAGGCGGA |
| TR\_007 | CP077106 | 525120 | 525217 | 98 | 21 | 5.0 | 82 | 1.57 | AGCAAGAGCGGAAGAAGCAAG | AGCAAGAGCGGAAGCAGCAAGAGAAGCAGAAGCGAGAGCCAGAGCGGAAGAGGCAATAGCAAGAGCGGAAGAAGCAAGAGCCAGAGCAGCAGAAGCAA |
| TR\_008 | CP077106 | 694777 | 694857 | 81 | 42 | 1.9 | 117 | 1.87 | CGGAGGCTTCGGAGGATACCCGGGACACGGTTTCGGCGGCTA | CGGAGGCTTCGGAGGCTACCCGGGGTACGGTTTTGGCGGCTACGGAGGCTTCGGAGGATATCCGGGACACGGTTTCGGCGG |
| TR\_009 | CP077106 | 694879 | 694917 | 39 | 18 | 2.2 | 69 | 1.72 | CGGTTTTGGCGGCTACGG | CGGTTTTGGCGGTTACGGCGGTTTTGGCGGCTACGGCGG |
| TR\_010 | CP077106 | 737317 | 737351 | 35 | 18 | 1.9 | 52 | 1.67 | AGGAGCAACCGGAGCAAG | AGGAGTAACCGGAGCAAGAGGAGCGACCGGAGCAA |

  

Tab 5-4-2 串联重复序列预测结果统计

| Number\_of\_Repeats | Total\_Bases | Percentage\_of\_Sequence |
| --- | --- | --- |
| 147 | 10068 | 0.26% |

  

##### 5.5 转座子预测

转座子是存在于染色体DNA上可以自主复制和移位的基本单位。转座子可以传播到其他细菌细胞，是自然界中细菌产生抗药性的重要来源。  
使用软件TransposonPSI(version:20100822)进行转座因子预测。

Tab 5-5-1 转座子预测结果

| ID | Type | Chr | Star | End | Strand |
| --- | --- | --- | --- | --- | --- |
| chain00001 | helitronORF | CP077106 | 1569601 | 1569819 | + |
| chain00002 | LINE | CP077106 | 2140454 | 2140816 | + |

  

##### 5.6 CRISPR序列预测

CRISPR（Clustered Regularly Interspaced Short Palindromic Repeats），规律成簇的间隔短回文重复，是大多数细菌、古细菌的一种天然免疫系统，对外来的质粒和噬菌体序列具有抵抗作用，能识别并使入侵的功能元件沉默，是一种对付攻击者的基因武器。  
我们使用CRISPRfinder 软件对基因组进行CRISPR 预测。

Tab 5-6-1 CRISPR结果统计表

| Total CRISPR | Credible CRISPR | Questionable CRISPR |
| --- | --- | --- |
| 0 | 0 | 0 |

  

Tab 5-6-2 CRISPR详细信息表

| Crispr\_Id | CRISPR\_Start | CRISPR\_End | CRISPR\_Length | Potential\_Orientation | Consensus\_Repeat | Repeat\_Length | Spacers\_Nb | Evidence\_Level |
| --- | --- | --- | --- | --- | --- | --- | --- | --- |

  

##### 5.7 基因岛预测

基因岛（Gene Islands，GIs）是有横向起源迹象的一部分基因组。一个基因岛可以与多种生物功能相关，能与共生或病原机理相关，与生物体的适应性相关等。  
使用在线工具IslandViewer4 对基因组进行基因岛预测。该在线工具集成了四个不同的基因组岛预测方法：IslandPick、SIGI-HMM、IslandPath-DIMOB、Islander.

Tab 5-7-1 基因岛预测统计表

| GIs number | GIs total length(bp) | Average length(bp) |
| --- | --- | --- |
| 1 | 28970 | 28970.00 |

  

Tab 5-7-2 基因岛预测详细信息

| GI\_ID | Island start | Island end | Length | GeneID | Gene start | Gene end | Strand | Product |
| --- | --- | --- | --- | --- | --- | --- | --- | --- |
| GI\_1 | 2091024 | 2119993 | 28970 | KSG66\_09895 | 2091024 | 2092175 | - | 3-phytase |
| GI\_1 | 2091024 | 2119993 | 28970 | KSG66\_09900 | 2092491 | 2092916 | + | SDR family NAD(P)-dependent oxidoreductase |
| GI\_1 | 2091024 | 2119993 | 28970 | KSG66\_09905 | 2092950 | 2093126 | - | hypothetical protein |
| GI\_1 | 2091024 | 2119993 | 28970 | KSG66\_09915 | 2093489 | 2093845 | - | YobA family protein |
| GI\_1 | 2091024 | 2119993 | 28970 | KSG66\_09920 | 2094294 | 2094530 | + | hypothetical protein |
| GI\_1 | 2091024 | 2119993 | 28970 | KSG66\_09925 | 2094656 | 2095030 | + | hypothetical protein |
| GI\_1 | 2091024 | 2119993 | 28970 | KSG66\_09935 | 2096082 | 2097221 | + | aspartate phosphatase |
| GI\_1 | 2091024 | 2119993 | 28970 | KSG66\_09940 | 2097211 | 2097393 | + | hypothetical protein |
| GI\_1 | 2091024 | 2119993 | 28970 | KSG66\_09945 | 2097702 | 2098721 | + | hypothetical protein |
| GI\_1 | 2091024 | 2119993 | 28970 | KSG66\_09950 | 2098782 | 2099141 | - | hypothetical protein |

  

##### 5.8 前噬菌体预测

整合在宿主基因组上的温和噬菌体的核酸称为前噬菌体（prophage）。前噬菌体序列的存在可能允许一些细菌获取抗生素抗性，增强对环境的适应性，提高粘附力或使细菌成为致病菌。  
使用软件 Phage\_Finder 预测前噬菌体。

Tab 5-8-1 Prophage统计表

| Number | Total\_Length | Average\_Length |
| --- | --- | --- |
| 1 | 90882 | 90882.00 |

  

Tab 5-8-2 Prophage详细信息表

| ID | #asmbl\_id | genome\_size | genome\_gc | begin\_region | end\_region | size\_region | label | type | region\_gc | best\_db\_match | begin\_gene | end\_gene | region\_orientation | #genes |
| --- | --- | --- | --- | --- | --- | --- | --- | --- | --- | --- | --- | --- | --- | --- |
| Prophage\_001 | CP077106 | 3942829 | 46.46 | 1209156 | 1300037 | 90882 | Large | prophage | 46.74 | NC\_001884 | KSG66\_06220 | KSG66\_06715 | + | 99 |

  

广州基迪奥生物科技有限公司


---

### 6 基础功能注释

我们提供注释的功能数据库包含Nr、Swiss-Prot、GO、KEGG、COG。

Tab 6-0-1 四大数据库注释统计表

| Total Gene | Nr | Swissport | COG | Kegg | annotation gene | without annotation gene number |
| --- | --- | --- | --- | --- | --- | --- |
| 3662 | 3662 | 3399 | 2796 | 2283 | 3662 | 0 |

  

- 四大数据库注释结果汇总表： All\_Genes.basic.annotation.xls

Fig 6-0-1 四大数据库注释维恩图

  

##### 6.1 Nr注释

Nr，Non-redundant protein database，即非冗余的蛋白质数据库，由NCBI创建并维护，内容比较全面，同时注释结果中会包含物种信息，根据基因注释到的物种情况，统计注释到的物种及基因数目。  
使用 blastp 将基因编码的氨基酸序列和数据库比对获得注释信息。

- 样本Bac Nr比对结果： Bac.blast.Nr.xls

Tab 6-1-1 样本 Bac blast 比对 Nr 结果表(前10行)

| Query\_id | Query\_length | Query\_start | Query\_end | Subject\_id | Subject\_length | Subject\_start | Subject\_end | Identity(%) | Positive | Gap | Align\_length | Score | E\_value | Subject\_annotation | Confident |
| --- | --- | --- | --- | --- | --- | --- | --- | --- | --- | --- | --- | --- | --- | --- | --- |
| KSG66\_00005 | 446 | 1 | 446 | A7Z0C3.1 | 446 | 1 | 446 | 100 | 1 | 0 | 446 | 914 | 0.0 | RecName: Full=Chromosomal replication initiator protein DnaA [Bacillus velezensis FZB42] [Bacillus velezensis] | YES |
| KSG66\_00005 | 446 | 1 | 446 | WP\_144464666.1 | 446 | 1 | 446 | 99 | 1 | 0 | 446 | 914 | 0.0 | chromosomal replication initiator protein DnaA [Bacillus velezensis] | YES |
| KSG66\_00005 | 446 | 1 | 446 | WP\_052826951.1 | 446 | 1 | 446 | 99 | 1 | 0 | 446 | 912 | 0.0 | chromosomal replication initiator protein DnaA [Bacillus amyloliquefaciens] | YES |
| KSG66\_00005 | 446 | 1 | 446 | WP\_099566346.1 | 446 | 1 | 446 | 99 | 1 | 0 | 446 | 912 | 0.0 | chromosomal replication initiator protein DnaA [Bacillus velezensis] | YES |
| KSG66\_00005 | 446 | 1 | 446 | AFJ60096.1 | 446 | 1 | 446 | 99 | 1 | 0 | 446 | 912 | 0.0 | chromosomal replication initiator protein [Bacillus amyloliquefaciens Y2] [Bacillus amyloliquefaciens] | YES |
| KSG66\_00005 | 446 | 1 | 446 | WP\_115941228.1 | 446 | 1 | 446 | 99 | 1 | 0 | 446 | 912 | 0.0 | chromosomal replication initiator protein DnaA [Bacillus velezensis] | YES |
| KSG66\_00005 | 446 | 2 | 446 | WP\_140957157.1 | 447 | 3 | 447 | 100 | 1 | 0 | 445 | 911 | 0.0 | chromosomal replication initiator protein DnaA [Bacillus velezensis] | YES |
| KSG66\_00005 | 446 | 1 | 446 | WP\_101562620.1 | 446 | 1 | 446 | 99 | 1 | 0 | 446 | 911 | 0.0 | chromosomal replication initiator protein DnaA [Bacillus velezensis] | YES |
| KSG66\_00005 | 446 | 1 | 446 | WP\_013350722.1 | 446 | 1 | 446 | 99 | 0.99 | 0 | 446 | 910 | 0.0 | chromosomal replication initiator protein DnaA [Bacillus amyloliquefaciens] | YES |
| KSG66\_00005 | 446 | 1 | 446 | WP\_121984320.1 | 446 | 1 | 446 | 99 | 0.99 | 0 | 446 | 910 | 0.0 | chromosomal replication initiator protein DnaA [Bacillus velezensis] | YES |

  

Fig 6-1-1 Nr 数据库注释物种统计图(前20物种)

  

##### 6.2 SwissProt注释

SwissProt 是经过严格筛选的高质量蛋白数据库，其所含序列信息少于NR 数据库，但其包含的是经过人工认证过的序列，可信度比较高。使用 diamond 将基因编码的氨基酸序列和数据库比对获得注释信息。

- 样本Bac SwissProt比对结果： Bac.blast.SwissProt.lxs

Tab 6-2-1 样本 Bac blast 比对 SwissProt 结果表(前10行)

| Query\_id | Query\_length | Query\_start | Query\_end | Subject\_id | Subject\_length | Subject\_start | Subject\_end | Identity(%) | Align\_length | Mismatch | Gap | Score | E\_value | Subject\_annotation | Confident |
| --- | --- | --- | --- | --- | --- | --- | --- | --- | --- | --- | --- | --- | --- | --- | --- |
| KSG66\_00005 | 446 | 1 | 446 | sp\|A7Z0C3\|DNAA\_BACVZ | 446 | 1 | 446 | 100.0 | 446 | 0 | 0 | 873.2 | 1.2e-252 | sp\|A7Z0C3\|DNAA\_BACVZ Chromosomal replication initiator protein DnaA OS=Bacillus velezensis (strain DSM 23117 / BGSC 10A6 / FZB42) OX=326423 GN=dnaA PE=3 SV=1 | NO |
| KSG66\_00005 | 446 | 1 | 446 | sp\|P05648\|DNAA\_BACSU | 446 | 1 | 446 | 97.3 | 446 | 12 | 0 | 854.0 | 7.7e-247 | sp\|P05648\|DNAA\_BACSU Chromosomal replication initiator protein DnaA OS=Bacillus subtilis (strain 168) OX=224308 GN=dnaA PE=1 SV=1 | NO |
| KSG66\_00005 | 446 | 1 | 446 | sp\|Q65PM2\|DNAA\_BACLD | 446 | 1 | 446 | 95.1 | 446 | 22 | 0 | 839.7 | 1.5e-242 | sp\|Q65PM2\|DNAA\_BACLD Chromosomal replication initiator protein DnaA OS=Bacillus licheniformis (strain ATCC 14580 / DSM 13 / JCM 2505 / NBRC 12200 / NCIMB 9375 / NRRL NRS-1264 / Gibson 46) OX=279010 GN=dnaA PE=3 SV=1 | NO |
| KSG66\_00005 | 446 | 1 | 446 | sp\|A8F8Y4\|DNAA\_BACP2 | 446 | 1 | 446 | 93.7 | 446 | 28 | 0 | 828.9 | 2.6e-239 | sp\|A8F8Y4\|DNAA\_BACP2 Chromosomal replication initiator protein DnaA OS=Bacillus pumilus (strain SAFR-032) OX=315750 GN=dnaA PE=3 SV=1 | NO |
| KSG66\_00005 | 446 | 1 | 446 | sp\|Q5L3Z2\|DNAA\_GEOKA | 450 | 1 | 448 | 86.4 | 448 | 59 | 2 | 765.8 | 2.7e-220 | sp\|Q5L3Z2\|DNAA\_GEOKA Chromosomal replication initiator protein DnaA OS=Geobacillus kaustophilus (strain HTA426) OX=235909 GN=dnaA PE=3 SV=1 | NO |
| KSG66\_00005 | 446 | 1 | 446 | sp\|C5D327\|DNAA\_GEOSW | 450 | 1 | 448 | 86.4 | 448 | 59 | 2 | 763.8 | 1.0e-219 | sp\|C5D327\|DNAA\_GEOSW Chromosomal replication initiator protein DnaA OS=Geobacillus sp. (strain WCH70) OX=471223 GN=dnaA PE=3 SV=1 | NO |
| KSG66\_00005 | 446 | 1 | 446 | sp\|A4IJ84\|DNAA\_GEOTN | 450 | 1 | 448 | 86.4 | 448 | 59 | 2 | 763.5 | 1.4e-219 | sp\|A4IJ84\|DNAA\_GEOTN Chromosomal replication initiator protein DnaA OS=Geobacillus thermodenitrificans (strain NG80-2) OX=420246 GN=dnaA PE=3 SV=1 | NO |
| KSG66\_00005 | 446 | 1 | 446 | sp\|C1ES08\|DNAA\_BACC3 | 446 | 1 | 446 | 84.5 | 446 | 69 | 0 | 752.3 | 3.1e-216 | sp\|C1ES08\|DNAA\_BACC3 Chromosomal replication initiator protein DnaA OS=Bacillus cereus (strain 03BB102) OX=572264 GN=dnaA PE=3 SV=1 | NO |
| KSG66\_00005 | 446 | 1 | 446 | sp\|C3P8P5\|DNAA\_BACAA | 446 | 1 | 446 | 84.5 | 446 | 69 | 0 | 751.9 | 4.1e-216 | sp\|C3P8P5\|DNAA\_BACAA Chromosomal replication initiator protein DnaA OS=Bacillus anthracis (strain A0248) OX=592021 GN=dnaA PE=3 SV=1 | NO |
| KSG66\_00005 | 446 | 1 | 446 | sp\|C3LIC2\|DNAA\_BACAC | 446 | 1 | 446 | 84.5 | 446 | 69 | 0 | 751.9 | 4.1e-216 | sp\|C3LIC2\|DNAA\_BACAC Chromosomal replication initiator protein DnaA OS=Bacillus anthracis (strain CDC 684 / NRRL 3495) OX=568206 GN=dnaA PE=3 SV=1 | NO |

  

##### 6.3 KEGG注释

KEGG是系统分析基因产物在细胞中的代谢途径以及基因产物功能的数据库，利用 KEGG可以进一步研究基因在生物学上的复杂行为。根据 KEGG 注释信息得到Gene的 Pathway（代谢通路）注释，便于从系统的生物学过程了解微生物功能。  
使用DIAMOND与KEGG数据库比对，获得基因所对应的注释结果。

- 样本Bac Pathway 分析结果报告： Bac.htm

Tab 6-3-1 样本 Bac blast 比对 KEGG 结果表(前10行)

| Query\_id | Subject\_id | Identity | Align\_length | Miss\_match | Gap | Query\_start | Query\_end | Subject\_start | Subject\_end | E\_value | Score | Subject\_annotation | Confident |
| --- | --- | --- | --- | --- | --- | --- | --- | --- | --- | --- | --- | --- | --- |
| KSG66\_00005 | bay:RBAM\_000010 | 100.0 | 446 | 0 | 0 | 1 | 446 | 1 | 446 | 4.0e-251 | 873.2 | dnaA; chromosomal replication initiator protein DnaA; K02313 dnaA; chromosomal replication initiator protein | YES |
| KSG66\_00005 | bamp:B938\_00005 | 100.0 | 446 | 0 | 0 | 1 | 446 | 1 | 446 | 4.0e-251 | 873.2 | dnaA; chromosomal replication initiation protein; K02313 dnaA; chromosomal replication initiator protein | YES |
| KSG66\_00005 | baml:BAM5036\_0002 | 100.0 | 446 | 0 | 0 | 1 | 446 | 1 | 446 | 4.0e-251 | 873.2 | dnaA; chromosomal replication initiator protein DnaA; K02313 dnaA; chromosomal replication initiator protein | YES |
| KSG66\_00005 | bama:RBAU\_0002 | 100.0 | 446 | 0 | 0 | 1 | 446 | 1 | 446 | 4.0e-251 | 873.2 | dnaA; chromosomal replication initiator protein DnaA; K02313 dnaA; chromosomal replication initiator protein | YES |
| KSG66\_00005 | bamn:BASU\_0002 | 100.0 | 446 | 0 | 0 | 1 | 446 | 1 | 446 | 4.0e-251 | 873.2 | dnaA; chromosomal replication initiator protein DnaA; K02313 dnaA; chromosomal replication initiator protein | YES |
| KSG66\_00005 | bamt:AJ82\_00005 | 100.0 | 446 | 0 | 0 | 1 | 446 | 1 | 446 | 4.0e-251 | 873.2 | chromosomal replication initiation protein; K02313 dnaA; chromosomal replication initiator protein | YES |
| KSG66\_00005 | bamy:V529\_00010 | 100.0 | 446 | 0 | 0 | 1 | 446 | 1 | 446 | 4.0e-251 | 873.2 | dnaA; chromosomal replication initiation protein; K02313 dnaA; chromosomal replication initiator protein | YES |
| KSG66\_00005 | bamc:U471\_00010 | 100.0 | 446 | 0 | 0 | 1 | 446 | 1 | 446 | 4.0e-251 | 873.2 | chromosomal replication initiator protein DnaA; K02313 dnaA; chromosomal replication initiator protein | YES |
| KSG66\_00005 | bvm:B9C48\_00005 | 100.0 | 446 | 0 | 0 | 1 | 446 | 1 | 446 | 4.0e-251 | 873.2 | chromosomal replication initiator protein DnaA; K02313 dnaA; chromosomal replication initiator protein | YES |
| KSG66\_00005 | bacp:SB24\_09730 | 100.0 | 446 | 0 | 0 | 1 | 446 | 1 | 446 | 4.0e-251 | 873.2 | chromosomal replication initiation protein; K02313 dnaA; chromosomal replication initiator protein | YES |

  

Fig 6-3-1 KEGG注释结果统计图

  

##### 6.4 GO注释

我们根据 Nr 注释信息，使用 Blast2GO 得到基因的 GO 注释信息，并对所有基因做 GO 功能分类统计，从宏观上认识该物种的基因功能分布特征。

- 样本Bac GO注释表： Bac.gene2GO.xls

- 样本Bac GO分类表： Bac.GO2gene.xls

Tab 6-4-1 样本 Bac GO 注释结果(前10行)

| GO ID (level1) | GO Term (level1) | GO ID (level2) | GO Term (level2) | number\_of\_Bac (All) | Genes\_of\_Bac |
| --- | --- | --- | --- | --- | --- |
| GO:0008150 | Biological Process | GO:0008152 | metabolic process | 1287 | KSG66\_00005,KSG66\_00010,KSG66\_00020,KSG66\_00030,KSG66\_00035,KSG66\_00070,KSG66\_00075,KSG66\_00080,KSG66\_00085,KSG66\_00090,KSG66\_00105,KSG66\_00110,KSG66\_00115,KSG66\_00125,KSG66\_00145,KSG66\_00205,KSG66\_00220,KSG66\_00230,KSG66\_00250,KSG66\_00260,KSG66\_00265,KSG66\_00270,KSG66\_00275,KSG66\_00290,KSG66\_00295,KSG66\_00300,KSG66\_00305,KSG66\_00315,KSG66\_00320,KSG66\_00325,KSG66\_00330,KSG66\_00340,KSG66\_00345,KSG66\_00355,KSG66\_00395,KSG66\_00410,KSG66\_00415,KSG66\_00420,KSG66\_00425,KSG66\_00430,KSG66\_00440,KSG66\_00445,KSG66\_00450,KSG66\_00455,KSG66\_00460,KSG66\_00465,KSG66\_00470,KSG66\_00475,KSG66\_00485,KSG66\_00565,KSG66\_00575,KSG66\_00580,KSG66\_00585,KSG66\_00590,KSG66\_00595,KSG66\_00600,KSG66\_00605,KSG66\_00610,KSG66\_00615,KSG66\_00620,KSG66\_00625,KSG66\_00640,KSG66\_00645,KSG66\_00655,KSG66\_00660,KSG66\_00665,KSG66\_00670,KSG66\_00675,KSG66\_00680,KSG66\_00685,KSG66\_00690,KSG66\_00695,KSG66\_00700,KSG66\_00705,KSG66\_00710,KSG66\_00715,KSG66\_00725,KSG66\_00730,KSG66\_00735,KSG66\_00740,KSG66\_00745,KSG66\_00750,KSG66\_00755,KSG66\_00760,KSG66\_00765,KSG66\_00770,KSG66\_00775,KSG66\_00780,KSG66\_00785,KSG66\_00790,KSG66\_00795,KSG66\_00800,KSG66\_00805,KSG66\_00810,KSG66\_00815,KSG66\_00820,KSG66\_00825,KSG66\_00835,KSG66\_00840,KSG66\_00845,KSG66\_00850,KSG66\_00855,KSG66\_00860,KSG66\_00865,KSG66\_00870,KSG66\_00890,KSG66\_00895,KSG66\_00900,KSG66\_00920,KSG66\_00925,KSG66\_00940,KSG66\_01075,KSG66\_01115,KSG66\_01120,KSG66\_01135,KSG66\_01140,KSG66\_01200,KSG66\_01220,KSG66\_01285,KSG66\_01290,KSG66\_01310,KSG66\_01345,KSG66\_01350,KSG66\_01360,KSG66\_01390,KSG66\_01400,KSG66\_01410,KSG66\_01420,KSG66\_01485,KSG66\_01500,KSG66\_01505,KSG66\_01510,KSG66\_01545,KSG66\_01550,KSG66\_01555,KSG66\_01650,KSG66\_01685,KSG66\_01710,KSG66\_01715,KSG66\_01725,KSG66\_01730,KSG66\_01745,KSG66\_01750,KSG66\_01755,KSG66\_01760,KSG66\_01800,KSG66\_01815,KSG66\_01820,KSG66\_01825,KSG66\_01850,KSG66\_01860,KSG66\_01900,KSG66\_01940,KSG66\_01985,KSG66\_01990,KSG66\_01995,KSG66\_02010,KSG66\_02050,KSG66\_02055,KSG66\_02060,KSG66\_02065,KSG66\_02070,KSG66\_02080,KSG66\_02095,KSG66\_02105,KSG66\_02110,KSG66\_02115,KSG66\_02135,KSG66\_02165,KSG66\_02180,KSG66\_02185,KSG66\_02190,KSG66\_02200,KSG66\_02205,KSG66\_02215,KSG66\_02230,KSG66\_02235,KSG66\_02365,KSG66\_02375,KSG66\_02380,KSG66\_02410,KSG66\_02430,KSG66\_02435,KSG66\_02440,KSG66\_02445,KSG66\_02460,KSG66\_02465,KSG66\_02475,KSG66\_02485,KSG66\_02500,KSG66\_02505,KSG66\_02510,KSG66\_02515,KSG66\_02520,KSG66\_02525,KSG66\_02595,KSG66\_02600,KSG66\_02620,KSG66\_02665,KSG66\_02710,KSG66\_02725,KSG66\_02750,KSG66\_02760,KSG66\_02795,KSG66\_02840,KSG66\_02850,KSG66\_02880,KSG66\_02885,KSG66\_02925,KSG66\_02940,KSG66\_02945,KSG66\_02970,KSG66\_03010,KSG66\_03035,KSG66\_03045,KSG66\_03100,KSG66\_03115,KSG66\_03120,KSG66\_03130,KSG66\_03135,KSG66\_03160,KSG66\_03195,KSG66\_03200,KSG66\_03210,KSG66\_03250,KSG66\_03255,KSG66\_03275,KSG66\_03300,KSG66\_03345,KSG66\_03355,KSG66\_03360,KSG66\_03365,KSG66\_03370,KSG66\_03375,KSG66\_03385,KSG66\_03395,KSG66\_03400,KSG66\_03420,KSG66\_03425,KSG66\_03430,KSG66\_03435,KSG66\_03440,KSG66\_03465,KSG66\_03470,KSG66\_03475,KSG66\_03480,KSG66\_03550,KSG66\_03625,KSG66\_03630,KSG66\_03650,KSG66\_03655,KSG66\_03695,KSG66\_03710,KSG66\_03735,KSG66\_03750,KSG66\_03775,KSG66\_03780,KSG66\_03790,KSG66\_03835,KSG66\_03870,KSG66\_03875,KSG66\_03880,KSG66\_03885,KSG66\_03900,KSG66\_03915,KSG66\_03920,KSG66\_03935,KSG66\_03945,KSG66\_03980,KSG66\_04000,KSG66\_04005,KSG66\_04025,KSG66\_04030,KSG66\_04050,KSG66\_04055,KSG66\_04060,KSG66\_04065,KSG66\_04070,KSG66\_04080,KSG66\_04085,KSG66\_04090,KSG66\_04110,KSG66\_04120,KSG66\_04125,KSG66\_04160,KSG66\_04165,KSG66\_04170,KSG66\_04195,KSG66\_04215,KSG66\_04260,KSG66\_04270,KSG66\_04300,KSG66\_04305,KSG66\_04310,KSG66\_04430,KSG66\_04485,KSG66\_04495,KSG66\_04500,KSG66\_04510,KSG66\_04535,KSG66\_04555,KSG66\_04565,KSG66\_04580,KSG66\_04600,KSG66\_04640,KSG66\_04680,KSG66\_04685,KSG66\_04690,KSG66\_04705,KSG66\_04710,KSG66\_04735,KSG66\_04770,KSG66\_04785,KSG66\_04790,KSG66\_04795,KSG66\_04800,KSG66\_04805,KSG66\_04810,KSG66\_04815,KSG66\_04825,KSG66\_04840,KSG66\_04860,KSG66\_04865,KSG66\_04870,KSG66\_04900,KSG66\_04940,KSG66\_04995,KSG66\_05005,KSG66\_05035,KSG66\_05055,KSG66\_05100,KSG66\_05105,KSG66\_05110,KSG66\_05115,KSG66\_05125,KSG66\_05135,KSG66\_05175,KSG66\_05180,KSG66\_05215,KSG66\_05220,KSG66\_05230,KSG66\_05280,KSG66\_05285,KSG66\_05290,KSG66\_05295,KSG66\_05320,KSG66\_05410,KSG66\_05445,KSG66\_05470,KSG66\_05490,KSG66\_05535,KSG66\_05540,KSG66\_05545,KSG66\_05550,KSG66\_05565,KSG66\_05595,KSG66\_05605,KSG66\_05610,KSG66\_05650,KSG66\_05690,KSG66\_05750,KSG66\_05755,KSG66\_05760,KSG66\_05775,KSG66\_05780,KSG66\_05785,KSG66\_05790,KSG66\_05795,KSG66\_05800,KSG66\_05875,KSG66\_05960,KSG66\_05975,KSG66\_05990,KSG66\_06010,KSG66\_06015,KSG66\_06060,KSG66\_06070,KSG66\_06075,KSG66\_06085,KSG66\_06110,KSG66\_06120,KSG66\_06130,KSG66\_06135,KSG66\_06145,KSG66\_06220,KSG66\_06230,KSG66\_06250,KSG66\_06280,KSG66\_06425,KSG66\_06455,KSG66\_06515,KSG66\_06565,KSG66\_06570,KSG66\_06580,KSG66\_06615,KSG66\_06620,KSG66\_06660,KSG66\_06675,KSG66\_06685,KSG66\_06705,KSG66\_06715,KSG66\_06725,KSG66\_06750,KSG66\_06755,KSG66\_06765,KSG66\_06780,KSG66\_06785,KSG66\_06790,KSG66\_06795,KSG66\_06800,KSG66\_06805,KSG66\_06810,KSG66\_06840,KSG66\_06850,KSG66\_06855,KSG66\_06865,KSG66\_06870,KSG66\_06905,KSG66\_06915,KSG66\_06920,KSG66\_06925,KSG66\_06930,KSG66\_06935,KSG66\_06940,KSG66\_06945,KSG66\_06950,KSG66\_06960,KSG66\_06970,KSG66\_06975,KSG66\_07005,KSG66\_07055,KSG66\_07070,KSG66\_07075,KSG66\_07095,KSG66\_07105,KSG66\_07110,KSG66\_07115,KSG66\_07130,KSG66\_07155,KSG66\_07160,KSG66\_07170,KSG66\_07185,KSG66\_07190,KSG66\_07205,KSG66\_07210,KSG66\_07225,KSG66\_07230,KSG66\_07245,KSG66\_07305,KSG66\_07310,KSG66\_07315,KSG66\_07320,KSG66\_07340,KSG66\_07360,KSG66\_07370,KSG66\_07435,KSG66\_07445,KSG66\_07450,KSG66\_07455,KSG66\_07465,KSG66\_07470,KSG66\_07515,KSG66\_07520,KSG66\_07530,KSG66\_07550,KSG66\_07580,KSG66\_07600,KSG66\_07605,KSG66\_07610,KSG66\_07615,KSG66\_07620,KSG66\_07625,KSG66\_07645,KSG66\_07650,KSG66\_07655,KSG66\_07660,KSG66\_07710,KSG66\_07720,KSG66\_07730,KSG66\_07740,KSG66\_07745,KSG66\_07750,KSG66\_07755,KSG66\_07760,KSG66\_07765,KSG66\_07770,KSG66\_07775,KSG66\_07780,KSG66\_07790,KSG66\_07795,KSG66\_07805,KSG66\_07810,KSG66\_07835,KSG66\_07840,KSG66\_07845,KSG66\_07850,KSG66\_07860,KSG66\_07865,KSG66\_07870,KSG66\_07875,KSG66\_07880,KSG66\_07890,KSG66\_07895,KSG66\_07905,KSG66\_07915,KSG66\_07920,KSG66\_07925,KSG66\_07930,KSG66\_07935,KSG66\_07940,KSG66\_07945,KSG66\_07950,KSG66\_07955,KSG66\_07960,KSG66\_07980,KSG66\_07990,KSG66\_08005,KSG66\_08010,KSG66\_08015,KSG66\_08025,KSG66\_08040,KSG66\_08045,KSG66\_08055,KSG66\_08060,KSG66\_08065,KSG66\_08070,KSG66\_08075,KSG66\_08080,KSG66\_08115,KSG66\_08160,KSG66\_08235,KSG66\_08245,KSG66\_08250,KSG66\_08255,KSG66\_08260,KSG66\_08270,KSG66\_08275,KSG66\_08280,KSG66\_08285,KSG66\_08290,KSG66\_08300,KSG66\_08315,KSG66\_08325,KSG66\_08330,KSG66\_08335,KSG66\_08340,KSG66\_08345,KSG66\_08350,KSG66\_08355,KSG66\_08370,KSG66\_08375,KSG66\_08385,KSG66\_08410,KSG66\_08440,KSG66\_08465,KSG66\_08470,KSG66\_08480,KSG66\_08495,KSG66\_08500,KSG66\_08505,KSG66\_08520,KSG66\_08525,KSG66\_08535,KSG66\_08540,KSG66\_08545,KSG66\_08550,KSG66\_08555,KSG66\_08565,KSG66\_08605,KSG66\_08620,KSG66\_08660,KSG66\_08665,KSG66\_08685,KSG66\_08690,KSG66\_08700,KSG66\_08720,KSG66\_08725,KSG66\_08770,KSG66\_08775,KSG66\_08780,KSG66\_08785,KSG66\_08790,KSG66\_08815,KSG66\_08885,KSG66\_08915,KSG66\_08920,KSG66\_08925,KSG66\_08955,KSG66\_08990,KSG66\_08995,KSG66\_09030,KSG66\_09040,KSG66\_09065,KSG66\_09130,KSG66\_09150,KSG66\_09155,KSG66\_09160,KSG66\_09165,KSG66\_09170,KSG66\_09175,KSG66\_09200,KSG66\_09205,KSG66\_09210,KSG66\_09215,KSG66\_09220,KSG66\_09225,KSG66\_09275,KSG66\_09290,KSG66\_09300,KSG66\_09305,KSG66\_09315,KSG66\_09350,KSG66\_09360,KSG66\_09365,KSG66\_09370,KSG66\_09375,KSG66\_09395,KSG66\_09400,KSG66\_09405,KSG66\_09410,KSG66\_09430,KSG66\_09435,KSG66\_09470,KSG66\_09480,KSG66\_09500,KSG66\_09525,KSG66\_09535,KSG66\_09550,KSG66\_09600,KSG66\_09610,KSG66\_09635,KSG66\_09720,KSG66\_09725,KSG66\_09755,KSG66\_09760,KSG66\_09780,KSG66\_09785,KSG66\_09830,KSG66\_09895,KSG66\_09965,KSG66\_09975,KSG66\_10045,KSG66\_10055,KSG66\_10070,KSG66\_10075,KSG66\_10080,KSG66\_10125,KSG66\_10130,KSG66\_10150,KSG66\_10165,KSG66\_10170,KSG66\_10175,KSG66\_10185,KSG66\_10190,KSG66\_10215,KSG66\_10225,KSG66\_10250,KSG66\_10265,KSG66\_10290,KSG66\_10295,KSG66\_10300,KSG66\_10315,KSG66\_10355,KSG66\_10395,KSG66\_10400,KSG66\_10410,KSG66\_10415,KSG66\_10420,KSG66\_10425,KSG66\_10430,KSG66\_10440,KSG66\_10445,KSG66\_10450,KSG66\_10455,KSG66\_10460,KSG66\_10465,KSG66\_10470,KSG66\_10480,KSG66\_10485,KSG66\_10515,KSG66\_10520,KSG66\_10525,KSG66\_10545,KSG66\_10550,KSG66\_10555,KSG66\_10560,KSG66\_10565,KSG66\_10570,KSG66\_10575,KSG66\_10580,KSG66\_10585,KSG66\_10590,KSG66\_10595,KSG66\_10600,KSG66\_10610,KSG66\_10615,KSG66\_10620,KSG66\_10625,KSG66\_10630,KSG66\_10635,KSG66\_10645,KSG66\_10660,KSG66\_10680,KSG66\_10690,KSG66\_10715,KSG66\_10720,KSG66\_10755,KSG66\_10765,KSG66\_10790,KSG66\_10810,KSG66\_10830,KSG66\_10840,KSG66\_10845,KSG66\_10865,KSG66\_10870,KSG66\_10875,KSG66\_10880,KSG66\_10890,KSG66\_10900,KSG66\_10910,KSG66\_10950,KSG66\_10955,KSG66\_10960,KSG66\_10965,KSG66\_10970,KSG66\_10975,KSG66\_10980,KSG66\_11020,KSG66\_11025,KSG66\_11030,KSG66\_11050,KSG66\_11090,KSG66\_11120,KSG66\_11125,KSG66\_11130,KSG66\_11140,KSG66\_11215,KSG66\_11220,KSG66\_11235,KSG66\_11245,KSG66\_11250,KSG66\_11265,KSG66\_11360,KSG66\_11380,KSG66\_11385,KSG66\_11390,KSG66\_11440,KSG66\_11460,KSG66\_11475,KSG66\_11485,KSG66\_11495,KSG66\_11500,KSG66\_11505,KSG66\_11510,KSG66\_11525,KSG66\_11575,KSG66\_11580,KSG66\_11600,KSG66\_11605,KSG66\_11610,KSG66\_11620,KSG66\_11625,KSG66\_11630,KSG66\_11650,KSG66\_11660,KSG66\_11720,KSG66\_11735,KSG66\_11765,KSG66\_11795,KSG66\_11845,KSG66\_11865,KSG66\_11875,KSG66\_11880,KSG66\_11895,KSG66\_11910,KSG66\_11915,KSG66\_11920,KSG66\_11925,KSG66\_11930,KSG66\_11945,KSG66\_11950,KSG66\_11960,KSG66\_11965,KSG66\_11975,KSG66\_11980,KSG66\_11990,KSG66\_12035,KSG66\_12055,KSG66\_12060,KSG66\_12065,KSG66\_12070,KSG66\_12075,KSG66\_12085,KSG66\_12100,KSG66\_12105,KSG66\_12140,KSG66\_12145,KSG66\_12150,KSG66\_12160,KSG66\_12170,KSG66\_12190,KSG66\_12215,KSG66\_12225,KSG66\_12245,KSG66\_12275,KSG66\_12315,KSG66\_12320,KSG66\_12385,KSG66\_12405,KSG66\_12425,KSG66\_12455,KSG66\_12475,KSG66\_12480,KSG66\_12485,KSG66\_12505,KSG66\_12510,KSG66\_12525,KSG66\_12540,KSG66\_12550,KSG66\_12605,KSG66\_12620,KSG66\_12635,KSG66\_12640,KSG66\_12650,KSG66\_12655,KSG66\_12665,KSG66\_12670,KSG66\_12715,KSG66\_12720,KSG66\_12730,KSG66\_12735,KSG66\_12755,KSG66\_12760,KSG66\_12765,KSG66\_12780,KSG66\_12785,KSG66\_12795,KSG66\_12800,KSG66\_12810,KSG66\_12815,KSG66\_12850,KSG66\_12870,KSG66\_12875,KSG66\_12895,KSG66\_12900,KSG66\_12905,KSG66\_12910,KSG66\_12920,KSG66\_12945,KSG66\_12950,KSG66\_12960,KSG66\_12965,KSG66\_12970,KSG66\_12975,KSG66\_12980,KSG66\_12990,KSG66\_13020,KSG66\_13025,KSG66\_13035,KSG66\_13040,KSG66\_13055,KSG66\_13060,KSG66\_13065,KSG66\_13075,KSG66\_13080,KSG66\_13085,KSG66\_13095,KSG66\_13100,KSG66\_13105,KSG66\_13110,KSG66\_13115,KSG66\_13130,KSG66\_13140,KSG66\_13150,KSG66\_13155,KSG66\_13160,KSG66\_13165,KSG66\_13180,KSG66\_13185,KSG66\_13195,KSG66\_13215,KSG66\_13225,KSG66\_13230,KSG66\_13235,KSG66\_13240,KSG66\_13245,KSG66\_13260,KSG66\_13265,KSG66\_13270,KSG66\_13285,KSG66\_13290,KSG66\_13300,KSG66\_13315,KSG66\_13320,KSG66\_13325,KSG66\_13330,KSG66\_13335,KSG66\_13340,KSG66\_13350,KSG66\_13355,KSG66\_13365,KSG66\_13370,KSG66\_13375,KSG66\_13385,KSG66\_13390,KSG66\_13435,KSG66\_13440,KSG66\_13445,KSG66\_13450,KSG66\_13455,KSG66\_13460,KSG66\_13480,KSG66\_13490,KSG66\_13500,KSG66\_13505,KSG66\_13510,KSG66\_13520,KSG66\_13525,KSG66\_13530,KSG66\_13550,KSG66\_13555,KSG66\_13570,KSG66\_13575,KSG66\_13600,KSG66\_13615,KSG66\_13720,KSG66\_13730,KSG66\_13735,KSG66\_13740,KSG66\_13755,KSG66\_13760,KSG66\_13780,KSG66\_13800,KSG66\_13810,KSG66\_13820,KSG66\_13830,KSG66\_13845,KSG66\_13855,KSG66\_13860,KSG66\_13885,KSG66\_13915,KSG66\_13935,KSG66\_13950,KSG66\_13960,KSG66\_13985,KSG66\_13990,KSG66\_14025,KSG66\_14040,KSG66\_14060,KSG66\_14075,KSG66\_14080,KSG66\_14085,KSG66\_14095,KSG66\_14125,KSG66\_14155,KSG66\_14165,KSG66\_14180,KSG66\_14185,KSG66\_14190,KSG66\_14200,KSG66\_14215,KSG66\_14370,KSG66\_14395,KSG66\_14415,KSG66\_14425,KSG66\_14480,KSG66\_14485,KSG66\_14515,KSG66\_14530,KSG66\_14535,KSG66\_14545,KSG66\_14555,KSG66\_14570,KSG66\_14585,KSG66\_14685,KSG66\_14765,KSG66\_14805,KSG66\_14830,KSG66\_14840,KSG66\_14850,KSG66\_14855,KSG66\_14865,KSG66\_14875,KSG66\_14890,KSG66\_14895,KSG66\_14905,KSG66\_14955,KSG66\_14990,KSG66\_14995,KSG66\_15010,KSG66\_15030,KSG66\_15065,KSG66\_15070,KSG66\_15095,KSG66\_15105,KSG66\_15120,KSG66\_15195,KSG66\_15210,KSG66\_15230,KSG66\_15260,KSG66\_15270,KSG66\_15275,KSG66\_15280,KSG66\_15290,KSG66\_15300,KSG66\_15315,KSG66\_15335,KSG66\_15380,KSG66\_15385,KSG66\_15395,KSG66\_15410,KSG66\_15415,KSG66\_15440,KSG66\_15445,KSG66\_15495,KSG66\_15510,KSG66\_15515,KSG66\_15595,KSG66\_15620,KSG66\_15635,KSG66\_15640,KSG66\_15660,KSG66\_15665,KSG66\_15685,KSG66\_15695,KSG66\_15700,KSG66\_15705,KSG66\_15710,KSG66\_15745,KSG66\_15750,KSG66\_15790,KSG66\_15805,KSG66\_15815,KSG66\_15825,KSG66\_15830,KSG66\_15970,KSG66\_15975,KSG66\_15980,KSG66\_15985,KSG66\_15990,KSG66\_15995,KSG66\_16005,KSG66\_16010,KSG66\_16020,KSG66\_16080,KSG66\_16085,KSG66\_16090,KSG66\_16110,KSG66\_16175,KSG66\_16180,KSG66\_16195,KSG66\_16220,KSG66\_16280,KSG66\_16290,KSG66\_16320,KSG66\_16350,KSG66\_16355,KSG66\_16370,KSG66\_16390,KSG66\_16405,KSG66\_16410,KSG66\_16420,KSG66\_16425,KSG66\_16430,KSG66\_16435,KSG66\_16440,KSG66\_16445,KSG66\_16450,KSG66\_16455,KSG66\_16475,KSG66\_16480,KSG66\_16485,KSG66\_16490,KSG66\_16495,KSG66\_16535,KSG66\_16540,KSG66\_16550,KSG66\_16555,KSG66\_16585,KSG66\_16605,KSG66\_16615,KSG66\_16655,KSG66\_16685,KSG66\_16715,KSG66\_16720,KSG66\_16730,KSG66\_16745,KSG66\_16750,KSG66\_16755,KSG66\_16760,KSG66\_16765,KSG66\_16770,KSG66\_16775,KSG66\_16795,KSG66\_16800,KSG66\_16805,KSG66\_16830,KSG66\_16835,KSG66\_16850,KSG66\_16905,KSG66\_16935,KSG66\_16940,KSG66\_16945,KSG66\_16970,KSG66\_16975,KSG66\_16985,KSG66\_17015,KSG66\_17025,KSG66\_17030,KSG66\_17050,KSG66\_17055,KSG66\_17060,KSG66\_17080,KSG66\_17100,KSG66\_17105,KSG66\_17115,KSG66\_17140,KSG66\_17165,KSG66\_17170,KSG66\_17190,KSG66\_17195,KSG66\_17225,KSG66\_17235,KSG66\_17265,KSG66\_17275,KSG66\_17285,KSG66\_17290,KSG66\_17295,KSG66\_17300,KSG66\_17315,KSG66\_17320,KSG66\_17325,KSG66\_17350,KSG66\_17370,KSG66\_17375,KSG66\_17380,KSG66\_17385,KSG66\_17390,KSG66\_17395,KSG66\_17400,KSG66\_17405,KSG66\_17415,KSG66\_17420,KSG66\_17430,KSG66\_17435,KSG66\_17445,KSG66\_17470,KSG66\_17475,KSG66\_17485,KSG66\_17495,KSG66\_17500,KSG66\_17505,KSG66\_17510,KSG66\_17515,KSG66\_17520,KSG66\_17525,KSG66\_17530,KSG66\_17535,KSG66\_17545,KSG66\_17550,KSG66\_17560,KSG66\_17565,KSG66\_17580,KSG66\_17590,KSG66\_17595,KSG66\_17600,KSG66\_17610,KSG66\_17620,KSG66\_17630,KSG66\_17640,KSG66\_17655,KSG66\_17665,KSG66\_17670,KSG66\_17690,KSG66\_17695,KSG66\_17700,KSG66\_17725,KSG66\_17770,KSG66\_17775,KSG66\_17790,KSG66\_17795,KSG66\_17800,KSG66\_17810,KSG66\_17815,KSG66\_17820,KSG66\_17825,KSG66\_17840,KSG66\_17850,KSG66\_17860,KSG66\_17865,KSG66\_17885,KSG66\_17905,KSG66\_17915,KSG66\_17960,KSG66\_17985,KSG66\_18005,KSG66\_18015,KSG66\_18020,KSG66\_18045,KSG66\_18050,KSG66\_18055,KSG66\_18060,KSG66\_18075,KSG66\_18105,KSG66\_18110,KSG66\_18120,KSG66\_18125,KSG66\_18130,KSG66\_18160,KSG66\_18185,KSG66\_18190,KSG66\_18200,KSG66\_18205,KSG66\_18210,KSG66\_18225,KSG66\_18230,KSG66\_18240,KSG66\_18245,KSG66\_18250,KSG66\_18270,KSG66\_18280,KSG66\_18295,KSG66\_18310,KSG66\_18315,KSG66\_18380,KSG66\_18385,KSG66\_18390,KSG66\_18395,KSG66\_18420,KSG66\_18425,KSG66\_18475,KSG66\_18570,KSG66\_18575,KSG66\_18580,KSG66\_18585,KSG66\_18590,KSG66\_18600,KSG66\_18610,KSG66\_18615,KSG66\_18670,KSG66\_18675,KSG66\_18705,KSG66\_18715,KSG66\_18720,KSG66\_18730,KSG66\_18735,KSG66\_18740,KSG66\_18745,KSG66\_18750,KSG66\_18755,KSG66\_18760,KSG66\_18770,KSG66\_18780,KSG66\_18785,KSG66\_18790,KSG66\_18795,KSG66\_18815,KSG66\_18835,KSG66\_18840,KSG66\_18855,KSG66\_18915,KSG66\_18955,KSG66\_18960,KSG66\_18965,KSG66\_18975,KSG66\_18980,KSG66\_19045,KSG66\_19070,KSG66\_19135,KSG66\_19160,KSG66\_19180,KSG66\_19210,KSG66\_19220,KSG66\_19240,KSG66\_19250,KSG66\_19305,KSG66\_19310,KSG66\_19320,KSG66\_19325,KSG66\_19330,KSG66\_19335,KSG66\_19345,KSG66\_19385,KSG66\_19390,KSG66\_19395,KSG66\_19410,KSG66\_19415 |
| GO:0008150 | Biological Process | GO:0009987 | cellular process | 1189 | KSG66\_00005,KSG66\_00010,KSG66\_00020,KSG66\_00030,KSG66\_00035,KSG66\_00070,KSG66\_00075,KSG66\_00080,KSG66\_00085,KSG66\_00090,KSG66\_00105,KSG66\_00110,KSG66\_00125,KSG66\_00145,KSG66\_00205,KSG66\_00220,KSG66\_00230,KSG66\_00250,KSG66\_00260,KSG66\_00270,KSG66\_00275,KSG66\_00290,KSG66\_00295,KSG66\_00300,KSG66\_00305,KSG66\_00310,KSG66\_00315,KSG66\_00320,KSG66\_00340,KSG66\_00345,KSG66\_00375,KSG66\_00395,KSG66\_00410,KSG66\_00415,KSG66\_00420,KSG66\_00425,KSG66\_00430,KSG66\_00440,KSG66\_00445,KSG66\_00450,KSG66\_00455,KSG66\_00460,KSG66\_00465,KSG66\_00470,KSG66\_00475,KSG66\_00485,KSG66\_00565,KSG66\_00575,KSG66\_00580,KSG66\_00585,KSG66\_00590,KSG66\_00595,KSG66\_00600,KSG66\_00605,KSG66\_00610,KSG66\_00615,KSG66\_00620,KSG66\_00625,KSG66\_00640,KSG66\_00655,KSG66\_00685,KSG66\_00690,KSG66\_00710,KSG66\_00715,KSG66\_00730,KSG66\_00835,KSG66\_00840,KSG66\_00845,KSG66\_00855,KSG66\_00865,KSG66\_00890,KSG66\_00920,KSG66\_00925,KSG66\_00935,KSG66\_01020,KSG66\_01115,KSG66\_01140,KSG66\_01195,KSG66\_01200,KSG66\_01205,KSG66\_01220,KSG66\_01290,KSG66\_01345,KSG66\_01350,KSG66\_01360,KSG66\_01375,KSG66\_01385,KSG66\_01400,KSG66\_01410,KSG66\_01420,KSG66\_01495,KSG66\_01500,KSG66\_01505,KSG66\_01540,KSG66\_01545,KSG66\_01650,KSG66\_01685,KSG66\_01710,KSG66\_01715,KSG66\_01725,KSG66\_01730,KSG66\_01750,KSG66\_01755,KSG66\_01760,KSG66\_01810,KSG66\_01815,KSG66\_01820,KSG66\_01825,KSG66\_01850,KSG66\_01860,KSG66\_01900,KSG66\_01985,KSG66\_01990,KSG66\_01995,KSG66\_02010,KSG66\_02035,KSG66\_02050,KSG66\_02055,KSG66\_02060,KSG66\_02065,KSG66\_02070,KSG66\_02095,KSG66\_02105,KSG66\_02110,KSG66\_02115,KSG66\_02180,KSG66\_02190,KSG66\_02215,KSG66\_02230,KSG66\_02235,KSG66\_02320,KSG66\_02375,KSG66\_02380,KSG66\_02385,KSG66\_02425,KSG66\_02430,KSG66\_02435,KSG66\_02440,KSG66\_02445,KSG66\_02465,KSG66\_02475,KSG66\_02485,KSG66\_02500,KSG66\_02505,KSG66\_02515,KSG66\_02520,KSG66\_02525,KSG66\_02620,KSG66\_02665,KSG66\_02755,KSG66\_02795,KSG66\_02835,KSG66\_02840,KSG66\_02850,KSG66\_02880,KSG66\_02885,KSG66\_02920,KSG66\_02925,KSG66\_02940,KSG66\_02945,KSG66\_02970,KSG66\_02990,KSG66\_03010,KSG66\_03035,KSG66\_03060,KSG66\_03100,KSG66\_03115,KSG66\_03120,KSG66\_03130,KSG66\_03135,KSG66\_03140,KSG66\_03160,KSG66\_03165,KSG66\_03195,KSG66\_03210,KSG66\_03250,KSG66\_03300,KSG66\_03320,KSG66\_03345,KSG66\_03355,KSG66\_03360,KSG66\_03365,KSG66\_03370,KSG66\_03375,KSG66\_03385,KSG66\_03395,KSG66\_03400,KSG66\_03425,KSG66\_03430,KSG66\_03435,KSG66\_03440,KSG66\_03460,KSG66\_03465,KSG66\_03480,KSG66\_03550,KSG66\_03625,KSG66\_03675,KSG66\_03690,KSG66\_03710,KSG66\_03715,KSG66\_03735,KSG66\_03755,KSG66\_03775,KSG66\_03780,KSG66\_03790,KSG66\_03835,KSG66\_03870,KSG66\_03875,KSG66\_03880,KSG66\_03900,KSG66\_03915,KSG66\_03920,KSG66\_03925,KSG66\_03935,KSG66\_03945,KSG66\_03950,KSG66\_03965,KSG66\_03980,KSG66\_04000,KSG66\_04005,KSG66\_04020,KSG66\_04025,KSG66\_04050,KSG66\_04055,KSG66\_04060,KSG66\_04065,KSG66\_04070,KSG66\_04080,KSG66\_04085,KSG66\_04090,KSG66\_04105,KSG66\_04110,KSG66\_04120,KSG66\_04125,KSG66\_04155,KSG66\_04160,KSG66\_04195,KSG66\_04205,KSG66\_04260,KSG66\_04270,KSG66\_04290,KSG66\_04300,KSG66\_04310,KSG66\_04430,KSG66\_04485,KSG66\_04510,KSG66\_04535,KSG66\_04555,KSG66\_04565,KSG66\_04575,KSG66\_04600,KSG66\_04640,KSG66\_04680,KSG66\_04685,KSG66\_04690,KSG66\_04705,KSG66\_04710,KSG66\_04735,KSG66\_04780,KSG66\_04785,KSG66\_04790,KSG66\_04795,KSG66\_04800,KSG66\_04810,KSG66\_04850,KSG66\_04865,KSG66\_04870,KSG66\_04885,KSG66\_04890,KSG66\_04900,KSG66\_04940,KSG66\_04950,KSG66\_04995,KSG66\_05005,KSG66\_05035,KSG66\_05055,KSG66\_05065,KSG66\_05070,KSG66\_05100,KSG66\_05105,KSG66\_05110,KSG66\_05115,KSG66\_05125,KSG66\_05140,KSG66\_05210,KSG66\_05230,KSG66\_05260,KSG66\_05290,KSG66\_05300,KSG66\_05320,KSG66\_05405,KSG66\_05410,KSG66\_05445,KSG66\_05490,KSG66\_05530,KSG66\_05535,KSG66\_05540,KSG66\_05545,KSG66\_05550,KSG66\_05565,KSG66\_05595,KSG66\_05605,KSG66\_05610,KSG66\_05620,KSG66\_05630,KSG66\_05640,KSG66\_05650,KSG66\_05655,KSG66\_05660,KSG66\_05665,KSG66\_05675,KSG66\_05690,KSG66\_05705,KSG66\_05750,KSG66\_05755,KSG66\_05760,KSG66\_05770,KSG66\_05775,KSG66\_05780,KSG66\_05785,KSG66\_05790,KSG66\_05795,KSG66\_05800,KSG66\_05875,KSG66\_05960,KSG66\_05975,KSG66\_06010,KSG66\_06015,KSG66\_06060,KSG66\_06075,KSG66\_06085,KSG66\_06110,KSG66\_06120,KSG66\_06130,KSG66\_06145,KSG66\_06150,KSG66\_06220,KSG66\_06230,KSG66\_06250,KSG66\_06280,KSG66\_06285,KSG66\_06290,KSG66\_06425,KSG66\_06455,KSG66\_06515,KSG66\_06565,KSG66\_06570,KSG66\_06580,KSG66\_06615,KSG66\_06620,KSG66\_06660,KSG66\_06675,KSG66\_06685,KSG66\_06705,KSG66\_06715,KSG66\_06730,KSG66\_06750,KSG66\_06755,KSG66\_06765,KSG66\_06780,KSG66\_06785,KSG66\_06790,KSG66\_06795,KSG66\_06805,KSG66\_06810,KSG66\_06815,KSG66\_06820,KSG66\_06840,KSG66\_06850,KSG66\_06855,KSG66\_06865,KSG66\_06905,KSG66\_06920,KSG66\_06925,KSG66\_06930,KSG66\_06935,KSG66\_06940,KSG66\_06950,KSG66\_06960,KSG66\_06970,KSG66\_06975,KSG66\_06990,KSG66\_07005,KSG66\_07070,KSG66\_07075,KSG66\_07095,KSG66\_07105,KSG66\_07110,KSG66\_07115,KSG66\_07130,KSG66\_07140,KSG66\_07155,KSG66\_07160,KSG66\_07185,KSG66\_07205,KSG66\_07210,KSG66\_07225,KSG66\_07230,KSG66\_07305,KSG66\_07310,KSG66\_07315,KSG66\_07320,KSG66\_07340,KSG66\_07360,KSG66\_07435,KSG66\_07445,KSG66\_07450,KSG66\_07455,KSG66\_07465,KSG66\_07505,KSG66\_07520,KSG66\_07530,KSG66\_07580,KSG66\_07585,KSG66\_07600,KSG66\_07605,KSG66\_07610,KSG66\_07615,KSG66\_07620,KSG66\_07625,KSG66\_07630,KSG66\_07655,KSG66\_07660,KSG66\_07690,KSG66\_07705,KSG66\_07710,KSG66\_07730,KSG66\_07735,KSG66\_07740,KSG66\_07745,KSG66\_07750,KSG66\_07755,KSG66\_07760,KSG66\_07765,KSG66\_07770,KSG66\_07775,KSG66\_07780,KSG66\_07785,KSG66\_07790,KSG66\_07795,KSG66\_07805,KSG66\_07810,KSG66\_07835,KSG66\_07840,KSG66\_07845,KSG66\_07850,KSG66\_07860,KSG66\_07865,KSG66\_07870,KSG66\_07875,KSG66\_07880,KSG66\_07890,KSG66\_07895,KSG66\_07915,KSG66\_07930,KSG66\_07935,KSG66\_07940,KSG66\_07945,KSG66\_07950,KSG66\_07955,KSG66\_07960,KSG66\_07970,KSG66\_07980,KSG66\_07985,KSG66\_08005,KSG66\_08010,KSG66\_08025,KSG66\_08040,KSG66\_08045,KSG66\_08055,KSG66\_08060,KSG66\_08065,KSG66\_08075,KSG66\_08080,KSG66\_08085,KSG66\_08090,KSG66\_08095,KSG66\_08100,KSG66\_08105,KSG66\_08115,KSG66\_08120,KSG66\_08140,KSG66\_08150,KSG66\_08155,KSG66\_08160,KSG66\_08170,KSG66\_08175,KSG66\_08180,KSG66\_08190,KSG66\_08195,KSG66\_08200,KSG66\_08205,KSG66\_08210,KSG66\_08220,KSG66\_08235,KSG66\_08250,KSG66\_08255,KSG66\_08260,KSG66\_08270,KSG66\_08275,KSG66\_08285,KSG66\_08290,KSG66\_08300,KSG66\_08315,KSG66\_08325,KSG66\_08330,KSG66\_08335,KSG66\_08345,KSG66\_08375,KSG66\_08385,KSG66\_08410,KSG66\_08440,KSG66\_08465,KSG66\_08470,KSG66\_08480,KSG66\_08490,KSG66\_08495,KSG66\_08500,KSG66\_08505,KSG66\_08520,KSG66\_08525,KSG66\_08535,KSG66\_08540,KSG66\_08545,KSG66\_08550,KSG66\_08555,KSG66\_08565,KSG66\_08605,KSG66\_08660,KSG66\_08685,KSG66\_08690,KSG66\_08700,KSG66\_08720,KSG66\_08725,KSG66\_08775,KSG66\_08780,KSG66\_08785,KSG66\_08790,KSG66\_08885,KSG66\_08915,KSG66\_08920,KSG66\_08925,KSG66\_08955,KSG66\_08960,KSG66\_08990,KSG66\_09030,KSG66\_09040,KSG66\_09055,KSG66\_09090,KSG66\_09130,KSG66\_09150,KSG66\_09160,KSG66\_09165,KSG66\_09170,KSG66\_09175,KSG66\_09200,KSG66\_09205,KSG66\_09210,KSG66\_09215,KSG66\_09225,KSG66\_09300,KSG66\_09315,KSG66\_09325,KSG66\_09350,KSG66\_09355,KSG66\_09360,KSG66\_09365,KSG66\_09370,KSG66\_09395,KSG66\_09400,KSG66\_09405,KSG66\_09410,KSG66\_09425,KSG66\_09430,KSG66\_09470,KSG66\_09480,KSG66\_09500,KSG66\_09600,KSG66\_09610,KSG66\_09615,KSG66\_09620,KSG66\_09635,KSG66\_09670,KSG66\_09720,KSG66\_09725,KSG66\_09745,KSG66\_09760,KSG66\_09785,KSG66\_09830,KSG66\_09895,KSG66\_09965,KSG66\_10045,KSG66\_10070,KSG66\_10075,KSG66\_10080,KSG66\_10115,KSG66\_10125,KSG66\_10130,KSG66\_10150,KSG66\_10170,KSG66\_10175,KSG66\_10185,KSG66\_10215,KSG66\_10225,KSG66\_10240,KSG66\_10250,KSG66\_10285,KSG66\_10290,KSG66\_10300,KSG66\_10325,KSG66\_10355,KSG66\_10395,KSG66\_10400,KSG66\_10410,KSG66\_10415,KSG66\_10420,KSG66\_10440,KSG66\_10445,KSG66\_10450,KSG66\_10455,KSG66\_10460,KSG66\_10465,KSG66\_10480,KSG66\_10485,KSG66\_10520,KSG66\_10545,KSG66\_10550,KSG66\_10555,KSG66\_10560,KSG66\_10565,KSG66\_10570,KSG66\_10575,KSG66\_10580,KSG66\_10585,KSG66\_10590,KSG66\_10595,KSG66\_10600,KSG66\_10610,KSG66\_10615,KSG66\_10620,KSG66\_10625,KSG66\_10630,KSG66\_10635,KSG66\_10640,KSG66\_10645,KSG66\_10660,KSG66\_10680,KSG66\_10690,KSG66\_10710,KSG66\_10715,KSG66\_10720,KSG66\_10730,KSG66\_10755,KSG66\_10790,KSG66\_10805,KSG66\_10810,KSG66\_10830,KSG66\_10840,KSG66\_10845,KSG66\_10865,KSG66\_10870,KSG66\_10875,KSG66\_10880,KSG66\_10900,KSG66\_10910,KSG66\_10950,KSG66\_10955,KSG66\_10965,KSG66\_10970,KSG66\_10975,KSG66\_10980,KSG66\_10995,KSG66\_11015,KSG66\_11020,KSG66\_11025,KSG66\_11030,KSG66\_11090,KSG66\_11125,KSG66\_11140,KSG66\_11220,KSG66\_11250,KSG66\_11265,KSG66\_11360,KSG66\_11380,KSG66\_11385,KSG66\_11460,KSG66\_11475,KSG66\_11480,KSG66\_11485,KSG66\_11495,KSG66\_11500,KSG66\_11505,KSG66\_11510,KSG66\_11525,KSG66\_11575,KSG66\_11605,KSG66\_11610,KSG66\_11620,KSG66\_11625,KSG66\_11630,KSG66\_11650,KSG66\_11700,KSG66\_11710,KSG66\_11735,KSG66\_11780,KSG66\_11845,KSG66\_11865,KSG66\_11875,KSG66\_11895,KSG66\_11910,KSG66\_11915,KSG66\_11920,KSG66\_11930,KSG66\_11945,KSG66\_11950,KSG66\_11960,KSG66\_11965,KSG66\_11975,KSG66\_11980,KSG66\_11990,KSG66\_12000,KSG66\_12055,KSG66\_12060,KSG66\_12065,KSG66\_12070,KSG66\_12075,KSG66\_12085,KSG66\_12135,KSG66\_12140,KSG66\_12150,KSG66\_12160,KSG66\_12170,KSG66\_12215,KSG66\_12275,KSG66\_12310,KSG66\_12315,KSG66\_12320,KSG66\_12345,KSG66\_12405,KSG66\_12425,KSG66\_12455,KSG66\_12475,KSG66\_12480,KSG66\_12500,KSG66\_12505,KSG66\_12510,KSG66\_12540,KSG66\_12550,KSG66\_12605,KSG66\_12620,KSG66\_12635,KSG66\_12640,KSG66\_12650,KSG66\_12655,KSG66\_12665,KSG66\_12670,KSG66\_12690,KSG66\_12715,KSG66\_12720,KSG66\_12730,KSG66\_12735,KSG66\_12755,KSG66\_12760,KSG66\_12765,KSG66\_12780,KSG66\_12785,KSG66\_12795,KSG66\_12825,KSG66\_12830,KSG66\_12835,KSG66\_12850,KSG66\_12870,KSG66\_12875,KSG66\_12895,KSG66\_12900,KSG66\_12905,KSG66\_12910,KSG66\_12920,KSG66\_12930,KSG66\_12945,KSG66\_12950,KSG66\_12960,KSG66\_12965,KSG66\_12970,KSG66\_12975,KSG66\_12980,KSG66\_12990,KSG66\_13020,KSG66\_13025,KSG66\_13035,KSG66\_13040,KSG66\_13055,KSG66\_13065,KSG66\_13075,KSG66\_13080,KSG66\_13085,KSG66\_13105,KSG66\_13110,KSG66\_13115,KSG66\_13130,KSG66\_13140,KSG66\_13145,KSG66\_13150,KSG66\_13155,KSG66\_13160,KSG66\_13215,KSG66\_13230,KSG66\_13260,KSG66\_13270,KSG66\_13275,KSG66\_13280,KSG66\_13285,KSG66\_13290,KSG66\_13300,KSG66\_13315,KSG66\_13320,KSG66\_13325,KSG66\_13330,KSG66\_13335,KSG66\_13340,KSG66\_13345,KSG66\_13355,KSG66\_13365,KSG66\_13370,KSG66\_13375,KSG66\_13385,KSG66\_13390,KSG66\_13435,KSG66\_13440,KSG66\_13445,KSG66\_13450,KSG66\_13455,KSG66\_13460,KSG66\_13480,KSG66\_13500,KSG66\_13505,KSG66\_13510,KSG66\_13520,KSG66\_13525,KSG66\_13530,KSG66\_13555,KSG66\_13570,KSG66\_13575,KSG66\_13590,KSG66\_13600,KSG66\_13720,KSG66\_13730,KSG66\_13735,KSG66\_13740,KSG66\_13755,KSG66\_13760,KSG66\_13780,KSG66\_13800,KSG66\_13820,KSG66\_13830,KSG66\_13845,KSG66\_13855,KSG66\_13860,KSG66\_13870,KSG66\_13885,KSG66\_13895,KSG66\_13915,KSG66\_13950,KSG66\_13960,KSG66\_13965,KSG66\_13985,KSG66\_13990,KSG66\_14025,KSG66\_14060,KSG66\_14070,KSG66\_14075,KSG66\_14080,KSG66\_14140,KSG66\_14180,KSG66\_14185,KSG66\_14190,KSG66\_14200,KSG66\_14215,KSG66\_14370,KSG66\_14390,KSG66\_14415,KSG66\_14435,KSG66\_14440,KSG66\_14445,KSG66\_14515,KSG66\_14555,KSG66\_14570,KSG66\_14585,KSG66\_14650,KSG66\_14675,KSG66\_14685,KSG66\_14805,KSG66\_14830,KSG66\_14840,KSG66\_14855,KSG66\_14905,KSG66\_14955,KSG66\_14990,KSG66\_14995,KSG66\_15010,KSG66\_15030,KSG66\_15065,KSG66\_15070,KSG66\_15095,KSG66\_15105,KSG66\_15110,KSG66\_15120,KSG66\_15210,KSG66\_15235,KSG66\_15245,KSG66\_15260,KSG66\_15270,KSG66\_15280,KSG66\_15290,KSG66\_15300,KSG66\_15315,KSG66\_15320,KSG66\_15335,KSG66\_15385,KSG66\_15395,KSG66\_15410,KSG66\_15415,KSG66\_15440,KSG66\_15445,KSG66\_15470,KSG66\_15510,KSG66\_15595,KSG66\_15630,KSG66\_15635,KSG66\_15640,KSG66\_15645,KSG66\_15660,KSG66\_15665,KSG66\_15685,KSG66\_15705,KSG66\_15745,KSG66\_15790,KSG66\_15805,KSG66\_15815,KSG66\_15825,KSG66\_15830,KSG66\_15970,KSG66\_15975,KSG66\_15980,KSG66\_15985,KSG66\_15990,KSG66\_15995,KSG66\_16000,KSG66\_16005,KSG66\_16020,KSG66\_16030,KSG66\_16080,KSG66\_16085,KSG66\_16090,KSG66\_16110,KSG66\_16195,KSG66\_16220,KSG66\_16340,KSG66\_16350,KSG66\_16355,KSG66\_16370,KSG66\_16390,KSG66\_16395,KSG66\_16405,KSG66\_16410,KSG66\_16415,KSG66\_16420,KSG66\_16425,KSG66\_16430,KSG66\_16435,KSG66\_16440,KSG66\_16445,KSG66\_16450,KSG66\_16455,KSG66\_16475,KSG66\_16480,KSG66\_16485,KSG66\_16490,KSG66\_16495,KSG66\_16535,KSG66\_16540,KSG66\_16550,KSG66\_16555,KSG66\_16560,KSG66\_16575,KSG66\_16585,KSG66\_16595,KSG66\_16600,KSG66\_16615,KSG66\_16635,KSG66\_16640,KSG66\_16645,KSG66\_16655,KSG66\_16660,KSG66\_16670,KSG66\_16675,KSG66\_16680,KSG66\_16685,KSG66\_16715,KSG66\_16720,KSG66\_16730,KSG66\_16735,KSG66\_16740,KSG66\_16745,KSG66\_16750,KSG66\_16755,KSG66\_16760,KSG66\_16765,KSG66\_16770,KSG66\_16775,KSG66\_16785,KSG66\_16795,KSG66\_16800,KSG66\_16805,KSG66\_16830,KSG66\_16835,KSG66\_16840,KSG66\_16905,KSG66\_16935,KSG66\_16940,KSG66\_16975,KSG66\_16985,KSG66\_17025,KSG66\_17030,KSG66\_17055,KSG66\_17060,KSG66\_17080,KSG66\_17100,KSG66\_17105,KSG66\_17115,KSG66\_17140,KSG66\_17160,KSG66\_17165,KSG66\_17180,KSG66\_17185,KSG66\_17190,KSG66\_17195,KSG66\_17200,KSG66\_17210,KSG66\_17225,KSG66\_17235,KSG66\_17265,KSG66\_17285,KSG66\_17290,KSG66\_17295,KSG66\_17300,KSG66\_17315,KSG66\_17320,KSG66\_17350,KSG66\_17370,KSG66\_17375,KSG66\_17380,KSG66\_17385,KSG66\_17390,KSG66\_17395,KSG66\_17400,KSG66\_17405,KSG66\_17415,KSG66\_17420,KSG66\_17430,KSG66\_17435,KSG66\_17445,KSG66\_17470,KSG66\_17475,KSG66\_17485,KSG66\_17490,KSG66\_17495,KSG66\_17500,KSG66\_17510,KSG66\_17515,KSG66\_17520,KSG66\_17525,KSG66\_17530,KSG66\_17535,KSG66\_17545,KSG66\_17550,KSG66\_17560,KSG66\_17565,KSG66\_17575,KSG66\_17580,KSG66\_17590,KSG66\_17595,KSG66\_17600,KSG66\_17610,KSG66\_17620,KSG66\_17630,KSG66\_17640,KSG66\_17655,KSG66\_17665,KSG66\_17670,KSG66\_17695,KSG66\_17700,KSG66\_17725,KSG66\_17770,KSG66\_17775,KSG66\_17795,KSG66\_17805,KSG66\_17810,KSG66\_17815,KSG66\_17820,KSG66\_17825,KSG66\_17830,KSG66\_17840,KSG66\_17850,KSG66\_17860,KSG66\_17865,KSG66\_17960,KSG66\_17985,KSG66\_18005,KSG66\_18030,KSG66\_18045,KSG66\_18050,KSG66\_18055,KSG66\_18060,KSG66\_18075,KSG66\_18110,KSG66\_18120,KSG66\_18125,KSG66\_18130,KSG66\_18135,KSG66\_18160,KSG66\_18185,KSG66\_18190,KSG66\_18200,KSG66\_18205,KSG66\_18210,KSG66\_18240,KSG66\_18245,KSG66\_18250,KSG66\_18270,KSG66\_18280,KSG66\_18315,KSG66\_18370,KSG66\_18375,KSG66\_18390,KSG66\_18400,KSG66\_18425,KSG66\_18470,KSG66\_18570,KSG66\_18575,KSG66\_18580,KSG66\_18585,KSG66\_18590,KSG66\_18595,KSG66\_18600,KSG66\_18610,KSG66\_18615,KSG66\_18670,KSG66\_18675,KSG66\_18705,KSG66\_18715,KSG66\_18720,KSG66\_18730,KSG66\_18735,KSG66\_18740,KSG66\_18745,KSG66\_18750,KSG66\_18755,KSG66\_18765,KSG66\_18770,KSG66\_18780,KSG66\_18785,KSG66\_18790,KSG66\_18795,KSG66\_18840,KSG66\_18855,KSG66\_18915,KSG66\_18955,KSG66\_18960,KSG66\_18965,KSG66\_18975,KSG66\_18980,KSG66\_19045,KSG66\_19070,KSG66\_19160,KSG66\_19210,KSG66\_19220,KSG66\_19240,KSG66\_19250,KSG66\_19305,KSG66\_19310,KSG66\_19320,KSG66\_19330,KSG66\_19380,KSG66\_19385,KSG66\_19390,KSG66\_19395,KSG66\_19405,KSG66\_19410 |
| GO:0008150 | Biological Process | GO:0044699 | single-organism process | 944 | KSG66\_00020,KSG66\_00030,KSG66\_00070,KSG66\_00075,KSG66\_00085,KSG66\_00090,KSG66\_00105,KSG66\_00110,KSG66\_00205,KSG66\_00295,KSG66\_00300,KSG66\_00305,KSG66\_00310,KSG66\_00315,KSG66\_00320,KSG66\_00340,KSG66\_00375,KSG66\_00415,KSG66\_00420,KSG66\_00440,KSG66\_00445,KSG66\_00450,KSG66\_00455,KSG66\_00460,KSG66\_00465,KSG66\_00470,KSG66\_00485,KSG66\_00590,KSG66\_00600,KSG66\_00605,KSG66\_00610,KSG66\_00615,KSG66\_00620,KSG66\_00655,KSG66\_00835,KSG66\_00935,KSG66\_01020,KSG66\_01035,KSG66\_01040,KSG66\_01045,KSG66\_01140,KSG66\_01205,KSG66\_01290,KSG66\_01345,KSG66\_01350,KSG66\_01360,KSG66\_01375,KSG66\_01385,KSG66\_01395,KSG66\_01400,KSG66\_01410,KSG66\_01495,KSG66\_01505,KSG66\_01510,KSG66\_01550,KSG66\_01620,KSG66\_01625,KSG66\_01650,KSG66\_01665,KSG66\_01685,KSG66\_01710,KSG66\_01725,KSG66\_01730,KSG66\_01745,KSG66\_01750,KSG66\_01755,KSG66\_01760,KSG66\_01820,KSG66\_01825,KSG66\_01860,KSG66\_01875,KSG66\_01940,KSG66\_01950,KSG66\_01955,KSG66\_01960,KSG66\_01990,KSG66\_02010,KSG66\_02035,KSG66\_02050,KSG66\_02065,KSG66\_02070,KSG66\_02075,KSG66\_02080,KSG66\_02105,KSG66\_02110,KSG66\_02115,KSG66\_02165,KSG66\_02180,KSG66\_02185,KSG66\_02190,KSG66\_02200,KSG66\_02215,KSG66\_02265,KSG66\_02320,KSG66\_02365,KSG66\_02370,KSG66\_02375,KSG66\_02385,KSG66\_02410,KSG66\_02425,KSG66\_02430,KSG66\_02435,KSG66\_02440,KSG66\_02465,KSG66\_02475,KSG66\_02725,KSG66\_02750,KSG66\_02755,KSG66\_02760,KSG66\_02835,KSG66\_02880,KSG66\_02920,KSG66\_02925,KSG66\_02990,KSG66\_03040,KSG66\_03045,KSG66\_03060,KSG66\_03100,KSG66\_03140,KSG66\_03200,KSG66\_03255,KSG66\_03275,KSG66\_03300,KSG66\_03320,KSG66\_03345,KSG66\_03355,KSG66\_03360,KSG66\_03365,KSG66\_03370,KSG66\_03375,KSG66\_03385,KSG66\_03395,KSG66\_03400,KSG66\_03425,KSG66\_03430,KSG66\_03460,KSG66\_03630,KSG66\_03675,KSG66\_03695,KSG66\_03715,KSG66\_03735,KSG66\_03755,KSG66\_03775,KSG66\_03790,KSG66\_03835,KSG66\_03870,KSG66\_03875,KSG66\_03885,KSG66\_03900,KSG66\_03915,KSG66\_03920,KSG66\_03925,KSG66\_03950,KSG66\_03965,KSG66\_04000,KSG66\_04005,KSG66\_04020,KSG66\_04025,KSG66\_04050,KSG66\_04055,KSG66\_04060,KSG66\_04065,KSG66\_04080,KSG66\_04090,KSG66\_04105,KSG66\_04110,KSG66\_04120,KSG66\_04155,KSG66\_04205,KSG66\_04260,KSG66\_04270,KSG66\_04290,KSG66\_04305,KSG66\_04430,KSG66\_04460,KSG66\_04495,KSG66\_04575,KSG66\_04580,KSG66\_04615,KSG66\_04640,KSG66\_04680,KSG66\_04685,KSG66\_04690,KSG66\_04705,KSG66\_04770,KSG66\_04780,KSG66\_04800,KSG66\_04805,KSG66\_04825,KSG66\_04850,KSG66\_04865,KSG66\_04885,KSG66\_04890,KSG66\_04940,KSG66\_04950,KSG66\_04955,KSG66\_05055,KSG66\_05110,KSG66\_05125,KSG66\_05140,KSG66\_05180,KSG66\_05205,KSG66\_05210,KSG66\_05215,KSG66\_05220,KSG66\_05260,KSG66\_05300,KSG66\_05320,KSG66\_05405,KSG66\_05425,KSG66\_05445,KSG66\_05470,KSG66\_05530,KSG66\_05535,KSG66\_05540,KSG66\_05545,KSG66\_05550,KSG66\_05565,KSG66\_05605,KSG66\_05610,KSG66\_05620,KSG66\_05630,KSG66\_05640,KSG66\_05650,KSG66\_05655,KSG66\_05660,KSG66\_05665,KSG66\_05675,KSG66\_05705,KSG66\_05730,KSG66\_05750,KSG66\_05755,KSG66\_05770,KSG66\_05775,KSG66\_05780,KSG66\_05785,KSG66\_05790,KSG66\_05795,KSG66\_05800,KSG66\_05990,KSG66\_06005,KSG66\_06010,KSG66\_06060,KSG66\_06070,KSG66\_06075,KSG66\_06120,KSG66\_06125,KSG66\_06135,KSG66\_06455,KSG66\_06490,KSG66\_06495,KSG66\_06500,KSG66\_06505,KSG66\_06565,KSG66\_06570,KSG66\_06615,KSG66\_06620,KSG66\_06655,KSG66\_06730,KSG66\_06750,KSG66\_06765,KSG66\_06780,KSG66\_06785,KSG66\_06790,KSG66\_06795,KSG66\_06805,KSG66\_06815,KSG66\_06820,KSG66\_06855,KSG66\_06870,KSG66\_06905,KSG66\_06910,KSG66\_06930,KSG66\_06935,KSG66\_06940,KSG66\_06960,KSG66\_06970,KSG66\_06975,KSG66\_07005,KSG66\_07055,KSG66\_07070,KSG66\_07075,KSG66\_07140,KSG66\_07165,KSG66\_07185,KSG66\_07210,KSG66\_07220,KSG66\_07225,KSG66\_07305,KSG66\_07310,KSG66\_07315,KSG66\_07320,KSG66\_07360,KSG66\_07435,KSG66\_07445,KSG66\_07450,KSG66\_07455,KSG66\_07465,KSG66\_07470,KSG66\_07530,KSG66\_07585,KSG66\_07600,KSG66\_07605,KSG66\_07610,KSG66\_07615,KSG66\_07620,KSG66\_07625,KSG66\_07630,KSG66\_07690,KSG66\_07705,KSG66\_07710,KSG66\_07730,KSG66\_07735,KSG66\_07740,KSG66\_07745,KSG66\_07750,KSG66\_07755,KSG66\_07760,KSG66\_07765,KSG66\_07770,KSG66\_07775,KSG66\_07780,KSG66\_07785,KSG66\_07805,KSG66\_07810,KSG66\_07820,KSG66\_07835,KSG66\_07890,KSG66\_07895,KSG66\_07915,KSG66\_07920,KSG66\_07925,KSG66\_07935,KSG66\_07940,KSG66\_07945,KSG66\_07950,KSG66\_07955,KSG66\_07970,KSG66\_07985,KSG66\_08040,KSG66\_08045,KSG66\_08065,KSG66\_08085,KSG66\_08090,KSG66\_08095,KSG66\_08100,KSG66\_08105,KSG66\_08115,KSG66\_08120,KSG66\_08140,KSG66\_08150,KSG66\_08155,KSG66\_08160,KSG66\_08170,KSG66\_08175,KSG66\_08180,KSG66\_08190,KSG66\_08195,KSG66\_08200,KSG66\_08205,KSG66\_08210,KSG66\_08255,KSG66\_08260,KSG66\_08270,KSG66\_08275,KSG66\_08285,KSG66\_08300,KSG66\_08335,KSG66\_08370,KSG66\_08375,KSG66\_08385,KSG66\_08440,KSG66\_08490,KSG66\_08495,KSG66\_08500,KSG66\_08520,KSG66\_08525,KSG66\_08550,KSG66\_08685,KSG66\_08690,KSG66\_08725,KSG66\_08765,KSG66\_08775,KSG66\_08780,KSG66\_08785,KSG66\_08815,KSG66\_08885,KSG66\_08920,KSG66\_08955,KSG66\_08960,KSG66\_08990,KSG66\_08995,KSG66\_09030,KSG66\_09040,KSG66\_09055,KSG66\_09065,KSG66\_09090,KSG66\_09150,KSG66\_09175,KSG66\_09200,KSG66\_09205,KSG66\_09210,KSG66\_09215,KSG66\_09225,KSG66\_09275,KSG66\_09315,KSG66\_09325,KSG66\_09355,KSG66\_09365,KSG66\_09370,KSG66\_09375,KSG66\_09395,KSG66\_09400,KSG66\_09410,KSG66\_09425,KSG66\_09435,KSG66\_09550,KSG66\_09600,KSG66\_09610,KSG66\_09615,KSG66\_09620,KSG66\_09635,KSG66\_09670,KSG66\_09725,KSG66\_09745,KSG66\_09785,KSG66\_09830,KSG66\_10070,KSG66\_10075,KSG66\_10125,KSG66\_10130,KSG66\_10150,KSG66\_10165,KSG66\_10170,KSG66\_10175,KSG66\_10240,KSG66\_10285,KSG66\_10290,KSG66\_10325,KSG66\_10355,KSG66\_10395,KSG66\_10400,KSG66\_10410,KSG66\_10420,KSG66\_10445,KSG66\_10480,KSG66\_10485,KSG66\_10515,KSG66\_10520,KSG66\_10525,KSG66\_10545,KSG66\_10550,KSG66\_10555,KSG66\_10560,KSG66\_10565,KSG66\_10570,KSG66\_10575,KSG66\_10580,KSG66\_10585,KSG66\_10590,KSG66\_10595,KSG66\_10600,KSG66\_10610,KSG66\_10615,KSG66\_10620,KSG66\_10625,KSG66\_10635,KSG66\_10645,KSG66\_10660,KSG66\_10680,KSG66\_10690,KSG66\_10720,KSG66\_10730,KSG66\_10765,KSG66\_10770,KSG66\_10790,KSG66\_10810,KSG66\_10830,KSG66\_10840,KSG66\_10845,KSG66\_10865,KSG66\_10870,KSG66\_10875,KSG66\_10880,KSG66\_10910,KSG66\_10965,KSG66\_10970,KSG66\_10975,KSG66\_10980,KSG66\_10995,KSG66\_11015,KSG66\_11020,KSG66\_11025,KSG66\_11050,KSG66\_11125,KSG66\_11130,KSG66\_11140,KSG66\_11235,KSG66\_11310,KSG66\_11315,KSG66\_11320,KSG66\_11360,KSG66\_11380,KSG66\_11385,KSG66\_11440,KSG66\_11460,KSG66\_11475,KSG66\_11485,KSG66\_11505,KSG66\_11525,KSG66\_11600,KSG66\_11605,KSG66\_11610,KSG66\_11620,KSG66\_11625,KSG66\_11630,KSG66\_11650,KSG66\_11700,KSG66\_11720,KSG66\_11780,KSG66\_11810,KSG66\_11815,KSG66\_11820,KSG66\_11825,KSG66\_11830,KSG66\_11845,KSG66\_11865,KSG66\_11910,KSG66\_11925,KSG66\_11960,KSG66\_11980,KSG66\_12000,KSG66\_12140,KSG66\_12160,KSG66\_12170,KSG66\_12245,KSG66\_12310,KSG66\_12315,KSG66\_12345,KSG66\_12455,KSG66\_12475,KSG66\_12480,KSG66\_12500,KSG66\_12510,KSG66\_12550,KSG66\_12560,KSG66\_12565,KSG66\_12570,KSG66\_12575,KSG66\_12635,KSG66\_12640,KSG66\_12655,KSG66\_12665,KSG66\_12680,KSG66\_12755,KSG66\_12760,KSG66\_12765,KSG66\_12780,KSG66\_12785,KSG66\_12795,KSG66\_12825,KSG66\_12830,KSG66\_12835,KSG66\_12870,KSG66\_12875,KSG66\_12920,KSG66\_12930,KSG66\_12950,KSG66\_12960,KSG66\_12965,KSG66\_12970,KSG66\_12975,KSG66\_12980,KSG66\_12990,KSG66\_13020,KSG66\_13035,KSG66\_13055,KSG66\_13060,KSG66\_13065,KSG66\_13075,KSG66\_13080,KSG66\_13085,KSG66\_13095,KSG66\_13100,KSG66\_13105,KSG66\_13110,KSG66\_13115,KSG66\_13130,KSG66\_13140,KSG66\_13145,KSG66\_13155,KSG66\_13160,KSG66\_13180,KSG66\_13185,KSG66\_13195,KSG66\_13200,KSG66\_13205,KSG66\_13215,KSG66\_13225,KSG66\_13230,KSG66\_13235,KSG66\_13275,KSG66\_13280,KSG66\_13285,KSG66\_13290,KSG66\_13300,KSG66\_13335,KSG66\_13345,KSG66\_13350,KSG66\_13365,KSG66\_13375,KSG66\_13385,KSG66\_13390,KSG66\_13435,KSG66\_13440,KSG66\_13445,KSG66\_13450,KSG66\_13455,KSG66\_13500,KSG66\_13505,KSG66\_13510,KSG66\_13530,KSG66\_13555,KSG66\_13575,KSG66\_13585,KSG66\_13590,KSG66\_13720,KSG66\_13730,KSG66\_13735,KSG66\_13740,KSG66\_13760,KSG66\_13780,KSG66\_13800,KSG66\_13820,KSG66\_13845,KSG66\_13860,KSG66\_13870,KSG66\_13895,KSG66\_13905,KSG66\_13935,KSG66\_13950,KSG66\_13960,KSG66\_13965,KSG66\_13985,KSG66\_14070,KSG66\_14075,KSG66\_14085,KSG66\_14125,KSG66\_14165,KSG66\_14180,KSG66\_14185,KSG66\_14190,KSG66\_14200,KSG66\_14340,KSG66\_14370,KSG66\_14385,KSG66\_14395,KSG66\_14415,KSG66\_14425,KSG66\_14480,KSG66\_14515,KSG66\_14530,KSG66\_14535,KSG66\_14570,KSG66\_14650,KSG66\_14670,KSG66\_14675,KSG66\_14765,KSG66\_14805,KSG66\_14830,KSG66\_14840,KSG66\_14850,KSG66\_14855,KSG66\_14890,KSG66\_14895,KSG66\_14905,KSG66\_14955,KSG66\_14960,KSG66\_14990,KSG66\_14995,KSG66\_15030,KSG66\_15065,KSG66\_15070,KSG66\_15095,KSG66\_15110,KSG66\_15210,KSG66\_15230,KSG66\_15235,KSG66\_15240,KSG66\_15245,KSG66\_15270,KSG66\_15275,KSG66\_15280,KSG66\_15290,KSG66\_15300,KSG66\_15320,KSG66\_15365,KSG66\_15380,KSG66\_15385,KSG66\_15415,KSG66\_15430,KSG66\_15440,KSG66\_15445,KSG66\_15470,KSG66\_15495,KSG66\_15540,KSG66\_15545,KSG66\_15550,KSG66\_15555,KSG66\_15585,KSG66\_15590,KSG66\_15595,KSG66\_15620,KSG66\_15630,KSG66\_15635,KSG66\_15640,KSG66\_15660,KSG66\_15665,KSG66\_15680,KSG66\_15695,KSG66\_15700,KSG66\_15705,KSG66\_15710,KSG66\_15715,KSG66\_15720,KSG66\_15725,KSG66\_15730,KSG66\_15735,KSG66\_15750,KSG66\_15825,KSG66\_15830,KSG66\_15850,KSG66\_15855,KSG66\_15860,KSG66\_15865,KSG66\_15875,KSG66\_15880,KSG66\_15885,KSG66\_15890,KSG66\_15970,KSG66\_15975,KSG66\_15980,KSG66\_15985,KSG66\_15990,KSG66\_16000,KSG66\_16010,KSG66\_16030,KSG66\_16080,KSG66\_16085,KSG66\_16090,KSG66\_16305,KSG66\_16310,KSG66\_16315,KSG66\_16340,KSG66\_16350,KSG66\_16390,KSG66\_16405,KSG66\_16415,KSG66\_16420,KSG66\_16425,KSG66\_16430,KSG66\_16435,KSG66\_16440,KSG66\_16445,KSG66\_16450,KSG66\_16455,KSG66\_16480,KSG66\_16485,KSG66\_16490,KSG66\_16535,KSG66\_16540,KSG66\_16560,KSG66\_16575,KSG66\_16595,KSG66\_16600,KSG66\_16605,KSG66\_16615,KSG66\_16635,KSG66\_16640,KSG66\_16645,KSG66\_16660,KSG66\_16670,KSG66\_16675,KSG66\_16680,KSG66\_16685,KSG66\_16720,KSG66\_16745,KSG66\_16750,KSG66\_16755,KSG66\_16765,KSG66\_16770,KSG66\_16775,KSG66\_16805,KSG66\_16810,KSG66\_16905,KSG66\_16940,KSG66\_16945,KSG66\_16950,KSG66\_16955,KSG66\_16960,KSG66\_16970,KSG66\_16975,KSG66\_16985,KSG66\_16990,KSG66\_17015,KSG66\_17035,KSG66\_17040,KSG66\_17050,KSG66\_17115,KSG66\_17160,KSG66\_17165,KSG66\_17180,KSG66\_17185,KSG66\_17200,KSG66\_17205,KSG66\_17210,KSG66\_17220,KSG66\_17235,KSG66\_17265,KSG66\_17290,KSG66\_17295,KSG66\_17300,KSG66\_17325,KSG66\_17350,KSG66\_17370,KSG66\_17375,KSG66\_17380,KSG66\_17385,KSG66\_17390,KSG66\_17395,KSG66\_17400,KSG66\_17405,KSG66\_17410,KSG66\_17415,KSG66\_17420,KSG66\_17430,KSG66\_17440,KSG66\_17475,KSG66\_17485,KSG66\_17490,KSG66\_17495,KSG66\_17500,KSG66\_17515,KSG66\_17520,KSG66\_17525,KSG66\_17530,KSG66\_17535,KSG66\_17545,KSG66\_17560,KSG66\_17565,KSG66\_17575,KSG66\_17580,KSG66\_17590,KSG66\_17595,KSG66\_17600,KSG66\_17630,KSG66\_17640,KSG66\_17665,KSG66\_17770,KSG66\_17790,KSG66\_17795,KSG66\_17805,KSG66\_17815,KSG66\_17825,KSG66\_17830,KSG66\_17840,KSG66\_17850,KSG66\_17860,KSG66\_17885,KSG66\_17915,KSG66\_17960,KSG66\_17985,KSG66\_18020,KSG66\_18030,KSG66\_18045,KSG66\_18050,KSG66\_18055,KSG66\_18060,KSG66\_18075,KSG66\_18105,KSG66\_18110,KSG66\_18120,KSG66\_18125,KSG66\_18135,KSG66\_18170,KSG66\_18185,KSG66\_18190,KSG66\_18200,KSG66\_18205,KSG66\_18210,KSG66\_18225,KSG66\_18230,KSG66\_18235,KSG66\_18240,KSG66\_18245,KSG66\_18250,KSG66\_18255,KSG66\_18275,KSG66\_18280,KSG66\_18285,KSG66\_18290,KSG66\_18315,KSG66\_18325,KSG66\_18370,KSG66\_18375,KSG66\_18380,KSG66\_18385,KSG66\_18390,KSG66\_18400,KSG66\_18420,KSG66\_18470,KSG66\_18575,KSG66\_18580,KSG66\_18585,KSG66\_18590,KSG66\_18595,KSG66\_18600,KSG66\_18610,KSG66\_18670,KSG66\_18705,KSG66\_18720,KSG66\_18730,KSG66\_18735,KSG66\_18740,KSG66\_18745,KSG66\_18750,KSG66\_18760,KSG66\_18765,KSG66\_18785,KSG66\_18790,KSG66\_18835,KSG66\_18840,KSG66\_18855,KSG66\_18955,KSG66\_18960,KSG66\_18965,KSG66\_18980,KSG66\_19045,KSG66\_19070,KSG66\_19175,KSG66\_19345,KSG66\_19380,KSG66\_19395,KSG66\_19405 |
| GO:0008150 | Biological Process | GO:0051179 | localization | 328 | KSG66\_00650,KSG66\_00830,KSG66\_00875,KSG66\_00880,KSG66\_01020,KSG66\_01035,KSG66\_01040,KSG66\_01045,KSG66\_01205,KSG66\_01290,KSG66\_01375,KSG66\_01385,KSG66\_01395,KSG66\_01475,KSG66\_01495,KSG66\_01620,KSG66\_01625,KSG66\_01665,KSG66\_01875,KSG66\_01935,KSG66\_01950,KSG66\_01955,KSG66\_01960,KSG66\_01975,KSG66\_02015,KSG66\_02020,KSG66\_02025,KSG66\_02030,KSG66\_02035,KSG66\_02075,KSG66\_02105,KSG66\_02110,KSG66\_02190,KSG66\_02265,KSG66\_02320,KSG66\_02370,KSG66\_02385,KSG66\_02395,KSG66\_02425,KSG66\_02615,KSG66\_02755,KSG66\_02760,KSG66\_02835,KSG66\_02845,KSG66\_02920,KSG66\_03025,KSG66\_03030,KSG66\_03040,KSG66\_03060,KSG66\_03140,KSG66\_03320,KSG66\_03460,KSG66\_03485,KSG66\_03675,KSG66\_03705,KSG66\_03715,KSG66\_03755,KSG66\_03790,KSG66\_03835,KSG66\_03870,KSG66\_03950,KSG66\_04020,KSG66\_04090,KSG66\_04105,KSG66\_04155,KSG66\_04205,KSG66\_04290,KSG66\_04455,KSG66\_04460,KSG66\_04480,KSG66\_04485,KSG66\_04490,KSG66\_04575,KSG66\_04615,KSG66\_04685,KSG66\_04780,KSG66\_04830,KSG66\_04835,KSG66\_04885,KSG66\_04890,KSG66\_04950,KSG66\_04955,KSG66\_05005,KSG66\_05065,KSG66\_05070,KSG66\_05140,KSG66\_05205,KSG66\_05210,KSG66\_05215,KSG66\_05260,KSG66\_05300,KSG66\_05405,KSG66\_05425,KSG66\_05530,KSG66\_05620,KSG66\_05630,KSG66\_05640,KSG66\_05655,KSG66\_05660,KSG66\_05665,KSG66\_05675,KSG66\_05730,KSG66\_05770,KSG66\_06005,KSG66\_06035,KSG66\_06095,KSG66\_06125,KSG66\_06480,KSG66\_06490,KSG66\_06495,KSG66\_06500,KSG66\_06505,KSG66\_06550,KSG66\_06555,KSG66\_06655,KSG66\_06730,KSG66\_06815,KSG66\_06820,KSG66\_06910,KSG66\_06930,KSG66\_06935,KSG66\_06940,KSG66\_07145,KSG66\_07150,KSG66\_07165,KSG66\_07180,KSG66\_07220,KSG66\_07435,KSG66\_07455,KSG66\_07465,KSG66\_07470,KSG66\_07735,KSG66\_07785,KSG66\_07820,KSG66\_07970,KSG66\_07985,KSG66\_08085,KSG66\_08090,KSG66\_08095,KSG66\_08100,KSG66\_08105,KSG66\_08115,KSG66\_08120,KSG66\_08140,KSG66\_08150,KSG66\_08155,KSG66\_08160,KSG66\_08175,KSG66\_08180,KSG66\_08190,KSG66\_08200,KSG66\_08640,KSG66\_08645,KSG66\_08765,KSG66\_08960,KSG66\_09055,KSG66\_09090,KSG66\_09230,KSG66\_09355,KSG66\_09425,KSG66\_09475,KSG66\_09615,KSG66\_09620,KSG66\_09670,KSG66\_09745,KSG66\_10285,KSG66\_10355,KSG66\_10770,KSG66\_11015,KSG66\_11310,KSG66\_11315,KSG66\_11320,KSG66\_11700,KSG66\_11710,KSG66\_11780,KSG66\_11810,KSG66\_11815,KSG66\_11820,KSG66\_11825,KSG66\_11830,KSG66\_12325,KSG66\_12345,KSG66\_12445,KSG66\_12560,KSG66\_12565,KSG66\_12570,KSG66\_12575,KSG66\_12680,KSG66\_12685,KSG66\_12950,KSG66\_13200,KSG66\_13205,KSG66\_13345,KSG66\_13375,KSG66\_13585,KSG66\_13750,KSG66\_13870,KSG66\_13880,KSG66\_13905,KSG66\_13920,KSG66\_13925,KSG66\_13930,KSG66\_13965,KSG66\_13975,KSG66\_13980,KSG66\_14000,KSG66\_14020,KSG66\_14070,KSG66\_14105,KSG66\_14110,KSG66\_14340,KSG66\_14385,KSG66\_14395,KSG66\_14625,KSG66\_14630,KSG66\_14635,KSG66\_14650,KSG66\_14670,KSG66\_14675,KSG66\_14855,KSG66\_14860,KSG66\_14960,KSG66\_15110,KSG66\_15220,KSG66\_15235,KSG66\_15240,KSG66\_15245,KSG66\_15320,KSG66\_15365,KSG66\_15430,KSG66\_15470,KSG66\_15485,KSG66\_15540,KSG66\_15545,KSG66\_15550,KSG66\_15555,KSG66\_15585,KSG66\_15590,KSG66\_15630,KSG66\_15680,KSG66\_15715,KSG66\_15720,KSG66\_15725,KSG66\_15730,KSG66\_15735,KSG66\_15800,KSG66\_15850,KSG66\_15855,KSG66\_15860,KSG66\_15865,KSG66\_15875,KSG66\_15880,KSG66\_15885,KSG66\_15890,KSG66\_16000,KSG66\_16030,KSG66\_16305,KSG66\_16310,KSG66\_16315,KSG66\_16340,KSG66\_16415,KSG66\_16560,KSG66\_16600,KSG66\_16620,KSG66\_16670,KSG66\_16675,KSG66\_16810,KSG66\_16815,KSG66\_16950,KSG66\_16955,KSG66\_16960,KSG66\_16990,KSG66\_17035,KSG66\_17040,KSG66\_17160,KSG66\_17180,KSG66\_17200,KSG66\_17205,KSG66\_17210,KSG66\_17220,KSG66\_17370,KSG66\_17375,KSG66\_17380,KSG66\_17385,KSG66\_17390,KSG66\_17395,KSG66\_17400,KSG66\_17405,KSG66\_17410,KSG66\_17440,KSG66\_17490,KSG66\_17575,KSG66\_17805,KSG66\_17830,KSG66\_17835,KSG66\_17840,KSG66\_18045,KSG66\_18050,KSG66\_18055,KSG66\_18060,KSG66\_18080,KSG66\_18110,KSG66\_18170,KSG66\_18205,KSG66\_18230,KSG66\_18235,KSG66\_18240,KSG66\_18245,KSG66\_18255,KSG66\_18275,KSG66\_18280,KSG66\_18285,KSG66\_18290,KSG66\_18325,KSG66\_18370,KSG66\_18375,KSG66\_18390,KSG66\_18400,KSG66\_18450,KSG66\_18470,KSG66\_18485,KSG66\_18500,KSG66\_18595,KSG66\_18765,KSG66\_18960,KSG66\_19195,KSG66\_19255,KSG66\_19405 |
| GO:0008150 | Biological Process | GO:0065007 | biological regulation | 325 | KSG66\_00005,KSG66\_00075,KSG66\_00250,KSG66\_00300,KSG66\_00315,KSG66\_00340,KSG66\_00345,KSG66\_00475,KSG66\_00565,KSG66\_00580,KSG66\_00590,KSG66\_00640,KSG66\_00655,KSG66\_00665,KSG66\_00925,KSG66\_00935,KSG66\_01040,KSG66\_01045,KSG66\_01115,KSG66\_01120,KSG66\_01200,KSG66\_01410,KSG66\_01420,KSG66\_01500,KSG66\_01860,KSG66\_01985,KSG66\_01990,KSG66\_02055,KSG66\_02060,KSG66\_02095,KSG66\_02180,KSG66\_02190,KSG66\_02230,KSG66\_02375,KSG66\_02380,KSG66\_02430,KSG66\_02435,KSG66\_02440,KSG66\_02510,KSG66\_02515,KSG66\_02520,KSG66\_02665,KSG66\_02840,KSG66\_02850,KSG66\_02880,KSG66\_02885,KSG66\_02945,KSG66\_02970,KSG66\_03010,KSG66\_03035,KSG66\_03135,KSG66\_03195,KSG66\_03250,KSG66\_03420,KSG66\_03440,KSG66\_03465,KSG66\_03550,KSG66\_03625,KSG66\_03690,KSG66\_03710,KSG66\_03775,KSG66\_03780,KSG66\_03880,KSG66\_04000,KSG66\_04005,KSG66\_04065,KSG66\_04070,KSG66\_04085,KSG66\_04125,KSG66\_04160,KSG66\_04195,KSG66\_04310,KSG66\_04510,KSG66\_04565,KSG66\_04600,KSG66\_04680,KSG66\_04705,KSG66\_04710,KSG66\_04735,KSG66\_04785,KSG66\_04790,KSG66\_04795,KSG66\_04810,KSG66\_04870,KSG66\_05035,KSG66\_05115,KSG66\_05180,KSG66\_05210,KSG66\_05230,KSG66\_05410,KSG66\_05490,KSG66\_05595,KSG66\_05690,KSG66\_05705,KSG66\_05975,KSG66\_06110,KSG66\_06130,KSG66\_06250,KSG66\_06280,KSG66\_06580,KSG66\_06660,KSG66\_06675,KSG66\_06705,KSG66\_06750,KSG66\_06800,KSG66\_06805,KSG66\_06810,KSG66\_06865,KSG66\_06905,KSG66\_06920,KSG66\_06925,KSG66\_06935,KSG66\_06945,KSG66\_06970,KSG66\_06975,KSG66\_06990,KSG66\_07005,KSG66\_07095,KSG66\_07155,KSG66\_07205,KSG66\_07210,KSG66\_07320,KSG66\_07435,KSG66\_07600,KSG66\_07605,KSG66\_07610,KSG66\_07615,KSG66\_07620,KSG66\_07625,KSG66\_07655,KSG66\_07660,KSG66\_07710,KSG66\_07730,KSG66\_07865,KSG66\_07880,KSG66\_07935,KSG66\_07980,KSG66\_08080,KSG66\_08160,KSG66\_08210,KSG66\_08220,KSG66\_08235,KSG66\_08285,KSG66\_08300,KSG66\_08410,KSG66\_08665,KSG66\_08720,KSG66\_08775,KSG66\_08915,KSG66\_08920,KSG66\_08960,KSG66\_09315,KSG66\_09360,KSG66\_09370,KSG66\_09500,KSG66\_09720,KSG66\_09760,KSG66\_10080,KSG66\_10115,KSG66\_10185,KSG66\_10190,KSG66\_10325,KSG66\_10400,KSG66\_10460,KSG66\_10630,KSG66\_10715,KSG66\_10730,KSG66\_10790,KSG66\_10810,KSG66\_10830,KSG66\_10950,KSG66\_10955,KSG66\_10960,KSG66\_10965,KSG66\_10995,KSG66\_11030,KSG66\_11460,KSG66\_11475,KSG66\_11480,KSG66\_11510,KSG66\_11605,KSG66\_11650,KSG66\_11735,KSG66\_11880,KSG66\_11930,KSG66\_11950,KSG66\_11975,KSG66\_12070,KSG66\_12075,KSG66\_12085,KSG66\_12150,KSG66\_12215,KSG66\_12275,KSG66\_12315,KSG66\_12320,KSG66\_12405,KSG66\_12425,KSG66\_12505,KSG66\_12795,KSG66\_12830,KSG66\_12835,KSG66\_12875,KSG66\_12930,KSG66\_13035,KSG66\_13040,KSG66\_13085,KSG66\_13110,KSG66\_13130,KSG66\_13285,KSG66\_13290,KSG66\_13325,KSG66\_13340,KSG66\_13375,KSG66\_13490,KSG66\_13530,KSG66\_13590,KSG66\_13600,KSG66\_13730,KSG66\_13755,KSG66\_13780,KSG66\_13800,KSG66\_13885,KSG66\_13915,KSG66\_13950,KSG66\_13960,KSG66\_13985,KSG66\_13990,KSG66\_14025,KSG66\_14140,KSG66\_14415,KSG66\_14435,KSG66\_14440,KSG66\_14445,KSG66\_14555,KSG66\_14685,KSG66\_15070,KSG66\_15315,KSG66\_15335,KSG66\_15365,KSG66\_15385,KSG66\_15395,KSG66\_15410,KSG66\_15440,KSG66\_15445,KSG66\_15515,KSG66\_15540,KSG66\_15545,KSG66\_15550,KSG66\_15555,KSG66\_15595,KSG66\_15685,KSG66\_15705,KSG66\_15745,KSG66\_15805,KSG66\_15815,KSG66\_15825,KSG66\_15830,KSG66\_15995,KSG66\_16005,KSG66\_16020,KSG66\_16110,KSG66\_16180,KSG66\_16195,KSG66\_16370,KSG66\_16405,KSG66\_16480,KSG66\_16495,KSG66\_16580,KSG66\_16585,KSG66\_16595,KSG66\_16600,KSG66\_16655,KSG66\_16685,KSG66\_16715,KSG66\_16720,KSG66\_16730,KSG66\_16795,KSG66\_16935,KSG66\_16985,KSG66\_17030,KSG66\_17055,KSG66\_17140,KSG66\_17190,KSG66\_17195,KSG66\_17225,KSG66\_17235,KSG66\_17285,KSG66\_17350,KSG66\_17485,KSG66\_17510,KSG66\_17520,KSG66\_17535,KSG66\_17550,KSG66\_17610,KSG66\_17620,KSG66\_17700,KSG66\_17725,KSG66\_17775,KSG66\_17860,KSG66\_17865,KSG66\_18005,KSG66\_18110,KSG66\_18130,KSG66\_18210,KSG66\_18245,KSG66\_18570,KSG66\_18615,KSG66\_18670,KSG66\_18675,KSG66\_18755,KSG66\_18780,KSG66\_18785,KSG66\_18795,KSG66\_18815,KSG66\_18840,KSG66\_18975,KSG66\_18980,KSG66\_19045,KSG66\_19160,KSG66\_19210,KSG66\_19220,KSG66\_19250,KSG66\_19305,KSG66\_19320,KSG66\_19395 |
| GO:0008150 | Biological Process | GO:0050789 | regulation of biological process | 303 | KSG66\_00005,KSG66\_00075,KSG66\_00250,KSG66\_00300,KSG66\_00315,KSG66\_00340,KSG66\_00345,KSG66\_00475,KSG66\_00565,KSG66\_00580,KSG66\_00590,KSG66\_00640,KSG66\_00655,KSG66\_00665,KSG66\_00925,KSG66\_00935,KSG66\_01115,KSG66\_01120,KSG66\_01200,KSG66\_01410,KSG66\_01420,KSG66\_01500,KSG66\_01860,KSG66\_01985,KSG66\_01990,KSG66\_02055,KSG66\_02060,KSG66\_02095,KSG66\_02180,KSG66\_02190,KSG66\_02230,KSG66\_02375,KSG66\_02380,KSG66\_02435,KSG66\_02440,KSG66\_02510,KSG66\_02515,KSG66\_02520,KSG66\_02665,KSG66\_02840,KSG66\_02850,KSG66\_02880,KSG66\_02885,KSG66\_02945,KSG66\_02970,KSG66\_03010,KSG66\_03035,KSG66\_03135,KSG66\_03195,KSG66\_03250,KSG66\_03420,KSG66\_03440,KSG66\_03465,KSG66\_03550,KSG66\_03625,KSG66\_03690,KSG66\_03710,KSG66\_03775,KSG66\_03780,KSG66\_03880,KSG66\_04000,KSG66\_04005,KSG66\_04070,KSG66\_04085,KSG66\_04125,KSG66\_04160,KSG66\_04195,KSG66\_04310,KSG66\_04510,KSG66\_04565,KSG66\_04600,KSG66\_04680,KSG66\_04705,KSG66\_04710,KSG66\_04735,KSG66\_04785,KSG66\_04790,KSG66\_04795,KSG66\_04810,KSG66\_04870,KSG66\_05035,KSG66\_05115,KSG66\_05180,KSG66\_05210,KSG66\_05230,KSG66\_05410,KSG66\_05490,KSG66\_05595,KSG66\_05690,KSG66\_05705,KSG66\_05975,KSG66\_06110,KSG66\_06130,KSG66\_06250,KSG66\_06280,KSG66\_06580,KSG66\_06660,KSG66\_06675,KSG66\_06705,KSG66\_06750,KSG66\_06800,KSG66\_06805,KSG66\_06810,KSG66\_06865,KSG66\_06920,KSG66\_06925,KSG66\_06935,KSG66\_06945,KSG66\_06970,KSG66\_06975,KSG66\_06990,KSG66\_07005,KSG66\_07095,KSG66\_07155,KSG66\_07205,KSG66\_07210,KSG66\_07435,KSG66\_07600,KSG66\_07605,KSG66\_07610,KSG66\_07615,KSG66\_07620,KSG66\_07625,KSG66\_07655,KSG66\_07660,KSG66\_07710,KSG66\_07730,KSG66\_07865,KSG66\_07880,KSG66\_07935,KSG66\_07980,KSG66\_08080,KSG66\_08160,KSG66\_08210,KSG66\_08220,KSG66\_08235,KSG66\_08285,KSG66\_08300,KSG66\_08410,KSG66\_08665,KSG66\_08720,KSG66\_08775,KSG66\_08915,KSG66\_08920,KSG66\_08960,KSG66\_09315,KSG66\_09360,KSG66\_09370,KSG66\_09500,KSG66\_09720,KSG66\_09760,KSG66\_10080,KSG66\_10185,KSG66\_10190,KSG66\_10325,KSG66\_10400,KSG66\_10460,KSG66\_10630,KSG66\_10715,KSG66\_10730,KSG66\_10790,KSG66\_10830,KSG66\_10950,KSG66\_10955,KSG66\_10960,KSG66\_10965,KSG66\_10995,KSG66\_11030,KSG66\_11460,KSG66\_11475,KSG66\_11510,KSG66\_11605,KSG66\_11650,KSG66\_11735,KSG66\_11880,KSG66\_11930,KSG66\_11950,KSG66\_11975,KSG66\_12070,KSG66\_12075,KSG66\_12085,KSG66\_12150,KSG66\_12215,KSG66\_12275,KSG66\_12315,KSG66\_12320,KSG66\_12405,KSG66\_12425,KSG66\_12505,KSG66\_12795,KSG66\_12830,KSG66\_12835,KSG66\_12875,KSG66\_12930,KSG66\_13035,KSG66\_13040,KSG66\_13110,KSG66\_13130,KSG66\_13285,KSG66\_13290,KSG66\_13325,KSG66\_13340,KSG66\_13375,KSG66\_13490,KSG66\_13590,KSG66\_13600,KSG66\_13730,KSG66\_13755,KSG66\_13780,KSG66\_13885,KSG66\_13915,KSG66\_13950,KSG66\_13960,KSG66\_13985,KSG66\_13990,KSG66\_14025,KSG66\_14415,KSG66\_14435,KSG66\_14440,KSG66\_14445,KSG66\_14555,KSG66\_14685,KSG66\_15070,KSG66\_15315,KSG66\_15335,KSG66\_15395,KSG66\_15410,KSG66\_15440,KSG66\_15445,KSG66\_15515,KSG66\_15595,KSG66\_15685,KSG66\_15745,KSG66\_15805,KSG66\_15815,KSG66\_15825,KSG66\_15830,KSG66\_15995,KSG66\_16005,KSG66\_16020,KSG66\_16110,KSG66\_16180,KSG66\_16195,KSG66\_16370,KSG66\_16405,KSG66\_16480,KSG66\_16495,KSG66\_16580,KSG66\_16585,KSG66\_16595,KSG66\_16600,KSG66\_16655,KSG66\_16685,KSG66\_16715,KSG66\_16720,KSG66\_16730,KSG66\_16795,KSG66\_16935,KSG66\_16985,KSG66\_17030,KSG66\_17055,KSG66\_17140,KSG66\_17190,KSG66\_17195,KSG66\_17225,KSG66\_17235,KSG66\_17285,KSG66\_17350,KSG66\_17485,KSG66\_17510,KSG66\_17520,KSG66\_17535,KSG66\_17550,KSG66\_17610,KSG66\_17620,KSG66\_17700,KSG66\_17725,KSG66\_17775,KSG66\_17860,KSG66\_17865,KSG66\_18005,KSG66\_18130,KSG66\_18210,KSG66\_18245,KSG66\_18570,KSG66\_18615,KSG66\_18670,KSG66\_18675,KSG66\_18755,KSG66\_18780,KSG66\_18785,KSG66\_18795,KSG66\_18815,KSG66\_18975,KSG66\_18980,KSG66\_19045,KSG66\_19160,KSG66\_19210,KSG66\_19220,KSG66\_19250,KSG66\_19305,KSG66\_19320,KSG66\_19395 |
| GO:0008150 | Biological Process | GO:0032502 | developmental process | 182 | KSG66\_00075,KSG66\_00225,KSG66\_00250,KSG66\_00280,KSG66\_00285,KSG66\_00290,KSG66\_00310,KSG66\_00315,KSG66\_00345,KSG66\_00365,KSG66\_00370,KSG66\_00395,KSG66\_00420,KSG66\_00640,KSG66\_00935,KSG66\_01685,KSG66\_01815,KSG66\_01850,KSG66\_01925,KSG66\_01930,KSG66\_01935,KSG66\_02075,KSG66\_02080,KSG66\_02165,KSG66\_02180,KSG66\_02435,KSG66\_02440,KSG66\_02725,KSG66\_02750,KSG66\_02990,KSG66\_03275,KSG66\_03980,KSG66\_04075,KSG66\_04220,KSG66\_04900,KSG66\_04915,KSG66\_05035,KSG66\_05065,KSG66\_05070,KSG66\_05180,KSG66\_05370,KSG66\_05375,KSG66\_05620,KSG66\_05630,KSG66\_05640,KSG66\_05655,KSG66\_05660,KSG66\_05665,KSG66\_05675,KSG66\_05705,KSG66\_05820,KSG66\_05825,KSG66\_05835,KSG66\_05840,KSG66\_05845,KSG66\_06220,KSG66\_06230,KSG66\_06425,KSG66\_06430,KSG66\_06435,KSG66\_06490,KSG66\_06495,KSG66\_06500,KSG66\_06505,KSG66\_06685,KSG66\_06715,KSG66\_06750,KSG66\_06800,KSG66\_06805,KSG66\_06900,KSG66\_06905,KSG66\_06950,KSG66\_06970,KSG66\_06975,KSG66\_07005,KSG66\_07210,KSG66\_07435,KSG66\_07445,KSG66\_07505,KSG66\_07525,KSG66\_07600,KSG66\_07605,KSG66\_07610,KSG66\_07615,KSG66\_07620,KSG66\_07625,KSG66\_07630,KSG66\_07650,KSG66\_07655,KSG66\_07660,KSG66\_07705,KSG66\_07875,KSG66\_07880,KSG66\_08235,KSG66\_08370,KSG66\_08475,KSG66\_08490,KSG66\_08515,KSG66\_08605,KSG66\_08950,KSG66\_08955,KSG66\_08980,KSG66\_08985,KSG66\_09005,KSG66\_09010,KSG66\_09315,KSG66\_10325,KSG66\_10400,KSG66\_10615,KSG66\_10625,KSG66\_10645,KSG66\_10705,KSG66\_10710,KSG66\_10730,KSG66\_10825,KSG66\_10830,KSG66\_10930,KSG66\_10935,KSG66\_10950,KSG66\_10955,KSG66\_10960,KSG66\_10965,KSG66\_10995,KSG66\_11390,KSG66\_11405,KSG66\_11460,KSG66\_11540,KSG66\_11550,KSG66\_11555,KSG66\_11565,KSG66\_11650,KSG66\_11655,KSG66\_12095,KSG66\_12100,KSG66\_12215,KSG66\_12500,KSG66\_12695,KSG66\_12795,KSG66\_12815,KSG66\_12820,KSG66\_12830,KSG66\_12835,KSG66\_13035,KSG66\_13170,KSG66\_13465,KSG66\_13570,KSG66\_13730,KSG66\_13780,KSG66\_14165,KSG66\_14415,KSG66\_14455,KSG66\_14585,KSG66\_14805,KSG66\_15005,KSG66\_15040,KSG66\_15430,KSG66\_15435,KSG66\_15970,KSG66\_16215,KSG66\_16370,KSG66\_16585,KSG66\_16595,KSG66\_16600,KSG66\_16865,KSG66\_17185,KSG66\_17190,KSG66\_17235,KSG66\_17245,KSG66\_17265,KSG66\_17315,KSG66\_17345,KSG66\_17350,KSG66\_17455,KSG66\_17485,KSG66\_17520,KSG66\_17530,KSG66\_17535,KSG66\_17560,KSG66\_17725,KSG66\_18040,KSG66\_18210,KSG66\_19375 |
| GO:0008150 | Biological Process | GO:0050896 | response to stimulus | 171 | KSG66\_00020,KSG66\_00145,KSG66\_00305,KSG66\_00325,KSG66\_00340,KSG66\_00460,KSG66\_00565,KSG66\_00585,KSG66\_00590,KSG66\_00735,KSG66\_00815,KSG66\_01410,KSG66\_01495,KSG66\_01580,KSG66\_01690,KSG66\_01820,KSG66\_01825,KSG66\_01990,KSG66\_02050,KSG66\_02180,KSG66\_02375,KSG66\_02380,KSG66\_02445,KSG66\_02525,KSG66\_02795,KSG66\_02880,KSG66\_03045,KSG66\_03135,KSG66\_03435,KSG66\_03440,KSG66\_03485,KSG66\_03735,KSG66\_03775,KSG66\_03780,KSG66\_04000,KSG66\_04005,KSG66\_04025,KSG66\_04065,KSG66\_04085,KSG66\_04110,KSG66\_04165,KSG66\_04260,KSG66\_04680,KSG66\_04705,KSG66\_04885,KSG66\_04890,KSG66\_05180,KSG66\_05210,KSG66\_05300,KSG66\_05320,KSG66\_05405,KSG66\_05520,KSG66\_05785,KSG66\_05960,KSG66\_06110,KSG66\_06455,KSG66\_06550,KSG66\_06555,KSG66\_06685,KSG66\_06750,KSG66\_06755,KSG66\_06805,KSG66\_06815,KSG66\_06820,KSG66\_06950,KSG66\_06970,KSG66\_06990,KSG66\_07210,KSG66\_07320,KSG66\_07880,KSG66\_07930,KSG66\_08105,KSG66\_08120,KSG66\_08150,KSG66\_08155,KSG66\_08160,KSG66\_08165,KSG66\_08210,KSG66\_08220,KSG66\_08225,KSG66\_08230,KSG66\_08290,KSG66\_08470,KSG66\_08520,KSG66\_08525,KSG66\_08915,KSG66\_08920,KSG66\_08960,KSG66\_09670,KSG66\_09725,KSG66\_10070,KSG66\_10075,KSG66\_10165,KSG66\_10395,KSG66\_10400,KSG66\_10410,KSG66\_10440,KSG66\_10755,KSG66\_10790,KSG66\_11090,KSG66\_11235,KSG66\_11265,KSG66\_11460,KSG66\_11895,KSG66\_11900,KSG66\_11965,KSG66\_12000,KSG66\_12060,KSG66\_12310,KSG66\_12315,KSG66\_12345,KSG66\_12540,KSG66\_12730,KSG66\_12735,KSG66\_12795,KSG66\_12930,KSG66\_13080,KSG66\_13130,KSG66\_13285,KSG66\_13290,KSG66\_13365,KSG66\_13370,KSG66\_13375,KSG66\_13445,KSG66\_13615,KSG66\_13730,KSG66\_13960,KSG66\_13975,KSG66\_13980,KSG66\_13985,KSG66\_14125,KSG66\_14395,KSG66\_14415,KSG66\_14435,KSG66\_14440,KSG66\_14445,KSG66\_14450,KSG66\_15385,KSG66\_15440,KSG66\_15445,KSG66\_15595,KSG66\_15825,KSG66\_15830,KSG66\_16405,KSG66\_16415,KSG66\_16480,KSG66\_16535,KSG66\_16540,KSG66\_16595,KSG66\_16600,KSG66\_16720,KSG66\_16990,KSG66\_17060,KSG66\_17235,KSG66\_17250,KSG66\_17535,KSG66\_17565,KSG66\_17805,KSG66\_17860,KSG66\_17960,KSG66\_18250,KSG66\_18275,KSG66\_18315,KSG66\_18670,KSG66\_18770,KSG66\_18785,KSG66\_18835,KSG66\_18840,KSG66\_19045,KSG66\_19330,KSG66\_19395 |
| GO:0008150 | Biological Process | GO:0071840 | cellular component organization or biogenesis | 140 | KSG66\_00075,KSG66\_00270,KSG66\_00275,KSG66\_00310,KSG66\_00315,KSG66\_00340,KSG66\_00625,KSG66\_00655,KSG66\_00670,KSG66\_00730,KSG66\_00920,KSG66\_01540,KSG66\_02180,KSG66\_02435,KSG66\_02440,KSG66\_02445,KSG66\_02795,KSG66\_02990,KSG66\_03435,KSG66\_03725,KSG66\_03980,KSG66\_04270,KSG66\_04680,KSG66\_04995,KSG66\_06230,KSG66\_06425,KSG66\_06515,KSG66\_06970,KSG66\_07005,KSG66\_07435,KSG66\_07580,KSG66\_07600,KSG66\_07605,KSG66\_07610,KSG66\_07615,KSG66\_07620,KSG66\_07625,KSG66\_07690,KSG66\_07865,KSG66\_07870,KSG66\_07930,KSG66\_07970,KSG66\_07985,KSG66\_08005,KSG66\_08020,KSG66\_08115,KSG66\_08120,KSG66\_08140,KSG66\_08170,KSG66\_08175,KSG66\_08180,KSG66\_08190,KSG66\_08195,KSG66\_08200,KSG66\_08205,KSG66\_08260,KSG66\_08295,KSG66\_08300,KSG66\_08700,KSG66\_08920,KSG66\_08955,KSG66\_09315,KSG66\_09370,KSG66\_10175,KSG66\_10240,KSG66\_10325,KSG66\_10400,KSG66\_10640,KSG66\_10645,KSG66\_10665,KSG66\_10710,KSG66\_10755,KSG66\_10805,KSG66\_10810,KSG66\_10830,KSG66\_10965,KSG66\_11460,KSG66\_11475,KSG66\_11650,KSG66\_11900,KSG66\_11975,KSG66\_11990,KSG66\_12150,KSG66\_12620,KSG66\_12650,KSG66\_12730,KSG66\_12735,KSG66\_12825,KSG66\_12830,KSG66\_12835,KSG66\_12930,KSG66\_13025,KSG66\_13035,KSG66\_13145,KSG66\_13260,KSG66\_13290,KSG66\_13590,KSG66\_13780,KSG66\_14390,KSG66\_14415,KSG66\_15645,KSG66\_16395,KSG66\_16595,KSG66\_16600,KSG66\_16615,KSG66\_16635,KSG66\_16640,KSG66\_16645,KSG66\_16660,KSG66\_16675,KSG66\_16680,KSG66\_16685,KSG66\_16735,KSG66\_16740,KSG66\_16745,KSG66\_16750,KSG66\_16755,KSG66\_16760,KSG66\_16765,KSG66\_16770,KSG66\_16775,KSG66\_16800,KSG66\_16830,KSG66\_16835,KSG66\_16840,KSG66\_17100,KSG66\_17185,KSG66\_17235,KSG66\_17350,KSG66\_17475,KSG66\_17485,KSG66\_17520,KSG66\_17595,KSG66\_18200,KSG66\_18205,KSG66\_18210,KSG66\_18915,KSG66\_19380,KSG66\_19385,KSG66\_19405 |
| GO:0008150 | Biological Process | GO:0023052 | signaling | 40 | KSG66\_00590,KSG66\_01410,KSG66\_01990,KSG66\_02375,KSG66\_02880,KSG66\_03775,KSG66\_04000,KSG66\_04005,KSG66\_04705,KSG66\_06750,KSG66\_06805,KSG66\_07210,KSG66\_08210,KSG66\_08960,KSG66\_10790,KSG66\_11460,KSG66\_12315,KSG66\_12795,KSG66\_12930,KSG66\_13285,KSG66\_13290,KSG66\_13375,KSG66\_13960,KSG66\_13985,KSG66\_15440,KSG66\_15445,KSG66\_15595,KSG66\_15825,KSG66\_15830,KSG66\_16405,KSG66\_16480,KSG66\_16595,KSG66\_16600,KSG66\_16720,KSG66\_17535,KSG66\_17860,KSG66\_18670,KSG66\_18785,KSG66\_19045,KSG66\_19395 |

  

Fig 6-4-1 GO功能分类图

  

##### 6.5 COG注释

COG，Cluster of Orthologous Groups of proteins，由NCBI创建并维护的蛋白数据库，根据细菌、藻类和真核生物完整基因组中编码蛋白的系统进化关系构建而成。COG数据库将功能分为26大类，以大写英文字母表示。  
使用DIAMOND与COG（Cluster of Orthologous Groups of proteins）数据库比对，获得基因对应的注释结果，并根据注释结果对蛋白进行功能分类。

Tab 6-5-1 样本 Bac COG 注释结果表(前10行)

| Query\_id | Subject\_id | Identity(%) | Align\_length | Mis\_match | Gap | Query\_start | Query\_end | Subject\_start | Subject\_end | E\_value | Score | Protein-or-Domain | COG-ID | Function-Description | Code | Functional-Categories; |
| --- | --- | --- | --- | --- | --- | --- | --- | --- | --- | --- | --- | --- | --- | --- | --- | --- |
| KSG66\_00005 | BS\_dnaA | 97.3 | 446 | 12 | 0 | 1 | 446 | 1 | 446 | 1.8e-247 | 854.0 | BS\_dnaA | COG0593 | ATPase involved in DNA replication initiation | L | Replication, recombination and repair ; |
| KSG66\_00005 | BH0001 | 79.5 | 449 | 88 | 2 | 1 | 446 | 1 | 448 | 9.3e-204 | 708.8 | BH0001 | COG0593 | ATPase involved in DNA replication initiation | L | Replication, recombination and repair ; |
| KSG66\_00005 | lin0001 | 73.9 | 449 | 110 | 3 | 1 | 446 | 1 | 445 | 7.9e-187 | 652.5 | lin0001 | COG0593 | ATPase involved in DNA replication initiation | L | Replication, recombination and repair ; |
| KSG66\_00005 | CAC0001 | 62.2 | 436 | 162 | 1 | 6 | 441 | 7 | 439 | 1.4e-151 | 535.4 | CAC0001 | COG0593 | ATPase involved in DNA replication initiation | L | Replication, recombination and repair ; |
| KSG66\_00005 | SA0001 | 60.4 | 447 | 171 | 2 | 6 | 446 | 5 | 451 | 1.3e-149 | 528.9 | SA0001 | COG0593 | ATPase involved in DNA replication initiation | L | Replication, recombination and repair ; |
| KSG66\_00005 | alr2009 | 46.4 | 448 | 229 | 4 | 6 | 446 | 8 | 451 | 2.6e-113 | 408.3 | alr2009 | COG0593 | ATPase involved in DNA replication initiation | L | Replication, recombination and repair ; |
| KSG66\_00005 | Cgl0001 | 55.5 | 353 | 155 | 1 | 96 | 446 | 167 | 519 | 7.1e-111 | 400.2 | Cgl0001 | COG0593 | ATPase involved in DNA replication initiation | L | Replication, recombination and repair ; |
| KSG66\_00005 | DR0002 | 47.7 | 447 | 216 | 5 | 15 | 446 | 1 | 444 | 1.5e-108 | 392.5 | DR0002 | COG0593 | ATPase involved in DNA replication initiation | L | Replication, recombination and repair ; |
| KSG66\_00005 | sll0848 | 47.9 | 445 | 207 | 4 | 6 | 441 | 6 | 434 | 1.9e-108 | 392.1 | sll0848 | COG0593 | ATPase involved in DNA replication initiation | L | Replication, recombination and repair ; |
| KSG66\_00005 | MT0001 | 58.6 | 331 | 136 | 1 | 111 | 441 | 168 | 497 | 3.4e-105 | 381.3 | MT0001 | COG0593 | ATPase involved in DNA replication initiation | L | Replication, recombination and repair ; |

  

Fig 6-5-1 COG 功能分类图

  

广州基迪奥生物科技有限公司


---

### 7 高级分析

除了基因注释较为通用的数据库，我们也提供了细分领域更加个性化的基因功能分析，以便于系统深入的功能分析。

##### 7.1 Pfam 注释

Pfam（Protein families database of alignments and hidden Markov models ）提供了完整准确的蛋白质家族和结构域分类信息，可广泛应用于蛋白家族查询和蛋白结构域的注释。  
使用Pfam Scan（https://www.ebi.ac.uk/Tools/pfa/pfamscan/）默认参数将目标序列与数据库比对。我们使用的数据库为2018.09更新的最新版Pfam 32.0。

- 样本Bac 蛋白结构相关注释汇总表： Bac.pfamA.xls

Tab 7-1-1 样本 Bac Pfam 注释结果表(前10行)

| seq id | alignment start | alignment end | envelope start | envelope end | hmm acc | hmm name | type | hmm start | hmm end | hmm length | bit score | E-value | significance | clan | PfamA\_definition |
| --- | --- | --- | --- | --- | --- | --- | --- | --- | --- | --- | --- | --- | --- | --- | --- |
| KSG66\_00005 | 7 | 65 | 6 | 68 | PF11638.8 | DnaA\_N | Domain | 3 | 61 | 64 | 65.3 | 3.1e-18 | 1 | CL0494 | DnaA N-terminal domain |
| KSG66\_00005 | 111 | 327 | 111 | 329 | PF00308.18 | Bac\_DnaA | Domain | 1 | 217 | 219 | 358.4 | 1.5e-107 | 1 | CL0023 | Bacterial dnaA protein |
| KSG66\_00005 | 356 | 424 | 356 | 424 | PF08299.11 | Bac\_DnaA\_C | Domain | 1 | 69 | 69 | 106.0 | 7.5e-31 | 1 | CL0123 | Bacterial dnaA protein helix-turn-helix |
| KSG66\_00010 | 1 | 127 | 1 | 127 | PF00712.19 | DNA\_pol3\_beta | Domain | 1 | 121 | 121 | 142.4 | 7e-42 | 1 | CL0060 | DNA polymerase III beta subunit, N-terminal domain |
| KSG66\_00010 | 136 | 251 | 136 | 251 | PF02767.16 | DNA\_pol3\_beta\_2 | Domain | 1 | 116 | 116 | 139.2 | 6.7e-41 | 1 | CL0060 | DNA polymerase III beta subunit, central domain |
| KSG66\_00010 | 254 | 376 | 253 | 376 | PF02768.15 | DNA\_pol3\_beta\_3 | Domain | 2 | 121 | 121 | 135.4 | 8e-40 | 1 | CL0060 | DNA polymerase III beta subunit, C-terminal domain |
| KSG66\_00015 | 5 | 69 | 5 | 70 | PF13275.6 | S4\_2 | Domain | 1 | 64 | 65 | 99.0 | 9.8e-29 | 1 | CL0492 | S4 domain |
| KSG66\_00020 | 2 | 348 | 2 | 368 | PF02463.19 | SMC\_N | Domain | 1 | 200 | 220 | 69.6 | 2.5e-19 | 1 | CL0023 | RecF/RecN/SMC N terminal domain |
| KSG66\_00025 | 3 | 72 | 2 | 73 | PF04025.12 | DUF370 | Family | 2 | 72 | 73 | 34.1 | 2.1e-08 | 1 | CL0007 | Domain of unknown function (DUF370) |
| KSG66\_00030 | 34 | 175 | 33 | 177 | PF02518.26 | HATPase\_c | Domain | 2 | 110 | 112 | 77.5 | 1e-21 | 1 | CL0025 | Histidine kinase-, DNA gyrase B-, and HSP90-like ATPase |

  

##### 7.2 PHI 注释

PHI-base（Pathogen Host Interactions），病原与宿主互作数据库，其内容经过实验验证，主要来源于真菌、卵菌和细菌病原，感染的宿主包括动物、植物、真菌以及昆虫。使用 blastp 默认参数进行比对。

- 样本Bac PHI比对结果： Bac.PHI.blast.xls

Tab 7-2-1 样本 Bac PHI 注释结果表(前10行)

| Query\_id | Subject\_id | Identity | Match\_length | Mismatch | GapOpen | Query\_start | Query\_end | Subject\_start | Subject\_end | Evalue | BitScore | Protein ID source | Gene | Pathogen species | Disease | Host description | Host species | Gene Function |
| --- | --- | --- | --- | --- | --- | --- | --- | --- | --- | --- | --- | --- | --- | --- | --- | --- | --- | --- |
| KSG66\_00035 | Q75WC5 | 47.1 | 839 | 398 | 4 | 3 | 796 | 2 | 839 | 9.9e-212 | 732.3 | Uniprot | gyrA | Burkholderia glumae | Bacterial grain rot | Monocots | Oryza sativa (related: rice) | DNA gyrase |
| KSG66\_00035 | Q75WC1 | 47.0 | 839 | 399 | 4 | 3 | 796 | 2 | 839 | 4.9e-211 | 729.9 | Uniprot | GyrA | Burkholderia glumae | Bacterial grain rot | Monocots | Oryza sativa (related: rice) | DNA gyrase (bacterial topoisomerase II) |
| KSG66\_00035 | Q75WC4 | 47.0 | 839 | 399 | 4 | 3 | 796 | 2 | 839 | 1.1e-210 | 728.8 | Uniprot | GyrA | Burkholderia glumae | Bacterial grain rot | Monocots | Oryza sativa (related: rice) | DNA gyrase (bacterial topoisomerase II) |
| KSG66\_00035 | Q75WC2 | 46.8 | 839 | 400 | 4 | 3 | 796 | 2 | 839 | 1.9e-210 | 728.0 | Uniprot | GyrA | Burkholderia glumae | Bacterial grain rot | Monocots | Oryza sativa (related: rice) | DNA gyrase (bacterial topoisomerase II) |
| KSG66\_00035 | Q75WC3 | 46.7 | 839 | 401 | 4 | 3 | 796 | 2 | 839 | 7.1e-210 | 726.1 | Uniprot | GyrA | Burkholderia glumae | Bacterial grain rot | Monocots | Oryza sativa (related: rice) | DNA gyrase (bacterial topoisomerase II) |
| KSG66\_00035 | Q6F4M1 | 68.2 | 132 | 42 | 0 | 43 | 174 | 1 | 132 | 4.3e-50 | 195.3 | Uniprot | GyrA | Flavobacterium psychrophilum | Bacterial cold-water disease | Bony Fishes | Oncorhynchus kisutch (related: coho salmon) | DNA gyrase (bacterial topoisomerase II) |
| KSG66\_00035 | Q6F4K2 | 68.2 | 132 | 42 | 0 | 43 | 174 | 1 | 132 | 1.3e-49 | 193.7 | Uniprot | GyrA | Flavobacterium psychrophilum | Bacterial cold-water disease | Bony fishes | Oncorhynchus mykiss (related: rainbow trout) | DNA gyrase (bacterial topoisomerase II) |
| KSG66\_00070 | D5AF41 | 68.3 | 489 | 152 | 1 | 2 | 487 | 4 | 492 | 2.7e-193 | 670.2 | Uniprot | IMPDH | Streptococcus suis | Meningitis | Even-toed ungulates | Sus scrofa (related: pig) | Inosine-5'-monophosphate dehydrogenase |
| KSG66\_00070 | E0SN64 | 56.2 | 484 | 207 | 3 | 5 | 485 | 3 | 484 | 6.0e-148 | 519.6 | Uniprot | guaB (Dda3937\_00004) | Dickeya dadantii | Soft rot disease | Eudicots | Cichorium intybus (related: chicory) | IMP dehydrogenase |
| KSG66\_00070 | G4N6P6 | 41.2 | 464 | 255 | 6 | 10 | 456 | 48 | 510 | 4.1e-88 | 320.9 | Uniprot | MoImd4 (MGG\_03699) | Magnaporthe oryzae | Rice blast | Monocots | Oryza sativa (related: rice) | Inosine-5'-monophosphate dehydrogenase |

  

##### 7.3 CAZy 注释

CAZy 全称为Carbohydrate-Active enZYmes Database，是碳水化合物酶相关的专业数据库，包括催化碳水化合物生物合成、降解以及修饰的相关酶系家族，对生物质转化等工业微生物领域有重要参考价值。  
使用blastp将基因序列与数据库比对注释。

- 样本Bac CAZy注释表： Bac.CAZy.annot.xls

- 样本Bac CAZy分类表： Bac.CAZy.class.xls

Tab 7-3-1 样本 Bac CAZy 注释结果表(前10行)

| Query\_id | Subject\_id | Identity | Align\_length | Miss\_match | Gap | Query\_start | Query\_end | Subject\_start | Subject\_end | E\_value | Score | CAZy\_levelB |
| --- | --- | --- | --- | --- | --- | --- | --- | --- | --- | --- | --- | --- |
| KSG66\_00035 | AKQ02536.1 | 60.8 | 97 | 37 | 1 | 194 | 289 | 482 | 578 | 8.7e-24 | 115.5 | AKQ02536.1 GT51 |
| KSG66\_00070 | AUX29448.1 | 33.5 | 474 | 301 | 9 | 11 | 477 | 352 | 818 | 2.8e-62 | 242.7 | AUX29448.1 GT4 |
| KSG66\_00075 | ASK79792.1 | 25.4 | 413 | 244 | 13 | 7 | 403 | 2 | 366 | 5.1e-10 | 68.9 | ASK79792.1 CBM50 |
| KSG66\_00090 | EAA63677.1 | 35.0 | 351 | 194 | 10 | 102 | 423 | 1332 | 1677 | 1.5e-40 | 170.2 | EAA63677.1 GH1\|GH95 |
| KSG66\_00115 | AVV92488.1 | 100.0 | 427 | 0 | 0 | 1 | 427 | 1 | 427 | 8.1e-215 | 749.2 | AVV92488.1 CBM50\|GH18 |
| KSG66\_00115 | AWE17667.1 | 100.0 | 427 | 0 | 0 | 1 | 427 | 1 | 427 | 8.1e-215 | 749.2 | AWE17667.1 CBM50\|GH18 |
| KSG66\_00115 | AXY68785.1 | 100.0 | 427 | 0 | 0 | 1 | 427 | 1 | 427 | 8.1e-215 | 749.2 | AXY68785.1 CBM50\|GH18 |
| KSG66\_00115 | QBK10278.1 | 100.0 | 427 | 0 | 0 | 1 | 427 | 1 | 427 | 8.1e-215 | 749.2 | QBK10278.1 CBM50\|GH18 |
| KSG66\_00115 | QDL80410.1 | 100.0 | 427 | 0 | 0 | 1 | 427 | 1 | 427 | 8.1e-215 | 749.2 | QDL80410.1 CBM50\|GH18 |
| KSG66\_00115 | ANF34925.1 | 99.5 | 426 | 2 | 0 | 2 | 427 | 1 | 426 | 6.9e-214 | 746.1 | ANF34925.1 CBM50\|GH18 |

  

Fig 7-3-1 CAZy 分类图

  

##### 7.4 分泌蛋白预测

分泌蛋白指在细胞内合成后，在信号肽的指导下穿过细胞膜分泌到细胞外起作用的蛋白质，如一些酶类，抗体和一部分激素等。许多致病因子或小分子代谢物都属于分泌蛋白，对于病原菌及代谢菌的研究有重要意义。

Tab 7-4-1 分泌蛋白预测统计

| Sample | Total\_Protein | SignalP | Transmembrane | Secret |
| --- | --- | --- | --- | --- |
| Bac | 3662 | 188 | 77 | 111 |

  

**含信号肽蛋白预测**

我们使用软件SignalP 4.1 对预测基因的蛋白序列进行分析，鉴定含有信号肽的蛋白。

Tab 7-4-2 含信号肽蛋白预测

| Gene\_ID | Signal peptide | cleave site | D score | neural\_networks |
| --- | --- | --- | --- | --- |
| KSG66\_00005 | N | 51 | 0.098 | SignalP-noTM |
| KSG66\_00010 | N | 38 | 0.114 | SignalP-noTM |
| KSG66\_00015 | N | 22,42 | 0.121 | SignalP-noTM |
| KSG66\_00020 | N | 18 | 0.123 | SignalP-noTM |
| KSG66\_00025 | N | 38,67 | 0.111 | SignalP-noTM |
| KSG66\_00030 | N | 12,56 | 0.127 | SignalP-noTM |
| KSG66\_00035 | N | 13,27 | 0.143 | SignalP-noTM |
| KSG66\_00065 | N | 33,53 | 0.099 | SignalP-noTM |
| KSG66\_00070 | N | 61 | 0.100 | SignalP-noTM |
| KSG66\_00075 | Y | 32 | 0.763 | SignalP-noTM |

  

**跨膜蛋白预测**

我们使用软件 TMHMM 对含有信号肽的蛋白序列进行分析，找出含有跨膜螺旋的蛋白，即跨膜蛋白。

Tab 7-4-3 跨膜蛋白预测详细信息

| ID | Length | ExpAA | First60 | PredHel | Topology |
| --- | --- | --- | --- | --- | --- |
| KSG66\_00075 | 443 | 16.11 | 12.98 | 1 | i9-27o |
| KSG66\_00435 | 296 | 17.23 | 17.23 | 1 | i5-23o |
| KSG66\_00930 | 185 | 0.32 | 0.32 | 0 | o |
| KSG66\_01045 | 318 | 2.94 | 2.94 | 0 | o |
| KSG66\_01055 | 414 | 16.98 | 16.94 | 1 | i7-24o |
| KSG66\_01060 | 637 | 2.69 | 2.68 | 0 | o |
| KSG66\_01245 | 158 | 23.22 | 21.95 | 1 | i5-27o |
| KSG66\_01270 | 246 | 6.94 | 6.91 | 0 | o |
| KSG66\_01285 | 295 | 20.98 | 20.95 | 1 | i7-26o |
| KSG66\_01505 | 371 | 7.19 | 7.18 | 0 | o |

  

**分泌蛋白预测**

筛选具有信号肽而在信号肽区域以外不具有跨膜螺旋结构的蛋白作为预测的分泌蛋白。

Tab 7-4-4 分泌蛋白预测详细信息

| Gene\_ID | Signal peptide | cleave site | D score | neural\_networks | Length | ExpAA | First60 | PredHel | Topology |
| --- | --- | --- | --- | --- | --- | --- | --- | --- | --- |
| KSG66\_00930 | Y | 27 | 0.699 | SignalP-noTM | 185 | 0.32 | 0.32 | 0 | o |
| KSG66\_01045 | Y | 20 | 0.892 | SignalP-noTM | 318 | 2.94 | 2.94 | 0 | o |
| KSG66\_01060 | Y | 21 | 0.837 | SignalP-noTM | 637 | 2.69 | 2.68 | 0 | o |
| KSG66\_01270 | Y | 27 | 0.680 | SignalP-noTM | 246 | 6.94 | 6.91 | 0 | o |
| KSG66\_01505 | Y | 20,29 | 0.657 | SignalP-noTM | 371 | 7.19 | 7.18 | 0 | o |
| KSG66\_01525 | Y | 22 | 0.721 | SignalP-noTM | 331 | 8.25 | 8.25 | 0 | o |
| KSG66\_01560 | Y | 20,27 | 0.610 | SignalP-noTM | 319 | 11.82 | 11.82 | 0 | o |
| KSG66\_01625 | Y | 20,32 | 0.678 | SignalP-noTM | 293 | 2.68 | 2.63 | 0 | o |
| KSG66\_01690 | Y | 26 | 0.946 | SignalP-noTM | 94 | 14.17 | 14.13 | 0 | o |
| KSG66\_01950 | Y | 23 | 0.775 | SignalP-noTM | 263 | 2.25 | 2.25 | 0 | o |

  

##### 7.5 TNSS效应蛋白预测

效应蛋白（effector）是细菌分泌系统研究中的一个热点，病原菌通过分泌系统TNSS（type N secretion systems，I型-VII型）将效应蛋白分泌至胞外或是宿主细胞中，影响细胞进程中免疫应答反应、细胞衰亡等各种重要活动最终引起病理反应。革兰氏阴性菌的T3SS是研究比较多的分泌系统，其中效应蛋白是T3SS分泌系统发挥毒力的部分，与细菌的致病性密切相关，常用于分子水平病原菌的感染机制、毒力作用等研究。  
我们基于蛋白序列功能数据库（KEGG，Nr）注释结果，提取分泌系统TNSS（type N secretion systems，I型-VII型）相关蛋白。并采用EffectiveT3软件对革兰氏阴性菌进行T3SS效应蛋白的预测。

Tab 7-5-1 TNSS系统效应蛋白注释结果

| GeneID | Symbol | Type | Nr | Pathway | KO |
| --- | --- | --- | --- | --- | --- |
| KSG66\_08175 | fliP | III | flagellar type III secretion system pore protein FliP [Bacillus velezensis FZB42] [Bacillus velezensis] | ko02040//Flagellar assembly//Cell motility//Cellular Processes | K02419//fliP; flagellar biosynthetic protein FliP |
| KSG66\_14785 | yukC | VII | type VII secretion protein EssB [Bacillus velezensis] | -- | -- |

  

##### 7.6 双组分系统预测

双组分系统（two-component system，TCSs）是机体识别和应对复杂环境变化的刺激-响应偶联机制。双组分系统的存在使细菌可以感受、应对、适应广泛的生存环境类型、环境压力，在渗透压等的刺激响应、群体感应、趋化性、耐药性等方面有重要作用。基于双组分系统的特征，我们预测并绘制细菌基因组上的双组分系统。

Tab 7-6-1 TCS预测结果

| GeneID\_HK | Transmembrane\_Helix | HisKA | HATPase\_c | GeneID\_RR | Response\_reg |
| --- | --- | --- | --- | --- | --- |
| KSG66\_01160 | 12-34;39-61 | - | 217-326 | KSG66\_01155 | 7-116 |
| KSG66\_01405 | 20-37;42-64;79-98;103-125;140-157 | - | 295-404 | KSG66\_01410 | 3-115 |
| KSG66\_01990 | 10-32;168-187 | 249-315 | 360-471 | KSG66\_01985 | 3-112 |
| KSG66\_02375 | 13-35;174-193 | - | 417-527 | KSG66\_02380 | 5-117 |
| KSG66\_02880 | 2-24;29-51;71-93;103-120;127-149 | 181-249 | 286-380 | KSG66\_02885 | 4-115 |
| KSG66\_03775 | 15-36;177-196 | - | 421-527 | KSG66\_03780 | 4-116 |
| KSG66\_04005 | 5-22;29-51 | 112-179 | 225-334 | KSG66\_04000 | 6-115 |
| KSG66\_04705 |  | 183-248 | 280-373 | KSG66\_04710 | 3-115 |
| KSG66\_06150 | 15-37;162-184 | 244-309 | 354-447 | KSG66\_06145 | 4-111 |
| KSG66\_06170 | 13-35 | - | 320-414 | KSG66\_06175 | 4-115 |

  

Fig 7-6-1 TCSs示意图

  

##### 7.7 CARD注释

CARD以Antibiotic Resistance Ontology（ARO）为分类单位（term）构建，用于关联抗生素模块及其目标、抗性机制、基因变异等信息。通过该数据库的注释，可以找到耐药性相关基因的名称，所耐受的抗生素种类等信息。  
我们使用Resistance Gene Identifier (RGI)将预测基因序列比对至数据库进行注释。

Tab 7-7-1 CARD注释表

| ORF\_ID | CUT\_OFF | PASS\_Bitscore | Best\_Hit\_Bitscore | Best\_Hit\_ARO | Best\_Identities | ARO | Model\_type | SNPs\_in\_Best\_Hit\_ARO | Other\_SNPs | Drug Class | Resistance Mechanism | AMR Gene Family |
| --- | --- | --- | --- | --- | --- | --- | --- | --- | --- | --- | --- | --- |
| KSG66\_00030 | Loose | 1000 | 853.203 | Clostridioides difficile gyrB conferring resistance to fluoroquinolone | 67.76 | 3004562 | protein variant model | R377G, I139R | T539N:2109 | fluoroquinolone antibiotic | antibiotic target alteration | fluoroquinolone resistant gyrB |
| KSG66\_00035 | Loose | 1500 | 996.112 | Clostridioides difficile gyrA conferring resistance to fluoroquinolones | 58.08 | 3003995 | protein variant model | P116A | Q106H:1545 | fluoroquinolone antibiotic | antibiotic target alteration | fluoroquinolone resistant gyrA |
| KSG66\_00120 | Loose | 300 | 23.8682 | Mycobacterium tuberculosis pncA mutations conferring resistance to pyrazinamide | 29.69 | 3003394 | protein variant model | N118T, D136N, T168N |  | pyrazinamide | antibiotic target alteration | pyrazinamide resistant pncA |
| KSG66\_00685 | Loose | 2300 | 1941.39 | Staphylococcus aureus rpoB mutants conferring resistance to rifampicin | 80.87 | 3003285 | protein variant model | A473T | D516G:1237, H526T:1237, L511R:1237, S512R:1237 | rifamycin antibiotic | antibiotic target alteration; antibiotic target replacement | rifamycin-resistant beta-subunit of RNA polymerase (rpoB) |
| KSG66\_00715 | Loose | 700 | 605.905 | Escherichia coli EF-Tu mutants conferring resistance to Enacyloxin IIa | 76.4 | 3003370 | protein variant model | Q124K | A376S:2137, R234F:2158 | elfamycin antibiotic | antibiotic target alteration | elfamycin resistant EF-Tu |
| KSG66\_00830 | Loose | 1300 | 30.0314 | Streptococcus pneumoniae PBP2b conferring resistance to amoxicillin | 22.9 | 3003042 | protein variant model | T488A | A100V:2380 | cephalosporin; cephamycin; penam | antibiotic target alteration | Penicillin-binding protein mutations conferring resistance to beta-lactam antibiotics |
| KSG66\_00875 | Loose | 1000 | 36.1946 | eatAv | 22.16 | 3003761 | protein variant model | T450I |  | macrolide antibiotic; lincosamide antibiotic; streptogramin antibiotic; tetracycline antibiotic; oxazolidinone antibiotic; phenicol antibiotic; pleuromutilin antibiotic | antibiotic target protection | ABC-F ATP-binding cassette ribosomal protection protein |
| KSG66\_01225 | Loose | 1000 | 36.1946 | eatAv | 23.49 | 3003761 | protein variant model | T450I |  | macrolide antibiotic; lincosamide antibiotic; streptogramin antibiotic; tetracycline antibiotic; oxazolidinone antibiotic; phenicol antibiotic; pleuromutilin antibiotic | antibiotic target protection | ABC-F ATP-binding cassette ribosomal protection protein |
| KSG66\_01770 | Loose | 800 | 25.7942 | Mycolicibacterium smegmatis ndh with mutation conferring resistance to isoniazid | 21.16 | 3003778 | protein variant model | H46P |  | isoniazid | antibiotic target alteration | antibiotic resistant ndh |
| KSG66\_01775 | Loose | 850 | 26.1794 | Staphylococcus aureus UhpT with mutation conferring resistance to fosfomycin | 21.2 | 3003902 | protein variant model | G358V |  | fosfomycin | antibiotic target alteration | UhpT |

  

##### 7.8 VFDB 注释

VFDB，Virulence Factors of Pathogenic Bacteria，毒力因子数据库，用于专门研究致病细菌、衣原体和支原体致病因子的数据库。包含24个种，共425个致病因子，24个致病岛，2359个与毒力因子的组成、结构、功能、致病机理和基因组等信息。毒力因子数据库能够为病原菌的入侵机理研究提供关键证据，同时结合比较基因组学等分析手段，可以从全基因组水平研究同属不同病原菌的毒力因子构成和基因组分布等特征。  
我们使用blastn与数据库进行比对注释。

Tab 7-8-1 VFDB注释表

| Query\_id | Subject\_id | Identity | Align\_length | Miss\_match | Gap | Query\_start | Query\_end | Subject\_start | Subject\_end | E\_value | Score | Description |
| --- | --- | --- | --- | --- | --- | --- | --- | --- | --- | --- | --- | --- |
| KSG66\_00580 | VFG000079 | 80.45 | 312 | 61 | 0 | 700 | 1011 | 697 | 1008 | 2e-30 | 135 | clpC - endopeptidase Clp ATP-binding chain C [Listeria monocytogenes EGD-e] (VF0072) |
| KSG66\_00580 | VFG000079 | 79.52 | 166 | 34 | 0 | 1766 | 1931 | 1763 | 1928 | 1e-07 | 60.0 | clpC - endopeptidase Clp ATP-binding chain C [Listeria monocytogenes EGD-e] (VF0072) |
| KSG66\_00580 | VFG000079 | 80.45 | 312 | 61 | 0 | 700 | 1011 | 697 | 1008 | 2e-30 | 135 | clpC - endopeptidase Clp ATP-binding chain C [Listeria monocytogenes EGD-e] (VF0072) |
| KSG66\_00580 | VFG000079 | 79.52 | 166 | 34 | 0 | 1766 | 1931 | 1763 | 1928 | 1e-07 | 60.0 | clpC - endopeptidase Clp ATP-binding chain C [Listeria monocytogenes EGD-e] (VF0072) |
| KSG66\_00580 | VFG049198 | 87.32 | 71 | 9 | 0 | 935 | 1005 | 932 | 1002 | 1e-10 | 69.9 | Kvar\_1151 - ATP-dependent chaperone ClpB [Klebsiella variicola At-22] (CVF861) |
| KSG66\_00580 | VFG049198 | 83.13 | 83 | 14 | 0 | 1717 | 1799 | 1897 | 1979 | 6e-06 | 54.0 | Kvar\_1151 - ATP-dependent chaperone ClpB [Klebsiella variicola At-22] (CVF861) |
| KSG66\_00580 | VFG049190 | 85.00 | 80 | 12 | 0 | 1843 | 1922 | 2023 | 2102 | 7e-09 | 63.9 | KOX\_00005 - protein disaggregation chaperone [Klebsiella oxytoca KCTC 1686] (CVF861) |
| KSG66\_00580 | VFG049189 | 85.00 | 80 | 12 | 0 | 1843 | 1922 | 1921 | 2000 | 7e-09 | 63.9 | A225\_4443 - clpB protein [Klebsiella oxytoca E718] (CVF861) |
| KSG66\_00580 | VFG049197 | 85.92 | 71 | 10 | 0 | 935 | 1005 | 944 | 1014 | 3e-08 | 61.9 | clpB - hypothetical protein [Klebsiella pneumoniae subsp. rhinoscleromatis SB3432] (CVF861) |
| KSG66\_00580 | VFG049196 | 85.92 | 71 | 10 | 0 | 935 | 1005 | 944 | 1014 | 3e-08 | 61.9 | clpB - protein disaggregation chaperone [Klebsiella pneumoniae subsp. pneumoniae NTUH-K2044] (CVF861) |

  

##### 7.9 次级代谢基因簇预测

一般情况下，参与次级代谢途径中生物合成酶的基因在染色体上成簇排列。基于指定类型的profile hidden Markov models, antismash 能准确鉴定所有已知的次级代谢基因簇。在antismash中，将次级代谢基因簇分为24类。最常见的次级代谢基因簇是type I polyketides synthase(T1pks)，type II polyketides synthase(T2pks)，type III polyketides synthase(T3pks)和non-ribosomal peptides synthase(NRPS)。例如：四环素、大环内酯类、安莎类、聚醚类由PKS途径合成；beta-内酰胺类、多肽类、糖肽类由NRPS途径合成。  
使用antismash 4.1.0 进行次级代谢基因簇的预测。预测结果如下

Tab 7-9-1 次级代谢产物基因簇预测结果(前10行)

| Cluster | Contig | Gene cluster Type | Gene cluster Genes |
| --- | --- | --- | --- |
| Cluster\_001 | CP077106 | nrps | KSG66\_01770;KSG66\_01775;KSG66\_01780;KSG66\_01785;KSG66\_01790;KSG66\_01795;KSG66\_01800;KSG66\_01810;KSG66\_01815;KSG66\_01820;KSG66\_01825;KSG66\_01830;KSG66\_01840;KSG66\_01845;KSG66\_01850;KSG66\_01855;KSG66\_01860;KSG66\_01865;KSG66\_01870;KSG66\_01875;KSG66\_01885;KSG66\_01890;KSG66\_01895;KSG66\_01900;KSG66\_01905;KSG66\_01910;KSG66\_01915;KSG66\_01920;KSG66\_01925;KSG66\_01930;KSG66\_01935;KSG66\_01940;KSG66\_01945;KSG66\_01950 |
| Cluster\_002 | CP077106 | otherks | KSG66\_04730;KSG66\_04735;KSG66\_04740;KSG66\_04745;KSG66\_04750;KSG66\_04755;KSG66\_04760;KSG66\_04765;KSG66\_04770;KSG66\_04775;KSG66\_04780;KSG66\_04785;KSG66\_04790;KSG66\_04795;KSG66\_04800;KSG66\_04805;KSG66\_04810;KSG66\_04815;KSG66\_04820;KSG66\_04825;KSG66\_04830;KSG66\_04835;KSG66\_04840;KSG66\_04845;KSG66\_04850;KSG66\_04855;KSG66\_04860;KSG66\_04865;KSG66\_04870;KSG66\_04875;KSG66\_04880;KSG66\_04885;KSG66\_04890;KSG66\_04895;KSG66\_04900;KSG66\_04905;KSG66\_04910;KSG66\_04915;KSG66\_04920;KSG66\_04925;KSG66\_04930 |
| Cluster\_003 | CP077106 | terpene | KSG66\_05335;KSG66\_05340;KSG66\_05345;KSG66\_05350;KSG66\_05355;KSG66\_05360;KSG66\_05365;KSG66\_05370;KSG66\_05375;KSG66\_05380;KSG66\_05385;KSG66\_05390;KSG66\_05395;KSG66\_05400;KSG66\_05405;KSG66\_05410;KSG66\_05420;KSG66\_05425;KSG66\_05430;KSG66\_05435;KSG66\_05440;KSG66\_05445;KSG66\_05450;KSG66\_05455 |
| Cluster\_004 | CP077106 | lantipeptide | KSG66\_06130;KSG66\_06135;KSG66\_06140;KSG66\_06145;KSG66\_06150;KSG66\_06155;KSG66\_06160;KSG66\_06165;KSG66\_06170;KSG66\_06175;KSG66\_06180;KSG66\_06185;KSG66\_06190;KSG66\_06195;KSG66\_06200;KSG66\_06205;KSG66\_06210;KSG66\_06215;KSG66\_06220;KSG66\_06225;KSG66\_06230;KSG66\_06235;KSG66\_06240;KSG66\_06245;KSG66\_06250;KSG66\_06255;KSG66\_06260;KSG66\_06265;KSG66\_06270;KSG66\_06275;KSG66\_06280;KSG66\_06285 |
| Cluster\_005 | CP077106 | transatpks | KSG66\_07165;KSG66\_07170;KSG66\_07175;KSG66\_07180;KSG66\_07185;KSG66\_07190;KSG66\_07195;KSG66\_07200;KSG66\_07205;KSG66\_07210;KSG66\_07215;KSG66\_07220;KSG66\_07225;KSG66\_07230;KSG66\_07235;KSG66\_07240;KSG66\_07245;KSG66\_07250;KSG66\_07255;KSG66\_07260;KSG66\_07265;KSG66\_07270;KSG66\_07275;KSG66\_07280;KSG66\_07285;KSG66\_07290;KSG66\_07295;KSG66\_07300;KSG66\_07305;KSG66\_07310;KSG66\_07315;KSG66\_07320;KSG66\_07325;KSG66\_07330;KSG66\_07335;KSG66\_07340;KSG66\_07345;KSG66\_07350;KSG66\_07355;KSG66\_07360;KSG66\_07370;KSG66\_07380;KSG66\_07385;KSG66\_07390 |
| Cluster\_006 | CP077106 | transatpks-nrps | KSG66\_08490;KSG66\_08495;KSG66\_08500;KSG66\_08505;KSG66\_08510;KSG66\_08515;KSG66\_08520;KSG66\_08525;KSG66\_08530;KSG66\_08535;KSG66\_08540;KSG66\_08545;KSG66\_08550;KSG66\_08555;KSG66\_08560;KSG66\_08565;KSG66\_08570;KSG66\_08575;KSG66\_08580;KSG66\_08585;KSG66\_08590;KSG66\_08595;KSG66\_08600;KSG66\_08605;KSG66\_08610;KSG66\_08615;KSG66\_08620;KSG66\_08625;KSG66\_08630;KSG66\_08635;KSG66\_08640;KSG66\_08645;KSG66\_08650;KSG66\_08655;KSG66\_08660;KSG66\_08665;KSG66\_08670;KSG66\_08675;KSG66\_08680;KSG66\_08685;KSG66\_08690;KSG66\_08695;KSG66\_08700;KSG66\_08705;KSG66\_08710;KSG66\_08715;KSG66\_08720 |
| Cluster\_007 | CP077106 | transatpks-nrps | KSG66\_09060;KSG66\_09065;KSG66\_09070;KSG66\_09075;KSG66\_09080;KSG66\_09085;KSG66\_09090;KSG66\_09095;KSG66\_09100;KSG66\_09105;KSG66\_09110;KSG66\_09115;KSG66\_09120;KSG66\_09125;KSG66\_09130;KSG66\_09135;KSG66\_09140;KSG66\_09145;KSG66\_09150;KSG66\_09155;KSG66\_09160;KSG66\_09165;KSG66\_09170;KSG66\_09175;KSG66\_09180;KSG66\_09185;KSG66\_09190;KSG66\_09195;KSG66\_09200;KSG66\_09205;KSG66\_09210;KSG66\_09215;KSG66\_09220;KSG66\_09225;KSG66\_09230;KSG66\_09240;KSG66\_09245;KSG66\_09250;KSG66\_09255;KSG66\_09260;KSG66\_09265;KSG66\_09270;KSG66\_09275;KSG66\_09280;KSG66\_09285;KSG66\_09290;KSG66\_09300;KSG66\_09305;KSG66\_09315;KSG66\_09320;KSG66\_09325;KSG66\_09330;KSG66\_09340;KSG66\_09345;KSG66\_09350;KSG66\_09355;KSG66\_09360;KSG66\_09365 |
| Cluster\_008 | CP077106 | terpene | KSG66\_09525;KSG66\_09530;KSG66\_09535;KSG66\_09540;KSG66\_09550;KSG66\_09555;KSG66\_09560;KSG66\_09565;KSG66\_09570;KSG66\_09575;KSG66\_09580;KSG66\_09585;KSG66\_09590;KSG66\_09595;KSG66\_09600;KSG66\_09605;KSG66\_09610;KSG66\_09615;KSG66\_09620;KSG66\_09625;KSG66\_09630;KSG66\_09635;KSG66\_09640 |
| Cluster\_009 | CP077106 | t3pks | KSG66\_10120;KSG66\_10125;KSG66\_10130;KSG66\_10135;KSG66\_10140;KSG66\_10145;KSG66\_10150;KSG66\_10155;KSG66\_10160;KSG66\_10165;KSG66\_10170;KSG66\_10175;KSG66\_10185;KSG66\_10190;KSG66\_10195;KSG66\_10200;KSG66\_10205;KSG66\_10210;KSG66\_10215;KSG66\_10220;KSG66\_10225;KSG66\_10230;KSG66\_10235;KSG66\_10240;KSG66\_10245;KSG66\_10250;KSG66\_10255;KSG66\_10260;KSG66\_10265;KSG66\_10270;KSG66\_10275;KSG66\_10280;KSG66\_10285;KSG66\_10290;KSG66\_10295;KSG66\_10300;KSG66\_10305;KSG66\_10310;KSG66\_10315;KSG66\_10325;KSG66\_10330;KSG66\_10335;KSG66\_10340;KSG66\_10345;KSG66\_10350;KSG66\_10355;KSG66\_10360;KSG66\_10365;KSG66\_10370;KSG66\_10375;KSG66\_10380;KSG66\_10385 |
| Cluster\_010 | CP077106 | transatpks | KSG66\_11025;KSG66\_11030;KSG66\_11035;KSG66\_11040;KSG66\_11045;KSG66\_11050;KSG66\_11055;KSG66\_11060;KSG66\_11065;KSG66\_11070;KSG66\_11075;KSG66\_11080;KSG66\_11085;KSG66\_11090;KSG66\_11095;KSG66\_11100;KSG66\_11105;KSG66\_11110;KSG66\_11115;KSG66\_11120;KSG66\_11125;KSG66\_11130;KSG66\_11135;KSG66\_11140;KSG66\_11145;KSG66\_11150;KSG66\_11155;KSG66\_11160;KSG66\_11165;KSG66\_11170;KSG66\_11175;KSG66\_11180;KSG66\_11185;KSG66\_11190;KSG66\_11195;KSG66\_11200;KSG66\_11205;KSG66\_11210;KSG66\_11215;KSG66\_11220;KSG66\_11225;KSG66\_11230;KSG66\_11235;KSG66\_11240;KSG66\_11245;KSG66\_11250;KSG66\_11255;KSG66\_11260;KSG66\_11265;KSG66\_11270;KSG66\_11275;KSG66\_11280;KSG66\_11285 |

  

Fig 7-9-1 次级代谢产物基因簇展示图

  

广州基迪奥生物科技有限公司


---

### 8 目录结构

```
GDD20120317-1_Genome_result                                报告总目录
├── 1_filter                                             测序评估结果目录
│   ├──Illumina                                              Illumina 数据质控目录
│   │   ├── *.{count,fill}.{pdf,png}                             reads 分布图
│   │   ├── *.{old,new}.{pdf,png}                                过滤前后碱基组成分布图
│   │   └── *_filter_stat.xls                                    数据过滤统计表
│   └──PacBio                                         PacBio 数据统计
│       ├── *.reads.length.{pdf,png}                             read长度统计图
│       └── *.reads.stat.xls                                     read统计
├── 2_Assembly                                           组装结果目录
│   ├── *.genome.fasta                                       组装的序列结果
│   ├── *_changes_stat.xls                                   校正结果统计表
│   ├── *_GC_Depth.{pdf,png}                                 GC-Depth 分布图
│   └── *.genome.stat.xls                                    组装结果统计表
├── 3_Component                                          基因组组分分析目录
│   ├── Gene_Prediction                                      编码基因预测目录
│   │   ├── *.{gene,protein}.fasta                               gene/protein 序列
│   │   ├── *.gene.length_distribution.{pdf,png}                 gene长度分布图
│   │   ├── *.gene.stat.xls                                      gene 统计
│   │   └── *.gtf                                                gtf 文件
│   ├── ncRNA                                                非编码RNA预测目录
│   │   ├── *.{rRNA,tRNA,sRNA}.fasta                             非编码RNA序列
│   │   ├── *.sRNA.xls                                           sRNA 预测结果
│   │   ├── *.ncRNA_stat.xls                                     非编码RNA统计
│   │   ├── *.rRNA.gff                                           rRNA gff文件
│   │   ├── *.tRNA.structure.txt                                 tRNA 二级结构表
│   │   └── *.tRNA.txt                                           tRNA 预测结果
│   ├── CRISPR                                               CRISPR 预测结果目录
│   │   ├── *.CRISPR.xls                                         CRISPR 预测结果
│   │   └── *.CRISPR_stat.xls                                    CRISPR 预测结果统计表
│   ├── GI                                                   Gene Islands 预测结果目录
│   │   ├── *.GIs.xls                                            Gene Islands 预测结果
│   │   └── *.GIs.stat.xls                                       Gene Islands 预测结果统计表
│   ├── Transposon                                           转座子预测结果目录
│   │   └── *.TransposonPSI.xls                                  转座子预测结果
│   ├── Prophage                                             前噬菌体预测目录
│   │   └── PFPR_result.xls                                      前噬菌体预测结果
│   └── Repeat                                               重复序列预测目录
│       ├── *.Interspersed_Repeats.stat.xls                      散在重复序列统计
│       ├── *.repeat.fasta                                       散在重复序列
│       ├── *.repeat.txt                                         散在重复序列预测结果
│       ├── *.Tandem_Repeat.stat.xls                             串联重复序列统计
│       └── *.Tandem_Repeat.xls                                  串联重复序列预测结果
├── 4_Basic_Annot                                        基因基本功能基本目录
│   ├── Basic_Annot_Summary                                  基本注释汇总目录
│   │   ├── *.4_database_annot.stat.xls                          四大数据库注释统计
│   │   ├── *.4_database_venn.{svg,png}                          四大数据库注释韦恩图
│   │   └── *.All_Genes.basic.annotation.xls                     基本注释汇总表
│   ├── GO                                                   GO 注释
│   │   ├── *.gene2GO.xls                                        GO 注释表
│   │   ├── *.GO2gene.xls                                        GO 分类表
│   │   └── *.GO.Level2.{svg,png}                                GO功能分类图
│   ├── KEGG                                                 KEGG 注释目录
│   │   ├── *.blast.kegg.xls                                     KEGG 比对结果
│   │   ├── *.htm                                                Parhway 分析结果网页报告
│   │   ├── *.ko                                                 基因ID 对应 KO 号列表
│   │   ├── *.kopath                                             基因ID 对应 KO、Pathway 列表
│   │   ├── *_map                                             t  Parhway 代谢通路图
│   │   ├── *.path.{pdf,png}                                  t  Parhway 分类图
│   │   └── *.path.xls                                           Pathway 列表
│   ├── COG                                                  COG 注释目录
│   │   ├── *.blast.cog.xls                                      COG 比对结果
│   │   ├── *.cog.class.annot.xls                                COG 分类表
│   │   ├── *.cog.gene.annot.xls                                 COG 注释
│   │   ├── *.cog.{pdf,png}                                      COG 分类图
│   ├── Nr                                                   Nr 注释目录
│   │   ├── *.blast.Nr.xls                                       Nr 比对结果
│   │   ├── *.Nr.species.stat.{pdf,png}                          Nr 物种统计图
│   │   └── *.Nr.species.stat.xls                                Nr 物种统计表
│   └── SwissProt                                            SwissProt 注释目录
│       └── *.blast.swsp.xls                                     SwissProt 比对结果
├── 5_Advanced_Annot                                     高级注释目录
│   ├── antiSMASH                                            antiSMASH 目录
│   │   ├── *.antiSMASH.xls                                      基因簇预测结果
│   │   └── *.gene_clusters.{svg,png}                            基因簇展示图
│   ├── CARD                                                 CARD 注释结果目录
│   │   └── *.CARD.xls                                           CARD 预测结果表
│   ├── CAZy                                                 CAZy 注释目录
│   │   ├── *.CAZy.5class.xls                                    CAZy大分类表
│   │   ├── *.CAZy.5class.xls.bar.{pdf,png}                      CAZy 统计图
│   │   ├── *.CAZy.annot.xls                                     CAZy 注释表
│   │   └── *.CAZy.class.xls                                     CAZy 分类表
│   ├── Pfam                                                 Pfam 注释目录
│   │   └── *.pfamA.xls                                          Pfam 注释表
│   ├── PHI                                                  PHI 注释目录
│   │   └── *.PHI.blast.xls                                      PHI 注释表
│   ├── TCS                                                  双组分系统预测结果目录
│   │   └── *.{svg,png}                                          双组分系统展示图
│   ├── VFDB                                                 VFDB 注释结果目录
│   │   └── *.VFDB.blast.xls                                     VFDB 注释结果表
│   ├── TNSS                                                 TNSS 注释结果目录
│   │   └── *.TNSS.xls                                           TNSS 注释结果表
│   └── Secretion                                            分泌蛋白预测结果目录
│       ├── *.secretion.stat.xls                                 分泌蛋白预测结果统计表
│       ├── *.secretion.xls                                      分泌蛋白预测结果表
│       ├── *.tmhmm.xls                                          跨膜蛋白预测结果表
│       └── *.signalP.xls                                        信号肽预测结果表
├── 6_Circos                                             基因组圈图目录
│   └── *.circular_karyo.{svg,png}                                基因组圈图
└── index.html                                           结题报告主页
```

广州基迪奥生物科技有限公司


---

### 9 软件和参考文献汇总

Tab 9-0-1 分析软件总览

| 分析软件 | 功能 | 版本/时间 | 参考文献/链接 |
| --- | --- | --- | --- |
| Fastp | Illumina测序数据质控 | version 0.20.0 | "Chen S, Zhou Y, Chen Y, et al. fastp: an ultra-fast all-in-one FASTQ preprocessor[J]. bioRxiv, 2018: 274100." |
| Falcon | PacBio数据组装基因组 | version 0.3.0 | "Chin C S, Peluso P, Sedlazeck F J, et al. Phased diploid genome assembly with single-molecule real-time sequencing[J]. Nature methods, 2016, 13(12): 1050." |
| Pilon | Illumina数据校正组装结果 | version 1.23 | "Walker BJ, Abeel T, Shea T, Priest M, Abouelliel A, et al. (2014) Pilon: An Integrated Tool for Comprehensive Microbial Variant Detection and Genome Assembly Improvement. PLoS ONE 9(11): e112963." |
| NCBI Prokaryotic Genome Annotation Pipeline | 基因预测和注释 |  | "Tatusova T, DiCuccio M, Badretdin A, et al. NCBI prokaryotic genome annotation pipeline[J]. Nucleic acids research, 2016, 44(14): 6614-6624." |
| Prokka | 基因预测 | version 1.11 | "Seemann T. Prokka: rapid prokaryotic genome annotation[J]. Bioinformatics, 2014, 30(14): 2068-2069." |
| tRNAscan | tRNA预测 | version 1.3.1 | "Lowe T M, Chan P P. tRNAscan-SE On-line: integrating search and context for analysis of transfer RNA genes[J]. Nucleic acids research, 2016, 44(W1): W54-W57." |
| RNAmmer | rRNA预测 | version 1.2 | "Lagesen K, Hallin P, R?dland E A, et al. RNAmmer: consistent and rapid annotation of ribosomal RNA genes[J]. Nucleic acids research, 2007, 35(9): 3100-3108." |
| cmscan | sRNA预测 | version 1.1.2 | "Nawrocki E P, Eddy S R. Infernal 1.1: 100-fold faster RNA homology searches[J]. Bioinformatics, 2013, 29(22): 2933-2935." |
| IslandPath-DIMOB | 基因岛GIs预测 | version 1.0.0 | "Bertelli C, Brinkman FSL. Improved genomic island predictions with IslandPath-DIMOB. Bioinformatics. 2018 Jul 1;34(13):2161-2167." |
| CRISPRfinder | CRISPR序列预测 | version 4.2.17 | "Grissa I, Vergnaud G, Pourcel C. CRISPRFinder: a web tool to identify clustered regularly interspaced short palindromic repeats[J]. Nucleic acids research, 2007, 35(suppl\_2): W52-W57." |
| TransposonPSI | 转座子预测 | version 20100822 | "Haas B. TransposonPSI: an application of PSI-Blast to mine (retro-) transposon ORF homologies[J]. Broad Institute, Cambridge, MA, USA, 2007." |
| RepeatMasker | 散在重复序列预测 | version 4.0.5 | "Tarailo‐Graovac M, Chen N. Using RepeatMasker to identify repetitive elements in genomic sequences[J]. Current protocols in bioinformatics, 2009, 25(1): 4.10. 1-4.10. 14." |
| TRF | 串联重复序列预测 | version 4.09 | "Benson G. Tandem repeats finder: a program to analyze DNA sequences[J]. Nucleic acids research, 1999, 27(2): 573-580." |
| phage\_finder | 前噬菌体预测 | version 2.0 | "Fouts D E. Phage\_Finder: automated identification and classification of prophage regions in complete bacterial genome sequences[J]. Nucleic acids research, 2006, 34(20): 5839-5851." |
| Pfam Scan | 用于Pfam数据库注释 | version 1.6 | "Finn R D, Bateman A, Clements J, et al. Pfam: the protein families database[J]. Nucleic acids research, 2013, 42(D1): D222-D230." |
| antiSMASH | 次级代谢基因簇预测 | version 4.1.0 | "Blin K, Wolf T, Chevrette M G, et al. antiSMASH 4.0—improvements in chemistry prediction and gene cluster boundary identification[J]. Nucleic acids research, 2017, 45(W1): W36-W41." |
| SignalP | 信号肽蛋白、跨膜蛋白预测 | version 4.1 | "Petersen T N, Brunak S, Von Heijne G, et al. SignalP 4.0: discriminating signal peptides from transmembrane regions[J]. Nature methods, 2011, 8(10): 785." |
| TMHMM | 跨膜蛋白预测 | version 2.0 | "Krogh A, Larsson B è, Von Heijne G, et al. Predicting transmembrane protein topology with a hidden Markov model: application to complete genomes[J]. Journal of molecular biology, 2001, 305(3): 567-580." |
| EffectiveT3 | 革兰氏阴性菌T3SS效应蛋白预测 | version 1.0.1 | "Arnold R, Brandmaier S, Kleine F, et al. Sequence-based prediction of type III secreted proteins[J]. PLoS pathogens, 2009, 5(4): e1000376." |
| BlastP | 氨基酸序列比对 | version 2.6.0 | Altschul S F, Gish W, Miller W, et al. Basic local alignment search tool[J]. Journal of molecular biology, 1990, 215(3): 403-410. |
| BlastN | 核酸序列比对 | version 2.7.1 | Altschul S F, Gish W, Miller W, et al. Basic local alignment search tool[J]. Journal of molecular biology, 1990, 215(3): 403-410. |
| DIAMOND | 氨基酸序列比对 | version 0.9.22 | https://github.com/bbuchfink/diamond |
| Blast2GO | GO注释 | version 2.3.5 | "Conesa A, G?tz S, García-Gómez J M, et al. Blast2GO: a universal tool for annotation, visualization and analysis in functional genomics research[J]. Bioinformatics, 2005, 21(18): 3674-3676." |
| RGI | CARD比对注释 | version 3.2.0 | "Jia B, Raphenya A R, Alcock B, et al. CARD 2017: expansion and model-centric curation of the comprehensive antibiotic resistance database[J]. Nucleic acids research, 2016: gkw1004." |

  

### 10 数据库总览

Tab 10-0-1 数据库总览

| 数据库 | 功能 | 版本/时间 | 软件 | 参数/比对阈值 | 数据库链接 |
| --- | --- | --- | --- | --- | --- |
| Nr | 非冗余的蛋白质数据库 | 20200419 | BlastP | evalue 1e-5 | NR |
| SwissProt | 高质量蛋白注释信息数据库 | 2020\_02 | Diamond | evalue 1e-5 | SwissProt |
| GO | 基因功能分类数据库 | 2019Nov | Blast2GO | 默认 | GO |
| KEGG | 京都基因与基因组百科全书 | 20200416 | Diamond | evalue 1e-5 | KEGG |
| COG | 同源蛋白簇数据库 | 2015 | Diamond | evalue 1e-5 | COG |
| Pfam | 蛋白家族数据库 | version 32.0 | Pfam\_Scan | 默认 | Pfam |
| CAZy | 碳水化合物酶数据库 | 20190808 | BlastP | evalue 1e-5 | CAZy |
| PHI-base | 病原宿主互作数据库 | version 4.8 | BlastP | evalue 1e-5 | PHI |
| CARD | 细菌耐药基因数据库 | version 3.0.8 | RGI | 默认 | CARD |
| VFDB | 毒力因子数据库 | 2020.04.17 | BlastN | evalue 1e-5 | VFDB |

  

### 11 附录

- 英文方法：细菌基因组测序英文方法
- 如果您的研究课题使用了基迪奥的测序和分析服务，我们期望您在论文发表时，在Method部分或Acknowledgement部分引用或提及基迪奥公司。可参考以下语句：**The cDNA / DNA / Small RNA libraries were sequenced on the Illumina/PacBio sequencing platform by Genedenovo Biotechnology Co., Ltd (Guangzhou, China). We are grateful to/thank Guangzhou Genedenovo Biotechnology Co., Ltd for assisting in sequencing and/or bioinformatics analysis.**

广州基迪奥生物科技有限公司


---

### 帮助文档
