# Supplementary material for: Isolation, identification, and whole-genome sequencing of high-yield protease bacteria from Daqu of ZhangGong Laojiu
Source: PLoS One. 2022 Apr 26;17(4):e0264677. doi: 10.1371/journal.pone.0264677 (PMC9041807; doi:10.1371/journal.pone.0264677)
Supplement: S1 Raw data — (ZIP) [file pone.0264677.s002.zip › Raw data/GDD20120317-1_Bacillus_velezensis_Genome_result/src/doc/Bac_Genome_ExpII.pdf]

DNA 提取及重测序文库构建

1. 基因组 DNA 的提取 2

1.1 CTAB 法提取基因组 DNA..........2

2. DNA 的质量检测..........3

2.1 NANODROP 微量分光光度计检测..........3

2.2 琼脂糖凝胶电泳检测..........4

3. 文库构建..........4

3.1 DNA 片段化..........5

3.2 末端修复、磷酸化并加 A..........5

3.3 接头连接..........6

3.4 片段筛选（插入片段 300-400 BP）..........6

3.5 PCR 文库富集..........7

3.6 用 AMPURE XP BEADS 纯化 PCR 反应..........7

4. 文库质检及测序..........8

1. 基因组 DNA 的提取

1.1 CTAB 法提取基因组 DNA

CTAB （十六烷基三甲基溴化铵）是一种阳离子去污剂，可溶解细胞膜，与核酸形成复合物，可使核酸沉淀出来。通过离心将 CTAB-核酸复合物与糖类、蛋白质等分离开来。随后将复合物溶于高盐溶液中，再加乙醇使核酸沉淀，而 CTAB 溶于乙醇，从而去除 CTAB。

主要仪器：4 ºC 离心机（型号 Eppendorf 5427R，公司德国艾本德股份公司，产地德国），振荡器（型号米欧 mix-28+，公司广州围古润仪器有限公司，产地中国），移液器（型号 Eppendorf，公司德国艾本德股份公司，产地德国），超纯水仪器（型号明澈 TM-D，公司 RephiLe Bioscience Ltd.，产地中国），水浴锅（型号精宏 DK-8D，公司广州合众生物科技有限公司，产地中国），-20ºC 冰箱（型号 DW-YW166A，公司中科美菱，产地中国）。

主要试剂：CTAB 试剂（公司广州化学试剂厂，产地广州），β-巯基乙醇（公司广州化学试剂厂，产地广州），氯仿（公司广州化学试剂厂，产地广州），异丙醇（公司广州化学试剂厂，产地广州），无水乙醇（公司广州化学试剂厂，产地广州），异戊醇（公司广州化学试剂厂，产地广州），酚：氯仿（25:24）（公司广州化学试剂厂，产地广州），RNase-Free Water （自配）。

实验步骤：

1) 取组织样品于液氮环境下充分研磨后，转移约 100 mg 至预冷的 2 mL 离心管中；
2) 加入 1 mL 预热的 CTAB 和 20 μL β-巯基乙醇，颠倒混匀；
3) 将混匀的样品放入 65 ℃ 水浴锅中约 1 h，水浴期间多次取出颠倒混匀；
4) 取出样品，冷却至室温后常温离心，12000 rpm 10 min；
5) 吸取上层水相，加入等体积的氯仿:异戊醇（24:1），充分混匀后常温离心，12000 rpm 10 min；
6) 吸取上层水相，加入等体积的酚:氯仿:异戊醇（25:24:1），充分混匀后常温

离心，12000 rpm 10 min；

7) 吸取上层水相，加入等体积的氯仿:异戊醇（24:1），充分混匀后常温离心，12000 rpm 10 min；
8) 吸取上层水相，加入等体积的异丙醇或 2 倍体积的无水乙醇，颠倒混匀，-20 ℃ 静置 30 min，常温离心，12000 rpm 10 min；
9) 弃上清，加 1 mL 75%乙醇，洗涤沉淀，室温离心，12000 rpm 5 min，弃上清；
10) 重复上一步；
11) 短暂离心，用移液器吸干乙醇，真空干燥 2-4 min；
12) 加入 50 µL ddH_2O，室温溶解 5 min，-20 ℃ 保存。

2. DNA 的质量检测

主要仪器：Nanorop 微量分光光度计（型号 NanoDrop 2000，公司赛默飞世尔科技，产地美国），琼脂糖凝胶电泳仪（型号 DYY-6C 型，公司北京六一仪器厂，产地北京），凝胶成像系统（型号 Tanon-2500，公司上海天能科技有限公司，产地中国）。

主要试剂：琼脂糖（型号 Biowest Agarose，供应商北京梦怡美商贸中心，品牌西班牙），goldview（型号 Goldview I，公司供应商北京梦怡美商贸中心，品牌 USA）。

2.1 NanoDrop 微量分光光度计检测

NanoDrop 检测核酸的 OD 值，A260/A280 比值应在 1.8-2.0 之间。

A260/A280<1.8 说明有蛋白质污染，>2.0 说明有 RNA 污染。

A260/230 比值在 2.2 左右，低于 1.8 表明有明显的有机物如糖、肽苯酚、盐离子等的污染。

2.2 琼脂糖凝胶电泳检测

质量完好的基因组 DNA 经琼脂糖凝胶电泳后，是一条单一的条带（如下图）。

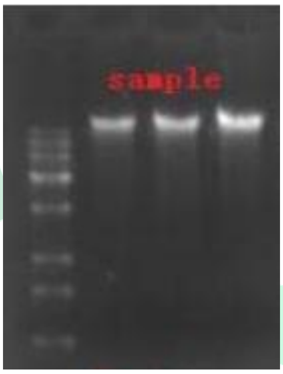

3. 文库构建

主要仪器：Novaseq 6000（Illumina），移液器（型号 Eppendorf，公司德国艾本德股份公司，产地德国），PCR 仪（型号 ETC811，公司东胜兴业科学仪器有限公司，产地中国）。

主要试剂：NEB 建库试剂盒（NEBNext® MLtra™ DNA Library Prep Kit for Illumina®,NEB#7370）无菌水（自配）。

流程图：

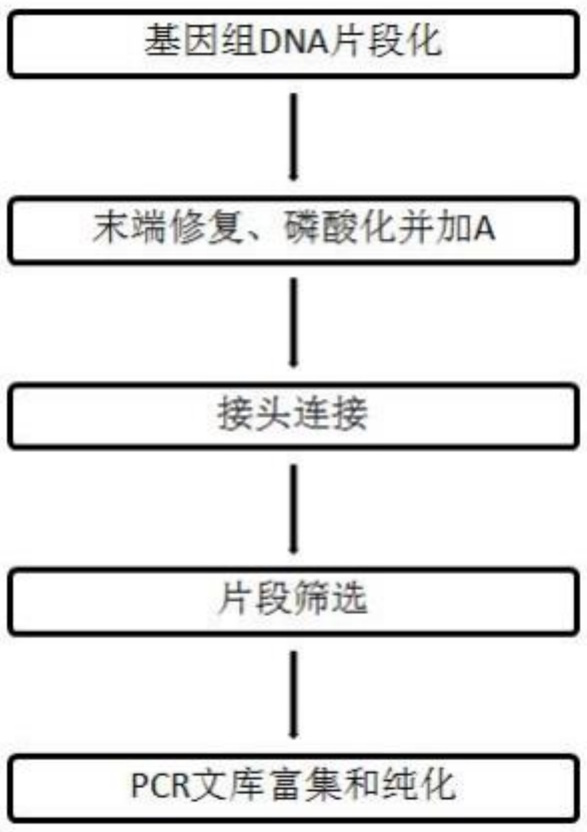

3.1 DNA 片段化

利用 NEBNext DNA 双链片段化酶，根据不同作用时间将 dsDNA 切割成 50-1000 bp 片段。单一片段的 PCR 扩增产物，原则上无需打断。

DNA (1 μg)	1-16 μL
10X Fragmentase Reaction Buffer v2	2 μL
Fragmentase	2 μL
Sterile Water	variable
Total volume	20 μL

3.2 末端修复、磷酸化并加 A

1) 取新的 nuclease free 离心管，按以下体系加入试剂，移液器吹打混匀。

End Prep Enzyme Mix	3μL
End Repair Reaction Buffer (10X)	6.5μL
Fragmented DNA	55.5μL
Total volume	65μL

2) 按以下 PCR 循环条件进行反应：

20 ℃	30 s
65 ℃	30 s
4 ℃	Forever

3.3 接头连接

1) 每个反应按如下试剂用量配制，并充分混匀。

Blunt/TA Ligase Master Mix	15 µL
NEBNext Adaptor for Illumina	2.5 µL
Ligation Enhancer	1µL
Total volume	83.5 µL

2) PCR 仪 20 □反应 15 min。

3) 在上一步混合液中加入 3 µL USER enzyme，充分混匀，37 □反应 15 min。

3.4 片段筛选（插入片段 300-400 bp）

1) 室温放置 AMPure XP beads，使其温度平衡至室温。

2) 加入 13.5 µL dH_2O 到连接产物中，使溶液总体系为 100 µL。

3) 加入 40 µL AMPure XP beads 到结合反应溶液中，移液器轻柔吹打至充分混匀。

4) 室温静置 5 min。

5) 反应管置于磁力架上 5 min；待溶液澄清后，始终保持样品于磁力架上，小心转移上清至新的 nuclease free 离心管。

6) 吸取 20 µL AMPure XP beads 至上清中，移液器轻柔吹打至溶液充分混匀。

7) 室温静置 5 min。

8) 反应管置于磁力架上 5 min；待溶液澄清后，始终保持样品于磁力架上，小心转移上清至新的 nuclease free 离心管。

9) 保持样品始终处于磁力架上，加入 200 µL 新鲜配制的 80%乙醇漂洗磁珠，室温孵育 30 s，小心移除上清。

10) 保持样品始终处于磁力架上，开盖空气干燥磁珠 5-10 min。

11) 将样品从磁力架中取出，加入 28 μL 10 mM Tris-HCl 或 0.1X TE(pH8.0) 进行 DNA 洗脱。移液器吹打充分混匀。

12) 室温静置 2 min。

13) 将反应管置于磁力架上，静置 5 min，待溶液澄清后，小心吸取 23 μL 上清至一个新的 nuclease free 离心管中，请勿触碰 AMPure XP beads。

3.5 PCR 文库富集

1) 按以下反应体系配制溶液，用移液器轻柔上下吹打混匀。

Adaptor Ligated DNA Fragments	23 μL
NEBNext High Fidelity 2X PCR Master Mix	25 μL
Index Primer	1 μL
Universal PCR Primer	1 μL
Total volume	50 μL

2) PCR 循环条件：

98 □ 30 s

(98 □ 10 s，65 □ 75 s，72 □ 30 s) 12 cycles

72 □ 5 min

4 □ Forever

3.6 用 AMPure XP Beads 纯化 PCR 反应

1) 室温平衡 AMPure XP beads 30 min，静置使其温度平衡至室温。

2) 颠倒或旋涡振荡使 AMPure XP beads 充分混匀，吸取 50 μL (1×) 加到 PCR 产物中，使用移液器轻柔吹打 10 次充分混匀。

3) 室温放置 5 min。

4) 反应管在磁力架上静置 5 min；待溶液澄清后，保持样品始终处于磁力架上，小心移除上清。

5) 保持管在磁力架上，加入 200 μL 新鲜配制的 80%乙醇漂洗磁珠，室温放置 30 s，然后小心移除上清。

6) 重复步骤 5)。

7) 保持样品始终处于磁力架中，开盖空气干燥磁珠 5-10 min。

8) 将样品从磁力架中取出，加入 33 μL 10 mM Tris-HCl (pH 8.0) 或 0.1X TE 进行 DNA 洗脱。

9)移液器吹打使充分混匀，室温静置 2 min。

10)将反应管短暂离心并置于磁力架上，静置 5 min，

11)待溶液澄清后，小心吸取 28 μL 上清至一个新的 nuclease free 离心管中，储存于-20 □中。

4. 文库质检及测序

主要仪器：安捷伦 2100（型号 Agilent 2100 Bioanalyzer，公司 Agilent Technologies，产地 Germany）。

主要试剂：质检试剂盒 DNA 1000 assay Kit 或 High Sensitivity DNA assay Kit（公司 Agilent Technologies，产地 Lithuania），ABI StepOnePlus Real-Time PCR System（公司 Life Technologies，产地美国）。

文库 2100 质检结果其峰图应为狭窄的单峰（如下图示），其片段大小根据插入片段大小而定。

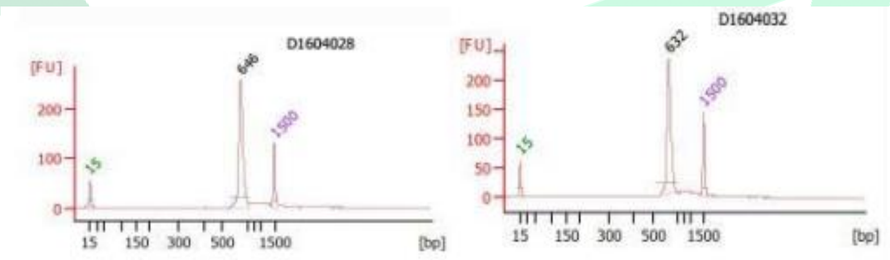

最后利用 ABI StepOnePlus Real-Time PCR System 进行定量，根据 Novaseq 6000 的 PE150 模式 Pooling 上机序。
