# Supplementary material for: Isolation, identification, and whole-genome sequencing of high-yield protease bacteria from Daqu of ZhangGong Laojiu
Source: PLoS One. 2022 Apr 26;17(4):e0264677. doi: 10.1371/journal.pone.0264677 (PMC9041807; doi:10.1371/journal.pone.0264677)
Supplement: S1 Raw data — (ZIP) [file pone.0264677.s002.zip › Raw data/GDD20120317-1_Bacillus_velezensis_Genome_result/src/doc/Bac_Genome_ExpIII.pdf]

三代 DNA 文库构建

1. CTAB 法基因组 DNA 的提取

CTAB 是一种阳离子去污剂，可溶解细胞膜，与核酸形成复合物，可使核酸沉淀出来。通过离心将 CTAB-核酸复合物与糖类、蛋白质等分离开来。随后将复合物溶于高盐溶液中，再加乙醇使核酸沉淀，而 CTAB 溶于乙醇，从而去除 CTAB。

主要仪器：4 ºC 离心机（型号 Eppendorf 5427R，公司德国艾本德股份公司，产地德国），振荡器（型号米欧 mix-28+，公司广州围古润仪器有限公司，产地中国），移液器（型号 Eppendorf，公司德国艾本德股份公司，产地德国），超纯水仪器（型号明澈 TM-D，公司 RephiLe Bioscience Ltd.，产地中国），水浴锅（型号精宏 DK-8D，公司广州合众生物科技有限公司，产地中国），-20ºC 冰箱（型号 DW-YW166A，公司中科美菱，产地中国）。

主要试剂：CTAB 试剂（公司广州化学试剂厂，产地广州），β-巯基乙醇（公司广州化学试剂厂，产地广州），氯仿（公司广州化学试剂厂，产地广州），异丙醇（公司广州化学试剂厂，产地广州），无水乙醇（公司广州化学试剂厂，产地广州），异戊醇（公司广州化学试剂厂，产地广州），酚：氯仿（25:24）（公司广州化学试剂厂，产地广州），RNase-Free Water （自配）。

实验步骤：

1) 取样品于液氮环境下充分研磨后，转移约 100 mg 至预冷的 2 mL 离心管中；
2) 加入 1 mL 预热的 CTAB 和 20 µL β-巯基乙醇，颠倒混匀；
3) 将混匀的样品放入 65 ℃ 水浴锅中约 1 h，水浴期间多次取出颠倒混匀；
4) 取出样品，冷却至室温后常温离心，12000 rpm 10 min；
5) 吸取上层水相，加入等体积的氯仿:异戊醇（24:1），充分混匀后常温离心，12000 rpm 10 min；

6) 吸取上层水相，加入等体积的酚:氯仿:异戊醇（25:24:1），充分混匀后常温离心，12000 rpm 10 min；
7) 吸取上层水相，加入等体积的氯仿:异戊醇（24:1），充分混匀后常温离心，12000 rpm 10 min；
8) 吸取上层水相，加入等体积的异丙醇或 2 倍体积的无水乙醇，颠倒混匀，-20 ℃ 静置 30 min，常温离心，12000 rpm 10 min；
9) 弃上清，加 1 mL 75%乙醇，洗涤沉淀，室温离心，12000 rpm 5 min，弃上清；
10) 重复上一步；
11) 短暂离心，用移液器吸干乙醇，真空干燥 2-4 min；
12) 加入 50 μL ddH_2O，室温溶解 5 min，-20 ℃ 保存。

2. DNA 的质量检测

主要仪器：Nanorop 微量分光光度计（型号 NanoDrop 2000，公司赛默飞世尔科技，产地美国），琼脂糖凝胶电泳仪（型号 DYY-6C 型，公司北京六一仪器厂，产地北京），凝胶成像系统（型号 Tanon-2500，公司上海天能科技有限公司，产地中国）。

主要试剂：琼脂糖（型号 Biowest Agarose，供应商北京梦怡美商贸中心，品牌西班牙），goldview（型号 Goldview I，公司供应商北京梦怡美商贸中心，品牌 USA）。

为保证测序质量，我们采用严格的指控把关文库的构建质量，检测标准如下：

琼脂糖凝胶电泳：分析样品 DNA 完整性；

NanoPhotometer spectrophotometer：检测 DNA 纯度（OD260/280 及 OD260/230 比值）。

3. 文库构建

AMPure PB Beads 纯化回收（可选）

如果样品浓度或纯度不达标可用 AMPure PB Beads 对 DNA 进行纯化回收。取 1-2 μgDNA 到新的 1.5 ml 离心管子。

（1）将 AMPure ® PB Bead 提前 30 min 从 2-8° C 取出，静

置使其温度平衡至室温。

（2）颠倒或旋涡振荡使 AMPure ® PB Bead 充分混匀，吸取等体积 (1 ×)加入到筛选后 DNA 样品中。

（3）在室温下，用宽口枪头缓慢吹打混匀 15 次,不要轻弹管子.室温孵育 5 min。

（4）孵育结束后，放入小离心机中短离 1 sec

（5）将样品置于磁力架上，待溶液澄清后（约 5 min），小心移除上清。

（6）保持样品处于磁力架上，加入 200 μl 新鲜配制的 80%乙醇漂洗磁珠，室温孵育 30 sec，小心移除上清。

（7）重复步骤 6 一次。

（8）取下管子放入小离心机中短离 3 sec。

（9）将样品置于磁力架上，约 10 sec，小心移除残余乙醇。

（10）保持样品处于磁力架上，在室温下开盖干燥磁珠约 5-10 min。

（11）将样品从磁力架上取出，加入 45.5 μl Elution Buffer，使用宽口枪头轻轻吸打充分混匀，室温静置 15 min 后置于磁力架上，待溶液澄清后（约 5 min）

（12）小心吸取 25 μl 上清至一个新的 Nuclease-free PCR 管中.

单链消化

试剂解冻后置于冰上，在冰上配置如下体系。

1. 用 Enzyme Dilution Buffer 稀释 DNA Prep Additive 配制成 Diluted DNA Prep Additive，混匀并短离。

试剂	体积
Enzyme Dilution Buffer	4.0 μL
DNA Prep Additive (stock)	1.0 μL
Total Volume	5.0μL

2. 使用下表建立一个反应，以去除 gDNA 单链末端。

试剂	体积

DNA Prep Buffer	7.0 μL
DNA	≤ 45.4 μL
Water	Up to 55 μL
NAD	0.6 μL
Diluted DNA Prep Additive	1.0 μL
DNA Prep Enzyme	1.0 μL
Total Volume	55.0 μL

3. 使用宽口枪头吹打混匀 10 次。不要轻弹管子。
4. 在微离心机中快速离心，将试管内的物质甩下。
5. 37 ℃孵育 15 min，4 ℃ Hold。继续下一步。

DNA 损伤修复

配置下面反应体系：

试剂	体积
DNA	55.0 μL
DNA Damage Repair Mix v2	2.0 μL
Total Volume	57.0 μL

1.使用宽口枪头吹打混匀 10 次。不要轻弹管子。
2. 在微离心机中快速离心，将试管内的物质甩下。
3. 37℃孵育 30 min，4 ℃ Hold。继续下一步。

DNA 末端修复和加 A

配置下面反应体系：

试剂	体积
DNA (Damage Repaired)	57.0 μL
End Prep Mix	3.0 μL
Total Volume	60.0 μL

1.使用宽口枪头吹打混匀 10 次。不要轻弹管子。

2. 在微离心机中快速离心，将试管内的物质甩下。

3. 20°C 孵育 10 min，65°C 孵育 30 min，4 °C Hold。 继续下一步。

接头连接

配置下面反应体系：

试剂	体积
DNA (End-Repaired)	60.0 μL
Overhang Adapter v3	5.0 μL
Ligation Mix	30.0 μL
Ligation Additive	1.0 μL
Ligation Enhancer	1.0 μL
Total Volume	97.0 μL

1.使用宽口枪头吹打混匀 10 次。不要轻弹管子。

2. 在微离心机中快速离心，将试管内的物质甩下。

3. 20℃孵育 60 min，4 ℃ Hold。 继续下一步。

连接产物用 0.45 X AMPure ® PB Bead 纯化两次

第一次纯化：

（1）连接产物中加入 3 μL Elution Buffer 使总体积达到 100 μl。

（2）颠倒或旋涡振荡使 AMPure ® PB Bead 充分混匀，吸取 45 μl (0.45 ×)加入到连接产物中。

（3）在室温下，用宽口枪头缓慢吹打混匀 15 次,不要轻弹管子.室温孵育 5 min。

（4）孵育结束后，放入小离心机中短离 1 sec。

（5）将样品置于磁力架上，待溶液澄清后（约 5 min），小心移除上清。

（6）保持样品处于磁力架上，加入 200 μl 新鲜配制的 80%乙醇漂洗磁珠，室温孵育 30 sec，小心移除上清。

（7）重复步骤 6 一次。

（8）取下管子放入小离心机中短离 3 sec。

（9）将样品置于磁力架上，约 10 sec，小心移除残余乙醇。

（10）保持样品处于磁力架上，在室温下开盖干燥磁珠约 5-10 min。

（11）将样品从磁力架上取出，加入 101 μl Elution Buffer，使用宽口枪头轻轻吸打充分混匀，室温静置 15 min 后置于磁力架上，待溶液澄清后（约 5 min）

（12）小心吸取 100 μl 上清至一个新的离心管中.

第二次纯化：

（1）连接产物中加入 3 μL Elution Buffer 使总体积达到 100 μl。

（2）颠倒或旋涡振荡使 AMPure ® PB Bead 充分混匀，吸取 45 μl (0.45 ×)加入到连接产物纯化一次后的样品中。

（3）在室温下，用宽口枪头缓慢吹打混匀 15 次,不要轻弹管子.室温孵育 5 min。

（4）孵育结束后，放入小离心机中短离 1 sec。

（5）将样品置于磁力架上，待溶液澄清后（约 5 min），小心移除上清。

（6）保持样品处于磁力架上，加入 200 μl 新鲜配制的 80% 乙醇漂洗磁珠，室温孵育 30 sec，小心移除上清。

（7）重复步骤 6 一次。

（8）取下管子放入小离心机中短离 3 sec。

（9）将样品置于磁力架上，约 10 sec，小心移除残余乙醇。

（10）保持样品处于磁力架上，在室温下开盖干燥磁珠约 5-10 min。

（11）将样品从磁力架上取出，加入 11 μl Elution Buffer，使用宽口枪头轻轻吸打充分混匀，室温静置 15 min 后置于磁力架上，待溶液澄清后（约 5 min）

（12）小心吸取 10 μl 上清至一个新的离心管中.

文库保存在-20℃。不反复冻融。
