# Supplementary material for: Isolation, identification, and whole-genome sequencing of high-yield protease bacteria from Daqu of ZhangGong Laojiu
Source: PLoS One. 2022 Apr 26;17(4):e0264677. doi: 10.1371/journal.pone.0264677 (PMC9041807; doi:10.1371/journal.pone.0264677)
Supplement: S1 Raw data — (ZIP) [file pone.0264677.s002.zip › Raw data/GDD20120317-1_Bacillus_velezensis_Genome_result/src/doc/Bac_Genome_Method.pdf]

Methods （Bacteria Genome Analysis）

Experimental procedure

1. DNA extraction

Genomic DNA was extracted using HiPure Bacterial DNA Kits (Magen, Guangzhou, China) according to the manufacturer's instructions. The DNA quality was detected using Qubit (Thermo Fisher Scientific, Waltham, MA) and Nanodrop (Thermo Fisher Scientific, Waltham, MA) accordingly.

2. PacBio or ONT sequencing

2.1 PacBio sequencing

Qualified genomic DNA was fragmented with G-tubes (Covaris, Woburn, MA, USA) and end-repaired to prepare SMRTbell DNA template libraries (with fragment size of >10 Kb selected by blue pippin system) according to the manufacturer's specification (PacBio, Menlo Park, CA). Liraray quality was detected by Qubit® 2.0 Flurometer (Life Technologies, CA, USA) and average fragment size was estimated on a Bioanalyzer 2100 (Agilent, Santa Clara, CA). SMRT sequencing was performed on the Pacific Biosciences Sequel (PacBio, Menlo Park, CA) according to standard protocols.

2.2 ONT sequencing

Qualified genomic DNA was fragmented with G-tubes (Covaris, Woburn, MA, USA) and end-repaired using NEBNext FFPE DNA Reapir kit (M6630, NEB, USA) and NEBNext End repair/dA-tailing Moudle reagents (E7546, NEB, USA). Then the fragments were ligated to adaptors to construct the sequencing library using NEBNext Quick Ligation Module reagents (E6056, NEB, USA). After purification using AMPure XP system (Beckman Coulter, Brea, CA, USA), library sequencing was performed on long-read sequencing platform Oxford Nanopore PromethION (Oxford Nanopore Technologies, Oxford, UK) according to standard protocols.

3. Illumina sequencing

Qualified genomic DNA was firstly sonicated randomly, and then end-repaired, A-tailed, and adaptor ligated using NEBNext® MLtra™ DNA Library Prep Kit for Illumina (NEB, USA) according to the preparation protocol. DNA fragments with length of 300-400 bp were enriched by PCR. At last, PCR products were purified using AMPure XP system (Beckman Coulter, Brea, CA, USA) and libraries were analysed for size distribution by 2100 Bioanalyzer (Agilent, Santa Clara, CA) and quantified using real-time PCR. Genome sequencing was performed on the Illumina Novaseq 6000 sequencer using the pair-end technology (PE 150).

Bioinformatic analysis

1. Denovo genome assembly and correction (Pacbio/ONT+Illumina)

Continuous long reads were attained from SMRT sequencing runs and were used for *de novo* assembly using Falcon (version0.3.0) [1].

Continuous long reads were attained from ONT sequencing and were used for *de novo* assembly using Flye (version 2.8.1-b1676) [21].

Raw data from Illumina platform were filtered using FASTP (version 0.20.0) [2] by the folowing standards,1) removing reads with ≥10 % unidentified nucleotides (N); 2) removing reads with ≥ 50 % bases having phred quality scores ≤20; 3) removing reads aligned to the barcode adapter. After filtering, resulted clean reads were used to correct the genome sequences to improve the quality of the assembly and determine the final genome sequences using Pilon (version 1.23) [3].

2. Genome component prediction

The ORFs (Open reading frames) were predicted using Prokka (version1.11) [4] or NCBI prokaryotic genome annotation pipeline [5]. Noncoding RNAs such as rRNAs prediction was carried out using rRNAmmer (version 1.2) [6] and tRNAs were identified by tRNAscan (version 1.3.1) [7], sRNAs were identified by cmscan (version 1.1.2) [8]. Gene Islands were predicted using IslandPath-DIMOB (version 1.0.0) [9]. CRISPR (Clustered Regularly Interspaced Short Palindromic Repeats) elements were predicted using CRISPRfinder (4.2.17) [10]. Transposons were predicted using TransposonPSI (version:20100822) [11]. Interpersed repeat elements were identified by RepeatMasker (version 4.0.5) [12]. Tendam repeat elements were identified by TRF (version 4.09) [13]. Prophage were identified using PHAST(version 2.0) [14].

3. Function annotations

We utilized several complementary approaches to annotate the assembled sequences. The genes were annotated by aligning with the deposited ones in diverse protein databases including National Center for Biotechnology Information (NCBI) non-redundant protein sequence (Nr) database, UniProt/Swiss-Prot, Kyoto Encyclopedia of Genes and Genomes (KEGG), Gene Ontology (GO), Cluster of Orthologous Groups of proteins (COG). Protein families annotation was applied with Pfam_Scan (version 1.6) [15] basing on Pfam database (version 32.0). Additional annotation was carried out basing on the following databases: Carbohydrate-Active enZYmes (CAZy), Pathogen Host Interactions (PHI), Virulence Factors of Pathogenic Bacteria (VFDB), CARD (Comprehensive Antibiotic Resistance Database). Gene clusters of secondary metabolites were predicted using antiSMASH (version 4.1.0) [16]. Two-component systems (TCSs) were predicted based

on their structure characteristics[17]. Secretory protein was predicted using SignalP4.0 [18]. Transmembrane protein was predicted using TMHMM (version 2.0) [19]. T3SS (type three secretion systems) were predicted using EffectiveT3 (version 1.0.1) [20].

References

[1] Chin C S, Peluso P, Sedlazeck F J, et al. Phased diploid genome assembly with single-molecule real-time sequencing[J]. Nature methods, 2016, 13(12): 1050.

[2] Chen S, Zhou Y, Chen Y, et al. fastp: an ultra-fast all-in-one FASTQ preprocessor[J]. bioRxiv, 2018: 274100.

[3] Walker BJ, Abeel T, Shea T, Priest M, Abouelliel A, et al. (2014) Pilon: An Integrated Tool for Comprehensive Microbial Variant Detection and Genome

Assembly Improvement. PLoS ONE 9(11): e112963.

[4] Seemann T. Prokka: rapid prokaryotic genome annotation[J]. Bioinformatics, 2014, 30(14): 2068-2069.

[5] Tatusova T, DiCuccio M, Badretdin A, et al. NCBI prokaryotic genome annotation pipeline[J]. Nucleic acids research, 2016, 44(14): 6614-6624.

[6] Lagesen K, Hallin P, Rødland E A, et al. RNAmmer: consistent and rapid annotation of ribosomal RNA genes[J]. Nucleic acids research, 2007, 35(9): 3100-3108.

[7] Lowe T M, Chan P P. tRNAscan-SE On-line: integrating search and context for analysis of transfer RNA genes[J]. Nucleic acids research, 2016, 44(W1): W54-W57.

[8] Nawrocki E P, Eddy S R. Infernal 1.1: 100-fold faster RNA homology searches[J]. Bioinformatics, 2013, 29(22): 2933-2935.

[9] Bertelli C, Brinkman FSL. Improved genomic island predictions with IslandPath-DIMOB. Bioinformatics. 2018 Jul 1;34(13):2161-2167.

[10] Grissa I, Vergnaud G, Pourcel C. CRISPRFinder: a web tool to identify clustered regularly interspaced short palindromic repeats[J]. Nucleic acids research, 2007, 35(suppl_2): W52-W57.

[11] Haas B. TransposonPSI: an application of PSI-Blast to mine (retro-) transposon ORF homologies[J]. Broad Institute, Cambridge, MA, USA, 2007.

[12] Tarailo-Graovac M, Chen N. Using RepeatMasker to identify repetitive elements in genomic sequences[J]. Current protocols in bioinformatics, 2009, 25(1): 4.10. 1-4.10. 14.

[13] Benson G. Tandem repeats finder: a program to analyze DNA sequences[J]. Nucleic acids research, 1999, 27(2): 573-580.

[14] Fouts D E. Phage_Finder: automated identification and classification of prophage regions in complete bacterial genome sequences[J]. Nucleic acids research, 2006, 34(20): 5839-5851.

[15] Finn R D, Bateman A, Clements J, et al. Pfam: the protein families database[J]. Nucleic acids research, 2013, 42(D1): D222-D230.

[16] Blin K, Wolf T, Chevrette M G, et al. antiSMASH 4.0—improvements in chemistry prediction and gene cluster boundary identification[J]. Nucleic acids research, 2017, 45(W1): W36-W41.

[17] Cheung J, Hendrickson W A. Sensor domains of two-component regulatory systems[J]. Current opinion in microbiology, 2010, 13(2): 116-123.

[18] Petersen T N, Brunak S, Von Heijne G, et al. SignalP 4.0: discriminating signal peptides from transmembrane regions[J]. Nature methods, 2011, 8(10): 785.

[19] Krogh A, Larsson B È, Von Heijne G, et al. Predicting transmembrane protein topology with a hidden Markov model: application to complete genomes[J]. Journal of molecular biology, 2001, 305(3): 567-580.

[20] Arnold R, Brandmaier S, Kleine F, et al. Sequence-based prediction of type III secreted proteins[J]. PLoS pathogens, 2009, 5(4): e1000376.

[21] Lin Y, Yuan J, Kolmogorov M, Shen MW, Chaisson M, Pevzner PA. Assembly of long error-prone reads using de Bruijn graphs. Proc Natl Acad Sci U S A. 2016;113(52):E8396-E8405. doi:10.1073/pnas.1604560113.
