# Supplementary material for: Isolation, identification, and whole-genome sequencing of high-yield protease bacteria from Daqu of ZhangGong Laojiu
Source: PLoS One. 2022 Apr 26;17(4):e0264677. doi: 10.1371/journal.pone.0264677 (PMC9041807; doi:10.1371/journal.pone.0264677)
Supplement: S1 Raw data — (ZIP) [file pone.0264677.s002.zip › Raw data/GDD20120317-1_Bacillus_velezensis_Genome_result/src/doc/help.html]

原始测序数据质控 

### 测序reads统计

我们将原始测序reads去除序列接头后获得的reads作为有效数据（clean data），将reads长度分布作为评估测序效果的主要内容。

**reads 信息统计表说明**

- Sample: 样本名
- Read Bases: 碱基数
- Number of Reads: read的数目
- Mean Length: read的平均长度
- Reads N50: 将所有read从长到短排序，并依次累加长度。当累加片段长度达到总片段长度（所有read 的长度）的 50% 时的那条read的长度

**reads 长度分布图**

x轴表示reads长度，y轴左侧坐标对应条形图，表示该长度下reads的数量，y轴右侧坐标对应曲线，曲线表示大于该长度的reads的累积数据量。

**Illumina 数据过滤统计表说明**

- Sample：样品名称
- Before filter：过滤前样品碱基信息
- After filter：过滤后样品碱基信息
- Bases：碱基总数（单位bp）
- Reads：reads总数
- Clean bases：过滤低质量reads后获得的有效数据碱基总数及占raw bases的百分比
- Clean reads：过滤低质量reads后获得的reads总数
- Q20（%）：测序碱基正确率达99%以上的碱基数及占过滤前/后碱基总数的比例
- Q30（%）：测序碱基正确率99.9%以上的碱基数占过滤前/后碱基总数的比例
- N（%）：N碱基数及占过滤前/后碱基总数的比例
- GC（%）：GC碱基数及占过滤前/后碱基总数的比例

**Illumina Reads分布图**

- Adaptor：表示含有接头序列的 reads 数及占总 reads 数的比例
- N containing：表示含未知碱基大于10%的 reads 数及占 reads 总数的比例
- Low quality：表示低质量的 reads 数及占 reads 总数的比例
- Clean：是原始序列数据经过去除杂质后得到的数据及占 reads 总数的比

**Illumina 碱基组成分布图**

图中，X轴上，1-150bp 代表 read1 的碱基位置，151-300bp 代表 read2 的碱基位置。  
A、T、C、G曲线各代表每个位置A、T、C、G碱基的比例。  
碱基组成平衡的情况下，A、T 曲线重合，G、C 曲线重合。  
如果测序中出现不正常的情况，碱基组成就可能不平衡。  
N 曲线代表每个位置未被测到的碱基的比例。  
mean 曲线表示碱基在每个位置的平均质量。  
Q20曲线代表碱基在每个位置的质量值≥Q20的碱基比例。  
过滤后的数据画碱基组成和质量值分布图可直观呈现数据质量情况。  

## 基因组组装

测序数据获得完整的基因组的基本原理：

#### 组装结果统计

**组装结果统计表说明**

- Sample：样本名字
- Total Number：Scaffold的个数
- Total length：组装结果的基因组大小
- Gap(N)：碱基N的个数
- GC content：GC含量的比例

**校正结果统计表说明**

- Sample：样品名称
- Genome size（bp）：基因组大小
- Correction（bp）：校正位点个数
- Insertion：校正插入的碱基数
- Deletion：校正删除的碱基数
- Substitution：校正替换的碱基数
- Percent：校正碱基数占基因组大小的比例
- Before GC%：校正前基因组GC含量
- After GC%：校正后基因组GC含量

**GC-Depth 分布图说明**

横轴表示GC含量，纵轴表示测序深度，右方是测序深度分布，上方是GC含量分布。由于每个物种的GC含量不同，同一个物种的GC含量会呈现集中分布。如果多数的点集中分布在一个比较窄的范围内，则表明不存在物种污染（左图）；如果分布在多个区域，则表明可能存在其他物种污染（右图）。

**reads 比对图**

组装结果末端和首端各取一段序列，两段序列拼在一起，将原始测序reads比对至拼接的序列，评估组装结果是否成环，即末端是否有缺失。  
如果大部分 reads 很好地连接了末端和首端，如下图的上半部分，说明组装结果末端没有缺失，已经成环。  
如果组装结果末端有缺失的序列，可以很清楚地在图中显示出来，如下图的下半部分，中间位置有 50bp 的缺失。  


**基因组圈图说明**

最外圈是基因组序列位置坐标，由外圈到里圈分别为：正链基因、负链基因、ncRNA(black表示tRNA, red表示rRNA)、GC含量（red 表示大于均值，blue表示小于均值）、GC skew（GC偏移，用来衡量G和C的相对含量，用于环状染色体中标记起点和终点；GC skew=(G-C)/(G+C)；purple表示大于0，orange表示小于0）。

## 基因组组分分析

### 编码基因预测

##### 编码基因预测结果统计表说明

- Sample：样本名字
- Total Number：Gene的个数
- Total length：基因总长度
- Maximum Length：最长的 Gene的长度
- Minimum Length：最短的Gene的长度
- GC content：GC含量的比例

**基因序列长度分布图图说明**

横坐标为预测的基因长度；纵坐标（左边）对应柱形图，表示该长度基因的数目；纵坐标（右边）对应折线图，表示该长度基因所占所有基因数目的百分比。

### 非编码RNA预测

**rRNA 预测结果表头说**

- ID: 编号
- Contig: Contig ID
- Start: 起始位置
- End: 终止位置
- Strand: 正负链
- Type: rRNA 类型

**tRNA 预测结果表头说**

- ID: 编号
- Contig: Contig ID
- Start: 起始位置
- End: 终止位置
- Type: tRNA携带的氨基酸类型
- Anti codon：tRNA上的反密码子
- Cove score：预测得分

#### sRNA 预测结果表头说明

- ID: 编号
- Contig: Contig ID
- Start: 起始位置
- End: 终止位置
- Strand: 正负链
- Target\_Family: 比对上数据库中的sRNA/miRNA 家族名称
- Target\_Acc: 比对上数据库中的sRNA/miRNA Accession编号
- Score: 比对分数
- E-value: E value值

#### 非编码RNA预测结果统计 的表头说明：

- Type：预测到的RNA类型
- Number：预测到的RNA个数
- Average length(bp)：预测到的该类型RNA的平均长度
- Total length：预测到的该类型RNA的总长度
- In genome(%)：预测到的RNA碱基数占基因组总碱基数的百分比

### 重复序列注释

        重复序列按照其在基因组上的的分布，可以分为两大类：散在重复序列、串联重复序列。散在重复序列是与串联重复序列的组织形式不同的另一类重复序列，是散在方式分布于基因组内的散在重复序列。这类DNA序列一般都是中度重复序列。利用RepeatMasker来预测基因组的散在重复序列。RepeatMasker是一款专门用于基因组重复序列识别的软件，几乎用于所有物种。我们利用RepeatMasker(http://www.repeatmasker.org/)来预测基因组的散在重复序列。然后利用专门的串联重复预测软件Tandem repeats finder来寻找串联重复。  
软件网址：http://www.repeatmasker.org/

#### 散在重复序列预测结果说明:

- Repeat type 类型：
- SINE，short interspersed nuclear elements，短散在重复序列
- LINE，long interspersed nuclear elements，长散在重复序列
- LTR，long terminal repeats，长末端重复序列
- DNA elements，DNA 转座子
- Unclassified，未定义类型

#### 串联重复序列预测结果表头说明:

- ID: 编号
- Contig: Contig序列ID
- Start: 起始位置
- End: 终止位置
- Repeat\_Size: 串联重复序列长度
- Consensus\_Size: 一致性序列长度
- Copies\_Number: 重复次数
- Score: 打分值
- Entropy: 熵值
- Consensus\_Pattern: 一致性序列
- Repeat\_Seq: 串联重复序列

#### 串联重复序列统计表说明:

- Number\_of\_Repeats: 串联重复序列总数
- Total\_Bases: 串联重复序列总碱基数
- Percentage\_of\_Sequence: 串联重复序列占基因组百分比

### 转座子

转座子是存在于染色体DNA上可以自主复制和移位的基本单位。转座过程指一段基因从原位上单独复制或断裂下来，环化后插入另一位点，并对其后的基因起调控作用。
最简单的转座子不含有任何宿主基因，常被称为插入序列(Insertional sequence，IS)，它们是细菌染色体或质粒DNA的正常组成部分，每个IS元件只编码转座酶。复合型转座子 (composite transposon)发现于细菌，带有同转座无关的一些基因，如抗药性基因；它的两端就是IS。Tn可以传播到其他细菌细胞，是自然界中细菌产生抗药性的重要来源。

#### 转座子预测结果表格说明:

- ID：Tn的编号
- Type：Tn类型，常见类型如下图
- Locus：Tn所在序列的编号
- Start：Tn的起始位置
- End：Tn的终止位置
- Strand：Tn所在的正负链

#### CRISPR序列预测

CRISPR（Clustered Regularly Interspaced Short Palindromic Repeats），规律成簇的间隔短回文重复，是大多数细菌、古细菌中一种天然免疫系统，对外来的质粒和噬菌体序列具有抵抗作用，能识别并使入侵的功能元件沉默，是一种对付攻击者的基因武器。

**CRISPR结果统计表说明**

- Total CRISPR: CRISPR 总数
- Credible CRISPR: 可靠的CRISPR数量
- Questionable CRISPR: 争议的CRISPR数量

**CRISPR结果说明**

- Crispr\_Id: CRISPR编号
- CRISPR\_Start: CRISPR起始位置
- CRISPR\_End: CRISPR终止位置
- CRISPR\_Length: CRISPR长度
- Potential\_Orientation: 序列方向
- Consensus\_Repeat: 一致性重复序列
- Repeat\_Length: 重复序列长度
- Spacers\_Nb: Spacer数量
- Evidence\_Level: 可信度，Level>=3 表明可信度较高

#### 基因岛预测

基因岛（Gene Islands，GIs）是有横向起源迹象的一部分基因组。一个基因岛可以与多种生物功能相关，能与共生或病原机理相关，与生物体的适应性相关等。基于其功能的不同可以划分为不同的子类，如病原性基因岛（PAIs）与致病原机理相关，抗生素抗性基因岛包含有许多抗生素抗性基因。在细菌中，许多III型分泌系统和IV型分泌系统位于基因岛内。碱基分析与系统发育判断表明，相同的基因岛能在近缘物种上发生各种水平（转化、结合、转导）的基因转移。许多基因岛有来源于外源基因如噬菌体、质粒的移动元件，两侧连有重复结构，另一些能自我剪切并转到其他合适的受体内。

**基因岛预测统计表**

- GIs number：预测的基因岛的个数
- Total length（bp）：基因岛的总长度
- Average length（bp）：基因岛的平均长度

**基因岛预测详细信息**

- GI\_ID：GI 编号
- Island start：基因岛在该序列的起始位置
- Island end：基因岛在该序列的终止位置
- Length：基因岛的长度
- Gene ID：基因编号
- Gene start：基因岛上基因的起始位置
- Gene end：基因岛上基因的终止位置
- Strand：基因所在的正负链信息
- Product：基因在Nr数据库的描述信息

#### 前噬菌体预测

整合在宿主基因组上的温和噬菌体的核酸称为前噬菌体（prophage）。带有前噬菌体的细菌称为溶源菌，具有无需外部感染而产生噬菌体的能力，并且这种能力可传递给后代。如果提供适当条件打破保持前噬菌体状态的机制，噬菌体基因组即可进行自主增殖，并使细胞裂解。  
前噬菌体序列的存在可能允许一些细菌获取抗生素抗性，增强对环境的适应性，提高粘附力或使细菌成为致病菌。同时，通过前噬菌体的研究有可能找到特异的抗生素甚至是先进的癌症治疗方法。  
使用软件Phage\_Finder 预测前噬菌体。  

**Prophage详细信息表**

- Number: Prophage数量
- Total\_Length: Prophage总长度
- Average\_Length: Prophage平均长度

**Prophage详细信息表**

- ID: 编号
- #asmbl\_id：Contig ID
- genome\_size：基因组大小
- genome\_gc：基因组 GC 含量
- begin\_region: 起始位置
- end\_region：终止位置
- size\_region：前噬菌体区域大小
- label：small/medium/large
- type：类型(prophage/integrated element/degenerate)
- region\_gc: 前噬菌体区域GC含量
- best\_db\_match：最优比对
- begin\_gene：起始基因
- end\_gene：终止基因
- region\_orientation：正负链
- #genes：前噬菌体区域包含的基因数量

### 基因功能注释

**四大数据库注释统计表：**

|  |  |
| --- | --- |
| Total Genes | 编码基因总数 |
| Nr | 能注释到Nr数据库的基因数 |
| Swissprot | 能注释到Swissprot数据库的基因数 |
| KOG | 能注释到KOG数据库的基因数 |
| Kegg | 能注释到Kegg数据库的基因数 |
| annotation gene | 有注释的基因的数目 |
| without annotation gene number | 没有注释的基因总数 |

**注释结果汇总表各列含义如下所示：**

|  |  |
| --- | --- |
| geneID | 基因ID |
| Symbol | Symbol 号 |
| Nr-ID | blast 比对到 Nr 数据库中序列名 |
| Nr-Score | blast 比对到 Nr 数据库的 Score |
| Nr-Evalue | blast 比对到 Nr 数据库的 Evalue |
| Nr-annotation | Nr 序列的描述 |
| Swissprot-ID | blast 比对到 Swissprot 数据库中序列名 |
| Swissprot-Score | blast 比对到 Swissprot 数据库的 Score |
| Swissprot-Evalue | blast 比对到 Swissprot 数据库的 Evalue |
| Swissprot-annotation | Swissprot 序列的描述 |
| COG/KOG-Protein-or-Domain | blast 比对到 COG/KOG 数据库中的蛋白或结构域 |
| COG/KOG-Score | blast 比对到 COG/KOG 数据库的 Score |
| COG/KOG-Evalue | blast 比对到 COG/KOG 数据库的 Evalue |
| COG/KOG-ID | COG/KOG 的 ID |
| COG/KOG-Function-Description | COG/KOG 功能描述 |
| KO-ID | KO 的 ID及注释 |
| KEGG-Evalue | blast 比对到 KEGG 数据库的 Evalue |
| KEGG-Score | blast 比对到 KEGG 数据库的 Score |
| KEGG-Gene | blast 比对到 KEGG 数据库中的序列名 |
| Pathway | Pathway |
| GO-BiologicalProcess | GO生物过程 |
| GO-MolecularFunction | GO分子功能 |
| Go-CellularComponent | GO细胞组分 |

  

  

通过blast将预测得到的基因序列比对到数据库 ，得到跟给定基因具有最高序列相似性的蛋白，从而得到该基因的蛋白功能注释信息。

#### blast比对各个数据库结果表各列含义如下所示：

|  |  |
| --- | --- |
| Query\_id | 基因ID |
| Subject\_id | 比对到数据库中序列名 |
| Identity | 比对的identity |
| Align\_length | 比对上的长度 |
| Miss\_match | 比对的错配数 |
| Gap | Gap的比例 |
| Query\_start | 比对上的部分在基因上的起始位置 |
| Query\_end | 比对上的部分在基因上的终止位置 |
| Subject\_start | 比对上的部分在数据库序列上的起始位置 |
| Subject\_end | 比对上的部分在数据库序列上的终止位置 |
| Positive | Positive的比例(Positive: 相同或相似的氨基酸视为Positive比对) |
| E\_value | 比对的Evalue |
| Score | 比对的分值 |
| Subject\_annotation | 数据库序列的描述 |
| Confident | 是否可信度较高，若 Identity >= 40% && Coverage >= 40% 为 "YES"，否则为 "NO" |

#### Nr注释

Nr，Non-redundant protein database，即非冗余的蛋白质数据库，由NCBI创建并维护，内容比较全面，同时注释结果中会包含物种信息，根据基因注释到的物种情况，统计注释到的物种及基因数目。  
使用blastp将基因编码的氨基酸序列和数据库比对获得注释信息。

**Nr 数据库注释物种统计图**

横坐标表示物种ID，纵坐标表示注释上的基因个数

#### SwissProt注释

SwissProt 是经过严格筛选的高质量蛋白数据库，其所含序列信息少于NR 数据库，但其包含的是经过人工认证过的序列，可信度比较高。  
使用diamond将基因编码的氨基酸序列和数据库比对获得注释信息。

#### COG注释

COG，Cluster of Orthologous Groups of proteins，由NCBI创建并维护的蛋白数据库，根据细菌、藻类和真核生物完整基因组中编码蛋白的系统进化关系构建而成。通过比对将某个蛋白序列注释到某一个COG中，每一簇COG由直系同源序列构成，从而可以推测该序列的功能。COG数据库按照功能分为26类，详见http://www.ncbi.nlm.nih.gov/COG/。构成每个COG 的蛋白被假定为来自一个祖先蛋白，由基因复制或垂直家系（物种形成）进化而来，保留与原始蛋白相似的功能，可用于蛋白功能预测、分类等。  
使用DIAMOND与COG（Cluster of Orthologous Groups of proteins）数据库比对，获得基因对应的注释结果，并根据注释结果对蛋白进行功能分类。

COG功能注释结果表各列含义如下所示：

- Query\_id 基因ID
- Subject\_id 比对到数据库中序列名
- Identity 比对的identity
- Align\_length 比对上的长度
- Miss\_match 比对的错配数
- Gap Gap的比例
- Query\_start 比对上的部分在基因上的起始位置
- Query\_end 比对上的部分在基因上的终止位置
- Subject\_start 比对上的部分在数据库序列上的起始位置
- Subject\_end 比对上的部分在数据库序列上的终止位置
- E\_value 比对的Evalue
- Score 比对的分值
- Protein-or-Domain 比对上的蛋白或结构域
- COG-ID COG的ID
- Function-Description 功能描述
- Code COG 功能代号
- Functional-Categories COG功能分类

**COG注释结果统计图**

横轴表示功能分类，以不同颜色展现，纵轴表示每个功能分类包含的预测基因的个数。

#### KEGG注释

KEGG（Kyoto Encyclopedia of Genes and Genomes），http://www.genome.jp/kegg/，系统分析基因产物和化合物在细胞中的代谢途径以及基因产物功能的数据库。  
在后基因时代一个重大挑战是如何使细胞和有机体在计算机上完整的表达和演绎，让计算机利用基因信息对更高层次和更复杂细胞活动和生物体行为作出计算推测。为达到此目的，人们建立了一个在相关知识基础上的网络推测计算工具。在给出染色体中一套完整的基因的情况下，它可以对蛋白质交互（互动）网络在各种细胞活动起的作用作出预测。  
KEGG 的 Pathway 数据库整合当前在分子互动网络（比如通道，联合体）的知识，KEGG 的 Genes/SSDB/KO 数据库提供关于在基因组计划中发现的基因和蛋白质的相关知识，KEGG 的Compound /Glycan/Reaction数据库提供生化复合物及反应方面的知识。  
KEGG是系统分析基因产物在细胞中的代谢途径以及基因产物功能的数据库，利用 KEGG可以进一步研究基因在生物学上的复杂行为。根据 KEGG 注释信息得到Gene的 Pathway注释。  
使用DIAMOND与KEGG数据库比对，获得基因所对应的注释结果。

注释到代谢通路结果表各列含义如下：

- KEGG\_A\_class ：A级分类
- KEGG\_B\_class ：B级分类
- Pathway ：通路名
- Count (\*) ：注释到该通路的基因的数目
- Pathway ID ：KEGG 数据库中的 Pathway ID
- Genes： 注释到该 Pathway 的基因
- KOs ：属于该 Pathway 的 KEGG Orthology

**KEGG注释结果统计图**

横轴表示基因数目，纵轴表示KEGG数据库的功能分类，黑色字体为level 1层级的分类，彩色字体为level 2层级。条形图上的数字代表注释上的基因数目。KEGG分类层级说明：https://www.kegg.jp/kegg/pathway.html#genetic。

#### GO注释

据 Nr 注释信息我们能得到 GO 功能注释。Gene Ontology（简称GO）是一个国际标准化的基因功能分类体系，提供了一套动态更新的标准词汇表（controlled vocabulary）来全面描述生物体中基因和基因产物的属性。  
GO注释包含三个 ontology，分别为：细胞组分（cellular component，the parts of a cell or its extracellular environment）；分子功能（molecular function，the elemental activities of a gene product at the molecular level, such as binding or catalysis）；生物过程（biological process，operations or sets of molecular events with a defined beginning and end, pertinent to the functioning of integrated living units: cells, tissues, organs, and organisms）。  
GO 的基本单位是 term（词条、节点），每个 term 都对应一个属性。   
我们根据 Nr 注释信息，使用 Blast2GO 得到基因的 GO 注释信息。Blast2GO 已被其它文献引用超过 150 次，是同行广泛认可的 GO 注释软件。  
得到每个基因的 GO 注释后，我们对所有基因做 GO 功能分类统计，从宏观上认识该物种的基因功能分布特征。

GO分类表各列含义如下：

- Level 1：GO本体的类别 （biological\_process 或 cellular\_component 或 molecular\_function）
- Level 2：GO 条目
- number\_of\_\*：注释到各 GO 条目 中的gene数量
- genes\_of\_\*：注释到各 GO 条目的 gene ID

#### 结构域分析（Pfam）

蛋白质一般包含1个或多个功能区域，称为结构域。不同结构域的组合产生了丰富的蛋白质，对蛋白质结构域的鉴定也可以推测蛋白质功能。Pfam（Protein families database of alignments and hidden Markov models ）提供了完整准确的蛋白质家族和结构域分类信息，以多序列比对信息和隐马尔可夫模型（HMM）表示，广泛应用于蛋白家族查询和蛋白结构域的注释。  
Pfam 包括 PfamA 和 PfamB。其中 PfamA 中所包含的蛋白结构数据都是已知并且得到验证的，每个蛋白结构域都有各自的定义（definition）。而 PfamB 中的数据是通过模型和算法预测出来的，并且未得到验证，是对 PfamA 的补充。

蛋白结构相关注释汇总表各列含义如下：

- seq id : Gene 的编号
- alignment start : Gene 编码蛋白序列比对的结构域起始位置
- alignment end : Gene 编码蛋白序列比对的结构域终止位置
- envelope start : HMM 模型预测的 Gene 编码蛋白序列的结构域起始位置
- envelope end : HMM 模型预测的 Gene 编码蛋白序列的结构域终止位置
- hmm acc : Gene 编码蛋白序列对应结构的 HMM 在 Pfam 中的编号
- hmm name : Gene 编码蛋白序列对应结构的 HMM 在 Pfam 中的名称
- type : Gene 编码蛋白序列匹配到 Pfam 数据库中对应结构的分类水平，蛋白家族或者结构域
- hmm start : 数据库中匹配序列的起始位置
- hmm end : 数据库中匹配序列的终止位置
- hmm length : 数据库中匹配序列的长度
- bit score : 根据比对和 HMM 模型得出的 Gene 编码蛋白序列结构的评分
- E-value : 比对注释的假阳性率
- significance : Gene 编码蛋白序列在数据库中匹配结构的数目
- clan : Pfam 数据库中按照蛋白质序列，结构以及 HMM 文件而分成的类群
- PfamA\_definition : 查询序列对应结构在 PfamA 中的名称

#### 病原与宿主互作数据库（PHI）注释

        PHI-base（Pathogen Host Interactions），病原宿主互作数据库，是一个免费开放的数据库，收录了经过实验验证或文献报道的能够感染植物、动物、真菌和昆虫的真菌、卵菌、细菌等病原菌的致病基因、毒力基因和效应蛋白基因。另外，数据库还收录了抗真菌化合物及其靶基因。
数据库基本每几个月就更新一次，目前数据库里共收录4460个基因、8046对互作关系、264个病原菌、176个宿主、410种疾病。PHI-base信息很齐全，会给出核酸序列、蛋白序列、功能注释、其他外部数据库的注释链接（Uniprot，Gene Ontology terms，EC Numbers，NCBI taxonomy，EMBL，PubMed and FRAC）。

PHI比对结果表格各列含义如下：

- Query\_id：基因组所预测基因编号
- Subject\_id：PHI数据库对应基因编号
- Identity：序列相似度
- Align\_length：序列比对上的长度
- Miss\_match：错配碱基数
- Gap：序列间隙
- Query\_start：目标基因比对起始位点
- Query\_end：目标基因比对终止位点
- Subject\_start：数据库对应基因比对起始位点
- Subject\_end：数据对应库基因比对终止位点
- E\_value：比对E值，越低表示可靠性越高
- Score：比对分值，越高表示比对结果越好
- PHI-base：PHI数据库对应基因编号
- DB\_Type：参考基因的数据库来源
- Accession：来源数据库对应编号
- Gene\_name：基因名称
- Pathogen\_NCBI\_Taxonomy\_ID：病原菌NCBI物种分类ID
- Pathogen\_species：病原菌物种
- Disease\_name：所引起疾病
- Monocot\_Dicot\_plant：宿主物种类别
- Host\_NCBI\_Taxonomy\_ID：宿主NCBI物种分类ID
- Experimental\_host：宿主
- Function：蛋白功能

#### 碳水化合物相关酶（CAZy）数据库注释

        CAZy 全称为Carbohydrate-Active enZYmes Database，碳水化合物酶相关的专业数据库，内容包括能催化碳水化合物降解、修饰、以及生物合成的相关酶系家族。其包含五个主要分类：糖苷水解酶（Glycoside Hydrolases, GHs）、糖基转移酶（Glycosyl Transferases, GTs）、多糖裂解酶（Polysaccharide Lyases, PLs）、碳水化合物辅助酶类（Auxiliary Activities, AAs）和糖类酯解酶（Carbohydrate Esterases, CEs）。此外，还包含与碳水化合物相关的modules（Carbohydrate-Binding Modules，CBMs）。

CAZy注释表各列含义如下：

- gene\_id ：gene 序列的 ID 号
- gene\_start ：比对上的部分在Gene 上的起始位置
- gene\_end ：比对上的部分在 Gene 上的终止位置
- identity ：比对的identity
- evalue ： 比对的Evalue
- subject\_id ：比对到数据库中序列名
- description : 数据库序列的描述
- CAZy\_levelB : CAZy levelB的名称

**CAZy分类统计图**

横轴表示酶的分类，纵轴表示该分类包含的基因数。

#### 分泌蛋白预测

分泌蛋白指在细胞内合成后，在信号肽的指导下穿过细胞膜分泌到细胞外起作用的蛋白质，如一些酶类，抗体和一部分激素等。许多致病因子或小分子代谢物都属于分泌蛋白，对于病原菌的研究有重要意义。引导新合成的蛋白质穿过膜结构的短肽链（长度5-30个氨基酸），即为信号肽，是分泌蛋白的标志之一。  
跨膜蛋白（transmembrane protein，TP)是一种贯穿生物膜两端的蛋白。许多跨膜蛋白的功能是作为通道或“装载码头”来实施拒绝或允许某种特定的物质跨过生物膜的运输、进入细胞，同时，也使废弃的副产品运出细胞。跨膜蛋白通过信号肽引导到达细胞膜，整合进细胞膜中，之后信号肽将被切除。

#### SignalP 结果统计表

|  |  |  |
| --- | --- | --- |
| total protein |  | 总蛋白数 |
| secretion protein |  | 分泌蛋白数量 |
| no secretion protein |  | 非分泌蛋白数量 |

**分泌蛋白预测统计**

- Sample：样品ID
- SignalP：具有信号肽结构的蛋白个数
- Transmembrane：具有跨膜结构的蛋白个数
- Secret：预测为分泌蛋白的个数

#### 含信号肽蛋白预测结果表

|  |  |  |
| --- | --- | --- |
| Gene\_ID |  | 基因编号 |
| Signal peptide |  | 是否预测为信号肽 |
| Cleave Site |  | 信号肽的氨基酸切断位置，如果为单数值，如19，则切断位点在第19个氨基酸；如果为范围，例如19,31，则切断位点在第19到31个氨基酸之间 |
| D score |  | D score: 信号肽预测分值，当此数值大于0.51，所预测结果存在信号肽 |
| Transmembrane |  | 预测方式，分为TM和noTM两种。TM为跨膜模式，noTM为非跨膜模式，signalP默认根据结果选用最好的预测方式 |

**跨膜蛋白预测详细信息**

- ID: 蛋白ID
- Length: 蛋白序列长度
- ExpAA: 跨膜螺旋氨基酸残基数量的期望值
- First60: 蛋白前60个氨基酸中跨膜螺旋的氨基酸量的期望值
- PredHel: 预测的跨膜螺旋数量
- Topology: 拓扑结构，o代表膜外，i代表膜内，中间的数字代表跨膜区的氨基酸

**TNSS效应蛋白预测**

效应蛋白是细菌分泌系统研究中的一个热点，病原菌通过分泌系统TNSS（type N secretion systems，I型-VII型）将效应蛋白分泌至胞外或是宿主细胞中，影响细胞进程中免疫应答反应、细胞衰亡等各种重要活动最终引起病理反应。革兰氏阴性菌的T3SS是研究比较多的分泌系统，其中效应蛋白（effector）是T3SS分泌系统发挥毒力的部分，与细菌的致病性密切相关，常用于分子水平病原菌的感染机制、毒力作用等研究。  
我们基于蛋白序列功能数据库（KEGG，Nr）注释结果，提取分泌系统TNSS（type N secretion systems，I型-VII型）相关蛋白。并采用EffectiveT3软件[]对革兰氏阴性菌进行T3SS效应蛋白的预测。

**TNSS系统效应蛋白注释结果**

- Gene ID：基因的编号
- Symbol：基因名称
- Type：分泌系统类型
- Nr：数据库描述
- Pathway ID: 基因所在KEGG代谢通路的编号及描述
- KO ID：KEGG对应的基因编号及描述

**双组分系统预测**

在分子生物学中，双组分系统（two-component system，TCSs，也称为双组分调控系统，two-component regulatory system）是机体识别和应对复杂环境变化的刺激-响应偶联机制，在原核、真核生物中都有发现，但值得注意的是，其在细菌中更为常见，最大的应激反应基因家族存在于细菌中。双组分系统的存在使细菌可以感受、应对、适应广泛的生存环境类型、环境压力，在渗透压、温度等的刺激响应，群体感应，趋化性，耐药性等方面有重要作用。原核生物中，双组分系统的基因数平均为30左右，占基因组1~2%的比例，有的物种甚至超过200个。  
双组分系统（two-component system，TCSs）是机体识别和应对复杂环境变化的刺激-响应偶联机制。双组分系统的存在使细菌可以感受、应对、适应广泛的生存环境类型、环境压力，在渗透压等的刺激响应、群体感应、趋化性、耐药性等方面有重要作用。基于双组分系统的特征，我们预测并绘制细菌基因组上的双组分系统。  
**双组分系统特征**  
“双组分”指“感受环境刺激的组分”和“作出应答响应的组分”，对应两个基因，基因编码的蛋白上有高度保守的结构域。其中感受刺激由组氨酸激酶受体结构域（ HATPase\_c domain）执行，应答响应由响应调节蛋白结构域（ Response\_reg domain）执行 [文献依据：Tierney AR, Rather PN. Roles of two-component regulatory systems in antibiotic resistance. Future Microbiol. 2019;14(6):533‐552. doi:10.2217/fmb-2019-0002]。大部分情况，两个基因在基因组上位置相邻。  
由于很多感受外界刺激的蛋白位于细胞膜上，所以有些双组分系统的蛋白含有跨膜螺旋结构域（Transmembrane\_Helix）。有些双组分系统还含有磷酸受体结构域（HisKA），可辅助信号传递。  

**预测步骤：**
首先基于Pfam注释结果，寻找含HATPase\_c和Response\_reg结构域的基因。然后挑选含两种结构域且在基因组上位置相邻的基因，作为潜在的TCSs。根据结构域位点信息绘制基因结构示意图，如果基因上含有跨膜螺旋和磷酸受体，则一起展示。  

**双组分系统预测结果表说明**

- GeneID\_HK: HK 基因ID
- Transmembrane\_Helix: 跨膜螺旋位置
- HisKA: HisKA结构域位置
- HATPase\_c: HATPase\_c结构域位置
- GeneID\_RR: 应答调节蛋白ID
- Response\_reg: Response\_reg结构域位置

**双组分系统示意图**

- HisKA（蓝色）: 磷酸受体结构域, 该区域含有一个保守组氨酸位点的 H-box, 能够自身磷酸化
- HATPase\_c（红色）: 位于胞内, 是组氨酸激酶的催化结构域, 主要负责把 ATP 的磷酸基团转移到组氨酸上
- Response\_reg（绿色）: Response regulator(RR), 应答调节蛋白
- 黑色模块：跨膜螺旋
- 两端编号：基因ID

**CARD注释**

CARD（Comprehensive Antibiotic Resistance Database）和ARDB（Antibiotic Resistance Genes Database）是两个使用最为广泛的细菌耐药基因数据库。但是ARDB数据库在2009年后已经停止更新。CARD它包含了ARDB数据库中所有的抗性信息，同时更新频率较高。CARD以Antibiotic Resistance Ontology（ARO）为分类单位（term）构建，用于关联抗生素模块及其目标、抗性机制、基因变异等信息。通过该数据库的注释，可以找到耐药性相关基因的名称，所耐受的抗生素种类等信息。

**CARD注释表**

- ORF ID：基因序列编号
- Cut off：RGI的筛选方法，有Perfect、Strict、和Loose三种
- PASS\_EVALUE：比对E值，越低表示可靠性越高
- Best\_Hit\_evalue：最佳匹配结果对应的E值
- Best\_Hit\_ARO：最佳匹配的AOR编号
- Best\_Identities：最佳匹配的序列相似度
- ARO：通过过滤阈值的所有ARO的ID
- ARO name：通过过滤阈值的所有ARO注释
- Model type：RGI预测类型，分为protein variant与protein homolog两种
- SNP：预测相比注释结果所对应的蛋白变异位点
- Best hit ARO category：最佳注释ARO所属抗性基因分类
- ARO category：所有通过阈值的ARO所属抗性基因分类

**VFDB 注释**

VFDB，Virulence Factors of Pathogenic Bacteria，毒力因子数据库，用于专门研究致病细菌、衣原体和支原体致病因子的数据库。包含24个种，共425个致病因子，24个致病岛，2359个与毒力因子的组成、结构、功能、致病机理和基因组等信息。毒力因子数据库能够为病原菌的入侵机理研究提供关键证据，同时结合比较基因组学等分析手段，可以从全基因组水平研究同属不同病原菌的毒力因子构成和基因组分布等特征。

**VFDB注释表**

- Query\_id：基因序列编号
- Subject\_id：比对到数据库中基因编号
- Identity：比对的identity
- Align\_length：比对上的长度
- Miss\_match：比对的错配数
- Gap：Gap的比例
- Query\_start：比对上的部分在基因上的起始位置
- Query\_end：比对上的部分在基因上的终止位置
- Subject\_start：比对上的部分在数据库序列上的起始位置
- Subject\_end：比对上的部分在数据库序列上的终止位置
- E\_value：比对的Evalue
- Score：比对的分值
- Align length：比对上的长度
- VFs：毒力因子
- Description：毒力因子的描述信息
- Organism：比对上的序列所在物种
- VFDB ID：注释到的毒力因子编号

#### 次级代谢基因簇预测

        一般情况下，参与次级代谢途径中生物合成酶的基因在染色体上成簇排列。基于指定类型的profile hidden Markov models, antismash 能准确鉴定所有已知的次级代谢基因簇。在antismash中，将次级代谢基因簇分为24类。最常见的次级代谢基因簇是type I polyketides synthase(T1pks)，type II polyketides synthase(T2pks)，type III polyketides synthase(T3pks)和non-ribosomal peptides synthase(NRPS)。例如：四环素、大环内酯类、安莎类、聚醚类由PKS途径合成；beta-内酰胺类、多肽类、糖肽类由NRPS途径合成。

#### 次级代谢产物基因簇预测结果

|  |  |  |
| --- | --- | --- |
| Cluster |  | 基因簇编号 |
| Contig |  | Contig ID |
| Gene cluster Type |  | 基因簇类型 |
| Gene cluster Genes |  | 基因簇包含的基因 |

**根据不同数据库/功能，我们采用不同软件进行功能注释：**

|  |  |  |  |
| --- | --- | --- | --- |
| 数据库/功能 | 作用 | 软件 | 参数 |
| Nr | 蛋白注释 | Blastp | evalue<=1e-5 |
| SwissProt | 蛋白注释 | Diamond | evalue<=1e-5 |
| KOG | 同源蛋白注释 | Diamond | evalue<=1e-5 |
| KEGG | 代谢通路相关蛋白 | Diamond | evalue<=1e-5 |
| GO | GO term注释 | Blast2GO | 默认 |
| Pfam | 蛋白结构域 | Pfam\_Scan | 默认 |
| CAZy | 碳水化合物酶注释 | Blastp | evalue<=1e-5 |
| PHI | 病原与宿主互作蛋白 | Blastp | evalue<=1e-5 |
| SignalP | 信号肽 | SignalP | 默认 |
| 次生代谢基因簇 | 次生代谢基因簇 | Antismash | 默认 |
