# Supplementary material for: Isolation, identification, and whole-genome sequencing of high-yield protease bacteria from Daqu of ZhangGong Laojiu
Source: PLoS One. 2022 Apr 26;17(4):e0264677. doi: 10.1371/journal.pone.0264677 (PMC9041807; doi:10.1371/journal.pone.0264677)
Supplement: S1 Raw data — (ZIP) [file pone.0264677.s002.zip › Raw data/GDD20120317-1_Bacillus_velezensis_Genome_result/src/doc/seq_stat.html]

»ùµÏ°ÂÉúÎï WGCNA¸»¼¯·ÖÎö °ïÖúÎÄµµ


### ²âÐòÆÀ¹À

²âÐòµÃµ½µÄÔ­Ê¼Í¼ÏñÊý¾Ý¾­ base calling ×ª»¯ÎªÐòÁÐÊý¾Ý£¬ÎÒÃÇ³ÆÖ®Îª raw data »ò raw reads£¬½á¹ûÒÔ FASTQ ÎÄ¼þ¸ñÊ½´æ´¢£¬°üº¬ reads µÄÐòÁÐÒÔ¼°¼î»ùµÄ²âÐòÖÊÁ¿¡£ÔÚ FASTQ ¸ñÊ½ÎÄ¼þÖÐÃ¿¸ö read ÓÉËÄÐÐÃèÊö£¬ÈçÏÂ£º

```
@A80GVTABXX:4:1:2587:1979#ACAGTGAT/1
NTTTGATATGTGTGAGGACGTCTGCAGCGTCACCTTTATCGGCCATGGT
+
BTTMKZXUUUdddddddddddddddddddddddddddadddddd^WYYU
			
```

Ã¿¸öÐòÁÐ¹²ÓÐ4ÐÐ£¬µÚ1ÐÐºÍµÚ3ÐÐÊÇÐòÁÐÃû³Æ£¨ÓÐµÄfqÎÄ¼þÎªÁË½ÚÊ¡´æ´¢¿Õ¼ä»áÊ¡ÂÔµÚÈýÐÐ¡°£«¡±ºóÃæµÄÐòÁÐÃû³Æ£©£¬ÓÉ²âÐòÒÇ²úÉú£»µÚ2ÐÐÊÇÐòÁÐ£»µÚ4ÐÐÊÇÐòÁÐµÄ²âÐòÖÊÁ¿£¬Ã¿¸ö×Ö·û¶ÔÓ¦µÚ2ÐÐÃ¿¸ö¼î»ù£¬µÚËÄÐÐÃ¿¸ö×Ö·û¶ÔÓ¦µÄASCIIÖµ¼õÈ¥64£¬¼´Îª¸Ã¼î»ùµÄ²âÐòÖÊÁ¿Öµ£¬±ÈÈçc¶ÔÓ¦µÄASCIIÖµÎª99£¬ÄÇÃ´Æä¶ÔÓ¦µÄ¼î»ùÖÊÁ¿ÖµÊÇ35¡£´ÓIllumina GA Pipeline v1.5¿ªÊ¼£¬¼î»ùÖÊÁ¿Öµ·¶Î§Îª2µ½41¡£ÏÂ±íÎªIllumina HiSeq^TM^ 2500²âÐò´íÎóÂÊÓë²âÐòÖÊÁ¿Öµ¼òÃ÷¶ÔÓ¦¹ØÏµ¡£Èç¹û²âÐò´íÎóÂÊÓÃE±íÊ¾£¬¼î»ùÖÊÁ¿ÖµÓÃSQ±íÊ¾£¬ÔòÓÐÏÂÁÐ¹ØÏµ£º

Illumina HiSeq^TM^ 2000 ²âÐò´íÎóÂÊÓë²âÐòÖÊÁ¿Öµ¼òÃ÷¶ÔÓ¦¹ØÏµ

| ²âÐò´íÎóÂÊ | ²âÐòÖÊÁ¿ | ¶ÔÓ¦×Ö·û |
| --- | --- | --- |
| 1% | 20 | T |
| 0.1% | 30 | ^ |
| 0.01% | 40 | h |

##### ²âÐòÖÊÁ¿ÆÀ¹À

|  |  |
| --- | --- |
|  | ×óÍ¼ÎªÑùÆ· Test ÖÐ¸÷Àà reads ËùÕ¼±ÈÀý·Ö²¼µÄÇé¿ö¡£  - ¡°Adaptor¡± ±íÊ¾Ö»º¬ÓÐ½ÓÍ·ÐòÁÐµÄ reads ÊýÕ¼×Ü reads ÊýµÄ±ÈÀý - ¡°High\_N\_rate¡± ±íÊ¾º¬Î´Öª¼î»ùµÄ reads ÊýÕ¼ reads ×ÜÊýµÄ±ÈÀý - ¡°Low\_quality¡± ±íÊ¾µÍÖÊÁ¿µÄ reads ÊýÕ¼ reads ×ÜÊýµÄ±ÈÀý - ¡°High\_quality\_clean¡± ÊÇÔ­Ê¼ÐòÁÐÊý¾Ý¾­¹ýÈ¥³ýÔÓÖÊºóµÃµ½µÄÊý¾ÝÕ¼ reads ×ÜÊýµÄ±ÈÀý |

##### Êý¾Ý¹ýÂË

ÎªÁË±£Ö¤Êý¾ÝÖÊÁ¿£¬ÒªÔÚÐÅÏ¢·ÖÎöÇ°¶ÔÔ­Ê¼Êý¾Ý½øÐÐÖÊ¿Ø£¬²¢ÇÒÍ¨¹ýÊý¾Ý¹ýÂËÀ´¼õÉÙÊý¾ÝÔëÒô¡£ÎÒÃÇ¶ÔÏÂ»úµÄ clean reads ÔÙ½øÐÐ¸üÑÏ¸ñµÄ¹ýÂË£¬µÃµ½ High quality clean reads£¬ÓÃÓÚºóÐøµÄÐÅÏ¢·ÖÎö¡£¹ýÂËµÄ²½ÖèÈçÏÂ:

1. È¥³ýº¬ adapter µÄ reads
2. È¥³ýº¬ N ±ÈÀý´óÓÚ 10% µÄreads
3. È¥³ýµÍÖÊÁ¿ reads (ÖÊÁ¿Öµ Q¡Ü10 µÄ¼î»ùÊýÕ¼ÕûÌõ read µÄ 40£¥ ÒÔÉÏ)

##### ¼î»ù×é³ÉÓëÖÊÁ¿·ÖÎö

|  |  |
| --- | --- |
|  | ÎÒÃÇÒ²»á¶Ô¹ýÂËºóµÄÊý¾Ý»­¼î»ù×é³ÉºÍÖÊÁ¿Öµ·Ö²¼Í¼£¬À´Ö±¹ÛµØ¿´Êý¾ÝÖÊÁ¿Çé¿ö¡£×óÍ¼ÖÐ£¬XÖáÉÏ£¬1-125bp ´ú±í read1 µÄ¼î»ùÎ»ÖÃ£¬126-250bp ´ú±í read2 µÄ¼î»ùÎ»ÖÃ¡£A¡¢T¡¢C¡¢GÇúÏß¸÷´ú±íÃ¿¸öÎ»ÖÃA¡¢T¡¢C¡¢G¼î»ùµÄ±ÈÀý£¬¼î»ù×é³ÉÆ½ºâµÄÇé¿öÏÂ£¬A¡¢T ÇúÏßÖØºÏ£¬G¡¢C ÇúÏßÖØºÏ¡£Èç¹û²âÐòÖÐ³öÏÖ²»Õý³£µÄÇé¿ö£¬¼î»ù×é³É¾Í¿ÉÄÜ²»Æ½ºâ¡£N ÇúÏß´ú±íÃ¿¸öÎ»ÖÃÎ´±»²âµ½µÄ¼î»ùµÄ±ÈÀý¡£mean ÇúÏß±íÊ¾¼î»ùÔÚÃ¿¸öÎ»ÖÃµÄÆ½¾ùÖÊÁ¿¡£Q20ºÍQ30ÇúÏß·Ö±ð´ú±í¼î»ùÔÚÃ¿¸öÎ»ÖÃµÄÖÊÁ¿Öµ>=Q20»òÕß>=Q30µÄ¼î»ù±ÈÀý¡£ |

##### ¹ýÂËÐÅÏ¢Í³¼Æ

¹ýÂËÇ°ºó¼î»ùÐÅÏ¢Í³¼Æ±íËµÃ÷

- Clean Data (PE) = Clean Reads1 Num x Read1 length + Clean Reads2 Num x Read2 length
- Clean Data (SE) = Clean Reads1 Num x Read1 length
- HQ Clean Data (PE) = HQ Clean Reads1 Num x Read1 length + HQ Clean Reads2 Num x Read2 length
- HQ Clean Data (SE) = HQ Clean Reads1 Num x Read1 length

Reads ¹ýÂËÐÅÏ¢Í³¼Æ±íËµÃ÷

- Clean Reads Num (PE) = Clean Reads1 + Clean Reads2
- HQ Clean Reads Num (PE) = HQ Clean Reads1 + HQ Clean Reads2
- Clean Reads Num (SE) = Clean Reads1
- HQ Clean Reads Num (SE) = HQ Clean Reads1
- Adapter £ºClean reads ÖÐº¬ÓÐ adapter µÄ reads ÊýÄ¿
- Low Quality £ºClean reads ÖÐº¬ÓÐµÍÖÊÁ¿¼î»ù´ïµ½Ò»¶¨³Ì¶ÈµÄ reads ÊýÄ¿
- Poly A £ºClean reads ÖÐº¬ÓÐ poly A µÄ reads ÊýÄ¿
- N £ºClean reads ÖÐº¬ÓÐ N ´ïµ½Ò»¶¨³Ì¶ÈµÄ reads ÊýÄ¿
