# Supplementary material for: Isolation, identification, and whole-genome sequencing of high-yield protease bacteria from Daqu of ZhangGong Laojiu
Source: PLoS One. 2022 Apr 26;17(4):e0264677. doi: 10.1371/journal.pone.0264677 (PMC9041807; doi:10.1371/journal.pone.0264677)
Supplement: S1 Raw data — (ZIP) [file pone.0264677.s002.zip › Raw data/GDD20120317-1_Bacillus_velezensis_Genome_result/src/js/jquery.nicescroll.js]

jquery.nicescroll/jquery.nicescroll.js at master · inuyaksa/jquery.nicescroll · GitHub


Skip to content

Sign up
Sign in

This repository

- Explore
- Features
- Enterprise
- Pricing

- Watch

  151
- Star

  1,805
- Fork

  686

# inuyaksa/**jquery.nicescroll**

- Code
- Issues
- Pull requests
- Wiki

- Pulse
- Graphs

### HTTPS clone URL

### Subversion checkout URL

You can clone with

HTTPS or 

Subversion.

Download ZIP

Permalink


*Branch:*
master

Switch branches/tags

- Branches
- Tags

Bleeding-edge


master

Nothing to show

3.6.0

3.5.6

Nothing to show

jquery.nicescroll/**jquery.nicescroll.js**

af737e0
Nov 21, 2014

inuyaksa
3.6.0

**9**
contributors

## Users who have contributed to this file

- inuyaksa
- GianlucaGuarini
- brandonaaron
- nasaorc
- levinalex
- smbeiragh
- eocanha
- yukulele
- zcourts

Raw
Blame
History

3634 lines (3068 sloc)
119 KB

|  |  |
| --- | --- |
|  | /\* jquery.nicescroll |
|  | -- version 3.6.0 |
|  | -- copyright 2014-11-21 InuYaksa\*2014 |
|  | -- licensed under the MIT |
|  | -- |
|  | -- http://nicescroll.areaaperta.com/ |
|  | -- https://github.com/inuyaksa/jquery.nicescroll |
|  | -- |
|  | \*/ |
|  |  |
|  | (function(factory) { |
|  | if (typeof define === 'function' && define.amd) { |
|  | // AMD. Register as anonymous module. |
|  | define(['jquery'], factory); |
|  | } else { |
|  | // Browser globals. |
|  | factory(jQuery); |
|  | } |
|  | }(function(jQuery) { |
|  | "use strict"; |
|  |  |
|  | // globals |
|  | var domfocus = false; |
|  | var mousefocus = false; |
|  | var tabindexcounter = 0; |
|  | var ascrailcounter = 2000; |
|  | var globalmaxzindex = 0; |
|  |  |
|  | var $ = jQuery; // sandbox |
|  |  |
|  | // http://stackoverflow.com/questions/2161159/get-script-path |
|  | function getScriptPath() { |
|  | var scripts = document.getElementsByTagName('script'); |
|  | var path = scripts[scripts.length - 1].src.split('?')[0]; |
|  | return (path.split('/').length > 0) ? path.split('/').slice(0, -1).join('/') + '/' : ''; |
|  | } |
|  |  |
|  | var vendors = ['webkit','ms','moz','o']; |
|  |  |
|  | var setAnimationFrame = window.requestAnimationFrame \|\| false; |
|  | var clearAnimationFrame = window.cancelAnimationFrame \|\| false; |
|  |  |
|  | if (!setAnimationFrame) { // legacy detection |
|  | for (var vx in vendors) { |
|  | var v = vendors[vx]; |
|  | if (!setAnimationFrame) setAnimationFrame = window[v + 'RequestAnimationFrame']; |
|  | if (!clearAnimationFrame) clearAnimationFrame = window[v + 'CancelAnimationFrame'] \|\| window[v + 'CancelRequestAnimationFrame']; |
|  | } |
|  | } |
|  |  |
|  | var ClsMutationObserver = window.MutationObserver \|\| window.WebKitMutationObserver \|\| false; |
|  |  |
|  | var \_globaloptions = { |
|  | zindex: "auto", |
|  | cursoropacitymin: 0, |
|  | cursoropacitymax: 1, |
|  | cursorcolor: "#424242", |
|  | cursorwidth: "5px", |
|  | cursorborder: "1px solid #fff", |
|  | cursorborderradius: "5px", |
|  | scrollspeed: 60, |
|  | mousescrollstep: 8 \* 3, |
|  | touchbehavior: false, |
|  | hwacceleration: true, |
|  | usetransition: true, |
|  | boxzoom: false, |
|  | dblclickzoom: true, |
|  | gesturezoom: true, |
|  | grabcursorenabled: true, |
|  | autohidemode: true, |
|  | background: "", |
|  | iframeautoresize: true, |
|  | cursorminheight: 32, |
|  | preservenativescrolling: true, |
|  | railoffset: false, |
|  | railhoffset: false, |
|  | bouncescroll: true, |
|  | spacebarenabled: true, |
|  | railpadding: { |
|  | top: 0, |
|  | right: 0, |
|  | left: 0, |
|  | bottom: 0 |
|  | }, |
|  | disableoutline: true, |
|  | horizrailenabled: true, |
|  | railalign: "right", |
|  | railvalign: "bottom", |
|  | enabletranslate3d: true, |
|  | enablemousewheel: true, |
|  | enablekeyboard: true, |
|  | smoothscroll: true, |
|  | sensitiverail: true, |
|  | enablemouselockapi: true, |
|  | // cursormaxheight:false, |
|  | cursorfixedheight: false, |
|  | directionlockdeadzone: 6, |
|  | hidecursordelay: 400, |
|  | nativeparentscrolling: true, |
|  | enablescrollonselection: true, |
|  | overflowx: true, |
|  | overflowy: true, |
|  | cursordragspeed: 0.3, |
|  | rtlmode: "auto", |
|  | cursordragontouch: false, |
|  | oneaxismousemode: "auto", |
|  | scriptpath: getScriptPath(), |
|  | preventmultitouchscrolling: true |
|  | }; |
|  |  |
|  | var browserdetected = false; |
|  |  |
|  | var getBrowserDetection = function() { |
|  |  |
|  | if (browserdetected) return browserdetected; |
|  |  |
|  | var \_el = document.createElement('DIV'), |
|  | \_style = \_el.style, |
|  | \_agent = navigator.userAgent, |
|  | \_platform = navigator.platform, |
|  | d = {}; |
|  |  |
|  | d.haspointerlock = "pointerLockElement" in document \|\| "webkitPointerLockElement" in document \|\| "mozPointerLockElement" in document; |
|  |  |
|  | d.isopera = ("opera" in window); // 12- |
|  | d.isopera12 = (d.isopera && ("getUserMedia" in navigator)); |
|  | d.isoperamini = (Object.prototype.toString.call(window.operamini) === "[object OperaMini]"); |
|  |  |
|  | d.isie = (("all" in document) && ("attachEvent" in \_el) && !d.isopera); //IE10- |
|  | d.isieold = (d.isie && !("msInterpolationMode" in \_style)); // IE6 and older |
|  | d.isie7 = d.isie && !d.isieold && (!("documentMode" in document) \|\| (document.documentMode == 7)); |
|  | d.isie8 = d.isie && ("documentMode" in document) && (document.documentMode == 8); |
|  | d.isie9 = d.isie && ("performance" in window) && (document.documentMode >= 9); |
|  | d.isie10 = d.isie && ("performance" in window) && (document.documentMode == 10); |
|  | d.isie11 = ("msRequestFullscreen" in \_el) && (document.documentMode >= 11); // IE11+ |
|  |  |
|  | d.isie9mobile = /iemobile.9/i.test(\_agent); //wp 7.1 mango |
|  | if (d.isie9mobile) d.isie9 = false; |
|  | d.isie7mobile = (!d.isie9mobile && d.isie7) && /iemobile/i.test(\_agent); //wp 7.0 |
|  |  |
|  | d.ismozilla = ("MozAppearance" in \_style); |
|  |  |
|  | d.iswebkit = ("WebkitAppearance" in \_style); |
|  |  |
|  | d.ischrome = ("chrome" in window); |
|  | d.ischrome22 = (d.ischrome && d.haspointerlock); |
|  | d.ischrome26 = (d.ischrome && ("transition" in \_style)); // issue with transform detection (maintain prefix) |
|  |  |
|  | d.cantouch = ("ontouchstart" in document.documentElement) \|\| ("ontouchstart" in window); // detection for Chrome Touch Emulation |
|  | d.hasmstouch = (window.MSPointerEvent \|\| false); // IE10 pointer events |
|  | d.hasw3ctouch = (window.PointerEvent \|\| false); //IE11 pointer events, following W3C Pointer Events spec |
|  |  |
|  | d.ismac = /^mac$/i.test(\_platform); |
|  |  |
|  | d.isios = (d.cantouch && /iphone\|ipad\|ipod/i.test(\_platform)); |
|  | d.isios4 = ((d.isios) && !("seal" in Object)); |
|  | d.isios7 = ((d.isios)&&("webkitHidden" in document)); //iOS 7+ |
|  |  |
|  | d.isandroid = (/android/i.test(\_agent)); |
|  |  |
|  | d.haseventlistener = ("addEventListener" in \_el); |
|  |  |
|  | d.trstyle = false; |
|  | d.hastransform = false; |
|  | d.hastranslate3d = false; |
|  | d.transitionstyle = false; |
|  | d.hastransition = false; |
|  | d.transitionend = false; |
|  |  |
|  | var a; |
|  | var check = ['transform', 'msTransform', 'webkitTransform', 'MozTransform', 'OTransform']; |
|  | for (a = 0; a < check.length; a++) { |
|  | if (typeof \_style[check[a]] != "undefined") { |
|  | d.trstyle = check[a]; |
|  | break; |
|  | } |
|  | } |
|  | d.hastransform = (!!d.trstyle); |
|  | if (d.hastransform) { |
|  | \_style[d.trstyle] = "translate3d(1px,2px,3px)"; |
|  | d.hastranslate3d = /translate3d/.test(\_style[d.trstyle]); |
|  | } |
|  |  |
|  | d.transitionstyle = false; |
|  | d.prefixstyle = ''; |
|  | d.transitionend = false; |
|  | check = ['transition', 'webkitTransition', 'msTransition', 'MozTransition', 'OTransition', 'OTransition', 'KhtmlTransition']; |
|  | var prefix = ['', '-webkit-', '-ms-', '-moz-', '-o-', '-o', '-khtml-']; |
|  | var evs = ['transitionend', 'webkitTransitionEnd', 'msTransitionEnd', 'transitionend', 'otransitionend', 'oTransitionEnd', 'KhtmlTransitionEnd']; |
|  | for (a = 0; a < check.length; a++) { |
|  | if (check[a] in \_style) { |
|  | d.transitionstyle = check[a]; |
|  | d.prefixstyle = prefix[a]; |
|  | d.transitionend = evs[a]; |
|  | break; |
|  | } |
|  | } |
|  | if (d.ischrome26) { // always use prefix |
|  | d.prefixstyle = prefix[1]; |
|  | } |
|  |  |
|  | d.hastransition = (d.transitionstyle); |
|  |  |
|  | function detectCursorGrab() { |
|  | var lst = ['-webkit-grab', '-moz-grab', 'grab']; |
|  | if ((d.ischrome && !d.ischrome22) \|\| d.isie) lst = []; // force setting for IE returns false positive and chrome cursor bug |
|  | for (var a = 0; a < lst.length; a++) { |
|  | var p = lst[a]; |
|  | \_style.cursor = p; |
|  | if (\_style.cursor == p) return p; |
|  | } |
|  | return 'url(//mail.google.com/mail/images/2/openhand.cur),n-resize'; // thank you google for custom cursor! |
|  | } |
|  | d.cursorgrabvalue = detectCursorGrab(); |
|  |  |
|  | d.hasmousecapture = ("setCapture" in \_el); |
|  |  |
|  | d.hasMutationObserver = (ClsMutationObserver !== false); |
|  |  |
|  | \_el = null; //memory released |
|  |  |
|  | browserdetected = d; |
|  |  |
|  | return d; |
|  | }; |
|  |  |
|  | var NiceScrollClass = function(myopt, me) { |
|  |  |
|  | var self = this; |
|  |  |
|  | this.version = '3.6.0'; |
|  | this.name = 'nicescroll'; |
|  |  |
|  | this.me = me; |
|  |  |
|  | this.opt = { |
|  | doc: $("body"), |
|  | win: false |
|  | }; |
|  |  |
|  | $.extend(this.opt, \_globaloptions); // clone opts |
|  |  |
|  | // Options for internal use |
|  | this.opt.snapbackspeed = 80; |
|  |  |
|  | if (myopt \|\| false) { |
|  | for (var a in self.opt) { |
|  | if (typeof myopt[a] != "undefined") self.opt[a] = myopt[a]; |
|  | } |
|  | } |
|  |  |
|  | this.doc = self.opt.doc; |
|  | this.iddoc = (this.doc && this.doc[0]) ? this.doc[0].id \|\| '' : ''; |
|  | this.ispage = /^BODY\|HTML/.test((self.opt.win) ? self.opt.win[0].nodeName : this.doc[0].nodeName); |
|  | this.haswrapper = (self.opt.win !== false); |
|  | this.win = self.opt.win \|\| (this.ispage ? $(window) : this.doc); |
|  | this.docscroll = (this.ispage && !this.haswrapper) ? $(window) : this.win; |
|  | this.body = $("body"); |
|  | this.viewport = false; |
|  |  |
|  | this.isfixed = false; |
|  |  |
|  | this.iframe = false; |
|  | this.isiframe = ((this.doc[0].nodeName == 'IFRAME') && (this.win[0].nodeName == 'IFRAME')); |
|  |  |
|  | this.istextarea = (this.win[0].nodeName == 'TEXTAREA'); |
|  |  |
|  | this.forcescreen = false; //force to use screen position on events |
|  |  |
|  | this.canshowonmouseevent = (self.opt.autohidemode != "scroll"); |
|  |  |
|  | // Events jump table |
|  | this.onmousedown = false; |
|  | this.onmouseup = false; |
|  | this.onmousemove = false; |
|  | this.onmousewheel = false; |
|  | this.onkeypress = false; |
|  | this.ongesturezoom = false; |
|  | this.onclick = false; |
|  |  |
|  | // Nicescroll custom events |
|  | this.onscrollstart = false; |
|  | this.onscrollend = false; |
|  | this.onscrollcancel = false; |
|  |  |
|  | this.onzoomin = false; |
|  | this.onzoomout = false; |
|  |  |
|  | // Let's start! |
|  | this.view = false; |
|  | this.page = false; |
|  |  |
|  | this.scroll = { |
|  | x: 0, |
|  | y: 0 |
|  | }; |
|  | this.scrollratio = { |
|  | x: 0, |
|  | y: 0 |
|  | }; |
|  | this.cursorheight = 20; |
|  | this.scrollvaluemax = 0; |
|  |  |
|  | this.isrtlmode = (this.opt.rtlmode == "auto") ? ((this.win[0] == window ? this.body : this.win).css("direction") == "rtl") : (this.opt.rtlmode === true); |
|  | // this.checkrtlmode = false; |
|  |  |
|  | this.scrollrunning = false; |
|  |  |
|  | this.scrollmom = false; |
|  |  |
|  | this.observer = false; // observer div changes |
|  | this.observerremover = false; // observer on parent for remove detection |
|  | this.observerbody = false; // observer on body for position change |
|  |  |
|  | do { |
|  | this.id = "ascrail" + (ascrailcounter++); |
|  | } while (document.getElementById(this.id)); |
|  |  |
|  | this.rail = false; |
|  | this.cursor = false; |
|  | this.cursorfreezed = false; |
|  | this.selectiondrag = false; |
|  |  |
|  | this.zoom = false; |
|  | this.zoomactive = false; |
|  |  |
|  | this.hasfocus = false; |
|  | this.hasmousefocus = false; |
|  |  |
|  | this.visibility = true; |
|  | this.railslocked = false; // locked by resize |
|  | this.locked = false; // prevent lost of locked status sets by user |
|  | this.hidden = false; // rails always hidden |
|  | this.cursoractive = true; // user can interact with cursors |
|  |  |
|  | this.wheelprevented = false; //prevent mousewheel event |
|  |  |
|  | this.overflowx = self.opt.overflowx; |
|  | this.overflowy = self.opt.overflowy; |
|  |  |
|  | this.nativescrollingarea = false; |
|  | this.checkarea = 0; |
|  |  |
|  | this.events = []; // event list for unbind |
|  |  |
|  | this.saved = {}; // style saved |
|  |  |
|  | this.delaylist = {}; |
|  | this.synclist = {}; |
|  |  |
|  | this.lastdeltax = 0; |
|  | this.lastdeltay = 0; |
|  |  |
|  | this.detected = getBrowserDetection(); |
|  |  |
|  | var cap = $.extend({}, this.detected); |
|  |  |
|  | this.canhwscroll = (cap.hastransform && self.opt.hwacceleration); |
|  | this.ishwscroll = (this.canhwscroll && self.haswrapper); |
|  |  |
|  | this.hasreversehr = (this.isrtlmode&&!cap.iswebkit); //RTL mode with reverse horizontal axis |
|  |  |
|  | this.istouchcapable = false; // desktop devices with touch screen support |
|  |  |
|  | //## Check WebKit-based desktop with touch support |
|  | //## + Firefox 18 nightly build (desktop) false positive (or desktop with touch support) |
|  | if (cap.cantouch && !cap.isios && !cap.isandroid && (cap.iswebkit \|\| cap.ismozilla)) { |
|  | this.istouchcapable = true; |
|  | cap.cantouch = false; // parse normal desktop events |
|  | } |
|  |  |
|  | //## disable MouseLock API on user request |
|  | if (!self.opt.enablemouselockapi) { |
|  | cap.hasmousecapture = false; |
|  | cap.haspointerlock = false; |
|  | } |
|  |  |
|  | /\* deprecated |
|  | this.delayed = function(name, fn, tm, lazy) { |
|  | }; |
|  | \*/ |
|  |  |
|  | this.debounced = function(name, fn, tm) { |
|  | var dd = self.delaylist[name]; |
|  | self.delaylist[name] = fn; |
|  | if (!dd) { |
|  | setTimeout(function() { |
|  | var fn = self.delaylist[name]; |
|  | self.delaylist[name] = false; |
|  | fn.call(self); |
|  | }, tm); |
|  | } |
|  | }; |
|  |  |
|  | var \_onsync = false; |
|  |  |
|  | this.synched = function(name, fn) { |
|  |  |
|  | function requestSync() { |
|  | if (\_onsync) return; |
|  | setAnimationFrame(function() { |
|  | \_onsync = false; |
|  | for (var nn in self.synclist) { |
|  | var fn = self.synclist[nn]; |
|  | if (fn) fn.call(self); |
|  | self.synclist[nn] = false; |
|  | } |
|  | }); |
|  | \_onsync = true; |
|  | } |
|  |  |
|  | self.synclist[name] = fn; |
|  | requestSync(); |
|  | return name; |
|  | }; |
|  |  |
|  | this.unsynched = function(name) { |
|  | if (self.synclist[name]) self.synclist[name] = false; |
|  | }; |
|  |  |
|  | this.css = function(el, pars) { // save & set |
|  | for (var n in pars) { |
|  | self.saved.css.push([el, n, el.css(n)]); |
|  | el.css(n, pars[n]); |
|  | } |
|  | }; |
|  |  |
|  | this.scrollTop = function(val) { |
|  | return (typeof val == "undefined") ? self.getScrollTop() : self.setScrollTop(val); |
|  | }; |
|  |  |
|  | this.scrollLeft = function(val) { |
|  | return (typeof val == "undefined") ? self.getScrollLeft() : self.setScrollLeft(val); |
|  | }; |
|  |  |
|  | // derived by by Dan Pupius www.pupius.net |
|  | var BezierClass = function(st, ed, spd, p1, p2, p3, p4) { |
|  |  |
|  | this.st = st; |
|  | this.ed = ed; |
|  | this.spd = spd; |
|  |  |
|  | this.p1 = p1 \|\| 0; |
|  | this.p2 = p2 \|\| 1; |
|  | this.p3 = p3 \|\| 0; |
|  | this.p4 = p4 \|\| 1; |
|  |  |
|  | this.ts = (new Date()).getTime(); |
|  | this.df = this.ed - this.st; |
|  | }; |
|  | BezierClass.prototype = { |
|  | B2: function(t) { |
|  | return 3 \* t \* t \* (1 - t); |
|  | }, |
|  | B3: function(t) { |
|  | return 3 \* t \* (1 - t) \* (1 - t); |
|  | }, |
|  | B4: function(t) { |
|  | return (1 - t) \* (1 - t) \* (1 - t); |
|  | }, |
|  | getNow: function() { |
|  | var nw = (new Date()).getTime(); |
|  | var pc = 1 - ((nw - this.ts) / this.spd); |
|  | var bz = this.B2(pc) + this.B3(pc) + this.B4(pc); |
|  | return (pc < 0) ? this.ed : this.st + Math.round(this.df \* bz); |
|  | }, |
|  | update: function(ed, spd) { |
|  | this.st = this.getNow(); |
|  | this.ed = ed; |
|  | this.spd = spd; |
|  | this.ts = (new Date()).getTime(); |
|  | this.df = this.ed - this.st; |
|  | return this; |
|  | } |
|  | }; |
|  |  |
|  | //derived from http://stackoverflow.com/questions/11236090/ |
|  | function getMatrixValues() { |
|  | var tr = self.doc.css(cap.trstyle); |
|  | if (tr && (tr.substr(0, 6) == "matrix")) { |
|  | return tr.replace(/^.\*\((.\*)\)$/g, "$1").replace(/px/g, '').split(/, +/); |
|  | } |
|  | return false; |
|  | } |
|  |  |
|  | if (this.ishwscroll) { |
|  | // hw accelerated scroll |
|  | this.doc.translate = { |
|  | x: 0, |
|  | y: 0, |
|  | tx: "0px", |
|  | ty: "0px" |
|  | }; |
|  |  |
|  | //this one can help to enable hw accel on ios6 http://indiegamr.com/ios6-html-hardware-acceleration-changes-and-how-to-fix-them/ |
|  | if (cap.hastranslate3d && cap.isios) this.doc.css("-webkit-backface-visibility", "hidden"); // prevent flickering http://stackoverflow.com/questions/3461441/ |
|  |  |
|  | this.getScrollTop = function(last) { |
|  | if (!last) { |
|  | var mtx = getMatrixValues(); |
|  | if (mtx) return (mtx.length == 16) ? -mtx[13] : -mtx[5]; //matrix3d 16 on IE10 |
|  | if (self.timerscroll && self.timerscroll.bz) return self.timerscroll.bz.getNow(); |
|  | } |
|  | return self.doc.translate.y; |
|  | }; |
|  |  |
|  | this.getScrollLeft = function(last) { |
|  | if (!last) { |
|  | var mtx = getMatrixValues(); |
|  | if (mtx) return (mtx.length == 16) ? -mtx[12] : -mtx[4]; //matrix3d 16 on IE10 |
|  | if (self.timerscroll && self.timerscroll.bh) return self.timerscroll.bh.getNow(); |
|  | } |
|  | return self.doc.translate.x; |
|  | }; |
|  |  |
|  | this.notifyScrollEvent = function(el) { |
|  | var e = document.createEvent("UIEvents"); |
|  | e.initUIEvent("scroll", false, true, window, 1); |
|  | e.niceevent = true; |
|  | el.dispatchEvent(e); |
|  | }; |
|  |  |
|  | var cxscrollleft = (this.isrtlmode) ? 1 : -1; |
|  |  |
|  | if (cap.hastranslate3d && self.opt.enabletranslate3d) { |
|  | this.setScrollTop = function(val, silent) { |
|  | self.doc.translate.y = val; |
|  | self.doc.translate.ty = (val \* -1) + "px"; |
|  | self.doc.css(cap.trstyle, "translate3d(" + self.doc.translate.tx + "," + self.doc.translate.ty + ",0px)"); |
|  | if (!silent) self.notifyScrollEvent(self.win[0]); |
|  | }; |
|  | this.setScrollLeft = function(val, silent) { |
|  | self.doc.translate.x = val; |
|  | self.doc.translate.tx = (val \* cxscrollleft) + "px"; |
|  | self.doc.css(cap.trstyle, "translate3d(" + self.doc.translate.tx + "," + self.doc.translate.ty + ",0px)"); |
|  | if (!silent) self.notifyScrollEvent(self.win[0]); |
|  | }; |
|  | } else { |
|  | this.setScrollTop = function(val, silent) { |
|  | self.doc.translate.y = val; |
|  | self.doc.translate.ty = (val \* -1) + "px"; |
|  | self.doc.css(cap.trstyle, "translate(" + self.doc.translate.tx + "," + self.doc.translate.ty + ")"); |
|  | if (!silent) self.notifyScrollEvent(self.win[0]); |
|  | }; |
|  | this.setScrollLeft = function(val, silent) { |
|  | self.doc.translate.x = val; |
|  | self.doc.translate.tx = (val \* cxscrollleft) + "px"; |
|  | self.doc.css(cap.trstyle, "translate(" + self.doc.translate.tx + "," + self.doc.translate.ty + ")"); |
|  | if (!silent) self.notifyScrollEvent(self.win[0]); |
|  | }; |
|  | } |
|  | } else { |
|  | // native scroll |
|  | this.getScrollTop = function() { |
|  | return self.docscroll.scrollTop(); |
|  | }; |
|  | this.setScrollTop = function(val) { |
|  | return self.docscroll.scrollTop(val); |
|  | }; |
|  | this.getScrollLeft = function() { |
|  | if (self.detected.ismozilla && self.isrtlmode) |
|  | return Math.abs(self.docscroll.scrollLeft()); |
|  | return self.docscroll.scrollLeft(); |
|  | }; |
|  | this.setScrollLeft = function(val) { |
|  | return self.docscroll.scrollLeft((self.detected.ismozilla && self.isrtlmode) ? -val : val); |
|  | }; |
|  | } |
|  |  |
|  | this.getTarget = function(e) { |
|  | if (!e) return false; |
|  | if (e.target) return e.target; |
|  | if (e.srcElement) return e.srcElement; |
|  | return false; |
|  | }; |
|  |  |
|  | this.hasParent = function(e, id) { |
|  | if (!e) return false; |
|  | var el = e.target \|\| e.srcElement \|\| e \|\| false; |
|  | while (el && el.id != id) { |
|  | el = el.parentNode \|\| false; |
|  | } |
|  | return (el !== false); |
|  | }; |
|  |  |
|  | function getZIndex() { |
|  | var dom = self.win; |
|  | if ("zIndex" in dom) return dom.zIndex(); // use jQuery UI method when available |
|  | while (dom.length > 0) { |
|  | if (dom[0].nodeType == 9) return false; |
|  | var zi = dom.css('zIndex'); |
|  | if (!isNaN(zi) && zi != 0) return parseInt(zi); |
|  | dom = dom.parent(); |
|  | } |
|  | return false; |
|  | } |
|  |  |
|  | //inspired by http://forum.jquery.com/topic/width-includes-border-width-when-set-to-thin-medium-thick-in-ie |
|  | var \_convertBorderWidth = { |
|  | "thin": 1, |
|  | "medium": 3, |
|  | "thick": 5 |
|  | }; |
|  |  |
|  | function getWidthToPixel(dom, prop, chkheight) { |
|  | var wd = dom.css(prop); |
|  | var px = parseFloat(wd); |
|  | if (isNaN(px)) { |
|  | px = \_convertBorderWidth[wd] \|\| 0; |
|  | var brd = (px == 3) ? ((chkheight) ? (self.win.outerHeight() - self.win.innerHeight()) : (self.win.outerWidth() - self.win.innerWidth())) : 1; //DON'T TRUST CSS |
|  | if (self.isie8 && px) px += 1; |
|  | return (brd) ? px : 0; |
|  | } |
|  | return px; |
|  | } |
|  |  |
|  | this.getDocumentScrollOffset = function() { |
|  | return {top:window.pageYOffset\|\|document.documentElement.scrollTop, |
|  | left:window.pageXOffset\|\|document.documentElement.scrollLeft}; |
|  | } |
|  |  |
|  | this.getOffset = function() { |
|  | if (self.isfixed) { |
|  | var ofs = self.win.offset(); // fix Chrome auto issue (when right/bottom props only) |
|  | var scrl = self.getDocumentScrollOffset(); |
|  | ofs.top-=scrl.top; |
|  | ofs.left-=scrl.left; |
|  | return ofs; |
|  | } |
|  | var ww = self.win.offset(); |
|  | if (!self.viewport) return ww; |
|  | var vp = self.viewport.offset(); |
|  | return { |
|  | top: ww.top - vp.top,// + self.viewport.scrollTop(), |
|  | left: ww.left - vp.left // + self.viewport.scrollLeft() |
|  | }; |
|  | }; |
|  |  |
|  | this.updateScrollBar = function(len) { |
|  | if (self.ishwscroll) { |
|  | self.rail.css({ //\*\* |
|  | height: self.win.innerHeight() - (self.opt.railpadding.top + self.opt.railpadding.bottom) |
|  | }); |
|  | if (self.railh) self.railh.css({ //\*\* |
|  | width: self.win.innerWidth() - (self.opt.railpadding.left + self.opt.railpadding.right) |
|  | }); |
|  |  |
|  | } else { |
|  | var wpos = self.getOffset(); |
|  | var pos = { |
|  | top: wpos.top, |
|  | left: wpos.left - (self.opt.railpadding.left + self.opt.railpadding.right) |
|  | }; |
|  | pos.top += getWidthToPixel(self.win, 'border-top-width', true); |
|  | pos.left += (self.rail.align) ? self.win.outerWidth() - getWidthToPixel(self.win, 'border-right-width') - self.rail.width : getWidthToPixel(self.win, 'border-left-width'); |
|  |  |
|  | var off = self.opt.railoffset; |
|  | if (off) { |
|  | if (off.top) pos.top += off.top; |
|  | if (self.rail.align && off.left) pos.left += off.left; |
|  | } |
|  |  |
|  | if (!self.railslocked) self.rail.css({ |
|  | top: pos.top, |
|  | left: pos.left, |
|  | height: ((len) ? len.h : self.win.innerHeight()) - (self.opt.railpadding.top + self.opt.railpadding.bottom) |
|  | }); |
|  |  |
|  | if (self.zoom) { |
|  | self.zoom.css({ |
|  | top: pos.top + 1, |
|  | left: (self.rail.align == 1) ? pos.left - 20 : pos.left + self.rail.width + 4 |
|  | }); |
|  | } |
|  |  |
|  | if (self.railh && !self.railslocked) { |
|  | var pos = { |
|  | top: wpos.top, |
|  | left: wpos.left |
|  | }; |
|  | var off = self.opt.railhoffset; |
|  | if (!!off) { |
|  | if (!!off.top) pos.top += off.top; |
|  | if (!!off.left) pos.left += off.left; |
|  | } |
|  | var y = (self.railh.align) ? pos.top + getWidthToPixel(self.win, 'border-top-width', true) + self.win.innerHeight() - self.railh.height : pos.top + getWidthToPixel(self.win, 'border-top-width', true); |
|  | var x = pos.left + getWidthToPixel(self.win, 'border-left-width'); |
|  | self.railh.css({ |
|  | top: y - (self.opt.railpadding.top + self.opt.railpadding.bottom), |
|  | left: x, |
|  | width: self.railh.width |
|  | }); |
|  | } |
|  |  |
|  |  |
|  | } |
|  | }; |
|  |  |
|  | this.doRailClick = function(e, dbl, hr) { |
|  | var fn, pg, cur, pos; |
|  |  |
|  | if (self.railslocked) return; |
|  | self.cancelEvent(e); |
|  |  |
|  | if (dbl) { |
|  | fn = (hr) ? self.doScrollLeft : self.doScrollTop; |
|  | cur = (hr) ? ((e.pageX - self.railh.offset().left - (self.cursorwidth / 2)) \* self.scrollratio.x) : ((e.pageY - self.rail.offset().top - (self.cursorheight / 2)) \* self.scrollratio.y); |
|  | fn(cur); |
|  | } else { |
|  | fn = (hr) ? self.doScrollLeftBy : self.doScrollBy; |
|  | cur = (hr) ? self.scroll.x : self.scroll.y; |
|  | pos = (hr) ? e.pageX - self.railh.offset().left : e.pageY - self.rail.offset().top; |
|  | pg = (hr) ? self.view.w : self.view.h; |
|  | fn((cur >= pos) ? pg: -pg);// (cur >= pos) ? fn(pg): fn(-pg); |
|  | } |
|  |  |
|  | }; |
|  |  |
|  | self.hasanimationframe = (setAnimationFrame); |
|  | self.hascancelanimationframe = (clearAnimationFrame); |
|  |  |
|  | if (!self.hasanimationframe) { |
|  | setAnimationFrame = function(fn) { |
|  | return setTimeout(fn, 15 - Math.floor((+new Date()) / 1000) % 16); |
|  | }; // 1000/60)}; |
|  | clearAnimationFrame = clearInterval; |
|  | } else if (!self.hascancelanimationframe) clearAnimationFrame = function() { |
|  | self.cancelAnimationFrame = true; |
|  | }; |
|  |  |
|  | this.init = function() { |
|  |  |
|  | self.saved.css = []; |
|  |  |
|  | if (cap.isie7mobile) return true; // SORRY, DO NOT WORK! |
|  | if (cap.isoperamini) return true; // SORRY, DO NOT WORK! |
|  |  |
|  | if (cap.hasmstouch) self.css((self.ispage) ? $("html") : self.win, { |
|  | '-ms-touch-action': 'none' |
|  | }); |
|  |  |
|  | self.zindex = "auto"; |
|  | if (!self.ispage && self.opt.zindex == "auto") { |
|  | self.zindex = getZIndex() \|\| "auto"; |
|  | } else { |
|  | self.zindex = self.opt.zindex; |
|  | } |
|  |  |
|  | if (!self.ispage && self.zindex != "auto") { |
|  | if (self.zindex > globalmaxzindex) globalmaxzindex = self.zindex; |
|  | } |
|  |  |
|  | if (self.isie && self.zindex == 0 && self.opt.zindex == "auto") { // fix IE auto == 0 |
|  | self.zindex = "auto"; |
|  | } |
|  |  |
|  | if (!self.ispage \|\| (!cap.cantouch && !cap.isieold && !cap.isie9mobile)) { |
|  |  |
|  | var cont = self.docscroll; |
|  | if (self.ispage) cont = (self.haswrapper) ? self.win : self.doc; |
|  |  |
|  | if (!cap.isie9mobile) self.css(cont, { |
|  | 'overflow-y': 'hidden' |
|  | }); |
|  |  |
|  | if (self.ispage && cap.isie7) { |
|  | if (self.doc[0].nodeName == 'BODY') self.css($("html"), { |
|  | 'overflow-y': 'hidden' |
|  | }); //IE7 double scrollbar issue |
|  | else if (self.doc[0].nodeName == 'HTML') self.css($("body"), { |
|  | 'overflow-y': 'hidden' |
|  | }); //IE7 double scrollbar issue |
|  | } |
|  |  |
|  | if (cap.isios && !self.ispage && !self.haswrapper) self.css($("body"), { |
|  | "-webkit-overflow-scrolling": "touch" |
|  | }); //force hw acceleration |
|  |  |
|  | var cursor = $(document.createElement('div')); |
|  | cursor.css({ |
|  | position: "relative", |
|  | top: 0, |
|  | "float": "right", |
|  | width: self.opt.cursorwidth, |
|  | height: "0px", |
|  | 'background-color': self.opt.cursorcolor, |
|  | border: self.opt.cursorborder, |
|  | 'background-clip': 'padding-box', |
|  | '-webkit-border-radius': self.opt.cursorborderradius, |
|  | '-moz-border-radius': self.opt.cursorborderradius, |
|  | 'border-radius': self.opt.cursorborderradius |
|  | }); |
|  |  |
|  | cursor.hborder = parseFloat(cursor.outerHeight() - cursor.innerHeight()); |
|  |  |
|  | cursor.addClass('nicescroll-cursors'); |
|  |  |
|  | self.cursor = cursor; |
|  |  |
|  | var rail = $(document.createElement('div')); |
|  | rail.attr('id', self.id); |
|  | rail.addClass('nicescroll-rails nicescroll-rails-vr'); |
|  |  |
|  | var v, a, kp = ["left","right","top","bottom"]; //\*\* |
|  | for (var n in kp) { |
|  | a = kp[n]; |
|  | v = self.opt.railpadding[a]; |
|  | (v) ? rail.css("padding-"+a,v+"px") : self.opt.railpadding[a] = 0; |
|  | } |
|  |  |
|  | rail.append(cursor); |
|  |  |
|  | rail.width = Math.max(parseFloat(self.opt.cursorwidth), cursor.outerWidth()); |
|  | rail.css({ |
|  | width: rail.width + "px", |
|  | 'zIndex': self.zindex, |
|  | "background": self.opt.background, |
|  | cursor: "default" |
|  | }); |
|  |  |
|  | rail.visibility = true; |
|  | rail.scrollable = true; |
|  |  |
|  | rail.align = (self.opt.railalign == "left") ? 0 : 1; |
|  |  |
|  | self.rail = rail; |
|  |  |
|  | self.rail.drag = false; |
|  |  |
|  | var zoom = false; |
|  | if (self.opt.boxzoom && !self.ispage && !cap.isieold) { |
|  | zoom = document.createElement('div'); |
|  |  |
|  | self.bind(zoom, "click", self.doZoom); |
|  | self.bind(zoom, "mouseenter", function() { |
|  | self.zoom.css('opacity', self.opt.cursoropacitymax); |
|  | }); |
|  | self.bind(zoom, "mouseleave", function() { |
|  | self.zoom.css('opacity', self.opt.cursoropacitymin); |
|  | }); |
|  |  |
|  | self.zoom = $(zoom); |
|  | self.zoom.css({ |
|  | "cursor": "pointer", |
|  | 'z-index': self.zindex, |
|  | 'backgroundImage': 'url(' + self.opt.scriptpath + 'zoomico.png)', |
|  | 'height': 18, |
|  | 'width': 18, |
|  | 'backgroundPosition': '0px 0px' |
|  | }); |
|  | if (self.opt.dblclickzoom) self.bind(self.win, "dblclick", self.doZoom); |
|  | if (cap.cantouch && self.opt.gesturezoom) { |
|  | self.ongesturezoom = function(e) { |
|  | if (e.scale > 1.5) self.doZoomIn(e); |
|  | if (e.scale < 0.8) self.doZoomOut(e); |
|  | return self.cancelEvent(e); |
|  | }; |
|  | self.bind(self.win, "gestureend", self.ongesturezoom); |
|  | } |
|  | } |
|  |  |
|  | // init HORIZ |
|  |  |
|  | self.railh = false; |
|  | var railh; |
|  |  |
|  | if (self.opt.horizrailenabled) { |
|  |  |
|  | self.css(cont, { |
|  | 'overflow-x': 'hidden' |
|  | }); |
|  |  |
|  | var cursor = $(document.createElement('div')); |
|  | cursor.css({ |
|  | position: "absolute", |
|  | top: 0, |
|  | height: self.opt.cursorwidth, |
|  | width: "0px", |
|  | 'background-color': self.opt.cursorcolor, |
|  | border: self.opt.cursorborder, |
|  | 'background-clip': 'padding-box', |
|  | '-webkit-border-radius': self.opt.cursorborderradius, |
|  | '-moz-border-radius': self.opt.cursorborderradius, |
|  | 'border-radius': self.opt.cursorborderradius |
|  | }); |
|  |  |
|  | if (cap.isieold) cursor.css({'overflow':'hidden'}); //IE6 horiz scrollbar issue |
|  |  |
|  | cursor.wborder = parseFloat(cursor.outerWidth() - cursor.innerWidth()); |
|  |  |
|  | cursor.addClass('nicescroll-cursors'); |
|  |  |
|  | self.cursorh = cursor; |
|  |  |
|  | railh = $(document.createElement('div')); |
|  | railh.attr('id', self.id + '-hr'); |
|  | railh.addClass('nicescroll-rails nicescroll-rails-hr'); |
|  | railh.height = Math.max(parseFloat(self.opt.cursorwidth), cursor.outerHeight()); |
|  | railh.css({ |
|  | height: railh.height + "px", |
|  | 'zIndex': self.zindex, |
|  | "background": self.opt.background |
|  | }); |
|  |  |
|  | railh.append(cursor); |
|  |  |
|  | railh.visibility = true; |
|  | railh.scrollable = true; |
|  |  |
|  | railh.align = (self.opt.railvalign == "top") ? 0 : 1; |
|  |  |
|  | self.railh = railh; |
|  |  |
|  | self.railh.drag = false; |
|  |  |
|  | } |
|  |  |
|  | // |
|  |  |
|  | if (self.ispage) { |
|  | rail.css({ |
|  | position: "fixed", |
|  | top: "0px", |
|  | height: "100%" |
|  | }); |
|  | (rail.align) ? rail.css({ |
|  | right: "0px" |
|  | }): rail.css({ |
|  | left: "0px" |
|  | }); |
|  | self.body.append(rail); |
|  | if (self.railh) { |
|  | railh.css({ |
|  | position: "fixed", |
|  | left: "0px", |
|  | width: "100%" |
|  | }); |
|  | (railh.align) ? railh.css({ |
|  | bottom: "0px" |
|  | }): railh.css({ |
|  | top: "0px" |
|  | }); |
|  | self.body.append(railh); |
|  | } |
|  | } else { |
|  | if (self.ishwscroll) { |
|  | if (self.win.css('position') == 'static') self.css(self.win, { |
|  | 'position': 'relative' |
|  | }); |
|  | var bd = (self.win[0].nodeName == 'HTML') ? self.body : self.win; |
|  | $(bd).scrollTop(0).scrollLeft(0); // fix rail position if content already scrolled |
|  | if (self.zoom) { |
|  | self.zoom.css({ |
|  | position: "absolute", |
|  | top: 1, |
|  | right: 0, |
|  | "margin-right": rail.width + 4 |
|  | }); |
|  | bd.append(self.zoom); |
|  | } |
|  | rail.css({ |
|  | position: "absolute", |
|  | top: 0 |
|  | }); |
|  | (rail.align) ? rail.css({ |
|  | right: 0 |
|  | }): rail.css({ |
|  | left: 0 |
|  | }); |
|  | bd.append(rail); |
|  | if (railh) { |
|  | railh.css({ |
|  | position: "absolute", |
|  | left: 0, |
|  | bottom: 0 |
|  | }); |
|  | (railh.align) ? railh.css({ |
|  | bottom: 0 |
|  | }): railh.css({ |
|  | top: 0 |
|  | }); |
|  | bd.append(railh); |
|  | } |
|  | } else { |
|  | self.isfixed = (self.win.css("position") == "fixed"); |
|  | var rlpos = (self.isfixed) ? "fixed" : "absolute"; |
|  |  |
|  | if (!self.isfixed) self.viewport = self.getViewport(self.win[0]); |
|  | if (self.viewport) { |
|  | self.body = self.viewport; |
|  | if ((/fixed\|absolute/.test(self.viewport.css("position"))) == false) self.css(self.viewport, { |
|  | "position": "relative" |
|  | }); |
|  | } |
|  |  |
|  | rail.css({ |
|  | position: rlpos |
|  | }); |
|  | if (self.zoom) self.zoom.css({ |
|  | position: rlpos |
|  | }); |
|  | self.updateScrollBar(); |
|  | self.body.append(rail); |
|  | if (self.zoom) self.body.append(self.zoom); |
|  | if (self.railh) { |
|  | railh.css({ |
|  | position: rlpos |
|  | }); |
|  | self.body.append(railh); |
|  | } |
|  | } |
|  |  |
|  | if (cap.isios) self.css(self.win, { |
|  | '-webkit-tap-highlight-color': 'rgba(0,0,0,0)', |
|  | '-webkit-touch-callout': 'none' |
|  | }); // prevent grey layer on click |
|  |  |
|  | if (cap.isie && self.opt.disableoutline) self.win.attr("hideFocus", "true"); // IE, prevent dotted rectangle on focused div |
|  | if (cap.iswebkit && self.opt.disableoutline) self.win.css({"outline": "none"}); // Webkit outline |
|  | //if (cap.isopera&&self.opt.disableoutline) self.win.css({"outline":"0"}); // Opera 12- to test [TODO] |
|  |  |
|  | } |
|  |  |
|  | if (self.opt.autohidemode === false) { |
|  | self.autohidedom = false; |
|  | self.rail.css({ |
|  | opacity: self.opt.cursoropacitymax |
|  | }); |
|  | if (self.railh) self.railh.css({ |
|  | opacity: self.opt.cursoropacitymax |
|  | }); |
|  | } else if ((self.opt.autohidemode === true) \|\| (self.opt.autohidemode === "leave")) { |
|  | self.autohidedom = $().add(self.rail); |
|  | if (cap.isie8) self.autohidedom = self.autohidedom.add(self.cursor); |
|  | if (self.railh) self.autohidedom = self.autohidedom.add(self.railh); |
|  | if (self.railh && cap.isie8) self.autohidedom = self.autohidedom.add(self.cursorh); |
|  | } else if (self.opt.autohidemode == "scroll") { |
|  | self.autohidedom = $().add(self.rail); |
|  | if (self.railh) self.autohidedom = self.autohidedom.add(self.railh); |
|  | } else if (self.opt.autohidemode == "cursor") { |
|  | self.autohidedom = $().add(self.cursor); |
|  | if (self.railh) self.autohidedom = self.autohidedom.add(self.cursorh); |
|  | } else if (self.opt.autohidemode == "hidden") { |
|  | self.autohidedom = false; |
|  | self.hide(); |
|  | self.railslocked = false; |
|  | } |
|  |  |
|  | if (cap.isie9mobile) { |
|  |  |
|  | self.scrollmom = new ScrollMomentumClass2D(self); |
|  |  |
|  | self.onmangotouch = function() { |
|  | var py = self.getScrollTop(); |
|  | var px = self.getScrollLeft(); |
|  |  |
|  | if ((py == self.scrollmom.lastscrolly) && (px == self.scrollmom.lastscrollx)) return true; |
|  |  |
|  | var dfy = py - self.mangotouch.sy; |
|  | var dfx = px - self.mangotouch.sx; |
|  | var df = Math.round(Math.sqrt(Math.pow(dfx, 2) + Math.pow(dfy, 2))); |
|  | if (df == 0) return; |
|  |  |
|  | var dry = (dfy < 0) ? -1 : 1; |
|  | var drx = (dfx < 0) ? -1 : 1; |
|  |  |
|  | var tm = +new Date(); |
|  | if (self.mangotouch.lazy) clearTimeout(self.mangotouch.lazy); |
|  |  |
|  | if (((tm - self.mangotouch.tm) > 80) \|\| (self.mangotouch.dry != dry) \|\| (self.mangotouch.drx != drx)) { |
|  | self.scrollmom.stop(); |
|  | self.scrollmom.reset(px, py); |
|  | self.mangotouch.sy = py; |
|  | self.mangotouch.ly = py; |
|  | self.mangotouch.sx = px; |
|  | self.mangotouch.lx = px; |
|  | self.mangotouch.dry = dry; |
|  | self.mangotouch.drx = drx; |
|  | self.mangotouch.tm = tm; |
|  | } else { |
|  |  |
|  | self.scrollmom.stop(); |
|  | self.scrollmom.update(self.mangotouch.sx - dfx, self.mangotouch.sy - dfy); |
|  | self.mangotouch.tm = tm; |
|  |  |
|  | var ds = Math.max(Math.abs(self.mangotouch.ly - py), Math.abs(self.mangotouch.lx - px)); |
|  | self.mangotouch.ly = py; |
|  | self.mangotouch.lx = px; |
|  |  |
|  | if (ds > 2) { |
|  | self.mangotouch.lazy = setTimeout(function() { |
|  | self.mangotouch.lazy = false; |
|  | self.mangotouch.dry = 0; |
|  | self.mangotouch.drx = 0; |
|  | self.mangotouch.tm = 0; |
|  | self.scrollmom.doMomentum(30); |
|  | }, 100); |
|  | } |
|  | } |
|  | }; |
|  |  |
|  | var top = self.getScrollTop(); |
|  | var lef = self.getScrollLeft(); |
|  | self.mangotouch = { |
|  | sy: top, |
|  | ly: top, |
|  | dry: 0, |
|  | sx: lef, |
|  | lx: lef, |
|  | drx: 0, |
|  | lazy: false, |
|  | tm: 0 |
|  | }; |
|  |  |
|  | self.bind(self.docscroll, "scroll", self.onmangotouch); |
|  |  |
|  | } else { |
|  |  |
|  | if (cap.cantouch \|\| self.istouchcapable \|\| self.opt.touchbehavior \|\| cap.hasmstouch) { |
|  |  |
|  | self.scrollmom = new ScrollMomentumClass2D(self); |
|  |  |
|  | self.ontouchstart = function(e) { |
|  | if (e.pointerType && e.pointerType != 2 && e.pointerType != "touch") return false; |
|  |  |
|  | self.hasmoving = false; |
|  |  |
|  | if (!self.railslocked) { |
|  |  |
|  | var tg; |
|  | if (cap.hasmstouch) { |
|  | tg = (e.target) ? e.target : false; |
|  | while (tg) { |
|  | var nc = $(tg).getNiceScroll(); |
|  | if ((nc.length > 0) && (nc[0].me == self.me)) break; |
|  | if (nc.length > 0) return false; |
|  | if ((tg.nodeName == 'DIV') && (tg.id == self.id)) break; |
|  | tg = (tg.parentNode) ? tg.parentNode : false; |
|  | } |
|  | } |
|  |  |
|  | self.cancelScroll(); |
|  |  |
|  | tg = self.getTarget(e); |
|  |  |
|  | if (tg) { |
|  | var skp = (/INPUT/i.test(tg.nodeName)) && (/range/i.test(tg.type)); |
|  | if (skp) return self.stopPropagation(e); |
|  | } |
|  |  |
|  | if (!("clientX" in e) && ("changedTouches" in e)) { |
|  | e.clientX = e.changedTouches[0].clientX; |
|  | e.clientY = e.changedTouches[0].clientY; |
|  | } |
|  |  |
|  | if (self.forcescreen) { |
|  | var le = e; |
|  | e = { |
|  | "original": (e.original) ? e.original : e |
|  | }; |
|  | e.clientX = le.screenX; |
|  | e.clientY = le.screenY; |
|  | } |
|  |  |
|  | self.rail.drag = { |
|  | x: e.clientX, |
|  | y: e.clientY, |
|  | sx: self.scroll.x, |
|  | sy: self.scroll.y, |
|  | st: self.getScrollTop(), |
|  | sl: self.getScrollLeft(), |
|  | pt: 2, |
|  | dl: false |
|  | }; |
|  |  |
|  | if (self.ispage \|\| !self.opt.directionlockdeadzone) { |
|  | self.rail.drag.dl = "f"; |
|  | } else { |
|  |  |
|  | var view = { |
|  | w: $(window).width(), |
|  | h: $(window).height() |
|  | }; |
|  |  |
|  | var page = { |
|  | w: Math.max(document.body.scrollWidth, document.documentElement.scrollWidth), |
|  | h: Math.max(document.body.scrollHeight, document.documentElement.scrollHeight) |
|  | }; |
|  |  |
|  | var maxh = Math.max(0, page.h - view.h); |
|  | var maxw = Math.max(0, page.w - view.w); |
|  |  |
|  | if (!self.rail.scrollable && self.railh.scrollable) self.rail.drag.ck = (maxh > 0) ? "v" : false; |
|  | else if (self.rail.scrollable && !self.railh.scrollable) self.rail.drag.ck = (maxw > 0) ? "h" : false; |
|  | else self.rail.drag.ck = false; |
|  | if (!self.rail.drag.ck) self.rail.drag.dl = "f"; |
|  | } |
|  |  |
|  | if (self.opt.touchbehavior && self.isiframe && cap.isie) { |
|  | var wp = self.win.position(); |
|  | self.rail.drag.x += wp.left; |
|  | self.rail.drag.y += wp.top; |
|  | } |
|  |  |
|  | self.hasmoving = false; |
|  | self.lastmouseup = false; |
|  | self.scrollmom.reset(e.clientX, e.clientY); |
|  |  |
|  | if (!cap.cantouch && !this.istouchcapable && !e.pointerType) { |
|  |  |
|  | var ip = (tg) ? /INPUT\|SELECT\|TEXTAREA/i.test(tg.nodeName) : false; |
|  | if (!ip) { |
|  | if (!self.ispage && cap.hasmousecapture) tg.setCapture(); |
|  | if (self.opt.touchbehavior) { |
|  | if (tg.onclick && !(tg.\_onclick \|\| false)) { // intercept DOM0 onclick event |
|  | tg.\_onclick = tg.onclick; |
|  | tg.onclick = function(e) { |
|  | if (self.hasmoving) return false; |
|  | tg.\_onclick.call(this, e); |
|  | }; |
|  | } |
|  | return self.cancelEvent(e); |
|  | } |
|  | return self.stopPropagation(e); |
|  | } |
|  |  |
|  | if (/SUBMIT\|CANCEL\|BUTTON/i.test($(tg).attr('type'))) { |
|  | pc = { |
|  | "tg": tg, |
|  | "click": false |
|  | }; |
|  | self.preventclick = pc; |
|  | } |
|  |  |
|  | } |
|  | } |
|  |  |
|  | }; |
|  |  |
|  | self.ontouchend = function(e) { |
|  | if (!self.rail.drag) return true; |
|  | if (self.rail.drag.pt == 2) { |
|  | if (e.pointerType && e.pointerType != 2 && e.pointerType != "touch") return false; |
|  | self.scrollmom.doMomentum(); |
|  | self.rail.drag = false; |
|  | if (self.hasmoving) { |
|  | self.lastmouseup = true; |
|  | self.hideCursor(); |
|  | if (cap.hasmousecapture) document.releaseCapture(); |
|  | if (!cap.cantouch) return self.cancelEvent(e); |
|  | } |
|  | } |
|  | else if (self.rail.drag.pt == 1) { |
|  | return self.onmouseup(e); |
|  | } |
|  |  |
|  | }; |
|  |  |
|  | var moveneedoffset = (self.opt.touchbehavior && self.isiframe && !cap.hasmousecapture); |
|  |  |
|  | self.ontouchmove = function(e, byiframe) { |
|  |  |
|  | if (!self.rail.drag) return false; |
|  |  |
|  | if (e.targetTouches && self.opt.preventmultitouchscrolling) { |
|  | if (e.targetTouches.length > 1) return false; // multitouch |
|  | } |
|  |  |
|  | if (e.pointerType && e.pointerType != 2 && e.pointerType != "touch") return false; |
|  |  |
|  | if (self.rail.drag.pt == 2) { |
|  | if (cap.cantouch && (cap.isios) && (typeof e.original == "undefined")) return true; // prevent ios "ghost" events by clickable elements |
|  |  |
|  | self.hasmoving = true; |
|  |  |
|  | if (self.preventclick && !self.preventclick.click) { |
|  | self.preventclick.click = self.preventclick.tg.onclick \|\| false; |
|  | self.preventclick.tg.onclick = self.onpreventclick; |
|  | } |
|  |  |
|  | var ev = $.extend({ |
|  | "original": e |
|  | }, e); |
|  | e = ev; |
|  |  |
|  | if (("changedTouches" in e)) { |
|  | e.clientX = e.changedTouches[0].clientX; |
|  | e.clientY = e.changedTouches[0].clientY; |
|  | } |
|  |  |
|  | if (self.forcescreen) { |
|  | var le = e; |
|  | e = { |
|  | "original": (e.original) ? e.original : e |
|  | }; |
|  | e.clientX = le.screenX; |
|  | e.clientY = le.screenY; |
|  | } |
|  |  |
|  | var ofy,ofx; |
|  | ofx = ofy = 0; |
|  |  |
|  | if (moveneedoffset && !byiframe) { |
|  | var wp = self.win.position(); |
|  | ofx = -wp.left; |
|  | ofy = -wp.top; |
|  | } |
|  |  |
|  | var fy = e.clientY + ofy; |
|  | var my = (fy - self.rail.drag.y); |
|  | var fx = e.clientX + ofx; |
|  | var mx = (fx - self.rail.drag.x); |
|  |  |
|  | var ny = self.rail.drag.st - my; |
|  |  |
|  | if (self.ishwscroll && self.opt.bouncescroll) { |
|  | if (ny < 0) { |
|  | ny = Math.round(ny / 2); |
|  | // fy = 0; |
|  | } else if (ny > self.page.maxh) { |
|  | ny = self.page.maxh + Math.round((ny - self.page.maxh) / 2); |
|  | // fy = 0; |
|  | } |
|  | } else { |
|  | if (ny < 0) { |
|  | ny = 0; |
|  | fy = 0; |
|  | } |
|  | if (ny > self.page.maxh) { |
|  | ny = self.page.maxh; |
|  | fy = 0; |
|  | } |
|  | } |
|  |  |
|  | var nx; |
|  | if (self.railh && self.railh.scrollable) { |
|  | nx = (self.isrtlmode) ? mx - self.rail.drag.sl : self.rail.drag.sl - mx; |
|  |  |
|  | if (self.ishwscroll && self.opt.bouncescroll) { |
|  | if (nx < 0) { |
|  | nx = Math.round(nx / 2); |
|  | // fx = 0; |
|  | } else if (nx > self.page.maxw) { |
|  | nx = self.page.maxw + Math.round((nx - self.page.maxw) / 2); |
|  | // fx = 0; |
|  | } |
|  | } else { |
|  | if (nx < 0) { |
|  | nx = 0; |
|  | fx = 0; |
|  | } |
|  | if (nx > self.page.maxw) { |
|  | nx = self.page.maxw; |
|  | fx = 0; |
|  | } |
|  | } |
|  |  |
|  | } |
|  |  |
|  | var grabbed = false; |
|  | if (self.rail.drag.dl) { |
|  | grabbed = true; |
|  | if (self.rail.drag.dl == "v") nx = self.rail.drag.sl; |
|  | else if (self.rail.drag.dl == "h") ny = self.rail.drag.st; |
|  | } else { |
|  | var ay = Math.abs(my); |
|  | var ax = Math.abs(mx); |
|  | var dz = self.opt.directionlockdeadzone; |
|  | if (self.rail.drag.ck == "v") { |
|  | if (ay > dz && (ax <= (ay \* 0.3))) { |
|  | self.rail.drag = false; |
|  | return true; |
|  | } else if (ax > dz) { |
|  | self.rail.drag.dl = "f"; |
|  | $("body").scrollTop($("body").scrollTop()); // stop iOS native scrolling (when active javascript has blocked) |
|  | } |
|  | } else if (self.rail.drag.ck == "h") { |
|  | if (ax > dz && (ay <= (ax \* 0.3))) { |
|  | self.rail.drag = false; |
|  | return true; |
|  | } else if (ay > dz) { |
|  | self.rail.drag.dl = "f"; |
|  | $("body").scrollLeft($("body").scrollLeft()); // stop iOS native scrolling (when active javascript has blocked) |
|  | } |
|  | } |
|  | } |
|  |  |
|  | self.synched("touchmove", function() { |
|  | if (self.rail.drag && (self.rail.drag.pt == 2)) { |
|  | if (self.prepareTransition) self.prepareTransition(0); |
|  | if (self.rail.scrollable) self.setScrollTop(ny); |
|  | self.scrollmom.update(fx, fy); |
|  | if (self.railh && self.railh.scrollable) { |
|  | self.setScrollLeft(nx); |
|  | self.showCursor(ny, nx); |
|  | } else { |
|  | self.showCursor(ny); |
|  | } |
|  | if (cap.isie10) document.selection.clear(); |
|  | } |
|  | }); |
|  |  |
|  | if (cap.ischrome && self.istouchcapable) grabbed = false; //chrome touch emulation doesn't like! |
|  | if (grabbed) return self.cancelEvent(e); |
|  | } |
|  | else if (self.rail.drag.pt == 1) { // drag on cursor |
|  | return self.onmousemove(e); |
|  | } |
|  |  |
|  | }; |
|  |  |
|  | } |
|  |  |
|  | self.onmousedown = function(e, hronly) { |
|  | if (self.rail.drag && self.rail.drag.pt != 1) return; |
|  | if (self.railslocked) return self.cancelEvent(e); |
|  | self.cancelScroll(); |
|  | self.rail.drag = { |
|  | x: e.clientX, |
|  | y: e.clientY, |
|  | sx: self.scroll.x, |
|  | sy: self.scroll.y, |
|  | pt: 1, |
|  | hr: (!!hronly) |
|  | }; |
|  | var tg = self.getTarget(e); |
|  | if (!self.ispage && cap.hasmousecapture) tg.setCapture(); |
|  | if (self.isiframe && !cap.hasmousecapture) { |
|  | self.saved.csspointerevents = self.doc.css("pointer-events"); |
|  | self.css(self.doc, { |
|  | "pointer-events": "none" |
|  | }); |
|  | } |
|  | self.hasmoving = false; |
|  | return self.cancelEvent(e); |
|  | }; |
|  |  |
|  | self.onmouseup = function(e) { |
|  | if (self.rail.drag) { |
|  | if (self.rail.drag.pt != 1) return true; |
|  | if (cap.hasmousecapture) document.releaseCapture(); |
|  | if (self.isiframe && !cap.hasmousecapture) self.doc.css("pointer-events", self.saved.csspointerevents); |
|  | self.rail.drag = false; |
|  | //if (!self.rail.active) self.hideCursor(); |
|  | if (self.hasmoving) self.triggerScrollEnd(); // TODO - check &&!self.scrollrunning |
|  | return self.cancelEvent(e); |
|  | } |
|  | }; |
|  |  |
|  | self.onmousemove = function(e) { |
|  | if (self.rail.drag) { |
|  | if (self.rail.drag.pt != 1) return; |
|  |  |
|  | if (cap.ischrome && e.which == 0) return self.onmouseup(e); |
|  |  |
|  | self.cursorfreezed = true; |
|  | self.hasmoving = true; |
|  |  |
|  | if (self.rail.drag.hr) { |
|  | self.scroll.x = self.rail.drag.sx + (e.clientX - self.rail.drag.x); |
|  | if (self.scroll.x < 0) self.scroll.x = 0; |
|  | var mw = self.scrollvaluemaxw; |
|  | if (self.scroll.x > mw) self.scroll.x = mw; |
|  | } else { |
|  | self.scroll.y = self.rail.drag.sy + (e.clientY - self.rail.drag.y); |
|  | if (self.scroll.y < 0) self.scroll.y = 0; |
|  | var my = self.scrollvaluemax; |
|  | if (self.scroll.y > my) self.scroll.y = my; |
|  | } |
|  |  |
|  | self.synched('mousemove', function() { |
|  | if (self.rail.drag && (self.rail.drag.pt == 1)) { |
|  | self.showCursor(); |
|  | if (self.rail.drag.hr) { |
|  | if (self.hasreversehr) { |
|  | self.doScrollLeft(self.scrollvaluemaxw-Math.round(self.scroll.x \* self.scrollratio.x), self.opt.cursordragspeed); |
|  | } else { |
|  | self.doScrollLeft(Math.round(self.scroll.x \* self.scrollratio.x), self.opt.cursordragspeed); |
|  | } |
|  | } |
|  | else self.doScrollTop(Math.round(self.scroll.y \* self.scrollratio.y), self.opt.cursordragspeed); |
|  | } |
|  | }); |
|  |  |
|  | return self.cancelEvent(e); |
|  | } |
|  | /\* |
|  | else { |
|  | self.checkarea = true; |
|  | } |
|  | \*/ |
|  | }; |
|  |  |
|  | if (cap.cantouch \|\| self.opt.touchbehavior) { |
|  |  |
|  | self.onpreventclick = function(e) { |
|  | if (self.preventclick) { |
|  | self.preventclick.tg.onclick = self.preventclick.click; |
|  | self.preventclick = false; |
|  | return self.cancelEvent(e); |
|  | } |
|  | } |
|  |  |
|  | self.bind(self.win, "mousedown", self.ontouchstart); // control content dragging |
|  |  |
|  | self.onclick = (cap.isios) ? false : function(e) { |
|  | if (self.lastmouseup) { |
|  | self.lastmouseup = false; |
|  | return self.cancelEvent(e); |
|  | } else { |
|  | return true; |
|  | } |
|  | }; |
|  |  |
|  | if (self.opt.grabcursorenabled && cap.cursorgrabvalue) { |
|  | self.css((self.ispage) ? self.doc : self.win, { |
|  | 'cursor': cap.cursorgrabvalue |
|  | }); |
|  | self.css(self.rail, { |
|  | 'cursor': cap.cursorgrabvalue |
|  | }); |
|  | } |
|  |  |
|  | } else { |
|  |  |
|  | var checkSelectionScroll = function(e) { |
|  | if (!self.selectiondrag) return; |
|  |  |
|  | if (e) { |
|  | var ww = self.win.outerHeight(); |
|  | var df = (e.pageY - self.selectiondrag.top); |
|  | if (df > 0 && df < ww) df = 0; |
|  | if (df >= ww) df -= ww; |
|  | self.selectiondrag.df = df; |
|  | } |
|  | if (self.selectiondrag.df == 0) return; |
|  |  |
|  | var rt = -Math.floor(self.selectiondrag.df / 6) \* 2; |
|  | self.doScrollBy(rt); |
|  |  |
|  | self.debounced("doselectionscroll", function() { |
|  | checkSelectionScroll() |
|  | }, 50); |
|  | }; |
|  |  |
|  | if ("getSelection" in document) { // A grade - Major browsers |
|  | self.hasTextSelected = function() { |
|  | return (document.getSelection().rangeCount > 0); |
|  | }; |
|  | } else if ("selection" in document) { //IE9- |
|  | self.hasTextSelected = function() { |
|  | return (document.selection.type != "None"); |
|  | }; |
|  | } else { |
|  | self.hasTextSelected = function() { // no support |
|  | return false; |
|  | }; |
|  | } |
|  |  |
|  | self.onselectionstart = function(e) { |
|  | /\* More testing - severe chrome issues |
|  | if (!self.haswrapper&&(e.which&&e.which==2)) { // fool browser to manage middle button scrolling |
|  | self.win.css({'overflow':'auto'}); |
|  | setTimeout(function(){ |
|  | self.win.css({'overflow':''}); |
|  | },10); |
|  | return true; |
|  | } |
|  | \*/ |
|  | if (self.ispage) return; |
|  | self.selectiondrag = self.win.offset(); |
|  | }; |
|  |  |
|  | self.onselectionend = function(e) { |
|  | self.selectiondrag = false; |
|  | }; |
|  | self.onselectiondrag = function(e) { |
|  | if (!self.selectiondrag) return; |
|  | if (self.hasTextSelected()) self.debounced("selectionscroll", function() { |
|  | checkSelectionScroll(e) |
|  | }, 250); |
|  | }; |
|  |  |
|  |  |
|  | } |
|  |  |
|  | if (cap.hasw3ctouch) { //IE11+ |
|  | self.css(self.rail, { |
|  | 'touch-action': 'none' |
|  | }); |
|  | self.css(self.cursor, { |
|  | 'touch-action': 'none' |
|  | }); |
|  | self.bind(self.win, "pointerdown", self.ontouchstart); |
|  | self.bind(document, "pointerup", self.ontouchend); |
|  | self.bind(document, "pointermove", self.ontouchmove); |
|  | } else if (cap.hasmstouch) { //IE10 |
|  | self.css(self.rail, { |
|  | '-ms-touch-action': 'none' |
|  | }); |
|  | self.css(self.cursor, { |
|  | '-ms-touch-action': 'none' |
|  | }); |
|  | self.bind(self.win, "MSPointerDown", self.ontouchstart); |
|  | self.bind(document, "MSPointerUp", self.ontouchend); |
|  | self.bind(document, "MSPointerMove", self.ontouchmove); |
|  | self.bind(self.cursor, "MSGestureHold", function(e) { |
|  | e.preventDefault() |
|  | }); |
|  | self.bind(self.cursor, "contextmenu", function(e) { |
|  | e.preventDefault() |
|  | }); |
|  | } else if (this.istouchcapable) { //desktop with screen touch enabled |
|  | self.bind(self.win, "touchstart", self.ontouchstart); |
|  | self.bind(document, "touchend", self.ontouchend); |
|  | self.bind(document, "touchcancel", self.ontouchend); |
|  | self.bind(document, "touchmove", self.ontouchmove); |
|  | } |
|  |  |
|  |  |
|  | if (self.opt.cursordragontouch \|\| (!cap.cantouch && !self.opt.touchbehavior)) { |
|  |  |
|  | self.rail.css({ |
|  | "cursor": "default" |
|  | }); |
|  | self.railh && self.railh.css({ |
|  | "cursor": "default" |
|  | }); |
|  |  |
|  | self.jqbind(self.rail, "mouseenter", function() { |
|  | if (!self.ispage && !self.win.is(":visible")) return false; |
|  | if (self.canshowonmouseevent) self.showCursor(); |
|  | self.rail.active = true; |
|  | }); |
|  | self.jqbind(self.rail, "mouseleave", function() { |
|  | self.rail.active = false; |
|  | if (!self.rail.drag) self.hideCursor(); |
|  | }); |
|  |  |
|  | if (self.opt.sensitiverail) { |
|  | self.bind(self.rail, "click", function(e) { |
|  | self.doRailClick(e, false, false) |
|  | }); |
|  | self.bind(self.rail, "dblclick", function(e) { |
|  | self.doRailClick(e, true, false) |
|  | }); |
|  | self.bind(self.cursor, "click", function(e) { |
|  | self.cancelEvent(e) |
|  | }); |
|  | self.bind(self.cursor, "dblclick", function(e) { |
|  | self.cancelEvent(e) |
|  | }); |
|  | } |
|  |  |
|  | if (self.railh) { |
|  | self.jqbind(self.railh, "mouseenter", function() { |
|  | if (!self.ispage && !self.win.is(":visible")) return false; |
|  | if (self.canshowonmouseevent) self.showCursor(); |
|  | self.rail.active = true; |
|  | }); |
|  | self.jqbind(self.railh, "mouseleave", function() { |
|  | self.rail.active = false; |
|  | if (!self.rail.drag) self.hideCursor(); |
|  | }); |
|  |  |
|  | if (self.opt.sensitiverail) { |
|  | self.bind(self.railh, "click", function(e) { |
|  | self.doRailClick(e, false, true) |
|  | }); |
|  | self.bind(self.railh, "dblclick", function(e) { |
|  | self.doRailClick(e, true, true) |
|  | }); |
|  | self.bind(self.cursorh, "click", function(e) { |
|  | self.cancelEvent(e) |
|  | }); |
|  | self.bind(self.cursorh, "dblclick", function(e) { |
|  | self.cancelEvent(e) |
|  | }); |
|  | } |
|  |  |
|  | } |
|  |  |
|  | } |
|  |  |
|  | if (!cap.cantouch && !self.opt.touchbehavior) { |
|  |  |
|  | self.bind((cap.hasmousecapture) ? self.win : document, "mouseup", self.onmouseup); |
|  | self.bind(document, "mousemove", self.onmousemove); |
|  | if (self.onclick) self.bind(document, "click", self.onclick); |
|  |  |
|  | self.bind(self.cursor, "mousedown", self.onmousedown); |
|  | self.bind(self.cursor, "mouseup", self.onmouseup); |
|  |  |
|  | if (self.railh) { |
|  | self.bind(self.cursorh, "mousedown", function(e) { |
|  | self.onmousedown(e, true) |
|  | }); |
|  | self.bind(self.cursorh, "mouseup", self.onmouseup); |
|  | } |
|  |  |
|  | if (!self.ispage && self.opt.enablescrollonselection) { |
|  | self.bind(self.win[0], "mousedown", self.onselectionstart); |
|  | self.bind(document, "mouseup", self.onselectionend); |
|  | self.bind(self.cursor, "mouseup", self.onselectionend); |
|  | if (self.cursorh) self.bind(self.cursorh, "mouseup", self.onselectionend); |
|  | self.bind(document, "mousemove", self.onselectiondrag); |
|  | } |
|  |  |
|  | if (self.zoom) { |
|  | self.jqbind(self.zoom, "mouseenter", function() { |
|  | if (self.canshowonmouseevent) self.showCursor(); |
|  | self.rail.active = true; |
|  | }); |
|  | self.jqbind(self.zoom, "mouseleave", function() { |
|  | self.rail.active = false; |
|  | if (!self.rail.drag) self.hideCursor(); |
|  | }); |
|  | } |
|  |  |
|  | } else { |
|  |  |
|  | self.bind((cap.hasmousecapture) ? self.win : document, "mouseup", self.ontouchend); |
|  | self.bind(document, "mousemove", self.ontouchmove); |
|  | if (self.onclick) self.bind(document, "click", self.onclick); |
|  |  |
|  | if (self.opt.cursordragontouch) { |
|  | self.bind(self.cursor, "mousedown", self.onmousedown); |
|  | self.bind(self.cursor, "mouseup", self.onmouseup); |
|  | //self.bind(self.cursor, "mousemove", self.onmousemove); |
|  | self.cursorh && self.bind(self.cursorh, "mousedown", function(e) { |
|  | self.onmousedown(e, true) |
|  | }); |
|  | //self.cursorh && self.bind(self.cursorh, "mousemove", self.onmousemove); |
|  | self.cursorh && self.bind(self.cursorh, "mouseup", self.onmouseup); |
|  | } |
|  |  |
|  | } |
|  |  |
|  | if (self.opt.enablemousewheel) { |
|  | if (!self.isiframe) self.bind((cap.isie && self.ispage) ? document : self.win /\*self.docscroll\*/ , "mousewheel", self.onmousewheel); |
|  | self.bind(self.rail, "mousewheel", self.onmousewheel); |
|  | if (self.railh) self.bind(self.railh, "mousewheel", self.onmousewheelhr); |
|  | } |
|  |  |
|  | if (!self.ispage && !cap.cantouch && !(/HTML\|^BODY/.test(self.win[0].nodeName))) { |
|  | if (!self.win.attr("tabindex")) self.win.attr({ |
|  | "tabindex": tabindexcounter++ |
|  | }); |
|  |  |
|  | self.jqbind(self.win, "focus", function(e) { |
|  | domfocus = (self.getTarget(e)).id \|\| true; |
|  | self.hasfocus = true; |
|  | if (self.canshowonmouseevent) self.noticeCursor(); |
|  | }); |
|  | self.jqbind(self.win, "blur", function(e) { |
|  | domfocus = false; |
|  | self.hasfocus = false; |
|  | }); |
|  |  |
|  | self.jqbind(self.win, "mouseenter", function(e) { |
|  | mousefocus = (self.getTarget(e)).id \|\| true; |
|  | self.hasmousefocus = true; |
|  | if (self.canshowonmouseevent) self.noticeCursor(); |
|  | }); |
|  | self.jqbind(self.win, "mouseleave", function() { |
|  | mousefocus = false; |
|  | self.hasmousefocus = false; |
|  | if (!self.rail.drag) self.hideCursor(); |
|  | }); |
|  |  |
|  | } |
|  |  |
|  | } // !ie9mobile |
|  |  |
|  | //Thanks to http://www.quirksmode.org !! |
|  | self.onkeypress = function(e) { |
|  | if (self.railslocked && self.page.maxh == 0) return true; |
|  |  |
|  | e = (e) ? e : window.e; |
|  | var tg = self.getTarget(e); |
|  | if (tg && /INPUT\|TEXTAREA\|SELECT\|OPTION/.test(tg.nodeName)) { |
|  | var tp = tg.getAttribute('type') \|\| tg.type \|\| false; |
|  | if ((!tp) \|\| !(/submit\|button\|cancel/i.tp)) return true; |
|  | } |
|  |  |
|  | if ($(tg).attr('contenteditable')) return true; |
|  |  |
|  | if (self.hasfocus \|\| (self.hasmousefocus && !domfocus) \|\| (self.ispage && !domfocus && !mousefocus)) { |
|  | var key = e.keyCode; |
|  |  |
|  | if (self.railslocked && key != 27) return self.cancelEvent(e); |
|  |  |
|  | var ctrl = e.ctrlKey \|\| false; |
|  | var shift = e.shiftKey \|\| false; |
|  |  |
|  | var ret = false; |
|  | switch (key) { |
|  | case 38: |
|  | case 63233: //safari |
|  | self.doScrollBy(24 \* 3); |
|  | ret = true; |
|  | break; |
|  | case 40: |
|  | case 63235: //safari |
|  | self.doScrollBy(-24 \* 3); |
|  | ret = true; |
|  | break; |
|  | case 37: |
|  | case 63232: //safari |
|  | if (self.railh) { |
|  | (ctrl) ? self.doScrollLeft(0): self.doScrollLeftBy(24 \* 3); |
|  | ret = true; |
|  | } |
|  | break; |
|  | case 39: |
|  | case 63234: //safari |
|  | if (self.railh) { |
|  | (ctrl) ? self.doScrollLeft(self.page.maxw): self.doScrollLeftBy(-24 \* 3); |
|  | ret = true; |
|  | } |
|  | break; |
|  | case 33: |
|  | case 63276: // safari |
|  | self.doScrollBy(self.view.h); |
|  | ret = true; |
|  | break; |
|  | case 34: |
|  | case 63277: // safari |
|  | self.doScrollBy(-self.view.h); |
|  | ret = true; |
|  | break; |
|  | case 36: |
|  | case 63273: // safari |
|  | (self.railh && ctrl) ? self.doScrollPos(0, 0): self.doScrollTo(0); |
|  | ret = true; |
|  | break; |
|  | case 35: |
|  | case 63275: // safari |
|  | (self.railh && ctrl) ? self.doScrollPos(self.page.maxw, self.page.maxh): self.doScrollTo(self.page.maxh); |
|  | ret = true; |
|  | break; |
|  | case 32: |
|  | if (self.opt.spacebarenabled) { |
|  | (shift) ? self.doScrollBy(self.view.h): self.doScrollBy(-self.view.h); |
|  | ret = true; |
|  | } |
|  | break; |
|  | case 27: // ESC |
|  | if (self.zoomactive) { |
|  | self.doZoom(); |
|  | ret = true; |
|  | } |
|  | break; |
|  | } |
|  | if (ret) return self.cancelEvent(e); |
|  | } |
|  | }; |
|  |  |
|  | if (self.opt.enablekeyboard) self.bind(document, (cap.isopera && !cap.isopera12) ? "keypress" : "keydown", self.onkeypress); |
|  |  |
|  | self.bind(document, "keydown", function(e) { |
|  | var ctrl = e.ctrlKey \|\| false; |
|  | if (ctrl) self.wheelprevented = true; |
|  | }); |
|  | self.bind(document, "keyup", function(e) { |
|  | var ctrl = e.ctrlKey \|\| false; |
|  | if (!ctrl) self.wheelprevented = false; |
|  | }); |
|  | self.bind(window,"blur",function(e){ |
|  | self.wheelprevented = false; |
|  | }); |
|  |  |
|  | self.bind(window, 'resize', self.lazyResize); |
|  | self.bind(window, 'orientationchange', self.lazyResize); |
|  |  |
|  | self.bind(window, "load", self.lazyResize); |
|  |  |
|  | if (cap.ischrome && !self.ispage && !self.haswrapper) { //chrome void scrollbar bug - it persists in version 26 |
|  | var tmp = self.win.attr("style"); |
|  | var ww = parseFloat(self.win.css("width")) + 1; |
|  | self.win.css('width', ww); |
|  | self.synched("chromefix", function() { |
|  | self.win.attr("style", tmp) |
|  | }); |
|  | } |
|  |  |
|  |  |
|  | // Trying a cross-browser implementation - good luck! |
|  |  |
|  | self.onAttributeChange = function(e) { |
|  | self.lazyResize(self.isieold ? 250 : 30); |
|  | }; |
|  |  |
|  | if (ClsMutationObserver !== false) { |
|  | self.observerbody = new ClsMutationObserver(function(mutations) { |
|  | mutations.forEach(function(mut){ |
|  | if (mut.type=="attributes") { |
|  | return ($("body").hasClass("modal-open")) ? self.hide() : self.show(); // Support for Bootstrap modal |
|  | } |
|  | }); |
|  | if (document.body.scrollHeight!=self.page.maxh) return self.lazyResize(30); |
|  | }); |
|  | self.observerbody.observe(document.body, { |
|  | childList: true, |
|  | subtree: true, |
|  | characterData: false, |
|  | attributes: true, |
|  | attributeFilter: ['class'] |
|  | }); |
|  | } |
|  |  |
|  | if (!self.ispage && !self.haswrapper) { |
|  | // redesigned MutationObserver for Chrome18+/Firefox14+/iOS6+ with support for: remove div, add/remove content |
|  | if (ClsMutationObserver !== false) { |
|  | self.observer = new ClsMutationObserver(function(mutations) { |
|  | mutations.forEach(self.onAttributeChange); |
|  | }); |
|  | self.observer.observe(self.win[0], { |
|  | childList: true, |
|  | characterData: false, |
|  | attributes: true, |
|  | subtree: false |
|  | }); |
|  | self.observerremover = new ClsMutationObserver(function(mutations) { |
|  | mutations.forEach(function(mo) { |
|  | if (mo.removedNodes.length > 0) { |
|  | for (var dd in mo.removedNodes) { |
|  | if (!!self && (mo.removedNodes[dd] == self.win[0])) return self.remove(); |
|  | } |
|  | } |
|  | }); |
|  | }); |
|  | self.observerremover.observe(self.win[0].parentNode, { |
|  | childList: true, |
|  | characterData: false, |
|  | attributes: false, |
|  | subtree: false |
|  | }); |
|  | } else { |
|  | self.bind(self.win, (cap.isie && !cap.isie9) ? "propertychange" : "DOMAttrModified", self.onAttributeChange); |
|  | if (cap.isie9) self.win[0].attachEvent("onpropertychange", self.onAttributeChange); //IE9 DOMAttrModified bug |
|  | self.bind(self.win, "DOMNodeRemoved", function(e) { |
|  | if (e.target == self.win[0]) self.remove(); |
|  | }); |
|  | } |
|  | } |
|  |  |
|  | // |
|  |  |
|  | if (!self.ispage && self.opt.boxzoom) self.bind(window, "resize", self.resizeZoom); |
|  | if (self.istextarea) self.bind(self.win, "mouseup", self.lazyResize); |
|  |  |
|  | // self.checkrtlmode = true; |
|  | self.lazyResize(30); |
|  |  |
|  | } |
|  |  |
|  | if (this.doc[0].nodeName == 'IFRAME') { |
|  | var oniframeload = function() { |
|  | self.iframexd = false; |
|  | var doc; |
|  | try { |
|  | doc = 'contentDocument' in this ? this.contentDocument : this.contentWindow.document; |
|  | var a = doc.domain; |
|  | } catch (e) { |
|  | self.iframexd = true; |
|  | doc = false |
|  | } |
|  |  |
|  | if (self.iframexd) { |
|  | if ("console" in window) console.log('NiceScroll error: policy restriced iframe'); |
|  | return true; //cross-domain - I can't manage this |
|  | } |
|  |  |
|  | self.forcescreen = true; |
|  |  |
|  | if (self.isiframe) { |
|  | self.iframe = { |
|  | "doc": $(doc), |
|  | "html": self.doc.contents().find('html')[0], |
|  | "body": self.doc.contents().find('body')[0] |
|  | }; |
|  | self.getContentSize = function() { |
|  | return { |
|  | w: Math.max(self.iframe.html.scrollWidth, self.iframe.body.scrollWidth), |
|  | h: Math.max(self.iframe.html.scrollHeight, self.iframe.body.scrollHeight) |
|  | }; |
|  | }; |
|  | self.docscroll = $(self.iframe.body); //$(this.contentWindow); |
|  | } |
|  |  |
|  | if (!cap.isios && self.opt.iframeautoresize && !self.isiframe) { |
|  | self.win.scrollTop(0); // reset position |
|  | self.doc.height(""); //reset height to fix browser bug |
|  | var hh = Math.max(doc.getElementsByTagName('html')[0].scrollHeight, doc.body.scrollHeight); |
|  | self.doc.height(hh); |
|  | } |
|  | self.lazyResize(30); |
|  |  |
|  | if (cap.isie7) self.css($(self.iframe.html), { |
|  | 'overflow-y': 'hidden' |
|  | }); |
|  | self.css($(self.iframe.body), { |
|  | 'overflow-y': 'hidden' |
|  | }); |
|  |  |
|  | if (cap.isios && self.haswrapper) { |
|  | self.css($(doc.body), { |
|  | '-webkit-transform': 'translate3d(0,0,0)' |
|  | }); // avoid iFrame content clipping - thanks to http://blog.derraab.com/2012/04/02/avoid-iframe-content-clipping-with-css-transform-on-ios/ |
|  | } |
|  |  |
|  | if ('contentWindow' in this) { |
|  | self.bind(this.contentWindow, "scroll", self.onscroll); //IE8 & minor |
|  | } else { |
|  | self.bind(doc, "scroll", self.onscroll); |
|  | } |
|  |  |
|  | if (self.opt.enablemousewheel) { |
|  | self.bind(doc, "mousewheel", self.onmousewheel); |
|  | } |
|  |  |
|  | if (self.opt.enablekeyboard) self.bind(doc, (cap.isopera) ? "keypress" : "keydown", self.onkeypress); |
|  |  |
|  | if (cap.cantouch \|\| self.opt.touchbehavior) { |
|  | self.bind(doc, "mousedown", self.ontouchstart); |
|  | self.bind(doc, "mousemove", function(e) { |
|  | return self.ontouchmove(e, true) |
|  | }); |
|  | if (self.opt.grabcursorenabled && cap.cursorgrabvalue) self.css($(doc.body), { |
|  | 'cursor': cap.cursorgrabvalue |
|  | }); |
|  | } |
|  |  |
|  | self.bind(doc, "mouseup", self.ontouchend); |
|  |  |
|  | if (self.zoom) { |
|  | if (self.opt.dblclickzoom) self.bind(doc, 'dblclick', self.doZoom); |
|  | if (self.ongesturezoom) self.bind(doc, "gestureend", self.ongesturezoom); |
|  | } |
|  | }; |
|  |  |
|  | if (this.doc[0].readyState && this.doc[0].readyState == "complete") { |
|  | setTimeout(function() { |
|  | oniframeload.call(self.doc[0], false) |
|  | }, 500); |
|  | } |
|  | self.bind(this.doc, "load", oniframeload); |
|  |  |
|  | } |
|  |  |
|  | }; |
|  |  |
|  | this.showCursor = function(py, px) { |
|  | if (self.cursortimeout) { |
|  | clearTimeout(self.cursortimeout); |
|  | self.cursortimeout = 0; |
|  | } |
|  | if (!self.rail) return; |
|  | if (self.autohidedom) { |
|  | self.autohidedom.stop().css({ |
|  | opacity: self.opt.cursoropacitymax |
|  | }); |
|  | self.cursoractive = true; |
|  | } |
|  |  |
|  | if (!self.rail.drag \|\| self.rail.drag.pt != 1) { |
|  | if ((typeof py != "undefined") && (py !== false)) { |
|  | self.scroll.y = Math.round(py \* 1 / self.scrollratio.y); |
|  | } |
|  | if (typeof px != "undefined") { |
|  | self.scroll.x = Math.round(px \* 1 / self.scrollratio.x); |
|  | } |
|  | } |
|  |  |
|  | self.cursor.css({ |
|  | height: self.cursorheight, |
|  | top: self.scroll.y |
|  | }); |
|  | if (self.cursorh) { |
|  | var lx = (self.hasreversehr) ? self.scrollvaluemaxw-self.scroll.x : self.scroll.x; |
|  | (!self.rail.align && self.rail.visibility) ? self.cursorh.css({ |
|  | width: self.cursorwidth, |
|  | left: lx + self.rail.width |
|  | }): self.cursorh.css({ |
|  | width: self.cursorwidth, |
|  | left: lx |
|  | }); |
|  | self.cursoractive = true; |
|  | } |
|  |  |
|  | if (self.zoom) self.zoom.stop().css({ |
|  | opacity: self.opt.cursoropacitymax |
|  | }); |
|  | }; |
|  |  |
|  | this.hideCursor = function(tm) { |
|  | if (self.cursortimeout) return; |
|  | if (!self.rail) return; |
|  | if (!self.autohidedom) return; |
|  | if (self.hasmousefocus && self.opt.autohidemode == "leave") return; |
|  | self.cursortimeout = setTimeout(function() { |
|  | if (!self.rail.active \|\| !self.showonmouseevent) { |
|  | self.autohidedom.stop().animate({ |
|  | opacity: self.opt.cursoropacitymin |
|  | }); |
|  | if (self.zoom) self.zoom.stop().animate({ |
|  | opacity: self.opt.cursoropacitymin |
|  | }); |
|  | self.cursoractive = false; |
|  | } |
|  | self.cursortimeout = 0; |
|  | }, tm \|\| self.opt.hidecursordelay); |
|  | }; |
|  |  |
|  | this.noticeCursor = function(tm, py, px) { |
|  | self.showCursor(py, px); |
|  | if (!self.rail.active) self.hideCursor(tm); |
|  | }; |
|  |  |
|  | this.getContentSize = |
|  | (self.ispage) ? |
|  | function() { |
|  | return { |
|  | w: Math.max(document.body.scrollWidth, document.documentElement.scrollWidth), |
|  | h: Math.max(document.body.scrollHeight, document.documentElement.scrollHeight) |
|  | } |
|  | } : (self.haswrapper) ? |
|  | function() { |
|  | return { |
|  | w: self.doc.outerWidth() + parseInt(self.win.css('paddingLeft')) + parseInt(self.win.css('paddingRight')), |
|  | h: self.doc.outerHeight() + parseInt(self.win.css('paddingTop')) + parseInt(self.win.css('paddingBottom')) |
|  | } |
|  | } : function() { |
|  | return { |
|  | w: self.docscroll[0].scrollWidth, |
|  | h: self.docscroll[0].scrollHeight |
|  | } |
|  | }; |
|  |  |
|  | this.onResize = function(e, page) { |
|  |  |
|  | if (!self \|\| !self.win) return false; |
|  |  |
|  | if (!self.haswrapper && !self.ispage) { |
|  | if (self.win.css('display') == 'none') { |
|  | if (self.visibility) self.hideRail().hideRailHr(); |
|  | return false; |
|  | } else { |
|  | if (!self.hidden && !self.visibility) self.showRail().showRailHr(); |
|  | } |
|  | } |
|  |  |
|  | var premaxh = self.page.maxh; |
|  | var premaxw = self.page.maxw; |
|  |  |
|  | var preview = { |
|  | h: self.view.h, |
|  | w: self.view.w |
|  | }; |
|  |  |
|  | self.view = { |
|  | w: (self.ispage) ? self.win.width() : parseInt(self.win[0].clientWidth), |
|  | h: (self.ispage) ? self.win.height() : parseInt(self.win[0].clientHeight) |
|  | }; |
|  |  |
|  | self.page = (page) ? page : self.getContentSize(); |
|  |  |
|  | self.page.maxh = Math.max(0, self.page.h - self.view.h); |
|  | self.page.maxw = Math.max(0, self.page.w - self.view.w); |
|  |  |
|  | if ((self.page.maxh == premaxh) && (self.page.maxw == premaxw) && (self.view.w == preview.w) && (self.view.h == preview.h)) { |
|  | // test position |
|  | if (!self.ispage) { |
|  | var pos = self.win.offset(); |
|  | if (self.lastposition) { |
|  | var lst = self.lastposition; |
|  | if ((lst.top == pos.top) && (lst.left == pos.left)) return self; //nothing to do |
|  | } |
|  | self.lastposition = pos; |
|  | } else { |
|  | return self; //nothing to do |
|  | } |
|  | } |
|  |  |
|  | if (self.page.maxh == 0) { |
|  | self.hideRail(); |
|  | self.scrollvaluemax = 0; |
|  | self.scroll.y = 0; |
|  | self.scrollratio.y = 0; |
|  | self.cursorheight = 0; |
|  | self.setScrollTop(0); |
|  | self.rail.scrollable = false; |
|  | } else { |
|  | self.page.maxh -= (self.opt.railpadding.top + self.opt.railpadding.bottom); //\*\* |
|  | self.rail.scrollable = true; |
|  | } |
|  |  |
|  | if (self.page.maxw == 0) { |
|  | self.hideRailHr(); |
|  | self.scrollvaluemaxw = 0; |
|  | self.scroll.x = 0; |
|  | self.scrollratio.x = 0; |
|  | self.cursorwidth = 0; |
|  | self.setScrollLeft(0); |
|  | self.railh.scrollable = false; |
|  | } else { |
|  | self.page.maxw -= (self.opt.railpadding.left + self.opt.railpadding.right); //\*\* |
|  | self.railh.scrollable = true; |
|  | } |
|  |  |
|  | self.railslocked = (self.locked) \|\| ((self.page.maxh == 0) && (self.page.maxw == 0)); |
|  | if (self.railslocked) { |
|  | if (!self.ispage) self.updateScrollBar(self.view); |
|  | return false; |
|  | } |
|  |  |
|  | if (!self.hidden && !self.visibility) { |
|  | self.showRail().showRailHr(); |
|  | } |
|  | else if (!self.hidden && !self.railh.visibility) self.showRailHr(); |
|  |  |
|  | if (self.istextarea && self.win.css('resize') && self.win.css('resize') != 'none') self.view.h -= 20; |
|  |  |
|  | self.cursorheight = Math.min(self.view.h, Math.round(self.view.h \* (self.view.h / self.page.h))); |
|  | self.cursorheight = (self.opt.cursorfixedheight) ? self.opt.cursorfixedheight : Math.max(self.opt.cursorminheight, self.cursorheight); |
|  |  |
|  | self.cursorwidth = Math.min(self.view.w, Math.round(self.view.w \* (self.view.w / self.page.w))); |
|  | self.cursorwidth = (self.opt.cursorfixedheight) ? self.opt.cursorfixedheight : Math.max(self.opt.cursorminheight, self.cursorwidth); |
|  |  |
|  | self.scrollvaluemax = self.view.h - self.cursorheight - self.cursor.hborder - (self.opt.railpadding.top + self.opt.railpadding.bottom); //\*\* |
|  |  |
|  | if (self.railh) { |
|  | self.railh.width = (self.page.maxh > 0) ? (self.view.w - self.rail.width) : self.view.w; |
|  | self.scrollvaluemaxw = self.railh.width - self.cursorwidth - self.cursorh.wborder - (self.opt.railpadding.left + self.opt.railpadding.right); //\*\* |
|  | } |
|  |  |
|  | /\* |
|  | if (self.checkrtlmode&&self.railh) { |
|  | self.checkrtlmode = false; |
|  | if (self.opt.rtlmode&&self.scroll.x==0) self.setScrollLeft(self.page.maxw); |
|  | } |
|  | \*/ |
|  |  |
|  | if (!self.ispage) self.updateScrollBar(self.view); |
|  |  |
|  | self.scrollratio = { |
|  | x: (self.page.maxw / self.scrollvaluemaxw), |
|  | y: (self.page.maxh / self.scrollvaluemax) |
|  | }; |
|  |  |
|  | var sy = self.getScrollTop(); |
|  | if (sy > self.page.maxh) { |
|  | self.doScrollTop(self.page.maxh); |
|  | } else { |
|  | self.scroll.y = Math.round(self.getScrollTop() \* (1 / self.scrollratio.y)); |
|  | self.scroll.x = Math.round(self.getScrollLeft() \* (1 / self.scrollratio.x)); |
|  | if (self.cursoractive) self.noticeCursor(); |
|  | } |
|  |  |
|  | if (self.scroll.y && (self.getScrollTop() == 0)) self.doScrollTo(Math.floor(self.scroll.y \* self.scrollratio.y)); |
|  |  |
|  | return self; |
|  | }; |
|  |  |
|  | this.resize = self.onResize; |
|  |  |
|  | this.lazyResize = function(tm) { // event debounce |
|  | tm = (isNaN(tm)) ? 30 : tm; |
|  | self.debounced('resize', self.resize, tm); |
|  | return self; |
|  | }; |
|  |  |
|  | // modified by MDN https://developer.mozilla.org/en-US/docs/DOM/Mozilla\_event\_reference/wheel |
|  | function \_modernWheelEvent(dom, name, fn, bubble) { |
|  | self.\_bind(dom, name, function(e) { |
|  | var e = (e) ? e : window.event; |
|  | var event = { |
|  | original: e, |
|  | target: e.target \|\| e.srcElement, |
|  | type: "wheel", |
|  | deltaMode: e.type == "MozMousePixelScroll" ? 0 : 1, |
|  | deltaX: 0, |
|  | deltaZ: 0, |
|  | preventDefault: function() { |
|  | e.preventDefault ? e.preventDefault() : e.returnValue = false; |
|  | return false; |
|  | }, |
|  | stopImmediatePropagation: function() { |
|  | (e.stopImmediatePropagation) ? e.stopImmediatePropagation(): e.cancelBubble = true; |
|  | } |
|  | }; |
|  |  |
|  | if (name == "mousewheel") { |
|  | event.deltaY = -1 / 40 \* e.wheelDelta; |
|  | e.wheelDeltaX && (event.deltaX = -1 / 40 \* e.wheelDeltaX); |
|  | } else { |
|  | event.deltaY = e.detail; |
|  | } |
|  |  |
|  | return fn.call(dom, event); |
|  | }, bubble); |
|  | }; |
|  |  |
|  |  |
|  |  |
|  | this.jqbind = function(dom, name, fn) { // use jquery bind for non-native events (mouseenter/mouseleave) |
|  | self.events.push({ |
|  | e: dom, |
|  | n: name, |
|  | f: fn, |
|  | q: true |
|  | }); |
|  | $(dom).bind(name, fn); |
|  | }; |
|  |  |
|  | this.bind = function(dom, name, fn, bubble) { // touch-oriented & fixing jquery bind |
|  | var el = ("jquery" in dom) ? dom[0] : dom; |
|  |  |
|  | if (name == 'mousewheel') { |
|  | if (window.addEventListener\|\|'onwheel' in document) { // modern brosers & IE9 detection fix |
|  | self.\_bind(el, "wheel", fn, bubble \|\| false); |
|  | } else { |
|  | var wname = (typeof document.onmousewheel != "undefined") ? "mousewheel" : "DOMMouseScroll"; // older IE/Firefox |
|  | \_modernWheelEvent(el, wname, fn, bubble \|\| false); |
|  | if (wname == "DOMMouseScroll") \_modernWheelEvent(el, "MozMousePixelScroll", fn, bubble \|\| false); // Firefox legacy |
|  | } |
|  | } else if (el.addEventListener) { |
|  | if (cap.cantouch && /mouseup\|mousedown\|mousemove/.test(name)) { // touch device support |
|  | var tt = (name == 'mousedown') ? 'touchstart' : (name == 'mouseup') ? 'touchend' : 'touchmove'; |
|  | self.\_bind(el, tt, function(e) { |
|  | if (e.touches) { |
|  | if (e.touches.length < 2) { |
|  | var ev = (e.touches.length) ? e.touches[0] : e; |
|  | ev.original = e; |
|  | fn.call(this, ev); |
|  | } |
|  | } else if (e.changedTouches) { |
|  | var ev = e.changedTouches[0]; |
|  | ev.original = e; |
|  | fn.call(this, ev); |
|  | } //blackberry |
|  | }, bubble \|\| false); |
|  | } |
|  | self.\_bind(el, name, fn, bubble \|\| false); |
|  | if (cap.cantouch && name == "mouseup") self.\_bind(el, "touchcancel", fn, bubble \|\| false); |
|  | } else { |
|  | self.\_bind(el, name, function(e) { |
|  | e = e \|\| window.event \|\| false; |
|  | if (e) { |
|  | if (e.srcElement) e.target = e.srcElement; |
|  | } |
|  | if (!("pageY" in e)) { |
|  | e.pageX = e.clientX + document.documentElement.scrollLeft; |
|  | e.pageY = e.clientY + document.documentElement.scrollTop; |
|  | } |
|  | return ((fn.call(el, e) === false) \|\| bubble === false) ? self.cancelEvent(e) : true; |
|  | }); |
|  | } |
|  | }; |
|  |  |
|  | if (cap.haseventlistener) { // W3C standard model |
|  | this.\_bind = function(el, name, fn, bubble) { // primitive bind |
|  | self.events.push({ |
|  | e: el, |
|  | n: name, |
|  | f: fn, |
|  | b: bubble, |
|  | q: false |
|  | }); |
|  | el.addEventListener(name, fn, bubble \|\| false); |
|  | }; |
|  | this.cancelEvent = function(e) { |
|  | if (!e) return false; |
|  | var e = (e.original) ? e.original : e; |
|  | e.preventDefault(); |
|  | e.stopPropagation(); |
|  | if (e.preventManipulation) e.preventManipulation(); //IE10 |
|  | return false; |
|  | }; |
|  | this.stopPropagation = function(e) { |
|  | if (!e) return false; |
|  | var e = (e.original) ? e.original : e; |
|  | e.stopPropagation(); |
|  | return false; |
|  | }; |
|  | this.\_unbind = function(el, name, fn, bub) { // primitive unbind |
|  | el.removeEventListener(name, fn, bub); |
|  | }; |
|  | } else { // old IE model |
|  | this.\_bind = function(el, name, fn, bubble) { // primitive bind |
|  | self.events.push({ |
|  | e: el, |
|  | n: name, |
|  | f: fn, |
|  | b: bubble, |
|  | q: false |
|  | }); |
|  | if (el.attachEvent) { |
|  | el.attachEvent("on" + name, fn); |
|  | } else { |
|  | el["on" + name] = fn; |
|  | } |
|  | }; |
|  | // Thanks to http://www.switchonthecode.com !! |
|  | this.cancelEvent = function(e) { |
|  | var e = window.event \|\| false; |
|  | if (!e) return false; |
|  | e.cancelBubble = true; |
|  | e.cancel = true; |
|  | e.returnValue = false; |
|  | return false; |
|  | }; |
|  | this.stopPropagation = function(e) { |
|  | var e = window.event \|\| false; |
|  | if (!e) return false; |
|  | e.cancelBubble = true; |
|  | return false; |
|  | }; |
|  | this.\_unbind = function(el, name, fn, bub) { // primitive unbind IE old |
|  | if (el.detachEvent) { |
|  | el.detachEvent('on' + name, fn); |
|  | } else { |
|  | el['on' + name] = false; |
|  | } |
|  | }; |
|  | } |
|  |  |
|  | this.unbindAll = function() { |
|  | for (var a = 0; a < self.events.length; a++) { |
|  | var r = self.events[a]; |
|  | (r.q) ? r.e.unbind(r.n, r.f): self.\_unbind(r.e, r.n, r.f, r.b); |
|  | } |
|  | }; |
|  |  |
|  | this.showRail = function() { |
|  | if ((self.page.maxh != 0) && (self.ispage \|\| self.win.css('display') != 'none')) { |
|  | self.visibility = true; |
|  | self.rail.visibility = true; |
|  | self.rail.css('display', 'block'); |
|  | } |
|  | return self; |
|  | }; |
|  |  |
|  | this.showRailHr = function() { |
|  | if (!self.railh) return self; |
|  | if ((self.page.maxw != 0) && (self.ispage \|\| self.win.css('display') != 'none')) { |
|  | self.railh.visibility = true; |
|  | self.railh.css('display', 'block'); |
|  | } |
|  | return self; |
|  | }; |
|  |  |
|  | this.hideRail = function() { |
|  | self.visibility = false; |
|  | self.rail.visibility = false; |
|  | self.rail.css('display', 'none'); |
|  | return self; |
|  | }; |
|  |  |
|  | this.hideRailHr = function() { |
|  | if (!self.railh) return self; |
|  | self.railh.visibility = false; |
|  | self.railh.css('display', 'none'); |
|  | return self; |
|  | }; |
|  |  |
|  | this.show = function() { |
|  | self.hidden = false; |
|  | self.railslocked = false; |
|  | return self.showRail().showRailHr(); |
|  | }; |
|  |  |
|  | this.hide = function() { |
|  | self.hidden = true; |
|  | self.railslocked = true; |
|  | return self.hideRail().hideRailHr(); |
|  | }; |
|  |  |
|  | this.toggle = function() { |
|  | return (self.hidden) ? self.show() : self.hide(); |
|  | }; |
|  |  |
|  | this.remove = function() { |
|  | self.stop(); |
|  | if (self.cursortimeout) clearTimeout(self.cursortimeout); |
|  | self.doZoomOut(); |
|  | self.unbindAll(); |
|  |  |
|  | if (cap.isie9) self.win[0].detachEvent("onpropertychange", self.onAttributeChange); //IE9 DOMAttrModified bug |
|  |  |
|  | if (self.observer !== false) self.observer.disconnect(); |
|  | if (self.observerremover !== false) self.observerremover.disconnect(); |
|  | if (self.observerbody !== false) self.observerbody.disconnect(); |
|  |  |
|  | self.events = null; |
|  |  |
|  | if (self.cursor) { |
|  | self.cursor.remove(); |
|  | } |
|  | if (self.cursorh) { |
|  | self.cursorh.remove(); |
|  | } |
|  | if (self.rail) { |
|  | self.rail.remove(); |
|  | } |
|  | if (self.railh) { |
|  | self.railh.remove(); |
|  | } |
|  | if (self.zoom) { |
|  | self.zoom.remove(); |
|  | } |
|  | for (var a = 0; a < self.saved.css.length; a++) { |
|  | var d = self.saved.css[a]; |
|  | d[0].css(d[1], (typeof d[2] == "undefined") ? '' : d[2]); |
|  | } |
|  | self.saved = false; |
|  | self.me.data('\_\_nicescroll', ''); //erase all traces |
|  |  |
|  | // memory leak fixed by GianlucaGuarini - thanks a lot! |
|  | // remove the current nicescroll from the $.nicescroll array & normalize array |
|  | var lst = $.nicescroll; |
|  | lst.each(function(i) { |
|  | if (!this) return; |
|  | if (this.id === self.id) { |
|  | delete lst[i]; |
|  | for (var b = ++i; b < lst.length; b++, i++) lst[i] = lst[b]; |
|  | lst.length--; |
|  | if (lst.length) delete lst[lst.length]; |
|  | } |
|  | }); |
|  |  |
|  | for (var i in self) { |
|  | self[i] = null; |
|  | delete self[i]; |
|  | } |
|  |  |
|  | self = null; |
|  |  |
|  | }; |
|  |  |
|  | this.scrollstart = function(fn) { |
|  | this.onscrollstart = fn; |
|  | return self; |
|  | }; |
|  | this.scrollend = function(fn) { |
|  | this.onscrollend = fn; |
|  | return self; |
|  | }; |
|  | this.scrollcancel = function(fn) { |
|  | this.onscrollcancel = fn; |
|  | return self; |
|  | }; |
|  |  |
|  | this.zoomin = function(fn) { |
|  | this.onzoomin = fn; |
|  | return self; |
|  | }; |
|  | this.zoomout = function(fn) { |
|  | this.onzoomout = fn; |
|  | return self; |
|  | }; |
|  |  |
|  | this.isScrollable = function(e) { |
|  | var dom = (e.target) ? e.target : e; |
|  | if (dom.nodeName == 'OPTION') return true; |
|  | while (dom && (dom.nodeType == 1) && !(/^BODY\|HTML/.test(dom.nodeName))) { |
|  | var dd = $(dom); |
|  | var ov = dd.css('overflowY') \|\| dd.css('overflowX') \|\| dd.css('overflow') \|\| ''; |
|  | if (/scroll\|auto/.test(ov)) return (dom.clientHeight != dom.scrollHeight); |
|  | dom = (dom.parentNode) ? dom.parentNode : false; |
|  | } |
|  | return false; |
|  | }; |
|  |  |
|  | this.getViewport = function(me) { |
|  | var dom = (me && me.parentNode) ? me.parentNode : false; |
|  | while (dom && (dom.nodeType == 1) && !(/^BODY\|HTML/.test(dom.nodeName))) { |
|  | var dd = $(dom); |
|  | if (/fixed\|absolute/.test(dd.css("position"))) return dd; |
|  | var ov = dd.css('overflowY') \|\| dd.css('overflowX') \|\| dd.css('overflow') \|\| ''; |
|  | if ((/scroll\|auto/.test(ov)) && (dom.clientHeight != dom.scrollHeight)) return dd; |
|  | if (dd.getNiceScroll().length > 0) return dd; |
|  | dom = (dom.parentNode) ? dom.parentNode : false; |
|  | } |
|  | return false; //(dom) ? $(dom) : false; |
|  | }; |
|  |  |
|  | this.triggerScrollEnd = function() { |
|  | if (!self.onscrollend) return; |
|  |  |
|  | var px = self.getScrollLeft(); |
|  | var py = self.getScrollTop(); |
|  |  |
|  | var info = { |
|  | "type": "scrollend", |
|  | "current": { |
|  | "x": px, |
|  | "y": py |
|  | }, |
|  | "end": { |
|  | "x": px, |
|  | "y": py |
|  | } |
|  | }; |
|  | self.onscrollend.call(self, info); |
|  | } |
|  |  |
|  | function execScrollWheel(e, hr, chkscroll) { |
|  | var px, py; |
|  |  |
|  | if (e.deltaMode == 0) { // PIXEL |
|  | px = -Math.floor(e.deltaX \* (self.opt.mousescrollstep / (18 \* 3))); |
|  | py = -Math.floor(e.deltaY \* (self.opt.mousescrollstep / (18 \* 3))); |
|  | } else if (e.deltaMode == 1) { // LINE |
|  | px = -Math.floor(e.deltaX \* self.opt.mousescrollstep); |
|  | py = -Math.floor(e.deltaY \* self.opt.mousescrollstep); |
|  | } |
|  |  |
|  | if (hr && self.opt.oneaxismousemode && (px == 0) && py) { // classic vertical-only mousewheel + browser with x/y support |
|  | px = py; |
|  | py = 0; |
|  |  |
|  | if (chkscroll) { |
|  | var hrend = (px < 0) ? (self.getScrollLeft() >= self.page.maxw) : (self.getScrollLeft() <= 0); |
|  | if (hrend) { // preserve vertical scrolling |
|  | py = px; |
|  | px = 0; |
|  | } |
|  | } |
|  |  |
|  | } |
|  |  |
|  | if (px) { |
|  | if (self.scrollmom) { |
|  | self.scrollmom.stop() |
|  | } |
|  | self.lastdeltax += px; |
|  | self.debounced("mousewheelx", function() { |
|  | var dt = self.lastdeltax; |
|  | self.lastdeltax = 0; |
|  | if (!self.rail.drag) { |
|  | self.doScrollLeftBy(dt) |
|  | } |
|  | }, 15); |
|  | } |
|  | if (py) { |
|  | if (self.opt.nativeparentscrolling && chkscroll && !self.ispage && !self.zoomactive) { |
|  | if (py < 0) { |
|  | if (self.getScrollTop() >= self.page.maxh) return true; |
|  | } else { |
|  | if (self.getScrollTop() <= 0) return true; |
|  | } |
|  | } |
|  | if (self.scrollmom) { |
|  | self.scrollmom.stop() |
|  | } |
|  | self.lastdeltay += py; |
|  | self.debounced("mousewheely", function() { |
|  | var dt = self.lastdeltay; |
|  | self.lastdeltay = 0; |
|  | if (!self.rail.drag) { |
|  | self.doScrollBy(dt) |
|  | } |
|  | }, 15); |
|  | } |
|  |  |
|  | e.stopImmediatePropagation(); |
|  | return e.preventDefault(); |
|  | }; |
|  |  |
|  | this.onmousewheel = function(e) { |
|  | if (self.wheelprevented) return; |
|  | if (self.railslocked) { |
|  | self.debounced("checkunlock", self.resize, 250); |
|  | return true; |
|  | } |
|  | if (self.rail.drag) return self.cancelEvent(e); |
|  |  |
|  | if (self.opt.oneaxismousemode == "auto" && e.deltaX != 0) self.opt.oneaxismousemode = false; // check two-axis mouse support (not very elegant) |
|  |  |
|  | if (self.opt.oneaxismousemode && e.deltaX == 0) { |
|  | if (!self.rail.scrollable) { |
|  | if (self.railh && self.railh.scrollable) { |
|  | return self.onmousewheelhr(e); |
|  | } else { |
|  | return true; |
|  | } |
|  | } |
|  | } |
|  |  |
|  | var nw = +(new Date()); |
|  | var chk = false; |
|  | if (self.opt.preservenativescrolling && ((self.checkarea + 600) < nw)) { |
|  | self.nativescrollingarea = self.isScrollable(e); |
|  | chk = true; |
|  | } |
|  | self.checkarea = nw; |
|  | if (self.nativescrollingarea) return true; // this isn't my business |
|  | var ret = execScrollWheel(e, false, chk); |
|  | if (ret) self.checkarea = 0; |
|  | return ret; |
|  | }; |
|  |  |
|  | this.onmousewheelhr = function(e) { |
|  | if (self.wheelprevented) return; |
|  | if (self.railslocked \|\| !self.railh.scrollable) return true; |
|  | if (self.rail.drag) return self.cancelEvent(e); |
|  |  |
|  | var nw = +(new Date()); |
|  | var chk = false; |
|  | if (self.opt.preservenativescrolling && ((self.checkarea + 600) < nw)) { |
|  | self.nativescrollingarea = self.isScrollable(e); |
|  | chk = true; |
|  | } |
|  | self.checkarea = nw; |
|  | if (self.nativescrollingarea) return true; // this isn't my business |
|  | if (self.railslocked) return self.cancelEvent(e); |
|  |  |
|  | return execScrollWheel(e, true, chk); |
|  | }; |
|  |  |
|  | this.stop = function() { |
|  | self.cancelScroll(); |
|  | if (self.scrollmon) self.scrollmon.stop(); |
|  | self.cursorfreezed = false; |
|  | self.scroll.y = Math.round(self.getScrollTop() \* (1 / self.scrollratio.y)); |
|  | self.noticeCursor(); |
|  | return self; |
|  | }; |
|  |  |
|  | this.getTransitionSpeed = function(dif) { |
|  | var sp = Math.round(self.opt.scrollspeed \* 10); |
|  | var ex = Math.min(sp, Math.round((dif / 20) \* self.opt.scrollspeed)); |
|  | return (ex > 20) ? ex : 0; |
|  | }; |
|  |  |
|  | if (!self.opt.smoothscroll) { |
|  | this.doScrollLeft = function(x, spd) { //direct |
|  | var y = self.getScrollTop(); |
|  | self.doScrollPos(x, y, spd); |
|  | }; |
|  | this.doScrollTop = function(y, spd) { //direct |
|  | var x = self.getScrollLeft(); |
|  | self.doScrollPos(x, y, spd); |
|  | }; |
|  | this.doScrollPos = function(x, y, spd) { //direct |
|  | var nx = (x > self.page.maxw) ? self.page.maxw : x; |
|  | if (nx < 0) nx = 0; |
|  | var ny = (y > self.page.maxh) ? self.page.maxh : y; |
|  | if (ny < 0) ny = 0; |
|  | self.synched('scroll', function() { |
|  | self.setScrollTop(ny); |
|  | self.setScrollLeft(nx); |
|  | }); |
|  | }; |
|  | this.cancelScroll = function() {}; // direct |
|  | } else if (self.ishwscroll && cap.hastransition && self.opt.usetransition && !!self.opt.smoothscroll) { |
|  | this.prepareTransition = function(dif, istime) { |
|  | var ex = (istime) ? ((dif > 20) ? dif : 0) : self.getTransitionSpeed(dif); |
|  | var trans = (ex) ? cap.prefixstyle + 'transform ' + ex + 'ms ease-out' : ''; |
|  | if (!self.lasttransitionstyle \|\| self.lasttransitionstyle != trans) { |
|  | self.lasttransitionstyle = trans; |
|  | self.doc.css(cap.transitionstyle, trans); |
|  | } |
|  | return ex; |
|  | }; |
|  |  |
|  | this.doScrollLeft = function(x, spd) { //trans |
|  | var y = (self.scrollrunning) ? self.newscrolly : self.getScrollTop(); |
|  | self.doScrollPos(x, y, spd); |
|  | }; |
|  |  |
|  | this.doScrollTop = function(y, spd) { //trans |
|  | var x = (self.scrollrunning) ? self.newscrollx : self.getScrollLeft(); |
|  | self.doScrollPos(x, y, spd); |
|  | }; |
|  |  |
|  | this.doScrollPos = function(x, y, spd) { //trans |
|  |  |
|  | var py = self.getScrollTop(); |
|  | var px = self.getScrollLeft(); |
|  |  |
|  | if (((self.newscrolly - py) \* (y - py) < 0) \|\| ((self.newscrollx - px) \* (x - px) < 0)) self.cancelScroll(); //inverted movement detection |
|  |  |
|  | if (self.opt.bouncescroll == false) { |
|  | if (y < 0) y = 0; |
|  | else if (y > self.page.maxh) y = self.page.maxh; |
|  | if (x < 0) x = 0; |
|  | else if (x > self.page.maxw) x = self.page.maxw; |
|  | } |
|  |  |
|  | if (self.scrollrunning && x == self.newscrollx && y == self.newscrolly) return false; |
|  |  |
|  | self.newscrolly = y; |
|  | self.newscrollx = x; |
|  |  |
|  | self.newscrollspeed = spd \|\| false; |
|  |  |
|  | if (self.timer) return false; |
|  |  |
|  | self.timer = setTimeout(function() { |
|  |  |
|  | var top = self.getScrollTop(); |
|  | var lft = self.getScrollLeft(); |
|  |  |
|  | var dst = {}; |
|  | dst.x = x - lft; |
|  | dst.y = y - top; |
|  | dst.px = lft; |
|  | dst.py = top; |
|  |  |
|  | var dd = Math.round(Math.sqrt(Math.pow(dst.x, 2) + Math.pow(dst.y, 2))); |
|  | var ms = (self.newscrollspeed && self.newscrollspeed > 1) ? self.newscrollspeed : self.getTransitionSpeed(dd); |
|  | if (self.newscrollspeed && self.newscrollspeed <= 1) ms \*= self.newscrollspeed; |
|  |  |
|  | self.prepareTransition(ms, true); |
|  |  |
|  | if (self.timerscroll && self.timerscroll.tm) clearInterval(self.timerscroll.tm); |
|  |  |
|  | if (ms > 0) { |
|  |  |
|  | if (!self.scrollrunning && self.onscrollstart) { |
|  | var info = { |
|  | "type": "scrollstart", |
|  | "current": { |
|  | "x": lft, |
|  | "y": top |
|  | }, |
|  | "request": { |
|  | "x": x, |
|  | "y": y |
|  | }, |
|  | "end": { |
|  | "x": self.newscrollx, |
|  | "y": self.newscrolly |
|  | }, |
|  | "speed": ms |
|  | }; |
|  | self.onscrollstart.call(self, info); |
|  | } |
|  |  |
|  | if (cap.transitionend) { |
|  | if (!self.scrollendtrapped) { |
|  | self.scrollendtrapped = true; |
|  | self.bind(self.doc, cap.transitionend, self.onScrollTransitionEnd, false); //I have got to do something usefull!! |
|  | } |
|  | } else { |
|  | if (self.scrollendtrapped) clearTimeout(self.scrollendtrapped); |
|  | self.scrollendtrapped = setTimeout(self.onScrollTransitionEnd, ms); // simulate transitionend event |
|  | } |
|  |  |
|  | var py = top; |
|  | var px = lft; |
|  | self.timerscroll = { |
|  | bz: new BezierClass(py, self.newscrolly, ms, 0, 0, 0.58, 1), |
|  | bh: new BezierClass(px, self.newscrollx, ms, 0, 0, 0.58, 1) |
|  | }; |
|  | if (!self.cursorfreezed) self.timerscroll.tm = setInterval(function() { |
|  | self.showCursor(self.getScrollTop(), self.getScrollLeft()) |
|  | }, 60); |
|  |  |
|  | } |
|  |  |
|  | self.synched("doScroll-set", function() { |
|  | self.timer = 0; |
|  | if (self.scrollendtrapped) self.scrollrunning = true; |
|  | self.setScrollTop(self.newscrolly); |
|  | self.setScrollLeft(self.newscrollx); |
|  | if (!self.scrollendtrapped) self.onScrollTransitionEnd(); |
|  | }); |
|  |  |
|  |  |
|  | }, 50); |
|  |  |
|  | }; |
|  |  |
|  | this.cancelScroll = function() { |
|  | if (!self.scrollendtrapped) return true; |
|  | var py = self.getScrollTop(); |
|  | var px = self.getScrollLeft(); |
|  | self.scrollrunning = false; |
|  | if (!cap.transitionend) clearTimeout(cap.transitionend); |
|  | self.scrollendtrapped = false; |
|  | self.\_unbind(self.doc[0], cap.transitionend, self.onScrollTransitionEnd); |
|  | self.prepareTransition(0); |
|  | self.setScrollTop(py); // fire event onscroll |
|  | if (self.railh) self.setScrollLeft(px); |
|  | if (self.timerscroll && self.timerscroll.tm) clearInterval(self.timerscroll.tm); |
|  | self.timerscroll = false; |
|  |  |
|  | self.cursorfreezed = false; |
|  |  |
|  | self.showCursor(py, px); |
|  | return self; |
|  | }; |
|  | this.onScrollTransitionEnd = function() { |
|  | if (self.scrollendtrapped) self.\_unbind(self.doc[0], cap.transitionend, self.onScrollTransitionEnd); |
|  | self.scrollendtrapped = false; |
|  | self.prepareTransition(0); |
|  | if (self.timerscroll && self.timerscroll.tm) clearInterval(self.timerscroll.tm); |
|  | self.timerscroll = false; |
|  | var py = self.getScrollTop(); |
|  | var px = self.getScrollLeft(); |
|  | self.setScrollTop(py); // fire event onscroll |
|  | if (self.railh) self.setScrollLeft(px); // fire event onscroll left |
|  |  |
|  | self.noticeCursor(false, py, px); |
|  |  |
|  | self.cursorfreezed = false; |
|  |  |
|  | if (py < 0) py = 0 |
|  | else if (py > self.page.maxh) py = self.page.maxh; |
|  | if (px < 0) px = 0 |
|  | else if (px > self.page.maxw) px = self.page.maxw; |
|  | if ((py != self.newscrolly) \|\| (px != self.newscrollx)) return self.doScrollPos(px, py, self.opt.snapbackspeed); |
|  |  |
|  | if (self.onscrollend && self.scrollrunning) { |
|  | self.triggerScrollEnd(); |
|  | } |
|  | self.scrollrunning = false; |
|  |  |
|  | }; |
|  |  |
|  | } else { |
|  |  |
|  | this.doScrollLeft = function(x, spd) { //no-trans |
|  | var y = (self.scrollrunning) ? self.newscrolly : self.getScrollTop(); |
|  | self.doScrollPos(x, y, spd); |
|  | }; |
|  |  |
|  | this.doScrollTop = function(y, spd) { //no-trans |
|  | var x = (self.scrollrunning) ? self.newscrollx : self.getScrollLeft(); |
|  | self.doScrollPos(x, y, spd); |
|  | }; |
|  |  |
|  | this.doScrollPos = function(x, y, spd) { //no-trans |
|  | var y = ((typeof y == "undefined") \|\| (y === false)) ? self.getScrollTop(true) : y; |
|  |  |
|  | if ((self.timer) && (self.newscrolly == y) && (self.newscrollx == x)) return true; |
|  |  |
|  | if (self.timer) clearAnimationFrame(self.timer); |
|  | self.timer = 0; |
|  |  |
|  | var py = self.getScrollTop(); |
|  | var px = self.getScrollLeft(); |
|  |  |
|  | if (((self.newscrolly - py) \* (y - py) < 0) \|\| ((self.newscrollx - px) \* (x - px) < 0)) self.cancelScroll(); //inverted movement detection |
|  |  |
|  | self.newscrolly = y; |
|  | self.newscrollx = x; |
|  |  |
|  | if (!self.bouncescroll \|\| !self.rail.visibility) { |
|  | if (self.newscrolly < 0) { |
|  | self.newscrolly = 0; |
|  | } else if (self.newscrolly > self.page.maxh) { |
|  | self.newscrolly = self.page.maxh; |
|  | } |
|  | } |
|  | if (!self.bouncescroll \|\| !self.railh.visibility) { |
|  | if (self.newscrollx < 0) { |
|  | self.newscrollx = 0; |
|  | } else if (self.newscrollx > self.page.maxw) { |
|  | self.newscrollx = self.page.maxw; |
|  | } |
|  | } |
|  |  |
|  | self.dst = {}; |
|  | self.dst.x = x - px; |
|  | self.dst.y = y - py; |
|  | self.dst.px = px; |
|  | self.dst.py = py; |
|  |  |
|  | var dst = Math.round(Math.sqrt(Math.pow(self.dst.x, 2) + Math.pow(self.dst.y, 2))); |
|  |  |
|  | self.dst.ax = self.dst.x / dst; |
|  | self.dst.ay = self.dst.y / dst; |
|  |  |
|  | var pa = 0; |
|  | var pe = dst; |
|  |  |
|  | if (self.dst.x == 0) { |
|  | pa = py; |
|  | pe = y; |
|  | self.dst.ay = 1; |
|  | self.dst.py = 0; |
|  | } else if (self.dst.y == 0) { |
|  | pa = px; |
|  | pe = x; |
|  | self.dst.ax = 1; |
|  | self.dst.px = 0; |
|  | } |
|  |  |
|  | var ms = self.getTransitionSpeed(dst); |
|  | if (spd && spd <= 1) ms \*= spd; |
|  | if (ms > 0) { |
|  | self.bzscroll = (self.bzscroll) ? self.bzscroll.update(pe, ms) : new BezierClass(pa, pe, ms, 0, 1, 0, 1); |
|  | } else { |
|  | self.bzscroll = false; |
|  | } |
|  |  |
|  | if (self.timer) return; |
|  |  |
|  | if ((py == self.page.maxh && y >= self.page.maxh) \|\| (px == self.page.maxw && x >= self.page.maxw)) self.checkContentSize(); |
|  |  |
|  | var sync = 1; |
|  |  |
|  | function scrolling() { |
|  | if (self.cancelAnimationFrame) return true; |
|  |  |
|  | self.scrollrunning = true; |
|  |  |
|  | sync = 1 - sync; |
|  | if (sync) return (self.timer = setAnimationFrame(scrolling) \|\| 1); |
|  |  |
|  | var done = 0; |
|  | var sx, sy; |
|  |  |
|  | var sc = sy = self.getScrollTop(); |
|  | if (self.dst.ay) { |
|  | sc = (self.bzscroll) ? self.dst.py + (self.bzscroll.getNow() \* self.dst.ay) : self.newscrolly; |
|  | var dr = sc - sy; |
|  | if ((dr < 0 && sc < self.newscrolly) \|\| (dr > 0 && sc > self.newscrolly)) sc = self.newscrolly; |
|  | self.setScrollTop(sc); |
|  | if (sc == self.newscrolly) done = 1; |
|  | } else { |
|  | done = 1; |
|  | } |
|  |  |
|  | var scx = sx = self.getScrollLeft(); |
|  | if (self.dst.ax) { |
|  | scx = (self.bzscroll) ? self.dst.px + (self.bzscroll.getNow() \* self.dst.ax) : self.newscrollx; |
|  | var dr = scx - sx; |
|  | if ((dr < 0 && scx < self.newscrollx) \|\| (dr > 0 && scx > self.newscrollx)) scx = self.newscrollx; |
|  | self.setScrollLeft(scx); |
|  | if (scx == self.newscrollx) done += 1; |
|  | } else { |
|  | done += 1; |
|  | } |
|  |  |
|  | if (done == 2) { |
|  | self.timer = 0; |
|  | self.cursorfreezed = false; |
|  | self.bzscroll = false; |
|  | self.scrollrunning = false; |
|  | if (sc < 0) sc = 0; |
|  | else if (sc > self.page.maxh) sc = self.page.maxh; |
|  | if (scx < 0) scx = 0; |
|  | else if (scx > self.page.maxw) scx = self.page.maxw; |
|  | if ((scx != self.newscrollx) \|\| (sc != self.newscrolly)) self.doScrollPos(scx, sc); |
|  | else { |
|  | if (self.onscrollend) { |
|  | self.triggerScrollEnd(); |
|  | } |
|  | } |
|  | } else { |
|  | self.timer = setAnimationFrame(scrolling) \|\| 1; |
|  | } |
|  | }; |
|  | self.cancelAnimationFrame = false; |
|  | self.timer = 1; |
|  |  |
|  | if (self.onscrollstart && !self.scrollrunning) { |
|  | var info = { |
|  | "type": "scrollstart", |
|  | "current": { |
|  | "x": px, |
|  | "y": py |
|  | }, |
|  | "request": { |
|  | "x": x, |
|  | "y": y |
|  | }, |
|  | "end": { |
|  | "x": self.newscrollx, |
|  | "y": self.newscrolly |
|  | }, |
|  | "speed": ms |
|  | }; |
|  | self.onscrollstart.call(self, info); |
|  | } |
|  |  |
|  | scrolling(); |
|  |  |
|  | if ((py == self.page.maxh && y >= py) \|\| (px == self.page.maxw && x >= px)) self.checkContentSize(); |
|  |  |
|  | self.noticeCursor(); |
|  | }; |
|  |  |
|  | this.cancelScroll = function() { |
|  | if (self.timer) clearAnimationFrame(self.timer); |
|  | self.timer = 0; |
|  | self.bzscroll = false; |
|  | self.scrollrunning = false; |
|  | return self; |
|  | }; |
|  |  |
|  | } |
|  |  |
|  | this.doScrollBy = function(stp, relative) { |
|  | var ny = 0; |
|  | if (relative) { |
|  | ny = Math.floor((self.scroll.y - stp) \* self.scrollratio.y) |
|  | } else { |
|  | var sy = (self.timer) ? self.newscrolly : self.getScrollTop(true); |
|  | ny = sy - stp; |
|  | } |
|  | if (self.bouncescroll) { |
|  | var haf = Math.round(self.view.h / 2); |
|  | if (ny < -haf) ny = -haf |
|  | else if (ny > (self.page.maxh + haf)) ny = (self.page.maxh + haf); |
|  | } |
|  | self.cursorfreezed = false; |
|  |  |
|  | var py = self.getScrollTop(true); |
|  | if (ny < 0 && py <= 0) return self.noticeCursor(); |
|  | else if (ny > self.page.maxh && py >= self.page.maxh) { |
|  | self.checkContentSize(); |
|  | return self.noticeCursor(); |
|  | } |
|  |  |
|  | self.doScrollTop(ny); |
|  | }; |
|  |  |
|  | this.doScrollLeftBy = function(stp, relative) { |
|  | var nx = 0; |
|  | if (relative) { |
|  | nx = Math.floor((self.scroll.x - stp) \* self.scrollratio.x) |
|  | } else { |
|  | var sx = (self.timer) ? self.newscrollx : self.getScrollLeft(true); |
|  | nx = sx - stp; |
|  | } |
|  | if (self.bouncescroll) { |
|  | var haf = Math.round(self.view.w / 2); |
|  | if (nx < -haf) nx = -haf; |
|  | else if (nx > (self.page.maxw + haf)) nx = (self.page.maxw + haf); |
|  | } |
|  | self.cursorfreezed = false; |
|  |  |
|  | var px = self.getScrollLeft(true); |
|  | if (nx < 0 && px <= 0) return self.noticeCursor(); |
|  | else if (nx > self.page.maxw && px >= self.page.maxw) return self.noticeCursor(); |
|  |  |
|  | self.doScrollLeft(nx); |
|  | }; |
|  |  |
|  | this.doScrollTo = function(pos, relative) { |
|  | var ny = (relative) ? Math.round(pos \* self.scrollratio.y) : pos; |
|  | if (ny < 0) ny = 0; |
|  | else if (ny > self.page.maxh) ny = self.page.maxh; |
|  | self.cursorfreezed = false; |
|  | self.doScrollTop(pos); |
|  | }; |
|  |  |
|  | this.checkContentSize = function() { |
|  | var pg = self.getContentSize(); |
|  | if ((pg.h != self.page.h) \|\| (pg.w != self.page.w)) self.resize(false, pg); |
|  | }; |
|  |  |
|  | self.onscroll = function(e) { |
|  | if (self.rail.drag) return; |
|  | if (!self.cursorfreezed) { |
|  | self.synched('scroll', function() { |
|  | self.scroll.y = Math.round(self.getScrollTop() \* (1 / self.scrollratio.y)); |
|  | if (self.railh) self.scroll.x = Math.round(self.getScrollLeft() \* (1 / self.scrollratio.x)); |
|  | self.noticeCursor(); |
|  | }); |
|  | } |
|  | }; |
|  | self.bind(self.docscroll, "scroll", self.onscroll); |
|  |  |
|  | this.doZoomIn = function(e) { |
|  | if (self.zoomactive) return; |
|  | self.zoomactive = true; |
|  |  |
|  | self.zoomrestore = { |
|  | style: {} |
|  | }; |
|  | var lst = ['position', 'top', 'left', 'zIndex', 'backgroundColor', 'marginTop', 'marginBottom', 'marginLeft', 'marginRight']; |
|  | var win = self.win[0].style; |
|  | for (var a in lst) { |
|  | var pp = lst[a]; |
|  | self.zoomrestore.style[pp] = (typeof win[pp] != "undefined") ? win[pp] : ''; |
|  | } |
|  |  |
|  | self.zoomrestore.style.width = self.win.css('width'); |
|  | self.zoomrestore.style.height = self.win.css('height'); |
|  |  |
|  | self.zoomrestore.padding = { |
|  | w: self.win.outerWidth() - self.win.width(), |
|  | h: self.win.outerHeight() - self.win.height() |
|  | }; |
|  |  |
|  | if (cap.isios4) { |
|  | self.zoomrestore.scrollTop = $(window).scrollTop(); |
|  | $(window).scrollTop(0); |
|  | } |
|  |  |
|  | self.win.css({ |
|  | "position": (cap.isios4) ? "absolute" : "fixed", |
|  | "top": 0, |
|  | "left": 0, |
|  | "z-index": globalmaxzindex + 100, |
|  | "margin": "0px" |
|  | }); |
|  | var bkg = self.win.css("backgroundColor"); |
|  | if (bkg == "" \|\| /transparent\|rgba\(0, 0, 0, 0\)\|rgba\(0,0,0,0\)/.test(bkg)) self.win.css("backgroundColor", "#fff"); |
|  | self.rail.css({ |
|  | "z-index": globalmaxzindex + 101 |
|  | }); |
|  | self.zoom.css({ |
|  | "z-index": globalmaxzindex + 102 |
|  | }); |
|  | self.zoom.css('backgroundPosition', '0px -18px'); |
|  | self.resizeZoom(); |
|  |  |
|  | if (self.onzoomin) self.onzoomin.call(self); |
|  |  |
|  | return self.cancelEvent(e); |
|  | }; |
|  |  |
|  | this.doZoomOut = function(e) { |
|  | if (!self.zoomactive) return; |
|  | self.zoomactive = false; |
|  |  |
|  | self.win.css("margin", ""); |
|  | self.win.css(self.zoomrestore.style); |
|  |  |
|  | if (cap.isios4) { |
|  | $(window).scrollTop(self.zoomrestore.scrollTop); |
|  | } |
|  |  |
|  | self.rail.css({ |
|  | "z-index": self.zindex |
|  | }); |
|  | self.zoom.css({ |
|  | "z-index": self.zindex |
|  | }); |
|  | self.zoomrestore = false; |
|  | self.zoom.css('backgroundPosition', '0px 0px'); |
|  | self.onResize(); |
|  |  |
|  | if (self.onzoomout) self.onzoomout.call(self); |
|  |  |
|  | return self.cancelEvent(e); |
|  | }; |
|  |  |
|  | this.doZoom = function(e) { |
|  | return (self.zoomactive) ? self.doZoomOut(e) : self.doZoomIn(e); |
|  | }; |
|  |  |
|  | this.resizeZoom = function() { |
|  | if (!self.zoomactive) return; |
|  |  |
|  | var py = self.getScrollTop(); //preserve scrolling position |
|  | self.win.css({ |
|  | width: $(window).width() - self.zoomrestore.padding.w + "px", |
|  | height: $(window).height() - self.zoomrestore.padding.h + "px" |
|  | }); |
|  | self.onResize(); |
|  |  |
|  | self.setScrollTop(Math.min(self.page.maxh, py)); |
|  | }; |
|  |  |
|  | this.init(); |
|  |  |
|  | $.nicescroll.push(this); |
|  |  |
|  | }; |
|  |  |
|  | // Inspired by the work of Kin Blas |
|  | // http://webpro.host.adobe.com/people/jblas/momentum/includes/jquery.momentum.0.7.js |
|  |  |
|  |  |
|  | var ScrollMomentumClass2D = function(nc) { |
|  | var self = this; |
|  | this.nc = nc; |
|  |  |
|  | this.lastx = 0; |
|  | this.lasty = 0; |
|  | this.speedx = 0; |
|  | this.speedy = 0; |
|  | this.lasttime = 0; |
|  | this.steptime = 0; |
|  | this.snapx = false; |
|  | this.snapy = false; |
|  | this.demulx = 0; |
|  | this.demuly = 0; |
|  |  |
|  | this.lastscrollx = -1; |
|  | this.lastscrolly = -1; |
|  |  |
|  | this.chkx = 0; |
|  | this.chky = 0; |
|  |  |
|  | this.timer = 0; |
|  |  |
|  | this.time = function() { |
|  | return +new Date(); //beautifull hack |
|  | }; |
|  |  |
|  | this.reset = function(px, py) { |
|  | self.stop(); |
|  | var now = self.time(); |
|  | self.steptime = 0; |
|  | self.lasttime = now; |
|  | self.speedx = 0; |
|  | self.speedy = 0; |
|  | self.lastx = px; |
|  | self.lasty = py; |
|  | self.lastscrollx = -1; |
|  | self.lastscrolly = -1; |
|  | }; |
|  |  |
|  | this.update = function(px, py) { |
|  | var now = self.time(); |
|  | self.steptime = now - self.lasttime; |
|  | self.lasttime = now; |
|  | var dy = py - self.lasty; |
|  | var dx = px - self.lastx; |
|  | var sy = self.nc.getScrollTop(); |
|  | var sx = self.nc.getScrollLeft(); |
|  | var newy = sy + dy; |
|  | var newx = sx + dx; |
|  | self.snapx = (newx < 0) \|\| (newx > self.nc.page.maxw); |
|  | self.snapy = (newy < 0) \|\| (newy > self.nc.page.maxh); |
|  | self.speedx = dx; |
|  | self.speedy = dy; |
|  | self.lastx = px; |
|  | self.lasty = py; |
|  | }; |
|  |  |
|  | this.stop = function() { |
|  | self.nc.unsynched("domomentum2d"); |
|  | if (self.timer) clearTimeout(self.timer); |
|  | self.timer = 0; |
|  | self.lastscrollx = -1; |
|  | self.lastscrolly = -1; |
|  | }; |
|  |  |
|  | this.doSnapy = function(nx, ny) { |
|  | var snap = false; |
|  |  |
|  | if (ny < 0) { |
|  | ny = 0; |
|  | snap = true; |
|  | } else if (ny > self.nc.page.maxh) { |
|  | ny = self.nc.page.maxh; |
|  | snap = true; |
|  | } |
|  |  |
|  | if (nx < 0) { |
|  | nx = 0; |
|  | snap = true; |
|  | } else if (nx > self.nc.page.maxw) { |
|  | nx = self.nc.page.maxw; |
|  | snap = true; |
|  | } |
|  |  |
|  | (snap) ? self.nc.doScrollPos(nx, ny, self.nc.opt.snapbackspeed): self.nc.triggerScrollEnd(); |
|  | }; |
|  |  |
|  | this.doMomentum = function(gp) { |
|  | var t = self.time(); |
|  | var l = (gp) ? t + gp : self.lasttime; |
|  |  |
|  | var sl = self.nc.getScrollLeft(); |
|  | var st = self.nc.getScrollTop(); |
|  |  |
|  | var pageh = self.nc.page.maxh; |
|  | var pagew = self.nc.page.maxw; |
|  |  |
|  | self.speedx = (pagew > 0) ? Math.min(60, self.speedx) : 0; |
|  | self.speedy = (pageh > 0) ? Math.min(60, self.speedy) : 0; |
|  |  |
|  | var chk = l && (t - l) <= 60; |
|  |  |
|  | if ((st < 0) \|\| (st > pageh) \|\| (sl < 0) \|\| (sl > pagew)) chk = false; |
|  |  |
|  | var sy = (self.speedy && chk) ? self.speedy : false; |
|  | var sx = (self.speedx && chk) ? self.speedx : false; |
|  |  |
|  | if (sy \|\| sx) { |
|  | var tm = Math.max(16, self.steptime); //timeout granularity |
|  |  |
|  | if (tm > 50) { // do smooth |
|  | var xm = tm / 50; |
|  | self.speedx \*= xm; |
|  | self.speedy \*= xm; |
|  | tm = 50; |
|  | } |
|  |  |
|  | self.demulxy = 0; |
|  |  |
|  | self.lastscrollx = self.nc.getScrollLeft(); |
|  | self.chkx = self.lastscrollx; |
|  | self.lastscrolly = self.nc.getScrollTop(); |
|  | self.chky = self.lastscrolly; |
|  |  |
|  | var nx = self.lastscrollx; |
|  | var ny = self.lastscrolly; |
|  |  |
|  | var onscroll = function() { |
|  | var df = ((self.time() - t) > 600) ? 0.04 : 0.02; |
|  |  |
|  | if (self.speedx) { |
|  | nx = Math.floor(self.lastscrollx - (self.speedx \* (1 - self.demulxy))); |
|  | self.lastscrollx = nx; |
|  | if ((nx < 0) \|\| (nx > pagew)) df = 0.10; |
|  | } |
|  |  |
|  | if (self.speedy) { |
|  | ny = Math.floor(self.lastscrolly - (self.speedy \* (1 - self.demulxy))); |
|  | self.lastscrolly = ny; |
|  | if ((ny < 0) \|\| (ny > pageh)) df = 0.10; |
|  | } |
|  |  |
|  | self.demulxy = Math.min(1, self.demulxy + df); |
|  |  |
|  | self.nc.synched("domomentum2d", function() { |
|  |  |
|  | if (self.speedx) { |
|  | var scx = self.nc.getScrollLeft(); |
|  | if (scx != self.chkx) self.stop(); |
|  | self.chkx = nx; |
|  | self.nc.setScrollLeft(nx); |
|  | } |
|  |  |
|  | if (self.speedy) { |
|  | var scy = self.nc.getScrollTop(); |
|  | if (scy != self.chky) self.stop(); |
|  | self.chky = ny; |
|  | self.nc.setScrollTop(ny); |
|  | } |
|  |  |
|  | if (!self.timer) { |
|  | self.nc.hideCursor(); |
|  | self.doSnapy(nx, ny); |
|  | } |
|  |  |
|  | }); |
|  |  |
|  | if (self.demulxy < 1) { |
|  | self.timer = setTimeout(onscroll, tm); |
|  | } else { |
|  | self.stop(); |
|  | self.nc.hideCursor(); |
|  | self.doSnapy(nx, ny); |
|  | } |
|  | }; |
|  |  |
|  | onscroll(); |
|  |  |
|  | } else { |
|  | self.doSnapy(self.nc.getScrollLeft(), self.nc.getScrollTop()); |
|  | } |
|  |  |
|  | } |
|  |  |
|  | }; |
|  |  |
|  |  |
|  | // override jQuery scrollTop |
|  |  |
|  | var \_scrollTop = jQuery.fn.scrollTop; // preserve original function |
|  |  |
|  | jQuery.cssHooks["pageYOffset"] = { |
|  | get: function(elem, computed, extra) { |
|  | var nice = $.data(elem, '\_\_nicescroll') \|\| false; |
|  | return (nice && nice.ishwscroll) ? nice.getScrollTop() : \_scrollTop.call(elem); |
|  | }, |
|  | set: function(elem, value) { |
|  | var nice = $.data(elem, '\_\_nicescroll') \|\| false; |
|  | (nice && nice.ishwscroll) ? nice.setScrollTop(parseInt(value)): \_scrollTop.call(elem, value); |
|  | return this; |
|  | } |
|  | }; |
|  |  |
|  | /\* |
|  | $.fx.step["scrollTop"] = function(fx){ |
|  | $.cssHooks["scrollTop"].set( fx.elem, fx.now + fx.unit ); |
|  | }; |
|  | \*/ |
|  |  |
|  | jQuery.fn.scrollTop = function(value) { |
|  | if (typeof value == "undefined") { |
|  | var nice = (this[0]) ? $.data(this[0], '\_\_nicescroll') \|\| false : false; |
|  | return (nice && nice.ishwscroll) ? nice.getScrollTop() : \_scrollTop.call(this); |
|  | } else { |
|  | return this.each(function() { |
|  | var nice = $.data(this, '\_\_nicescroll') \|\| false; |
|  | (nice && nice.ishwscroll) ? nice.setScrollTop(parseInt(value)): \_scrollTop.call($(this), value); |
|  | }); |
|  | } |
|  | }; |
|  |  |
|  | // override jQuery scrollLeft |
|  |  |
|  | var \_scrollLeft = jQuery.fn.scrollLeft; // preserve original function |
|  |  |
|  | $.cssHooks.pageXOffset = { |
|  | get: function(elem, computed, extra) { |
|  | var nice = $.data(elem, '\_\_nicescroll') \|\| false; |
|  | return (nice && nice.ishwscroll) ? nice.getScrollLeft() : \_scrollLeft.call(elem); |
|  | }, |
|  | set: function(elem, value) { |
|  | var nice = $.data(elem, '\_\_nicescroll') \|\| false; |
|  | (nice && nice.ishwscroll) ? nice.setScrollLeft(parseInt(value)): \_scrollLeft.call(elem, value); |
|  | return this; |
|  | } |
|  | }; |
|  |  |
|  | /\* |
|  | $.fx.step["scrollLeft"] = function(fx){ |
|  | $.cssHooks["scrollLeft"].set( fx.elem, fx.now + fx.unit ); |
|  | }; |
|  | \*/ |
|  |  |
|  | jQuery.fn.scrollLeft = function(value) { |
|  | if (typeof value == "undefined") { |
|  | var nice = (this[0]) ? $.data(this[0], '\_\_nicescroll') \|\| false : false; |
|  | return (nice && nice.ishwscroll) ? nice.getScrollLeft() : \_scrollLeft.call(this); |
|  | } else { |
|  | return this.each(function() { |
|  | var nice = $.data(this, '\_\_nicescroll') \|\| false; |
|  | (nice && nice.ishwscroll) ? nice.setScrollLeft(parseInt(value)): \_scrollLeft.call($(this), value); |
|  | }); |
|  | } |
|  | }; |
|  |  |
|  | var NiceScrollArray = function(doms) { |
|  | var self = this; |
|  | this.length = 0; |
|  | this.name = "nicescrollarray"; |
|  |  |
|  | this.each = function(fn) { |
|  | for (var a = 0, i = 0; a < self.length; a++) fn.call(self[a], i++); |
|  | return self; |
|  | }; |
|  |  |
|  | this.push = function(nice) { |
|  | self[self.length] = nice; |
|  | self.length++; |
|  | }; |
|  |  |
|  | this.eq = function(idx) { |
|  | return self[idx]; |
|  | }; |
|  |  |
|  | if (doms) { |
|  | for (var a = 0; a < doms.length; a++) { |
|  | var nice = $.data(doms[a], '\_\_nicescroll') \|\| false; |
|  | if (nice) { |
|  | this[this.length] = nice; |
|  | this.length++; |
|  | } |
|  | }; |
|  | } |
|  |  |
|  | return this; |
|  | }; |
|  |  |
|  | function mplex(el, lst, fn) { |
|  | for (var a = 0; a < lst.length; a++) fn(el, lst[a]); |
|  | }; |
|  | mplex( |
|  | NiceScrollArray.prototype, ['show', 'hide', 'toggle', 'onResize', 'resize', 'remove', 'stop', 'doScrollPos'], |
|  | function(e, n) { |
|  | e[n] = function() { |
|  | var args = arguments; |
|  | return this.each(function() { |
|  | this[n].apply(this, args); |
|  | }); |
|  | }; |
|  | } |
|  | ); |
|  |  |
|  | jQuery.fn.getNiceScroll = function(index) { |
|  | if (typeof index == "undefined") { |
|  | return new NiceScrollArray(this); |
|  | } else { |
|  | var nice = this[index] && $.data(this[index], '\_\_nicescroll') \|\| false; |
|  | return nice; |
|  | } |
|  | }; |
|  |  |
|  | jQuery.extend(jQuery.expr[':'], { |
|  | nicescroll: function(a) { |
|  | return ($.data(a, '\_\_nicescroll')) ? true : false; |
|  | } |
|  | }); |
|  |  |
|  | $.fn.niceScroll = function(wrapper, opt) { |
|  | if (typeof opt == "undefined") { |
|  | if ((typeof wrapper == "object") && !("jquery" in wrapper)) { |
|  | opt = wrapper; |
|  | wrapper = false; |
|  | } |
|  | } |
|  | opt = $.extend({},opt); // cloning |
|  | var ret = new NiceScrollArray(); |
|  | if (typeof opt == "undefined") opt = {}; |
|  |  |
|  | if (wrapper \|\| false) { |
|  | opt.doc = $(wrapper); |
|  | opt.win = $(this); |
|  | } |
|  | var docundef = !("doc" in opt); |
|  | if (!docundef && !("win" in opt)) opt.win = $(this); |
|  |  |
|  | this.each(function() { |
|  | var nice = $(this).data('\_\_nicescroll') \|\| false; |
|  | if (!nice) { |
|  | opt.doc = (docundef) ? $(this) : opt.doc; |
|  | nice = new NiceScrollClass(opt, $(this)); |
|  | $(this).data('\_\_nicescroll', nice); |
|  | } |
|  | ret.push(nice); |
|  | }); |
|  | return (ret.length == 1) ? ret[0] : ret; |
|  | }; |
|  |  |
|  | window.NiceScroll = { |
|  | getjQuery: function() { |
|  | return jQuery |
|  | } |
|  | }; |
|  |  |
|  | if (!$.nicescroll) { |
|  | $.nicescroll = new NiceScrollArray(); |
|  | $.nicescroll.options = \_globaloptions; |
|  | } |
|  |  |
|  | })); |

Jump to Line

Go

- Status
- API
- Training
- Shop
- Blog
- About
- Pricing


- © 2015 GitHub, Inc.
- Terms
- Privacy
- Security
- Contact
- Help

Something went wrong with that request. Please try again.

You signed in with another tab or window. Reload to refresh your session.
You signed out in another tab or window. Reload to refresh your session.
