# Supplementary material for: Isolation, identification, and whole-genome sequencing of high-yield protease bacteria from Daqu of ZhangGong Laojiu
Source: PLoS One. 2022 Apr 26;17(4):e0264677. doi: 10.1371/journal.pone.0264677 (PMC9041807; doi:10.1371/journal.pone.0264677)
Supplement: Attachment — Submitted filename: identification and whole genome sequencing of bacteria.docx [file pone.0264677.s004.docx]

**Isolation, identification and whole genome sequencing of high-yield protease bacteria from daqu of Zhanggong Laojiu**

**Yanbo Liu ^1,2,3,4^·Junying Fu^1,3,4^ ·Linlin Wang^1,3,4^·Zhijun Zhao ^1,3,4^·**

**Huihui Wang^1,3,4^·Suna Han^2^·Xiyu Sun ^1,3,5^·Chunmei Pan ^1,3,4🖂^**

1 College of Food and Biological Engineering(Liquor College), Henan University of Animal Husbandry and Economy,Zhengzhou ,Henan 450046, China,2 Postdoctoral Programme, Henan Yangshao Distillery Co., Ltd., Henan 472400, China,3 Henan Liquor Style Engineering Technology Research Center, Henan University of Animal Husbandry and Economy, Zhengzhou ,Henan 450046, China,4 Zhengzhou Key Laboratory of Liquor Brewing Microbial Technology, Henan University of Animal Husbandry and Economy, Zhengzhou ,Henan 450046, China ,5 ZhangGongLaoJiu Wine Co. Ltd., Ningling , Henan476733, China

Corresponding author **🖂** sige518888@163.com

**Abstract:** A total of 296 strains of protease-producing bacteria were isolated and purified from the yeast for making hard liquor of Zhanggong Laojiu. After calculating the ratio of transparent ring diameter to colony diameter, and measuring protease activities, a strain of high-yield protease bacteria DW-7 was screened out, with the protease activity of 99.54 U/ mL. Through morphology observation, 16S rDNA sequence analysis and physiological and biochemical tests, DW-7 was determined to be *Bacillus velezensis*. Meanwhile, whole genome sequencing (WGS) using PacbBio and Illumina platform was performed, and gene annotation was conducted using the COG, KEGG, NR and GO databases. The results showed that the genome of DW-7 was 3,942,829 bp long, and among them, GC accounted for 46.45%. A total of 3,662 protein-encoding genes were predicted, with the total length of 3,402,822 bp. 2,283, 2,796, and 2,127 genes were annotated in the COG, KEGG, and GO databases, respectively. A total of 196 high-yield protease genes were mainly enriched in the metabolism of alanine, aspartic acid, and glutamate, metabolism of glycine, serine, and threonine, ABC transporter and transporter pathways.

**Key words:** The yeast for making hard liquor; Protease-producing bacteria; Isolation and identification; Whole genome sequencing; Gene function annotation

**Introduction**

The Chinese Luzhou-flavor liquor process is a typical oriental solid-state fermentation. Liquor fermentation is a complex process in an open environment, which is coordinated by various microorganisms and enzymes. The microorganisms in liquor mainly come from the yeast for making hard liquor, the surrounding environment, pit mud and yellow water ^[1]^. The yeast for making hard liquor is a unique saccharification starter for solid liquor brewing in China. It not only contains important crude enzymes and complex microflora, but also the source of important biologically active substances like aroma precursors that determine the flavor characteristics ^[2-3]^. The quality of the yeast for making hard liquor directly affects the quality, yield and style of liquor.

The microorganisms in the yeast for making hard liquor mainly include bacteria, yeasts, molds and a small amount of actinomycetes. Bacteria is a vital type of microorganisms in the yeast for making hard liquor, the amount and quantity of which directly determine its quality. Bacillus sp. is the dominant flora in the yeast for making hard liquor, which produces various enzymes that is closely linked with the formation of flavor substances during the fermentation process ^[4]^. It is reported that many flavor substances in liquor are derived from proteins, and the functions of proteins and their enzymes are of significance to the flavor of liquor. Protease is an enzyme that hydrolyzes peptide bonds into amino acids or short peptides ^[5]^, which is characterized by maintaining enzyme activities under acidic, neutral and alkaline conditions. It is a metabolite of various microorganisms in the production of the yeast for making hard liquor ^[6]^. In the process of solid-state liquor fermentation, proteases in the yeast for making hard liquor are able to promote the dissolution of raw materials, decompose proteins, produce amino acids, provide nitrogen sources, aroma precursors and flavor components, promote the growth of microorganisms, degrade yeast cells, improve the utilization of raw materials, provide , and cooperate in the co-fermentation with other microbial, inhibit massive production of fusel oil, enhance the yield and quality of the original liquor, and the aroma ^[7-8]^.

WGS is a fast, low-cost, and high-efficiency third-generation sequencing technology that is able to provide a complete genome sequence of bacteria. The differences between species of the same genus can be better identified using WGS and gene annotations using online databases like GO, KEGG, COG, NR, etc. WGS has become an efficient detection method that is widely applied to identify microbial communities in intestinal flora, soil and fungi ^[9-10]^. It is of great significance in mining the core genome, identifying specific genes, and analyzing functional genomics, which contributes to explore the diversity and biological characteristics of unknown microbial groups ^[11]^.

At present, domestic and foreign research mainly focuses on the physical and chemical properties and purification technologies of proteases, as well as the methods to obtain high-yield protease strains, and protease products through strain mutagenesis, genetic engineering, etc. For example, Lu et al.(2012)^[12]^ screened out a high-yield neutral protease bacterium from the high-temperature yeast for making hard liquor, which is identified as *Bacillus amyloliquefaciens*. Nie et al.(2015)^[13]^ initially screened by calculating the transparent ring diameter and re-screened by measuring protease activity, and finally yielded 2 strains of high-yield proteases from the yeast for making hard liquor of Jiang-flavor Chinese spirits, which are identified as *Bacillus subtilis*. However, the exact mechanism underlying the screening of high-yield proteases from the yeast for making hard liquor of the Chinese Luzhou-flavor liquor has been rarely reported. In the present study, we screened out a strain of high-yield protease bacteria from the yeast for making hard liquor of Zhanggong Laojiu. After WGS using PacbBio and Illumina platform, the whole genome of the strain was obtained. Following gene annotations using online databases of GO, KEGG, COG, NR and Swiss-Prot, we further revealed the key enriched genes and metabolic pathways. Our findings provide theoretical references for mining the high-yield proteases, and its biological functions.

**Materials and Methods**

**Materials and reagents**

The medium-temperature starter was provided by the Henan Zhanggong Laojiu Wine Co., Ltd. Sodium carbonate anhydrous was from Tianjin Dingshengxin Chemical Co., Ltd; L-tyrosine, agar powder, casein, trichloroacetic acid and sodium chloride were from Tianjin Kemiou Chemical Reagent Co., Ltd; Beef extract peptone was from Beijing Aoboxing Biotechnology Co., Ltd; Folin-Ciocalteu reagent was from Solarbio, Biotechnology Co., Ltd; Ezup Column Bacteria Genomic DNA Purification Kit was from Sangon Biotech, Co, Ltd; DNA 1000 assay kit was from Lithuania Agilent Technologies; ABI StepOnePlus Real-Time PCR System was from Life Technologies.

**Instruments**

Constant-temperature incubator was from Shanghai Shcimo Medical Device Manufacturing Co., Ltd; High speed refrigerated centrifuge was from Shanghai Anting Device Manufacturing Co., Ltd; Dk-8D digital thermostatic laboratory water bath was from Guangzhou Hezhong Biotechnology Co., Ltd; Three-dimensional autoclave was from Shanghai Shenan Medical Device Manufacturing Co., Ltd; 752 UV-VIS spectrophotometer was from Shanghai Jinghua Instruments; Biological purification table was from Suzhou Purification Equipment Co., Ltd; PacBio Single Molecule, Real-Time (SMRT) Sequencing was from Pacific Biosciences of California; Novaseq 6000 was from Illumina; 2100 Bioanalyzer Instrument was from Agilent Technologies; Eppendorf 5427R centrifuge was from Eppendorf; NanoDrop 2000 spectrophotometer from Thermal Fisher Scientific; DYY-6C agarose gel electrophoresis was from Beijing Liuyi Biotechnology Co., Ltd; PCR instrument was from Dongsheng Xingye Scientific Instrument Co., Ltd.

**Culture medium**

Casein medium ^[14]^: 3 g beef extract, 5 g sodium chloride, 10 g peptone, 20 g agar powder and 4 g casein in the constant volume 1000 mL of distilled water at pH 7.0-7.2, and sterilized at 121℃ for 20 min.

Beef extract peptone agar medium: 3 g beef extract, 5 g sodium chloride, 10 g peptone, 20 g agar powder and 4 g casein in the constant volume 1000 mL of distilled water at pH 7.0-7.2, and sterilized at 121℃ for 20 min.

Fluid medium ^[15]^: 3 g beef extract, 5 g sodium chloride and 10 g peptone in the constant volume 1000 mL of distilled water at pH 7.0-7.2, and sterilized at 121℃ for 20 min.

Solid medium ^[16]^: 20 g bran and 20 mL of distilled water, and sterilized at 121℃ for 20 min.

**Experimental methods**

**Isolation of protease-producing bacteria**

Isolation: The yeast for making hard liquor was ground into powder and 10 g powder was added into an Erlenmeyer flask containing 90 mL of sterile distilled water. After gently shaking in a shaking incubator at 150 r/min for 30 min ^[17]^, 1 mL of the suspension was transferred to a tube containing 9 mL of sterile water, and diluted successively in 10 times. 0.1 mL of 10^-3^, 10^-4^, 10^-5^, and 10^-6^-times diluted bacteria solution was plated, and inverted cultured at 37℃ for 24 h ^[18]^.

Initial screening:Under aseptic conditions, a sterile pipette tip was used to dip a small amount of purified bacteria on the casein medium plate, created three parallels, and invert cultured for 24 h to observe the formation of transparent ring. The ratio (D/d) of transparent ring diameter (D) to colony diameter (d) was calculated, and those with a larger D/d were selected for re-screening ^[19]^.

**Screening of protease-producing bacteria**

Preparation of standard curve

The standard curve was plotted using the absorbance value and the concentration of L-tyrosine as the ordinate and abscissa, respectively.

Preparation of crude enzyme solution

the bacteria were inoculated to the solid medium of beef extract peptone, inverted cultured at 37°C for 24 h, and then a single colony with obvious morphology was picked into the liquid medium, and cultured on a shaker at 37°C for 24 h. The bacterial suspension was inoculated to the solid culture medium with 10% of the inoculation amount, and incubated at 37°C for 3 d. 10 g of solid fermentation product was added into a 250 mL Erlenmeyer flask containing 90 mL of distilled water, and incubated in a 40°C water bath for 1 h. Stir it every 15 min. After centrifugation, a crude enzyme solution was obtained ^[20]^.

Measurement of protease activity using the Folin-Ciocalteu method

Calculation of protease activity

Under the conditions of 1 mL of protease solution, pH 7.5 and 40℃ temperature, the amount of protease required to hydrolyze casein to produce 1 μg L-tyrosine per minute was defined as the protease activity.

Protease activity (U/mL) = $\frac{\text{A × 4 × N × OD}}{\text{10}}$ ;

Where A was a constant obtained from the standard curve, which was equal to the number of micrograms of L-tyrosine equivalent to OD_680_ = 1; 4 indicated 1 mL of solution collected from the total volume of 4 mL for measurement; N was the dilution multiple of protease solution to be measured; 10 indicated the reaction time of 10 min and OD was the mean optical density of the parallel sample.

**Identification of protease-producing bacteria**

Morphology identification

The high-yield protease-producing bacteria were activated on the plate, inoculated on the casein medium by the spot inoculation method, and subjected to Gram staining. The morphology was observed under a microscope ^[21]^.

Molecular biology identification

Using the DNA extracted from the high-yield protease-producing bacteria by the DNA extraction kit as a template, the 16S rDNA gene in the bacterial genomic DNA was amplified using the bacterial universal primers 27F (5'-AGA GTT TCA TCT GGC TCA G-3') and 1492R (5'-GGT TAC CTT GTT ACG ACT T-3'). PCR was performed at 94°C pre-denaturation for 5 min, followed by 35 cycles at 94°C denaturation for 0.5 min, 55°C annealing for 0.75 min, and 72°C extension for 1.5 min. At last, repair and extension at 72°C was performed for 10 min, and the sample was stored at 4°C. A 50 μL PCR amplification system was prepared, and the amplification results were detected in 1% agarose electrophoresis to observe the specific products and their relative molecular masses. The amplified products were finally sent to Shanghai Sangon Biotech Co., Ltd. for sequencing ^[22]^. Spliced sequences were compared with data in the NCBI database, and the standard bacteria with similar genetic relationships were analyzed using MEGA 6.0 software to construct a phylogenetic tree, thus visually observing the genera and species of the bacteria ^[23]^.

Physiological and biochemical tests

Physiological and biochemical tests of the high-yield protease-producing bacteria were performed based on *the 8^th^ Edition Bergey’s Mannual of Determinative Bacteriology* and *the Common Identification Methods of General Bacteria* ^[24]^.

**WGS analysis**

DNA extraction

Genomic DNAs were extracted according to the instructions of the bacterial DNA extraction kit. To ensure the quality of WGS, and the purity and concentration of DNAs, they were sent to Guangzhou Gene Denovo Biotechnology Co., Ltd. for sequencing.

PacbBio sequencing

g-TUBE for genomic DNA shearing was performed to process genomic DNA into 8-10k fragments, followed by DNA end repair. The obtained SMRT Bell DNA template was sent to quality detection, and Agilent 2100 was used to evaluate the insert size. Finally, sequencing was performed using the PacBio platform.

Illumina sequencing

Genomic DNA fragments were prepared using dsDNA fragmentase, followed by end repair, phosphorylation, adding polyA tails and sequencing adapters. DNA fragments were purified by the AMPure XP magnetic beads, and target fragments with 300-400 bp were subjected to PCR to establish the library. PCR products were further purified by the AMPure XP magnetic beads, and examined using Agilent 2100. Qualified products were sequenced using the Illumina Noveseq 6000 platform.

Bioinformatic analyses

Low-quality data in the raw data (reads) of PacbBio and Illumina sequencing should be filtered to ensure the accuracy and reliability of bioinformatic analyses. Clean data were assembled for analyzing genomics containing target strains, including genomic components of strains, tandem repeat prediction, tRNA prediction, rRNA prediction, sRNA prediction, genomic island prediction, transposons prediction and prephage prediction performed using the TRF (version 4.09) ^[25]^, tRNAscan (version 1.3.1) ^[26]^, rRNAmmer (version 1.2) ^[27]^, cmscan (version 1.1.2) ^[28]^, IslandPath-DIMOB (version 1.0.0) ^[29]^, TransposonPSI (version: 20100822) ^[30]^ and PHAST (version 2.0) ^[31]^, respectively. Gene function annotation was performed using the following online databases: COG database (<https://www.ncbi.nlm.nih.gov/COG/>), KEGG database (<https://www.genome.jp/kegg/>), NR database (<ftp://ftp.ncbi.nih.gov:21/blast/db/FASTA/>), SwissProt database database (<https://www.uniprot.org/>), GO database (<http://geneontology.org/>).

**Results**

**Isolation of protease-producing bacteria**

Isolation: Through gradient dilution and plate coating, a total of 296 strains of protease-producing bacteria were screened out from the yeast for making hard liquor of Zhanggong Laojiu.

Initial screening: A total of 296 strains of protease-producing bacteria were inoculated on the casein medium, and inverted cultured at 37°C for 24 h. The calculated D/d was shown in Table 1.

**Table 1 diameter of transparent ring and strain diameter ratio**

| No. | d/cm | D/cm | D/d |
| --- | --- | --- | --- |
| DW-2 | 0.60±0.06 | 2.30±0.10 | 3.84±0.25 |
| DW-7 | 0.57±0.15 | 2.50±0.06 | 4.39±0.98 |
| DW-8 | 0.50±0.06 | 1.90±0.06 | 3.80±0.34 |
| DW-22 | 0.60±0.06 | 2.20±0.12 | 3.69±0.62 |
| DW-32 | 0.60±0.06 | 2.40±0.10 | 4.00±0.25 |
| DW-40 | 0.50±0.00 | 1.80±0.06 | 3.60±0.12 |

D/d of DW-7 was 4.39, which was the highest among isolated strains, suggesting the strong protease activity and degradation ability to casein. To obtain high-yield protease-producing bacteria, we measured the protease activity in the following 6 strains.

**Screening of high-yield protease-producing bacteria**

Standard curving plotting

As shown in Figure 1, the regression equation of the L-tyrosine standard curve was y = 0.0102x - 0.0057, and the correlation coefficient (R) was 0.9977, indicating a good linear relationship.

| L-tyrosine standard curve（μg/mL） | Light absorption value |
| --- | --- |
| 0 | 0 |
| 10 | 0.098 |
| 20 | 0.197 |
| 30 | 0.283 |
| 40 | 0.403 |
| 50 | 0.513 |

**Fig.1 L-tyrosine standard curve**

Measurement of protease activity using the Folin-Ciocalteu method

As shown in Table 2, the protease activity of DW-7 remained the highest among the 6 tested strains at 99.54 U/mL. We subsequently examined the morphology, molecular biology and physiological and biochemical properties of it.

**Table 2 Determination of protease activity**

| No. | OD-value | protease activity（U/mL） |
| --- | --- | --- |
| DW-2 | 0.555±0.01 | 87.56±1.79 |
| DW-7 | 0.631±0.01 | 99.54±2.23 |
| DW-8 | 0.540±0.00 | 85.19±0.11 |
| DW-22 | 0.525±0.00 | 82.82±0.00 |
| DW-32 | 0.574±0.01 | 90.55±1.90 |
| DW-40 | 0.497±0.03 | 78.41±4.24 |

**Identification of high-yield protease-producing bacteria**

Morphology identification

Colonies were milky white, with a moist and viscous surface, and wrinkles. They were round-shaped and had smooth edges without flagella (Figure 2a). Gram staining was further performed to observe cell morphology under a microscope. The bacteria were stained red, suggesting the Gram-negative bacteria. Cells were rod-shaped (Figure 2b).

**Fig.2 (a) Colony morphology of DW-7; (b) Morphology of DW-7 cells (magnification =1000×)**

Molecular biology identification

The DNA fragment of DW-7 was amplified by PCR and detected by agarose gel electrophoresis. A phylogenetic tree was visualized using MEGA 6.0. As shown in Figure 3, the size of the amplified product was about 1,500 bp, which was consistent with that of the target product.

1500bp

**Fig.3 Electrophoresis results of DW-7**

DW-7 had the closest relationship with *Bacillus velezensis* at 97.81% through comparing data in the NCBI database (Figure 4).

**Fig.4 Phylogenetic tree of DW-7**

Physiological and biochemical test results

DW-7 presented a positive Catalase test, which was able to use D-mannitol to produce acid, survive on a medium containing 10% NaCl or at pH 5.0. It could not use citrate but propionate was available (Table 3). According to the physiological and biochemical test results, as well as 16S rDNA sequence analysis, DW-7 was validated as *Bacillus velezensis*.

**Table 3. Physiological and biochemical tests of DW-7 strain**

| Experiment | Result |
| --- | --- |
| Tolerance to 10% NaCl | + |
| Tolerance to pH 5.0 | + |
| D-mannitol acid production | + |
| Catalase test | + |
| Propionate | + |
| Citrate | - |

+ was positive result, and – was negative result.

**Genome assembly of DW-7**

WGS of DW-7 was performed using the PacBio sequencing platform, and the results showed that there were 193,753 effective reads after filtration, with the mean sequence length at 8380.3 bp (Table 4).

**Table 4 Genome statistics of DW-7**

| **Type** | **Length** | **Amount** |
| --- | --- | --- |
| Sequence | 3942829 bp | 1 |
| Gene | 3402822 bp | 3662 |
| GC content | 46.46% |  |
| Total transposon length | 4515 bp | 64 |
| Long scattered sequence | 1869 bp | 24 |
| Short scattered sequence | 1474 bp | 22 |
| Long terminal repeat sequence | 185 bp | 3 |
| tandem repeat sequences | 10086 bp | 147 |
| tRNA | 6639 bp | 86 |
| rRNA | 41367 bp | 37 |
| sRNA | 2227 bp | 17 |
| Gene Islands | 28970 bp | 1 |
| prophage | 90882 bp | 1 |

The DW-7 chromosome genome formed a circular genome at 3,942,829 bp, and GC accounted for 46.46%. Protein-encoding genes were predicted using the NCBI database. A total of 3,662 genes were obtained, with the total length of 3,402,822 bp, the longest gene length of 16,299 bp, and the shortest gene length of 75 bp. The GC content accounted for 46.46%. There were 64 transposons in the whole genome of DW-7, with the total length of 4,515 bp. There were 24 long interspersed repeats with a total length of 1,869 bp, accounting for 0.05% of the genome, and 22 short interspersed repeats with a total length of 1,474 bp, accounting for 0.04% of the genome. Three long terminal repeats with a total length of 185 bp, and 147 tandem repeats with a total length of 10,068 bp, accounting for 0.26% of the genome were detected. There were 86 tRNAs in the genome of DW-7, with a total length of 6,639 bp and the mean length of 77 bp. Nine 5S rRNAs with a total length of 1,044 bp were detected, with the mean length of 116 bp. Nine 16S rRNAs with a total length of 13,948 bp and the mean length at 1,549 bp were detected. There were 9 23S rRNAs with a total length of 26,375 bp and the mean length of 2,930 bp. A total of 17 sRNAs with the total length of 2,227 bp and the mean length of 131 bp were detected. One genomic island with a total length of 28,970 bp, and 1 prephage with a total length of 90,882 bp were detected.

**Bacterial genome components**

Circular genome maps were depicted based on assembled genome sequences and prediction results of protein-encoding genes, which fully visualized genomic characteristics (Figure 5).

**Fig 5 Complete map of the bacterial DW-7 genome**

The outermost circle of the circular genome maps of DW-7 showed a total length of 3,402,822 bp. The second and third circle were positive and negative-strand coding regions, respectively. Different colors represented different functions annotated by COG database. A total of 3,662 protein-encoding genes were identified, of which 2,796 (76.35%) genes were annotated by COG. Metabolism, information storage and processing, cell function and signal transmission, and atypical features were the 4 main categories. A total of 25 types were annotated, including general function prediction only, amino acid transport and metabolism, unknown function, transcription, carbohydrate transport and metabolism, etc. The fourth circle was ncRNAs, in which tRNAs and rRNAs were colored in black and red, respectively. The fifth circle was the GC content, in which orange and blue indicated that GC content was higher or lower than the mean value in the whole genomes, respectively. The sixth circle was the GC-Skew value. Collectively, circular genome maps of DW-7 directly visualized the distribution of DW-7 genome.

**Gene annotation using COG**

After comparing function annotations between DIAMOND and COG databases, annotations were classified and depicted in Figure 6.

**Fig 6 Functional classification of COG**

Function annotation of identified protein-encoding genes in DW-7 was performed using COG, and a total of 2,796 protein-coding genes involving 25 categories were annotated. Among them, 445 (15.92%) protein-encoding genes were enriched in the general function prediction only, which was the most enriched category, followed by 336 (12.02%) enriched in the amino acid transport and metabolism. In addition, there were 274 (9.80%) protein-encoding genes enriched in transcription, 245 (8.76%) enriched in carbohydrate transport and metabolism and 205 (7.33%) enriched in inorganic ion transport and metabolism. A total of 302 (10.80%) protein-encoding genes were enriched in functional positions, which need to be further explored.

**Gene annotation using KEGG**

The amino acid sequence of DW-7 was compared with data obtained from the KEGG database, and annotation results were yielded by combining genes of target species and their corresponding function annotation information. It is shown that a total of 2,249 genes in DW-7 were enriched in 23 pathways and 5 major functions of metabolism, genetic information processing, environmental information processing, cell process, and biological system (Figure 7).

**Fig 7 Bacterial gene functional annotation KEGG metabolic pathway**

In the category of metabolic process, 12 pathways were annotated that 242 and 206 genes were enriched in the carbohydrate metabolism and amino acid metabolism pathway, respectively. Four pathways in the category of genetic information processing were annotated, and the translation pathway was the mostly annotated one with 85 genes enriched in. There were 147 and 124 genes enriched in the membrane transport and signal transduction pathway, respectively, which belonged to the category of environmental information processing. In addition, 3 pathways were annotated in the category of cell process, in which the prokaryotic cell community was the mostly annotated one with 85 genes enriched in. In the category of biological system, 4 and 3 protein-encoding genes were enriched in the environmental adaptation and the immune system, respectively.

According to the KEGG annotation information, 196 protein-encoding genes in DW-7 were enriched in the following pathways that were highly correlated with proteases, including the metabolism of alanine, aspartic acid, and glutamate, metabolism of glycine, serine, and threonine, ABC transporter and transporter pathways. These protein-encoding genes included the KSG66-11125 (serine dehydratase), KSG66-13075 (aspartate kinase), KSG66-14990 (serine kinase), KSG66 -10425 (aspartate aminotransferase), etc.

**Gene annotation using nr and Swiss-Prot**

The gene sequence of DW-7 was translated into the corresponding amino acid sequence, and compared with data from the nr database (Figure 8).

Fig 8 Annotated species statistics of Nr database (top 20 species)

A total of 3,662 genes in DW-7 were annotated using nr database. Among them, there were 2,709, 389, 198, 107, 70, 53 and 41 genes enriched in the top 7 species of *Bacilus velezensis*, *Bacillus amyloliquefaciens*, *Bacillus*, *Bacillus subtilis*, *Mycobacteroides abscessus*, *Bacillus sp.5B6* and *Streptococcus pneumoniae*.

Swiss-Prot is a selected protein sequence database that describes protein function, structure, post-translational modification, mutation, etc. A total of 3,399 genes in DW-7 were functionally annotated in this database.

**Gene annotation using GO**

According to the annotation information in nr, GO function annotations were obtained to comprehensively describe the attributes of genes and gene products in DW-7 (Figure 9).

Fig 9 GO classification of bacterial gene function annotation

A total of 2,127 genes in DW-7 were annotated in the GO database. GO terms include the biological process (BP), cellular component (CC), and molecular function (MF), in which, there are 18, 10, and 10 elements, respectively. The top 3 annotated BPs were the metabolic process, biological process and single-cell tissue process, in which 1,287, 1,189 and 944 genes were enriched in, respectively. There were 647, 528, 527, and 527 genes enriched in the top 4 CCs, including the cell membrane, cell membrane composition, cell, and genes related with cell components, respectively. A total of 1,152, 888, and 133 genes were enriched in the mostly annotated MFs of the catalytic activity, gene and binding, and transport activity, respectively.

**Conclusion**

In the present experiment, 296 strains of protease-producing bacteria were isolated from the yeast for making hard liquor of Zhanggong Laojiu. After calculating D/d and Folin-Ciocalteu method, the protease activity of the isolated DW-7 was 99.54 U/ mL. Later, we examined the morphology, and identified through 16S rDNA sequence analysis and physiological and biochemical tests, DW-7 was determined to be *Bacillus velezensis*. WGS analysis showed that the genome of DW-7 was 3,942,829 bp long, and among them, GC accounted for 46.45%. Annotated using COG, there were 2,796 protein-encoding genes in DW-7 mainly enriched in the general function prediction only, amino acid transport and metabolism, transcription, carbohydrate transport and metabolism, and inorganic ion transport and metabolism. There were 2,283 genes annotated in the KEGG database, which were mainly enriched in the carbohydrate metabolism, amino acid metabolism, translation, membrane transport and signal transduction pathways. A total of 2,127 genes in the GO database were annotated, which were mainly enriched in the metabolic function, cellular process, cell membrane, cell membrane components, catalytic activity, and gene and binding.

Research of high-yield proteases from the yeast for making hard liquor of the Chinese Luzhou-flavor liquor has been rarely reported. Yuan et al. ^[14]^ isolated and purified 6 protease-producing bacteria from the yeast for making hard liquor of the Chinese Luzhou-flavor liquor, with the maximum protease activity at 53.40 U/mL. Zhao et al.(2015) ^[32]^ identified a strain 3J-1 with a relatively high protease activity at 70.15 U/mL. The protease activity of DW-7 screened in the present study was 99.54 U/mL, which was at a high level. Proteases are widely found in animals, plants and microorganisms, which catalyze the hydrolysis of proteins into polypeptides and amino acids. During the process of liquor brewing, polypeptides and amino acids formed by protease degradation of proteins pose a significant influence on the fermentation of yeasts and lactic acid bacteria, and thus influence the flavor of liquor. Proteases are categorized into serine proteases, metalloproteases, ;cysteine proteases and aspartic proteases based on their mechanisms targeting on substrates and the composition of active centers. Here, WGS analysis of DW-7 obtained 196 genes that were highly related to proteases, which were mainly enriched in the metabolism of alanine, aspartic acid, and glutamate, the metabolism of glycine, serine, and threonine, ABC transporter and transporter pathway.

Taken together, our study preliminarily explored the biological characteristics and metabolic functions of the high-yield protease-producing strain DW-7 by WGS analysis. Abundant genomic information was obtained that provided massive references for identifying the functional characteristics of the strain, and the foundation for elucidating the background of the high-yield protease-producing feature. Our results are of great significance to develop novel high-yield proteases in the yeast for making hard liquor.

**Acknowledgments**

This work supported by the Major Science and Technology Projects of Henan Province of China(181100211400),Key Technologies Research and Development Program of Henan Province of China(202102110130) , Scientific Research Foundation for Docotors of Henan University of Animal Husbandry and Economy(2018HNUAHEDF011) and Key Subject Projects of Henan University of Animal Husbandry and Economy.

**References**

[1]Zhou R, ChenNY, Tang D. Preliminary Research on the Diversity & the Distribution of Microorganism in Multiple-grains Luzhou-flavor Distilleries in Yibin[J]. Liquor-Making Science & Technology, 2010, 4.

[2]Liu J,Chen J,Fan Y,etal. Biochemical characterisation and dominance of different hydrolases in different types of Daqu–a Chinese industrial fermentation starter[J]. Journal of the Science of Food and Agriculture, 2018, 98(1): 113-121.

[3]Fan G, Fu Z, Sun B, et al. Roles of aging in the production of light-flavored Daqu[J]. Journal of bioscience and bioengineering, 2019, 127(3): 309-317.

[4]Yang J G, Dou X, Ma Y Y. Diversity and dynamic succession of microorganisms during Daqu preparation for Luzhou‐flavour liquor using second‐generation sequencing technology[J]. Journal of the Institute of Brewing, 2018, 124(4): 498-507.

[5]Yin X, Yoshizaki Y, Ikenaga M, et al. Manufactural impact of the solid-state saccharification process in rice-flavor baijiu production[J]. Journal of bioscience and bioengineering, 2020, 129(3): 315-321.

[6]Zhang Y, Huang Y, Qiu S. Solid-state fermentation condition and its optimization of protease production by Geotrichum candidum in Moutai-flavor Daqu [J]. China Brewing, 2012, 3.

[7]Fan G, Sun B, Fu Z, et al. Analysis of physicochemical indices, volatile flavor components, and microbial community of a light-flavor Daqu[J]. Journal of the American Society of Brewing Chemists, 2018, 76(3): 209-218.

[8]Wu Q, Chen L, Xu Y. Yeast community associated with the solid state fermentation of traditional Chinese Maotai-flavor liquor[J]. International journal of food microbiology, 2013, 166(2): 323-330.

[9]Tyler A D, Mataseje L, Urfano C J, et al. Evaluation of Oxford Nanopore’s MinION sequencing device for microbial whole genome sequencing applications[J]. Scientific reports, 2018, 8(1): 1-12.

[10]McDermott P F, Tyson G H, Kabera C, et al. Whole-genome sequencing for detecting antimicrobial resistance in nontyphoidal Salmonella[J]. Antimicrobial agents and chemotherapy, 2016, 60(9): 5515-5520.

[11]Ronholm J, Nasheri N, Petronella N, et al. Navigating microbiological food safety in the era of whole-genome sequencing[J]. Clinical microbiology reviews, 2016, 29(4): 837-857.

[12]Lu Z,Yan J,Li E Z, *et al*. The screening of high-yielding neutral protease bacteria and the conditions of their enzyme production[J]. Heilongjiang Agricultural Science, 2016 (05): 110-114.

[13]Nie H F, Jiang Y L, Shen Y, *et al.* The separation and identification of high-yielding protease bacteria in the sauce-scented large-shaped large-scale song[J]. Brewing Technology, 2015 (12): 41-44.

[14] Yuan X L, Huang D, Peng J. Isolation of Protease-producing Bacteria Strains from Luzhou-flavor Daqu and Study on Its Protease-producing Conditions[J]. Liquor-Making Science & Technology, 2012: 08.

[15]Zhong X M, Fu J, Lan J, *et al*. Comparison of protease conditions and protease properties in Actinomucor elegans, Aspegillus oryzae and Rhizopus oligosporus[J]. Food and Fermentation Industry, 2009, 35 (05): 40-44.

[16]Wang J, Wang X D, Luo X Y, *et al.* Screening and identification of high-yielding protease functional bacteria in soy sauce[J]. China Brewing, 2015, 34 (10): 43-46.

[17] Wei C H, Huang Z G, Huang D, *et al*. The isolation identification of high-temperature large-yielding protease bacteria and its enzyme properties study[J]. Food and Machinery, 2014 (4): 24 - 29.

[18] Ming H M, Dong R L, Xu D F, *et al.* The separation and preliminary identification of the dominant bacteria in the strong-scented large-shaped large-scale song[J]. Brewing Technology, 2013 (12): 57-60.

[19] Wang P P. The screening of protease-producing bacteria and the study of solid fermentation to produce high-quality protein raw materials[J]. Master's degree thesis from Henan Agricultural University, 2010.

[20]Xiao W, Wang X D, Ban S D, *et al.*. Screening and identification of neutral protease heat-loving bacteria in soy sauce-scented daqu[J]. Brewing Technology, 2015(02): 50-53.

[21] Wang Y T, Zheng R F, Han M Y, *et al.* Screening and identification of protease -producing [J]. amylase spore spore. Feed Expo, 2018 (03): 4-7.

[22]Faja O M, Sharad A A, Younis K M, *et.al.* Isolation, detection of virulence genes, antibiotic resistance genes, plasmid profile, and molecular typing among Vibrio parahaemolyticus isolated in Malaysian seawater from recreational beaches and fish.[J]. Veterinary world, 2019, 12(7).

[23] Stackebrand T E, Goebei B M. Taxonomic note: a place for DNA-DNA reassociation and 16S rRNA sequence analysis in the present species definition in bacteriology[J]. International Journal of Systematic and Evolutionary Microbiology, 1994, 44(4): 846-849.

[24]Dvorak H F, Orenstein N S, Dvorak A M, *et.al.* Isolation of the cytoplasmic granules of guinea pig basophilic leukocytes: identification of esterase and protease activities.[J]. Journal of Immunology, 1977, 119(1).

[25] Benson G. Tandem repeats finder: a program to analyze DNA sequences[J]. Nucleic acids research, 1999, 27(2): 573-580.

[26] Lowe T M, Chan P P. tRNAscan-SE On-line: integrating search and context for analysis of transfer RNA genes[J]. Nucleic acids research, 2016, 44(W1): W54-W57.

[27] Lagesen K, Hallin P, Rødland E A, et al. RNAmmer: consistent and rapid annotation of ribosomal RNA genes[J]. Nucleic acids research, 2007, 35(9): 3100-3108.

[28] Nawrocki E P, Eddy S R. Infernal 1.1: 100-fold faster RNA homology searches[J]. Bioinformatics, 2013, 29(22): 2933-2935.

[29] Bertelli C, Brinkman FSL. Improved genomic island predictions with IslandPath- DIMOB. Bioinformatics. 2018 Jul 1;34(13):2161-2167.

[30] Haas B. TransposonPSI: an application of PSI-Blast to mine (retro-) transposon ORF homologies[J]. Broad Institute, Cambridge, MA, USA, 2007.

[31] Fouts D E. Phage_Finder: automated identification and classification of prophage regions in complete bacterial genome sequences[J]. Nucleic acids research, 2006, 34(20): 5839-5851.

[32]Zhao Q L, Wu T X, Liu W. Screening and enzyme live determination of a wine-making bacterium in the sauce[J]. Brewing Technology, 2015 (11): 41-44.
