# Supplementary material for: Isolation, identification, and whole-genome sequencing of high-yield protease bacteria from Daqu of ZhangGong Laojiu
Source: PLoS One. 2022 Apr 26;17(4):e0264677. doi: 10.1371/journal.pone.0264677 (PMC9041807; doi:10.1371/journal.pone.0264677)
Supplement: Attachment — Submitted filename: 20220112-Response to Reviewers.docx [file pone.0264677.s005.docx]

Dear Editor and Reviewers:

Thank you for your letter and for the reviewers' comments concerning our manuscript. Those comments are all valuable and very helpful for revising and improving our paper, as well as the important guiding significance to our researches. But I have a question that I don't know how to modify, and would like to ask you.

1.‘Funding Information’ and ‘Financial Disclosure’ sections do not match.

First of all, our magazine required that there should be no funding information in the manuscript. I wrote the funding information in the cover letter.I understand that this problem is cover letter and submit websites do not match the fund information, then I go to submit sites funding Information sections to add a funding source.but I can't add it.

Could you tell me how to do that or could you help me modify it? In addition,I have provided the correct fund information below.

**Funding Information**

This work supported by the Major Science and Technology Projects of Henan Province of China(181100211400),Key Technologies Research and Development Program of Henan Province of China(202102110130) , Scientific Research Foundation for Docotors of Henan University of Animal Husbandry and Economy(2018HNUAHEDF011) and Key Subject Projects of Henan University of Animal Husbandry and Economy(C3060020). The funders had no role in study design, data collection and analysis, decision to publish, or preparation of the manuscript.

If there are any shortcomings in the article, please tell me immediately, and I will seriously revise it again.Once again, thank you very much for your comments and suggestion.

Thank you and best regards.

Yours sincerely,

Yanbo Liu E-mail: yanboliu@hnuahe.edu.cn
